# Catalytic Lewis Base Additive Enables Selective Copper-Catalyzed Borylative α-C–H Allylation of Alicyclic Amines

**DOI:** 10.1021/jacs.2c07969

**Published:** 2022-08-24

**Authors:** Borja Pérez-Saavedra, Álvaro Velasco-Rubio, Eva Rivera-Chao, Jesús A. Varela, Carlos Saá, Martín Fañanás-Mastral

**Affiliations:** Centro Singular de Investigación en Química Biolóxica e Materiais Moleculares (CiQUS), Departamento de Química Orgánica, Universidade de Santiago de Compostela, 15782 Santiago de Compostela, Spain

## Abstract

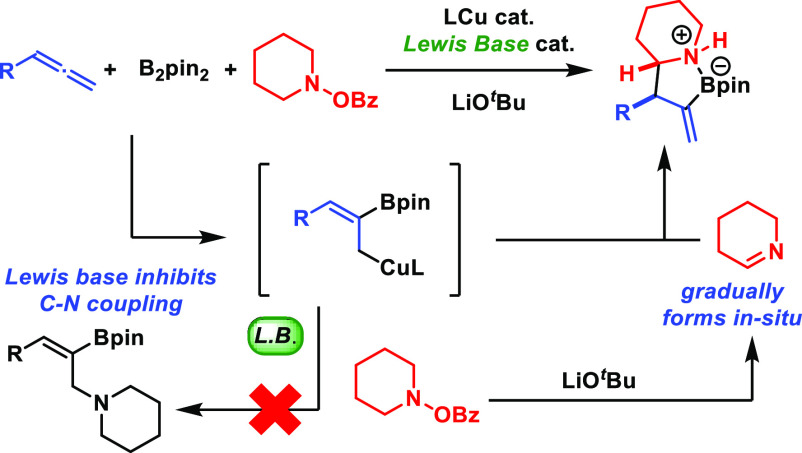

Functionalized alicyclic amines are important building blocks for the synthesis of bioactive natural compounds and drugs. Existing methods of functionalization are typically limited to the synthesis of protected amines or the use of highly basic organometallic reagents that can compromise functional group tolerance. Here, we report a novel approach that enables the transformation of *O*-benzoyl hydroxylamines into α-functionalized cyclic secondary amines by means of a copper-catalyzed regio-, stereo-, and chemoselective coupling with allenes and bis(pinacolato)diboron. A key feature of the present transformation is the use of a catalytic Lewis base additive which inhibits the competing C–N bond forming reaction between the catalytically generated boron-substituted allylcopper intermediate with the *O*-benzoyl hydroxylamine, thus enabling in situ transformation of the latter into an alicyclic imine which undergoes selective C–C bond formation with the allylcopper species.

## Introduction

**Figure 1:**
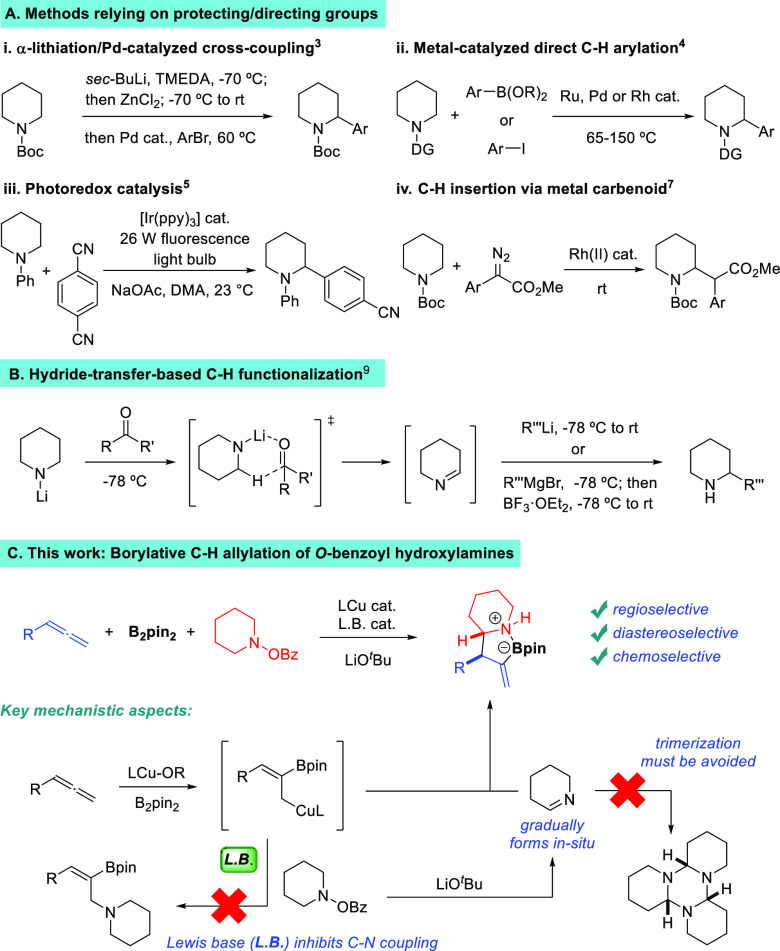
Methods for α-C–H functionalization of alicyclic amines.

Saturated *N*-heterocycles represent one of the most important classes of compounds in drug discovery.^1^ As such, synthetic procedures to diversely functionalize the alicyclic amine framework are in high demand. Among the different methodologies that have been developed for this purpose, a particularly attractive strategy to access substituted saturated azaheterocycles is the α-C–H bond functionalization of cyclic amines.^2^ Despite great advances in the field, current procedures typically rely on the use of a directing group on the nitrogen atom, limiting their utility to the synthesis of tertiary or protected secondary alicyclic amines. A commonly used approach includes the α-lithiation of *N*-Boc-protected azaheterocycles, followed by transmetalation to an organozinc and subsequent palladium-catalyzed Negishi coupling.^3^ Other strategies based on protecting groups have relied on transition metal-catalyzed direct C–H arylation reactions,^4^ photoredox catalysis,^5^ intramolecular hydride transfer,^6^ and C–H insertions via metal carbenoids^7^ (Figure 1A).

An alternative strategy to generate α-substituted saturated nitrogen heterocycles is the generation of a cyclic imine and its engagement with a nucleophile. However, alicyclic imines are unstable, and they tend to undergo trimerization, resulting in nonreactive compounds.^8^ Recently, Seidel and co-workers reported a method for the in situ generation of alicyclic imines based on the reduction of a sacrificial ketone hydride acceptor by the corresponding *N*-lithiated amine.^9^ The cyclic imine can then be trapped by an organolithium compound^9a^ or a Grignard reagent using Lewis acid activation,^9b^ thus resulting in a net α-C–H functionalization (Figure 1B). Although this method represents an interesting entry to α-functionalized secondary alicyclic amines, cryogenic temperatures are required, and the stoichiometric use of organometallic reagents can impose extra steps associated with the preparation and purification of these sensitive compounds. Moreover, in some cases, their high basicity can compromise its compatibility with certain functional groups.

An attractive way to produce α-functionalized alicyclic amines would be the catalytic transformation of an unsaturated hydrocarbon into an organometallic intermediate, which can then react with an alicyclic imine. Following this idea, we focused on the development of a catalytic transformation based on the in situ generation of boron-substituted allyl-copper species by addition of Cu-Bpin to an allene.^10,11^ Given the inherent instability of saturated cyclic imines, a catalytic carboboration process involving in situ formation of this electrophilic coupling partner would be highly desirable. Inspired by the work of You and co-workers, where they showed that *O*-benzoyl cyclic hydroxylamines can evolve into the corresponding cyclic imines under basic conditions,^12^ we envisioned a borylative coupling of allenes with B_2_pin_2_ and cyclic *O*-benzoyl hydroxylamines where the catalytic allylcopper intermediate should selectively react with the cyclic imine generated from the *O*-benzoyl hydroxylamine (Figure 1C). Three-component carboboration of allenes typically involve several challenges associated with the regio- and stereoselective generation of the catalytic Bpin-substituted allyl copper intermediate and the control over the stereo- and site selectivity of its electrophilic trapping. Furthermore, the proposed transformation imposes an important additional selectivity issue since it requires a catalytic system capable of generating an allyl copper intermediate reactive enough to promote an efficient C–C bond formation with the imine while precluding the direct C–N coupling with the *O*-benzoyl hydroxylamine precursor. Transient organocopper reagents have indeed been reported to react with *O*-benzoyl hydroxylamines,^13^ and that reactivity must be shut down. Several requirements must be met to achieve this successfully. The rate of the reaction between the allylcopper intermediate and the cyclic *O*-benzoyl hydroxylamine must be slower than the rate of imine formation. Moreover, the imine trapping by the allylcopper intermediate, i.e., the C–C bond forming step, must be fast enough to avoid imine trimerization. We here report the successful implementation of this idea and thus the development of a catalytic process that allows for the selective synthesis of boron-substituted α-allylated cyclic secondary amines. We have found that the use of a catalytic Lewis base additive in combination with a Cu/bisphosphine catalyst is crucial to inhibit the C–N coupling and thus to enable an efficient imine trapping.

## Results and Discussion

### Preliminary Studies and Optimization of the Reaction Conditions

We began our studies by surveying the reaction between phenylallene **1**, B_2_pin_2_ and morpholino benzoate **2** (Table 1).^14^ Initial experiments using several copper catalysts already showed the challenging nature of this multicomponent reaction. The use of monodentate phosphine ligands provided almost exclusively the C–N coupling product **4** in low yields together with regioisomeric mixtures of allene protoboration^15^ products **5** and **6** (entries 1–2). With NHC–Cu complexes derived from sterically bulky aryl-substituted heterocyclic ligands, chemoselectivity improved, favoring the C–C bond formation, although α-functionalized morpholine **3** was obtained in very low yields (entries 3 and 4). We then turned to evaluate bidentate phosphines and found that large bite-angle phosphines favored the formation of product **3** (entries 5–9), although with yields and selectivity still far from satisfactory.

**Table 1:**
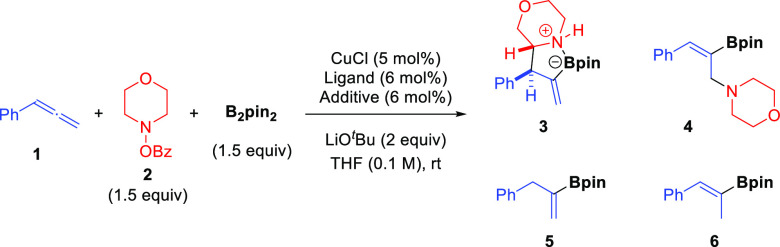
Optimization Studies

entry^a^	ligand	additive	**3** (%)^b^	**4** (%)^b^	**5** (%)^b^	**6** (%)^b^
1	PPh_3_		<5	30	30	25
2	PCy_3_		15	42	23	14
3	IMes		24	36	19	<5
4	IPr		22	<5	5	<5
5	Xantphos		30	<5	30	<5
6	DPEphos		50	24	25	<5
7	dppf		35	14	12	<5
8	dppe		11	<5	<5	<5
9	dppp		40	18	20	<5
10	dcpe		25	16	12	<5
11	dcpe*^c^		60	<5	<5	15
12	dcpe	P(*O*)Ph_3_	72	<5	<5	8
13	dcpe^d^		35	<5	<5	<5
14			<5	30	22	<5
15		P(*O*)Ph_3_	<5	8	6	<5
16^e^	dcpe	P(*O*)Ph_3_	60	<5	<5	10
17^f^	dcpe	P(*O*)Ph_3_	23	28	30	15

a Reactions run on a 0.3 mmol scale. Diastereomeric ratio of **3** >95:5 in all cases.

b Determined by ^1^H NMR analysis using 1,3,5-trimethoxybenzene as the internal standard.

c dcpe* = dcpe:dcpe(O):dcpe(O)_2_ in a 3:1:3 ratio.

d 12 mol %.

e NaO^*t*^Bu used instead of LiO^*t*^Bu.

f NaOMe used instead of LiO^*t*^Bu.

An intriguing observation was that the use of different batches of bis(dicyclohexylphosphino)ethane (dcpe) resulted in drastically different results (entries 10 and 11). Analysis of these two batches revealed that the use of pure dcpe led to a mixture of C–C coupling product **3** and C–N coupling product **4** in a 1.5:1 ratio (entry 10). However, the use of a partially oxidized sample consisting of a 3:1:3 mixture of dcpe, dcpe monoxide (dcpe(O)), and dcpe bis(oxide) (dcpe(O)_2_) provided **3** with total chemoselectivity as a single diastereomer in 60% yield (entry 11). Motivated by this finding, we surveyed the cooperative effect of several Lewis bases with the Cu/dcpe catalyst (see the Supporting Information, Tables S2 and S3).^16^ Among the Lewis bases tested, the use of catalytic amounts of P(*O*)Ph_3_ provided the best results. The optimized CuCl/dcpe/P(*O*)Ph_3_ catalyst allowed for almost full control with respect to side reactions and provided the α-functionalized product **3** as a single isomer in 72% yield with total diastereocontrol (entry 12). Excess of dcpe ligand also led to the selective formation of **3**, although in a diminished yield (entry 13). Importantly, in the absence of a phosphorous ligand, selectivity was switched toward C–N coupling product **4**, which was obtained in 30% yield (entry 14). The same selectivity was observed when a catalytic amount of P(*O*)Ph_3_ was used, although **4** was obtained in an almost negligible yield (entry 15). The choice of base was also important to achieve high levels of selectivity. Indeed, while the use of NaO^*t*^Bu led to a similar result, the use of NaOMe instead or LiO^*t*^Bu caused a significant drop in the chemoselectivity, providing a mixture of C–C and C–N coupling products (entries 16–17).

### Substrate Scope and Structural Modification of Products

**Figure 2:**
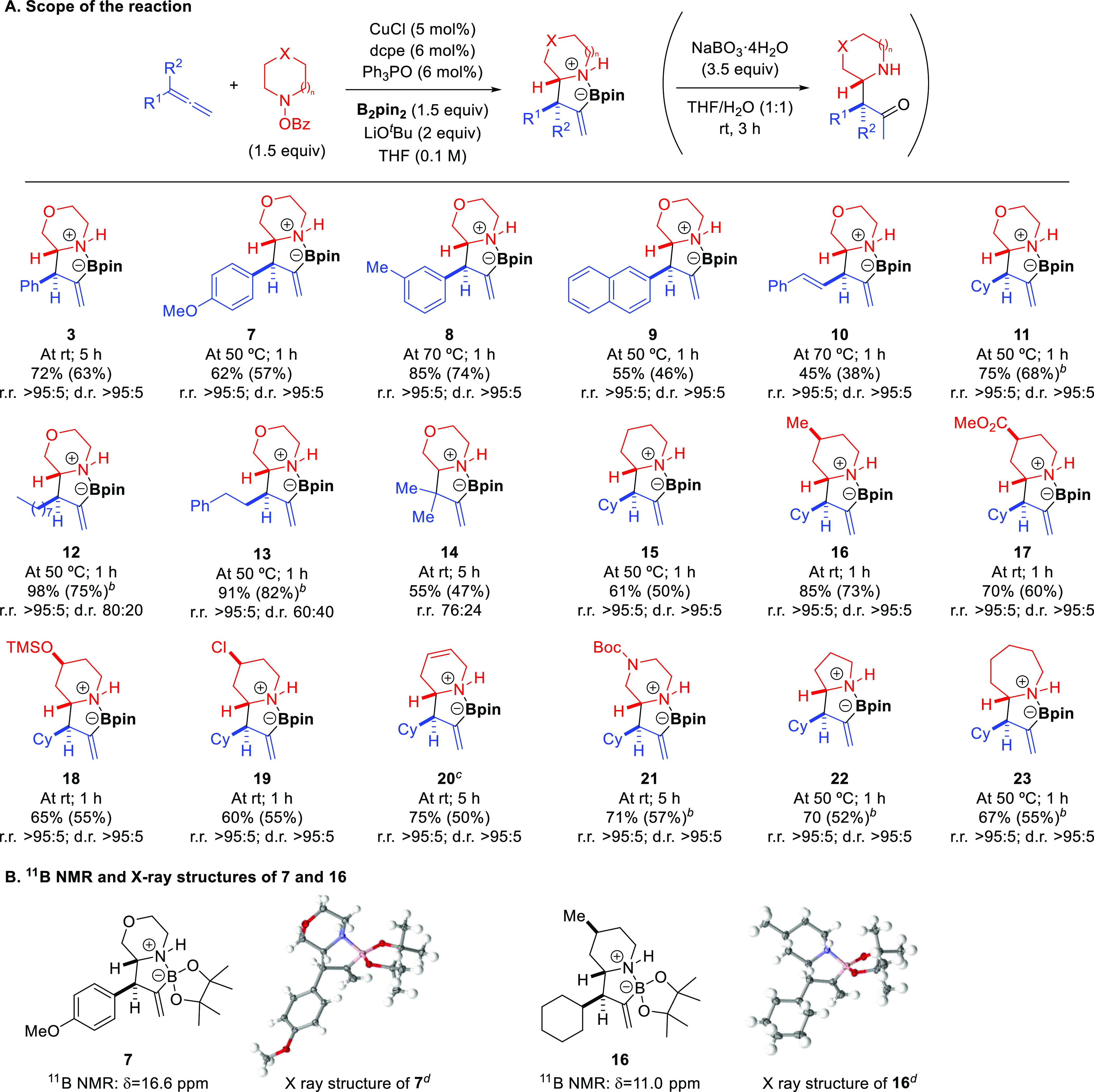
(A) Scope of the copper-catalyzed borylative α-allylation of *O*-benzoyl cyclic hydroxylamines. ^*a*^Reactions run on a 0.3 mmol scale. Regioisomeric ratios (r.r.; C–C vs C–N coupling) and diastereomeric ratios (d.r.) were determined by ^1^H NMR analysis. Yields of isolated products are shown in brackets. ^*b*^Those values refer to the oxidized product. ^*c*^Synthesized from 4-(acryloyloxy)piperidin-1-yl benzoate. (B) ^*d*^Ellipsoids shown at 50% probability.

Having established optimized conditions, we set out to explore the scope of this three-component borylative α-C–H allylation (Figure 2A). In general, the reaction proved to be remarkably effective for a wide range of allenes and cyclic amines, providing the corresponding α-functionalized alicyclic amines with excellent levels of regio- and stereocontrol. Common functional groups such as esters, silyl ethers, alkenes, halides, and carbamates were well tolerated under the reaction conditions. In some cases, alkenyl boronates were unstable to silica gel and an additional oxidation step was carried out for a more facile purification of the resulting ketone. Allenes bearing aryl (**3**, **7**–**9**), alkenyl (**10**), and aliphatic (**11**–**14**) groups proved to be efficient substrates for this three-component coupling. Remarkably, full diastereoselectivity was achieved in nearly all cases, and it was only eroded when allenes bearing linear alkyl substituents (**12**, **13**) were used. Notably, an allene bearing a conjugated alkenyl group, which adds extra points of reactivity, could be used without formation of side products arising from competitive Cu-Bpin olefin insertion or vinylogous S_E_2″ trapping of the organocopper intermediate. Although this allene showed diminished reactivity, the reaction still afforded product **10** with complete regio- and diastereoselectivity. 1,1-Disubstitution in the allene caused a slight decrease on the regioselectivity (r.r. = 76:24), although it allowed to obtain pure α-allylated amine **14** bearing contiguous quaternary and tertiary stereocentres. Gratifyingly, this method could be extended to other alicyclic amine frameworks such as piperidine (**15**–**20**), piperazine (**21**), pyrrolidine (**22**), and azepane (**23**), obtaining in all cases the product in good yield with perfect regio- and diastereoselectivity. Of note is the reaction with 4-substituted piperidine derivatives, which afforded α-functionalized products **16**–**19** with total stereocontrol over the three newly created stereocenters. Interestingly, the use of an acrylate-substituted piperidine benzoate allowed for the synthesis of endocyclic olefin **20** through an in situ elimination process (see the Supporting Information for details). Relative configuration of products was assigned based on the X-ray crystallographic analysis of products **7** and **16** (Figure 2B). Interestingly, it was also observed that these products exist as azaboraspiro compounds where the boron center features a sp^3^-hybridization resulting from a dative nitrogen–boron coordination. ^11^B NMR analysis of products **3** and **7**–**23** (^11^B: δ = 10–16 ppm) also suggests the presence of this interaction in solution.

**Figure 3:**
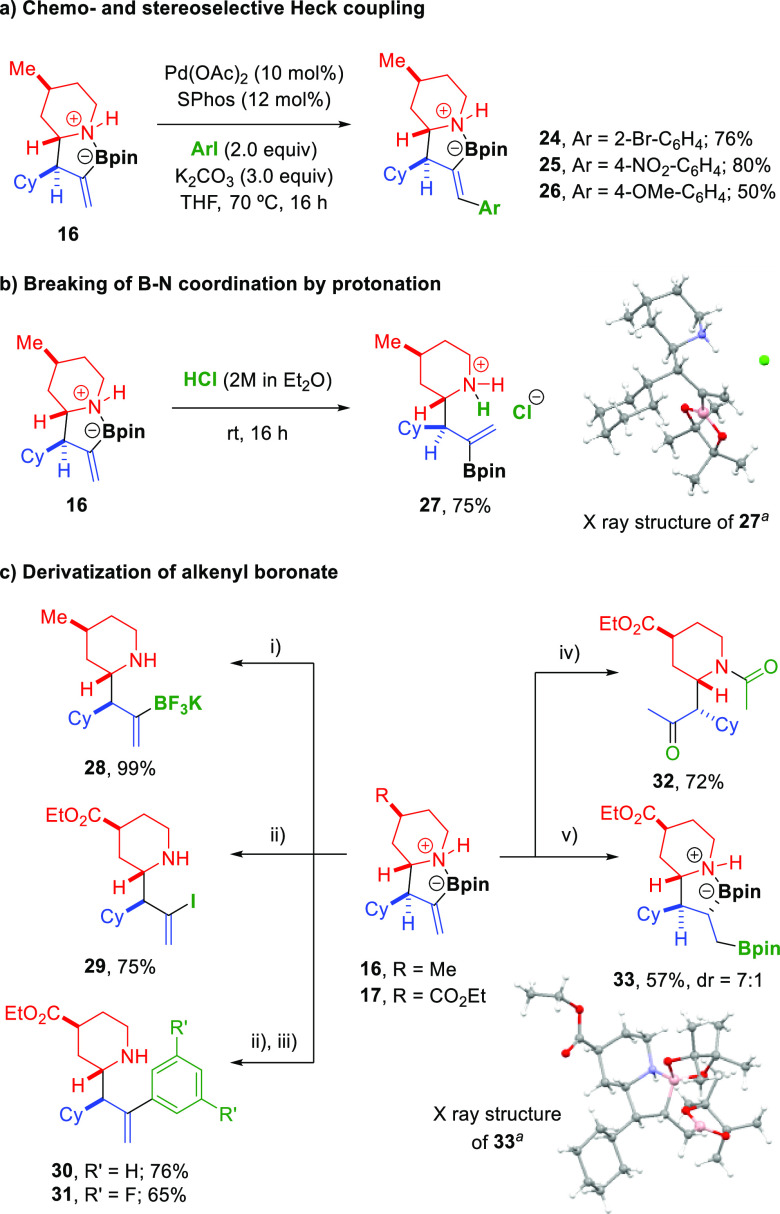
(A–C) Structural modification of products. (i) KHF_2_ (4.5 equiv), MeOH/H_2_O 2:1, rt, 3 h. (ii) NIS (1.5 equiv), THF, rt, 2 h. (iii) ArB(OH)_2_ (1.5 equiv), Pd(PPh_3_)_4_ (5 mol %), K_2_CO_3_ (3 equiv), 1,4-dioxane/H_2_O 6:1, 100 °C, 2 h. (iv) Cu(OAc)_2_ (1 equiv), Et_3_N (2 equiv), 4 Å MS, MeCN, 80 °C, 20 h. (v) ICyCuCl (10 mol %), NaO^*t*^Bu (1.2 equiv), B_2_(pin)_2_ (1.5 equiv), THF, rt, 16 h. ^*a*^ Ellipsoids shown at 30% probability.

An important feature of the present methodology is that it allows the introduction of a functionalized allyl group at the amine ring, which can be diversely modified to access more complex structures in a straightforward manner. Remarkably, the nitrogen–boron coordination present in the α-allylated alicyclic amines served as the basis for a chemoselective structural modification. Reaction of **16** with different aryl iodides catalyzed by a Pd/SPhos complex afforded Heck coupling products **24**–**26** in good yields with total *E*-selectivity (Figure 3a). Importantly, the alkenylboronate ester group remained intact and no traces of Suzuki cross-coupling products were detected.^17^ This excellent chemoselectivity may be explained by the effect of the B–N coordination, which likely hampers transmetalation, a key step in the Suzuki cross-coupling, thus favoring the double bond insertion into the Pd(II)–Ar intermediate, which guides the reaction through the Heck coupling pathway. Interestingly, the B–N coordination could be broken by protonation of the nitrogen atom with hydrochloric acid, which afforded salt **27** (Figure 3b). Despite the strong B–N coordination, the boronic ester moiety could be readily transformed into the corresponding alkenyl trifluoroborate **28** and alkenyl iodide **29** by treatment with KHF_2_ and NIS, respectively (Figure 3c). C–C bond formation was also possible through an iodination/Suzuki coupling sequence as illustrated with the synthesis of products **30** and **31**. Curiously, treatment of compound **17** with Cu(OAc)_2_ under Chan–Lam conditions^18^ did not result in the azetidine formation but produced N-acetylated ketone **32**, which may result from a copper-promoted C–B acetoxylation followed by an intramolecular acetyl transfer (see the Supporting Information for details). The alkenyl boronate moiety was also used as a handle to install another boron functionality in the carbon chain by means of a site-selective copper-catalyzed protoboration.^19^ Thus, by using ICyCuCl as the catalyst, reaction of compound **17** with B_2_pin_2_ and NaO^*t*^Bu in tetrahydrofuran (THF) resulted in the diastereoselective formation of vicinal diboronate **33**.

### Mechanistic Investigations

The most intriguing observation done during our optimization studies was the high level of chemoselectivity (C–C vs C–N coupling) achieved in this transformation when a catalytic amount of a Lewis base (i.e., P(*O*)Ph_3_) was used. It is important to note that this effect was only observed for the dcpe and dppe ligands (see the Supporting Information, Table S4), thus pointing at a special behavior of the resulting copper catalysts. To gather insight about the origin of these high levels of chemoselectivity, density functional theory (DFT) calculations were performed using the coupling of allene **1**, morpholino benzoate **2**, and B_2_pin_2_ as the model reaction (see the Supporting Information for details).

**Figure 4:**
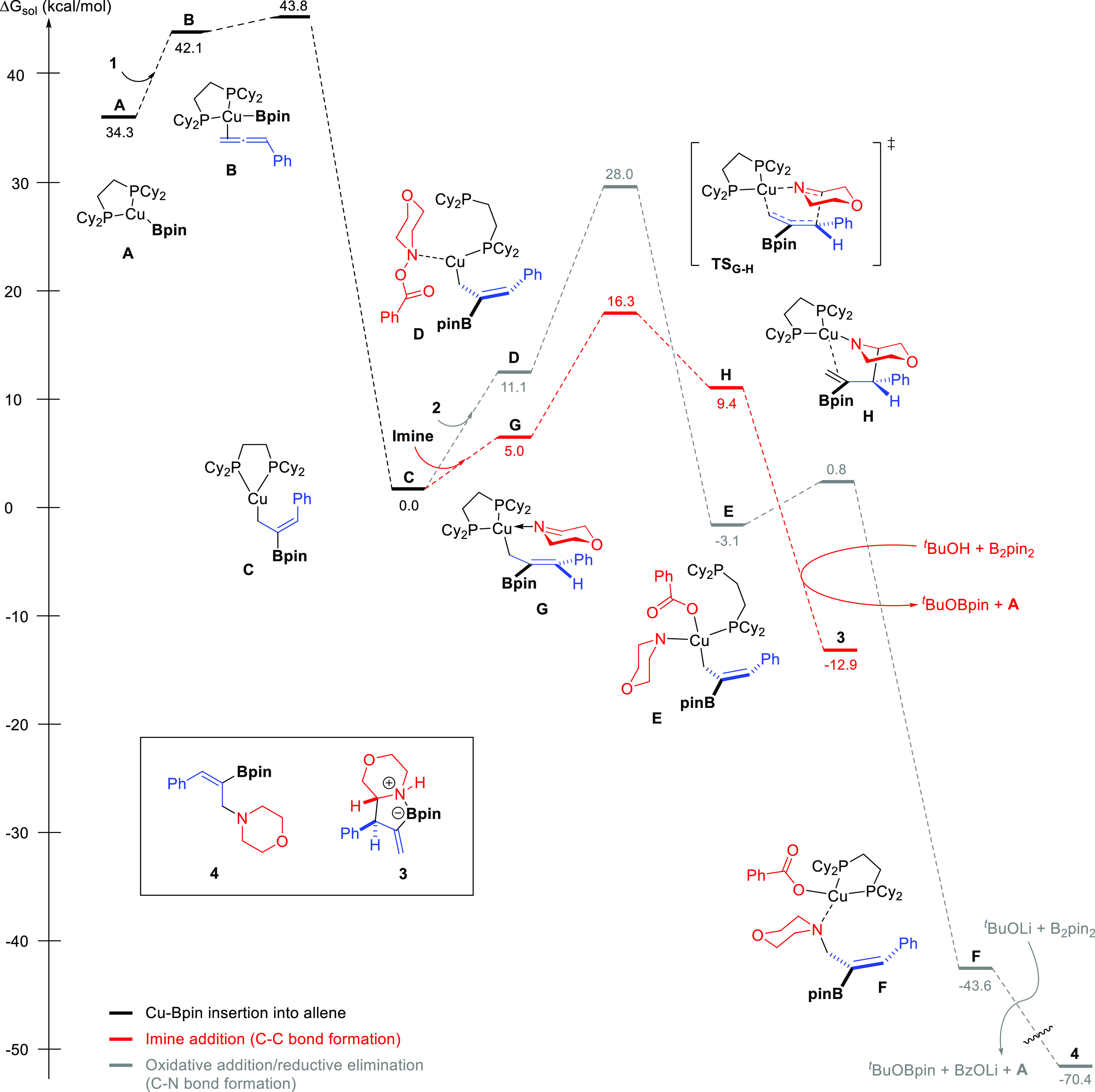
Free energy profile for the most favored pathways leading to the formation of C–C coupling product **3** and C–N coupling product **4** involving Cu/dcpe intermediates. Computational studies were performed at the B3LYP-D3/6-311++G(d,p)-SDD_THF(SMD)_//B3LYP-D3/6-31G(d)-SDD level of theory. Energies are relative to complex **C** combined with those of the relevant substrates.

Based on literature precedents,^10,11^ formation of allylcopper intermediate **C** would proceed through regio- and stereoselective insertion of **1** into Cu(dcpe)Bpin complex **A**. Indeed, calculations showed that this step features a low activation energy barrier of 9.5 kcal/mol (Figure 4). We then investigated the evolution of **C** either to C–N coupling product **4** or C–C coupling product **3** (Figure 4). For the formation of C–N coupling product **4**, we initially considered the oxidative addition of the N–O bond of **2** into copper complex **C** followed by reductive elimination (Figure 4, gray pathway). Note that the dcpe ligand readily dissociates one phosphine unit upon Cu–N coordination to generate intermediate **D**. Benzoate-assisted S_N_2-type oxidative addition^20^ into **D** features a high energy barrier (Δ*G*^‡^ = 28.0 kcal/mol) and results in intermediate **E**, which would undergo facile reductive elimination (Δ*G*^‡^ = 3.9 kcal/mol). An alternative pathway for the formation of C–N coupling product **4** involving the formation of the branched isomeric allylcopper species by metallotropic rearrangement^15b^ of **C** and subsequent S_E_2′ substitution with morpholino benzoate **2** (see the Supporting Information, Figure S18) was found to be an even more kinetically demanding process (Δ*G*^‡^ = 29.4 kcal/mol). We then explored the reaction between allylcopper intermediate **C** and the cyclic imine generated from **2** (Figure 4, red pathway). The most favorable route for this transformation involves a six-membered transition state (**TS_G-H_**) in which copper coordinates the imine nitrogen and which features an energy of only 16.3 kcal/mol.^21^ Other possible pathways such as half-chair-like transition structure involving coordination of the imine nitrogen atom to boron were found to be much higher in energy (see the Supporting Information, Figure S22). The fact that the pathway for imine trapping is much more favorable than the oxidative addition/reductive elimination pathway raises the question of why a mixture of C–C coupling product **3** and C–N coupling product **4** is obtained in the absence of a Lewis base (Table 1, entry 10). By spectroscopic studies, we observed that the alicyclic imine is formed gradually during the reaction (see the Supporting Information for details). Furthermore, DFT calculations showed that this transformation features an energy barrier of 16.6 kcal/mol (see the Supporting Information, Figure S23). Thus, the initial concentration of imine might be low to produce product **3** at the beginning of the reaction, thus allowing competing reactions to take place. However, the energetic barrier for the N–O bond oxidative addition in the Cu/dcpe system is too high to make this pathway competitive at the working temperature.^22^

**Figure 5:**
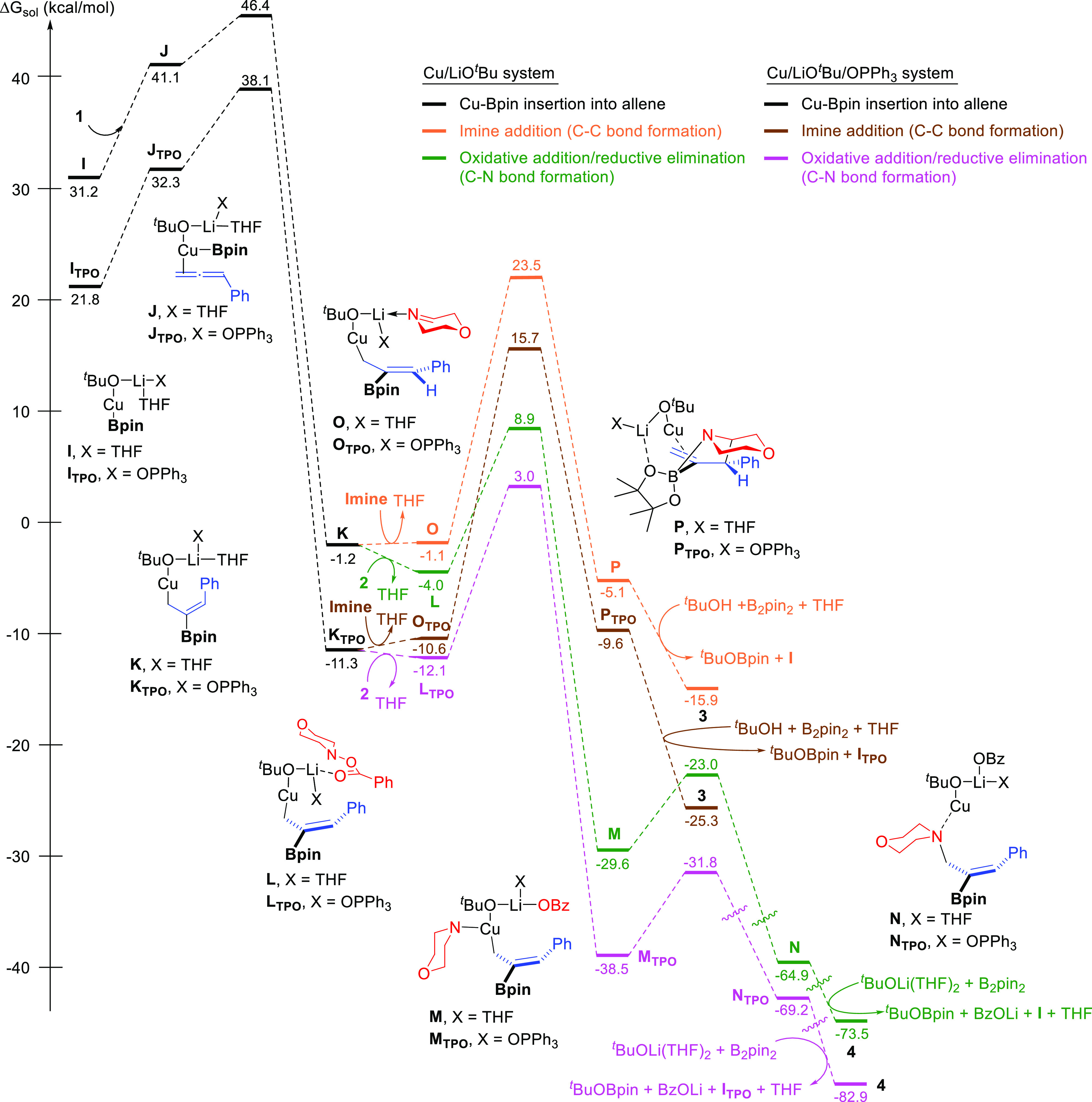
Free energy profile for the pathways leading to the formation of C–C coupling product **3** and C–N coupling product **4** involving dcpe-free copper systems. Computational studies were performed at the B3LYP-D3/6-311++G(d,p)-SDD_THF(SMD)_//B3LYP-D3/6-31G(d)-SDD level of theory. Energies are relative to complex **C** combined with those of the relevant substrates.

At this point, we envisaged that the formation of other Cu species may be responsible for the formation of the C–N coupling product. Since the absence of a phosphine ligand led to the selective formation of C–N coupling product **4** (Table 1, entry 14), we carried out calculations on the reaction involving phosphine-free copper species (Figure 5). Assuming the formation of Cu(Bpin)(O^*t*^Bu)Li complex **I**,^23^ generated from reaction of CuO^*t*^Bu and B_2_Pin_2_ in the presence of excess of LiO^*t*^Bu, and subsequent allene insertion, we calculated the possible pathways for the resulting allylcopper complex **K**. Inclusion of explicit solvent molecules was done given their importance for calculation of Li complexes.^24^ The pathway involving a disolvated complex was found to be the most favored one (see the Supporting Information, Figure S19) and thus was used for comparison with the Cu(dcpe) system. In this case, after coordination of **2** to **K** to form complex **L**, a lower activation energy barrier of 12.9 kcal/mol was found for the N–O bond oxidative addition, thus representing a feasible pathway for the formation of **4** at the reaction temperature (Figure 5, green pathway). In sharp contrast, the activation energy barrier for the formation of C–C coupling product **3** from imine complex **O** (Figure 5, orange pathway) was significantly higher for this system (Δ*G*^‡^ = 24.6 kcal/mol).

**Figure 6:**
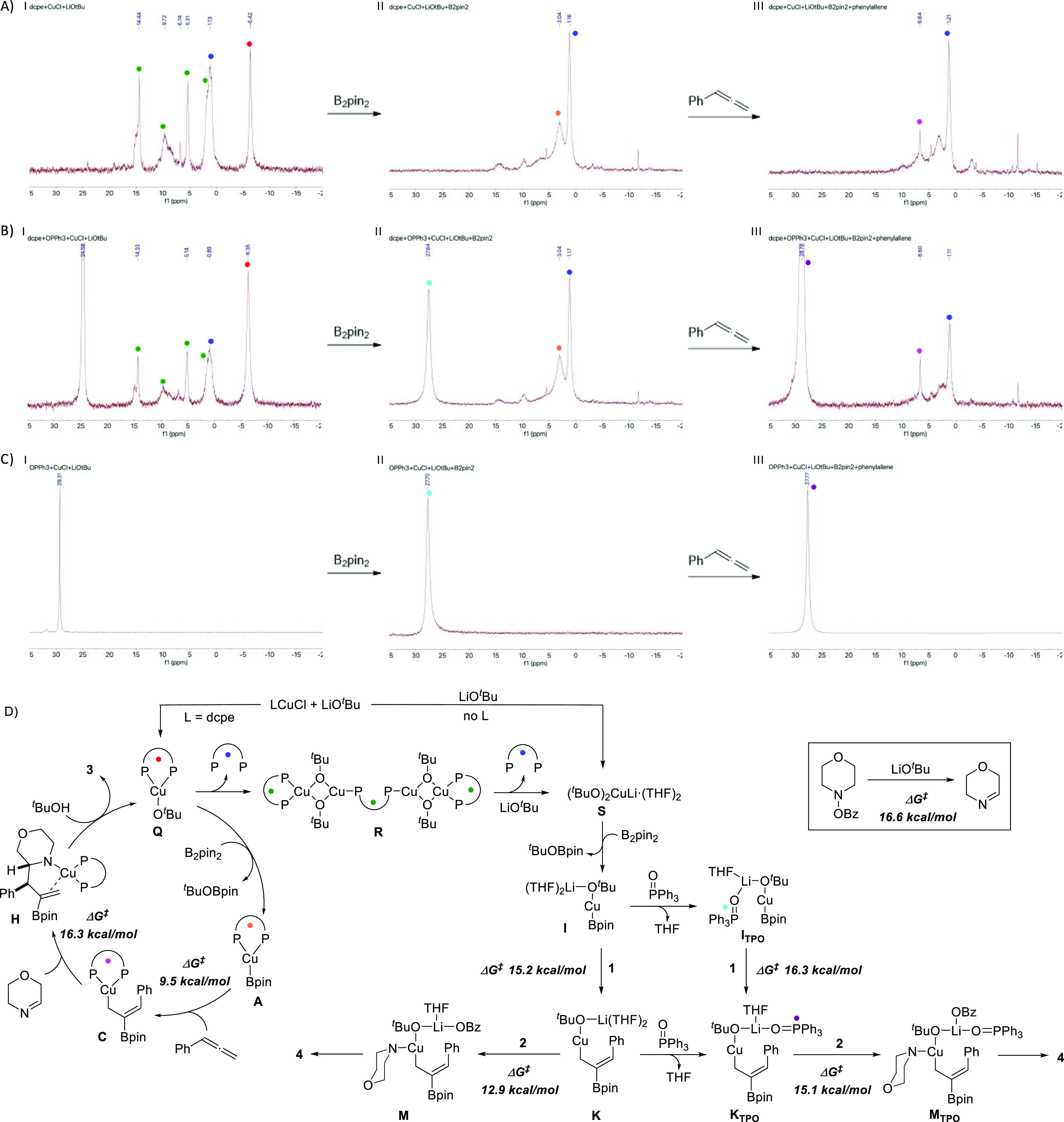
Spectroscopic ^31^P NMR study of (A) CuCl/dcpe/LiO^*t*^Bu system, (B) CuCl/dcpe/P(*O*)Ph_3_/LiO^*t*^Bu system, and (C) CuCl/P(*O*)Ph_3_/LiO^*t*^Bu system. (D) Proposed mechanism.

In line with these DFT calculations, ^31^P NMR spectroscopic studies revealed that the dcpe ligand dissociates from the metal center during the formation of the copper *tert*-butoxide complex. When CuCl (1.0 equiv), dcpe (1.1 equiv), and LiO^*t*^Bu (2.0 equiv) were mixed (Figure 6A, spectrum I), we observed the formation of Cu(dcpe)-O^*t*^Bu (**Q**), derived aggregates **R**,^23^ and a free dcpe ligand that most probably involves formation of (CuO^*t*^Bu)_*n*_,^25^ which in the presence of excess LiO^*t*^Bu would form phosphine-free copper bis(*tert*-butoxide) species **S**. Addition of B_2_pin_2_ led to the disappearance of the LCu-O^*t*^Bu signals, and a new peak assigned to Cu(dcpe)-Bpin (**A**) was formed (Figure 6A, spectrum II). Subsequent addition of phenylallene afforded the allyl-Cu(dcpe) complex **C** with ∼50% conv. (Figure 6A, spectrum III). In both cases, the presence of a free dcpe ligand suggests that there is no ligand reassociation and points at the presence of nonligated Cu-Bpin complex **I** and allyl-Cu complex **K**, respectively.

In Cu(I) complexes, alkoxide ligands mainly act as σ-donors, thus providing the oxygen atom enough Lewis basicity to facilitate aggregation by alkoxo bridging in a process that can lead to ligand dissociation.^23,25^ Accordingly, the large size of the dcpe ligand and its hemilability may favor the formation of oligomeric species and ligand dissociation at the Cu-O^*t*^Bu stage. Taken together, these experiments suggest that dcpe ligand dissociation is problematic and leads to phosphine-free Cu species, which may be responsible for the formation of side-product **4** especially at the beginning of the reaction when the imine concentration is low. In this sense, the lower chemoselectivity observed when sodium methoxide is used as the base (Table 1, entry 17) may be due to the higher propensity of the resulting copper alkoxide complexes to undergo aggregation.^23^

So, what is the role of the catalytic Lewis base additive and why does it increase chemoselectivity? ^31^P NMR of a mixture of CuCl (1.0 equiv), dcpe (1.1 equiv) P(*O*)Ph_3_ (1.0 equiv), and LiO^*t*^Bu (2.0 equiv) also revealed the formation of Cu aggregates **R** and ligand dissociation, although in a minor extent (Figure 6B, spectrum I).^26^ Under these conditions, formation of Cu(dcpe)-O^*t*^Bu **Q** was mainly observed, while broadening of the P(*O*)Ph_3_ may suggest coordination to other Cu species (Figure 6B, spectrum I). Addition of B_2_pin_2_ yielded Cu(dcpe)-Bpin **A**, a free dcpe ligand, and new coordinated P(*O*)Ph_3_ species **I_TPO_** (Figure 6B, spectrum II). Interestingly, this same species **I_TPO_** (27.7 ppm) was formed when CuCl (1.0 equiv), P(*O*)Ph_3_ (1.1 equiv), LiO^*t*^Bu (2.0 equiv), and B_2_pin_2_ (1.2 equiv) were mixed in the absence of dcpe (Figure 6C, spectrum II). This, together with the fact that the same dcpe intermediates are observed in both experiments (Figure 6A,B), may suggest that no heteroleptic Cu-Bpin complex is formed in the presence of P(*O*)Ph_3_. A similar behavior was observed upon allene addition that yielded allyl-Cu(dcpe) complex **C** and bound P(*O*)Ph_3_ species, which appear together with a free dcpe ligand (Figure 6B, spectrum III). These experiments suggest that P(*O*)Ph_3_ has a dual role by reducing aggregation (and thus ligand dissociation) and, in a higher extent, by interacting with the Cu intermediates generated from the phosphine-free CuO^*t*^Bu to produce a likely less active system **K_TPO_** for C–N bond formation. Indeed, DFT calculations systematically showed higher activation energy barriers for all the pathways related to the Cu/LiO^*t*^Bu/POPh_3_ system (Figure 5, pink and brown pathways) when compared to those of the phosphine-free Cu system (Figure 5, green and orange pathways). The fact that only a trace amount of product **4** was obtained when P(*O*)Ph_3_ was used as the sole ligand (Table 1, entry 15) also supports such a deactivating interaction.

**Figure 7:**
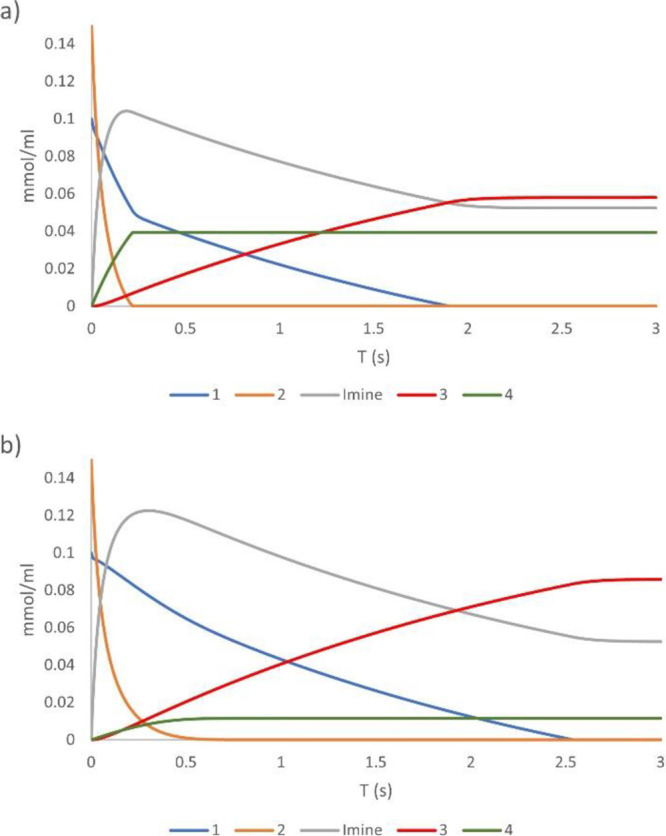
Microkinetic simulations according to the calculated Gibbs energy profiles under (a) P(*O*)Ph_3_-free conditions (Table 1, entry 10) and (b) in the presence of P(*O*)Ph_3_ (Table 1, entry 12).

Further demonstration of the effect of the catalytic Lewis base additive on the reaction outcome was obtained by microkinetic analysis^27^ of the relative final distribution of products at 298 K according to the calculated Gibbs energy profiles (Figures 4 and 5), using the conditions shown in Table 1, entries 10 and 12. In the absence of P(*O*)Ph_3_, and assuming a 1:1 ratio between Cu(dcpe)Bpin (**A**) and Cu(O^*t*^Bu)(Bpin)Li (**I**) complexes,^28^ microkinetic simulation shows that formation of C–N coupling product **4** is competitive at initial times when the imine concentration is still low. As a result, products **3**:**4** are formed in a 1.5:1 ratio (Figure 7a). In the presence of P(*O*)Ph_3_ (Figure 7b), the lower efficiency of complex **I_TPO_** results in almost the exclusive formation of C–C coupling product **3** (**3**:**4**, 7.5:1 ratio).

The above findings show that the homoleptic Cu/dcpe system is responsible for the C–C bond formation (Figure 6D). Ligand dissociation leads to the generation of new phosphine-free copper species with different reactivities, which results in a decrease of chemoselectivity. The presence of P(*O*)Ph_3_ does not interfere in the Cu/dcpe catalytic cycle but coordinates to the phosphine-free Cu species likely by metal ion chelation^29^ leading to less-active intermediates.^30^ Thus, the Lewis base additive inhibits the catalytic activity toward C–N coupling but does not participate in the C–C bond forming event.

## Conclusions

In summary, we have developed a copper-catalyzed borylative α-C–H allylation of cyclic *O*-benzoyl hydroxylamines. The method provides functionalized cyclic secondary amines with very high levels of chemo-, regio-, and stereoselectivity. Interesting structural features of these products are the presence of a synthetically versatile boron-containing allyl group and an azaboraspiro structure established by a dative nitrogen–boron coordination. This novel transformation occurs via the trapping of a catalytic allylcopper intermediate with a cyclic imine, which is in situ generated from the *O*-benzoyl hydroxylamine. Key to selectively achieve this transformation is the use of a catalytic Lewis base additive in combination with a Cu/dcpe catalyst. A combined spectroscopic and computational study reveals that the involved bis-phosphine Cu-alkoxide species are prone to ligand loss, resulting in phosphine-free Cu intermediates that lead to a lower chemoselectivity. The Lewis base additive deactivates these species by metal ion chelation, thus precluding C–N bond formation and allowing the C–C bond forming product to be obtained with excellent selectivity. We expect that these new findings will serve as the basis for the design of new transformations, especially those that involve the use of hemilabile ligands.

## Supporting Information Available

List of starting materials and ligands, optimization studies, experimental procedures and compound characterization data, imine formation experiments, details of ^31^P NMR study of copper intermediates, microkinetic simulations and computational details, Cartesian coordinates, imaginary frequencies, and absolute energies in hartrees for all optimized geometries (PDF)
