# Supplementary material for: Catalytic Lewis Base Additive Enables Selective Copper-Catalyzed Borylative α-C–H Allylation of Alicyclic Amines
Source: J Am Chem Soc. 2022 Aug 24;144(35):16206–16. doi: 10.1021/jacs.2c07969 (PMC9460777; doi:10.1021/jacs.2c07969)
Supplement: Supplementary file 1 — ja2c07969_si_001.pdf [file ja2c07969_si_001.pdf]

Catalytic Lewis Base Additive Enables Selective Copper-Catalyzed Borylative α-C-H Allylation of Alicyclic Amines

Borja Pérez Saavedra, Álvaro Velasco Rubio, Eva Rivera-Chao, Jesús A. Varela, Carlos Saá and Martín Fañanás-Mastral*

Centro Singular de Investigación en Química Biolóxica y Materiais Moleculares (CiQUS), Departamento de. Química Orgánica, Universidade de Santiago de Compostela 15782 Santiago de Compostela, Spain

Correspondence to: martin.fananas@usc.es

Supporting Information

Table of contents

1. General methods *4*

2. List of starting materials and ligands *5*

3. Optimization studies *10*

4. General procedures *14*

5. Product characterization *15*

6. Product derivatization *22*

6.1. Heck coupling 22

6.2. Protonation of 16 23

6.3. Derivatization of alkenyl boronate 24

6.4. Conversion of 16 into tosyl amide 54 for er determination (see Table S6) 27

7. Reaction with asymmetric O-benzoyl hydroxylamine 55 .... *28*

8. NMR spectra *29*

9. Stereochemical determination of Heck products by NOESY experiment *76*

10. X-Ray diffraction analysis data *77*

10.1. X-Ray diffraction analysis data of product 7 77

10.2. X-Ray diffraction analysis data of product 16 78

10.3. X-Ray diffraction analysis data of product 27 79

10.4. X-Ray diffraction analysis data of product 33 80

11. Proposed mechanism for the formation of product 32 .... *81*

12. Imine formation experiments *82*

13. ^{31}P NMR studies of phosphine-Cu complexes *88*

14. DFT calculations *97*

14.1. Computational details 97

14.2. Free energy profiles for the pathways associated to the Cu/dcpe system (Figure S18) 98

14.3. Evaluation of explicit solvent molecules for calculations involving the phosphine-free Cu system (Figure S19) 100

14.4. Free energy profiles for the pathways associated to the phosphine-free Cu system (Figure S20) 101

14.5. Free energy profile for the Cu/LiO^{t}Bu/P(O)Ph_3 system (Figure S21) 102

14.6. Free energy profiles for the addition of allyl copper complex C into alicyclic imine derived from morpholino benzoate 2 (Figure S22) 103

14.7. Free energy profile for the formation of the imine derived from morpholino benzoate 2 (Figure S23) 104

14.8. Optimized transition states for the formation of compound 16: Explanation of the diastereocontrol over the three stereocenters 105

14.9. Cartesian coordinates in Å for all calculated structures 106
14.9.1. Cartesian coordinates for common species 106
14.9.2. Cartesian coordinates for the Cu/dcpe system (Figures S18, S22 and S24) 118
14.9.3. Cartesian coordinates for the phosphine-free Cu system (Figures S19 and S20).......................... 189
14.9.4. Cartesian coordinates for the Cu/LiO^{t}Bu/$P(O)Ph_3$ system (Figure S21).. 244
14.9.5. Cartesian coordinates for the imine formation (Figure S23).. 275

15. Microkinetic simulations 280

15.1 Thermal rate constants k (298 K) for steps involving the most significant transitions states for microkinetic simulations 280

15.2 Microkinetic simulation for the reaction in the absence of dcpe ligand (conditions: Table 1, entry 14) 281

15.3 Microkinetic simulation for the reaction in the absence of dcpe ligand and in the presence of 6 mol% of $P(O)Ph_3$ (conditions: Table 1, entry 15)... 281

15.4 Microkinetic simulation for the reaction using dcpe ligand (conditions: Table 1, entry 10) 282

15.5 Microkinetic simulation for the reaction using dcpe ligand and $P(O)Ph_3$ as catalytic additive (conditions: Table 1, entry 12) 283

1. General methods

All reactions were performed under argon atmosphere using oven dried glassware and using standard Schlenk techniques. Solvents were dried using an MBraun SPS 800 system. All chemicals were purchased from Acros Organics Ltd., Aldrich Chemical Co. Ltd., Alfa Aesar, Apollo, Strem Chemicals Inc., Fluorochem Ltd. Or TCI Europe N. V. chemical companies and used without further purification, unless otherwise noted.

Analytical thin layer chromatography was performed on silica-coated aluminum plates (60 F_{254} Merck). Compounds were visualized using 254 nm UV light or by oxidation treatment (solution of 1.5 g of $KMnO_4$, 10 g of $KHCO_3$ and 1.25 mL of an aqueous solution of NaOH (10 (w/w)%) in 200 mL of H_2O) and heat. Preparative thin layer chromatography was carried out on 20 x 20 cm silica-coated glass plates (60 F_{254} Merck, 1mm). Flash column chromatography was performed on silica gel (60, 230-400 mesh, Merck).

GC-MS analyses were performed in an Agilent instrument GC-6890N equipped with Chemical Ionization (CI) MS-5973 detector. High Resolution Mass spectrometry was carried out in a Bruker microTOF spectrometer.

HPLC analysis was performed in a Waters Acquity Arc System, consisting of a quaternary pump, column oven and auto-sampler coupled with a 2998 PDA detector. Empower QS software was used to monitor signals from a diode-array detector and to process the collected analytical data.

^{1}H-, ^{13}C-, ^{19}F- and ^{11}B-NMR experiments were carried out using a Varian Inova 500 MHz or Varian Mercury 300 MHz spectrometer. For ^{1}H- and ^{13}C-NMR, chemical shifts are reported relative to residual solvent peaks. Coupling constants (*J*) are given in Hertz (Hz). Multiplicities are reported as follows: s = singlet, d = doublet, t= triplet, q = quartet, m = multiplet or as a combination of them. Carbon atoms directly attached to boron could not been detected by ^{13}C-RMN due to quadrupolar relaxation. External references were used for ^{19}F-NMR (trifluorotoluene: -62.5 ppm in C_6D_6) and for ^{11}B-NMR ($BF_3{\cdot}OEt_2$: 0 ppm in D_2O).

In order to preclude side hydroboration reactions, commercial allenes and B_2pin_2 were dried over $MgSO_4$ prior to being used.

2. List of starting materials and ligands

- **Allenes:**

Allenes **38** and **41** were purchased from Aldrich Chemical Co. Ltd. Allenes **1**,[1] **34-36**,[1] **37**,[2] **39**[3] and **40**[1] were synthesized as reported in literature.

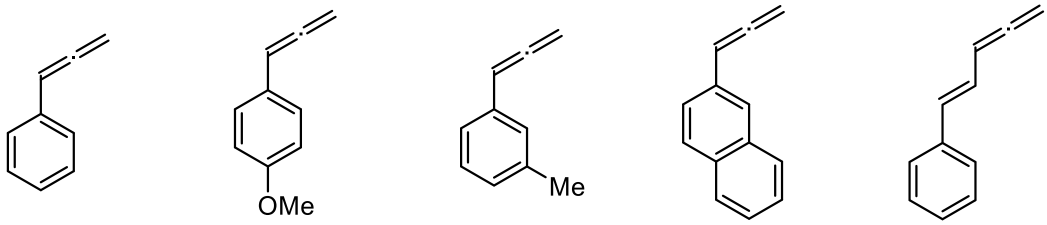

1 **34** **35** **36** **37**

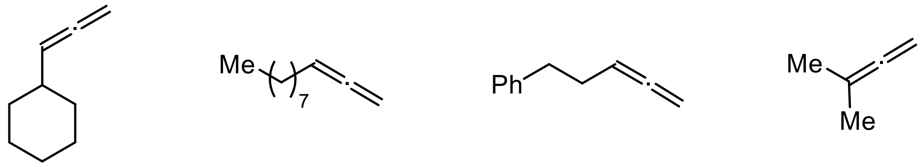

38 **39** **40** **41**

- ***O*-Benzoyl hydroxylamines**

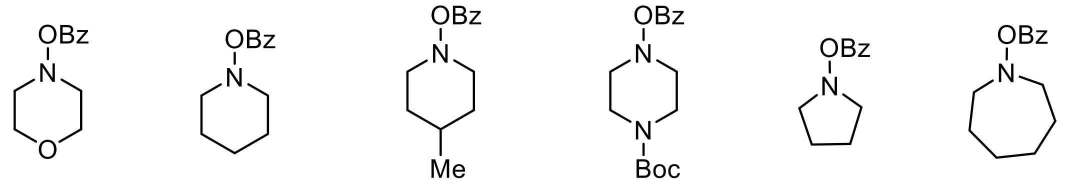

2 **42** **43** **44** **45** **46**

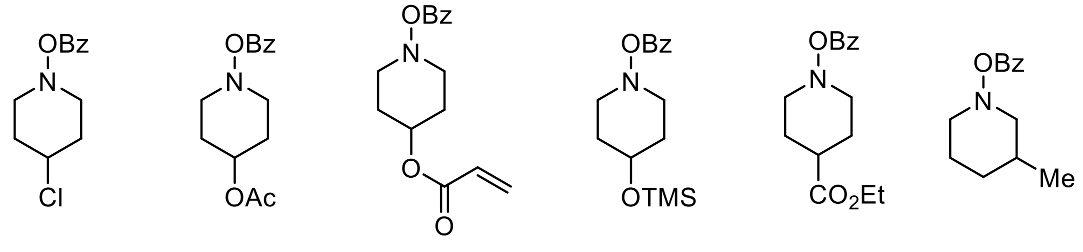

47 **48** **49** **50** **51** **55**

---

[1] Liu, J.; Han, Z.; Wang, X.; Wang, Z.; Ding, K. *J. Am. Chem. Soc.* **2015**, *137*, 15346–15349.
[2] Velegraki, G.; Stratakis, M. J. *J. Org. Chem.* **2013**, *78*, 8880–8884.
[3] Kuang, J.; Ma, S. *J. Org. Chem.* **2009**, *74*, 1763–1765.

Synthesis of *O*-benzoyl hydroxylamines 2, 42-47, 51, 55

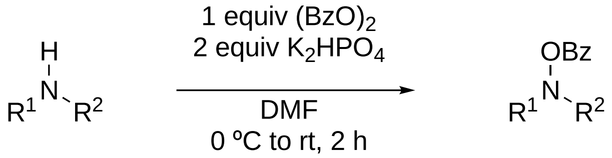

O-Benzoyl hydroxylamines were synthesized according to a modified literature procedure.[4]

A flame-dried round bottom flask equipped with magnetic stirring bar was charged with benzoyl peroxide (70%, 1 equiv) and K_2HPO_4 (2 equiv). The system was put under vacuum and refilled with argon for three times. Then, DMF (0.2 M) was added, and the mixture was cooled to 0 ºC. The corresponding amine (1.5 equiv) was added dropwise, and the suspension was stirred at rt for 2 h. The reaction was quenched with deionized water (20 mL) and the contents were stirred vigorously for several minutes until all solids dissolved. The reaction mixture was extracted with CH_2Cl_2 (3x30 mL). The organic phase was collected and washed with saturated aq. $NaHCO_3$ (2x20 mL), saturated aq. NH_4Cl (25 mL) and brine (25 mL), dried over anhydrous Na_2SO_4, filtered, and concentrated under vacuum. The residue was purified by flash column chromatography on silica gel using a mixture of AcOEt/Hexanes (1:9) as eluent.

Morpholino benzoate (2)[4]

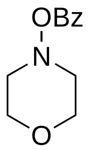

Obtained in 75% yield as a white solid.
^{1}H NMR (300 MHz, $CDCl_3$) δ 8.00 (dq, *J* = 7.1, 1.3 Hz, 2H), 7.64 – 7.51 (m, 1H), 7.51 – 7.38 (m, 2H), 4.21 – 3.70 (m, 4H), 3.51-3.36 (m, 2H), 3.06 (d, *J* = 10.8 Hz, 2H). **^{13}C NMR** (75 MHz, $CDCl_3$) δ 164.7 (C), 133.3 (CH), 129.6 (C), 129.3 (CH), 128.6 (CH), 65.9 (CH_2), 57.1 (CH_2).

Piperidin-1-yl benzoate (42)[4]

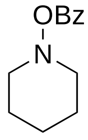

Obtained in 70% yield as a white solid.
^{1}H NMR (300 MHz, $CDCl_3$) δ 8.05 – 7.95 (m, 2H), 7.60 – 7.48 (m, 1H), 7.47 – 7.36 (m, 2H), 3.50 (s, 2H), 2.78 (s, 2H), 1.81 (q, *J* = 5.7 Hz, 4H), 1.66 (s, 1H), 1.28 (s, 1H). **^{13}C NMR** (75 MHz, $CDCl_3$) δ 164.8 (C), 133.0 (CH), 129.8 (C), 129.5 (CH), 128.5 (CH), 57.6 (CH_2), 25.1 (CH_2), 23.4 (CH_2).

4-Methylpiperidin-1-yl benzoate (43)[4]

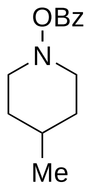

Obtained in 85% yield as a white solid.
^{1}H NMR (300 MHz, $CDCl_3$) δ 8.06 – 7.93 (m, 2H), 7.60 – 7.50 (m, 1H), 7.50 – 7.37 (m, 2H), 3.53 (d, *J* = 9.3 Hz, 2H), 2.74 (t, *J* = 10.8 Hz, 2H), 1.79 – 1.46 (m, 5H), 0.96 (d, *J* = 5.9 Hz, 3H). **^{13}C NMR** (75 MHz, $CDCl_3$) δ 164.9 (C), 133.0 (CH), 129.8 (C), 129.5 (CH), 128.5 (CH), 57.3 (CH_2), 33.7 (CH_2), 30.2 (CH), 21.3 (CH_3).

[4] Gou, Q.; Liu, G.; Liu, Z.-N.; Qin, J. *Chem. Eur. J.* **2015**, *21*, 15491-15495.

tert-Butyl 4-(benzoyloxy)piperazine-1-carboxylate (44)[4]

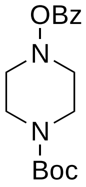

Obtained in 80% yield as a white solid.

^{1}H NMR (300 MHz, $CDCl_3$) δ 8.05 – 7.95 (m, 2H), 7.63 – 7.50 (m, 1H), 7.50 – 7.38 (m, 2H), 4.02 (s, 2H), 3.67 – 3.18 (m, 4H), 2.90 (s, 2H), 1.48 (s, 9H). **^{13}C NMR** (75 MHz, $CDCl_3$) δ 164.7 (C), 154.6 (C), 133.4 (CH), 129.6 (C), 129.3 (CH), 128.6 (CH), 80.4 (C), 56.0 (CH_2), 42.2 (CH_2), 28.5 (3xCH_3).

Pyrrolidin-1-yl benzoate (45)[5]

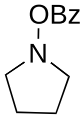

Obtained in 50% yield as a white solid.

^{1}H NMR (300 MHz, CDC_3) δ 8.00 – 7.91 (m, 2H), 7.59 – 7.47 (m, 1H), 7.41 (t, J = 7.6 Hz, 2H), 3.29 (td, J = 5.9, 3.3 Hz, 4H), 2.10 – 1.79 (m, 4H). **^{13}C NMR** (75 MHz, $CDCl_3$) δ 165.2 (C), 133.0 (CH), 129.7 (C), 129.5 (CH), 128.4 (CH), 57.8 (CH_2), 22.3 (CH_2).

Azepan-1-yl benzoate (46)[5]

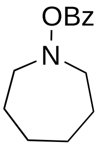

Obtained in 75% yield as a white solid.

^{1}H NMR ^{1}H NMR (300 MHz, $CDCl_3$) δ 8.00 (dq, J = 7.1, 1.3 Hz, 2H), 7.59 – 7.51 (m, 1H), 7.48 – 7.38 (m, 2H), 3.38 – 3.28 (m, 4H), 1.88 – 1.78 (m, 4H), 1.72 – 1.65 (m, 4H). **^{13}C NMR** (75 MHz, $CDCl_3$) δ 164.9 (C), 133.0 (CH), 129.9 (C), 129.5 (CH), 128.5 (CH), 59.6 (CH_2), 26.6 (CH_2), 24.3 (CH_2).

4-Chloropiperidin-1-yl benzoate (47)[6]

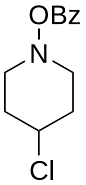

Obtained in 70% yield as a yellow solid.

^{1}H NMR (300 MHz, $CDCl_3$) δ 7.99 (d, J = 7.6 Hz, 2H), 7.62 – 7.50 (m, 1H), 7.55 – 7.37 (m, 2H), 4.52 – 3.94 (m, 1H), 3.74 – 2.75 (m, 4H), 2.44 – 2.24 (m, 2H), 2.23 – 2.01 (m, 2H). **^{13}C NMR** (75 MHz, $CDCl_3$) δ 164.7 (C), 133.2 (CH), 129.5 (C), 129.4 (CH), 128.5 (CH), 55.2 (CH), 51.9 (CH_2), 33.2 (CH_2).

Ethyl 1-(benzoyloxy)piperidine-4-carboxylate (51)[7]

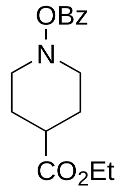

Obtained in 75% yield as a colorless oil.

^{1}H NMR (300 MHz, $CDCl_3$) δ 8.04 – 7.92 (m, 2H), 7.60 – 7.49 (m, 1H), 7.41 (td, J = 7.1, 1.1 Hz, 2H), 4.14 (q, J = 7.0 Hz, 2H), 3.59 (s, 1H), 3.26 (s, 1H), 2.88-2.50 (m, 2H), 2.35 (s, 1H), 2.15 – 1.93 (m, 4H), 1.25 (dd, J = 8.2, 6.1 Hz, 3H). **^{13}C NMR** (75 MHz, $CDCl_3$) δ 174.1 (C), 164.7 (C), 133.1 (CH), 129.5 (CH), 128.5 (CH), 60.7 (CH_2), 56.2 (CH_2), 40.5 (CH), 27.7 (CH_2), 14.3 (CH_3).

3-Methylpiperidin-1-yl benzoate (55)

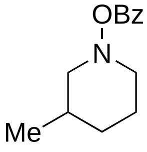

Obtained in 60% yield as a pale-yellow solid.

^{1}H NMR (500 MHz, $CDCl_3$) δ 8.04 – 7.97 (m, 2H), 7.57 – 7.51 (m, 1H), 7.42 (t, J = 7.8 Hz, 2H), 3.53 (dd, J = 18.9, 9.2 Hz, 2H), 2.61 (s, 1H), 2.34 (t, J = 10.3 Hz, 1H), 2.08 – 1.66 (m, 5H), 0.94 (d, J = 6.7 Hz, 3H). **^{13}C NMR** (126 MHz, $CDCl_3$)

[5] Dong, Z.; Dong, G. *J. Am. Chem. Soc.* **2013**, *135*, 18350.
[6] Dhanju, S.; Blazejewski, B. W.; Crich, D. *J. Org. Chem.* **2017**, *82*, 5345.
[7] Zhou, P.-X.; Ye, Y.-Y.; Ma, J.-W.; Zheng, L.; Tang, Q.; Qiu, Y.-F.; Song, B.; Qiu, Z.-H.; Xu, P.-F.; Liang, Y.-M. *J. Org. Chem.* **2014**, *79*, 6627.

δ 164.9 (C), 133.0 (CH), 129.8 (CH), 129.5 (CH), 128.5 (CH), 65.0 (CH_2), 57.2 (CH_2), 32.2 (CH), 31.6 (CH_2), 24.8 (CH_2), 19.6 (CH_3).

Synthesis of *O*-Benzoyl hydroxylamines 48-50

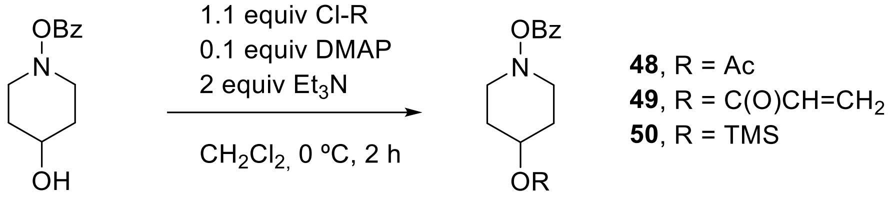

S1

A flame-dried round bottom flask equipped with magnetic stirring bar was charged with compound **S1**[8] (1 equiv) and DMAP (0.1 equiv). It was put in vacuum and refilled with argon for three times. CH_2Cl_2 (0.2 M) was added, and the reaction was cooled to 0 ºC. Then, Et_3N (2 equiv) was added, followed by the corresponding chloride (1.1 equiv) (Note: the addition must be done dropwise, evolution of HCl was observed). The reaction was stirred for 2 h at 0 ºC. Afterwards, the reaction was quenched with brine and extracted with CH_2Cl_2 (3x15 mL). The combined organic layers were dried over Na_2SO_4 and purified by silica column chromatography using 20% of AcOEt in Hexanes as eluent to give the desired product.

4-Acetoxypiperidin-1-yl benzoate (48)

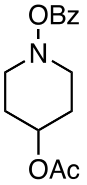

Obtained in 60% yield as a colorless oil from acyl chloride.
^{1}H NMR (300 MHz, $CDCl_3$) δ 8.10 – 7.94 (m, 2H), 7.64 – 7.50 (m, 1H), 7.42 (dd, *J* = 8.3, 6.9 Hz, 2H), 5.12 – 4.83 (m, 1H), 3.51 (bs, 1H), 3.33 (bs, 1H), 3.16 (bs, 1H), 2.95 (bs, 1H), 2.27 (s, 3H), 2.16 – 1.92 (m, 4H). **^{13}C NMR** (75 MHz, $CDCl_3$) δ 176.0 (C), 164.7 (C), 133.2 (CH), 129.4 (CH), 129.22(CH), 128.4 (CH), 67.9(CH), 53.7 (CH_2), 52.2 (CH_2), 50.2 (CH_2), 30.3 (CH_2), 28.8 (CH_3). **HRMS** (APCI, m/z): calculated for $C_{14}H_{18}NO_4$ [M^+ + H]: 264.1236; found: 264.1230.

4-(Acryloyloxy)piperidin-1-yl benzoate (49)

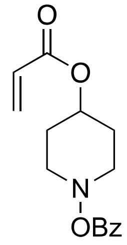

Obtained in 90 % yield as an off-white solid using acryloyl chloride.
^{1}H NMR (300 MHz, $CDCl_3$) δ 7.99 (d, *J* = 7.6 Hz, 2H), 7.62 – 7.48 (m, 1H), 7.48 – 7.34 (m, 2H), 6.41 (d, *J* = 17.3 Hz, 1H), 6.13 (t, *J* = 13.6 Hz, 1H), 5.83 (d, *J* = 10.3 Hz, 1H), 5.12 – 4.82 (m, 1H), 3.65 – 2.85 (m, 4H), 2.22 – 1.89 (m, 4H). **^{13}C NMR, DEPT** (75 MHz, $CDCl_3$) δ 165.5 (C), 164.7 (C), 133.2 (CH), 131.0 (CH_2), 129.5 (CH), 129.4 (C), 128.7 (CH), 128.5 (CH), 68.8 (CH), 53.9 (CH_2), 52.4 (CH_2), 29.0 (2xCH_2). **HRMS** (APCI, m/z): calculated for $C_{15}H_{18}NO_4$ [M^+ + H]: 276.1236; found: 276.1240.

---

[8] Rucker, R. P.; Whittaker, A. M.; Dang, H.; Lalic, G. *Angew. Chem. Int. Ed.* **2012**, *51*, 3953.

4-((Trimethylsilyl)oxy)piperidin-1-yl benzoate (50)

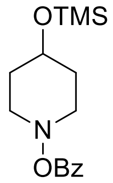

Obtained in 77% yield as a colorless oil using trimethylsilyl chloride. **^{1}H-NMR** (300 MHz, $CDCl_3$) δ 8.06 – 7.94 (m, 2H), 7.55 (t, *J* = 7.4 Hz, 1H), 7.43 (t, *J* = 7.5 Hz, 2H), 3.97 – 3.68 (m, 1H), 3.54 (s, 1H), 3.38 – 3.19 (m, 2H), 2.88 (s, 1H), 2.03 – 1.74 (m, 4H), 0.13 (s, 9H). **^{13}C-NMR, DEPT** (75 MHz, $CDCl_3$) δ 164.9 (C), 133.1 (CH), 129.6 (CH), 128.5 (CH), 64.4 (CH), 54.4 (CH_2), 52.3 (CH_2), 33.3 (CH_2), 32.5 (CH_2), 0.3 (CH_3). **HRMS** (APCI, m/z): calculated for $C_{12}H_{16}NO_3$ [M^+ - TMS]: 222.1125; found: 222.1128.

- **Ligands**

Ligands were purchased from commercial sources and used without further purification.

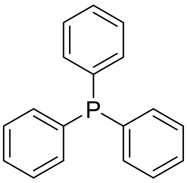

PPh_3

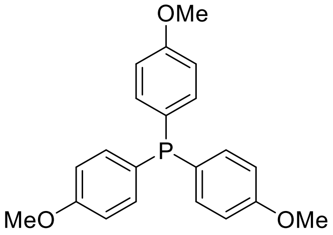

P(4-OMe-C_6H_4)$_3$

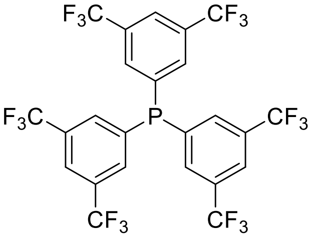

P(3,5-(CF_3)-C_6H_3)$_3$

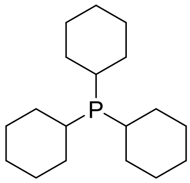

PCy_3

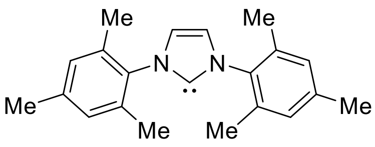

IMes

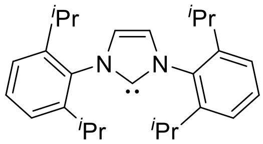

IPr

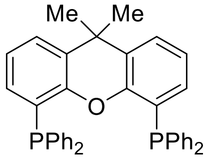

Xantphos

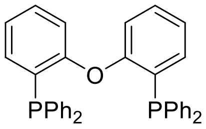

DPEphos

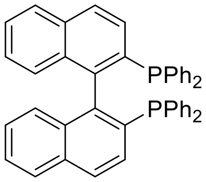

rac-BINAP

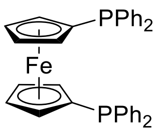

dppf

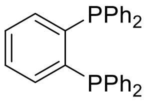

dppbz

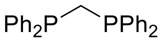

dppm

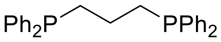

dppp

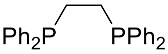

dppe

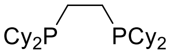

dcpe

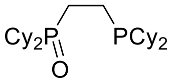

dcpeO

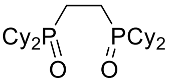

dcpeO_2

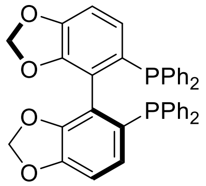

(*S*)-Segphos

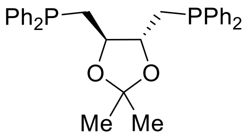

(*R*,*R*)-DIOP

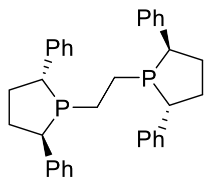

(*R*,*R*)-Ph-BPE

3. Optimization studies

Table S1. Full screening of ligands (without additive)

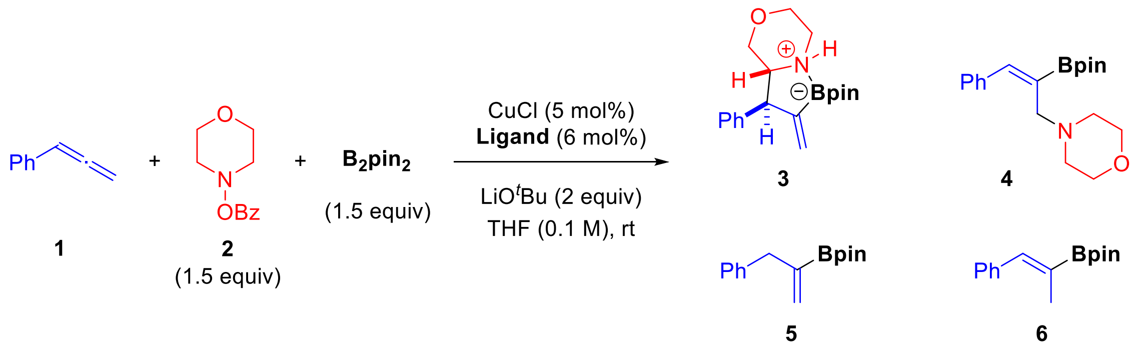

Entry[a]	Ligand	**3** (%)[b]	**4** (%)[b]	**5** (%)[b]	**6** (%)[b]
1	PPh_3	<5	30	30	25
2	$P(4\text{-}OMe\text{-}C_6H_4)_3$	<5	31	31	23
3	$P(3,5\text{-}(CF_3)\text{-}C_6H_3)_3$	<5	30	35	30
4	PCy_3	15	42	23	14
5	IMes	24	36	19	<5
6	IPr	22	<5	5	<5
7	Xantphos	30	<5	30	<5
8	DPEphos	50	24	25	<5
9	*rac*-BINAP	31	35	31	<5
10	dppf	35	14	12	<5
11	dppbz	17	<5	13	<5
12	dppm	<5	<5	<5	<5
13	dppe	11	<5	<5	<5
14	dppp	40	18	20	<5
15	dcpe	25	16	12	<5
16	dcpe*[c]	60	<5	<5	15

[a] Reactions run on a 0.3 mmol scale. Diastereomeric ratio of **3** was >95:5 in all cases. [b] Determined by ^{1}H NMR analysis using 1,3,5-trimethoxybenzene as internal standard. [c] dcpe* = dcpe : dcpe(O) : $dcpe(O)_2$ in a 3:1:3 ratio.

Table S2. Evaluation of phosphine/phosphine oxide cooperativity effect

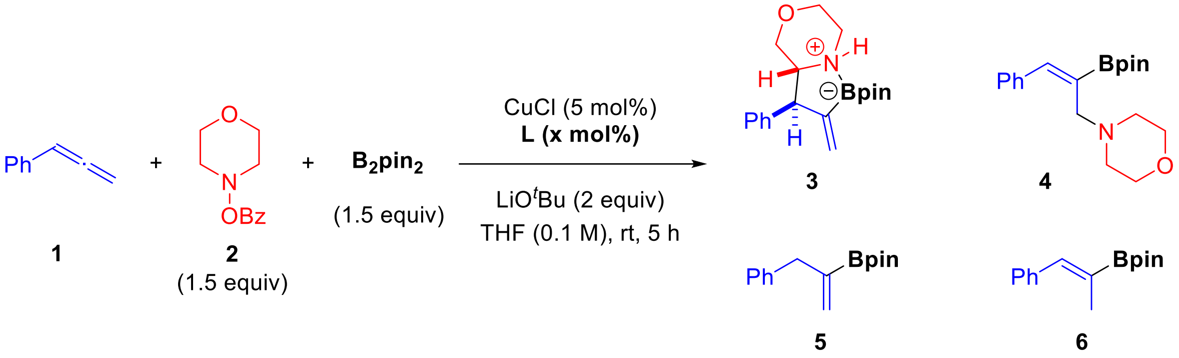

Entry[a]	L (mol%)	**3** (%)[b]	**4** (%)[b]	**5** (%)[b]	**6** (%)[b]
1	dcpe*[c] (6)	60	<5	<5	15
2	dcpe (6)	25	16	12	<5
3	dcpe (9)	25	<5	10	<5
4	dcpe (12)	35	<5	<5	<5
5	-	<5	30	32	<5
6	dcpe (2.5)	<5	25	30	<5
7	dcpeO_2 (6)	<5	14	10	<5
8	dppe (6)	11	<5	<5	<5
9	dppeO (6)	<5	43	35	<5
10	dcpe (6) + P(O)Ph_3 (6)	72	<5	<5	8
11	P(O)Ph_3 (6)	<5	8	6	<5

[a] Reactions run on a 0.3 mmol scale. Diastereomeric ratio of **3** was >95:5 in all cases. [b] Determined by ^{1}H NMR analysis using 1,3,5-trimethoxybenzene as internal standard. [c] dcpe* = dcpe : dcpe(O) : dcpe$(O)_2$ in a 3:1:3 ratio.

Table S3. Screening of different Lewis bases

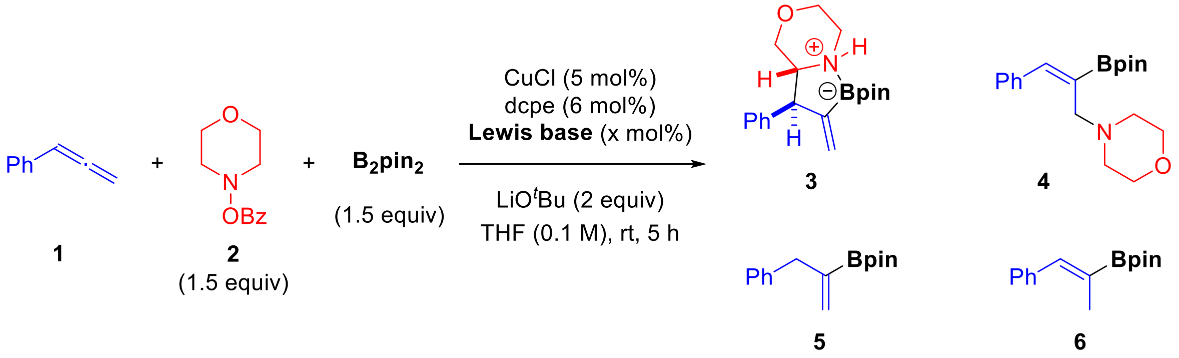

Entry[a]	Lewis base (mol%)	**3** (%)[b]	**4** (%)[b]	**5** (%)[b]	**6** (%)[b]
1	P(O)Ph_3 (6)	72	<5	<5	8
2	P(O)Ph_3 (12)	56	<5	<5	11
3	P(O)Ph_3 (50)	55	<5	<5	27
4	P(O)Bu_3 (6)	30	<5	<5	13
5	HMPA (6)	48	<5	<5	<5
6	PPh_3 (6)	62	<5	<5	16
7	PCy_3 (6)	25	<5	<5	13

[a] Reactions run on a 0.3 mmol scale. Diastereomeric ratio of **3** was >95:5 in all cases. [b] Determined by ^{1}H NMR analysis using 1,3,5-trimethoxybenzene as internal standard.

Table S4. Evaluation of the Lewis base effect with different copper catalysts

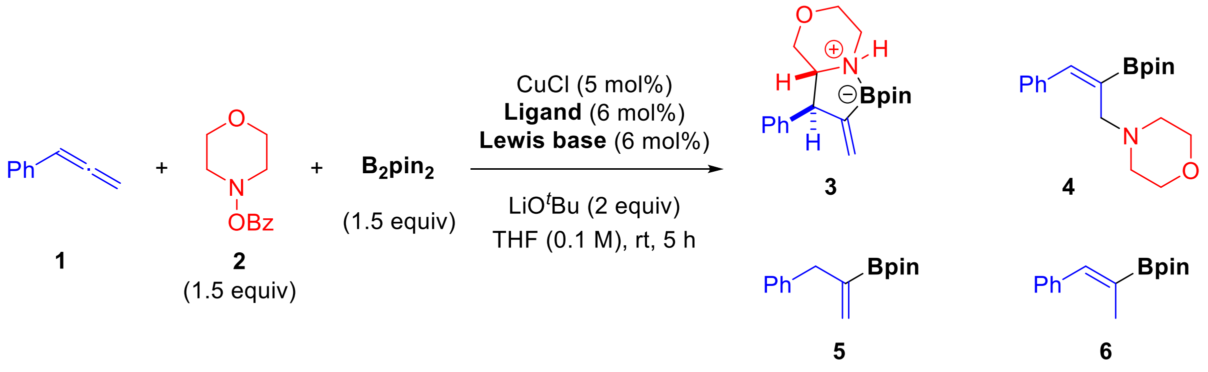

Entry[a]	Ligand	Lewis base	**3** (%)[b]	**4** (%)[b]	**5** (%)[b]	**6** (%)[b]
1	dcpe	-	25	16	12	<5
2	dcpe	$P(O)Ph_3$	72	<5	<5	8
3	dcpe	PPh_3	62	<5	<5	16
4	dppe	-	11	<5	<5	<5
5	dppe	$P(O)Ph_3$	42	<5	20	<5
6	Xantphos	-	30	<5	30	<5
7	Xantphos	$P(O)Ph_3$	30	<5	32	<5
8	DPEphos	-	50	24	25	<5
9	DPEphos	$P(O)Ph_3$	46	27	27	<5
10	DPEphos	PPh_3	45	20	30	<5
11	*rac*-BINAP	-	31	35	31	<5
12	*rac*-BINAP	$P(O)Ph_3$	30	39	28	<5
13	*rac*-BINAP	PPh_3	25	27	40	<5
14	dppf	-	35	14	12	<5
15	dppf	$P(O)Ph_3$	44	25	30	<5
16	dppp	-	40	18	20	<5
17	dppp	$P(O)Ph_3$	36	15	22	<5

[a] Reactions run on a 0.3 mmol scale. Diastereomeric ratio of **3** was >95:5 in all cases. [b] Determined by ^{1}H NMR analysis using 1,3,5-trimethoxybenzene as internal standard.

Table S5. Screening of bases and solvents under different reaction conditions

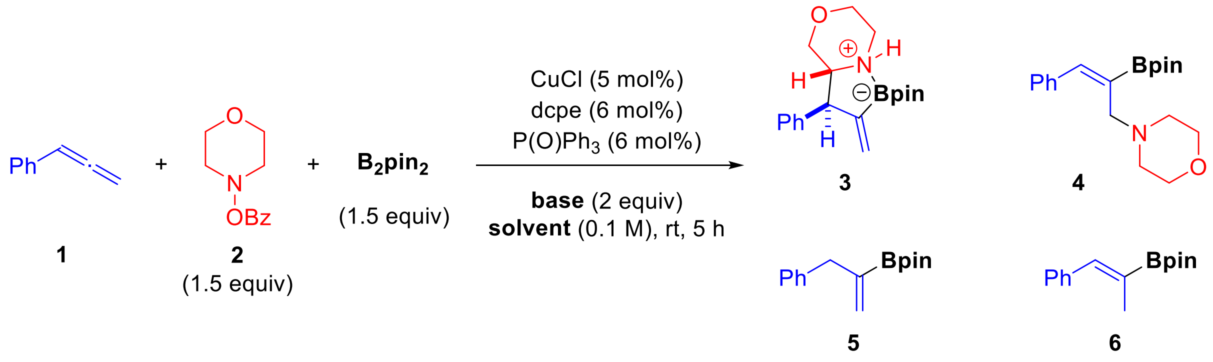

Entry[a]	Base	Solvent	**3** (%)[b]	**4** (%)[b]	**5** (%)[b]	**6** (%)[b]
1	LiO^{t}Bu	THF	72	<5	<5	8
2[c,d]	LiO^{t}Bu	THF	68	<5	<5	20
3[e]	LiO^{t}Bu	THF	45	<5	<5	10
4[f]	LiO^{t}Bu	THF	40	<5	<5	12
5[g]	LiO^{t}Bu	THF	6	10	40	12
6	NaO^{t}Bu	THF	60	<5	<5	10
7	KO^{t}Bu	THF	-	-	-	-
8	NaOMe	THF	23	28	30	15
9	LiO^{t}Bu	1,4-dioxane	20	<5	<5	21
10	LiO^{t}Bu	toluene	25	<5	<5	20
11	LiO^{t}Bu	CH_2Cl_2	17	<5	9	28
12	LiO^{t}Bu	DMF	-	-	-	-
13	LiO^{t}Bu	DMA	-	-	-	-

[a] Reactions run on a 0.3 mmol scale. Diastereomeric ratio of **3** was >95:5 in all cases. [b] Determined by ^{1}H NMR analysis using 1,3,5-trimethoxybenzene as internal standard. [c] Reaction run at 50 ºC. [d] Reaction completed in 1 h. [e] [**1**] = 0.2 M. [f] [**1**] = 0.05 M. [g] A solution of **2** was added over 3 h using a syringe pump.

Table S6. Screening of chiral ligands

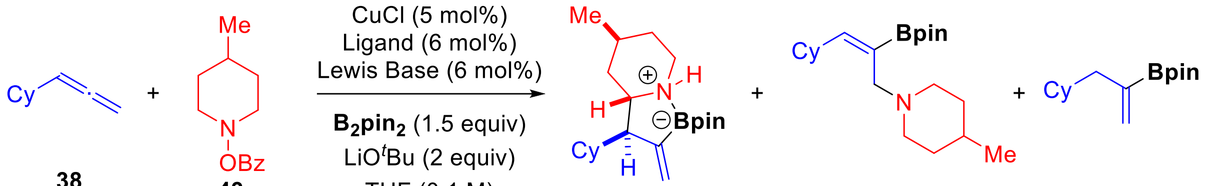

38 **43** (1.5 equiv) **16** **52** **53**

Entry[a]	Ligand	Lewis base	**16** (%)[b] / (e.r.)[c]	**52** (%)[b]	**53** (%)[b]
1	(*S*)-BINAP	-	<5	39	33
2	(*S*)-BINAP	P(O)Ph_3	<5	31	29
3	(*R*,*R*)-DIOP	-	<5	20	25
4	(*R*,*R*)-DIOP	P(O)Ph_3	<5	28	30
5	(*S*)-Segphos	-	<5	45	40
6	(*S*)-Segphos	P(O)Ph_3	<5	45	40
7	(*R*,*R*)-Ph-BPE	-	18 / (30.5:69.5)	45	30
8	(*R*,*R*)-Ph-BPE	P(O)Ph_3	25 / (31.5:68.5)	60	10

[a] Reactions run on a 0.3 mmol scale. [b] Determined by ^{1}H NMR analysis using 1,3,5-trimethoxybenzene as internal standard. [c] Enantiomeric ratio was determined by conversion of **16** into tosyl amide **54** and using chiral HPLC analysis (see section 6.4).

4. General procedures

Copper-catalyzed borylative α-C-H allylation of *O*-benzoyl hydroxylamines (general procedure A)

A screw cap vial was charged with CuCl (0.015 mmol, 0.05 equiv), dcpe (0.018 mmol, 0.06 equiv) and LiO^{t}Bu (0.6 mmol, 2 equiv) in a glovebox. The vial was removed from the glovebox, $P(O)Ph_3$ (0.018 mmol, 0.06 equiv) was added, it was purged with argon three times and 2 mL of THF was added. A solution of allene **7** (0.3 mmol, 1 equiv), *O*-benzoyl hydroxylamine **2** (0.45 mmol, 1.5 equiv) and B_2pin_2 (0.45 mmol, 1.5 equiv) in 1 mL of THF was prepared in a dry separate vial. This solution was added to the first vial and the mixture was stirred at room temperature until complete conversion (TLC). When the reaction has finished, 1,3,5-trimethoxybenzene (3 mmol) as an internal standard to determine the yield by ^{1}H-NMR analysis. The mixture was diluted with EtOAc and it was washed with a saturated aqueous solution of NH_4Cl. The aqueous phase was extracted twice with EtOAc and the combined organic extracts were dried with anhydrous Na_2SO_4, filtered and solvents were evaporated under vacuum. The product was purified by flash column chromatography on silica gel, deactivated with Et_3N when indicated, using mixtures of MeOH/Et_2O, EtOAc/*n*-hexane or Et_2O/*n*-hexane as eluents.

In cases in which the product cannot be isolated by column chromatography, an oxidation was performed following general procedure B (see below).

Synthesis of β-aminoketones by oxidation of azaboraspiro compounds (general procedure B):

After reaction workup, the crude was dissolved in 6 mL of THF/H_2O (1:1) and $NaBO_3{\cdot}4H_2O$ (1.05 mmol, 3.5 equiv) was added. The mixture was vigorously stirred at room temperature for 3 h. Then an aqueous saturated solution of NH_4Cl was added and it was extracted three times with Et_2O. The combined organic extracts were dried with anhydrous Na_2SO_4, filtered and solvents were evaporated under vacuum. The resulting α-aminoketone was purified by flash column chromatography on silica gel using a mixture of MeOH/Et_2O as eluent.

5. Product characterization

(3*S,3a*R**)-4',4',5',5'-Tetramethyl-2-methylene-3-phenylhexahydro-4*H*-1λ^4,8λ^4-spiro[[1,2]azaborolo[5,1-*c*][1,4]oxazine-1,2'-[1,3,2]dioxaborolane] (3)**

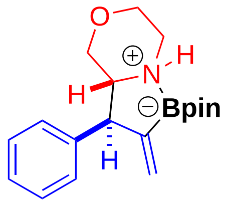

Synthesized from phenylallene **1** and morpholino benzoate **2** following the general procedure A at room temperature (5 h). Colorless oil obtained in 50% yield after flash column chromatography on deactivated silica gel (MeOH/Et_2O 3:97).

^{1}H-NMR (500 MHz, $CDCl_3$) δ 7.23 – 7.17 (m, 4H), 7.15 – 7.09 (m, 1H), 5.53 (t, *J* = 2.4 Hz, 1H), 5.18 (s, 1H), 3.75 (dt, *J* = 12.1, 4.3 Hz, 1H), 3.64 – 3.56 (m, 2H), 3.51 (dd, *J* = 12.2, 3.2 Hz, 1H), 3.44 (dd, *J* = 12.2, 4.2 Hz, 1H), 3.24 (dt, *J* = 11.7, 3.7 Hz, 1H), 2.95 (ddd, *J* = 13.8, 8.3, 3.5 Hz, 1H), 2.84 (ddd, *J* = 13.8, 5.1, 2.9 Hz, 1H), 1.15 (s, 12H). **^{13}C-NMR, DEPT** (75 MHz, $CDCl_3$) δ 142.0 (C), 129.3 (CH), 128.6 (CH), 126.7 (CH), 123.0 (CH_2), 81.2 (C), 67.7 (CH_2), 66.7 (CH_2), 58.2 (CH), 50.6 (CH), 42.1 (CH_2), 25.6 (CH_3), 25.5 (CH_3). **^{11}B-NMR** (160 MHz, $CDCl_3$) δ 17.36. **HRMS** (ESI, m/z): calculated for $C_{19}H_{29}BNO_3$ [M^+ + H]: 330.2235; found: 330.2237.

(3*S,3a*R**)-3-(4-Methoxyphenyl)-4',4',5',5'-tetramethyl-2-methylenehexahydro-4*H*-1λ^4,8λ^4-spiro[[1,2]azaborolo[5,1-*c*][1,4]oxazine-1,2'-[1,3,2]dioxaborolane] (7)**

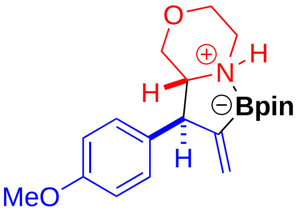

Synthesized from 1-methoxy-4-(propa-1,2-dien-1-yl)benzene **34** and morpholino benzoate **2** following the general procedure A at 70 ºC (1 h). White solid obtained in 57% yield after flash column chromatography on deactivated silica gel (MeOH/Et_2O 3:97).

^{1}H-NMR (500 MHz, $CDCl_3$) δ 7.17 (d, *J* = 8.7 Hz, 2H), 6.81 (d, *J* = 8.8 Hz, 2H), 5.58 (t, *J* = 2.4 Hz, 1H), 5.24 (s, 1H), 3.83 (dt, *J* = 12.0, 3.9 Hz, 1H), 3.77 (s, 3H), 3.64 (ddd, *J* = 11.6, 8.5, 2.7 Hz, 2H), 3.58 (dd, *J* = 12.2, 3.2 Hz, 1H), 3.51 (dd, *J* = 12.2, 4.0 Hz, 1H), 3.26 (dt, *J* = 11.7, 3.6 Hz, 1H), 3.01 (ddd, *J* = 13.8, 8.5, 3.5 Hz, 1H), 2.90 (ddd, *J* = 13.8, 4.8, 2.9 Hz, 1H), 1.22 (s, 12H). **^{13}C-NMR, DEPT** (75 MHz, $CDCl_3$) δ 158.4 (C), 134.0 (C),130.3 (2xCH), 122.6 (CH_2), 114.0 (2xCH), 81.0 (C), 67.4 (CH_2), 66.7 (CH_2), 58.3 (CH), 55.3 (CH_3), 49.6 (CH), 41.8 (CH_2), 25.6 (2xCH_3), 25.5(2xCH_3). **^{11}B-NMR** (160 MHz, $CDCl_3$) δ 16.58. **HRMS** (ESI, m/z): calculated for $C_{20}H_{31}BNO_3$ [M^+ + H]: 330.2235; found: 330.2238.

(3*S,3a*R**)-4',4',5',5'-Tetramethyl-2-methylene-3-(*m*-tolyl)hexahydro-4*H*-1λ^4,8λ^4-spiro[[1,2]azaborolo[5,1-*c*][1,4]oxazine-1,2'-[1,3,2]dioxaborolane] (8)**

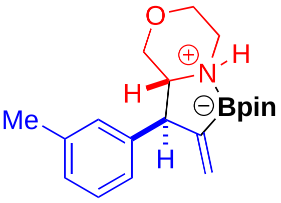

Synthesized from 1-methyl-3-(propa-1,2-dien-1-yl)benzene **35** and morpholino benzoate **2** following the general procedure A at 70 ºC (1 h). Colorless oil obtained in 74% yield after flash column chromatography on silica gel (MeOH/Et_2O 1.5:98.5).

^{1}H-NMR (500 MHz, $CDCl_3$) δ 7.15 (t, *J* = 7.6 Hz, 1H), 7.07 (s, 1H), 7.05 (d, *J* = 7.6 Hz, 1H), 7.00 (d, *J* = 7.6 Hz, 1H), 5.59 (s, 1H), 5.26 (s, 1H), 3.82 (dd, *J* = 7.9, 4.2 Hz, 1H), 3.69 – 3.62 (m, 2H), 3.58 (dd, *J* = 12.1, 3.0 Hz, 1H), 3.50 (dd, *J* = 12.1, 4.1 Hz, 1H), 3.33 (dt, *J* = 11.6, 3.4 Hz, 1H), 3.03 (ddd, *J* = 11.9, 8.2, 3.4 Hz, 1H), 2.91 (ddd, *J* = 13.7, 4.9, 2.9 Hz, 1H), 2.30 (s, 3H), 1.22 (s, 12H). **^{13}C-NMR, DEPT** (75 MHz, $CDCl_3$) δ 141.8 (C),

138.1 (C), 129.9 (CH), 128.4 (CH), 127.4 (CH), 126.3 (CH), 123.3 (CH_2), 81.2 (C), 67.9 (CH_2), 66.8 (CH_2), 58.0 (CH), 51.0 (CH), 42.2 (CH_2), 25.5 (4xCH_3), 21.5 (CH_3). **^{11}B-NMR** (160 MHz, $CDCl_3$) δ 18.57. **HMRS** (ESI, m/z): calculated for $C_{20}H_{31}BNO_3$ [M^+ + H]: 344.2392; found: 344.2398.

(3*S,3a*R**)-4',4',5',5'-Tetramethyl-2-methylene-3-(naphthalen-2-yl)hexahydro-4*H*-1λ^4,8λ^4-spiro[[1,2]azaborolo[5,1-*c*][1,4]oxazine-1,2'-[1,3,2]dioxaborolane] (9)**

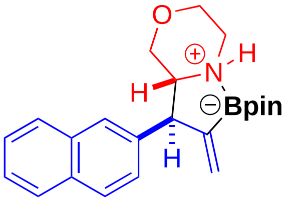

Synthesized from 2-(propa-1,2-dien-1-yl)naphthalene **36** and morpholino benzoate **2** following the general procedure A at 70 ºC (1 h). Colorless oil obtained in 74% yield after flash column chromatography on deactivated silica gel (MeOH/Et_2O 2:98).

^{1}H-NMR (500 MHz, $CDCl_3$) δ 7.81 – 7.74 (m, 3H), 7.71 (s, 1H), 7.47 – 7.39 (m, 3H), 5.63 (t, *J* = 2.5 Hz, 1H), 5.27 (s, 1H), 3.92 – 3.83 (m, 2H), 3.66 (ddd, *J* = 11.9, 8.8, 2.8 Hz, 1H), 3.62 – 3.53 (m, 2H), 3.43 (dt, *J* = 11.9, 3.7 Hz, 1H), 3.07 (ddd, *J* = 13.9, 8.9, 3.6 Hz, 1H), 2.94 (ddd, *J* = 13.9, 4.7, 2.8 Hz, 1H), 1.24 (s, 6H), 1.24 (s, 6H). **^{13}C-NMR, DEPT** (75 MHz, $CDCl_3$) δ 139.3 (C), 133.4 (C), 132.5 (C), 128.3 (CH), 128.1 (CH), 127.6 (2xCH), 127.2 (CH), 126.0 (CH), 125.5 (CH), 122.7 (CH_2), 80.9 (C), 67.1 (CH_2), 66.5 (CH_2), 57.9 (CH), 50.2 (CH), 41.6 (CH_2), 30.3 (CH), 25.5 (2xCH_3), 25.4 (2xCH_3). **^{11}B-NMR** (160 MHz, $CDCl_3$) δ 16.02. **HRMS** (ESI, m/z): calculated for $C_{23}H_{31}BNO_3$ [M^+ + H]: 380.2392; found: 380.2390.

(3*R,3a*S**)-4',4',5',5'-Tetramethyl-2-methylene-3-((*E*)-styryl)hexahydro-4*H*-1λ^4,8λ^4-spiro[[1,2]azaborolo[5,1-*c*][1,4]oxazine-1,2'-[1,3,2]dioxaborolane] (10)**

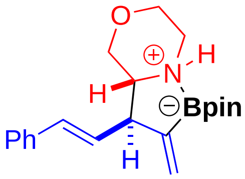

Synthesized from (*E*)-penta-1,3,4-trien-1-ylbenzene **37** and morpholino benzoate **2** following the general procedure A at 70 ºC (1 h). Brown solid obtained in 38% yield after flash column chromatography on deactivated silica gel (MeOH/Et_2O 1:99).

^{1}H-NMR (500 MHz, $CDCl_3$) δ 7.27 (d, *J* = 7.2 Hz, 2H), 7.25 – 7.19 (m, 2H), 7.13 (t, *J* = 7.2 Hz, 1H), 6.37 (d, *J* = 15.8 Hz, 1H), 6.02 (dd, *J* = 15.7, 9.0 Hz, 1H), 5.62 (s,1H), 5.43 (s, 1H), 3.73 (ddd, *J* = 12.3, 8.7, 4.0 Hz, 2H), 3.64 – 3.56 (m, 2H), 3.26 – 3.19 (m, 1H), 2.97 – 2.86 (m, 2H), 2.80 (ddd, *J* = 13.6, 5.2, 2.9 Hz, 1H), 1.16 (s, 12H). **^{13}C-NMR, DEPT** (75 MHz, $CDCl_3$) δ 137.2 (C), 132.9 (CH), 129.7 (CH), 128.6 (2xCH), 127.4 (CH), 126.3 (2xCH), 123.0 (CH_2), 81.1 (C), 67.5 (CH_2), 66.5 (CH_2), 56.6 (CH), 48.7 (CH), 42.0 (CH_2), 25.5 (2xCH_3), 25.4 (2xCH_3). **^{11}B-NMR** (160 MHz, $CDCl_3$) δ 16.73. **HRMS** (ESI, m/z): calculated for $C_{21}H_{31}BNO_3$ [M^+ + H]: 356.2392; found: 356.2394.

(3*R,3a*R**)-3-Cyclohexyl-4',4',5',5'-tetramethyl-2-methylenehexahydro-4*H*-1λ^4,8λ^4-spiro[[1,2]azaborolo[5,1-*c*][1,4]oxazine-1,2'-[1,3,2]dioxaborolane] (11)**

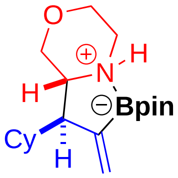

Synthesized from cyclohexylallene **38** and morpholino benzoate **2** following the general procedure A at room temperature (5 h). White solid obtained in 35% after crystallization in Et_2O saturating the atmosphere with pentane.

^{1}H-NMR (500 MHz, $CDCl_3$) δ 5.65 (d, *J* = 3.4 Hz, 1H), 5.41 (d, *J* = 3.4 Hz, 1H), 3.77 (dd, *J* = 11.7, 3.2 Hz, 1H), 3.69 – 3.58 (m, 2H), 3.50 – 3.45 (m, 1H), 3.14 (ddd, *J* = 8.1, 6.9, 3.2 Hz, 1H), 2.85 (t, *J* = 4.8 Hz, 2H), 2.12 (t, *J* = 6.9 Hz, 1H), 1.75 – 1.65 (m, 3 H), 1.65 – 1.59 (m, 1H), 1.50 (dddd, *J* = 11.6, 8.8, 5.2, 2.3 Hz, 1H), 1.28 (m, 1H),

1.21 (s, 6H), 1.19 (s, 6H), 1.18 – 1.0 (m, 3 H), 0.88 (t, *J* = 7.1 Hz, 2H). ^{13}**C-NMR, DEPT** (75 MHz, $CDCl_3$) δ 125.2 (CH_2), 81.7 (C), 70.6 (CH_2), 66.9 (CH_2), 54.4 (CH), 51.2 (CH), 44.4 (CH_2), 39.0 (CH), 31.8 (CH_2), 30.4 (CH_2), 26.9 (CH_2), 26.8 (CH_2), 26.7 (CH_2), 25.3 (2xCH_3), 25.1 (2xCH_3). ^{11}**B-NMR** (160 MHz, $CDCl_3$) δ 17.33. **HRMS** (ESI, m/z): calculated for $C_{19}H_{35}BNO_3$ [M^+ + H]: 336.2705; found: 336.2703.

(*S)-1-Cyclohexyl-1-((*R**)-morpholin-3-yl)propan-2-one (11-[O])**

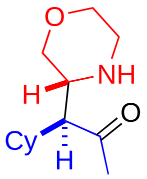

Synthesized from cyclohexylallene **38** and morpholino benzoate **2** following general procedure B. Colorless oil obtained in 68% yield after flash column chromatography on silica gel (MeOH/Et_2O 3:97).

^{1}**H-NMR** (300 MHz, $CDCl_3$) δ 3.86 – 3.81 (m, 1H), 3.76 (dt, *J* = 11.2, 2.7 Hz, 1H), 3.47 – 3.36 (m, 1H), 3.22 – 3.10 (m, 2H), 2.88 (dd, *J* = 10.0, 3.1 Hz, 2H), 2.44 – 2.38 (m, 1H), 2.20 (s, 3H), 1.90 – 1.60 (m, 7H), 1.32-1.12 (m, 2H), 1.11-0.87 (m, 2H). ^{13}**C-NMR, DEPT** (75 MHz, $CDCl_3$) δ 212.5 (C), 71.3 (CH_2), 67.9 (CH_2), 59.7 (CH), 53.7 (CH), 46.1 (CH_2), 37.1 (CH), 34.1 (CH_3), 31.6 (CH_2), 30.2 (CH_2), 26.5 (CH_2), 26.3 (2xCH_2). **HRMS** (ESI, m/z): calculated for $C_{13}H_{24}NO_2$ [M^+ + H]: 226.1802; found: 226.1804.

(*S)-3-((*R**)-Morpholin-3-yl)undecan-2-one (12-[O]) + (*S**)-3-((*S**)-morpholin-3-yl)undecan-2-one (12’-[O])**

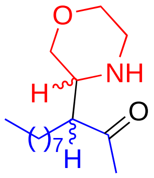

Synthesized from undeca-1,2-diene **39** and morpholino benzoate **2** following general procedure B. Colourless oil obtained in 75% combined yield as an 80:20 mixture after flash column chromatography on silica gel (EtOAc/*n*-hexane 1:1).*

^{1}**H-NMR** (500 MHz, $CDCl_3$) δ 3.84 (dd, *J* = 11.1, 3.1 Hz, 1H **12-[O]**), 3.78 – 3.72 (m, 1H **12-[O]** + 1H **12’-[O]**), 3.67 (dd, *J* = 11.1, 3.0 Hz, 1H **12’-[O]**), 3.49 – 3.45 (m, 1H **12’-[O]**), 3.43 (td, *J* = 11.0, 2.9 Hz, 1H, **12-[O]**), 3.29 (dd, *J* = 11.1, 9.5 Hz, 1H **12’-[O]**), 3.20 (dd, *J* = 11.1, 9.7 Hz, 1H **12-[O]**), 3.00 (ddd, *J* = 9.6, 8.5, 3.1 Hz, 1H **12-[O]**), 2.95 (ddd, *J* = 9.4, 6.4, 2.9 Hz, 1H **12’-[O]**), 2.92 – 2.86 (m, 1H **12-[O]** + 1H **12’-[O]**), 2.82 (dt, *J* = 12.6, 2.5 Hz, 1H **12-[O]**), 2.52 (ddd, *J* = 10.3, 6.4, 4.0 Hz, 1H **12’-[O]**), 2.45 (td, *J* = 8.7, 4.4 Hz, 1H **12-[O]**), 2.17 (s, 3H **12’-[O]**), 2.16 (s, 3H **12-[O]**), 1.70 – 1.61 (m, 1H **12’-[O]**), 1.60 – 1.43 (m, 2H **12-[O]** + 1H **12’-[O]**), 1.32 – 1.13 (m, 12H **12-[O]** + 12 H **12’-[O]**), 0.86 (t, *J* = 7.0 Hz, 3H **12-[O]** + 3H **12’-[O]**). ^{13}**C-NMR, DEPT** (126 MHz, $CDCl_3$) δ 211.9 (C **12-[O]**), 211.5 (C **12’-[O]**), 70.9 (CH_2 **12-[O]**), 70.6 (CH_2 **12’-[O]**), 67.9 (CH_2 **12-[O]**), 67.7 (CH_2 **12’-[O]**), 55.9 (CH **12’-[O]**), 55.6 (CH **12-[O]**), 55.3 (CH **12-[O]**), 54.6 (CH **12’-[O]**), 46.2 (CH_2 **12’-[O]**), 45.9 (CH_2 **12-[O]**), 31.9 (CH_2 **12-[O]**+ **12’-[O]**), 30.4 (CH_3 **12’-[O]**), 30.4 (CH_3 **12-[O]**), 29.9 (CH_2 **12-[O]** + **12’-[O]**), 29.5 (CH_2 **12’-[O]**), 29.4 (CH_2 **12-[O]**), 29.3 (CH_2 **12’-[O]**), 29.3 (CH_2 **12-[O]**), 28.7 (CH_2 **12-[O]**), 27.9 (CH_2 **12’-[O]**), 27.8 (CH_2 **12’-[O]**), 27.2 (CH_2 **12-[O]**), 22.7 (CH_2 **12-[O]** + **12’-[O]**), 14.2 (CH_2 **12-[O]** + **12’-[O]**). **HRMS** (ESI, m/z): calculated for $C_{15}H_{30}NO_2$ [M^+ + H]: 256.2271; found: 256.2273.

(*S)-3-(*R**)-Morpholin-3-yl)-5-phenylpentan-2-one (13-[O]) + (*S**)-3-((*S**)-morpholin-3-yl)-5-phenylpentan-2-one (13’-[O])**

Synthesized from penta-3,4-dien-1-ylbenzene **40** and morpholino benzoate **2** following the general procedure B. Colourless oil obtained in 82% combined yield as a 60:40 mixture of

diastereomers. Both diastereomers could be separated by flash column chromatography on silica gel (EtOAc/hexanes 8:2).

(*S)-3-((*R**)-Morpholin-3-yl)-5-phenylpentan-2-one (13-[O])**

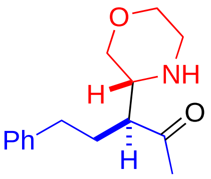

^{1}H-NMR (300 MHz, $CDCl_3$) δ 7.34 – 7.13 (m, 5H), 3.76 (dt, *J* = 11.1, 2.8 Hz, 1H), 3.69 (dd, *J* = 11.1, 2.9 Hz, 1H), 3.53 – 3.42 (m, 1H), 3.31 (t, *J* = 10.3 Hz, 1H), 3.05 – 2.94 (m, 1H), 2.93 – 2.86 (m, 2H), 2.65 – 2.43 (m, 3H), 2.21 (s, 3H), 2.15 – 1.98 (m, 1H), 1.91 – 1.80 (m, 1H). **^{13}C-NMR, DEPT** (75 MHz, $CDCl_3$) δ 211.1 (C), 141.3 (C), 128.6 (2xCH), 128.5 (2xCH), 126.3 (CH), 70.5 (CH_2), 67.7 (CH_2), 56.0 (CH), 53.9 (CH), 46.2 (CH_2), 33.9 (CH_2), 31.6 (CH_3), 29.4 (CH_2). **HRMS** (ESI, m/z): calculated for $C_{15}H_{22}NO_2$ [M^+ + H]: 248.1645; found: 248.1650.

(*S)-3-((*S**)-Morpholin-3-yl)-5-phenylpentan-2-one (13'-[O])**

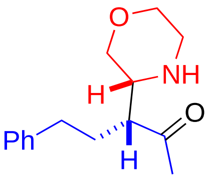

^{1}H-NMR (300 MHz, $CDCl_3$) δ 7.34-7.15 (m, 5H), 3.87 (dd, *J* = 11.1, 2.9 Hz, 1H), 3.83 – 3.72 (m, 2H), 3.45 (td, *J* = 10.8, 3.4 Hz, 1H), 3.23 (dd, *J* = 11.0, 9.8 Hz, 1H), 3.08 (td, *J* = 10.0, 9.1, 2.9 Hz, 1H), 2.99 – 2.82 (m, 2H), 2.68 – 2.43 (m, 2H), 2.20 (d, *J* = 1.0 Hz, 3H), 2.02 – 1.80 (m, 3H), 1.73 (s, 1H). **^{13}C-NMR, DEPT** (75 MHz, $CDCl_3$) δ 211.5 (C), 141.1 (C), 128.7 (2xCH), 128.4 (2xCH), 126.4 (CH), 70.8 (CH_2), 67.9 (CH_2), 55.7 (CH), 54.7 (CH), 46.0 (CH_2), 33.5 (CH_2), 30.6 (CH_3), 30.3 (CH_2). **HRMS** (ESI, m/z): calculated for $C_{15}H_{22}NO_2$ [M^+ + H]: 248.1645; found: 248.1647.

3,3,4',4',5',5'-Hexamethyl-2-methylenehexahydro-4*H*-1λ^4,8λ^4-spiro[[1,2]azaborolo[5,1-*c*][1,4]oxazine-1,2'-[1,3,2]dioxaborolane] (14)

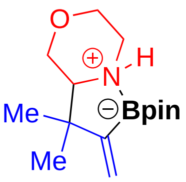

Synthesized from 3-methylbuta-1,2-diene **41** and morpholino benzoate **2** following the general procedure A at room temperature (5 h). colourless oil obtained in 47% yield after flash column chromatography on silica gel (MeOH/Et_2O 3:97).

^{1}H-NMR (300 MHz, $CDCl_3$) δ 5.39 (d, *J* = 2.2 Hz, 1H), 5.29 (d, *J* = 2.3 Hz, 1H), 3.95 (dd, *J* = 12.0, 3.2 Hz, 1H), 3.87 (dt, *J* = 12.1, 3.0 Hz, 1H), 3.62 – 3.51 (m, 1H), 3.41 (dd, *J* = 11.9, 9.5 Hz, 1H), 3.07 – 2.96 (m, 2H), 2.76 (dd, *J* = 9.6, 3.2 Hz, 1H), 1.20 (s, 12H), 1.10 (s, 3H), 0.94 (s, 3H). **^{13}C-NMR, DEPT** (75 MHz, $CDCl_3$) δ 116.4 (CH_2), 80.4 (C), 67.7 (CH_2), 66.2 (CH_2), 63.5 (CH), 44.5 (CH_2), 40.8 (C), 26.5 (2xCH_3), 26.1 (3xCH_3), 23.7 (CH_3). **^{11}B-NMR** (160 MHz, CDC13) δ 16.17. **HRMS** (ESI, m/z): calculated for $C_{15}H_{29}BNO_3$ [M^+ + H]: 282.2235; found: 282.2237.

(3*S,3a*S**)-3-Cyclohexyl-4',4',5',5'-tetramethyl-2-methyleneoctahydro-1λ^4,8λ^4-spiro[[1,2]azaborolo[1,5-*a*]pyridine-1,2'-[1,3,2]dioxaborolane] (15)**

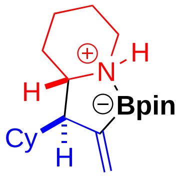

Synthesized from cyclohexylallene **38** and piperidin-1-yl benzoate **42** following the general procedure A at room temperature (5 h). White solid obtained in 40% yield after precipitation in Et_2O filtration.

^{1}H-NMR (500 MHz, $CDCl_3$) δ 5.38 (s, 1H), 5.19 (s, 1H), 3.03 – 2.94 (m, 1H), 2.92 – 2.83 (m, 1H), 2.79 – 2.71 (m, 1H), 2.05 – 1.98 (m, 1H), 1.90 – 1.80 (m, 2H), 1.75 – 1.63 (m, 5H), 1.63 – 1.54 (m, 3H), 1.53 – 1.34 (m, 4H), 1.24 (m, 1H), l.15 (s, 6 H),

1.12 (s, 6H), 1.03(m, 2H). **^{13}C-NMR, DEPT** (126 MHz, $CDCl_3$) δ 116.7 (CH_2), 79.6 (C), 58.2 (CH), 51.0 (CH), 43.1 (CH_2), 40.8 (CH), 31.5 (CH_2), 30.6 (CH_2), 29.8 (CH_2), 27.1 (CH_2), 27.1 (CH_2), 26.8 (CH_2), 26.2 (2xCH_3), 25.7 (2xCH_3), 25.2 (CH_2), 21.5 (CH_2). **^{11}B-NMR** (160 MHz, $CDCl_3$) δ 11.70. **HRMS** (ESI, m/z): calculated for $C_{20}H_{37}BNO_2$ [M^+ + H]: 334.2912; found: 334.2913.

(3*R,3a*S**,5*S**)-3-Cyclohexyl-4',4',5,5',5'-pentamethyl-2-methyleneoctahydro-1λ^4,8λ^4-spiro[[1,2]azaborolo[1,5-*a*]pyridine-1,2'-[1,3,2]dioxaborolane] (16)**

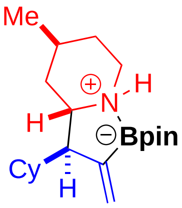

Synthesized from cyclohexylallene **38** and 4-methylpiperidin-1-yl benzoate **43** following the general procedure A at 50 °C (1 h). Colourless oil obtained in 73% yield after flash column chromatography on silica gel (MeOH/Et_2O 1.5:98.5).

^{1}H-NMR (500 MHz, $CDCl_3$) δ 5.41 (dd, *J* = 3.4, 2.2 Hz, 1H), 5.28 (dd, *J* = 3.5, 1.8 Hz, 1H), 3.39 (ddd, *J* = 10.8, 5.5, 2.0 Hz, 1H), 2.94 (dt, *J* = 14.1, 3.3 Hz, 1H), 2.47 (td, *J* = 13.5, 3.0 Hz, 1H), 2.21 – 2.15 (m, 1H), 1.85 – 1.79 (m, 1H), 1.78 – 1.61 (m, 8H), 1.42 – 1.30 (m, 3H), 1.21 (m, 2H) 1.16 (s, 6H), 1.12 (s, 6H), 1.05 (m, 2H), 0.95 (d, *J* = 6.4 Hz, 3H). **^{13}C-NMR** (75 MHz, $CDCl_3$) δ 117.1 (CH_2), 79.4 (C), 55.4 (CH), 47.5 (CH), 41.6 (CH), 41.1 (CH_2), 35.4 (CH_2), 33.2 (CH_2), 32.0 (CH_2), 30.0 (CH_2), 27.4 (CH_2), 27.2 (CH_2), 26.8 (CH_2), 25.7 (2xCH_3), 25.2 (2xCH_3), 24.6 (CH), 22.0 (CH_3). **^{11}B-NMR** (160 MHz, $CDCl_3$) δ 11.05. **HRMS** (ESI, m/z): calculated for $C_{21}H_{39}BNO_2$ [M^+ + H]: 348.3068; found: 348.3066.

Ethyl (3*R,3a*S**,5*S**)-3-cyclohexyl-4',4',5',5'-tetramethyl-2-methyleneoctahydro-1λ^4,8λ^4-spiro[[1,2]azaborolo[1,5-*a*]pyridine-1,2'-[1,3,2]dioxaborolane]-5-carboxylate (17)**

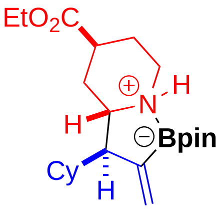

Synthesized from cyclohexylallene **38** and ethyl 1-(benzoyloxy)piperidine-4-carboxylate **51** following general procedure A at rt (1 h). White solid obtained in 60% yield after flash column chromatography on deactivated silica gel (Et_2O).

^{1}H NMR (300 MHz, $CDCl_3$) δ 5.43 (s, 1H), 5.36 – 5.21 (m, 1H), 4.14 (q, *J* = 7.1 Hz, 2H), 3.40 – 3.28 (m, 1H), 2.94 (d, *J* = 13.9 Hz, 1H), 2.60 (s, 2H), 2.15 – 2.02 (m, 1H), 1.94 (q, *J* = 4.4 Hz, 3H), 1.69 (t, *J* = 13.5 Hz, 7H), 1.41 (s, 2H), 1.29 – 1.17 (m, 7H), 1.15 (s, 6H), 1.11 (s, 6H). **^{13}C NMR** (126 MHz, $CDCl_3$) ^{13}C NMR (126 MHz, $CDCl_3$) δ 174.2 (C), 118.3 (CH_2), 79.8 (C), 60.8 (CH_2), 54.6 (CH), 40.6 (CH_2), 36.1 (CH), 31.7 (CH_2), 30.3 (CH), 29.7 (CH_2), 29.7 (CH_2), 27.0 (CH_2), 26.9 (CH_2), 26.6 (CH_2), 25.6 (2xCH_3), 25.2 (CH_3), 24.9 (CH_3), 14.2 (CH_3). **^{11}B-NMR** (160 MHz, $CDCl_3$) δ 13.7. **HRMS** (APCI, m/z): calculated for $C_{23}H_{41}BNO_4$ [M^+ + H]: 406.3129; found:406.3130.

(3*R,3a*S**,5*S**)-3-Cyclohexyl-4',4',5',5'-tetramethyl-2-methylene-5-((trimethylsilyl)oxy)octahydro-1λ^4,8λ^4-spiro[[1,2]azaborolo[1,5-*a*]pyridine-1,2'-[1,3,2]dioxaborolane] (18)**

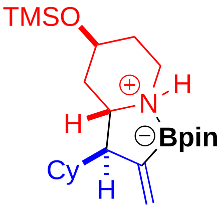

Synthesized from cyclohexylallene **38** and 4-((trimethylsilyl)oxy)piperidin-1-yl benzoate **50** following general procedure A at rt (1 h). Colorless oil obtained in 55% yield after flash column chromatography on deactivated silica gel (Et_2O).

^{1}H NMR (300 MHz, $CDCl_3$) δ 5.40 (d, *J* = 3.1 Hz, 1H), 5.22 (s, 1H), 3.91 (d, *J* = 6.5 Hz, 1H), 3.47 – 3.30 (m, 1H), 3.10 – 2.95 (m, 1H), 2.72 (d, *J* = 12.4 Hz, 1H), 1.97 (s, 1H), 1.88 – 1.57 (m, 9H), 1.41 (s, 3H),1.17-1.18 (m, 4H) 1.16 (s, 6H), 1.13 (s, 6H), 0.10 (s, 9H). **^{13}C NMR** (126 MHz, $CDCl_3$) δ 117.4 (CH_2), 79.7 (C), 64.4 (CH), 55.0 (CH), 40.0 (CH_2), 38.0 (CH_2), 33.7 (CH_2), 31.4 (CH_2), 31.2 (CH_2), 30.9 (CH_2), 30.5 (CH), 27.0 (CH_2), 26.8 (CH_2), 26.0 (2xCH_3), 25.6 (2xCH_3), 25.0 (CH), 0.3 (3xCH_3). **^{11}B-NMR** (160 MHz, $CDCl_3$) δ 11.7. **HRMS** (APCI, m/z): calculated for $C_{23}H_{45}BNO_3Si$ [M^+ + H]: 422.3262; found: 422.3270.

(3*R,3a*S**,5*S**)-5-Chloro-3-cyclohexyl-4',4',5',5'-tetramethyl-2-methyleneoctahydro-1λ^4,8λ^4-spiro[[1,2]azaborolo[1,5-*a*]pyridine-1,2'-[1,3,2]dioxaborolane] (19)**

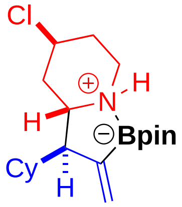

Synthesized from cyclohexylallene **38** and 4 4-chloropiperidin-1-yl benzoate **47** following general procedure A at rt (1 h). Yellow solid obtained in 55% yield after flash column chromatography on deactivated silica gel ($MeOH/Et_2O$ 1.5:98.5).

^{1}H NMR (500 MHz, $CDCl_3$) δ 5.58 (s, 1H), 5.35 (s, 1H), 4.39 (t, *J* = 4.7 Hz, 1H), 3.45 (s, 1H), 3.11 (ddd, *J* = 13.1, 9.6, 3.2 Hz, 1H), 2.89 (s, 1H), 2.11 (s, 1H), 2.01 – 1.92 (m, 3H), 1.84 (dt, *J* = 13.8, 4.5 Hz, 1H), 1.75 – 1.59 (m, 7H), 1.49 (dtt, *J* = 8.7, 6.0, 2.9 Hz, 1H), 1.20 (s, 7H), 1.19 (s, 7H), 1.11 – 1.01 (m, 2H), 0.96 – 0.85 (m, 1H). **^{13}C NMR** (126 MHz, $CDCl_3$) δ 125.5 (CH_2), 81.1 (C), 55.9 (CH), 53.7 (CH), 52.5 (CH_2), 40.1 (CH_2), 38.8 (CH), 38.1 (CH_2), 31.5 (CH_2), 30.3 (CH_2), 26.7 (CH_2), 26.7 (CH_2), 26.6 (CH_2), 25.5 (2xCH_3), 25.2 (2xCH_3), 24.9 (CH). **^{11}B-NMR** (160 MHz, $CDCl_3$) δ 11.2. **HRMS** (APCI, m/z): calculated for $C_{20}H_{36}BClNO_2$ [M^+ + H]: 368.2528; found:368.2523.

(3*R,3a*S**)-3-Cyclohexyl-4',4',5',5'-tetramethyl-2-methylene-2,3,3a,4,7,8-hexahydro-1λ^4,8λ^4-spiro[[1,2]azaborolo[1,5-*a*]pyridine-1,2'-[1,3,2]dioxaborolane] (20)**

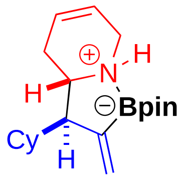

Synthesized from cyclohexylallene **38** and 4-(acryloyloxy)piperidin-1-yl benzoate **49** following general procedure A at rt (1 h). White solid obtained in 50% yield after flash column chromatography on deactivated silica gel ($MeOH/Et_2O$ 1.5:98.5).

^{1}H NMR (500 MHz, $CDCl_3$) δ 5.82 – 5.75 (m, 1H), 5.75 – 5.69 (m, 1H), 5.34 (dd, *J* = 3.3, 2.1 Hz, 1H), 5.17 – 5.12 (m, 1H), 3.61 – 3.51 (m, 1H), 3.13 – 3.03 (m, 1H), 2.47 (ddd, *J* = 13.6, 11.2, 5.3 Hz, 1H), 2.16 – 1.98 (m, 3H), 1.75 – 1.62 (m, 5H), 1.62 – 1.54 (m, 1H), 1.44 (dddd, *J* = 11.7, 8.7, 5.5, 2.8 Hz, 1H), 1.10 (s, 6H), 1.07 (s, 6H). **^{13}C NMR** (126 MHz, $CDCl_3$) δ 128.6 (CH), 123.5 (CH), 117.2 (CH_2), 79.5 (C), 55.2 (CH), 53.5 (CH), 41.4 (CH), 37.9 (CH_2), 31.2 (CH_2), 30.7 (CH_2), 29.7 (CH_2), 26.9 (CH_2), 26.8 (CH_2), 26.7 (CH_2), 25.5 (2xCH_3), 25.1 (2xCH_3), 24.9 (CH), 23.4 (CH_2). **^{11}B-NMR** (160 MHz, $CDCl_3$) δ 11.3. **HRMS** (APCI, m/z): calculated for $C_{20}H_{35}BNO_2$ [M^+ + H]: 332.2759; found: 332.2764.

tert-Butyl (R*)-3-((S*)-1-cyclohexyl-2-oxopropyl)piperazine-1-carboxylate (21-[O])

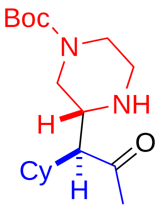

Synthesized from cyclohexylallene **38** and *tert*-butyl 4-benzoylpiperazine-1-carboxylate **44** following the general procedure B. Colourless oil obtained in 57% yield after flash column chromatography on silica gel (MeOH/Et_2O 3:97). **^{1}H-NMR** (500 MHz, $CDCl_3$) δ 4.13 – 3.79 (m, 1H), 3.56 – 3.26 (m, 1H), 2.98 – 2.87 (m, 2H), 2.80 – 2.62 (m, 2H), 2.47 (t, *J* = 7.1 Hz, 1H), 2.20 (s, 3H), 1.85 – 1.60 (m, 7H), 1.47 (s, 9H), 1.33 – 0.95 (m, 6H). **^{13}C-NMR, DEPT** (126 MHz, $CDCl_3$) δ 212.9 (C), 154.7 (C), 79.9 (C), 60.6 (CH), 54.1 (CH), 45.6 (CH_2), 37.4 (CH), 31.7 (2xCH_2), 28.5 (3xCH_3), 26.7 (CH_2), 26.5 (CH_2), 26.4 (3xCH_2). **HRMS** (ESI, m/z): calculated for $C_{18}H_{33}N_2O_3$ [M^+ + H]: 325.2486; found: 325.2488.

(S*)-1-Cyclohexyl-1-((S*)-pyrrolidin-2-yl)propan-2-one (22-[O])

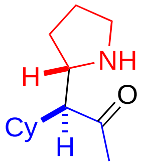

Synthesized from cyclohexylallene **38** and pyrrolidin-1-yl benzoate **45** following general procedure B. Colourless oil obtained in 52% yield after flash column chromatography on silica gel (MeOH/Et_2O 5:95).
^{1}H-NMR (500 MHz, $CDCl_3$) δ 3.92 – 3.84 (m, 1H), 3.50 – 3.41 (m, 1H), 3.34 – 3.26 (m, 1H), 3.13 (dd, *J* = 8.2, 4.4 Hz, 1H), 2.37 (s, 3H), 2.24 – 2.16 (m, 1H), 2.11 – 1.59 (m, 9H), 1.31 – 0.98 (m, 5H). **^{13}C-NMR, DEPT** (126 MHz, $CDCl_3$) δ 212.6 (C), 59.3 (CH), 59.1 (CH), 45.7 (CH_2), 39.9 (CH), 33.6 (CH_3), 31.8 (CH_2), 30.4 (CH_2), 30.1 (CH_2), 27.1 (CH_2), 26.9 (CH_2), 26.4 (CH_2), 24.3 (CH_2). **HRMS** (ESI, m/z): calculated for $C_{13}H_{24}NO$ [M^+ + H]: 210.1852; found: 210.1855.

(S*)-1-((S*)-Azepan-2-yl)-1-cyclohexylpropan-2-one (23-[O])

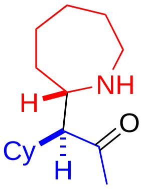

Synthesized from cyclohexylallene **38** and azepan-1-yl benzoate **46** following the general procedure B. Colourless oil obtained in 55% yield after flash column chromatography on silica gel (MeOH/Et_2O 1.5:98.5).
^{1}H-NMR (500 MHz, $CDCl_3$) δ 2.97 (ddd, *J* = 10.6, 7.3, 3.6 Hz, 1H), 2.89 (ddd, *J* = 14.0, 7.0, 4.2 Hz, 1H), 2.68 (ddd, *J* = 14.1, 7.6, 4.2 Hz, 1H), 2.39 (t, *J* = 7.3 Hz, 1H), 2.16 (s, 3H), 1.85 (ddt, *J* = 13.3, 6.5, 3.4 Hz, 2H), 1.80 – 1.60 (m, 7H), 1.60 – 1.50 (m, 3H), 1.20 – 1.03 (m, 4H). **^{13}C-NMR, DEPT** (126 MHz, $CDCl_3$) δ 214.1 (C), 64.6 (CH), 57.2 (CH), 47.1 (CH_2), 37.9 (CH), 35.3 (CH_2), 34.1 (CH_3), 31.9 (CH_2), 29.9 (CH_2), 27.2 (CH_2), 27.0 (CH_2), 26.8 (CH_2), 26.6 (CH_2), 26.6 (CH_2). **HRMS** (ESI, m/z): calculated for $C_{15}H_{28}NO$ [M^+ + H]: 238.2165; found: 238.2168.

6. Product derivatization

6.1. Heck coupling

A 5 mL sealed tube equipped with stirring magnetic bar was flamed-dried under vacuum, cooled to rt and backfilled with argon. Then it was charged with $Pd(OAc)_2$ (0.02 mmol, 0.1 equiv), SPhos (0.024 mmol, 0.12 equiv), K_2CO_3 (0.6 mmol, 3 equiv), the corresponding aryl (pseudo)halide (0.4 mmol, 2 equiv) and **16** (0.2 mmol, 1 equiv). Afterwards, it was put in vacuum and backfilled with argon for 3 times. Then 2 mL of THF were added and the reaction was heated to 70 °C for 16 h. The reaction was cooled to rt and quenched with a saturated solution of NH_4Cl(aq) (20 mL) and extracted with EtOAc (2 x 20 mL). The combination of organic phases was dried over Na_2SO_4 and concentrated in vacuo. The residue was then purified using as eluent a mixture of EtOAc/Hexanes to give the pure product.

(3*R,3a*S**,5*S**)-2-((*E*)-2-bromobenzylidene)-3-cyclohexyl-4',4',5,5',5'-pentamethyloctahydro-1λ4,8λ4-spiro[[1,2]azaborolo[1,5-*a*]pyridine-1,2'-[1,3,2]dioxaborolane]** (**24**)

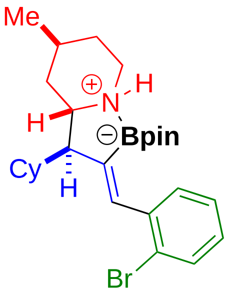

Synthesized from **16** and 1-bromo-2-iodobenzene. White foam isolated in 76% yield after flash column chromatography on silica gel (EtOAc/Hexanes 7:3).

^{1}H NMR (300 MHz, $CDCl_3$) δ 7.53 (d, *J* = 7.9 Hz, 1H), 7.23 – 7.14 (m, 2H), 7.01 (ddd, *J* = 8.8, 5.6, 3.4 Hz, 1H), 6.71 (d, *J* = 2.3 Hz, 1H), 3.49 (dd, *J* = 7.9, 3.2 Hz, 1H), 3.04 (d, *J* = 13.7 Hz, 1H), 2.74 (dt, *J* = 9.7, 3.2 Hz, 1H), 2.60 (t, *J* = 13.5 Hz, 1H), 1.84 – 1.24 (m, 15H), 1.20 (s, 6H), 1.18 (s, 6H), 1.15-1.05 (m, 2H) 0.96 (d, *J* = 6.2 Hz, 3H). **^{13}C NMR** (126 MHz, $CDCl_3$) δ 140.9 (C), 131.2 (CH), 129.7 (CH), 128.4 (CH), 126.2 (CH), 125.7 (CH), 122.9 (C), 78.4 (C), 74.0 (C), 54.0 (CH), 42.5 (CH), 40.6 (CH_2), 35.3 (CH_2), 31.9 (CH_2), 30.8 (CH_2), 28.9 (CH_2), 26.3 (CH_2), 26.1 (CH_2), 25.5 (CH_2), 24.8 (CH), 24.0 (CH_3), 23.9 (CH_3), 23.8 (CH), 20.9 (CH_3). **^{11}B NMR** (160 MHz, $CDCl_3$) δ 9.9. **HRMS** (ESI, m/z): calculated for $C_{27}H_{42}BBrNO_2$ [M^+ + H]: 502.2486; found: 502.2493.

(3*R,3a*S**,5*S**)-3-cyclohexyl-4',4',5,5',5'-pentamethyl-2-((*E*)-4-nitrobenzylidene)octahydro-1λ4,8λ4-spiro[[1,2]azaborolo[1,5-*a*]pyridine-1,2'-[1,3,2]dioxaborolane]** (**25**)

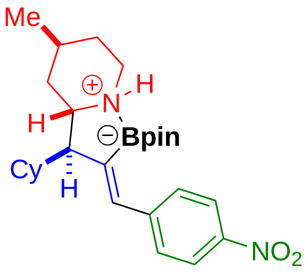

Synthesized from **16** and 1-iodo-4-nitrobenzene White foam isolated in 80% yield after flash column chromatography on silica gel (EtOAc/Hexanes 7:3).

^{1}H NMR (500 MHz, $CDCl_3$) δ 8.2 – 8.1 (m, 2H), 7.4 (d, *J* = 8.4 Hz, 2H), 6.8 (d, *J* = 2.4 Hz, 1H), 3.6 (t, *J* = 7.7 Hz, 1H), 3.0 (dt, *J* = 14.1, 3.3 Hz, 1H), 3.0 – 2.9 (m, 1H), 2.5 (t, *J* = 13.4 Hz, 1H), 2.0 (s, 2H), 1.8 (d, *J* = 14.3 Hz, 2H), 1.77-1.51 (m, 13H), 1.4 (ddd, *J* = 14.1, 11.5, 5.5 Hz, 1H), 1.2 (s, 6H), 1.1 (s, 6H), 1.0 (d, *J* = 6.2 Hz, 3H). **^{13}C NMR** (126 MHz, $CDCl_3$) δ

147.6 (C), 145.3 (C), 128.8 (CH), 128.5 (CH), 123.5 (CH), 79.6 (C), 75.0 (C), 54.8 (CH), 41.7 (CH), 41.6 (CH_2), 36.7 (CH_2), 32.6 (CH_2), 29.9 (CH_2), 27.2 (CH_2), 26.9 (CH_2), 26.5 (CH_2), 25.9 (CH_3), 25.0 (CH_3), 24.9 (CH_3), 24.8 (CH_3), 24.7 (CH), 21.9 (CH_3). **^{11}B NMR** (160 MHz, $CDCl_3$) δ 11.6. **HRMS** (ESI, m/z): calculated for $C_{27}H_{42}BN_2O_4$[M^+ + H]: 469.3232; found: 469.3240.

(3*R,3a*S**,5*S**)-3-cyclohexyl-2-((*E*)-4-methoxybenzylidene)-4',4',5,5',5'-pentamethyloctahydro-1λ4,8λ4-spiro[[1,2]azaborolo[1,5-*a*]pyridine-1,2'-[1,3,2]dioxaborolane]** (**26**)

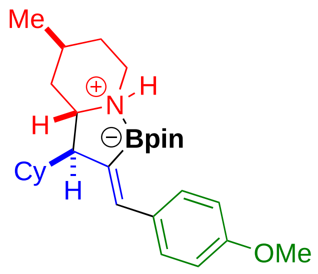

Synthesized from **16** and 1-iodo-4-methoxybenzene White foam isolated in 50% yield (full conv.) after flash column chromatography on silica gel (EtOAc/Hexanes 6:4).
Using 1-bromo-4-methoxybenzene: 25% yield (40% conv.)
Using 4-methoxyphenyl trifluoromethanesulfonate: 35% yield (50% conv.).
^{1}H NMR (500 MHz, $CDCl_3$) δ 7.2 (d, J = 8.2 Hz, 2H), 6.8 (d, J = 8.2 Hz, 2H), 6.7 (s, 1H), 3.8 (s, 3H), 3.6 (s, 1H), 3.0 – 2.8 (m, 2H), 2.5 (d, J = 15.1 Hz, 1H), 2.0 – 1.3 (m, 13H), 1.3 (d, J = 7.3 Hz, 2H), 1.2 (s, 2H), 1.2 (s, 6H), 1.15 (s, 6H), 1.08-1.01 (m, 3H), 10.96 (d, J = 6.2 Hz, 3H). **^{13}C NMR** (126 MHz, $CDCl_3$) δ 129.9 (CH), 129.6 (CH), 113.3 (CH), 79.3 (C), 75.0 (C), 60.4 (CH_2), 55.1 (CH_3), 54.6 (CH), 44.3 (CH), 41.5 (CH_2), 41.0 (CH), 36.9 (CH_2), 32.8 (CH_2), 29.7 (CH_2), 27.4 (CH_2), 27.0 (CH_2), 26.6 (CH_2), 25.8 (CH_3), 24.9 (CH_3), 24.8 (CH_3), 24.7 (CH_3), 24.6 (CH), 21.9 (CH_3).**^{11}B NMR** (160 MHz, $CDCl_3$) δ 10.0. **HRMS** (ESI, m/z): calculated for $C_{28}H_{45}BNO_3$[M^+ + H]: 454.3487; found: 454.3495.

6.2. Protonation of 16

HCl (2M in Et_2O) 0.4 mmol, 2 equiv) was added to a stirred solution of **16** (0.2 mmol) in Et_2O (2 mL) under argon atmosphere. The reaction was stirred for 16 h at rt. Then the volatiles were removed in vacuo to give the desired product as an amorphous white solid in 75% yield.

(4*S,6*S**)-6-((*R**)-1-cyclohexyl-2-(4,4,5,5-tetramethyl-1,3,2-dioxaborolan-2-yl)allyl)-4-methyl-1λ4-piperidin-2-ylium chloride (27)**

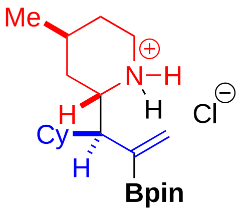

^{1}H-NMR (300 MHz, $CDCl_3$) δ 6.1 (d, J = 2.4 Hz, 1H), 5.82-5.75 (m, 1H), 3.9 (s, 1H), 3.5 (d, J = 6.9 Hz, 1H), 3.2 (m, 2H), 2.6 (m, 1H), 2.0 (s, 2H), 1.96-1.77 (m, 3H), 1.7 – 1.6 (m, 6H), 1.52-1.46 (m, 2H), 1.3 (s, 6H), 1.2 (s, 6H), 1.0 (d, J = 6.5 Hz, 3H), 0.93-0.72 (m, 2H). **^{13}C-NMR** (75 MHz, $CDCl_3$) δ 117.1 (CH_2), 79.4 (C), 55.4 (CH), 47.5 (CH), 41.6 (CH), 41.1 (CH_2), 35.4 (CH_2), 33.2 (CH_2), 32.0 (CH_2), 30.0 (CH_2), 27.4 (CH_2), 27.2 (CH_2), 26.8 (CH_2), 25.7 (2xCH_3), 25.2 (2xCH_3), 24.6 (CH), 22.0 (CH_3). **^{11}B-NMR** (160 MHz, $CDCl_3$) δ 32.0. **HRMS** (ESI, m/z): calculated for $C_{21}H_{39}BNO_2$ [M^+ + H- Cl]: 348.3068; found: 348.3066.

6.3. Derivatization of alkenyl boronate

- **Trifuloroborate salt formation**

Potassium ((*R)-3-cyclohexyl-3-((2*S**,4*S**)-4-methylpiperidin-2-yl)prop-1-en-2-yl)trifluoroborate (28)**

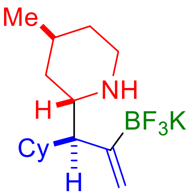

In a screw cap vial, product **16** (0.2 mmol, 1 equiv) was dissolved in 3 ml of a 2:1 MeOH/H_2O mixture. Then, KHF_2 (0.9 mmol, 4.5 equiv) was added and the reaction was stirred at room temperature for 3 h. After this time, volatiles were removed under reduce pressure and the resulting solid was washed with Et_2O (3x2 ml) to obtain the pure product as a white solid in 99% yield.

^{1}H-NMR (300 MHz, $CDCl_3$) δ 6.74 (s, 1H), 5.55 (d, *J* = 4.1 Hz, 1H), 5.14 (d, *J* = 4.1 Hz, 1H), 3.59 (d, *J* = 7.9 Hz, 1H), 3.54 – 3.41 (m, 1H), 3.12 – 3.02 (m, 1H), 2.16 – 2.03 (m, 2H), 2.01 – 1.82 (m, 2H), 1.78 – 1.42 (m, 8H), 1.40 – 1.13 (m, 3H), 1.06 (d, *J* = 7.0 Hz, 3H), 0.99 – 0.86 (m, 2H). **^{13}C-NMR, DEPT** (75 MHz, $CDCl_3$) δ 124.6 (CH_2), 55.8 (CH), 53.7 (CH), 40.6 (CH_2), 36.9 (CH), 33.9 (CH_2), 32.2 (CH_2), 30.8 (CH_2), 29.2 (CH_2), 26.6 (CH_2), 26.5 (CH_2), 26.5 (CH_2), 24.3 (CH), 18.7 (CH_3). **^{11}B NMR** (160 MHz, $CDCl_3$) δ 4.8 ppm. **^{19}F-NMR** (282 MHz, $CDCl_3$) δ -136.25 ppm. **HRMS** (APCI, m/z): calculated for $C_{15}H_{26}BF_3N$ [M^+ - K]: 288.2116; found: 288.2124.

- **Iodination**

(2*S,4*S**)-Ethyl 2-((*S**)-1-cyclohexyl-2-iodoallyl)piperidine-4-carboxylate (29)**

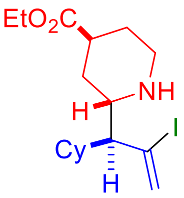

A screw cap vial was charged with product **17** (0.2 mmol, 1 equiv) and *N*-iodosuccinimide (0.3 mmol, 1.5 equiv), and the mixture was dissolved in dry THF (1 ml). The solution was left stirring for 2 h at room temperature. After this time, the reaction was quenched with H_2O (10 ml) and extracted with AcOEt (3x10 ml). The combination of organic phases was dried over Na_2SO_4 and concentrated in vacuo. The residue was purified by flash column chromatography (100% hexane to 100% AcOEt) obtaining the product as a brown solid in 75% yield.

^{1}H-NMR (500 MHz, $CDCl_3$) δ 6.24 (s, 1H), 6.01 (s, 1H), 4.26 – 4.09 (m, 2H), 3.03 (dt, *J* = 12.5, 3.7 Hz, 1H), 2.94 (ddd, *J* = 10.8, 8.2, 2.5 Hz, 1H), 2.80 (dt, *J* = 5.2, 2.4 Hz, 1H), 2.74 (td, *J* = 12.4, 2.9 Hz, 1H), 2.24 – 2.14 (m, 1H), 2.13 – 2.04 (m, 1H), 1.85 (t, *J* = 6.8 Hz, 1H), 1.79 – 1.52 (m, 9H), 1.56 – 1.34 (m, 1H), 1.27 (t, *J* = 7.1 Hz, 3H), 1.22 – 1.08 (m, 4H).**^{13}C-NMR, DEPT** (126 MHz, $CDCl_3$) δ 174.7 (C), 130.4 (CH_2), 111.4 (C), 60.6 (CH_2), 60.2 (CH), 54.3 (CH), 43.9 (CH_2), 38.4 (CH), 38.4 (CH), 31.9 (CH_2), 30.9 (CH_2), 28.5 (CH_2), 27.4 (CH_2), 27.0 (CH_2), 26.9 (CH_2), 26.6 (CH_2), 14.4 (CH_3). **HRMS** (APCI, m/z): calculated for $C_{17}H_{29}INO_2$ [M^+ + H]: 406.1238; found: 406.1246.

- **Suzuki coupling:**

A 5 mL sealed tube equipped with stirring magnetic bar was flamed-dried under vacuum, cooled to rt and backfilled with argon. Then it was charged with $Pd(PPh_3)_4$ (0.01 mmol, 0.05 equiv), K_2CO_3 (0.6 mmol, 3 equiv), $ArB(OH)_2$ (0.3 mmol, 1.5 equiv) and **29** (0.2 mmol, 1 equiv). Afterwards, it was put in vacuum and backfilled with argon for 3 times. Then 1.4 mL

of 1,4-dioxane:H_2O (6:1) were added and the reaction was heated to 100 °C for 16 h. The reaction was cooled to rt and quenched with a saturated aq. solution of NH_4Cl (20 mL) and extracted with AcOEt (2 x 20 mL). The combination of organic phases was dried over Na_2SO_4 and concentrated in vacuo. The residue was then purified using the indicated eluent to give the pure product.

(2*S,4*S**)-Ethyl 2-((*R**)-1-cyclohexyl-2-phenylallyl)piperidine-4-carboxylate (30)**

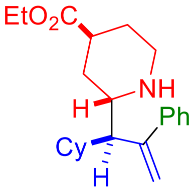

Yellow oil isolated in 76% yield after flash column chromatography on silica gel (Et_2O/MeOH, 95:5).
^{1}H-NMR (500 MHz, $CDCl_3$) δ 7.42 (d, *J* = 7.5 Hz, 2H), 7.30 (dt, *J* = 7.5 Hz, 2H), 7.23 (t, *J* = 7.5 Hz, 1H), 5.43 (s, 1H), 5.09 (s, 1H), 4.25 – 4.10 (m, 2H), 3.02 – 2.91 (m, 2H), 2.81 – 2.74 (m, 1H), 2.72 (td, *J* = 12.4, 2.7 Hz, 1H), 2.55 (t, *J* = 7.2 Hz, 1H), 2.17 (dq, *J* = 13.3, 2.4 Hz, 1H), 2.07 (dp, *J* = 13.5, 2.5 Hz, 1H), 1.84 – 1.66 (m, 4H), 1.66 – 1.53 (m, 2H), 1.42 – 1.34 (m, 2H), 1.27 (t, *J* = 7.1 Hz, 3H), 1.19 (tt, *J* = 12.5, 3.3 Hz, 2H), 1.15 – 1.00 (m, 2H), 0.89 (qd, *J* = 12.5, 3.4 Hz, 1H).**^{13}C-NMR, DEPT** (126 MHz, $CDCl_3$) δ 175.1 (C), 149.0 (C), 145.6 (C), 128.3 (CH), 127.2 (CH), 126.8 (CH), 114.8 (CH_2), 60.5 (CH_2), 54.4 (CH), 54.1 (CH), 44.1 (CH_2), 39.2 (CH), 38.5 (CH), 32.4 (CH_2), 32.1 (CH_2), 28.2 (CH_2), 27.7 (CH_2), 27.1 (CH_2), 26.8 (CH_2), 26.7 (CH_2), 14.4 (CH_3). **HRMS** (APCI, m/z): calculated for $C_{23}H_{34}NO_2$ [M^+ + H]: 356.2584; found: 356.2573.

(2*S,4*S**)-Ethyl 2-((*R**)-1-cyclohexyl-2-(3,5-difluorophenyl)allyl)piperidine-4-carboxylate (31)**

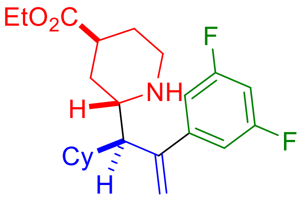

Yellow oil isolated in 65% yield after flash column chromatography on silica gel (DCM/MeOH, 90:10).
^{1}H-NMR (500 MHz, $CDCl_3$) δ 7.03 – 6.89 (m, 2H), 6.67 (tt, *J* = 8.8, 2.3 Hz, 1H), 5.50 (s, 1H), 5.16 (s, 1H), 4.29 – 4.07 (m, 2H), 3.06 – 2.94 (m, 2H), 2.83 – 2.64 (m, 2H), 2.45 (t, *J* = 7.3 Hz, 1H), 2.21 – 2.01 (m, 2H), 1.83 – 1.55 (m, 7H), 1.40 (m, 2H), 1.27 (t, *J* = 7.1 Hz, 3H), 1.21 – 0.96 (m, 4H), 0.92 – 0.75 (m, 1H). **^{13}C-NMR, DEPT** (126 MHz, $CDCl_3$) δ 174.9 (C), 163.0 (dd, *J* = 247.6, 13.2 Hz) (C), 149.0 (C), 147.2 (C), 116.6 (CH_2), 109.7 (dd, *J* = 19.7, 6.5 Hz) (CH), 102.5 (t, *J* = 25.5 Hz) (CH), 60.6 (CH_2), 54.2 (CH), 54.0 (CH), 44.0 (CH_2), 39.1 (CH), 38.3 (CH), 32.4 (CH_2), 31.8 (CH_2), 28.1 (CH_2), 27.4 (CH_2), 26.9 (CH_2), 26.7 (CH_2), 26.6 (CH_2), 14.4 (CH_3). **^{19}F-NMR** (282 MHz, $CDCl_3$) δ -110.19 (t, *J* = 9.0 Hz). **HRMS** (APCI, m/z): calculated for $C_{23}H_{32}F_2NO_2$ [M^+ + H]: 392.2396 found: 392.2404.

- **"Chan-Lam" acetoxylation/intramolecular acetyl transfer**

(2*S,4*S**)-Ethyl 1-acetyl-2-((*S**)-1-cyclohexyl-2-oxopropyl)piperidine-4-carboxylate (32)**

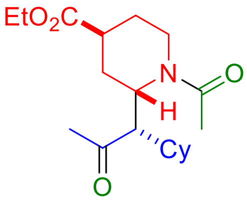

A solution of product **17** (0.1 mmol, 1 equiv), $Cu(OAc)_2$ (0.1 mmol, 1 equiv), Et_3N (0.2 mmol, 2 equiv), and powdered activated 4 Å molecular sieves in MeCN (1 mL) was sealed in an oven-dried screw cap vial under air and stirred at 80 °C for 20 h. The reaction mixture was allowed to cool to room temperature and filtered through Celite (eluent Et_2O), and the filtrate was evaporated to give a residue that was purified by flash column chromatography with

deactivated silica (MeOH/Et_2O 2:98) obtaining the product as a brown oil in 72% yield as a 1.4:1 mixture of rotamers (major rotamer – red, minor rotamer – green, mixture of both purple). **^{1}H-NMR** (500 MHz, $CDCl_3$) δ 5.21 (dd, J = 13.9, 3.7 Hz, 1H), 4.59 (dd, J = 13.9, 4.0 Hz, 1H), 4.23 (dd, J = 11.6, 4.9 Hz, 1H), 4.07 – 3.97 (m, 4H), 3.47 (d, J = 14.4 Hz, 1H), 3.17 (dd, J = 11.4, 4.1 Hz, 1H), 3.03 (t, J = 13.4 Hz, 1H), 2.62 (dd, J = 11.7, 5.9 Hz, 1H), 2.57 – 2.40 (m, 3H), 1.98 (s, 3H), 1.97 (s, 3H), 1.91 (s, 6H), 1.96 – 1.82 (m, 4H), 1.69 – 1.59 (m, 8H), 1.53 (td, J = 13.9, 5.4 Hz, 6H), 1.44 – 1.27 (m, 4H), 1.18 – 1.12 (m, 8H), 1.08 – 0.81 (m, 6H). **^{13}C-NMR, DEPT** (126 MHz, $CDCl_3$) δ 210.2 (C), 210.0 (C), 174.3 (C), 170.2 (C), 169.4 (C), 60.9 (CH_2), 58.4 (CH), 54.1 (CH), 52.6 (CH), 46.6 (CH), 41.2 (CH_2), 38.4 (CH), 38.2 (CH), 36.8 (CH), 36.5 (CH), 35.9 (CH_2), 34.8 (CH_i), 32.8 (CH_2), 32.2 (CH_2), 29.2 (CH_2), 29.1 (CH_2), 28.9 (CH_3), 28.8 (CH_2), 28.5 (CH_2), 28.2 (CH_2), 27.1 (CH_2), 26.8 (CH_2), 26.7 (CH_2), 26.5 (CH_2), 26.4 (CH_2), 26.3 (CH_2), 26.2 (CH_2), 21.8 (CH_3), 21.3 (CH_3), 14.3 (CH_3). **HRMS** (APCI, m/z): calculated for $C_{19}H_{32}BNO_4$ [M^+ + H]: 338.2326 found 338.2320.

- **Copper-catalyzed protoboration**

(2*S,4*S**)-Ethyl 2-((1*S**,2*R**)-1-cyclohexyl-2,3-bis(4,4,5,5-tetramethyl-1,3,2-dioxaborolan-2-yl)propyl)piperidine-4-carboxylate (33)**

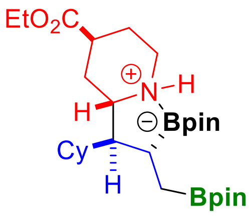

In an oven-dried screw cap vial, a solution of CuCl (0.01 mmol, 0.1 equiv), ICyCl (0.01 mmol, 0.1 equiv) and NaO^{t}Bu (0.02 mmol, 0.2 equiv) in THF (0.3 ml) was stirred for 30 min to form the catalyst. An oven-dried Schlenk tube was charged with NaO^{t}Bu (0.12 mmol, 1.2 equiv), $B_2(pin)_2$ (0.15 mmol, 1.5 equiv) and product **17** (0.1 mmol, 1 equiv) and dissolved in dry THF (0.7 ml). The preformed ICyCuCl was added to the Schlenk tube, and the mixture was left stirring overnight at room temperature. After this time, the residue was filtrated through a plug of celite using Et_2O as eluent. The resulting crude was purified by flash column chromatography using deactivated silica (hexane/AcOEt, 8:2) obtaining the product as a white solid in 57% yield (7:1 mixture of diastereomers). Spectroscopic data collected only for major isomer.

^{1}H-NMR (500 MHz, $CDCl_3$**)** δ 4.23 – 4.07 (m, 2H), 3.08 (d, J = 15.0 Hz, 1H), 2.79 – 2.66 (m, 2H), 2.65 – 2.40 (m, 2H), 2.38 – 2.22 (m, 1H), 1.79 – 1.67 (m, 5H), 1.62 (d, J = 10.4 Hz, 2H), 1.45 – 1.31 (m, 2H), 1.25 (m, J = 7.3 Hz, 12H), 1.22 – 1.11 (m, 7H), 1.06 (m, J = 4.4 Hz, 12H), 1.03 – 0.90 (m, 3H), 0.64 – 0.52 (m, 2H).**^{13}C-NMR, DEPT** (126 MHz, $CDCl_3$) δ 173.5 (C), 82.4 (C), 78.8 (C), 61.0 (CH_2), 57.6 (CH), 42.7 (CH), 40.5 (CH_2), 37.6 (CH), 31.8 (CH_2), 30.8 (CH_2), 29.8 (CH_2), 27.0 (CH_2), 26.9 (CH_2), 26.7 (CH), 26.3 (CH_3), 25.4 (CH_3), 25.1 (CH_3), 14.2 (CH_3).**^{11}B-NMR** (160 MHz, $CDCl_3$) δ 37.0, 12.4 ppm. **HRMS** (APCI, m/z): calculated for $C_{29}H_{54}B_2NO_6$ [M^+ + H]: 534.4132 found 534.4149.

6.4. Conversion of 16 into tosyl amide 54 for er determination (see Table S6)

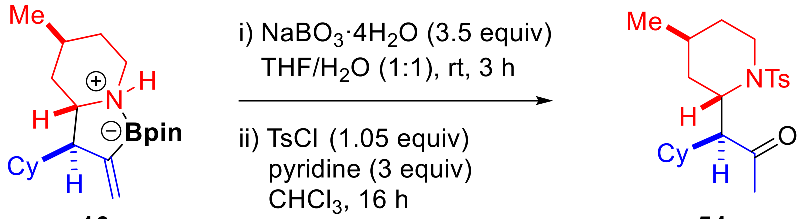

16 **54**

Crude product **16** was dissolved in 6 mL of THF/H_2O (1:1) and $NaBO_3{\cdot}4H_2O$ (1.05 mmol, 3.5 equiv) was added. The mixture was vigorously stirred at room temperature for 3 h. Then an aqueous saturated solution of NH_4Cl was added and the aqueous layer was extracted three times with Et_2O. The combined organic extracts were dried with anhydrous Na_2SO_4, filtered and solvents were evaporated under vacuum. The resulting α-aminoketone was purified by flash column chromatography on silica gel using a 15% MeOH (Et_2O) as eluent.

The pure α-aminoketone was dissolved in $CHCl_3$ (0.2 M) and tosyl chloride (1.05 equiv) and pyridine (3 equiv) were added. The mixture was stirred for 16 h at rt. Then CH_2Cl_2 (10 mL) was added, and the organic phase was washed with a saturated aqueous solution of $CuSO_4$ (2x10mL), aq. HCl 5% (2x10 mL) and brine. The organic layer was dried with anhydrous Na_2SO_4, filtered and solvents were evaporated under vacuum. The resulting tosyl amide **54** was purified by flash column chromatography on silica gel using a mixture of AcOEt/Hexanes (1:9) as eluent.

1-Cyclohexyl-1-(4-methyl-1-tosylpiperidin-2-yl)propan-2-one (54)

^{1}H NMR (500 MHz, $CDCl_3$) δ 7.7 (d, *J* = 8.0 Hz, 2H), 7.4 – 7.2 (m, 2H), 4.6 (dd, *J* = 11.5, 5.0 Hz, 1H), 3.7 (dd, *J* = 15.0, 4.6 Hz, 1H), 3.0 (ddd, *J* = 15.7, 13.4, 3.0 Hz, 1H), 3.0 (dd, *J* = 11.4, 5.1 Hz, 1H), 2.4 (s, 3H), 2.1 (s, 3H), 1.9 – 1.5 (m, 6H), 1.3 – 1.2 (m, 6H), 1.1 (qd, *J* = 9.9, 5.1 Hz, 2H), 1.1 – 0.8 (m, 2H), 0.8 (d, *J* = 6.4 Hz, 3H). **^{13}C NMR, DEPT** (126 MHz, $CDCl_3$) δ 210.2 (C), 143.3 (C), 138.3 (C), 129.7 (CH), 127.6 (CH), 56.7 (CH), 53.1 (CH), 41.3 (CH_2), 38.5 (CH), 34.0 (CH_2), 32.4 (CH_2), 32.1 (CH_2), 32.1 (CH), 27.7 (CH_2), 27.1 (CH_2), 26.7 (CH_2), 26.4 (CH_2), 25.0 (CH_3), 22.5 (CH_3), 21.7 (CH_3). **HRMS** (APCI, m/z): calculated for $C_{22}H_{34}NO_3S$ [M^+ + H]: 392.2254; found: 392.2265. **Optical rotation:** $[\alpha]_D^{22}$ -6.73 (*c*=0.275, $CHCl_3$).

HPLC conditions: 10% 2-propanol (Hexanes), Lux® 3µm i-Amylose-3, 1mL/min. t_R (min) = 23.5, 25.4.

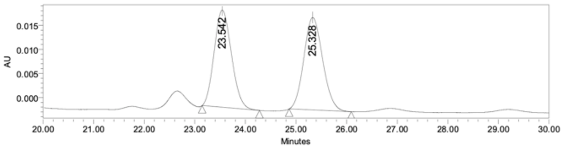

Peak Results

	Name	RT	Area	% Area
1		23.542	478579	49.02
2		25.328	497788	50.98

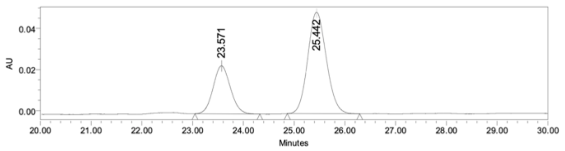

Peak Results

	Name	RT	Area	% Area
1		23.571	553913	30.53
2		25.442	1260528	69.47

7. Reaction with asymmetric *O*-benzoyl hydroxylamine 55

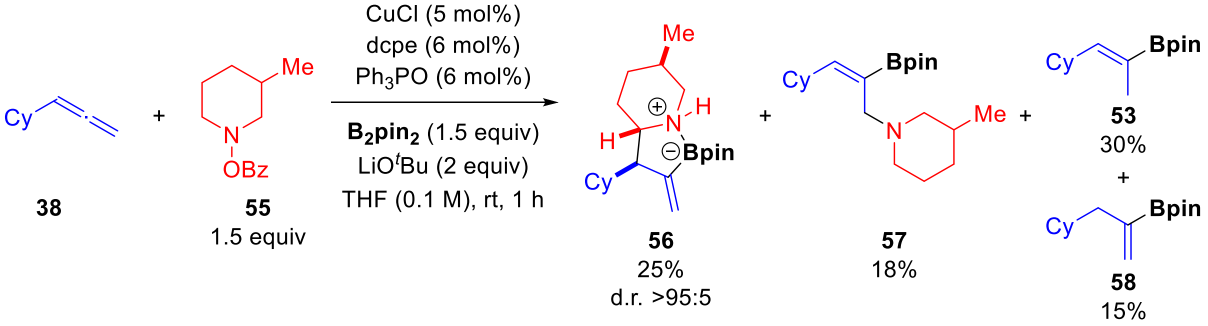

Scheme S1. Yield values refer to NMR yield

Product **56** was obtained with total regio- and diastereoselectivity, albeit with a decrease of chemoselectivity. Despite the high level of regioselectivity, formation of regioisomeric imines cannot be discarded. Formation of an inactive imine might result in the formation of ^{t}BuOH which is not consumed in the catalytic cycle thus accumulating and being available for the protonation of the allyl-copper intermediate. This would explain the high yield obtained for protoboration products **53** and **58**. In case two different imines are formed, only the less hindered one seems to undergo the borylative coupling. This is supported by the fact that reaction does not work with a disubstituted hydroxylamine (Scheme S2).

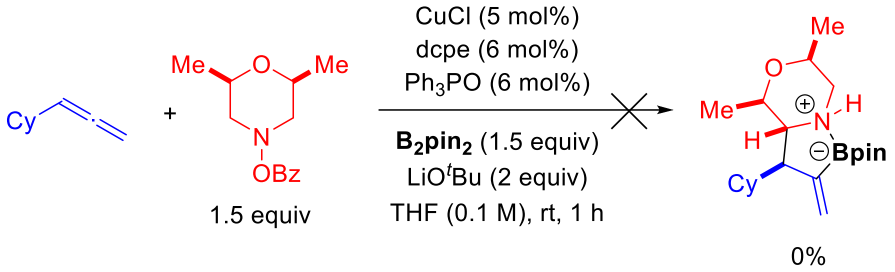

Scheme S2

(3*R,3a*S**,6*R**)-3-Cyclohexyl-4',4',5',5',6-pentamethyl-2-methyleneoctahydro-1λ⁴,8λ⁴-spiro[[1,2]azaborolo[1,5-*a*]pyridine-1,2'-[1,3,2]dioxaborolane] (56)**

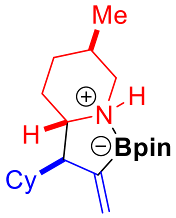

Synthesized from cyclohexylallene **38** and 3-methylpiperidin-1-yl benzoate **55** following general procedure A at rt (1 h). Colourless oil obtained in 15% yield after flash column chromatography on silica gel (MeOH/Et_2O 1.5:98.5).

^{1}H NMR (500 MHz, $CDCl_3$) δ 5.40 (t, J = 2.6 Hz, 1H), 5.27 (t, J = 2.5 Hz, 1H), 3.37 – 3.27 (m, 1H), 2.88 – 2.72 (m, 2H), 2.18 – 2.06 (m, 2H), 1.79 – 1.64 (m, 10H), 1.53 - 1.44 (m, 1H), 1.40 – 1.31 (m, 1H), 1.28 – 1.22 (m, 4H), 1.15 (s, 6H), 1.11 (s, 6H), 0.87 (d, J = 6.6 Hz, 3H). **^{13}C NMR** (126 MHz, $CDCl_3$) δ 117.2 (CH_2), 79.5 (C), 54.6 (CH), 47.7 (CH_2), 46.9 (CH), 41.8 (CH), 32.0 (CH_2), 30.7 (CH), 30.1 (CH_2), 27.4 (CH_2), 27.2 (CH_2), 27.1 (CH_2), 26.9 (CH_2), 26.6 (CH_2), 25.8 (2xCH_3), 25.3 (2xCH_3), 19.4 (CH_3). **^{11}B-NMR** (160 MHz, $CDCl_3$) δ 10.4. **HRMS** (ESI, m/z): calculated for $C_{21}H_{39}BNO_2$ [M^+ + H]: 348.3068; found: 348.3072.

8. NMR spectra

^{1}H-NMR, 300 MHz, $CDCl_3$

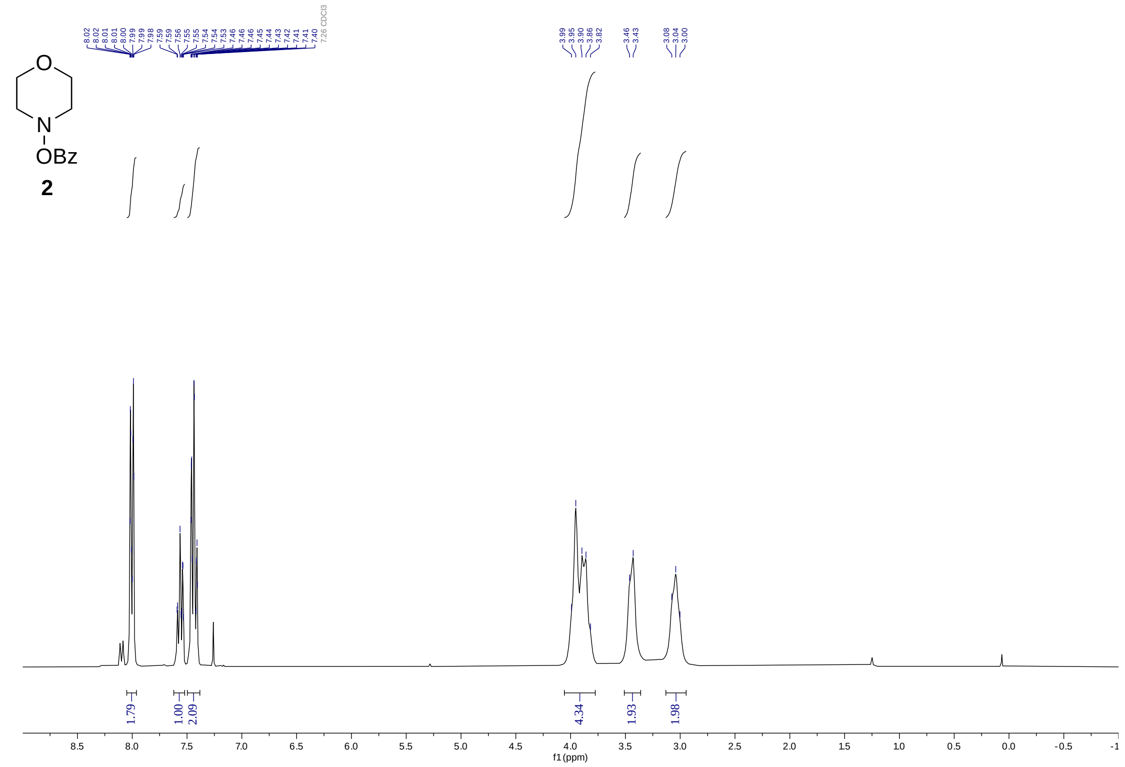

^{13}C-NMR, 75 MHz, $CDCl_3$

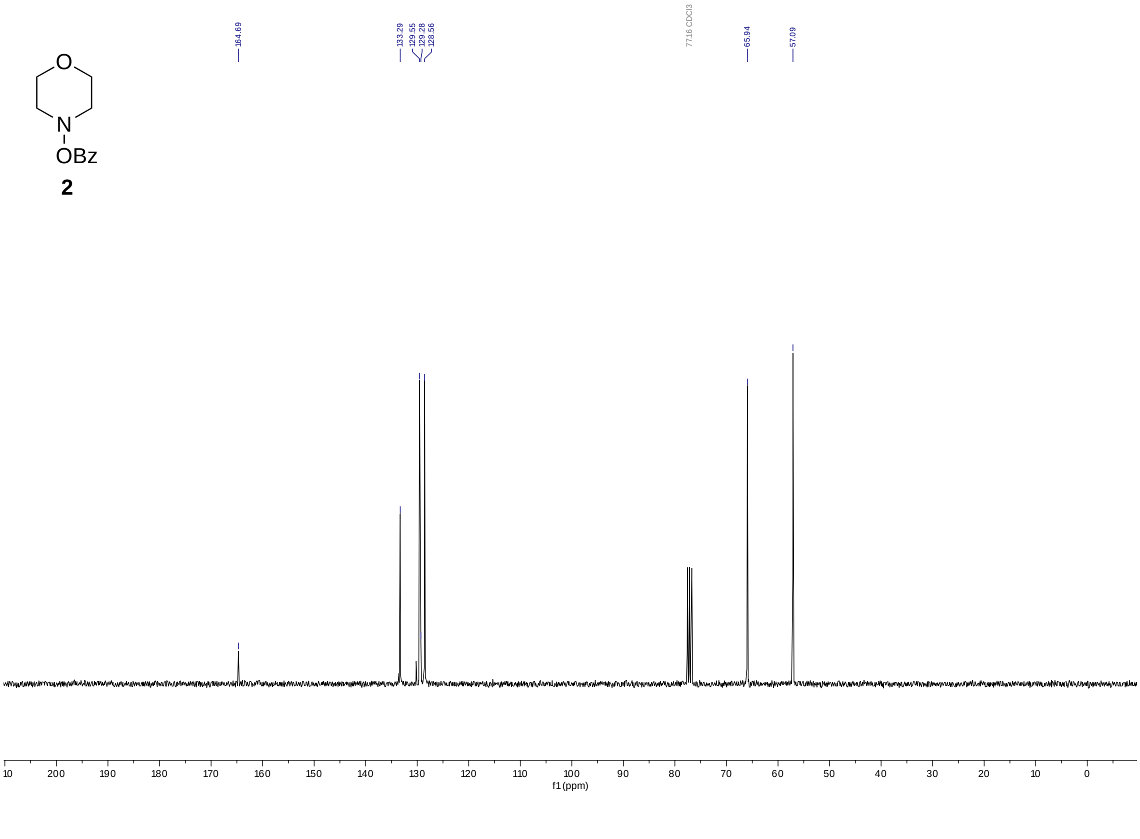

^{1}H-NMR, 300 MHz, $CDCl_3$

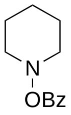

42

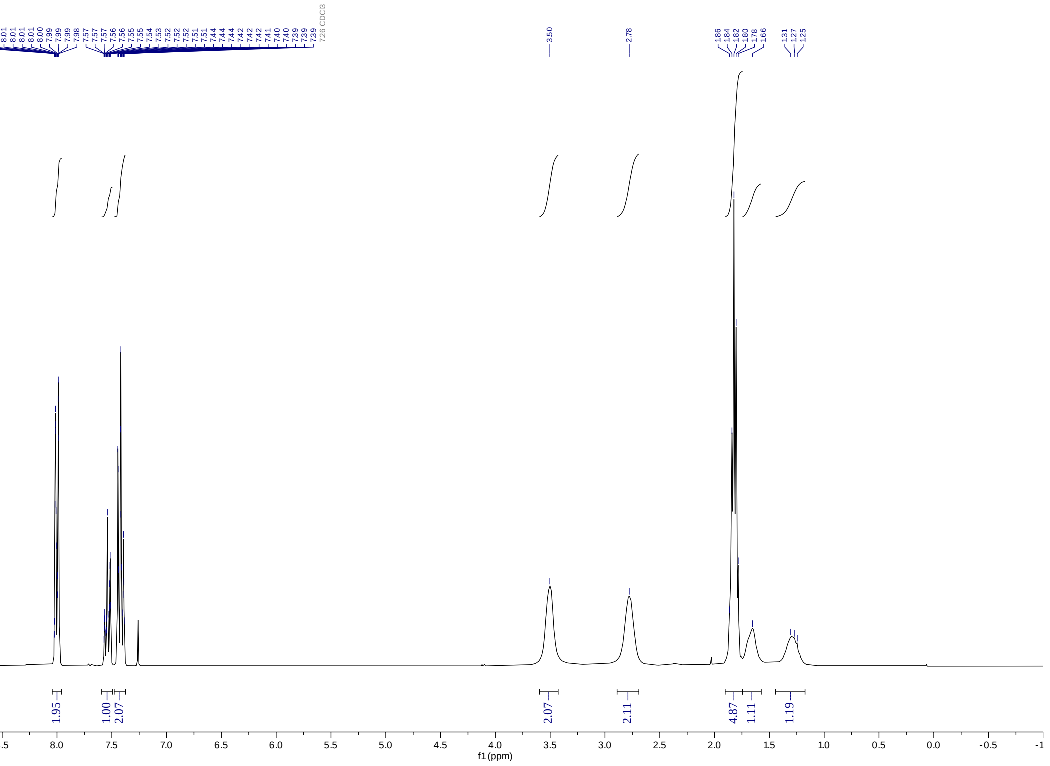

^{13}C-NMR, 75 MHz, $CDCl_3$

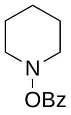

42

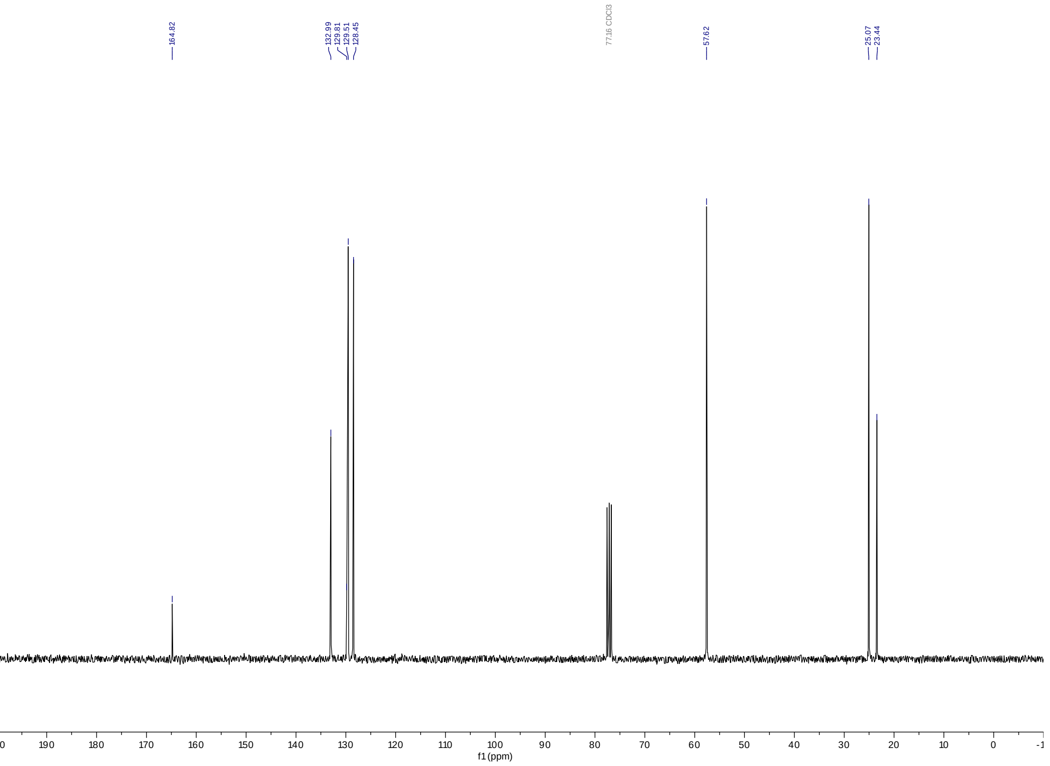

^{1}H-NMR, 300 MHz, $CDCl_3$

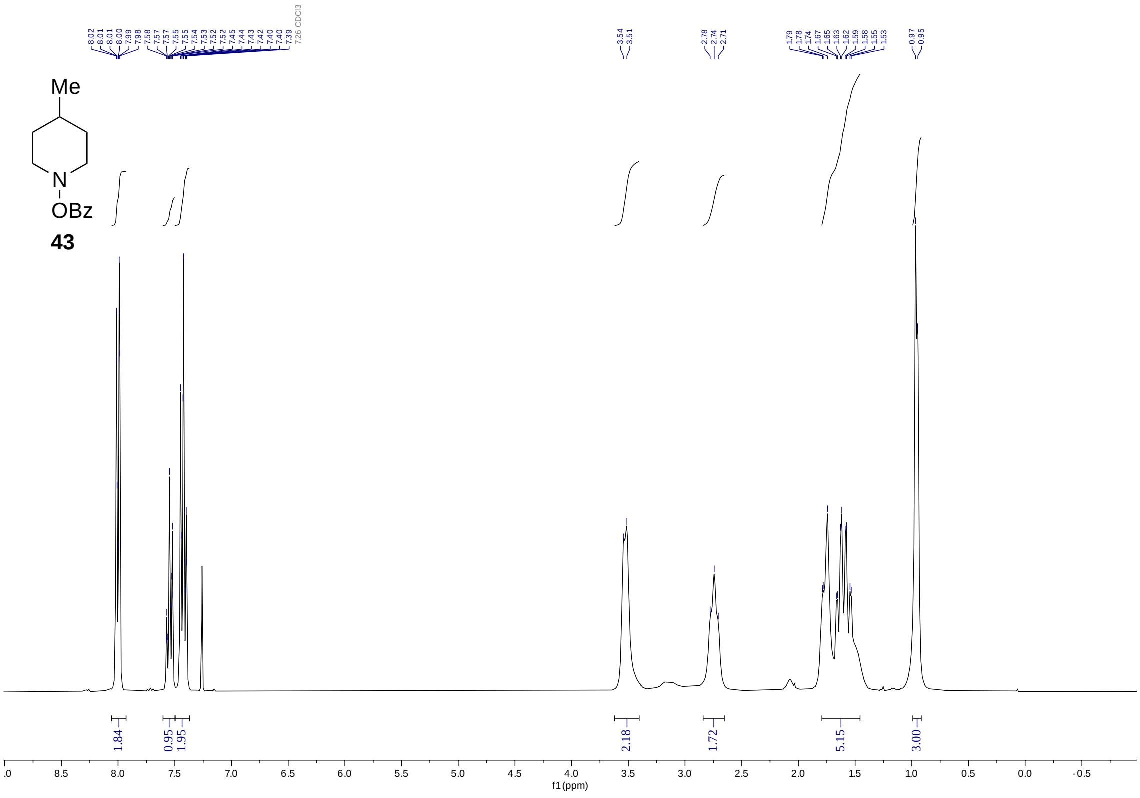

^{13}C-NMR, 75 MHz, $CDCl_3$

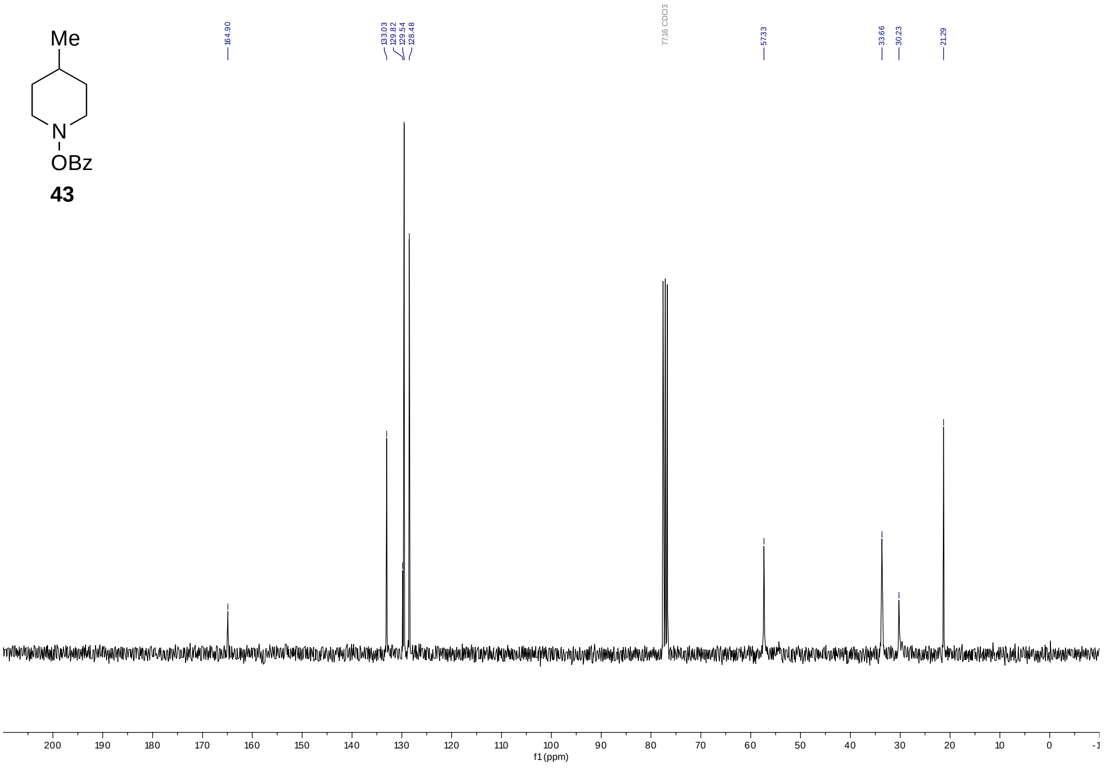

^{1}H-NMR, 300 MHz, $CDCl_3$

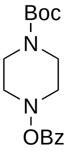

44

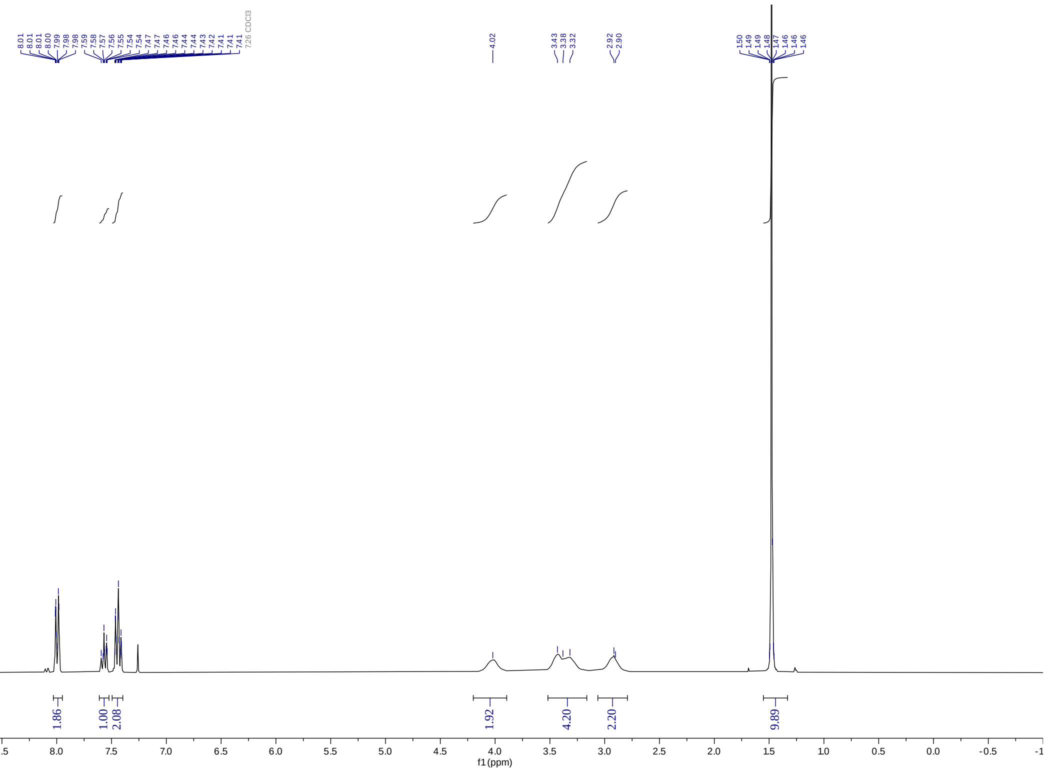

12. ^{13}C-NMR, 75 MHz, $CDCl_3$

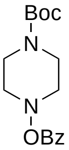

44

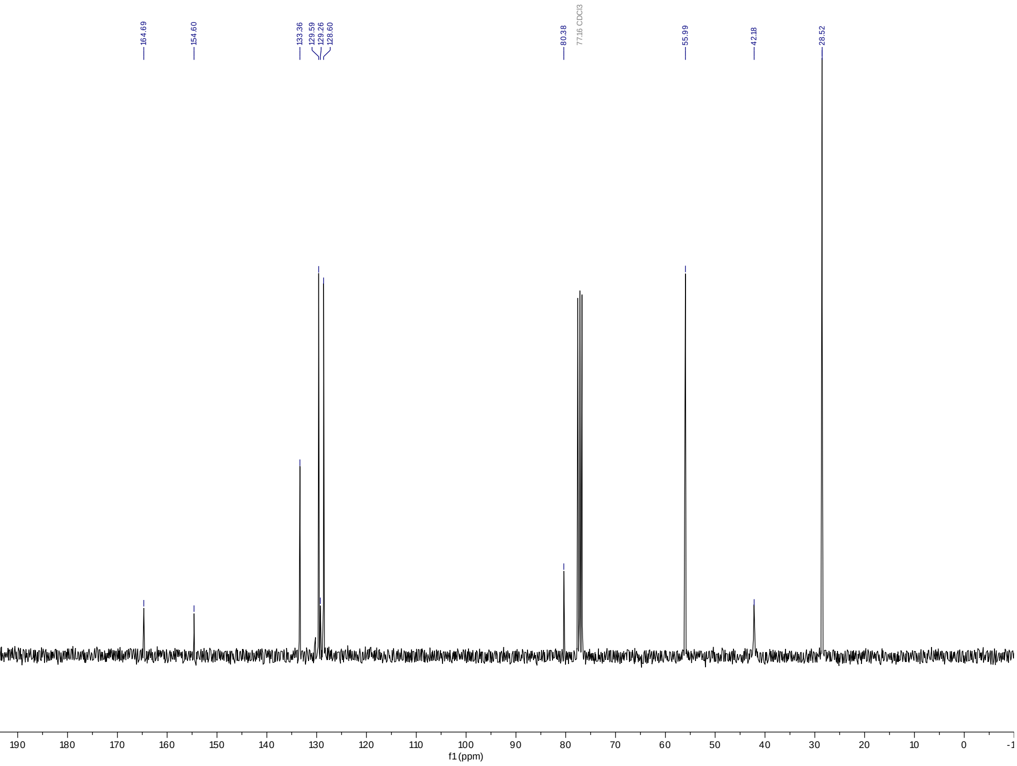

^{1}H-NMR, 300 MHz, $CDCl_3$

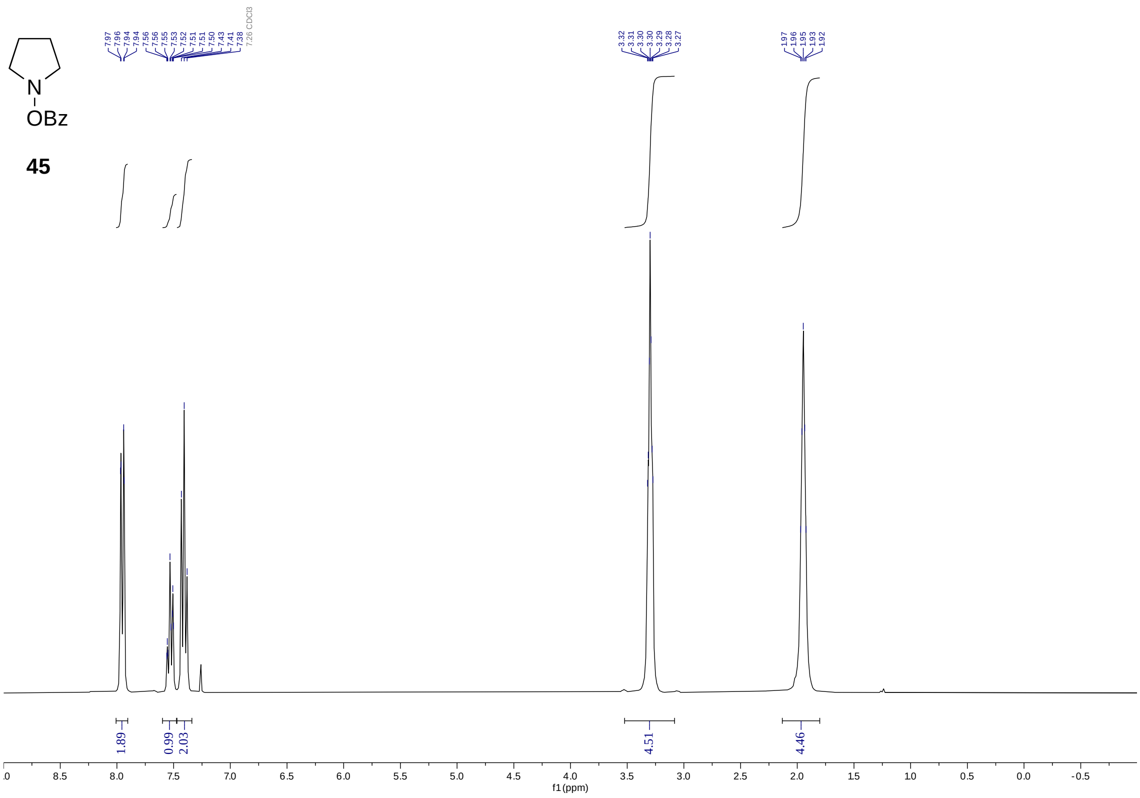

^{13}C-NMR, 75 MHz, $CDCl_3$

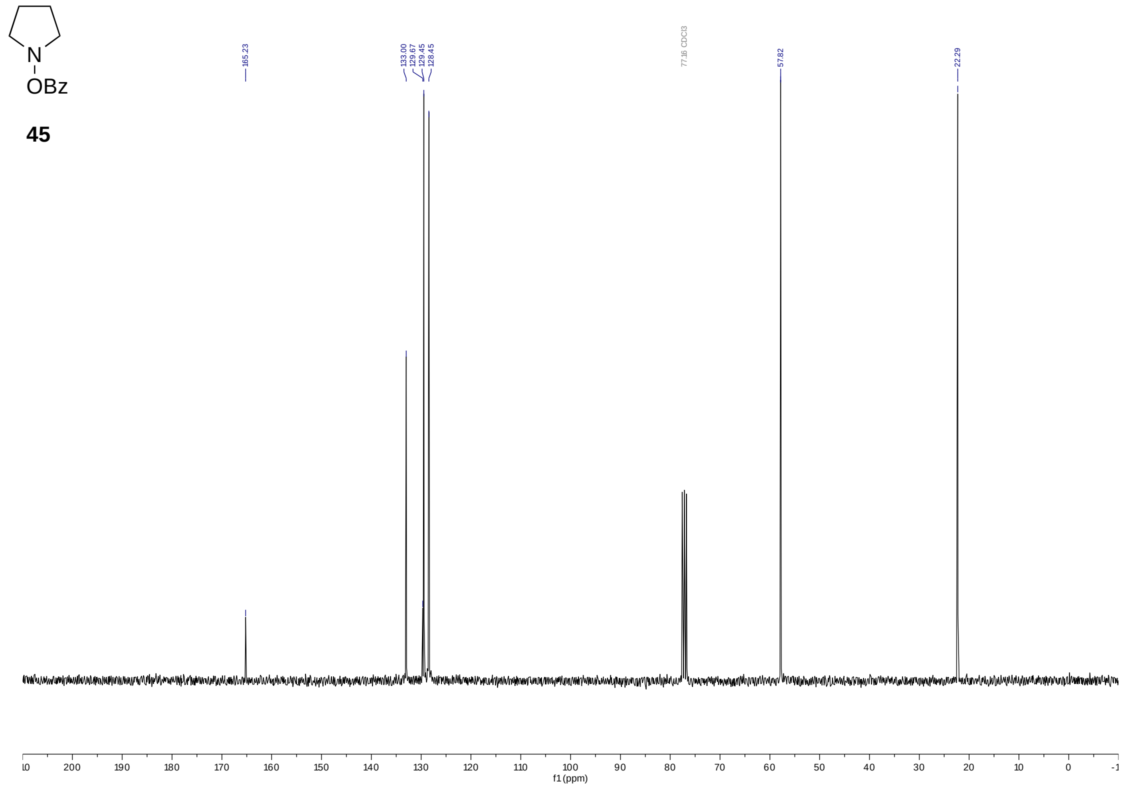

^{1}H-NMR, 300 MHz, $CDCl_3$

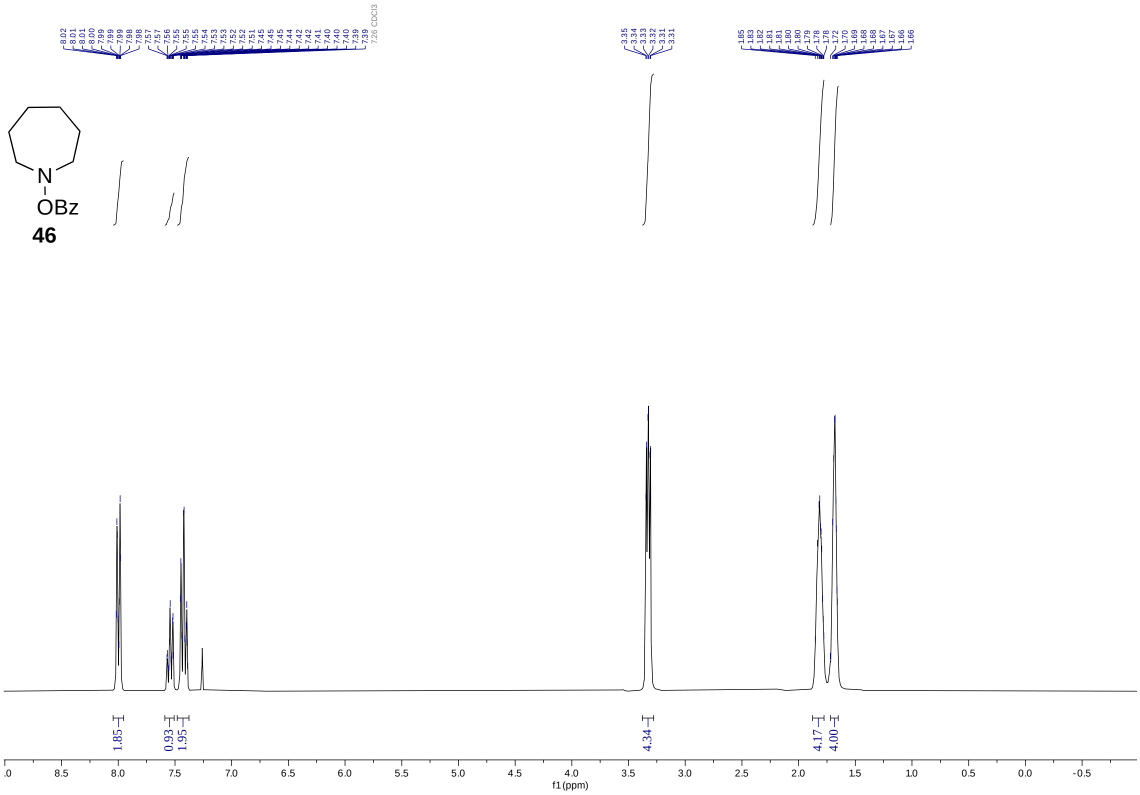

^{13}C-NMR, 75 MHz, $CDCl_3$

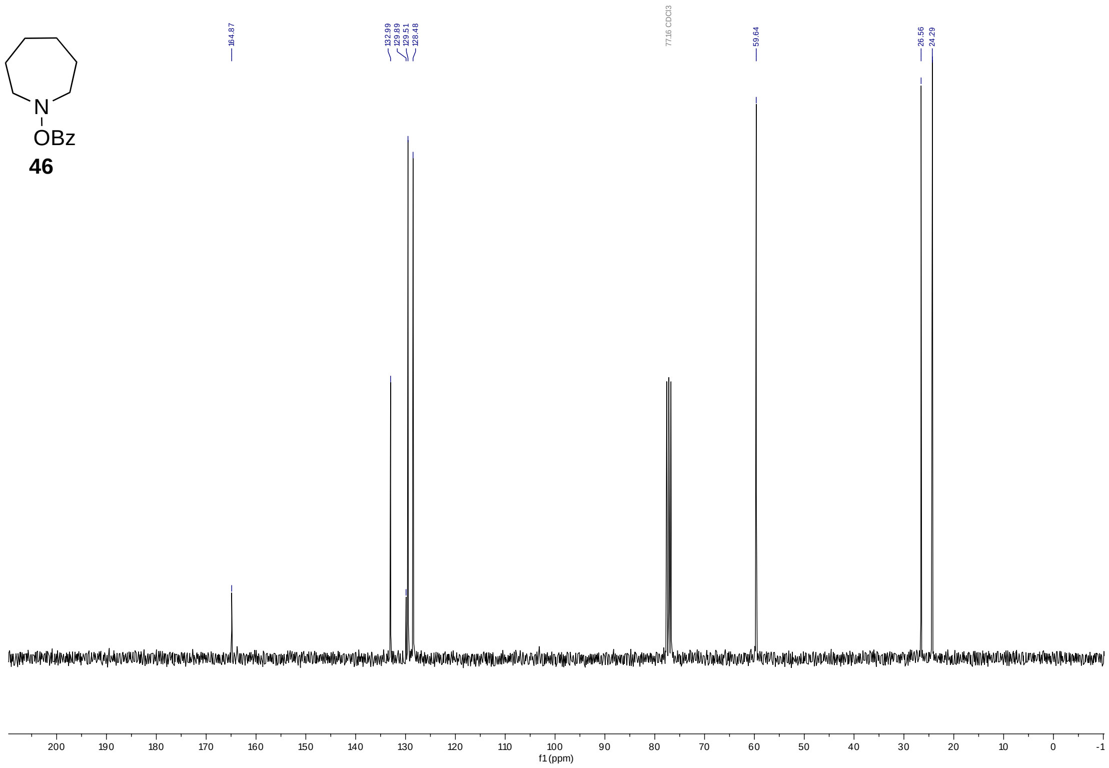

^{1}H-NMR, 300 MHz, $CDCl_3$

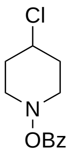

47

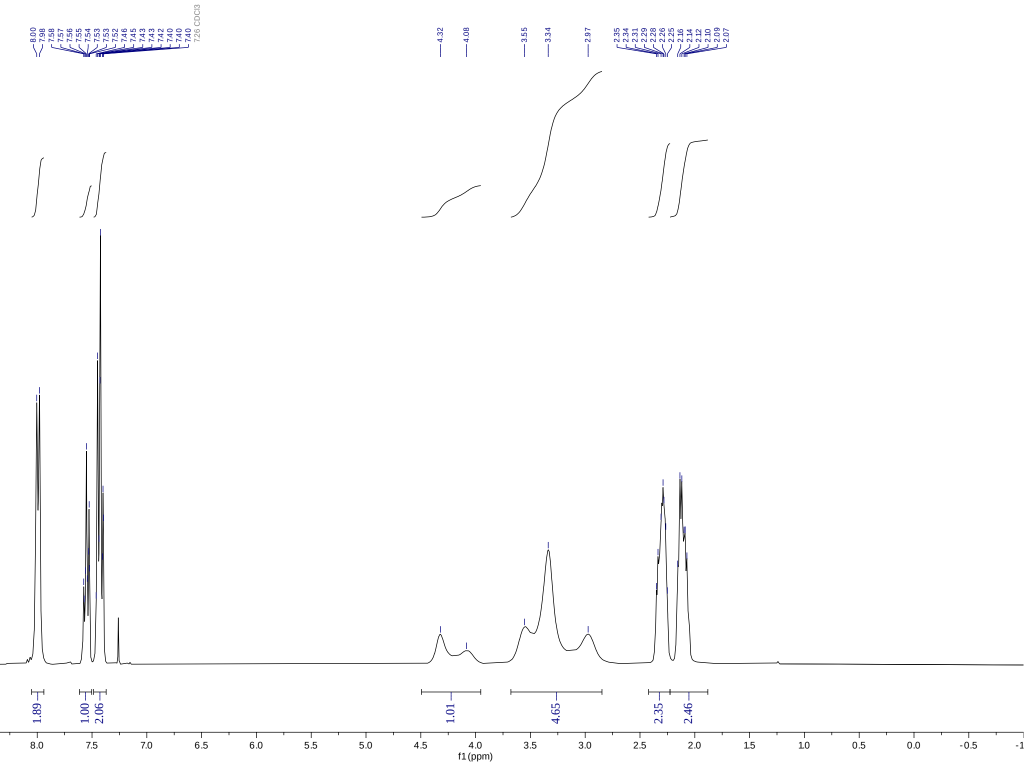

^{13}C-NMR, 75 MHz, $CDCl_3$

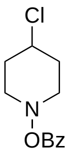

47

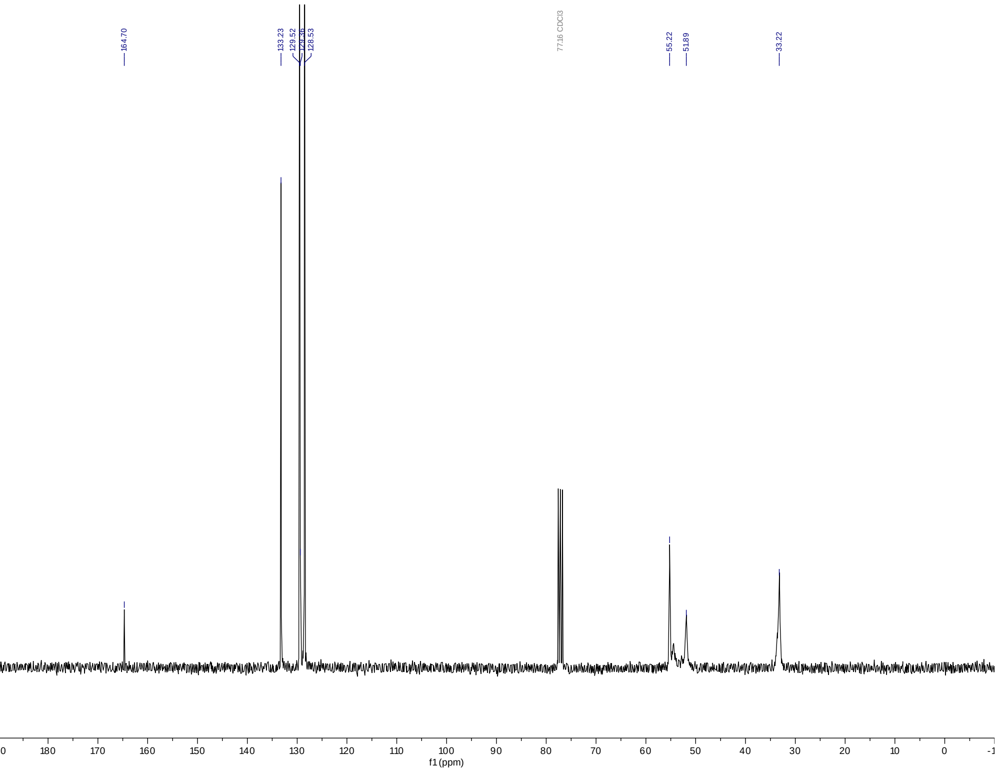

^{1}H-NMR, 300 MHz, $CDCl_3$

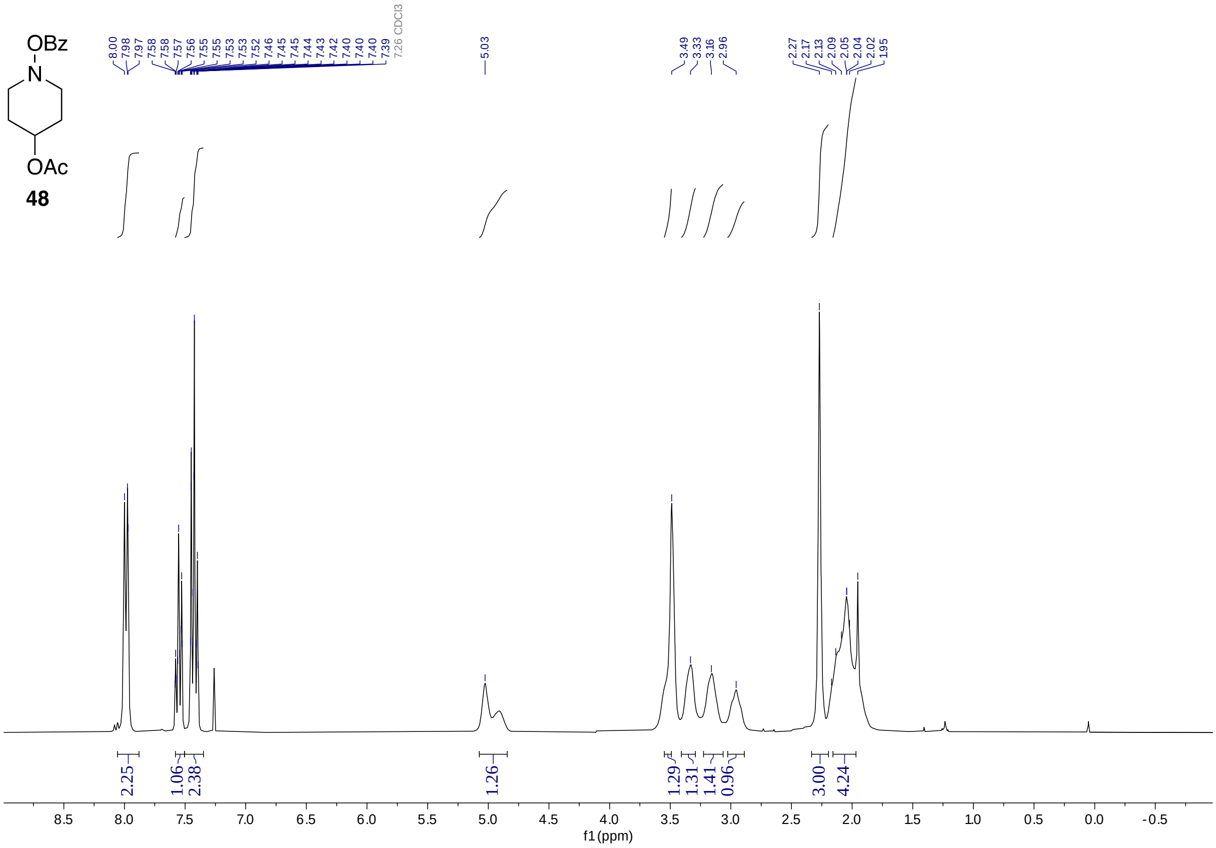

Note: The peak at 3.50 ppm belongs to MeOH.

^{13}C-NMR, 75 MHz, $CDCl_3$

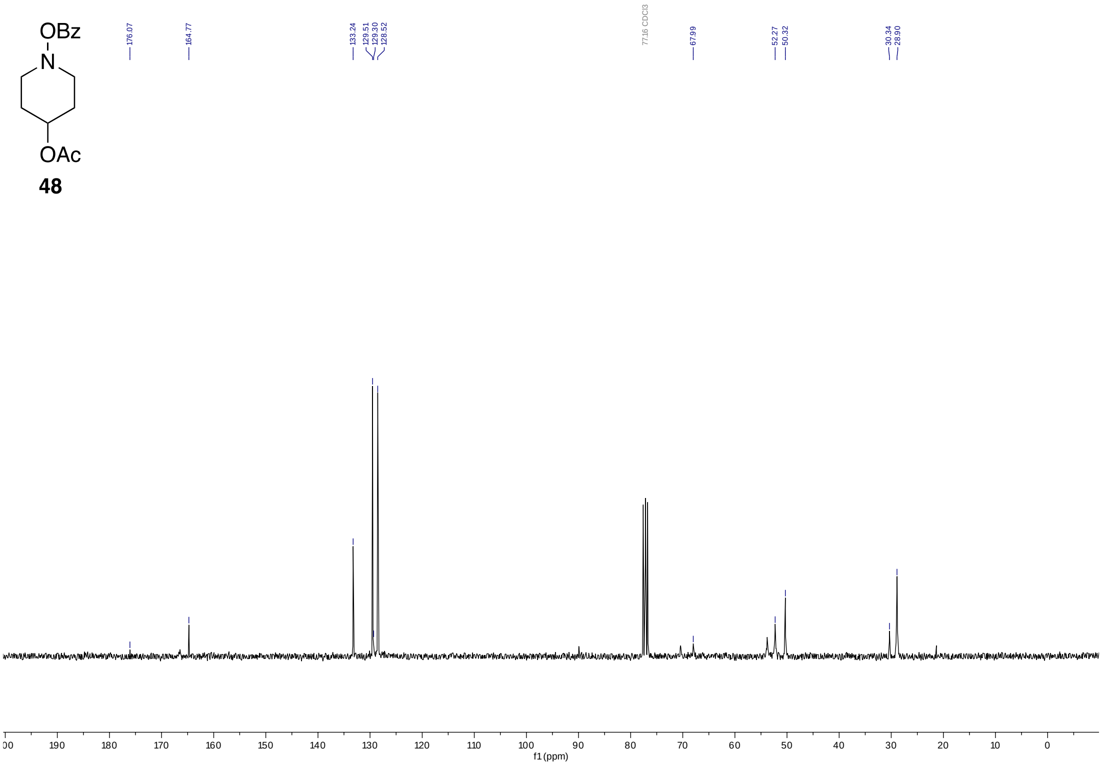

^{1}H-NMR, 300 MHz, $CDCl_3$

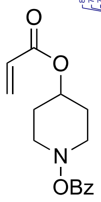

49

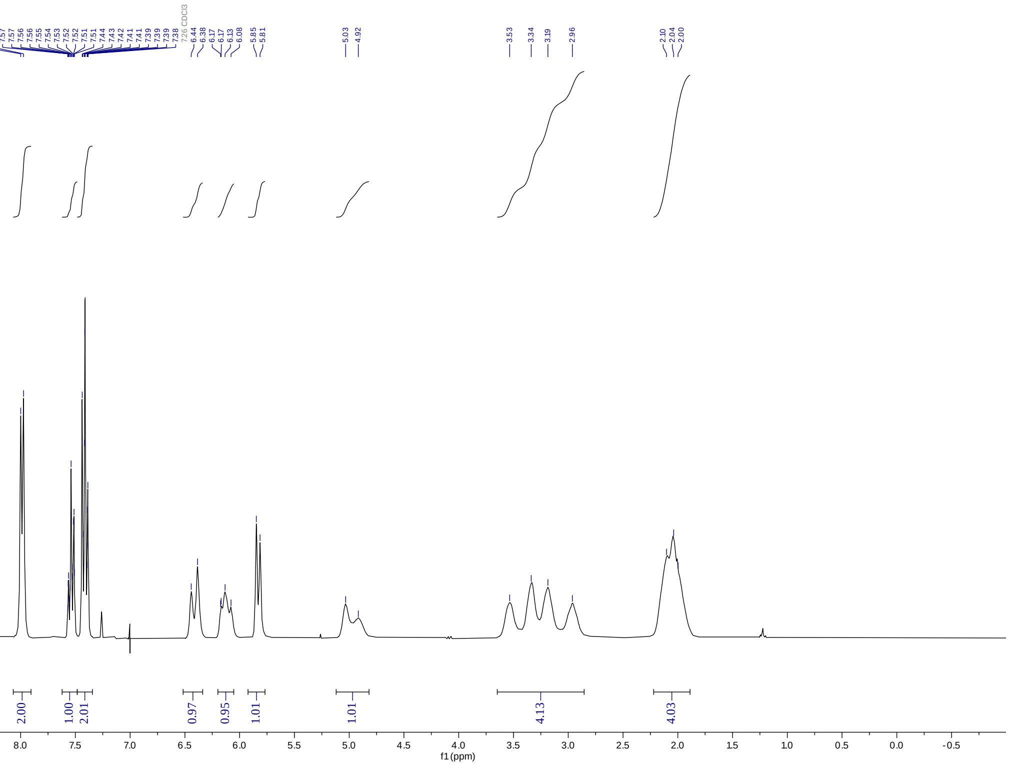

^{13}C-NMR, 75 MHz, $CDCl_3$

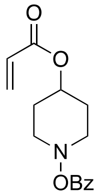

49

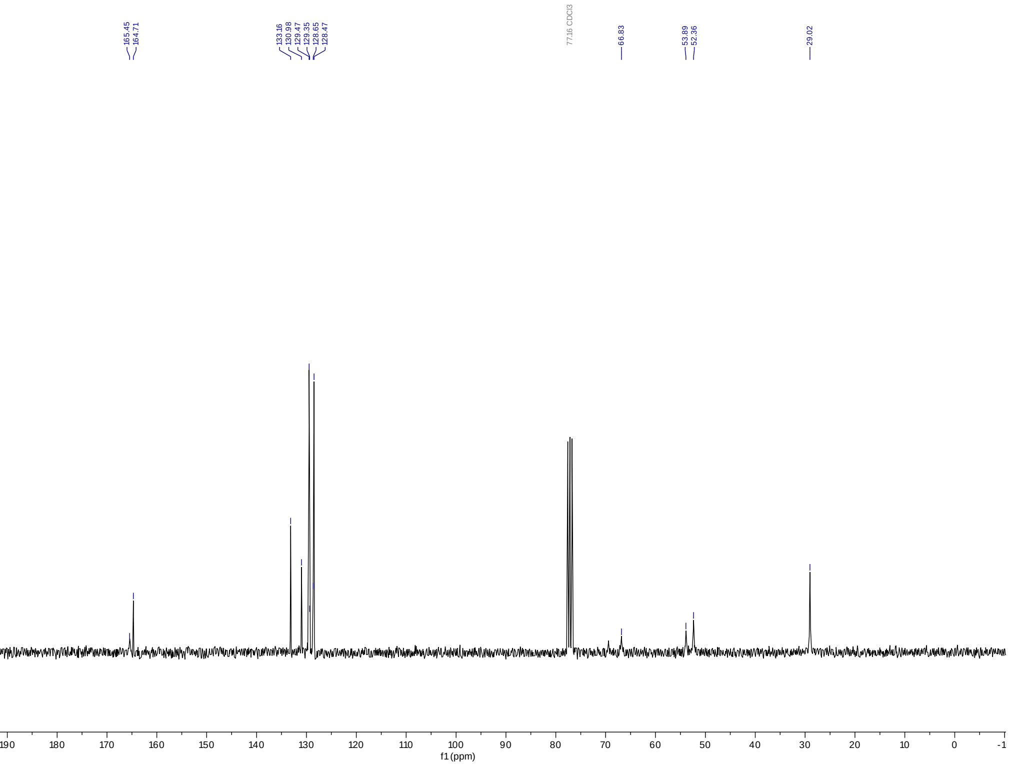

^{1}H-NMR, 300 MHz, $CDCl_3$

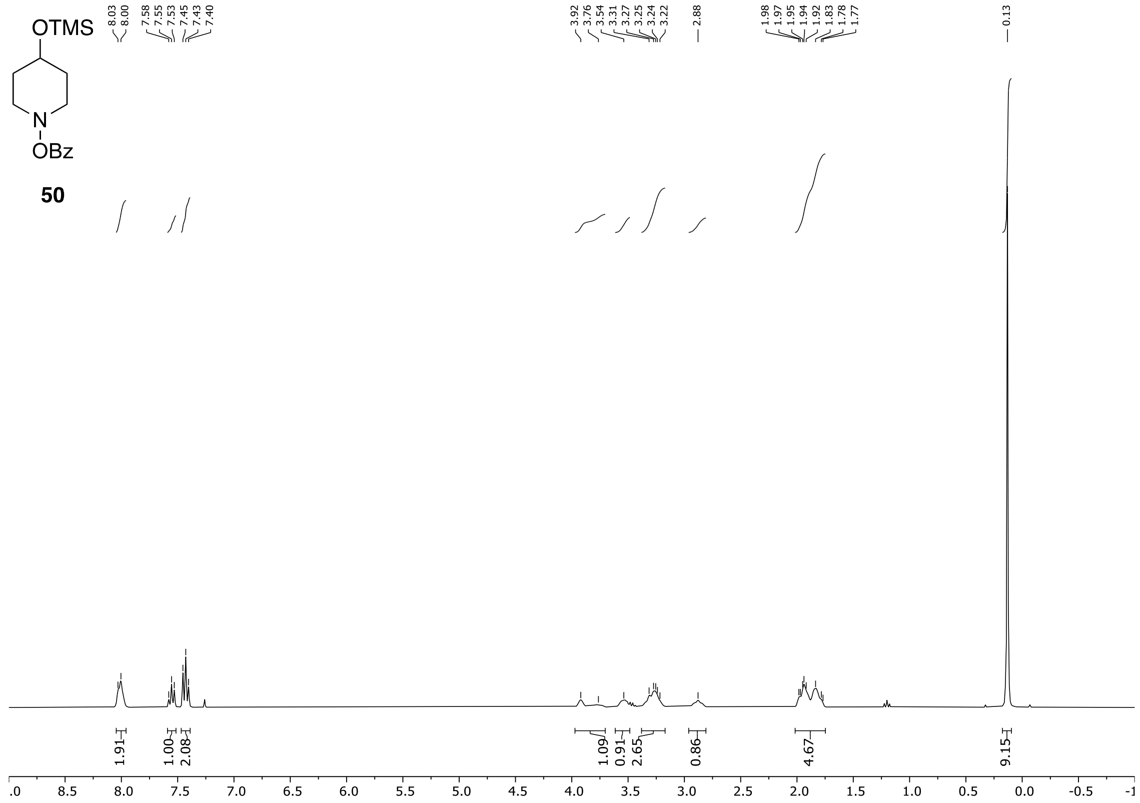

^{13}C-NMR, 75 MHz, $CDCl_3$

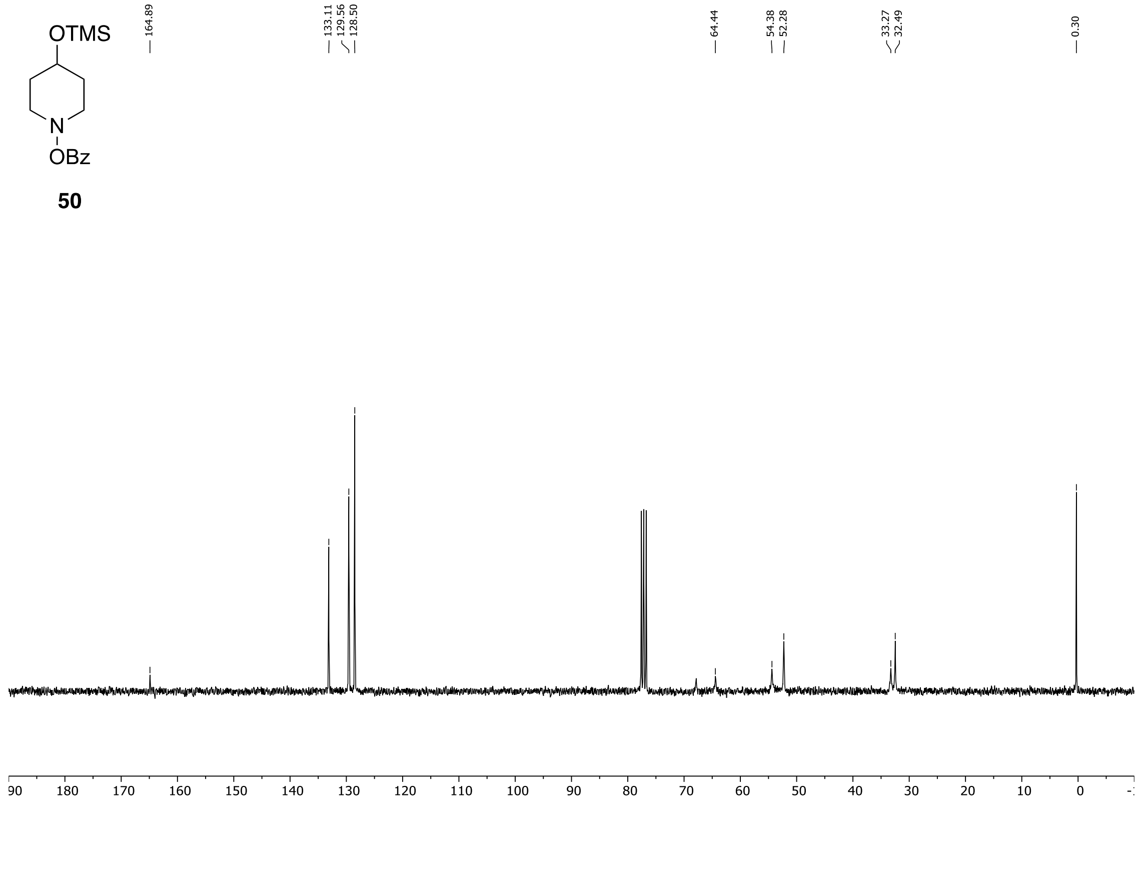

^{1}H-NMR, 300 MHz, $CDCl_3$

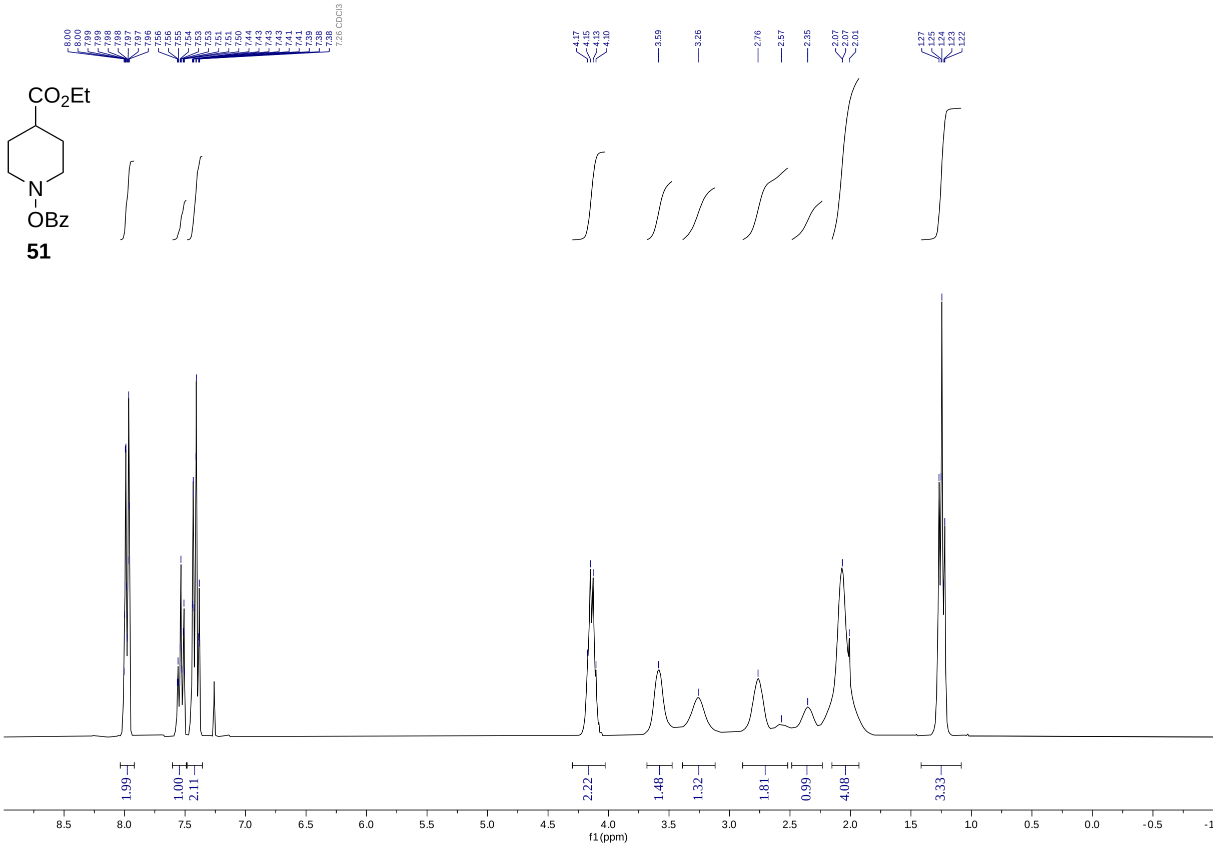

^{13}C-NMR, 75 MHz, $CDCl_3$

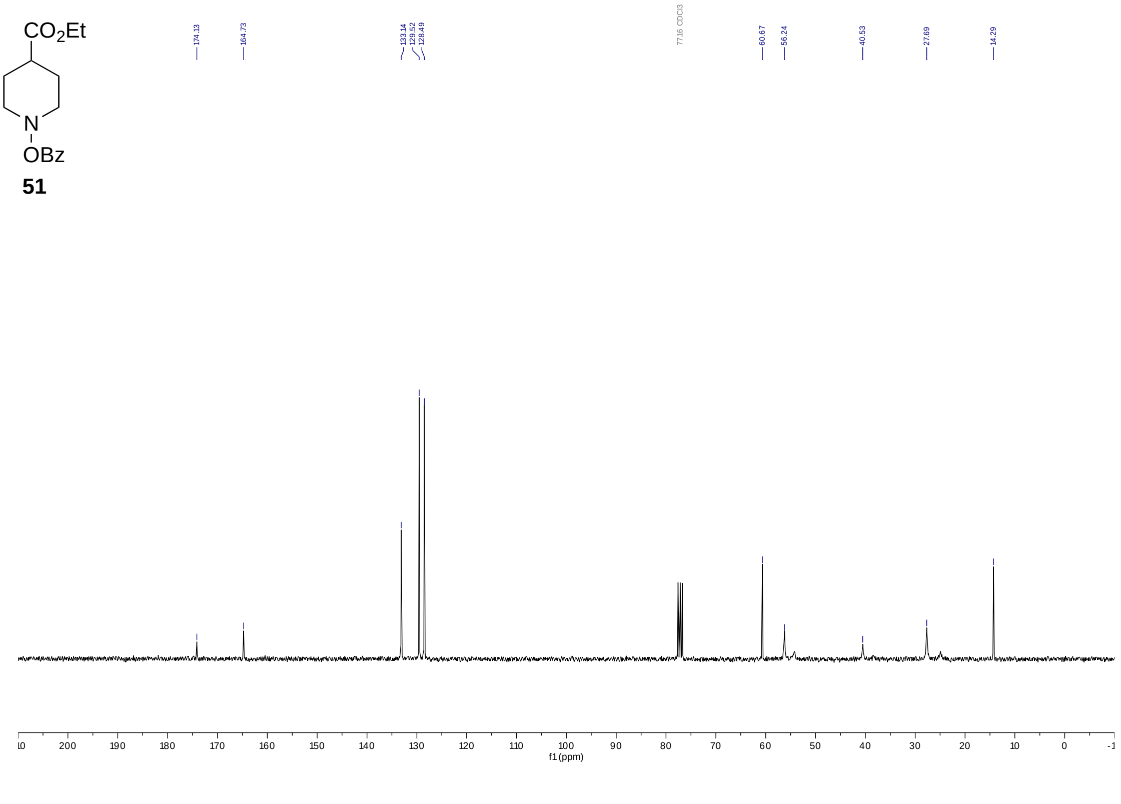

^{1}H-NMR, 500 MHz, $CDCl_3$

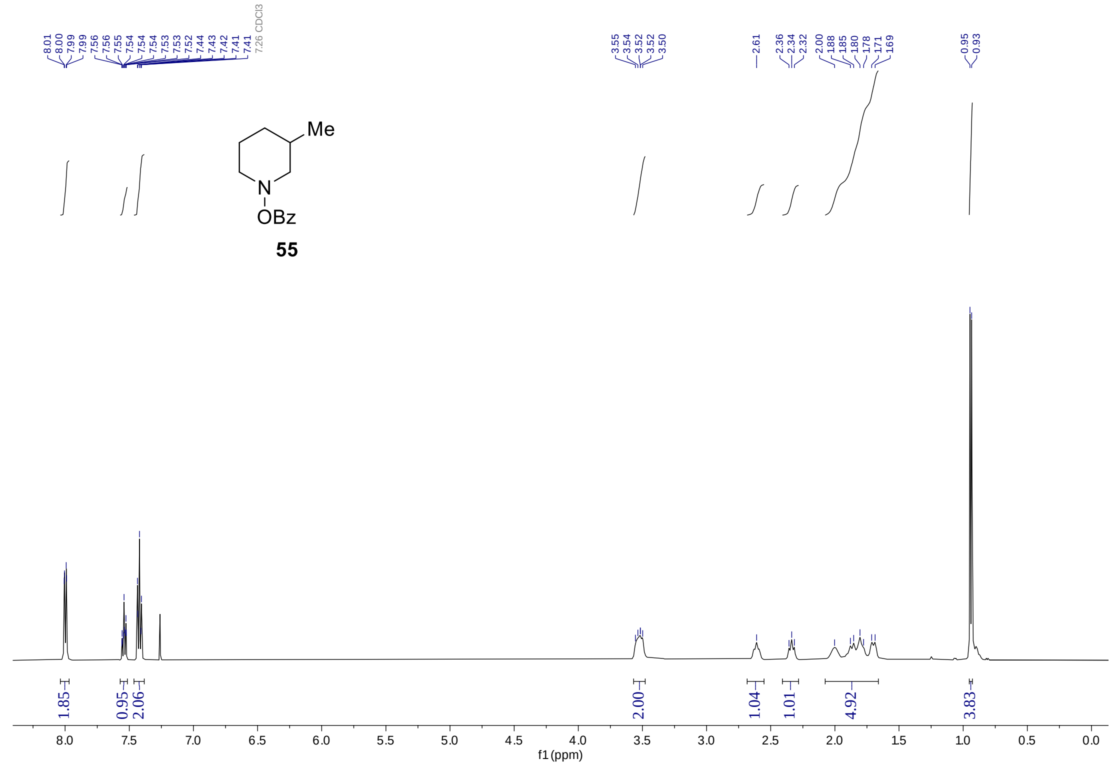

^{13}C-NMR, 126 MHz, $CDCl_3$

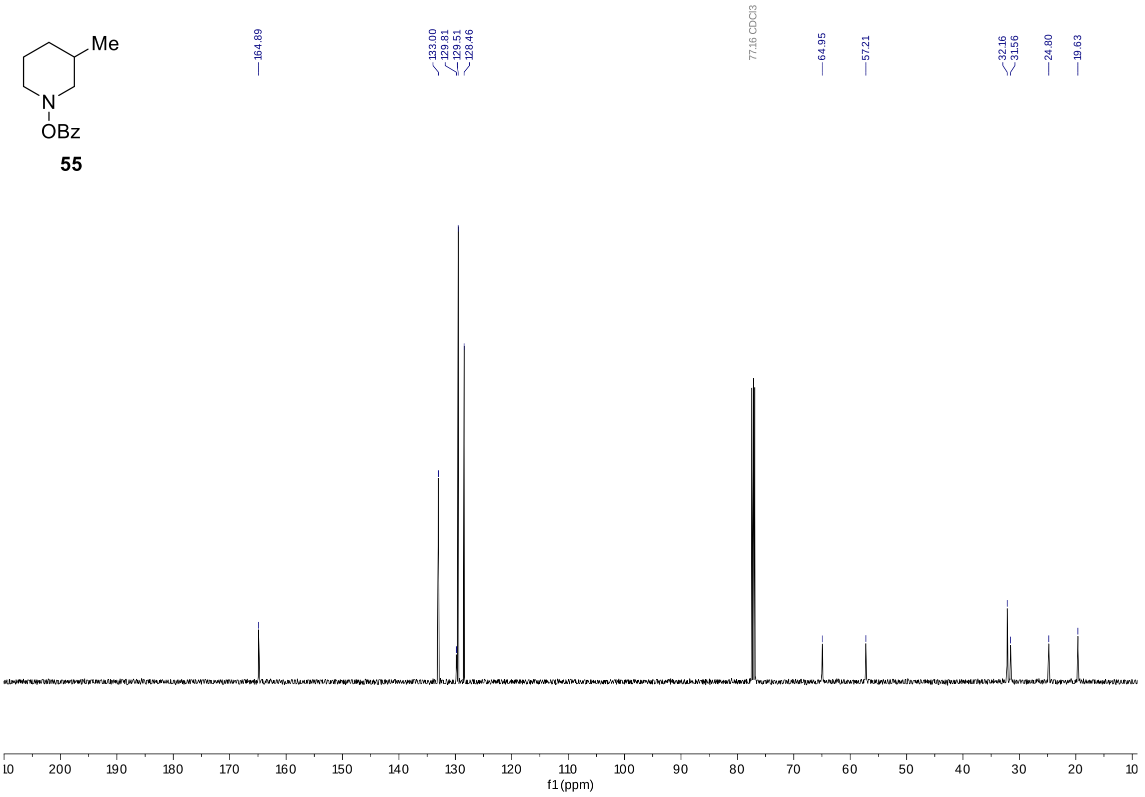

^{1}H-NMR, 500 MHz, $CDCl_3$

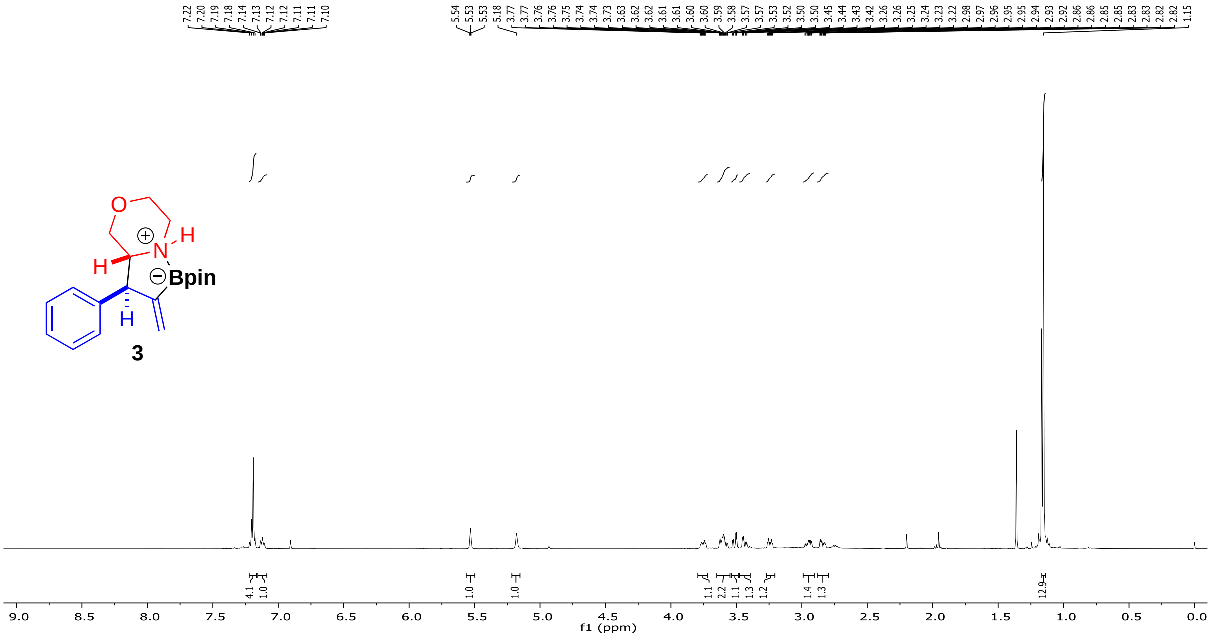

^{13}C-NMR, 75 MHz, $CDCl_3$

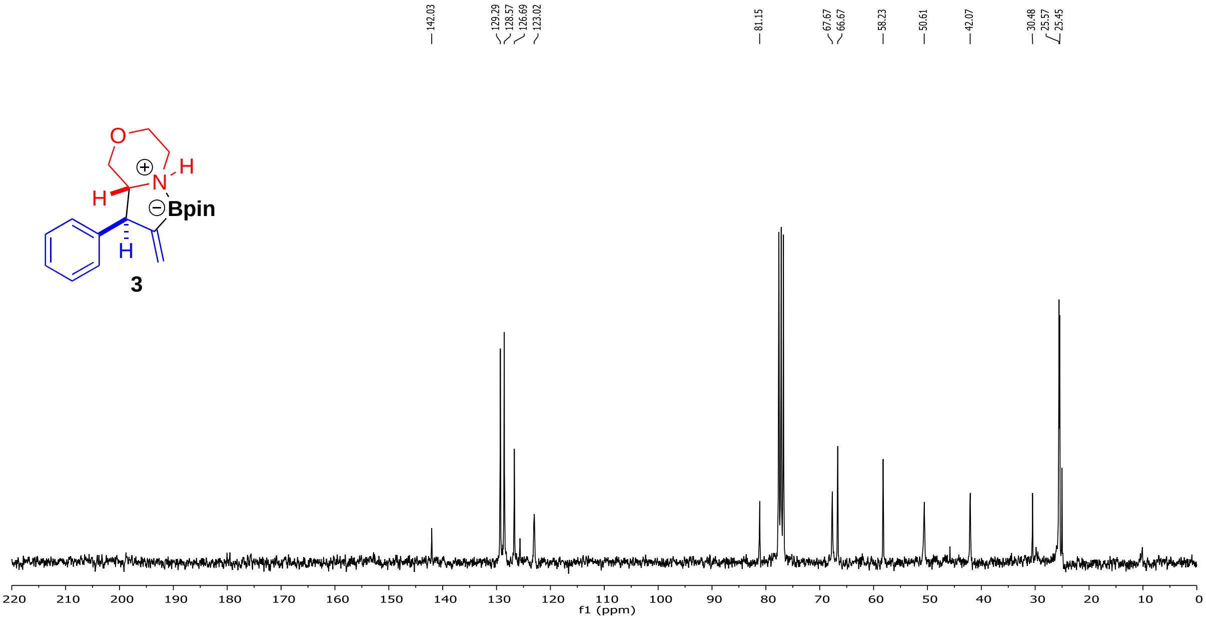

^{1}H-NMR, 500 MHz, $CDCl_3$

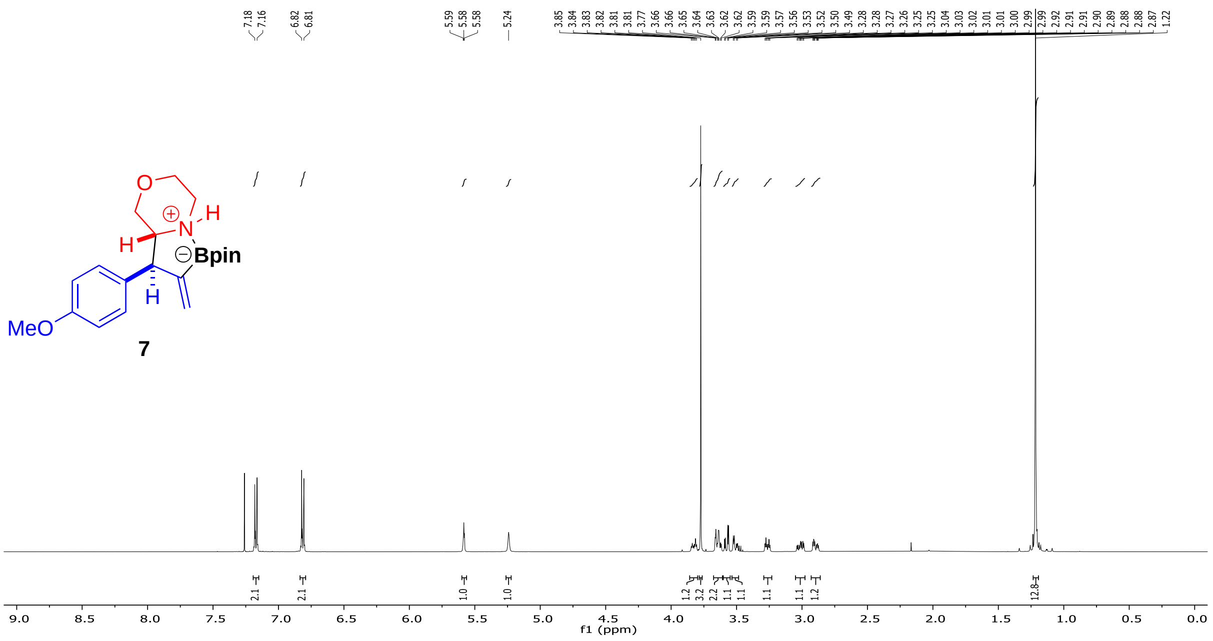

^{13}C-NMR, 75 MHz, $CDCl_3$

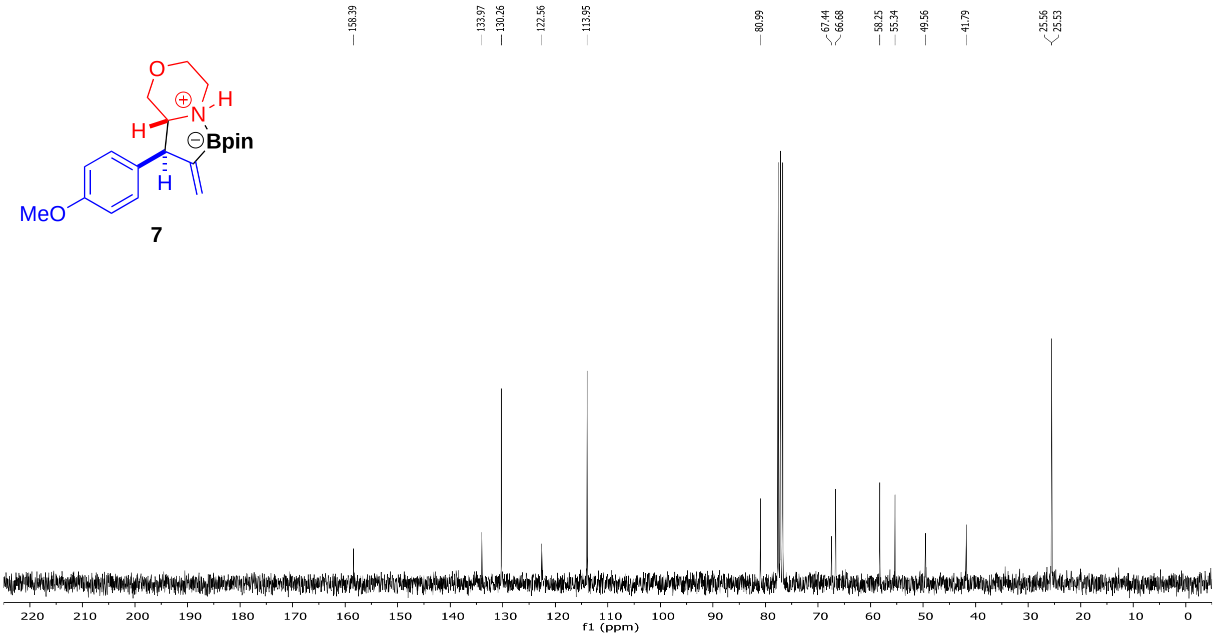

^{1}H-NMR, 500 MHz, $CDCl_3$

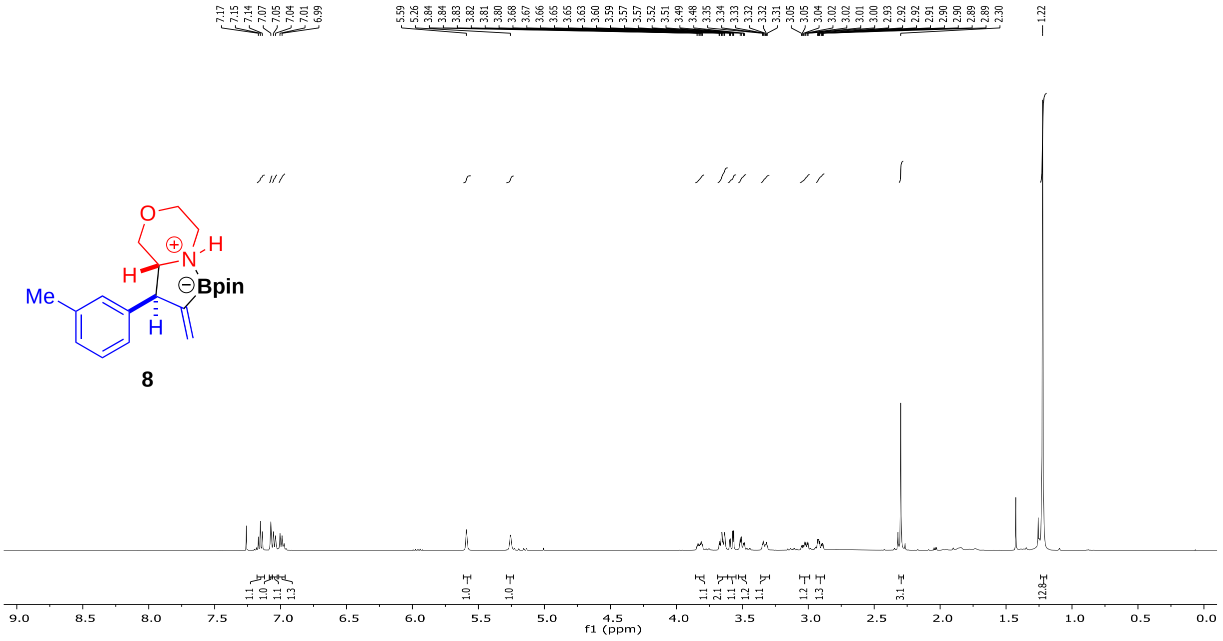

^{13}C-NMR, 75 MHz, $CDCl_3$

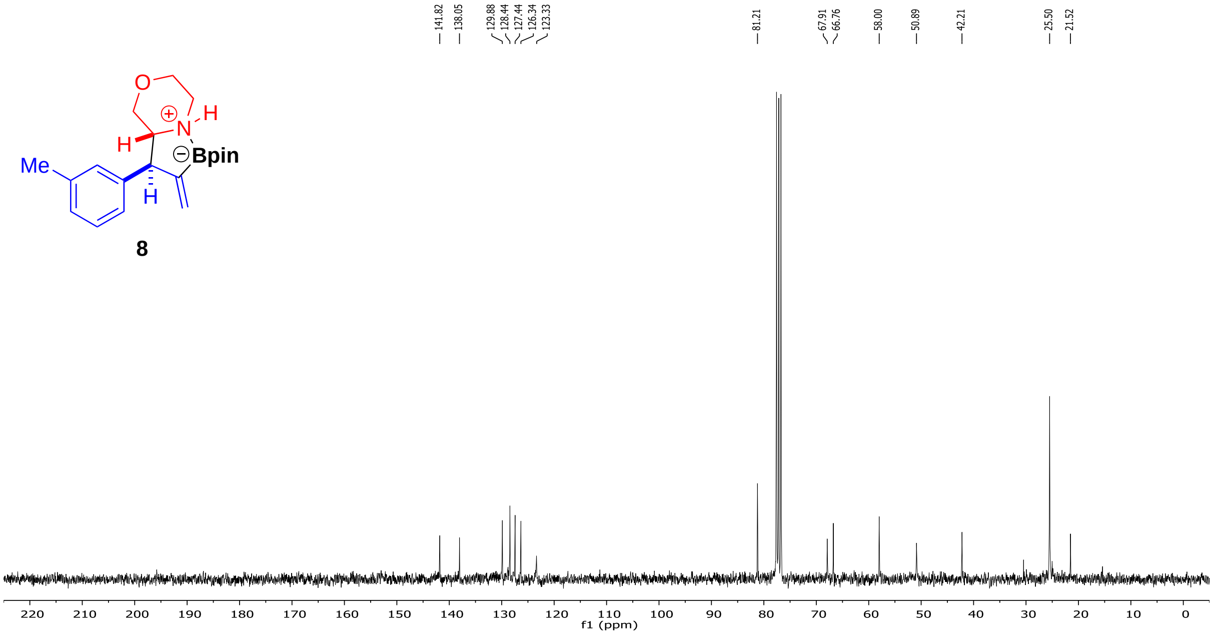

^{1}H-NMR, 500 MHz, $CDCl_3$

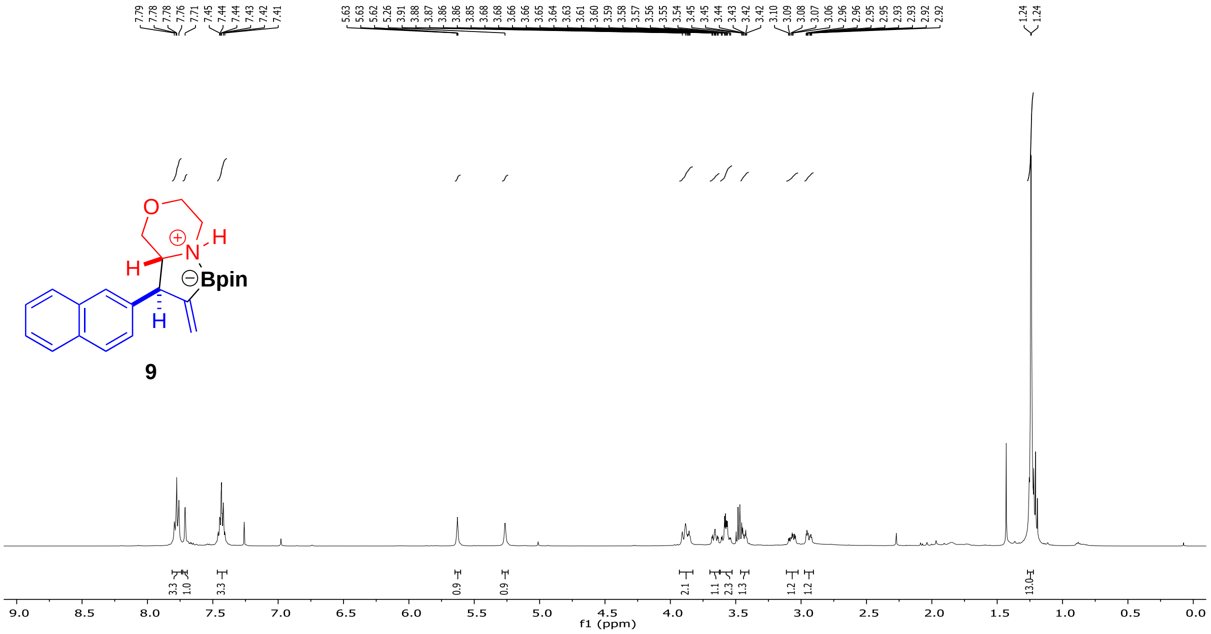

9

^{13}C-NMR, 75 MHz, $CDCl_3$

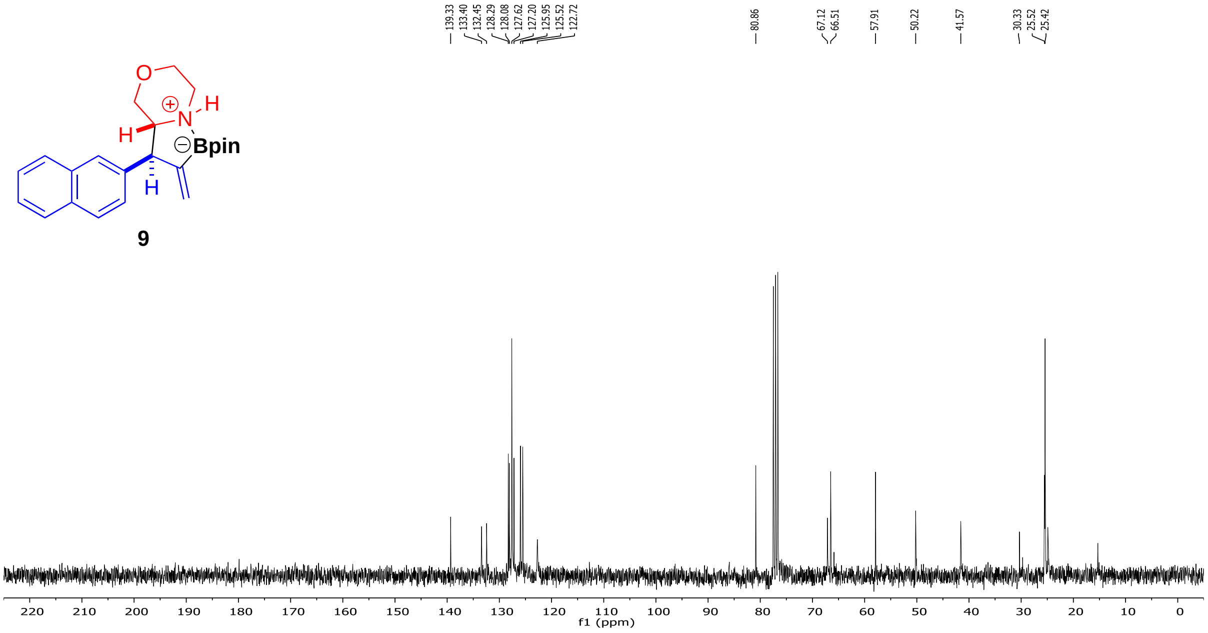

9

^{1}H-NMR, 500 MHz, $CDCl_3$

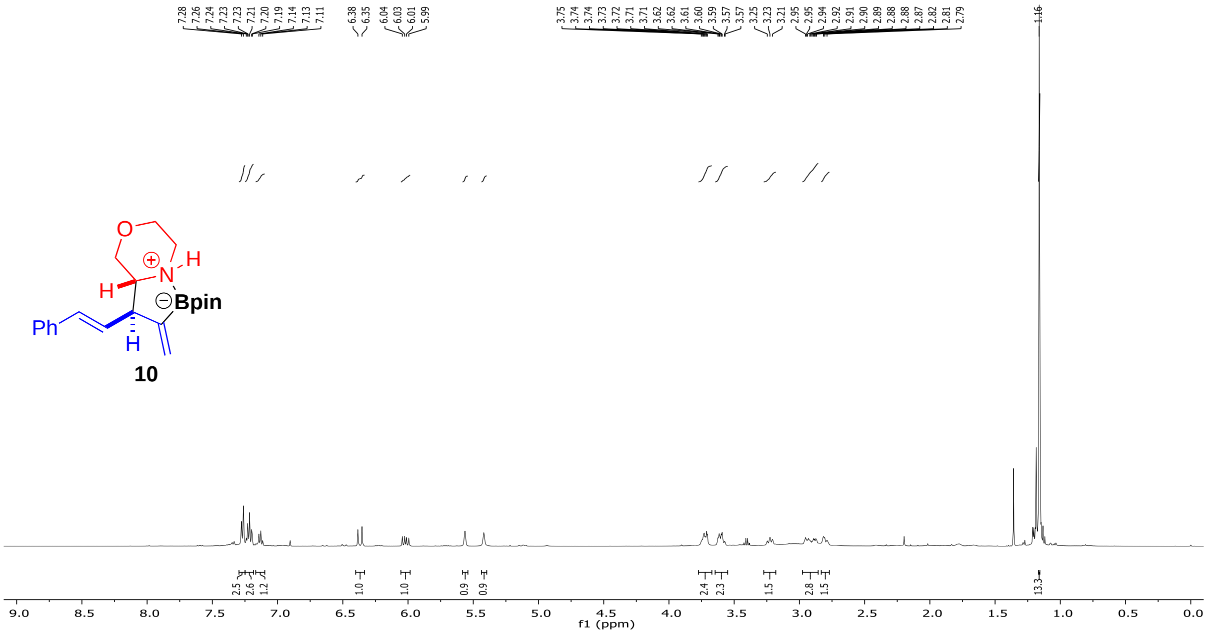

^{13}C-NMR, 75 MHz, $CDCl_3$

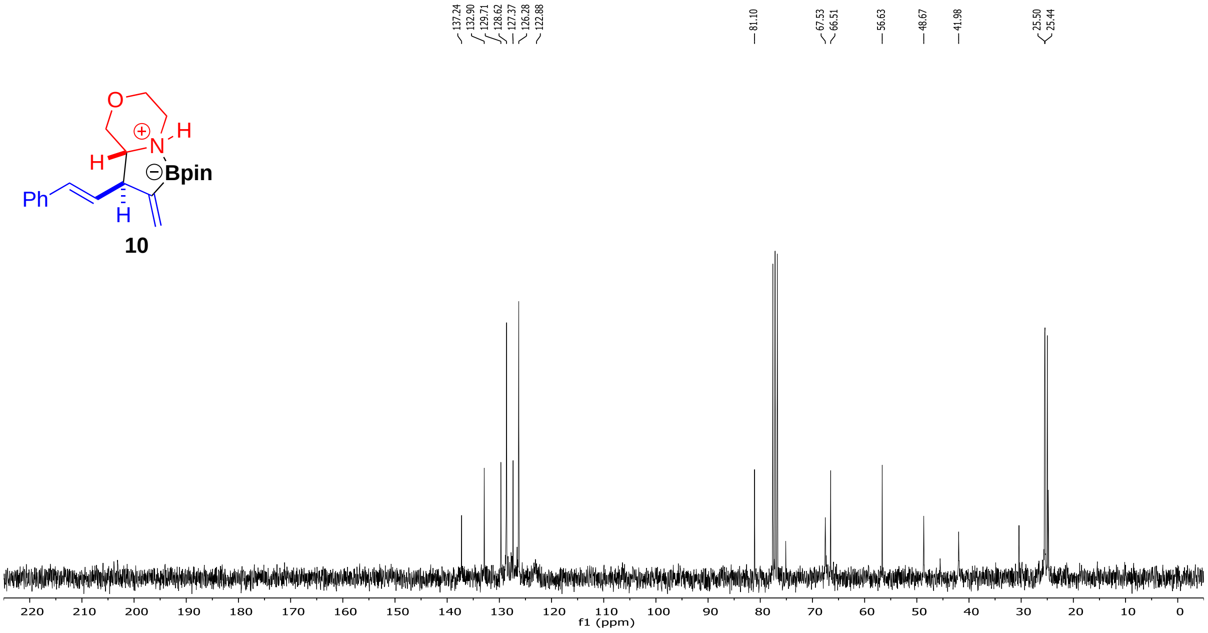

^{1}H-NMR, 500 MHz, $CDCl_3$

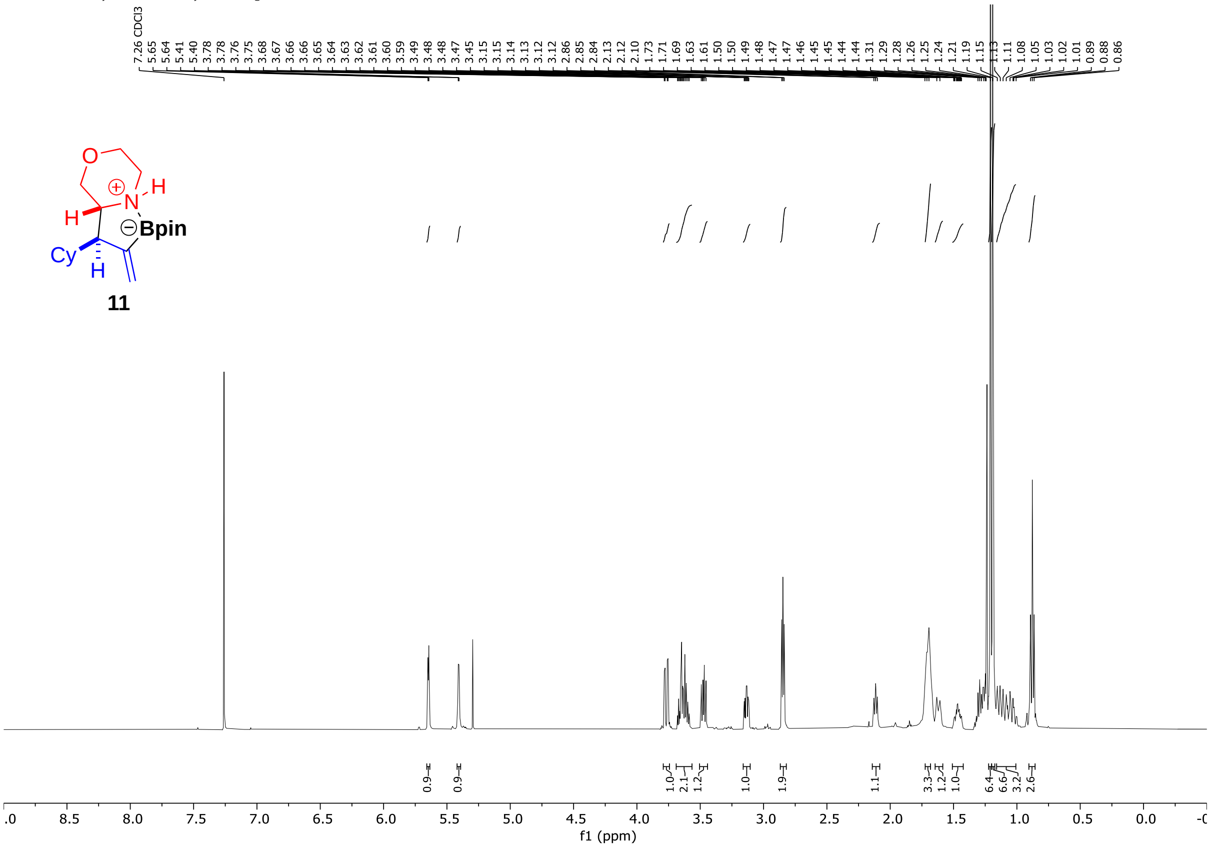

^{13}C-RMN, 75 MHz, $CDCl_3$

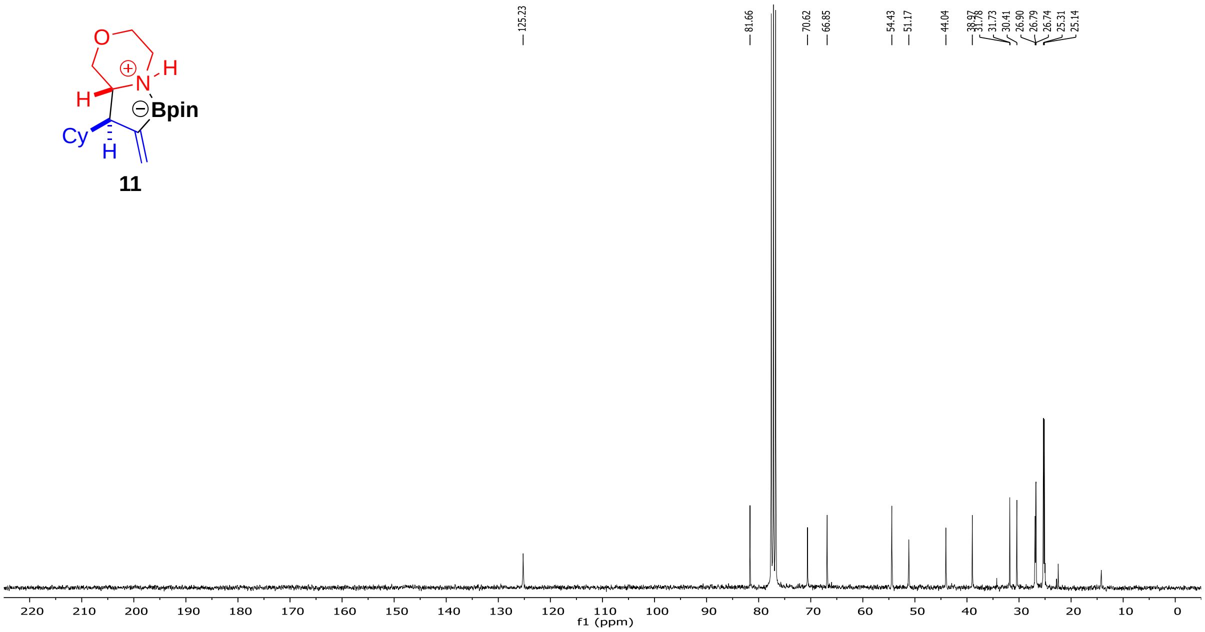

^{1}H-NMR, 500 MHz, $CDCl_3$

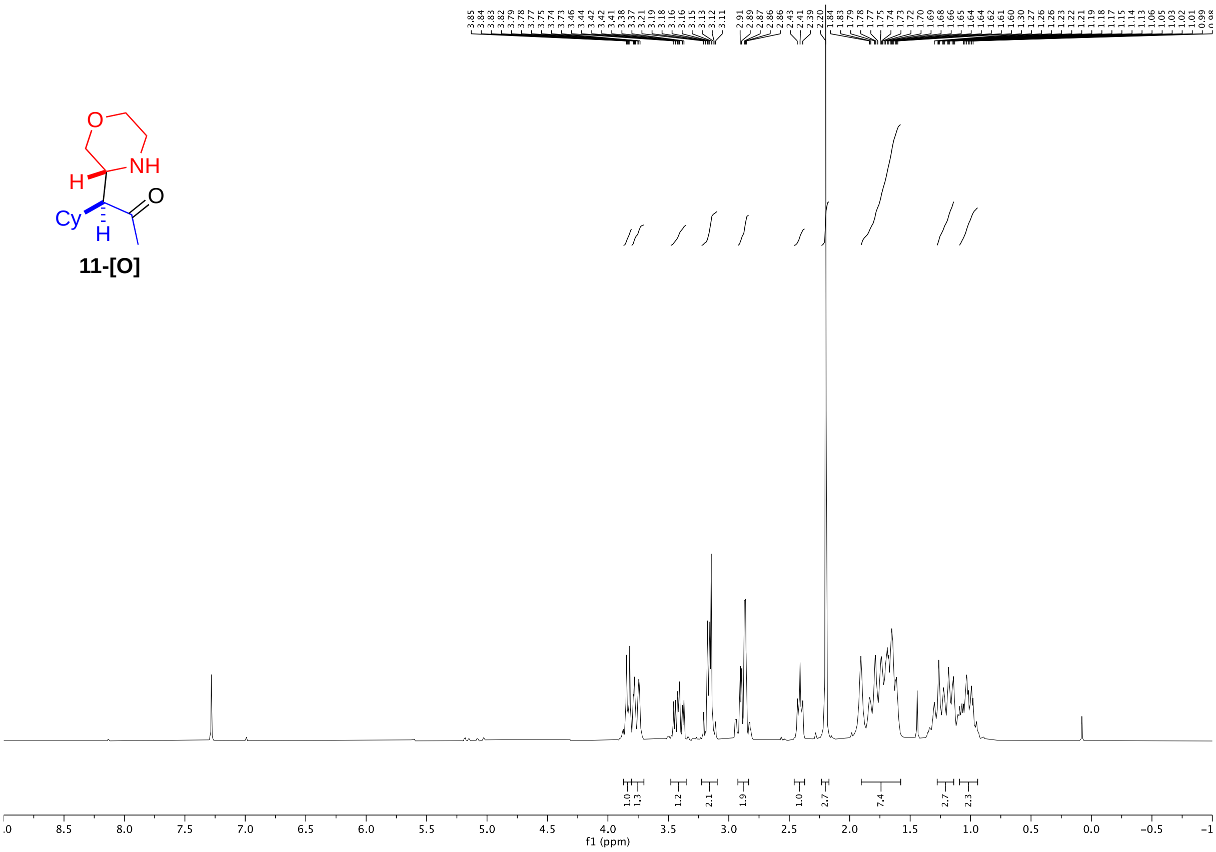

^{13}C-NMR, 75 MHz, $CDCl_3$

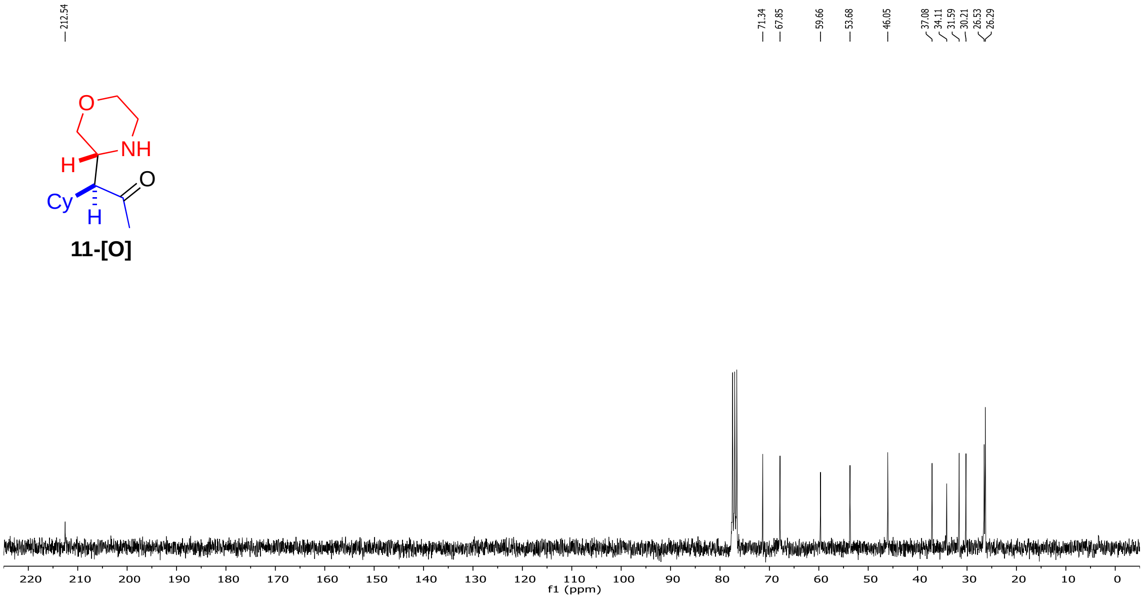

^{1}H-NMR, 500 MHz, $CDCl_3$

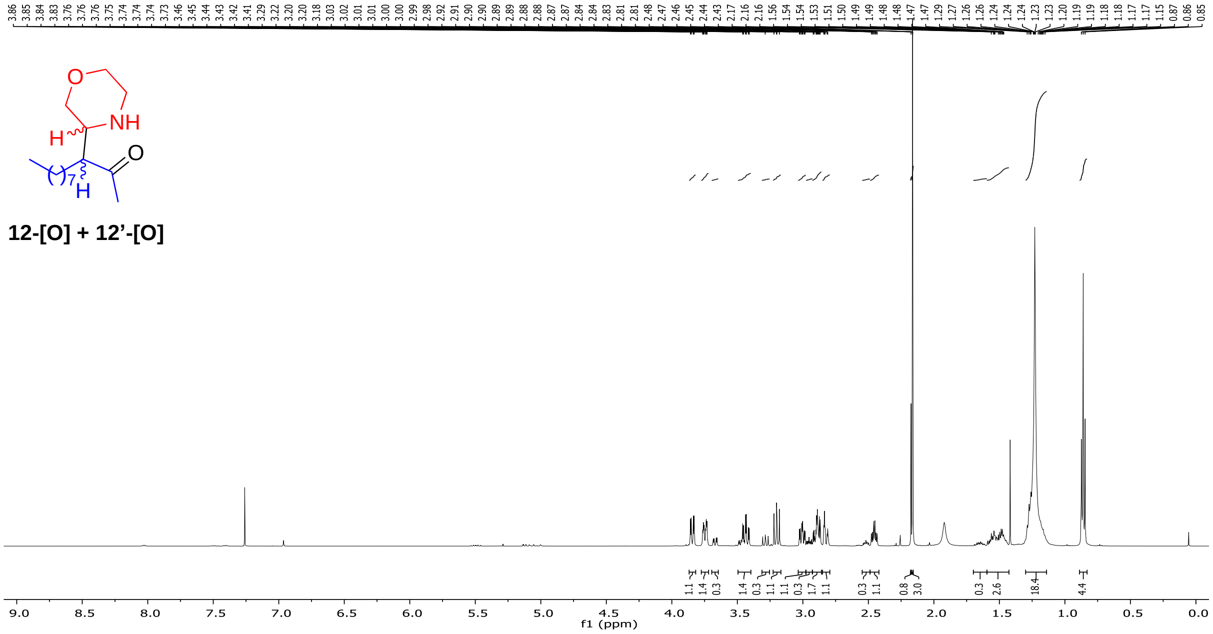

12-[O] + 12’-[O]

^{13}C-NMR, 75 MHz, $CDCl_3$

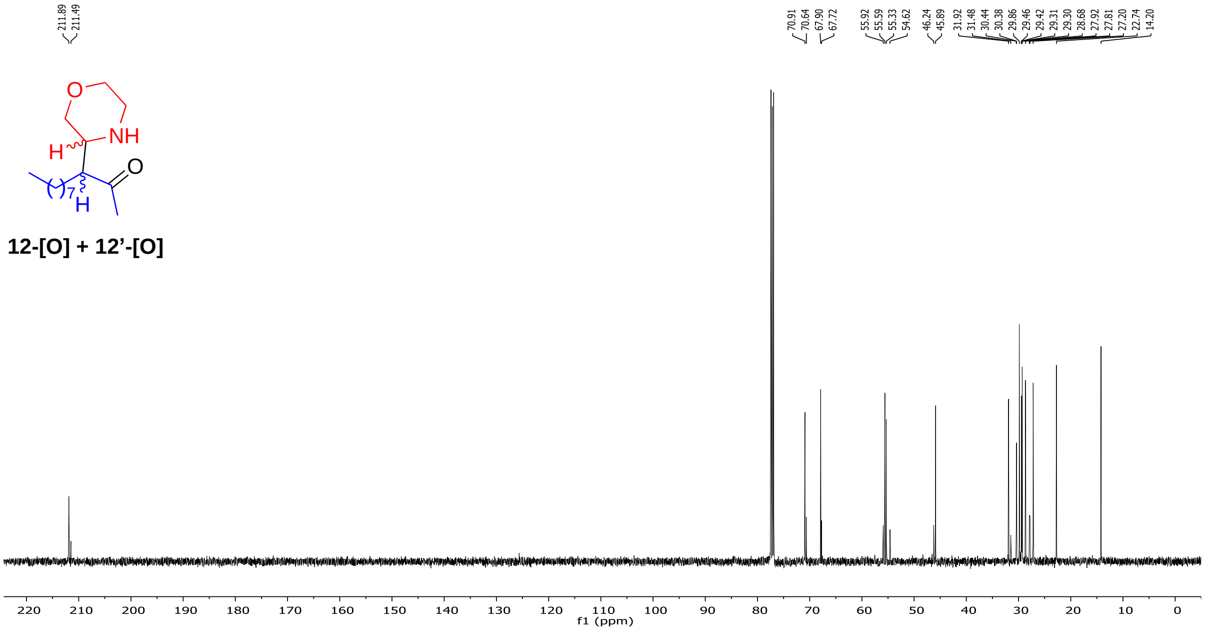

12-[O] + 12’-[O]

^{1}H-NMR, 500 MHz, $CDCl_3$

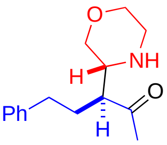

13-[O]

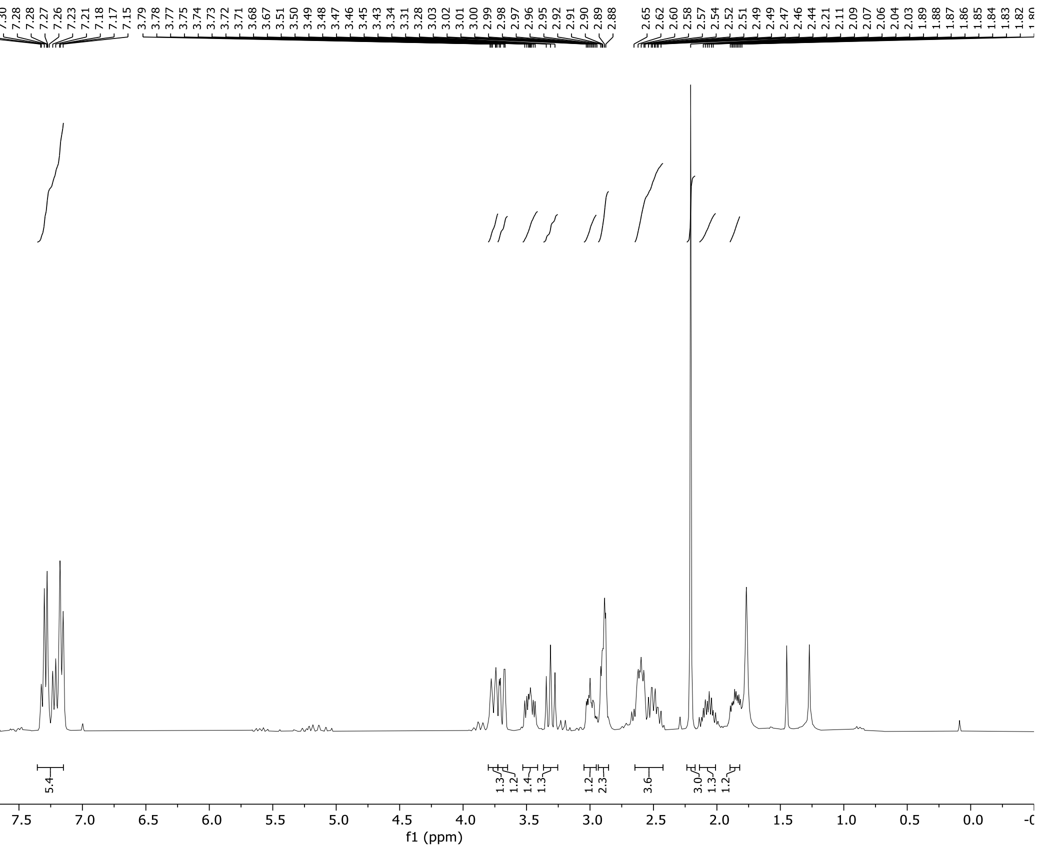

^{13}C-NMR, 75 MHz, $CDCl_3$

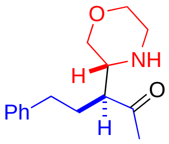

13-[O]

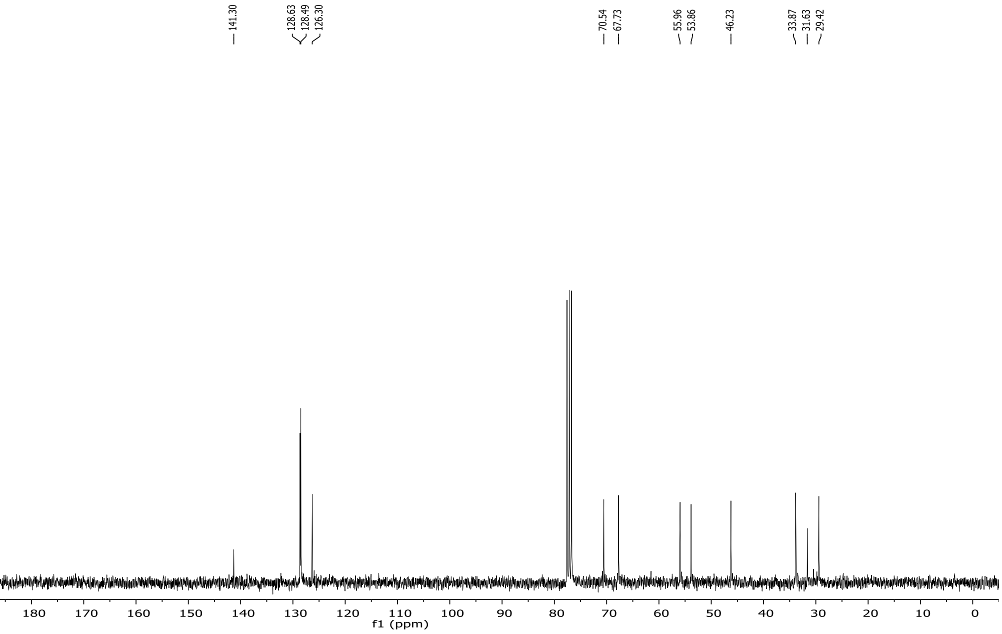

^{1}H-NMR, 500 MHz, $CDCl_3$

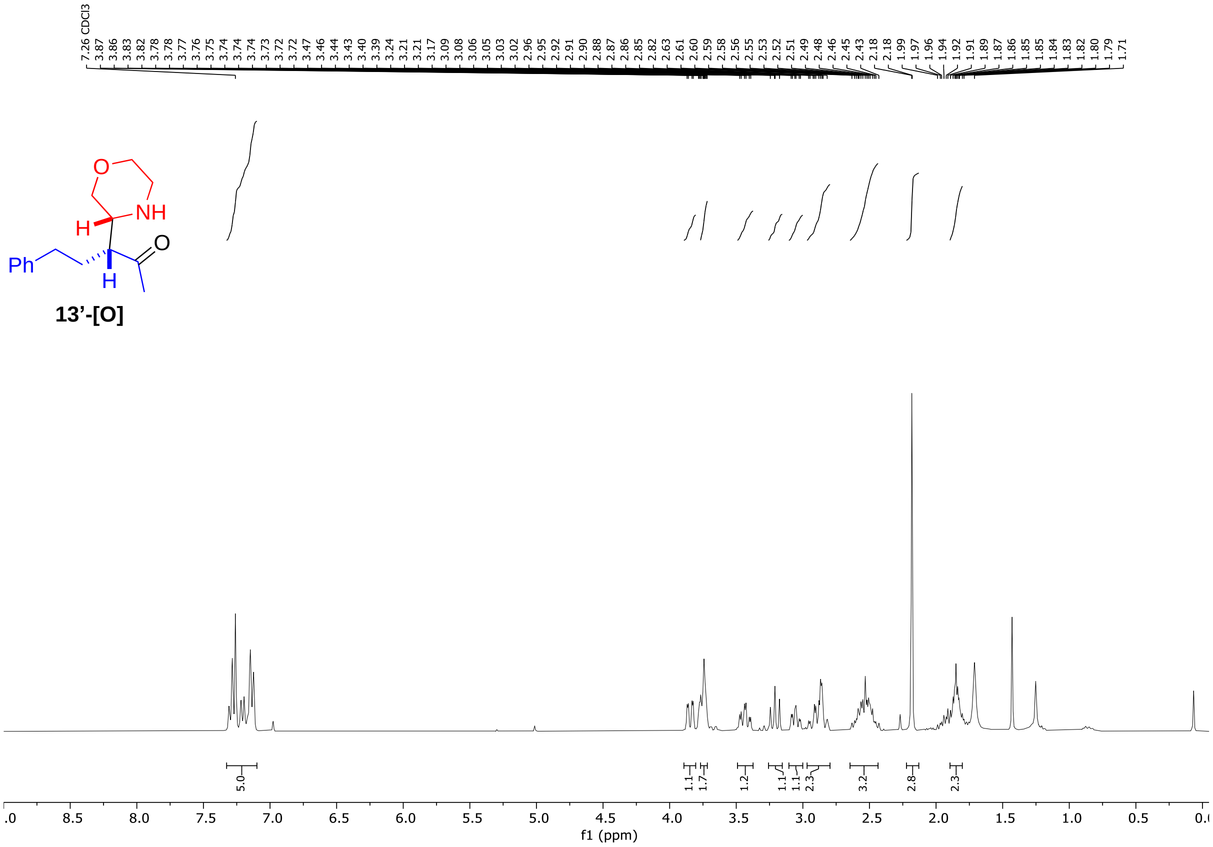

^{13}C-NMR, 75 MHz, $CDCl_3$

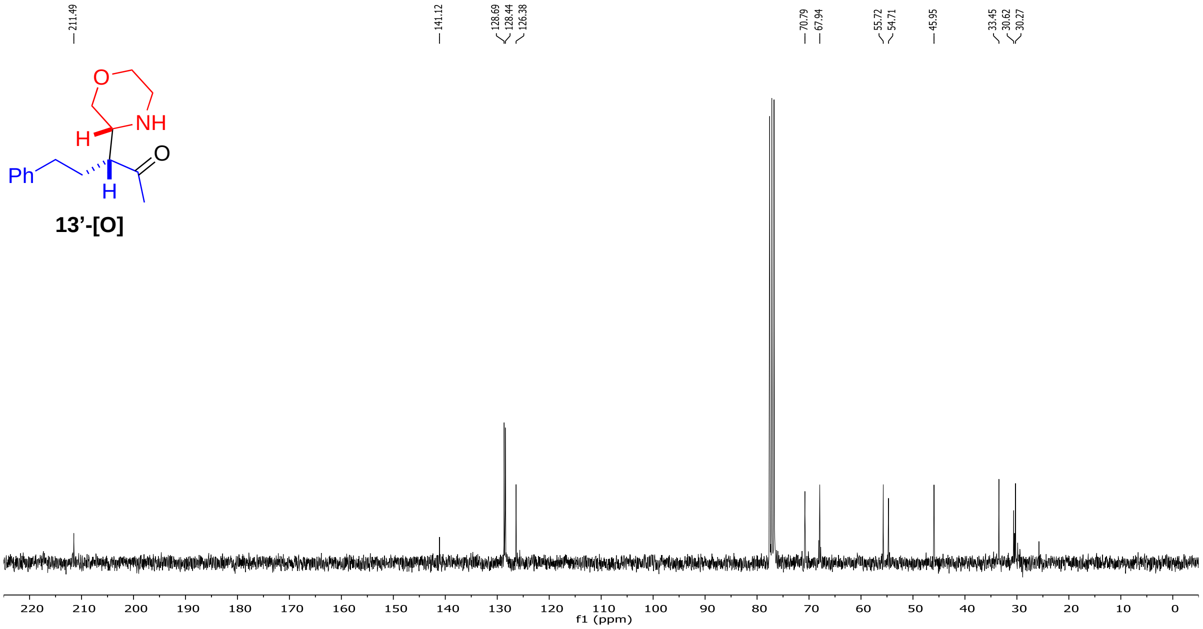

^{1}H-NMR, 500 MHz, $CDCl_3$

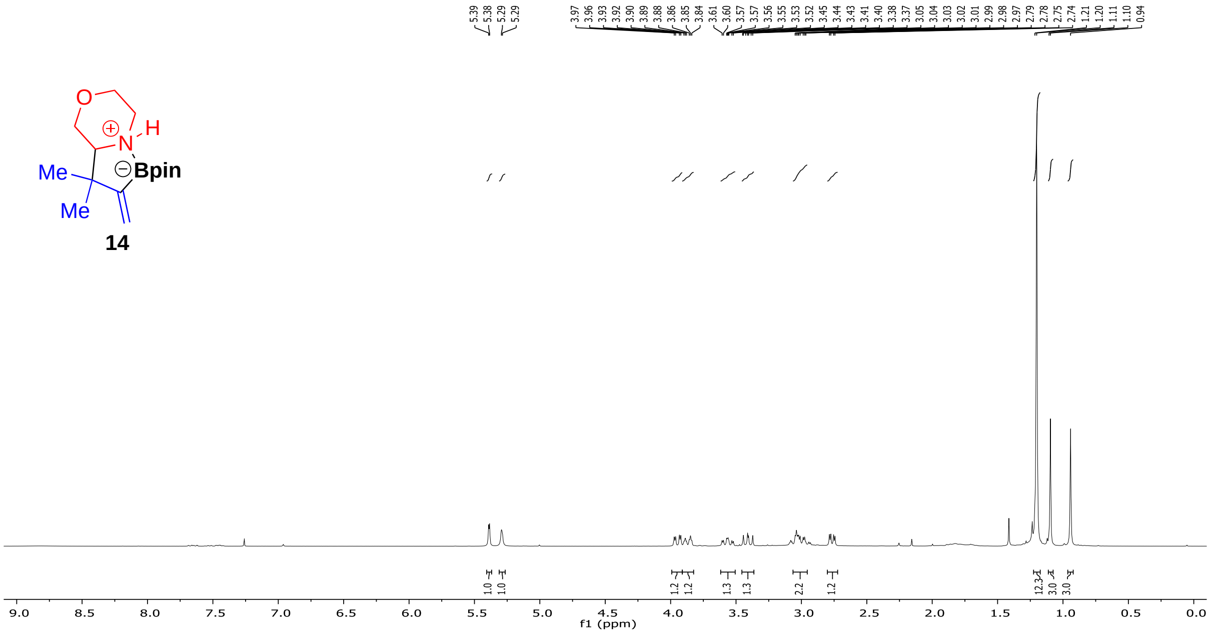

^{13}C-NMR, 75 MHz, $CDCl_3$

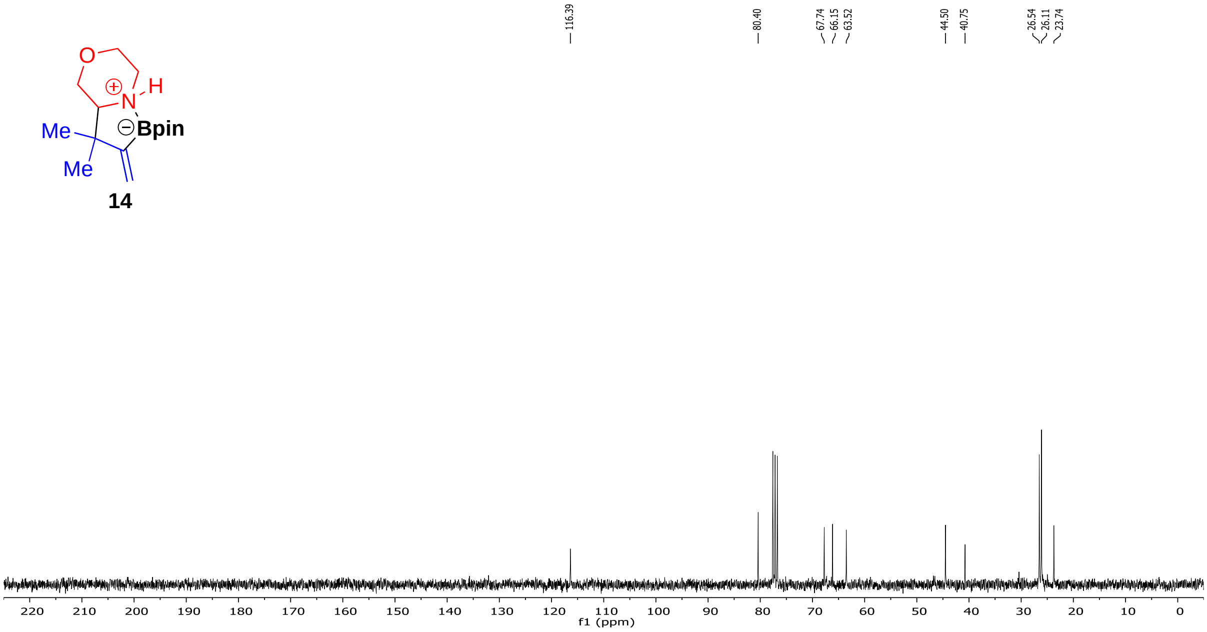

^{1}H-NMR, 500 MHz, $CDCl_3$

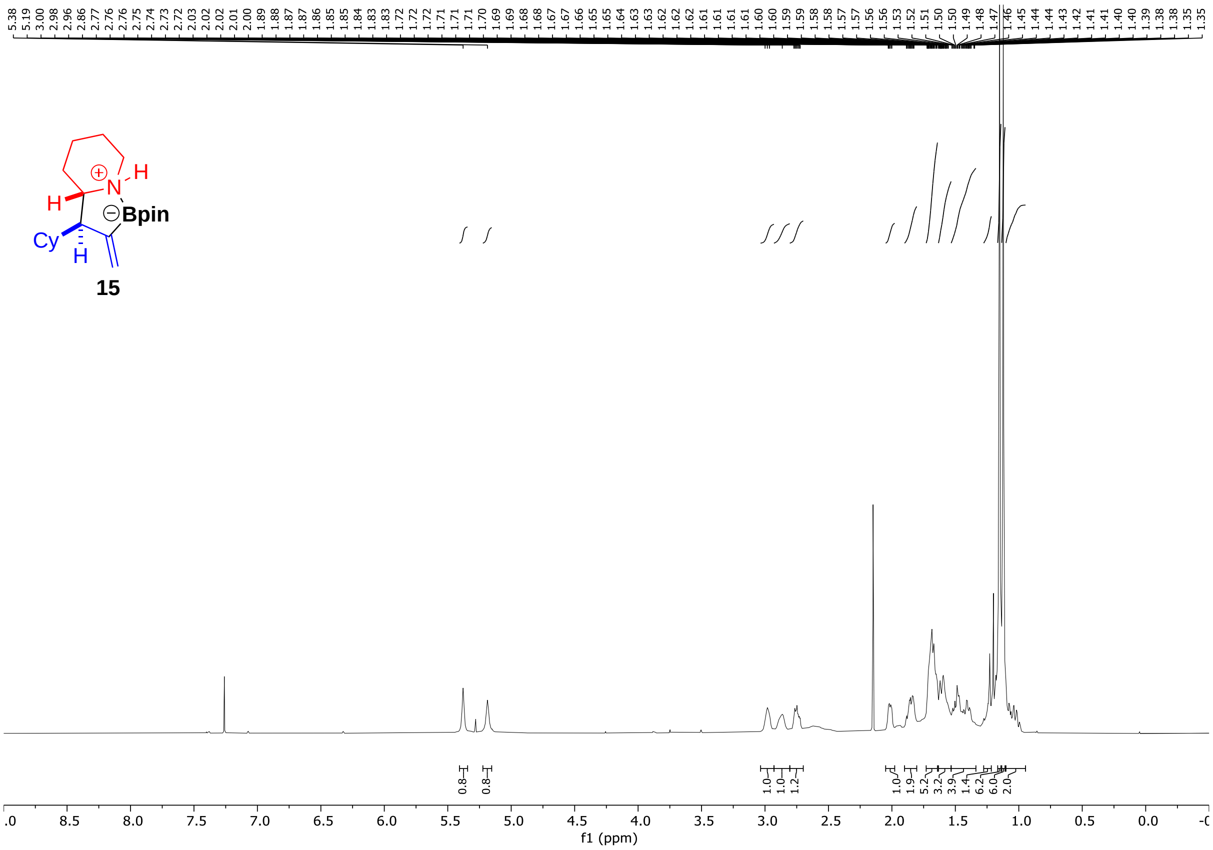

^{13}C-NMR, 126 MHz, $CDCl_3$

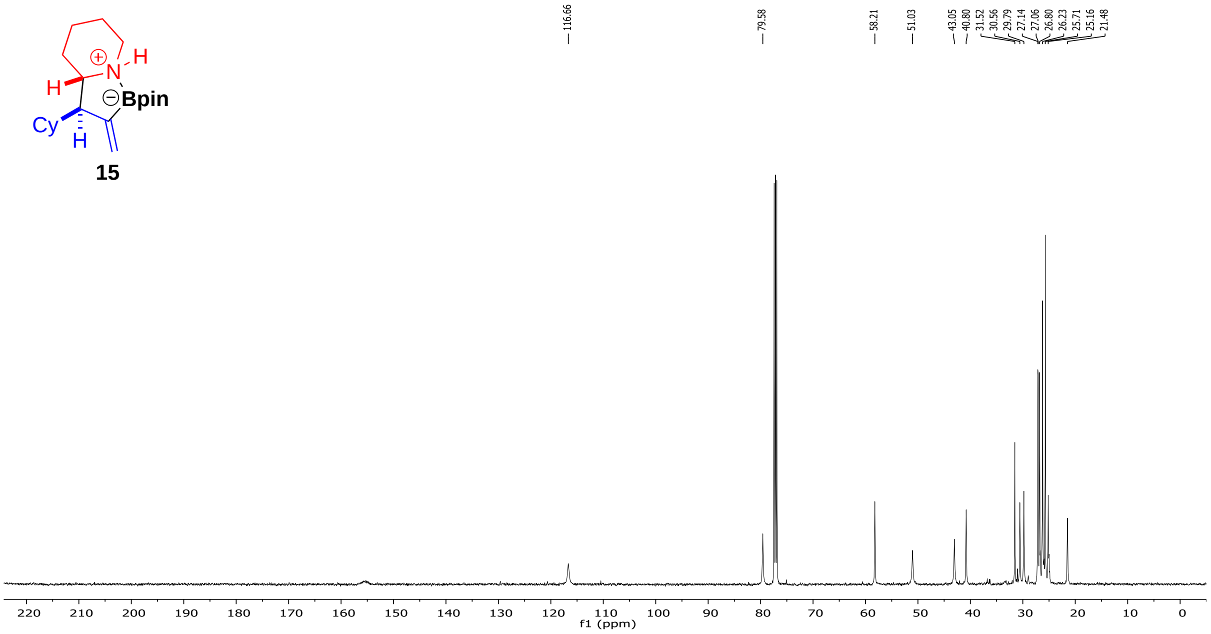

^{1}H-NMR, 500 MHz, $CDCl_3$

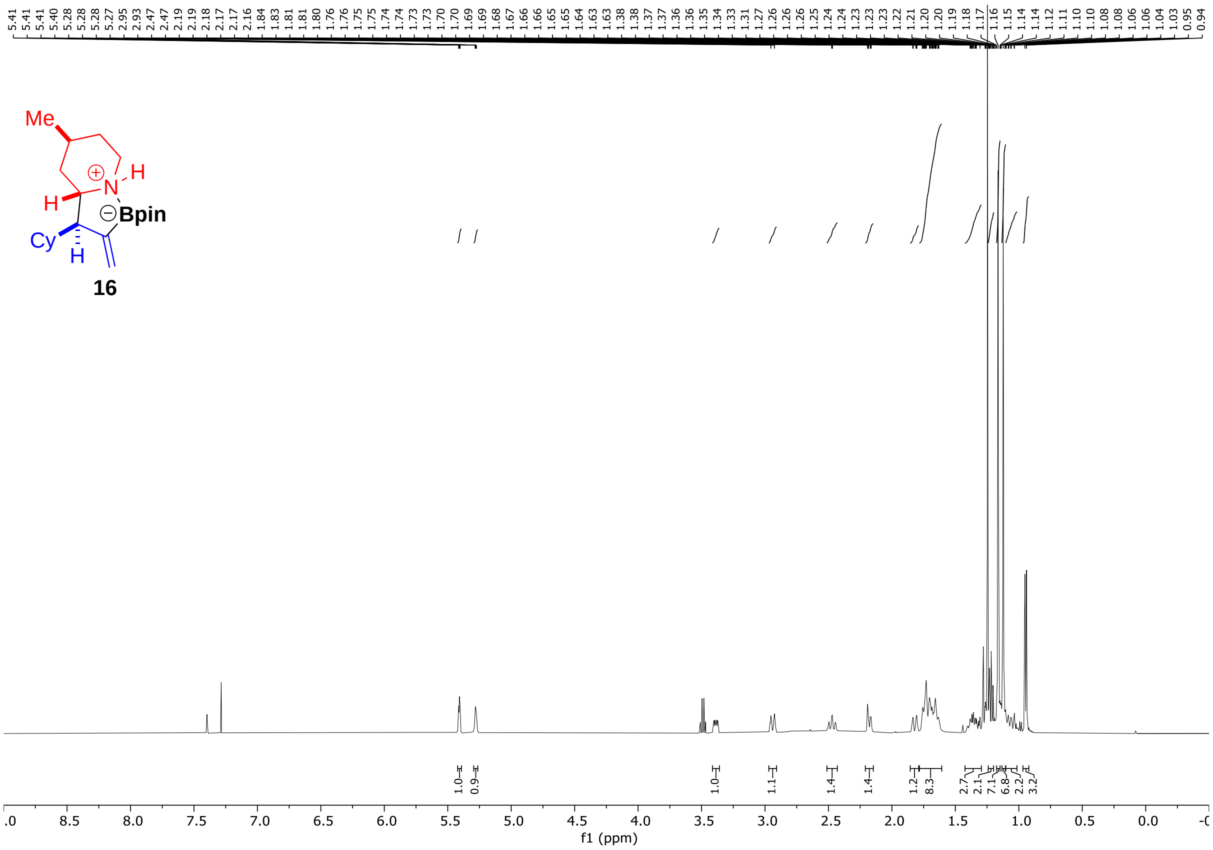

^{13}C-NMR, 126 MHz, $CDCl_3$

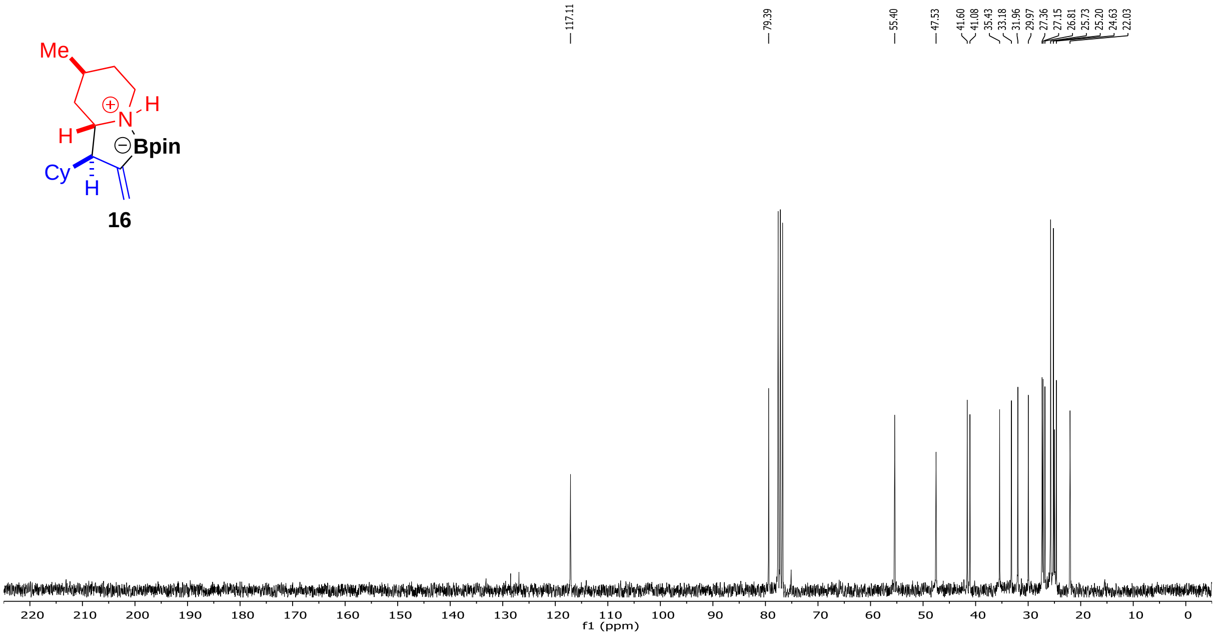

^{1}H-NMR, 500 MHz, $CDCl_3$

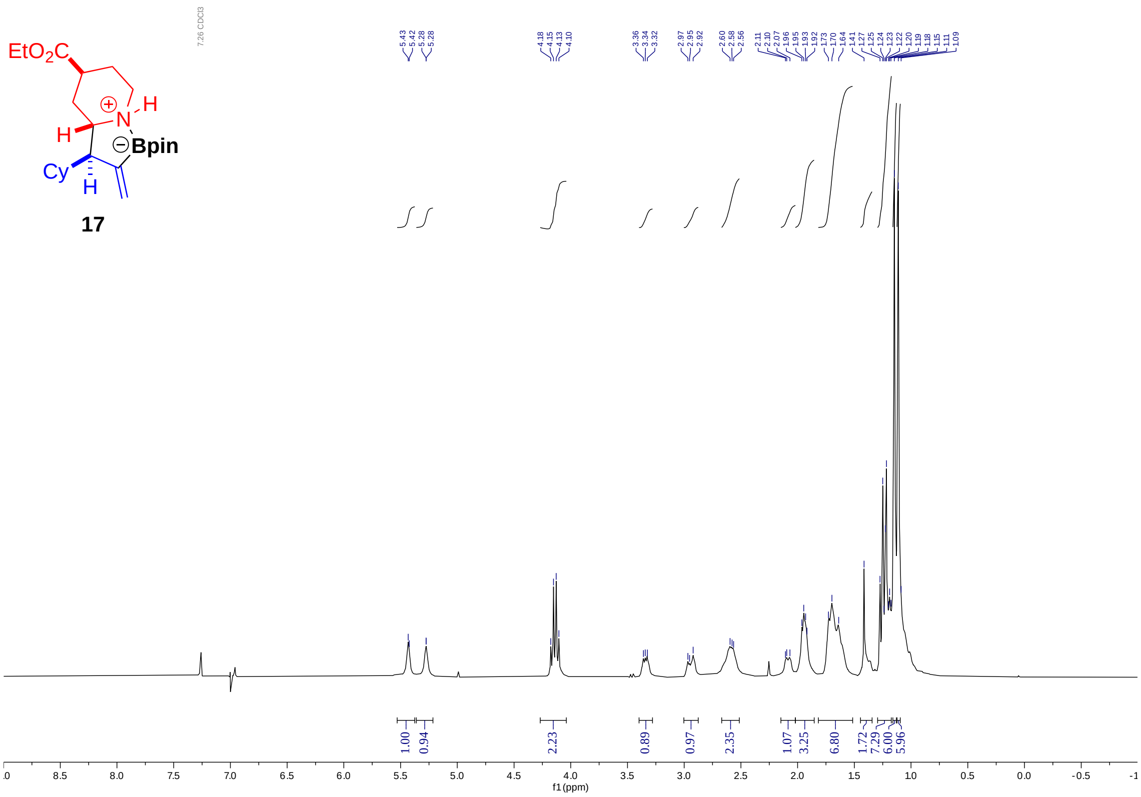

^{13}C-NMR, 126 MHz, $CDCl_3$

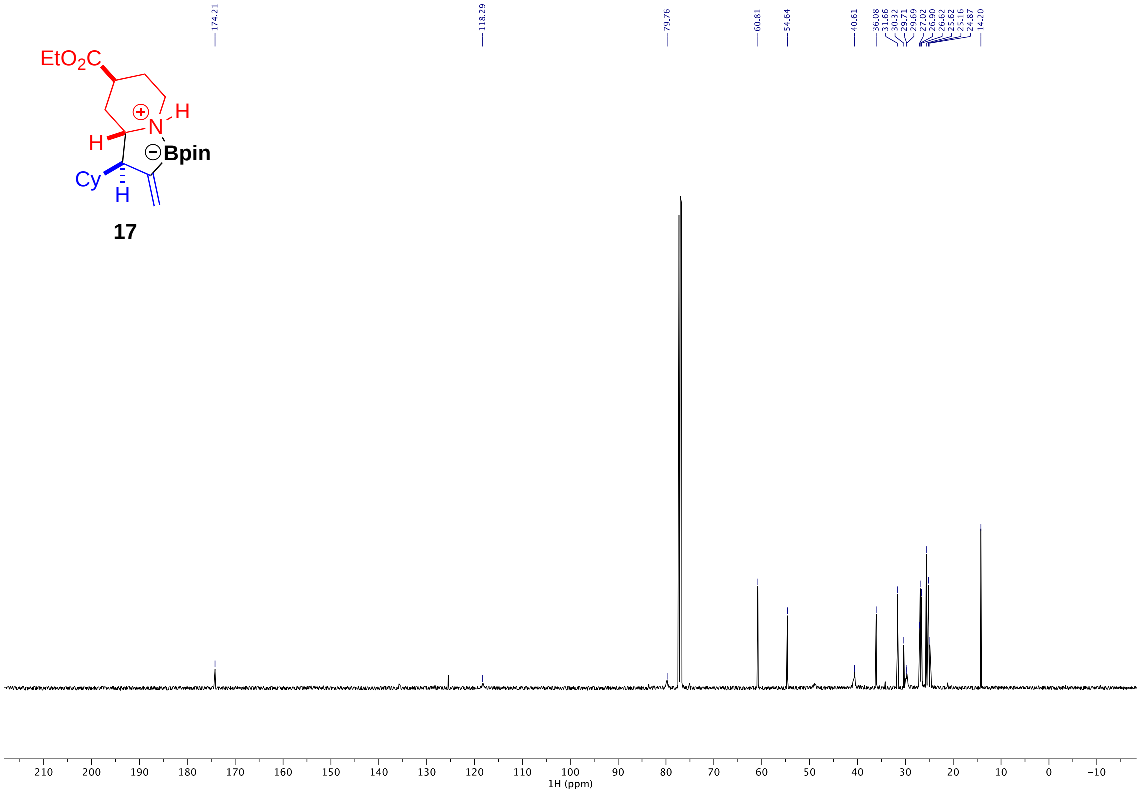

^{1}H-NMR, 500 MHz, $CDCl_3$

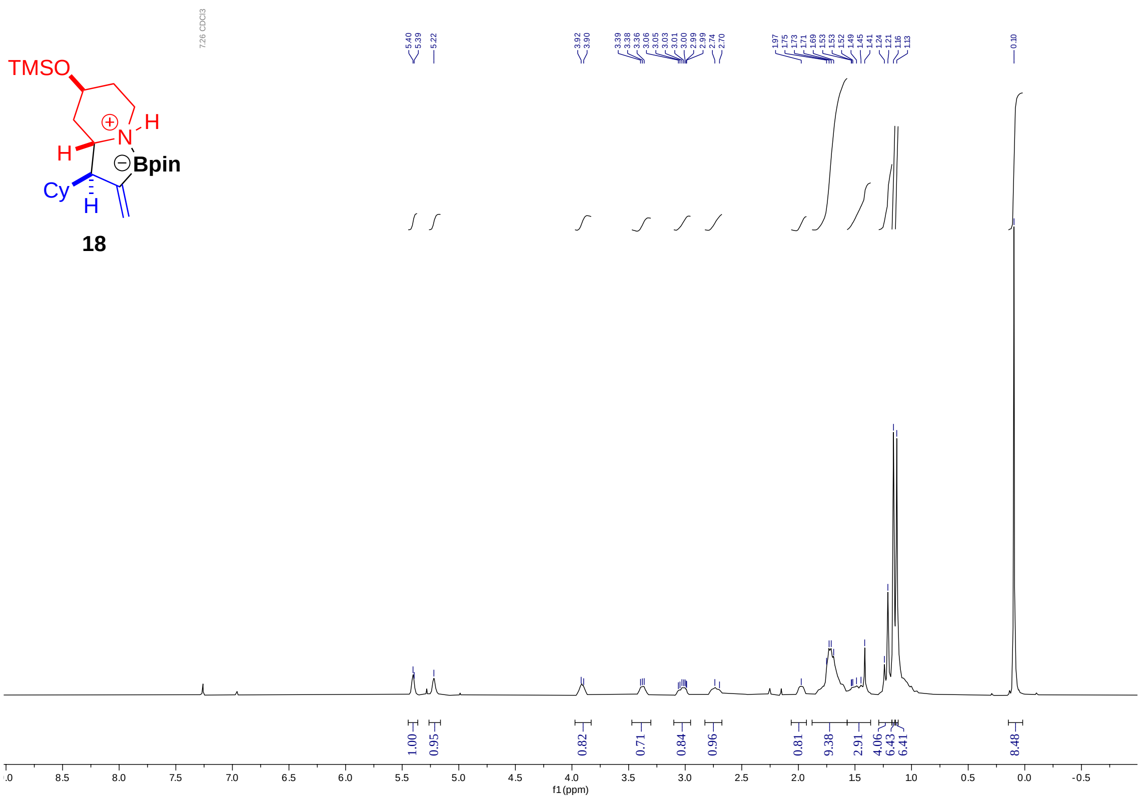

^{13}C-NMR, 126 MHz, $CDCl_3$

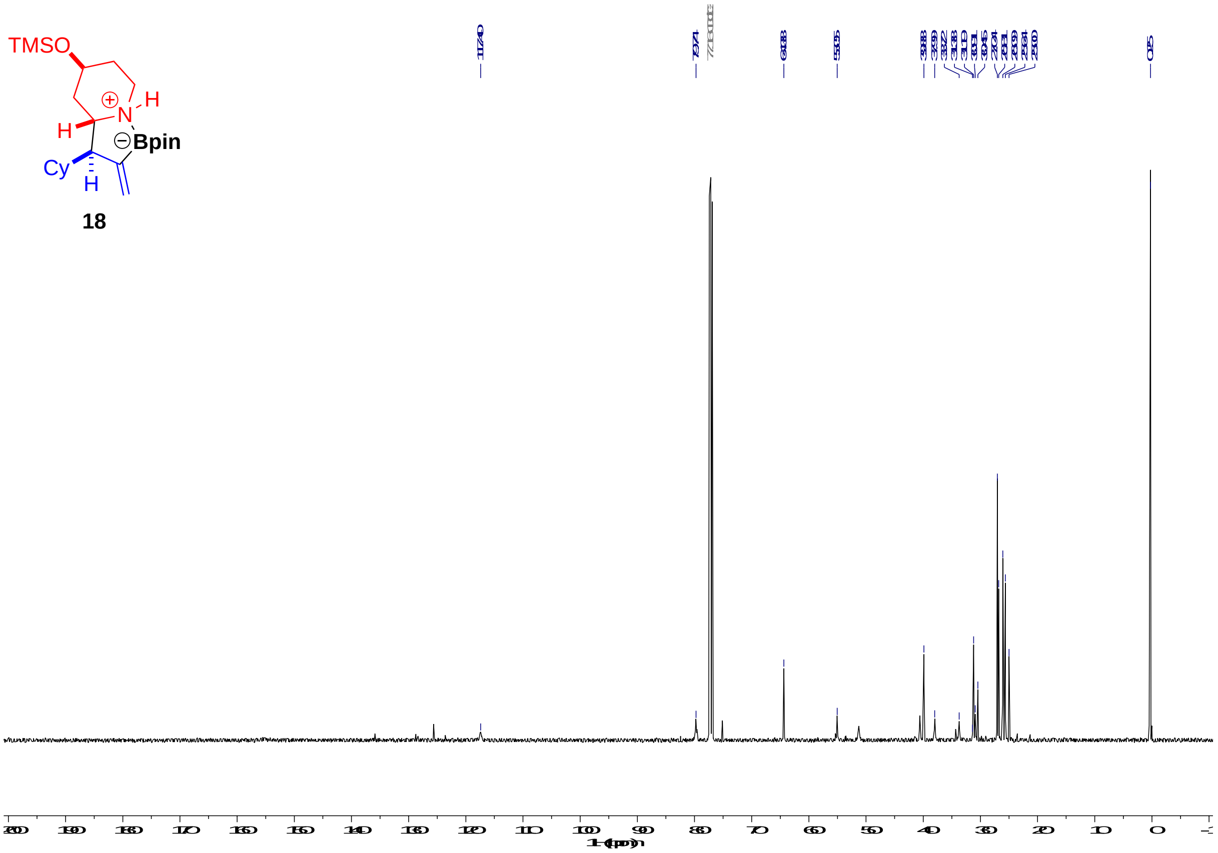

^{1}H-NMR, 500 MHz, $CDCl_3$

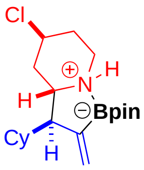

19

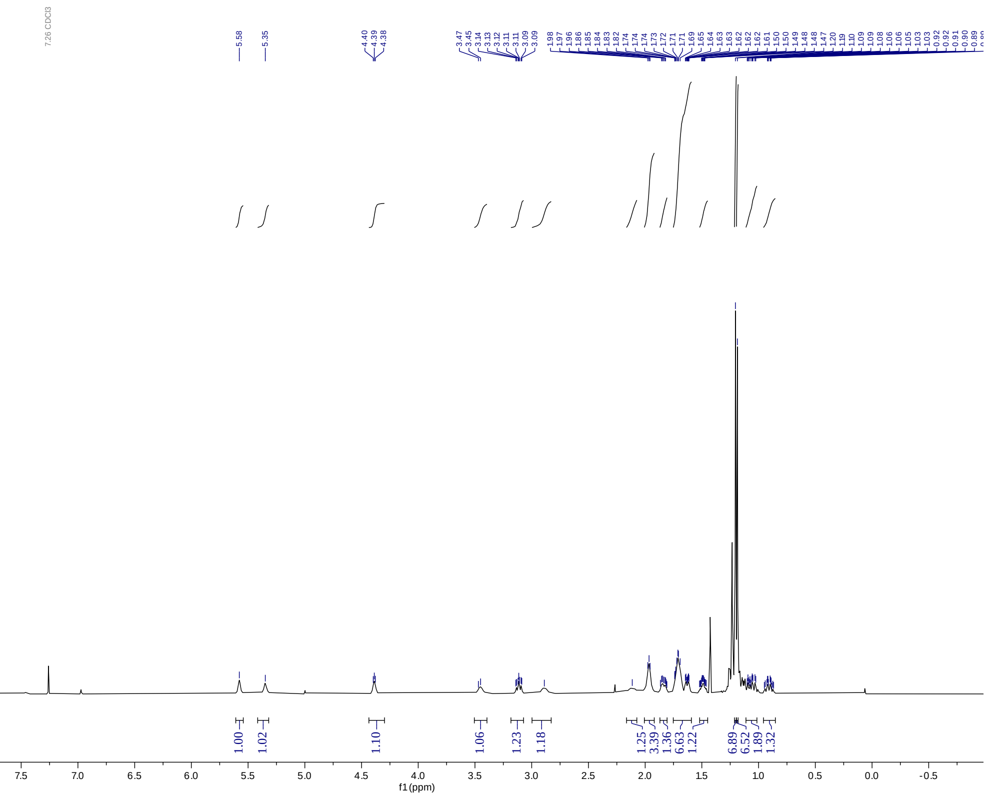

^{13}C-NMR, 126 MHz, $CDCl_3$

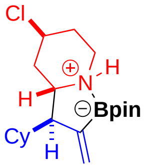

19

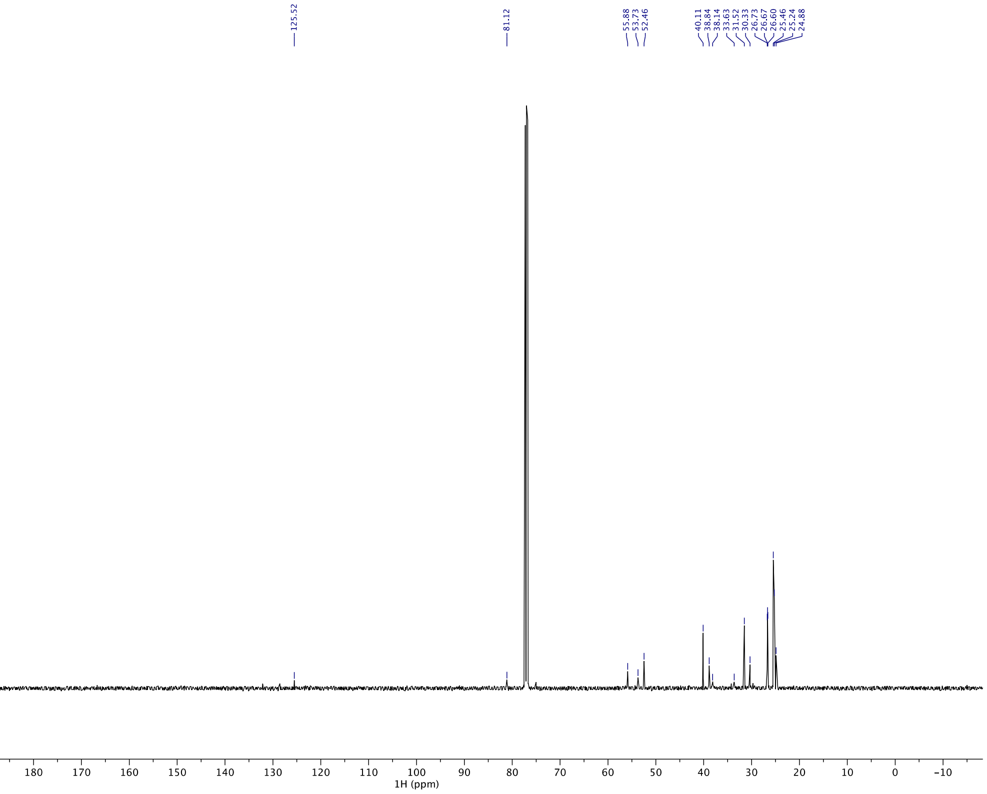

^{1}H-NMR, 500 MHz, $CDCl_3$

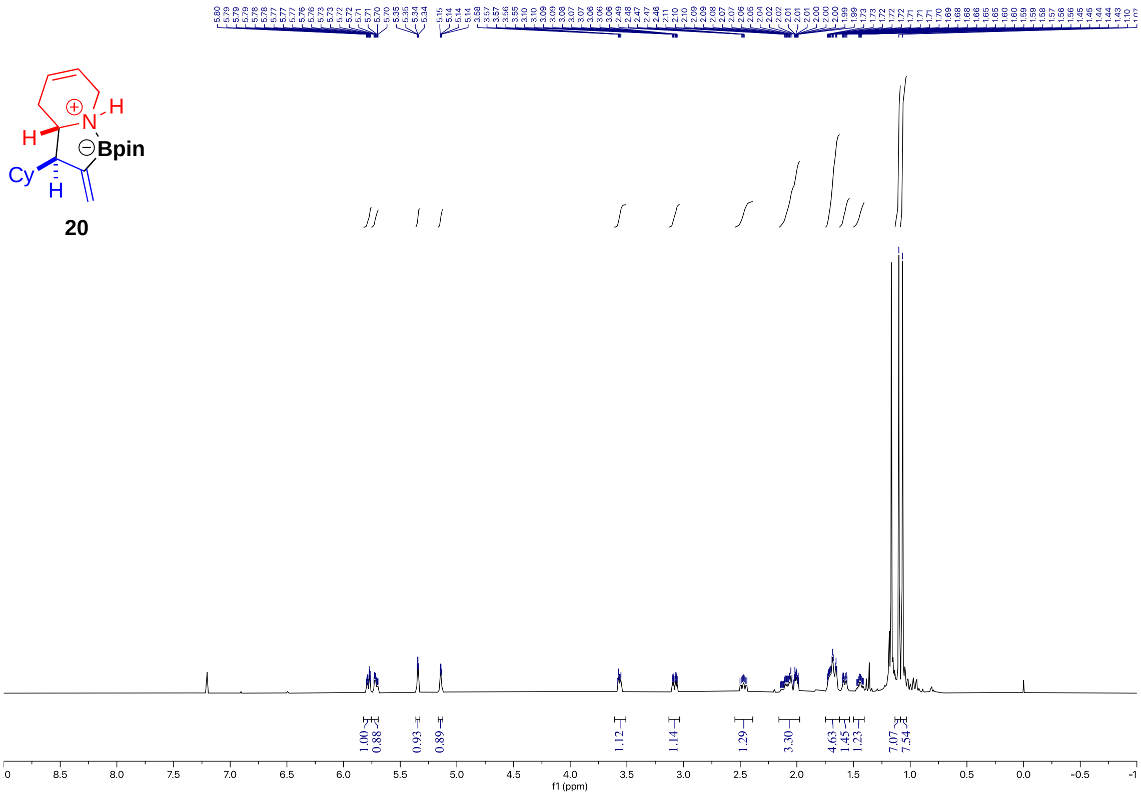

20

^{13}C-NMR, 126 MHz, $CDCl_3$

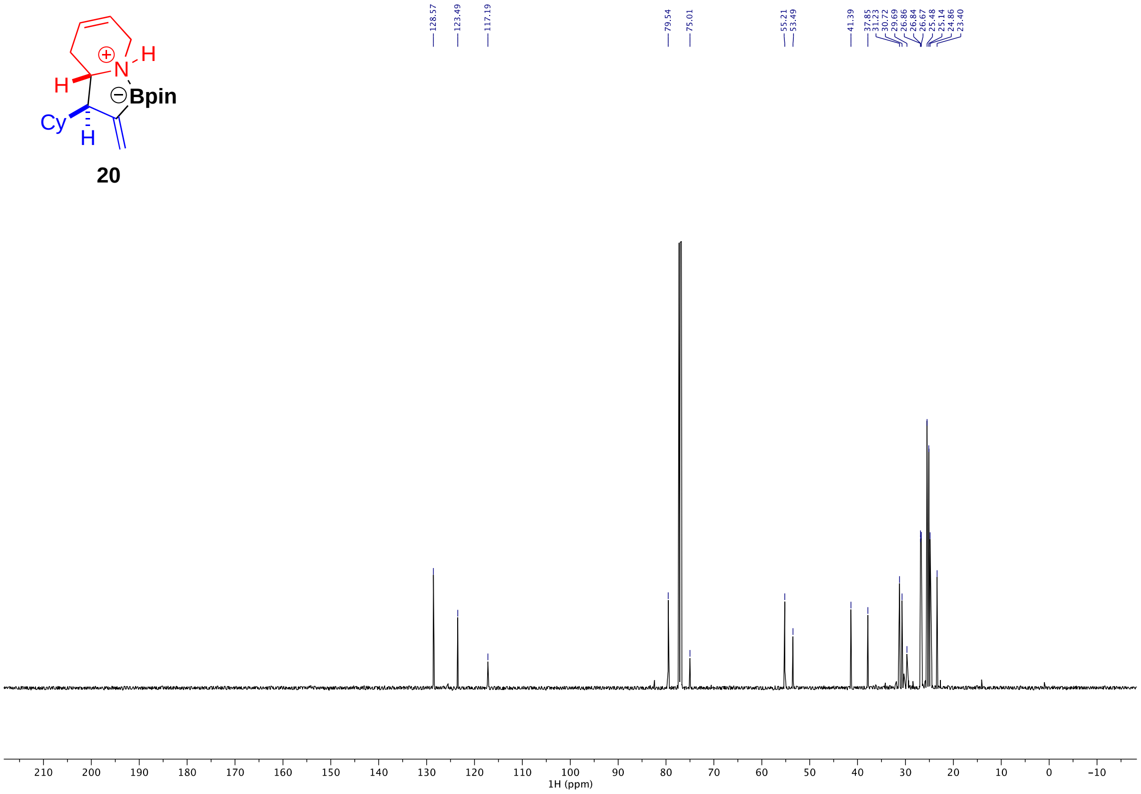

20

^{1}H-NMR, 500 MHz, $CDCl_3$

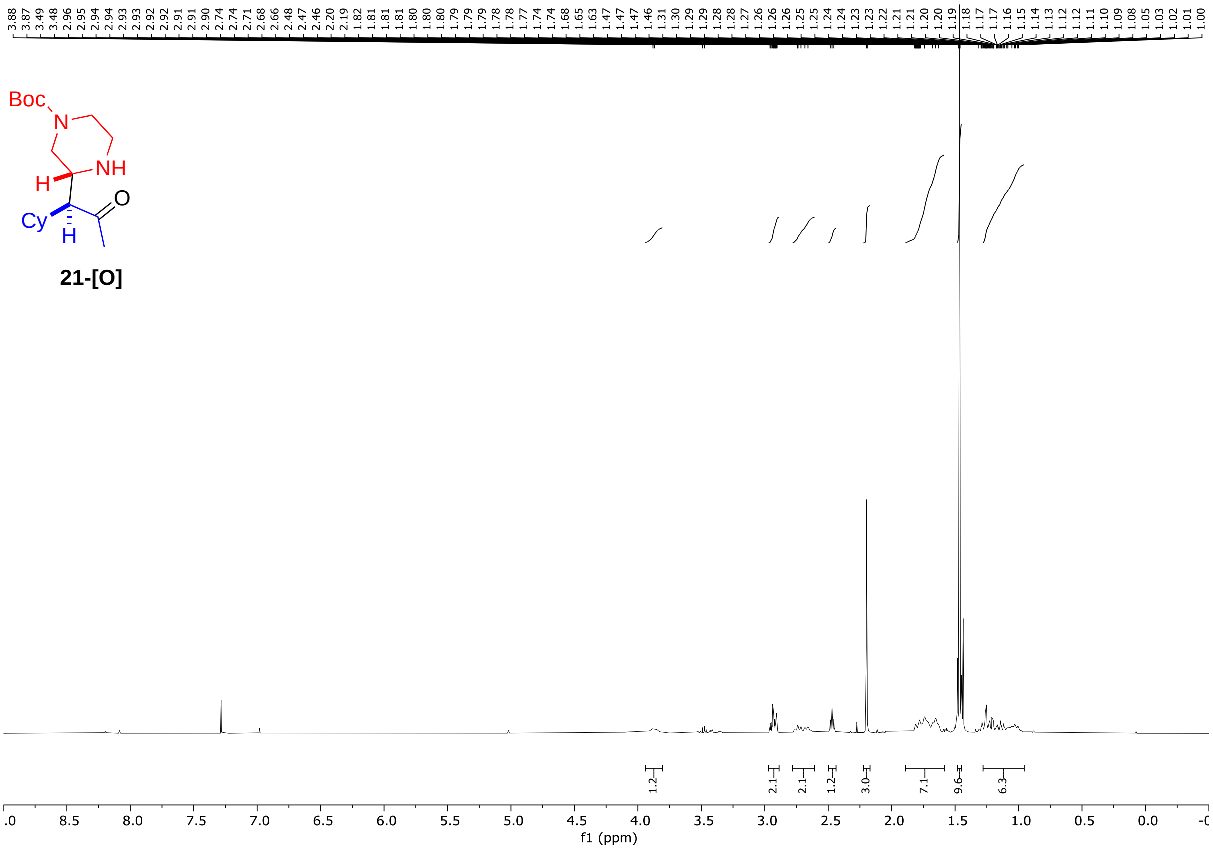

^{13}C-NMR, 75 MHz, $CDCl_3$

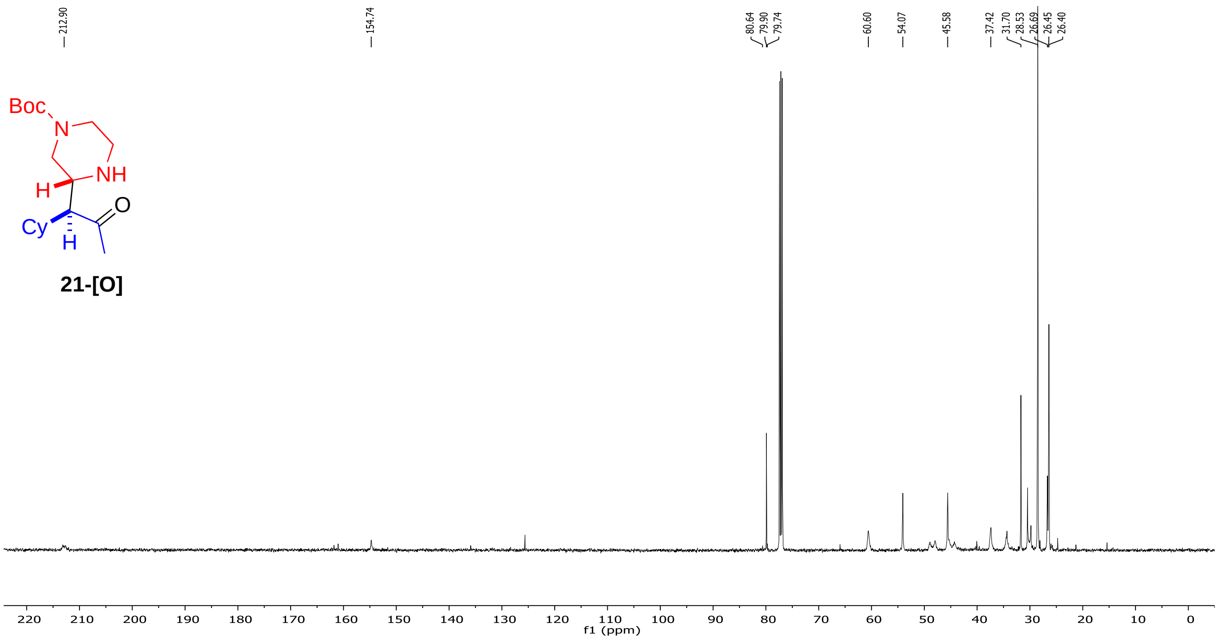

^{1}H-NMR, 500 MHz, $CDCl_3$

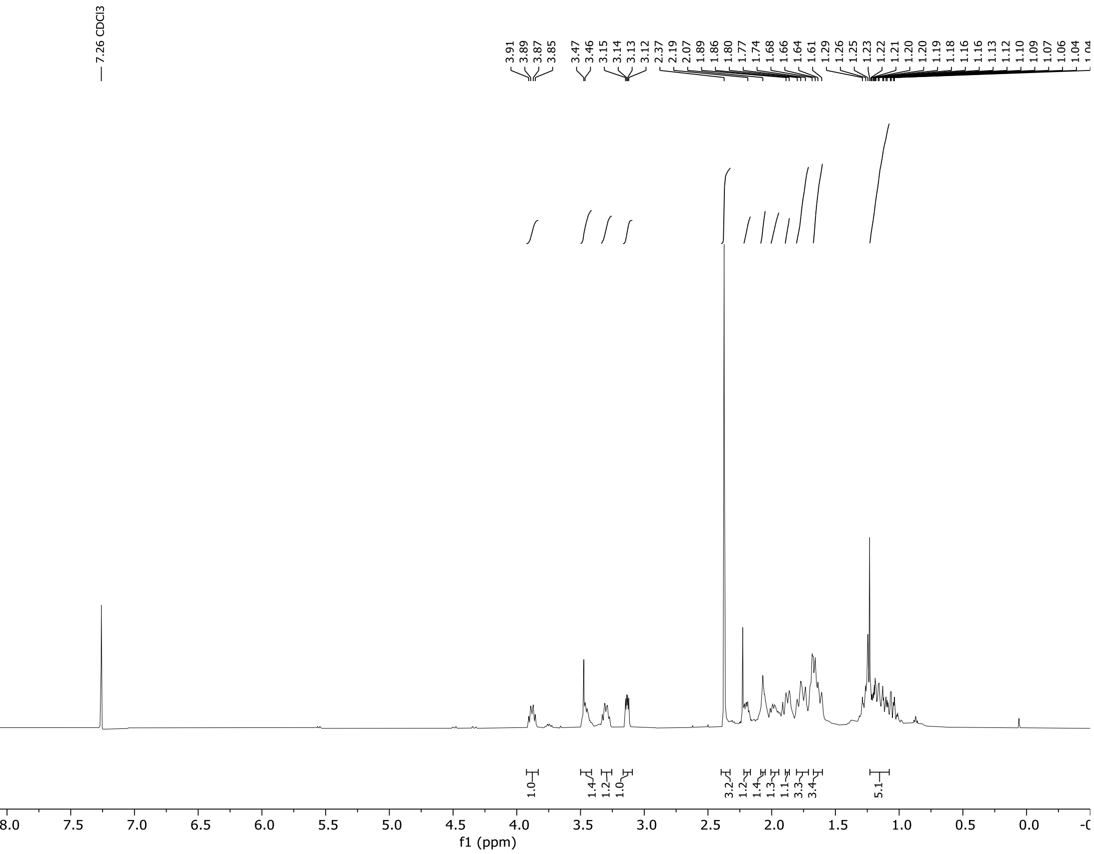

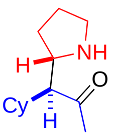

22-[O]

^{13}C-NMR, 75 MHz, $CDCl_3$

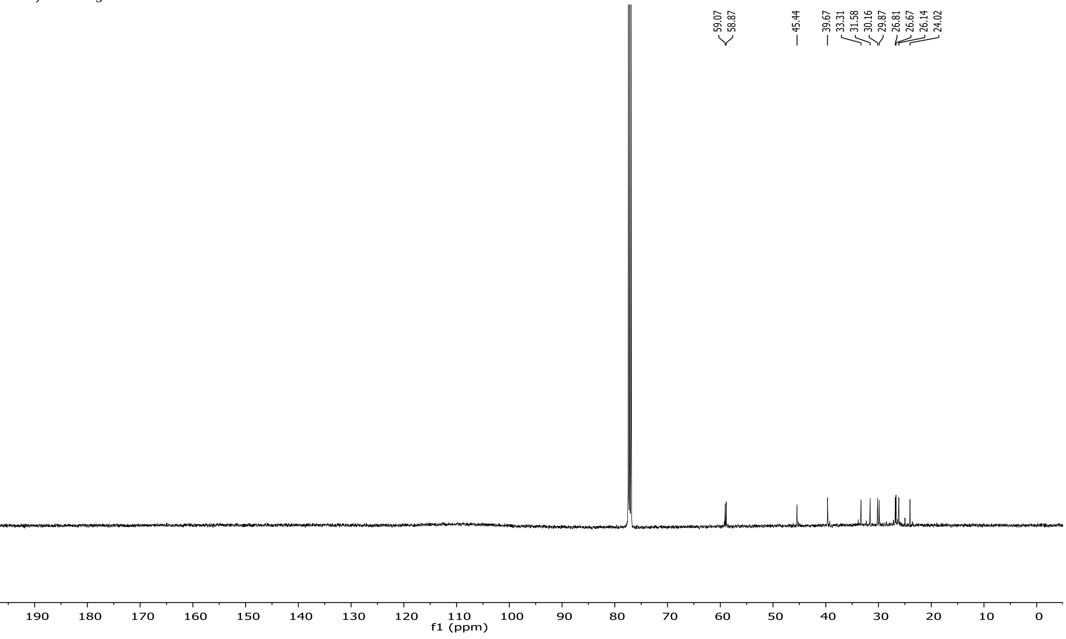

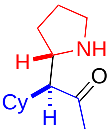

22-[O]

^{1}H-NMR, 500 MHz, $CDCl_3$

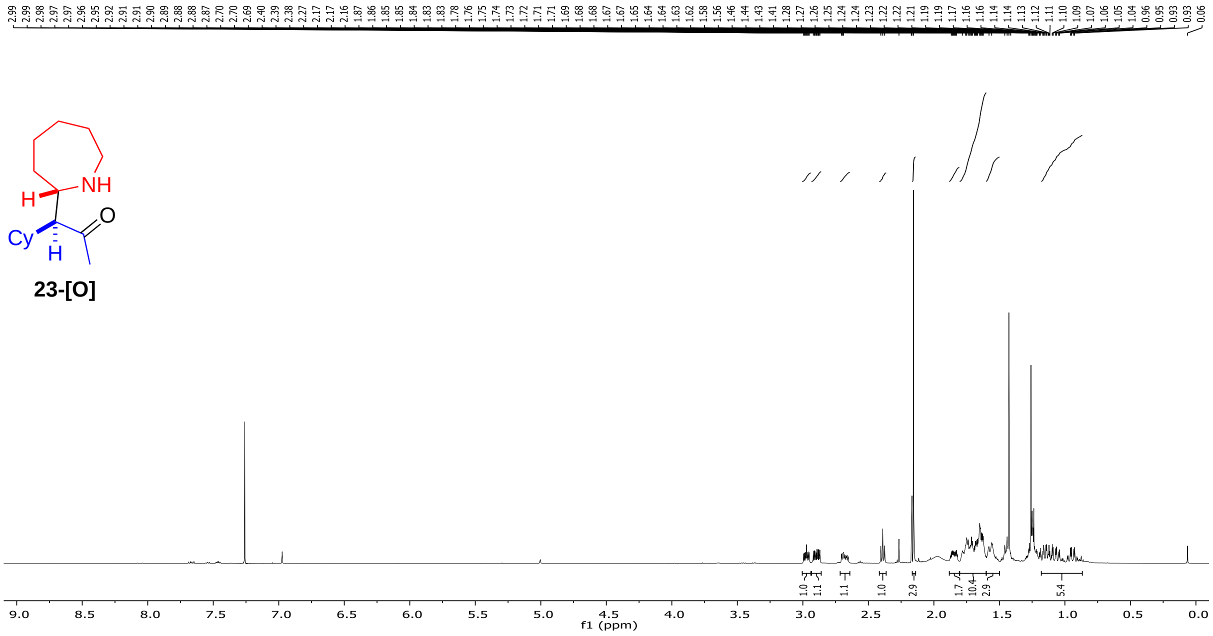

^{13}C-NMR, 126 MHz, $CDCl_3$

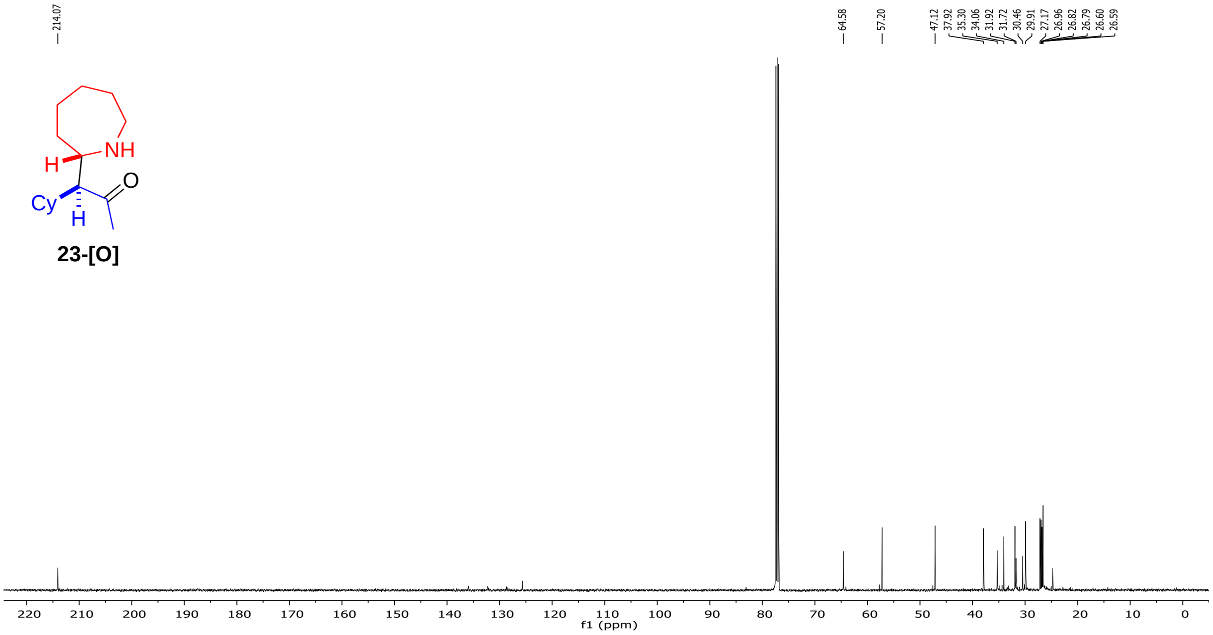

^{1}H-NMR, 500 MHz, $CDCl_3$

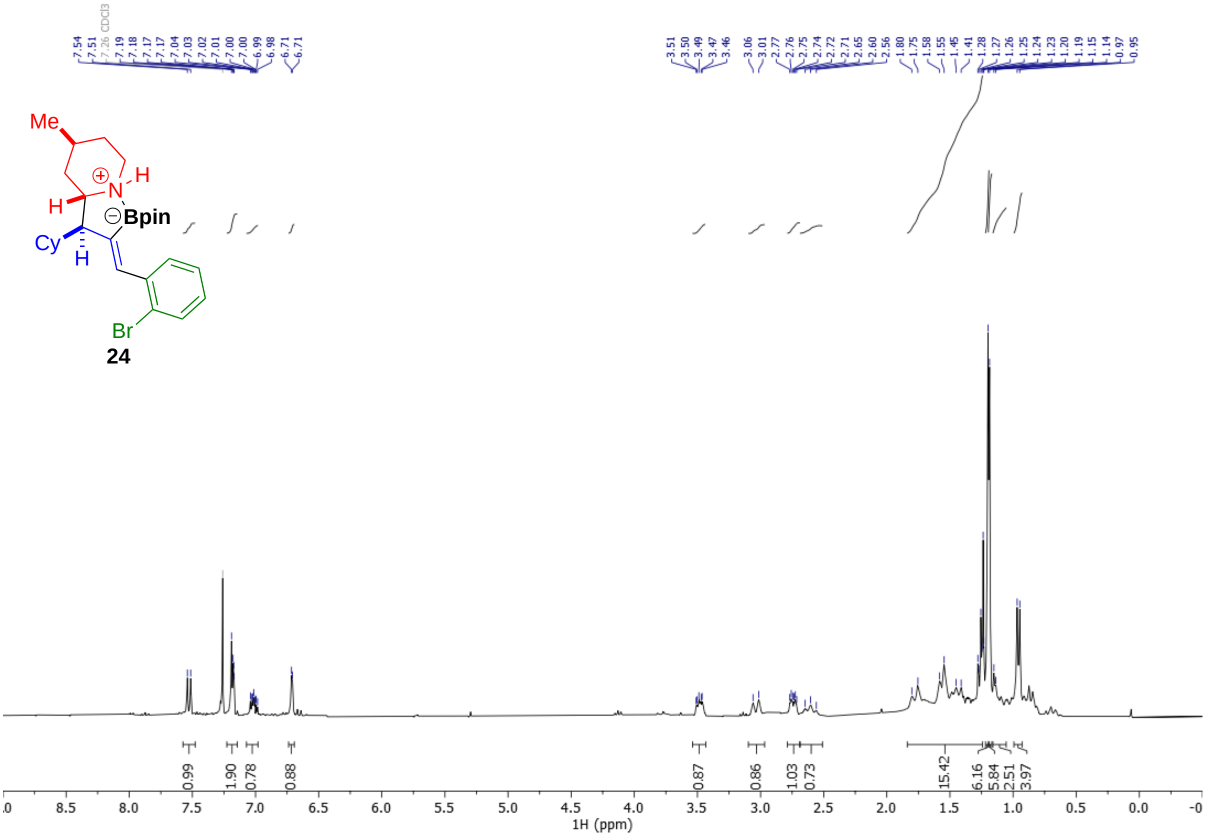

^{13}C-NMR, 126 MHz, $CDCl_3$

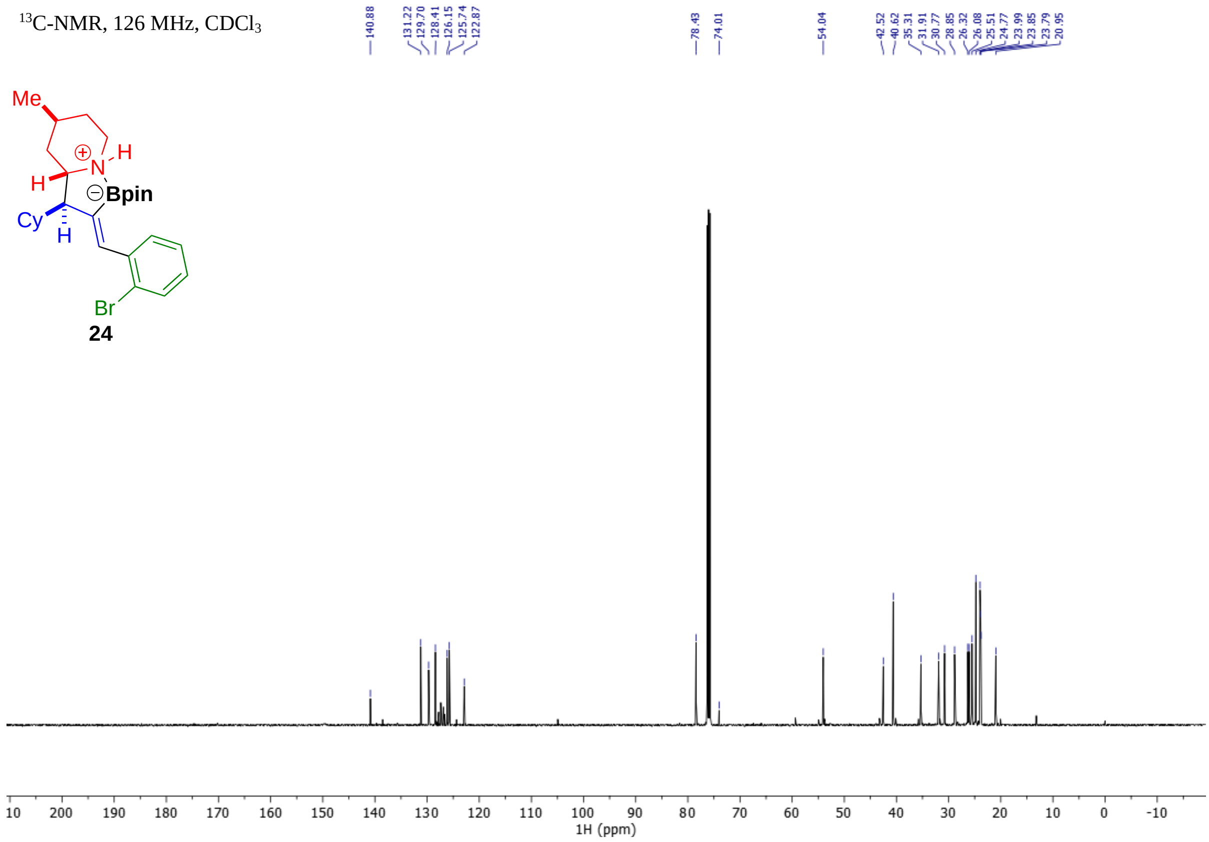

^{1}H-NMR, 500 MHz, $CDCl_3$

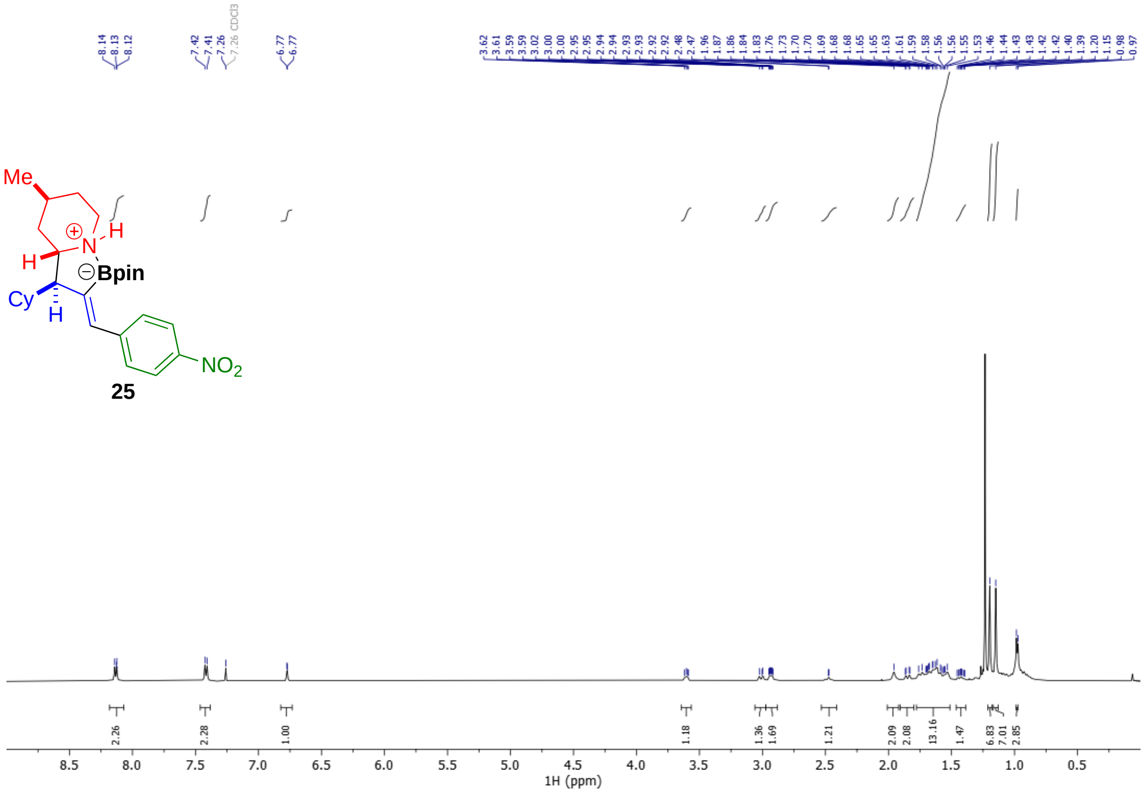

^{13}C-NMR, 126 MHz, $CDCl_3$

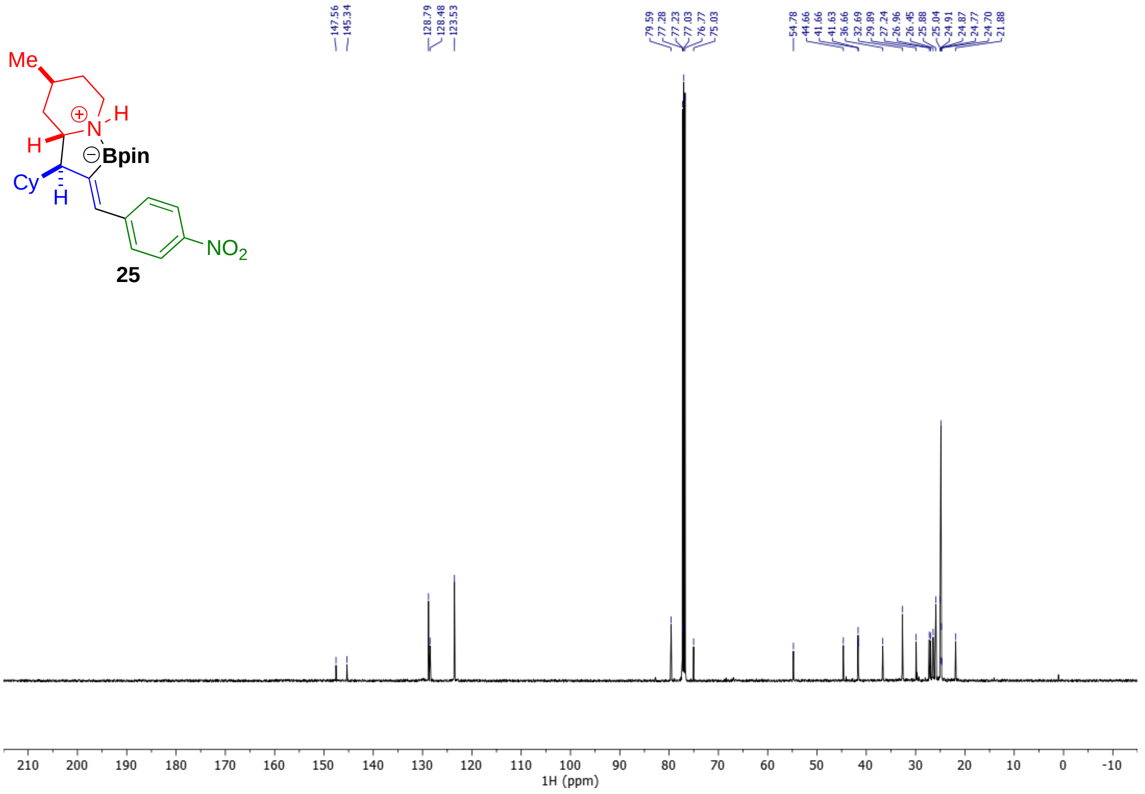

^{1}H-NMR, 500 MHz, $CDCl_3$

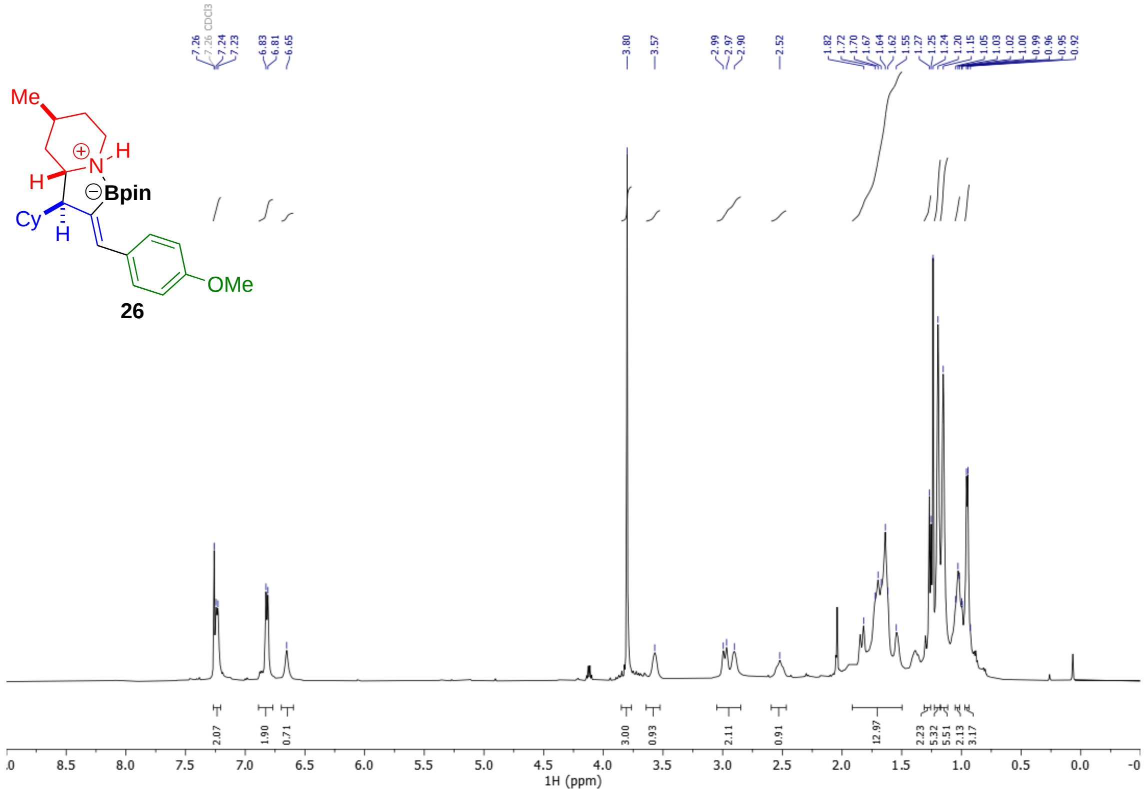

^{13}C-NMR, 126 MHz, $CDCl_3$

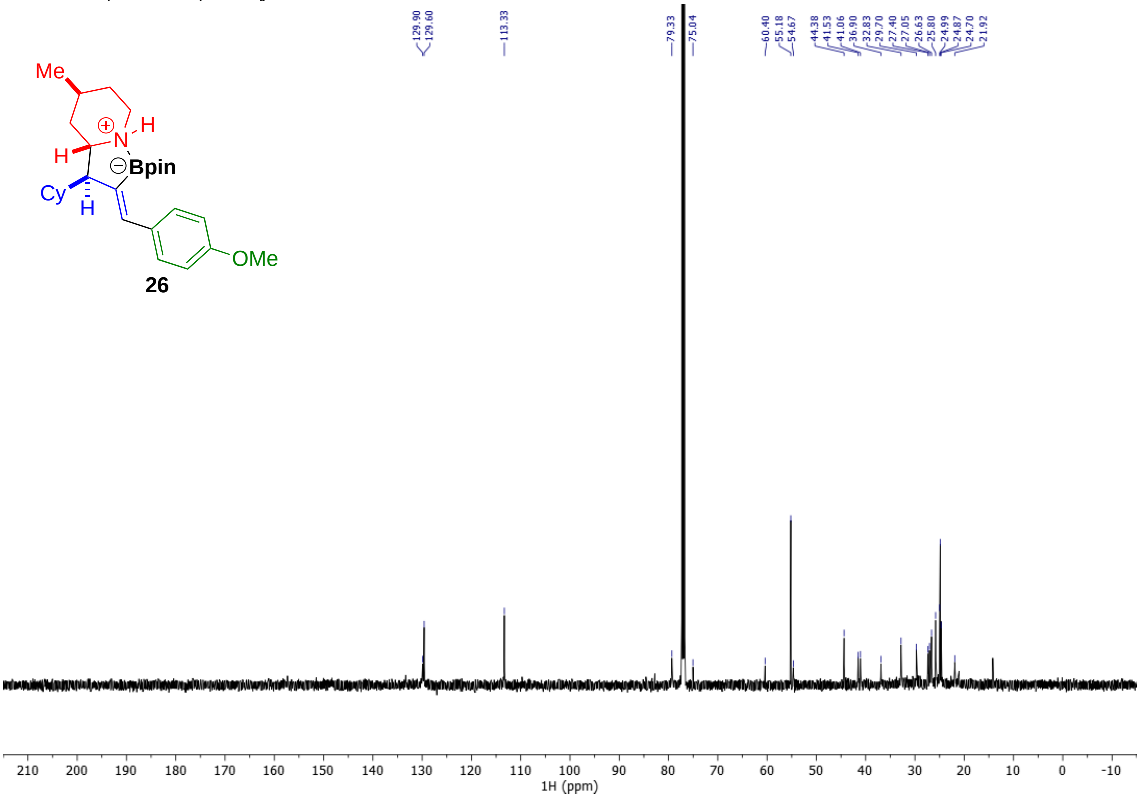

^{1}H-NMR, 300 MHz, $CDCl_3$

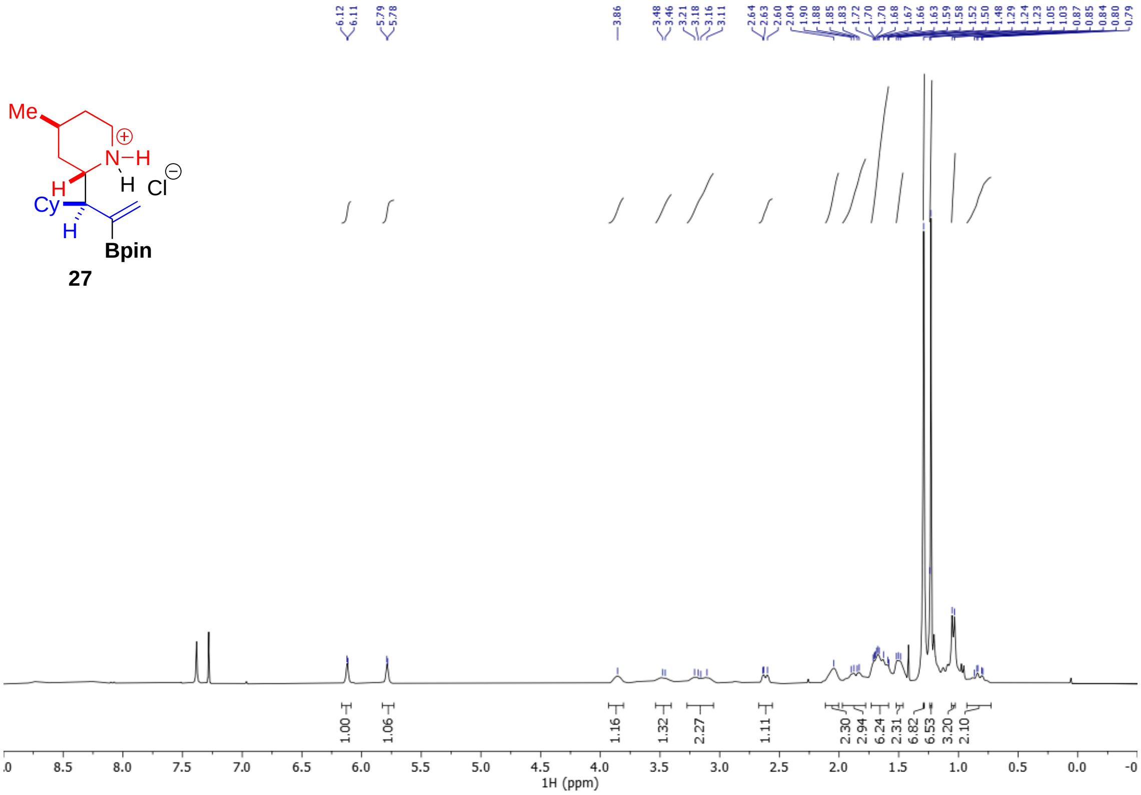

27

^{13}C-NMR, 126 MHz, $CDCl_3$

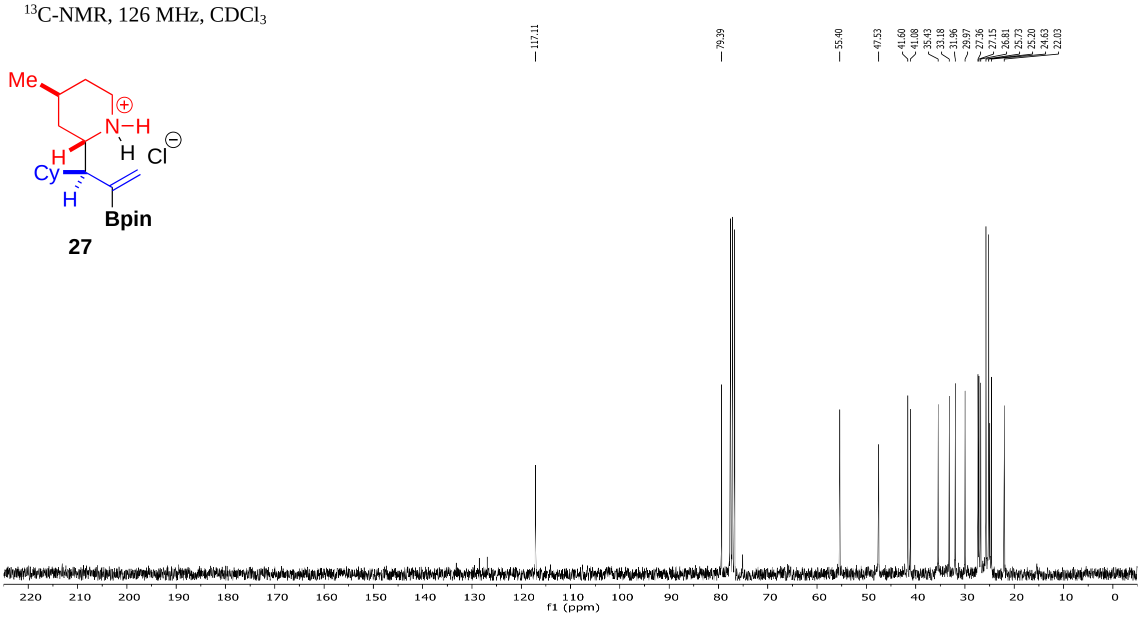

27

^{1}H-NMR, 300 MHz, $CDCl_3$

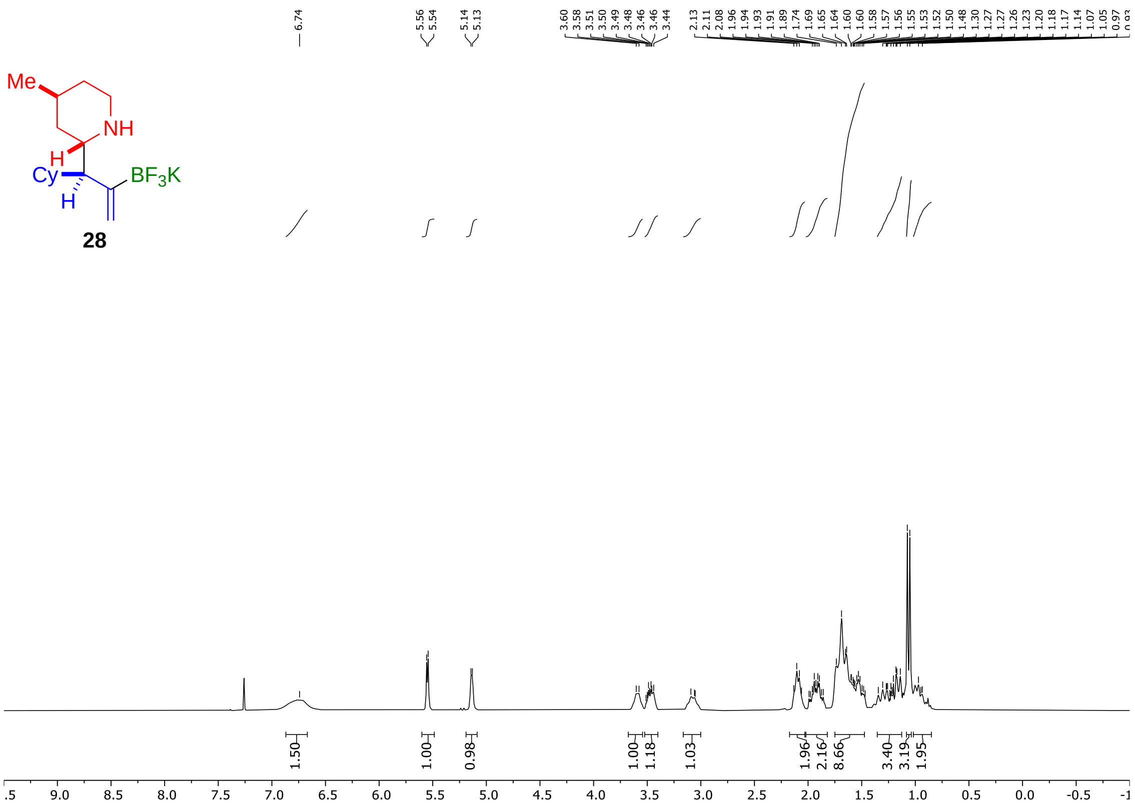

^{13}C-NMR, 126 MHz, $CDCl_3$

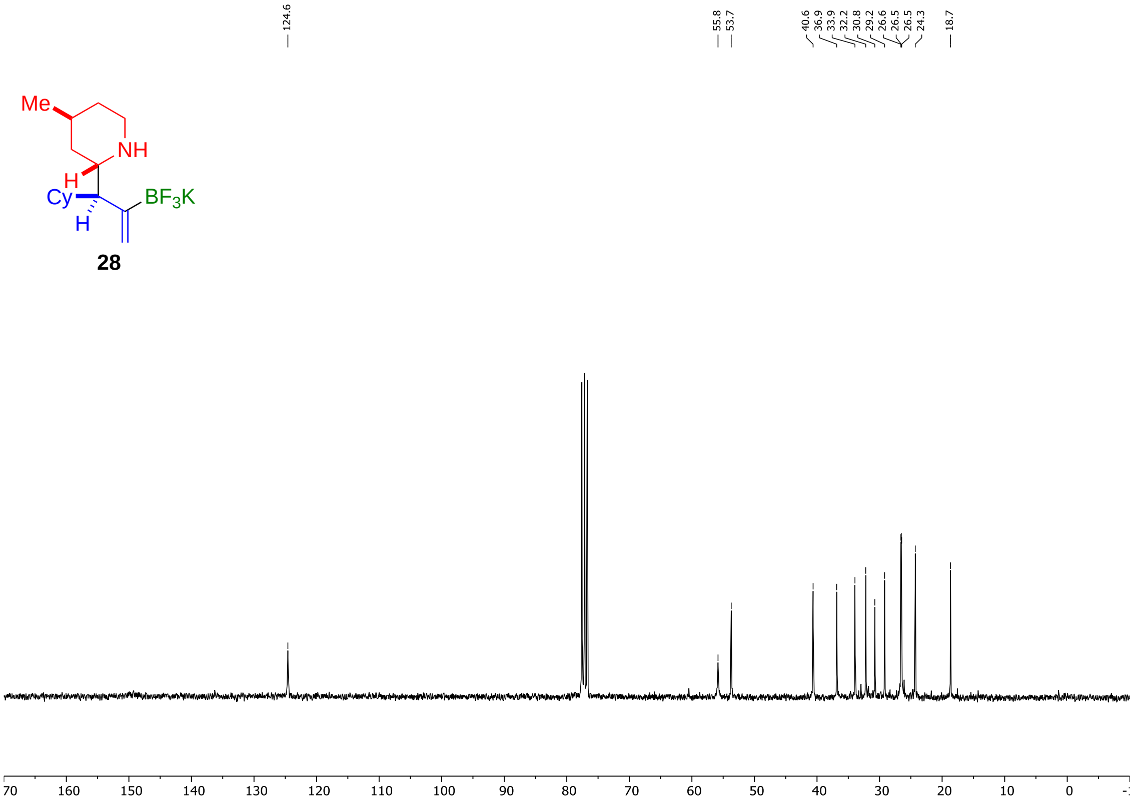

^{19}F-NMR, 282 MHz, $CDCl_3$

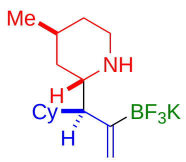

28

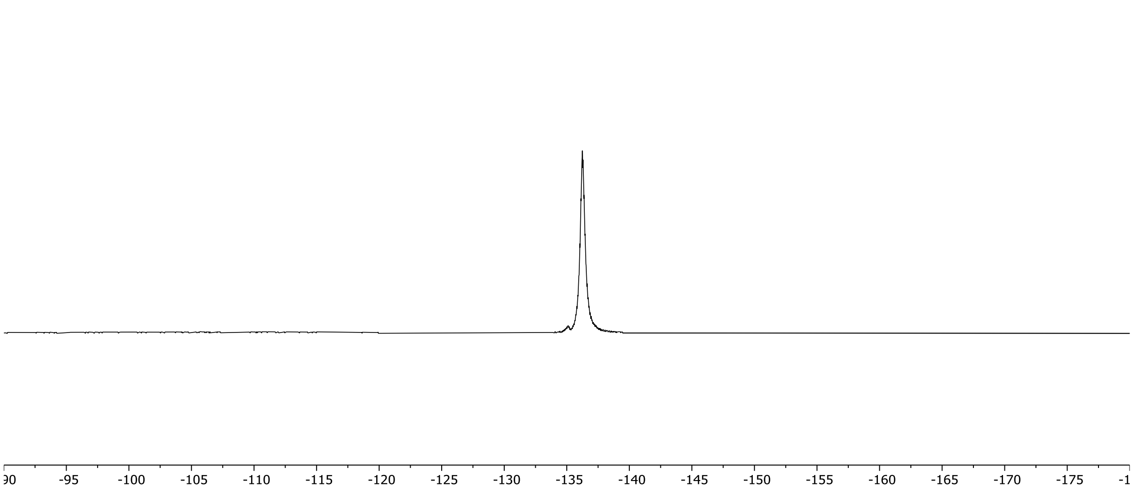

^{1}H-NMR, 500 MHz, $CDCl_3$

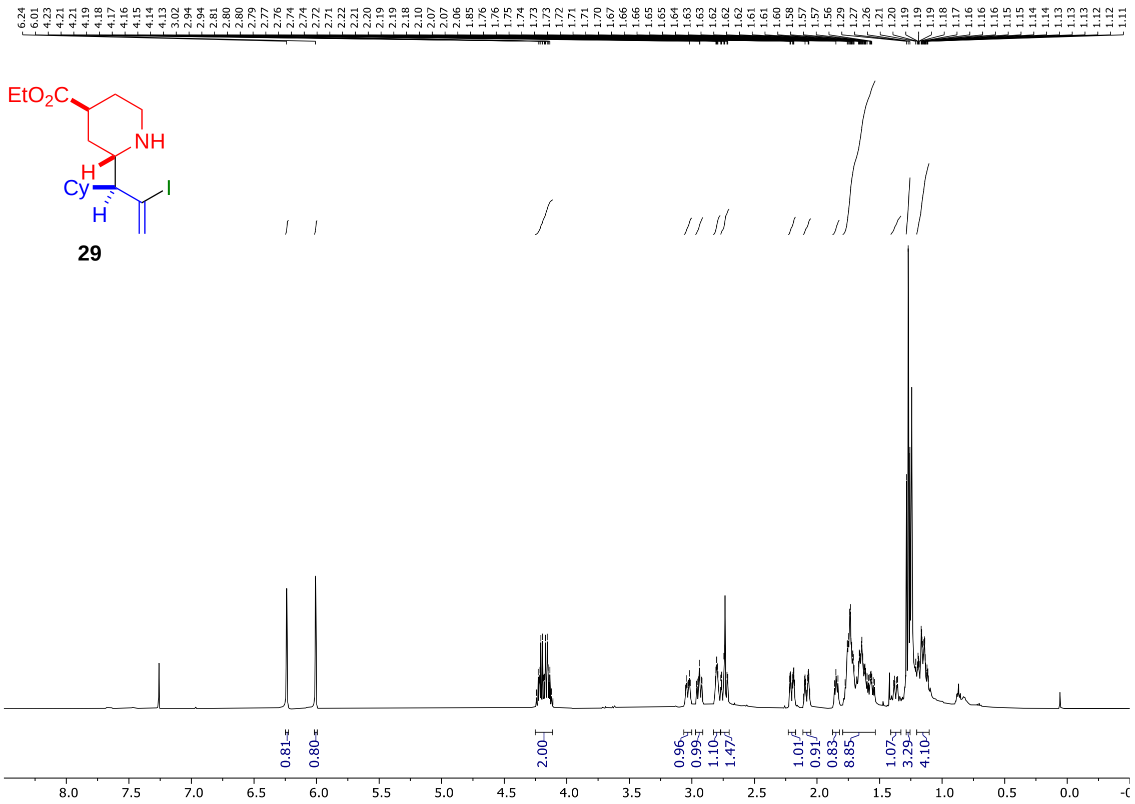

^{13}C-NMR, 126 MHz, $CDCl_3$

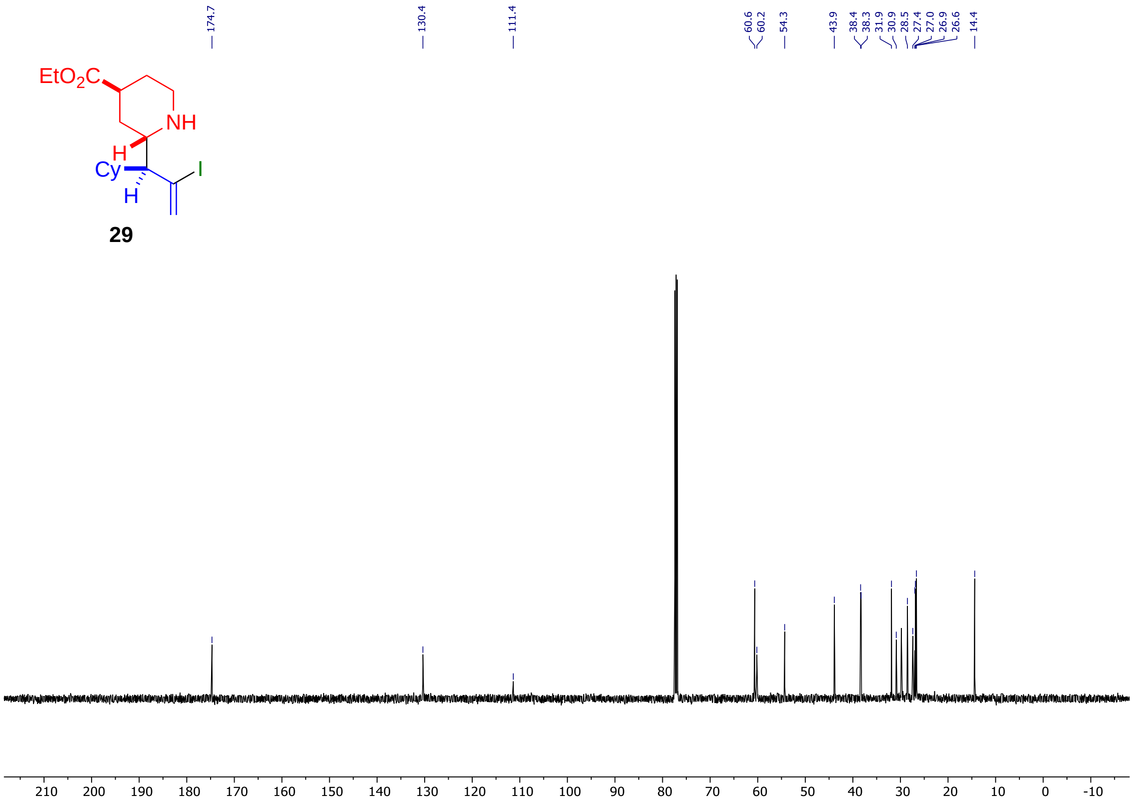

^{1}H-NMR, 500 MHz, $CDCl_3$

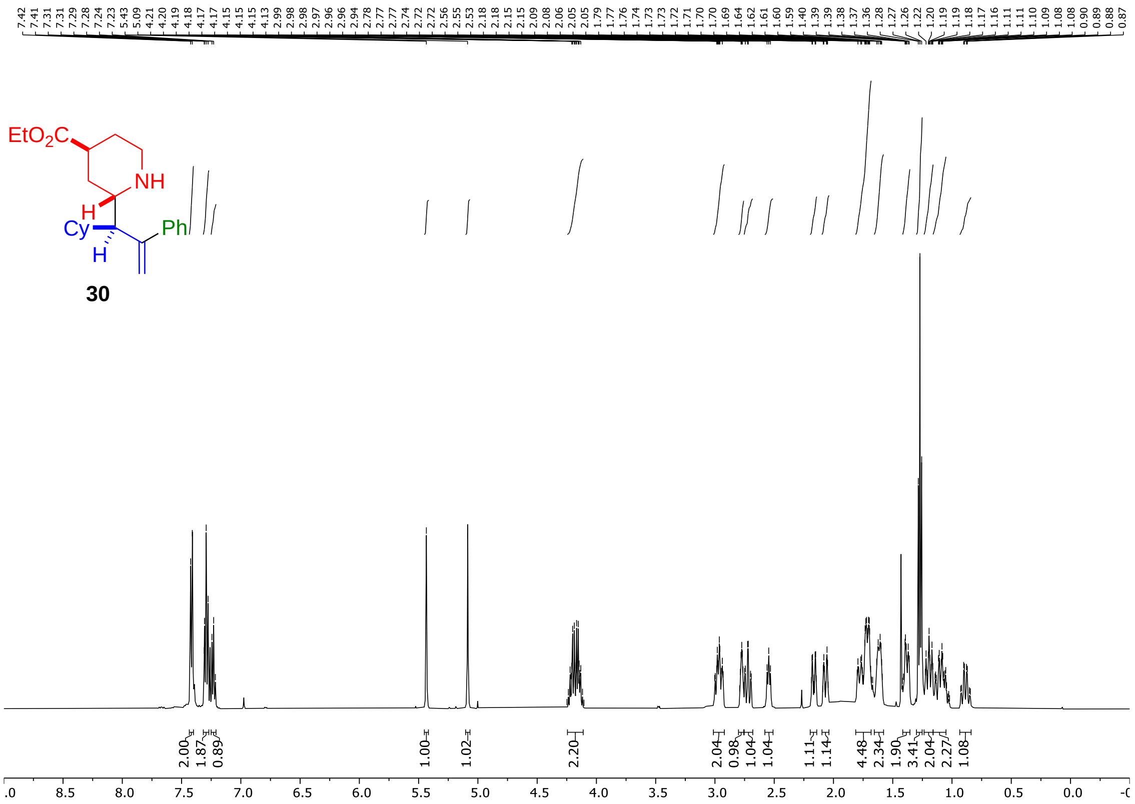

^{13}C-NMR, 126 MHz, $CDCl_3$

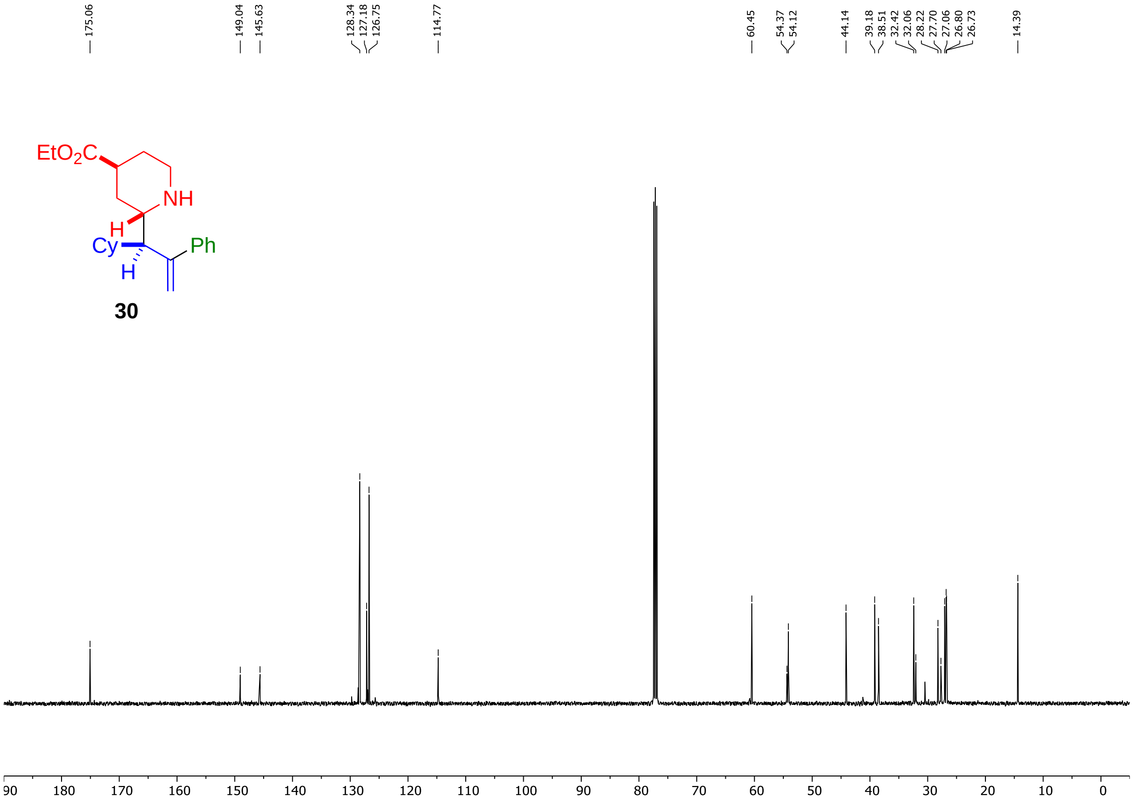

^{1}H-NMR, 300 MHz, $CDCl_3$

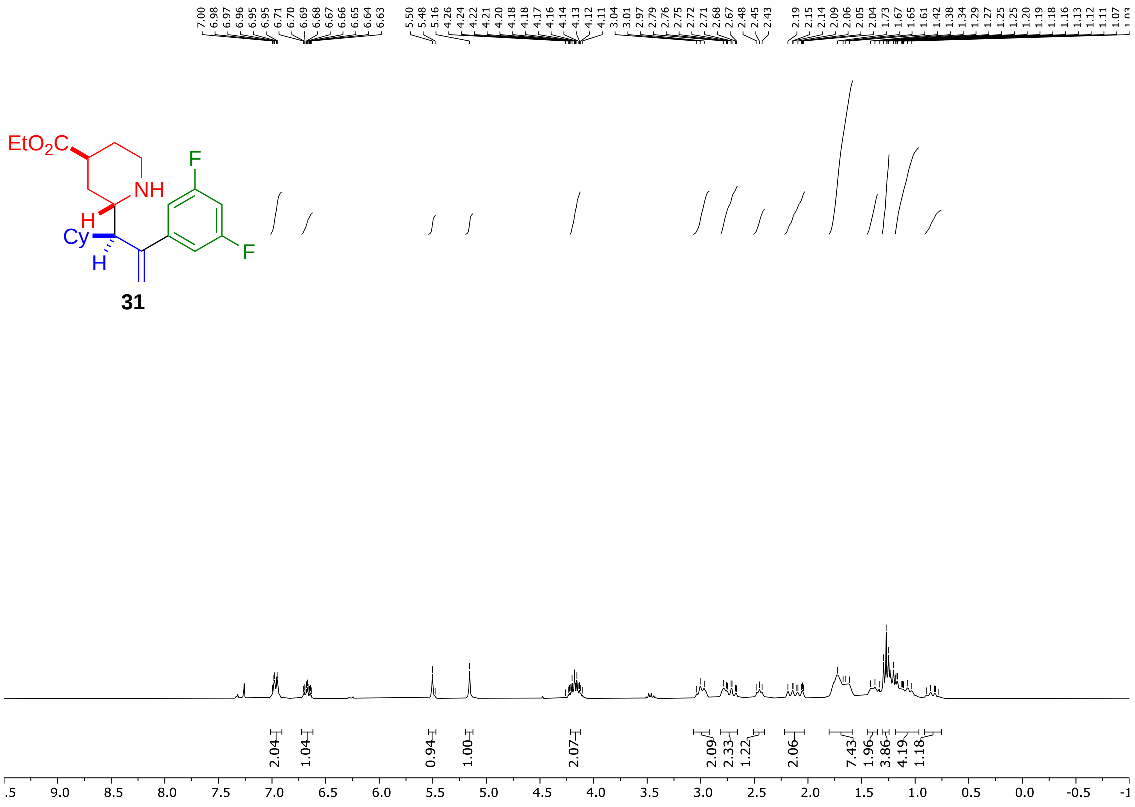

^{13}C-NMR, 126 MHz, $CDCl_3$

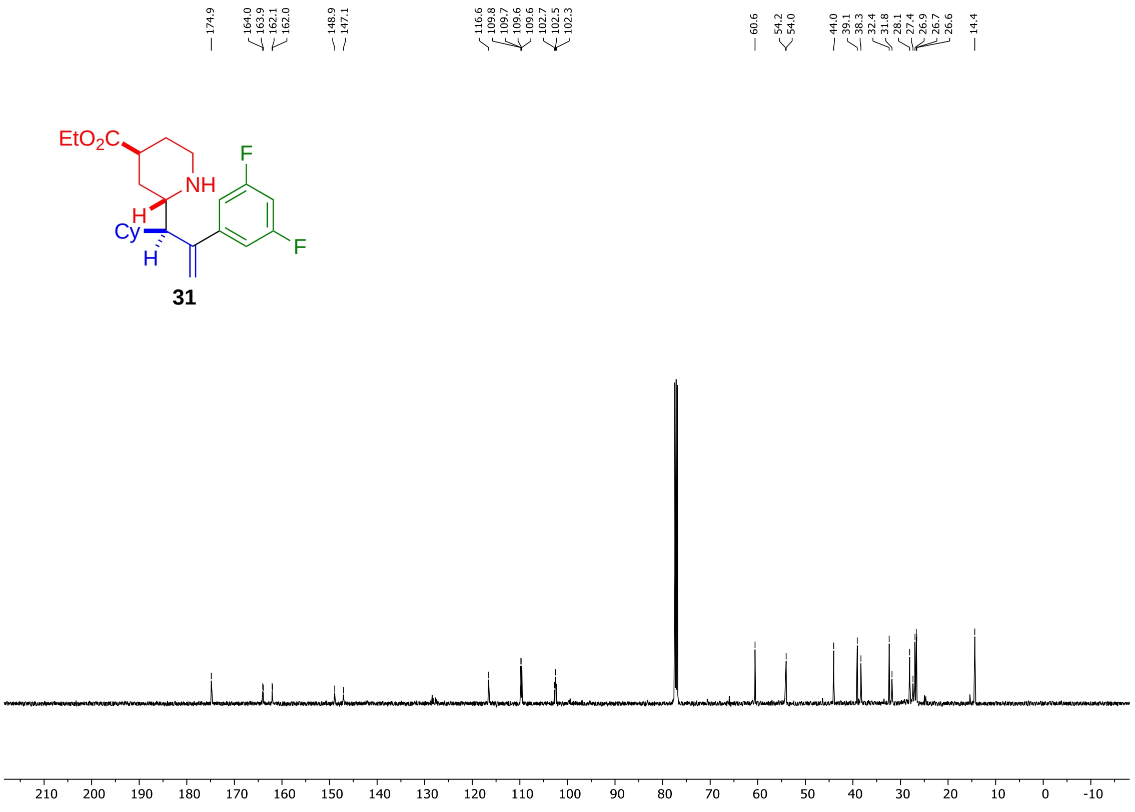

^{19}F-NMR, 282 MHz, $CDCl_3$

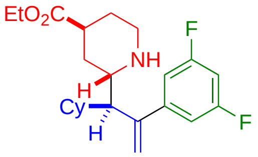

31

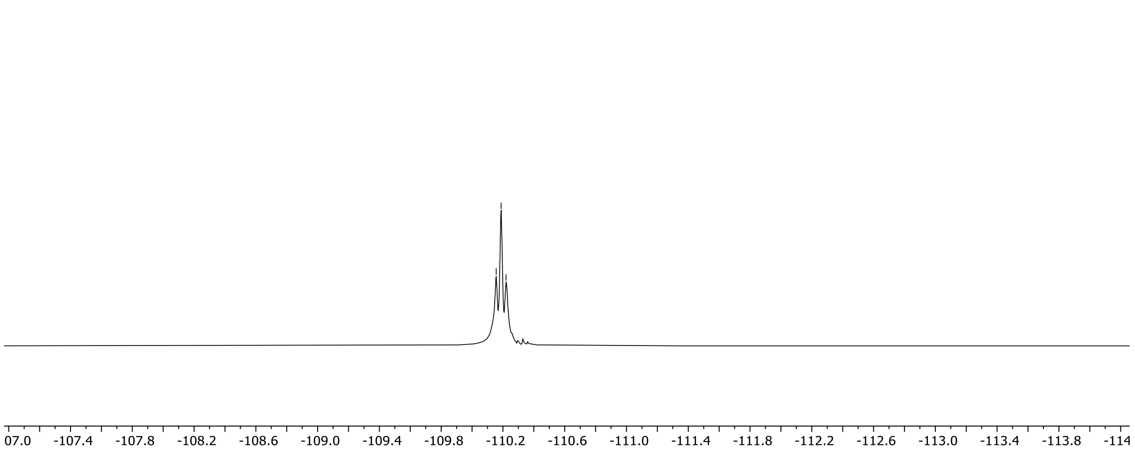

^{1}H-NMR, 500 MHz, $CDCl_3$

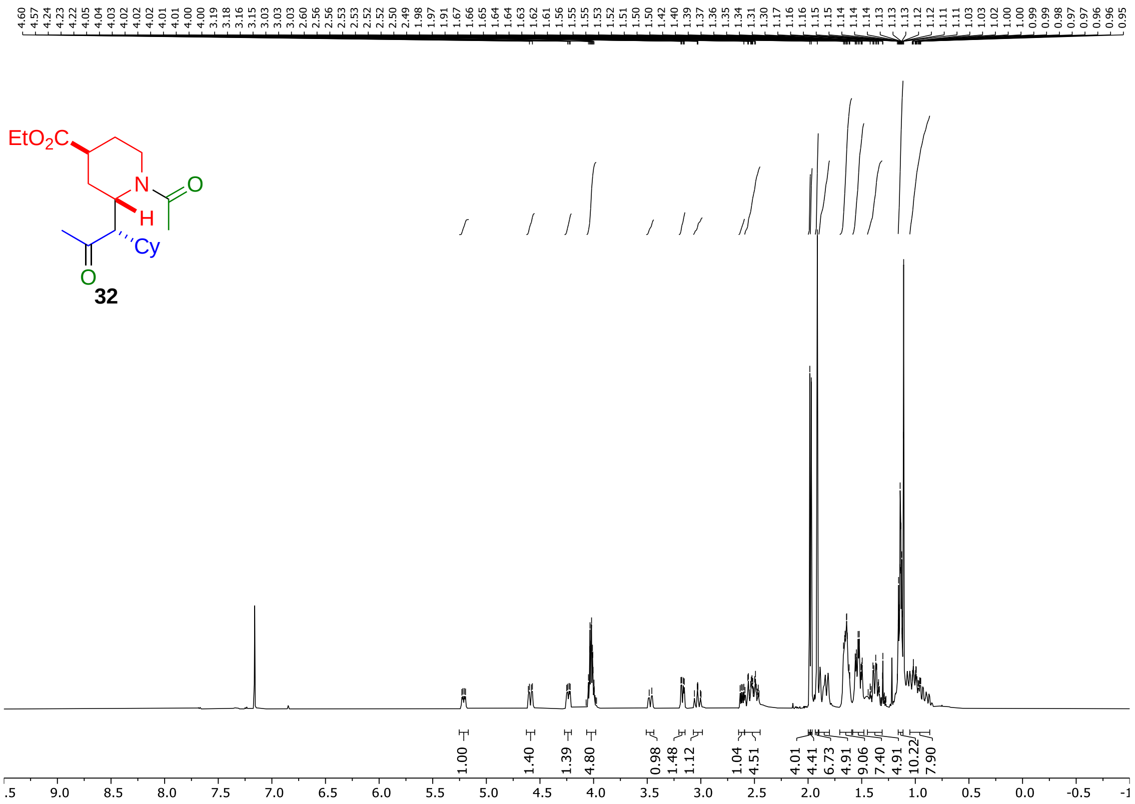

^{13}C-NMR, 126 MHz, $CDCl_3$

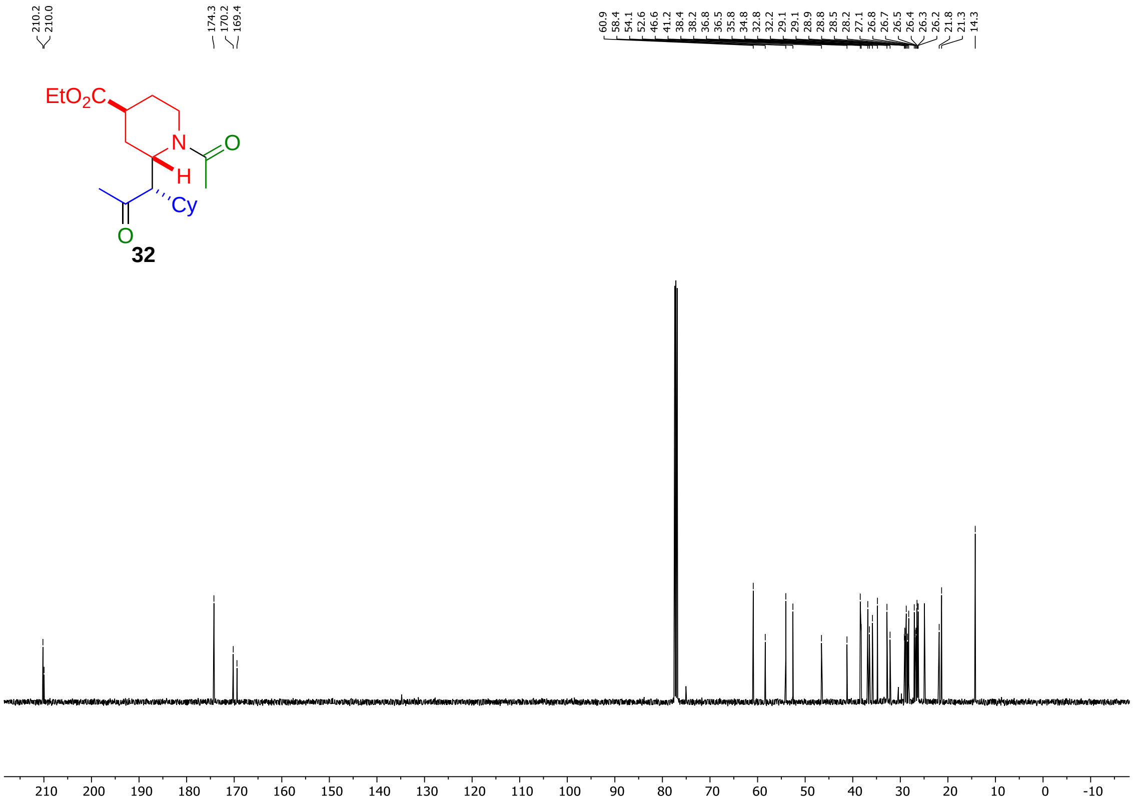

^{1}H-NMR, 500 MHz, $CDCl_3$

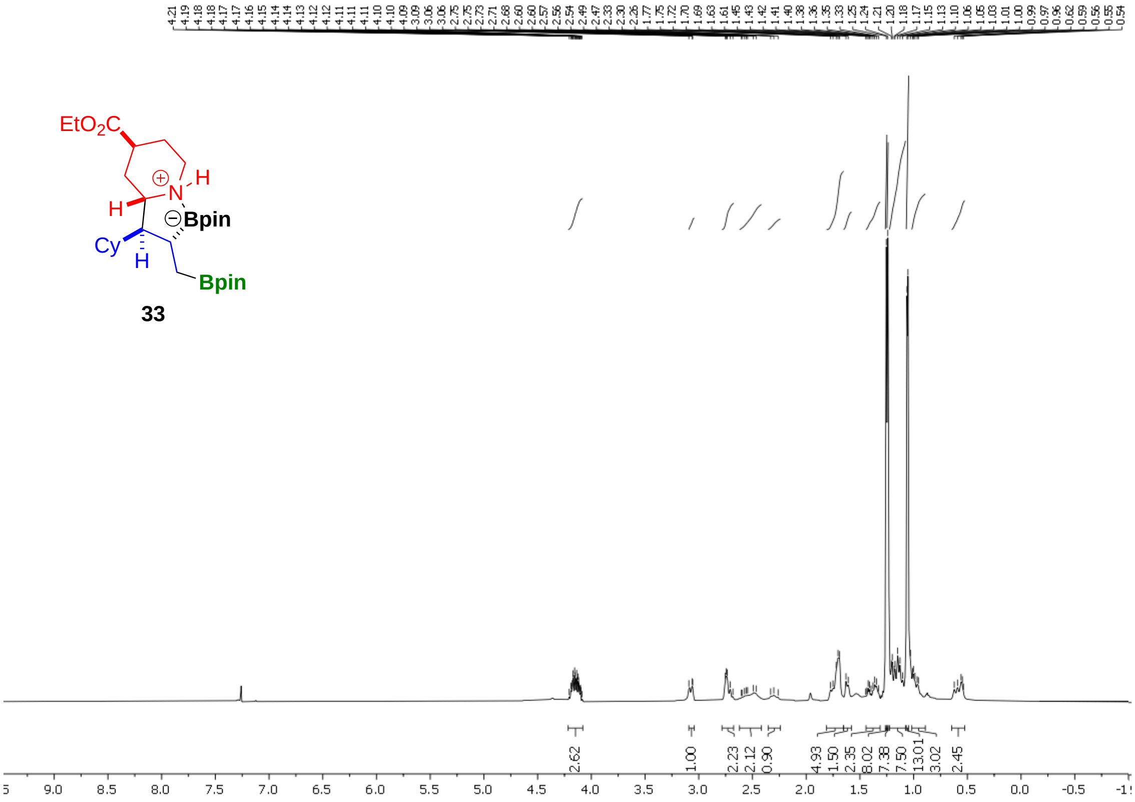

^{13}C-NMR, 126 MHz, $CDCl_3$

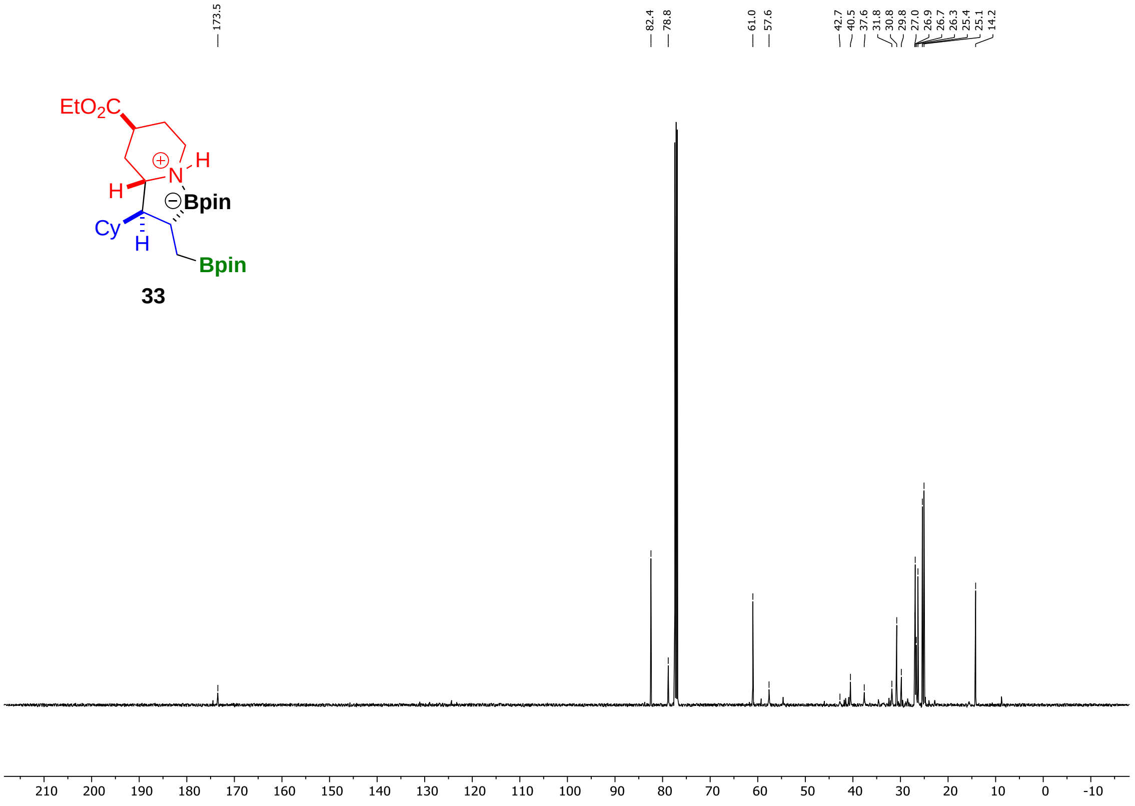

^{1}H-NMR, 500 MHz, $CDCl_3$

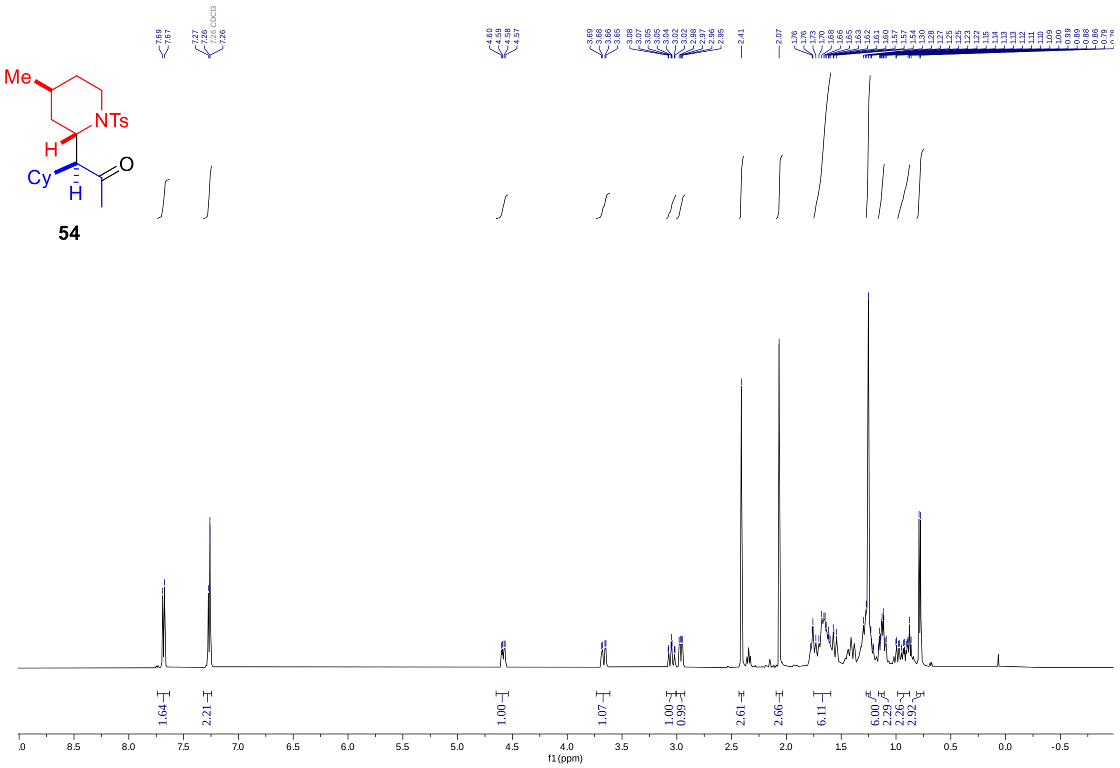

^{13}C-NMR, 126 MHz, $CDCl_3$

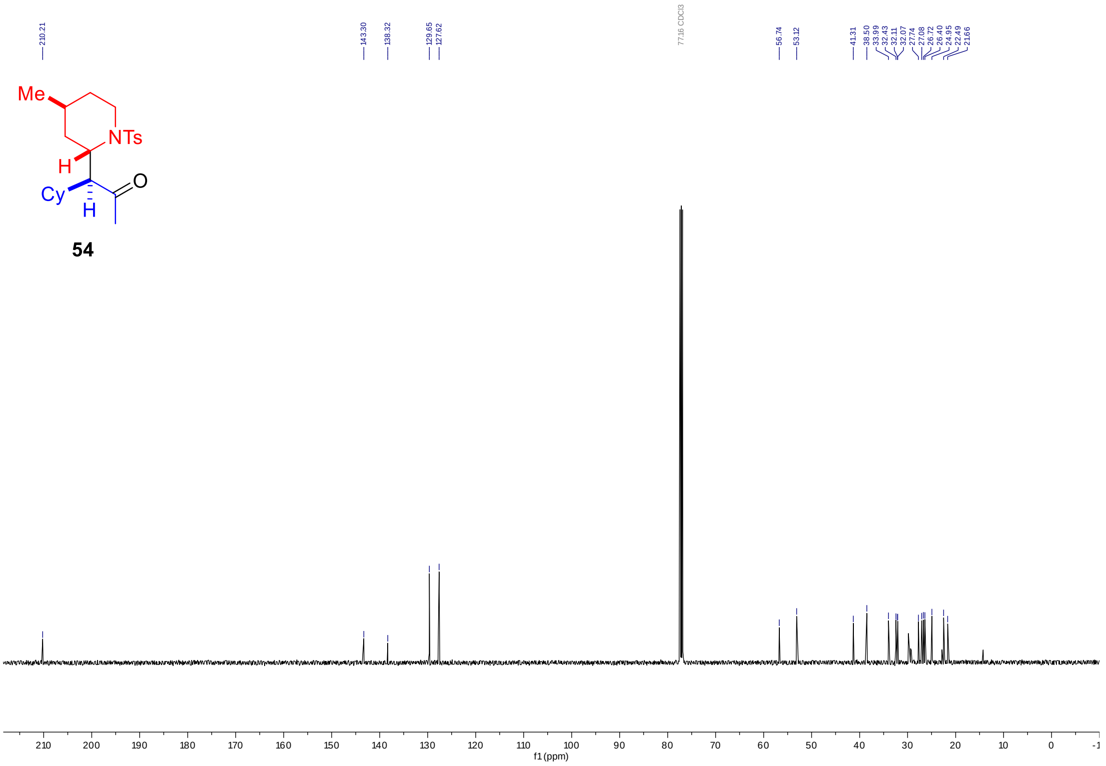

^{1}H-NMR, 500 MHz, $CDCl_3$

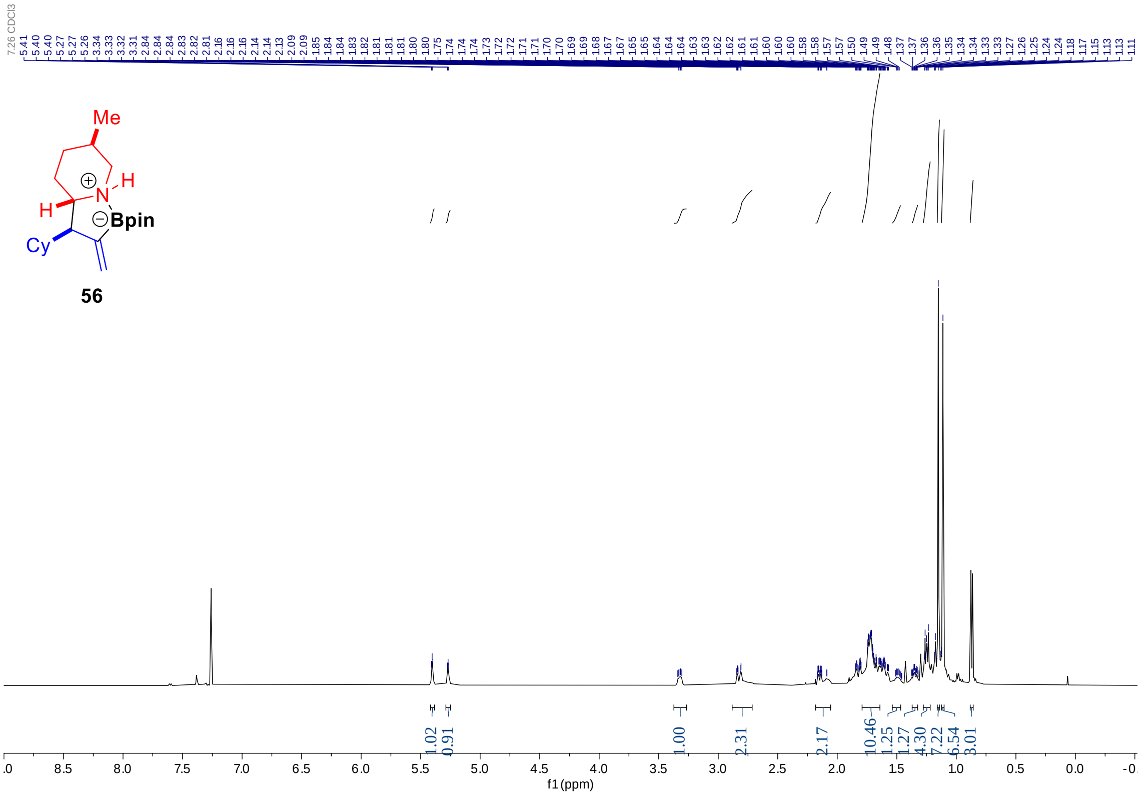

^{13}C-NMR, 126 MHz, $CDCl_3$

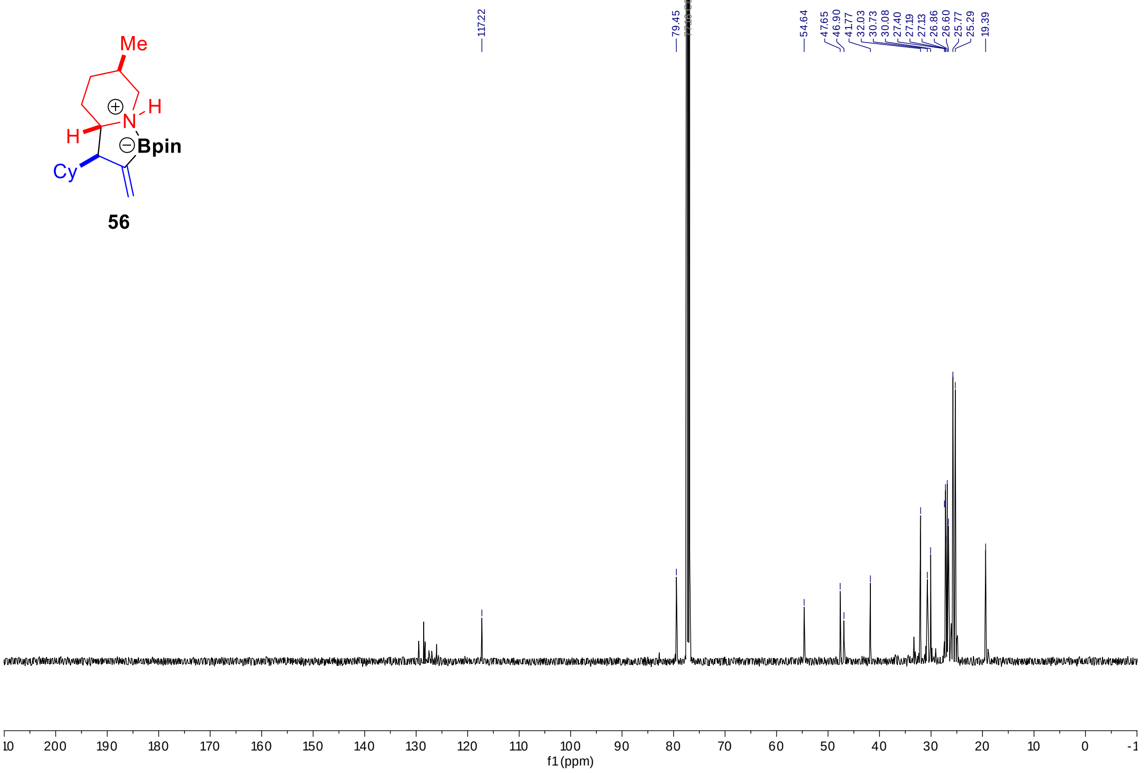

COSY, 500 MHz, $CDCl_3$

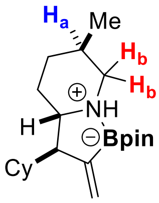

56

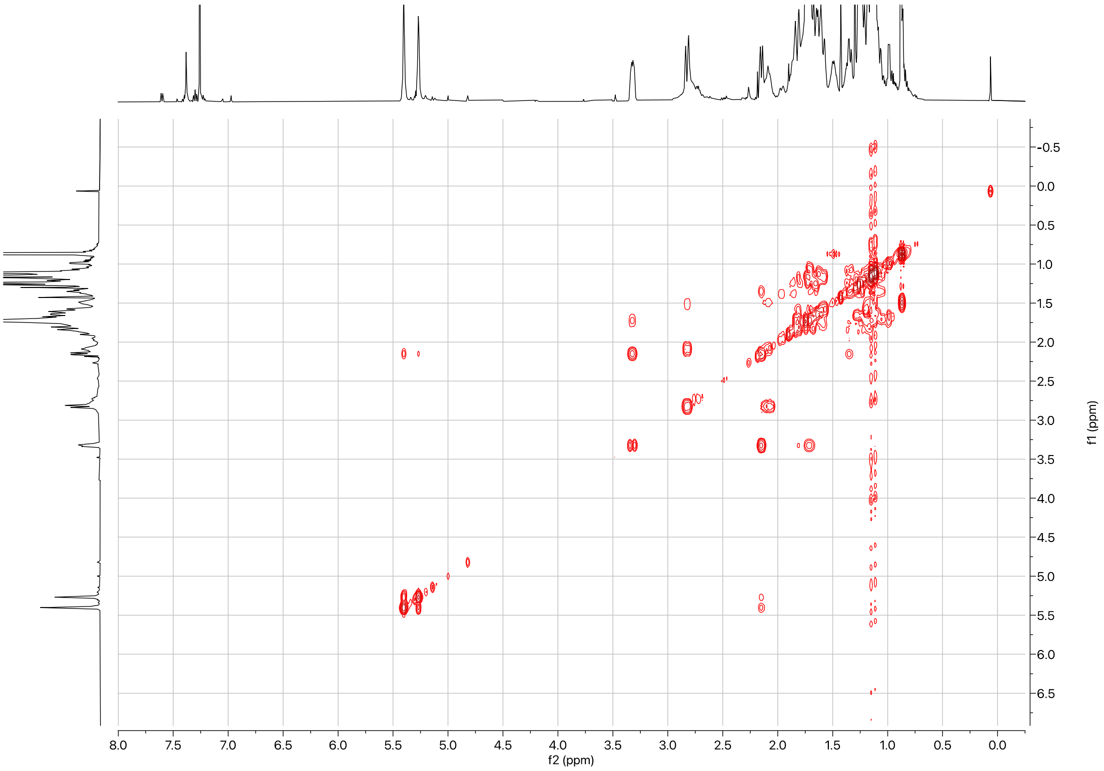

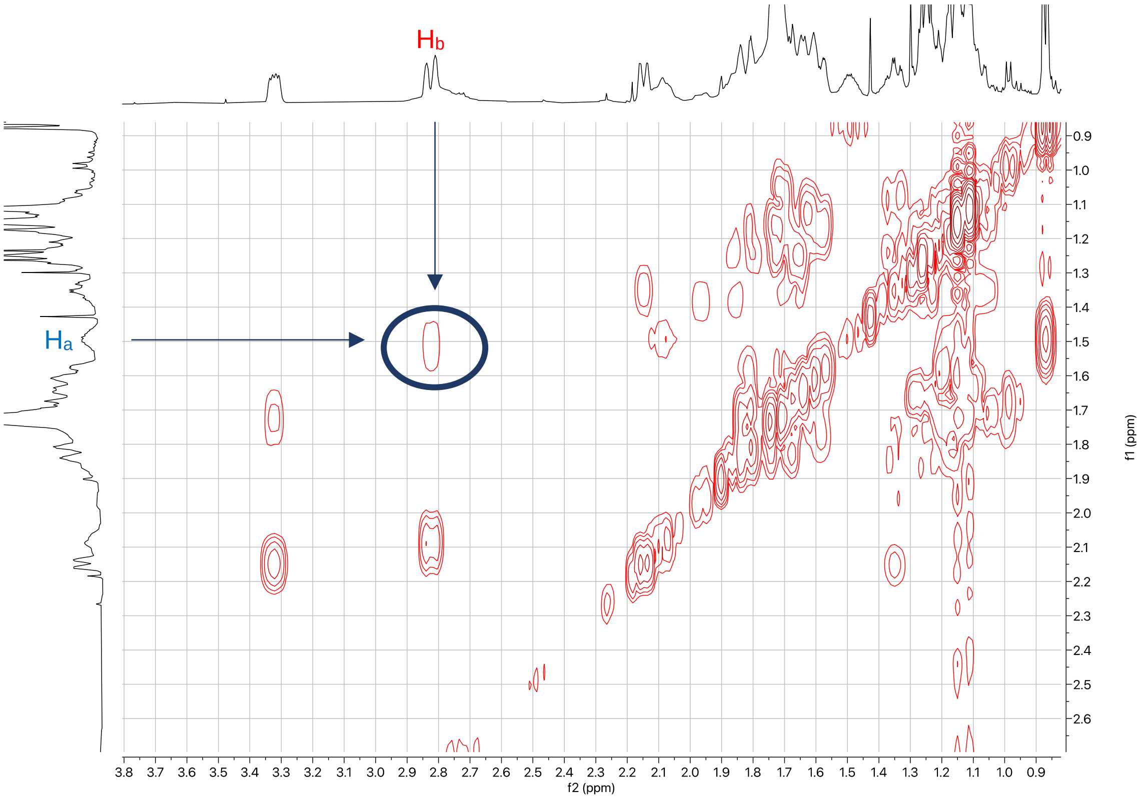

9. Stereochemical determination of Heck products by NOESY experiment

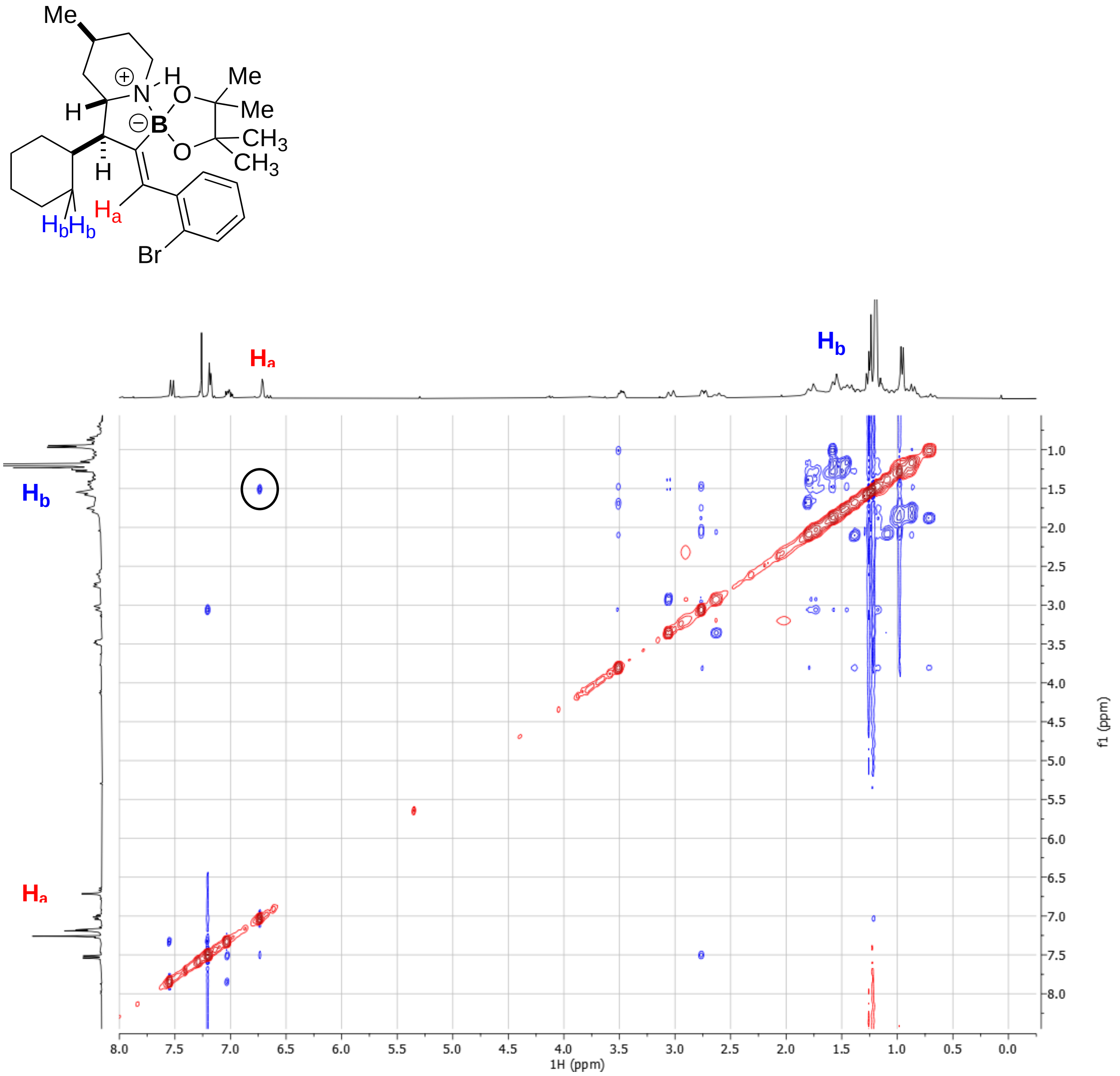

The NOESY experiment shows a cross-peak between the olefinic proton (H_a) and the CH_2 (H_b) of the cyclohexyl substituent. Furthermore, the absence of cross-peak between the vinyl proton and the methyl groups of Bpin supports the proposed stereochemistry.

10. X-Ray diffraction analysis data

10.1. X-Ray diffraction analysis data of product 7

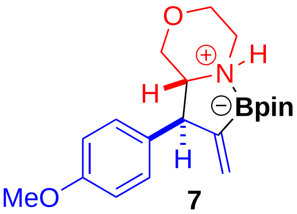

7

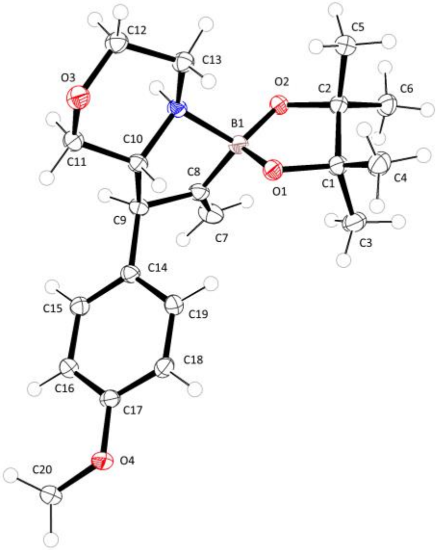

Figure S1. ORTEP plot of **7** with 50% ellipsoids.

Chemical formula	$C_{20}H_{30}BNO_4$	
Formula weight	359.26 g/mol	
Temperature	100(2) K	
Wavelength	1.54178 Å	
Crystal size	0.120 x 0.140 x 0.160 mm	
Crystal system	monoclinic	
Space group	P 1 21/n 1	
Unit cell dimensions	a = 9.4669(2) Å	α = 90°
	b = 19.0793(4) Å	β = 95.5380(10)°
	c = 10.5684(2) Å	γ = 90°
Volume	1899.97(7) $Å^3$	
Z	4	
Density (calculated)	1.256 g/cm^3	
Absorption coefficient	0.684 mm^{-1}	
F(000)	776	

10.2. X-Ray diffraction analysis data of product 16

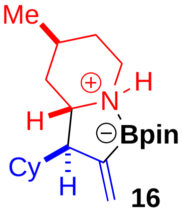

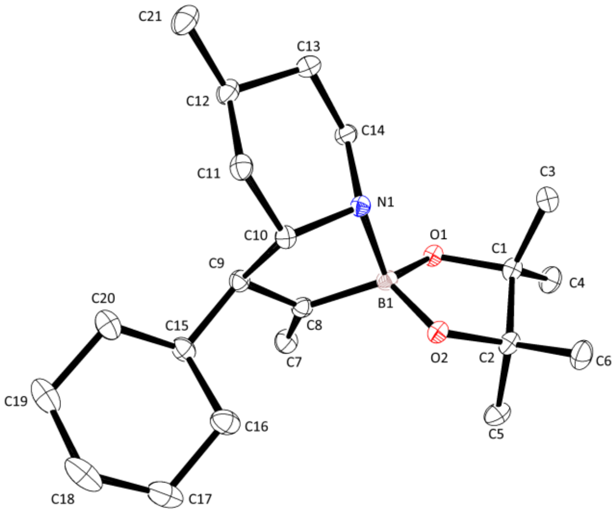

Figure S2. ORTEP plot of **16** with 50% ellipsoids.

Chemical formula	$2(C_{21}H_{38}BNO_2)\cdot BH_3O_3$	
Formula weight	756.50 g/mol	
Temperature	100(2) K	
Wavelength	1.54178 Å	
Crystal size	0.060 x 0.070 x 0.440 mm	
Crystal system	monoclinic	
Space group	I 1 2/a 1	
Unit cell dimensions	a = 21.2645(7) Å	α = 90°
	b = 12.3398(4) Å	β = 100.144(2)°
	c = 35.4577(13) Å	γ = 90°
Volume	9158.6(5) $Å^3$	
Z	8	
Density (calculated)	1.097 g/cm^3	
Absorption coefficient	0.561 mm^{-1}	
F(000)	3328	

10.3. X-Ray diffraction analysis data of product 27

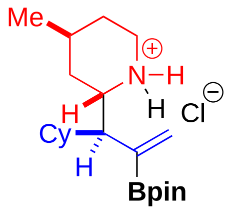

27

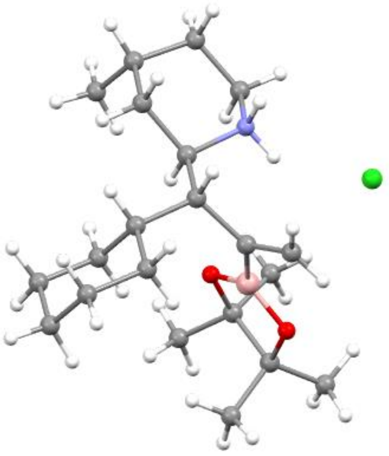

Figure S3. ORTEP plot of **27** with 30% ellipsoids.

Crystal data

Chemical formula	$C_{21}H_{39}BClNO_2$	
Formula weight	383.79 g/mol	
Temperature	100(2) K	
Wavelength	1.54178 Å	
Crystal size	0.200 x 0.293 x 0.378 mm	
Crystal habit	clear light orange block	
Crystal system	monoclinic	
Space group	P 1 21/c 1	
Unit cell dimensions	a = 13.0026(5) Å	α = 90°
	b = 11.0817(4) Å	β = 91.2307(16)°
	c = 31.7303(12) Å	γ = 90°
Volume	4571.0(3) $Å^3$	
Z	8	
Density (calculated)	1.115 g/cm^3	
Absorption coefficient	1.571 mm^{-1}	
F(000)	1680	

10.4. X-Ray diffraction analysis data of product 33

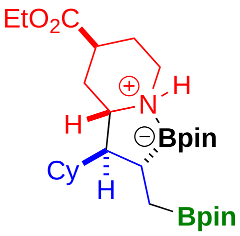

33

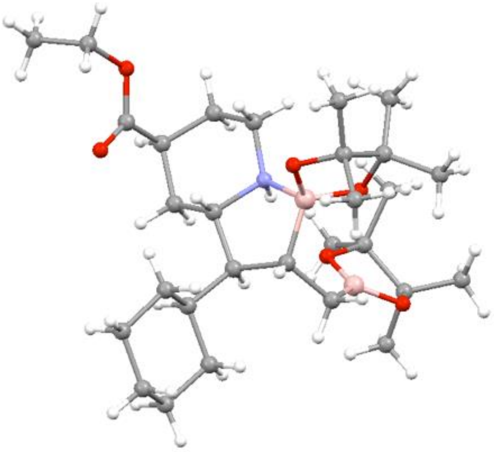

Figure S4. ORTEP plot of **33** with 30% ellipsoids.

Crystal data

Chemical formula	($C_{29}H_{53}B_2NO_6$)	
Formula weight	533.36 g/mol	
Temperature	100(2) K	
Wavelength	1.54178 Å	
Crystal size	0.26 x 0.24 x 0.17 mm	
Crystal habit	Block, clear colourless	
Crystal system	Triclinic	
Space group	*P*-1	
Unit cell dimensions	a = 8.5292(3) Å	α = 105.0567(16)°
	b = 19.2668(7) Å	β = 90.356(2)°
	c = 19.6127(8) Å	γ = 94.6767(18)°
Volume	3100.7(2) $Å^3$	
Z	2	
Density (calculated)	1.142 g/cm^3	
Absorption coefficient	0.61 mm^{-1}	
F(000)	1168	

11. Proposed mechanism for the formation of product 32

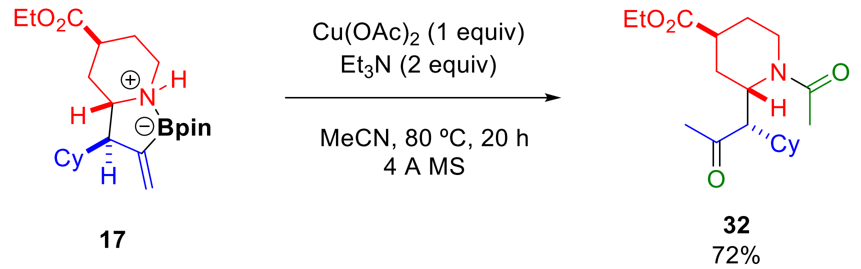

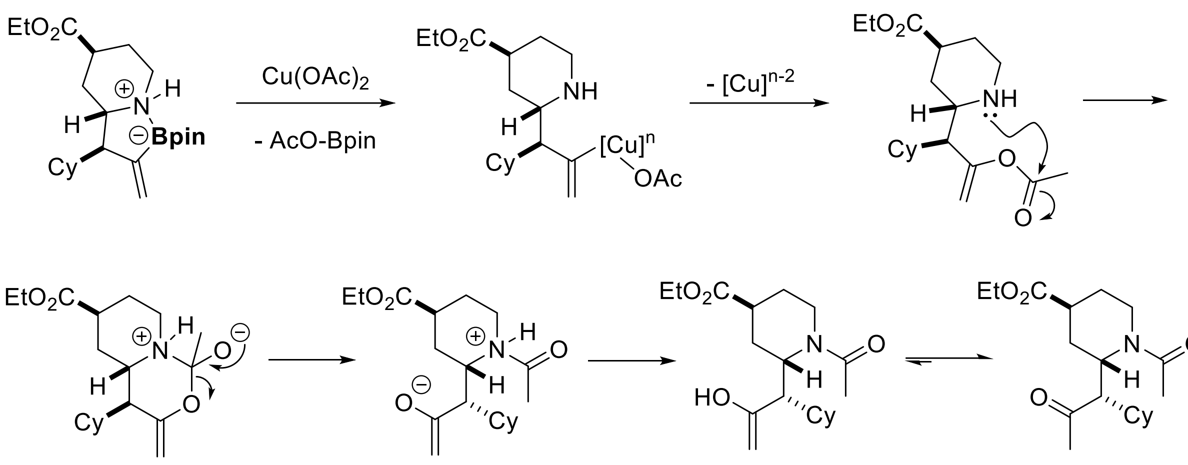

12. Imine formation experiments

NOTE: The ^{1}H NMR spectra was recorded in a Varian 300 MHz spectrometer. Chemical shifts and integrals of ^{1}H NMR signals are reported in ppm relative to CH_2Br_2 internal standard (CH_2Br_2 δ = 5.20 ppm). LiO^{t}Bu and THF-d_8 was purchased from Sigma Aldrich. Experiments were carried out at room temperature.

- **Imine formation experiment at a concentration of 0.195 M:**

A flame-dried NMR tube was charged with piperidin-1-yl benzoate **42** (10 mg, 0.95 mmol, 1 equiv). The NMR tube was subjected to vacuum and refilled with argon, repeating this cycle for three times. Then, THF-d_8 (0.5 mL, 0.2 M) CH_2Br_2 (17 mg, 0.1 mmol, 1 equiv, internal standard) were added. A ^{1}H NMR was acquired at that time (t = 0). Then, solid LiO^{t}Bu (24 mg, 0.3 mmol, 3 equiv) (weighed inside the glove box) was added under an argon flow and ^{1}H NMR (8 scans) was acquired every 2 min. Note: the integrated signals were the imine **52** peaks at 7.70 ppm (H_b, in blue), and at 2.1 ppm (H_c, in green), and of the **42** peak at 2.6 ppm (H_a, in red).

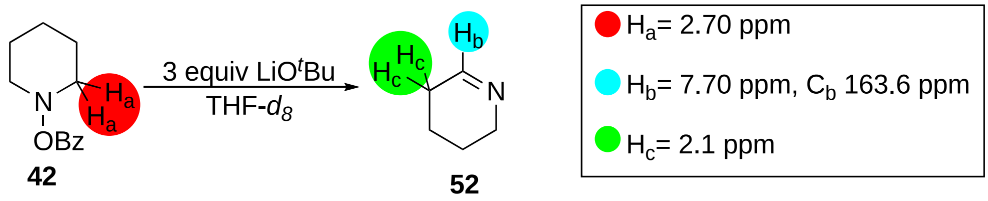

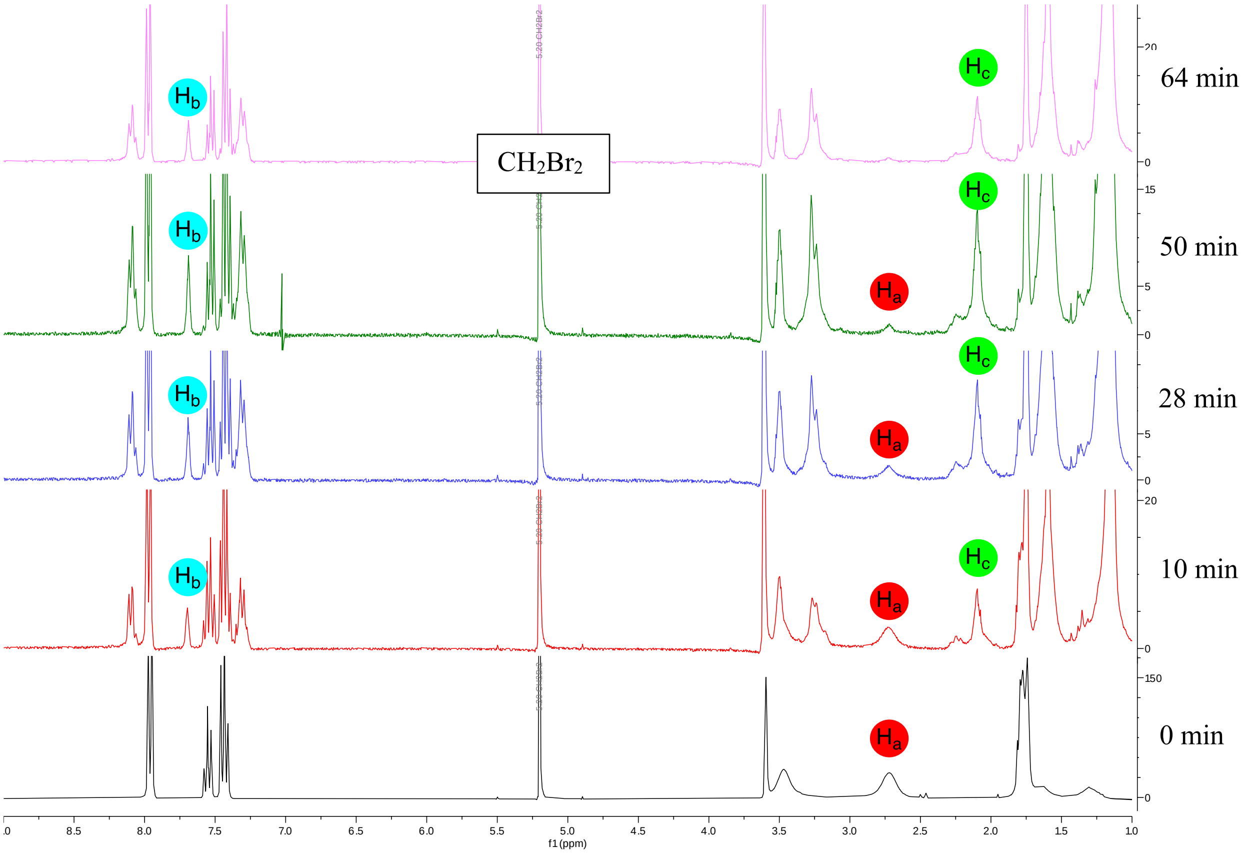

Figure S5. ^{1}H NMR at 0 min (black), 10 min (red), 28 min (blue), 50 min (green) and 64 min (purple).

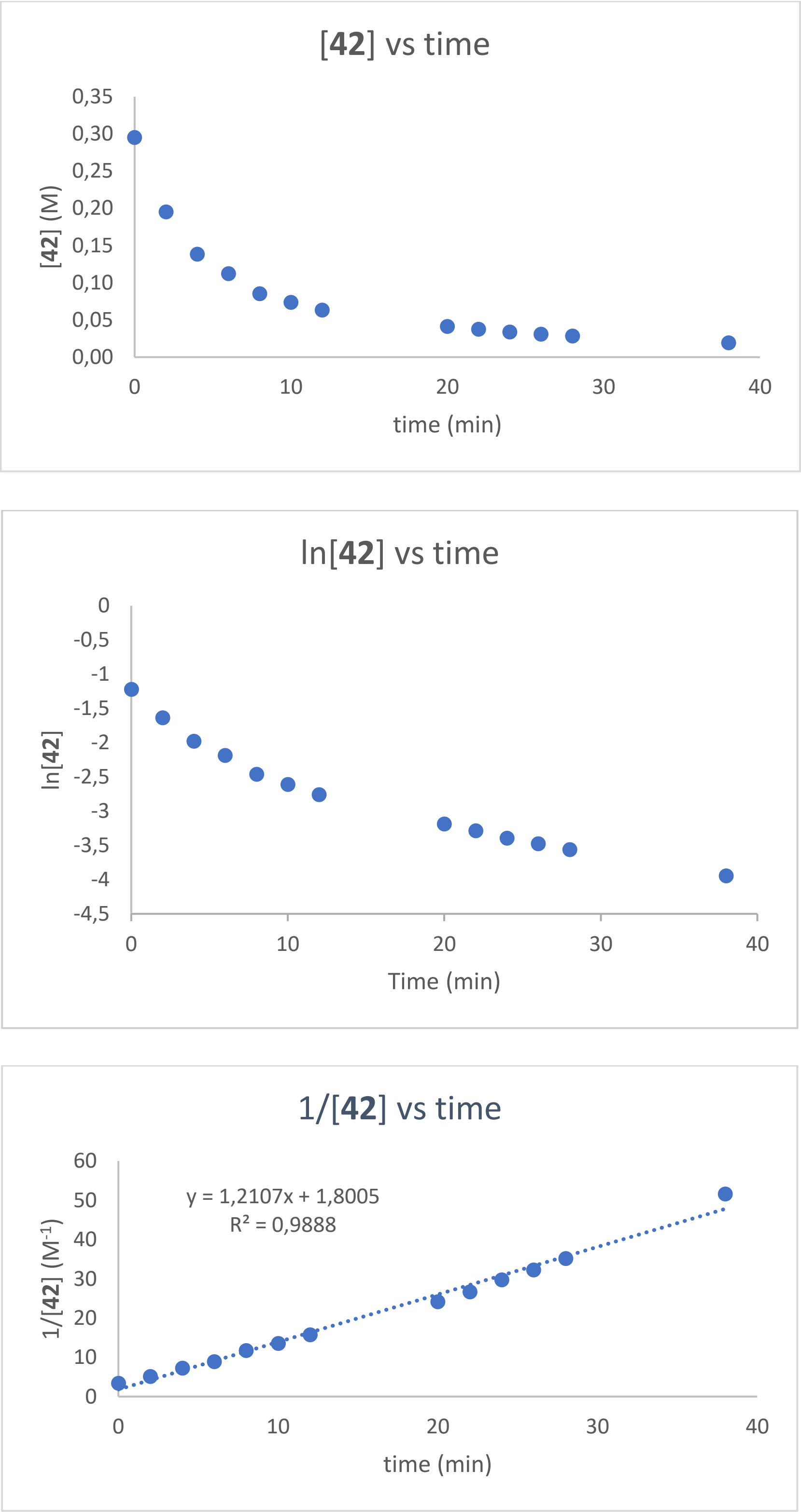

Figure S6. Imine formation plots at a concentration of 0.195 M. a) [**42**] vs time. b) ln[**42**] vs time. c) 1/[**42**] vs time.

After 60 min, it was observed that piperidin-1-yl benzoate **42** was fully converted into 2,3,4,5-tetrahydropyridine **52**.

- **Imine formation experiment at a concentration of 0.97 M:**

A flame-dried NMR tube was charged with piperidin-1-yl benzoate **42** (100 mg, 0.487 mmol ,1 equiv). The NMR tube was subjected to vacuum and refilled with argon, repeating this cycle for three times. Then, THF-d_8 (0.5 mL, 0.97 M) and CH_2Br_2 (84.7 mg, 0.487 mmol, 1 equiv, internal standard) were added. A ^{1}H NMR was acquired at that time (t = 0). Then, solid LiO^{t}Bu (119 mg, 1.46 mmol, 3 equiv) (weighed inside the glove box) was added under an argon flow and ^{1}H NMR (8 scans) and ^{13}C NMR (120 scans) were acquired every 4 mins. Note: in ^{1}H NMR the followed signals were the **52** at 7.70 ppm (H_b, in blue), and of the peak of **42** at 2.6 ppm (H_a, in red), and of the trimer peak (**53**) at 3.76 ppm (H_d, in orange). In ^{13}C NMR, the followed signals were the carbonyl of **42** at 164.3 ppm (C in purple) and the carbon of **52** at 163.6 ppm (C_b in blue).

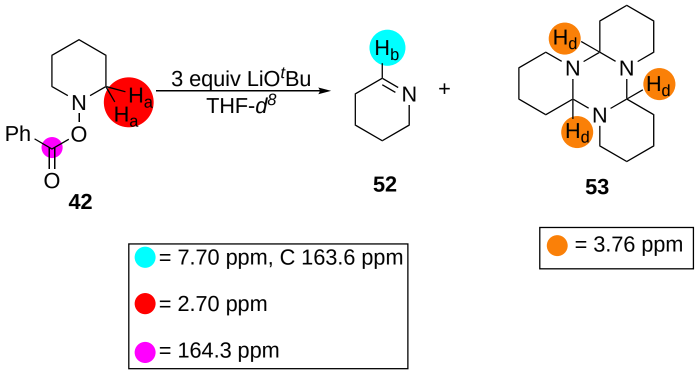

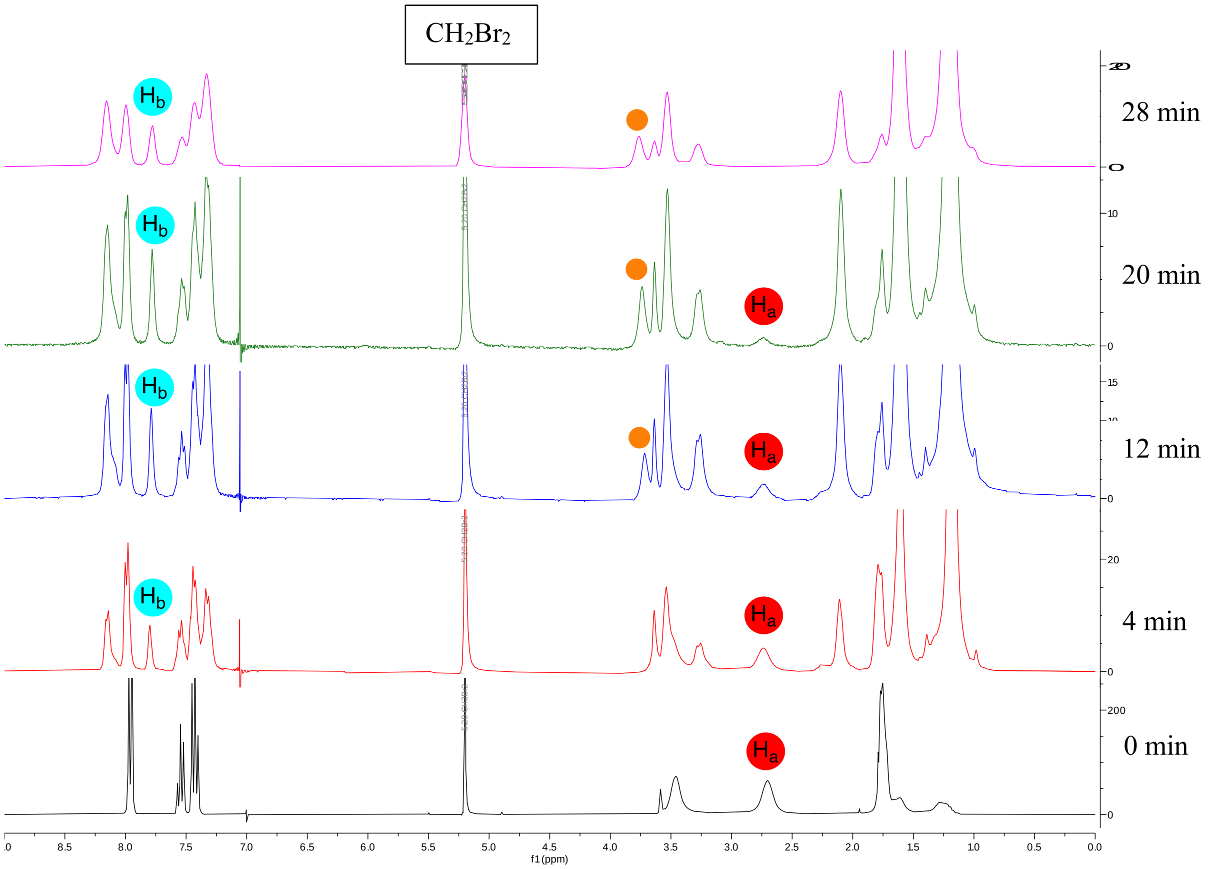

Figure S7. ^{1}H NMR spectra at 0 min (black), 4 min (red), 12 min (blue), 20 min (gren) and 28 min (purple).

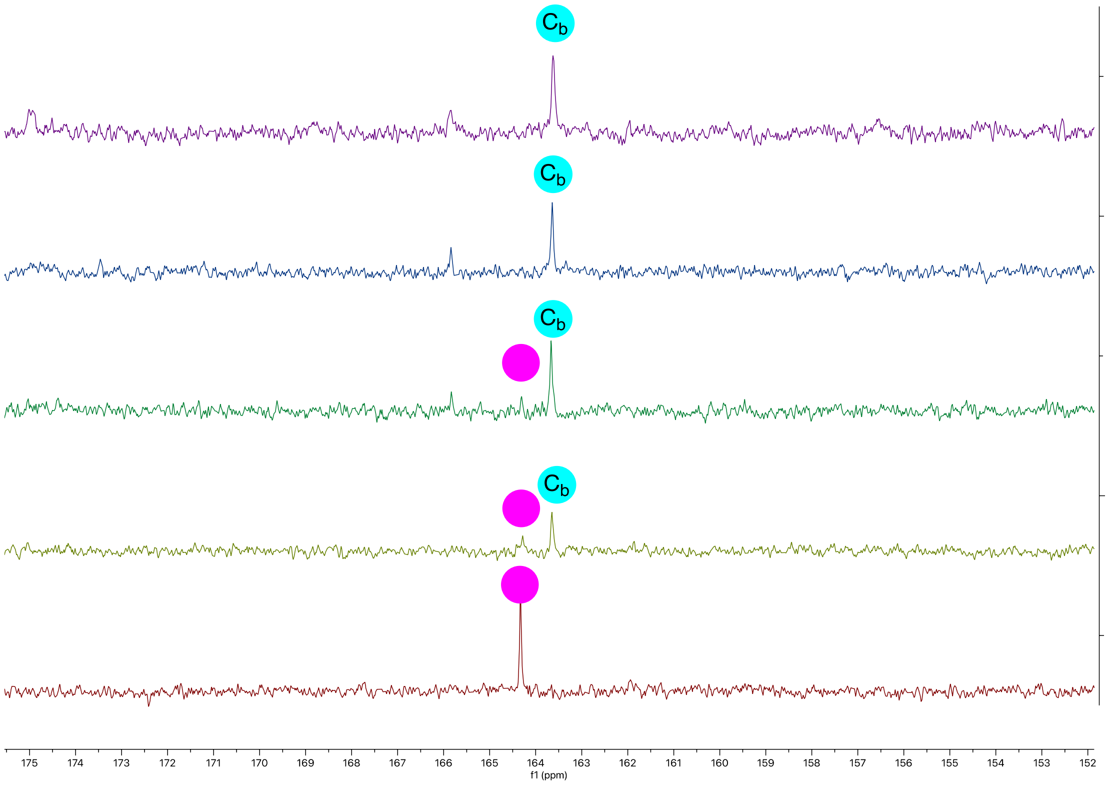

Figure S8. ^{13}C NMR spectra at 0 min (red), 4 min(yellow), 12 min (green), 20 min (blue) and 28 min (purple).

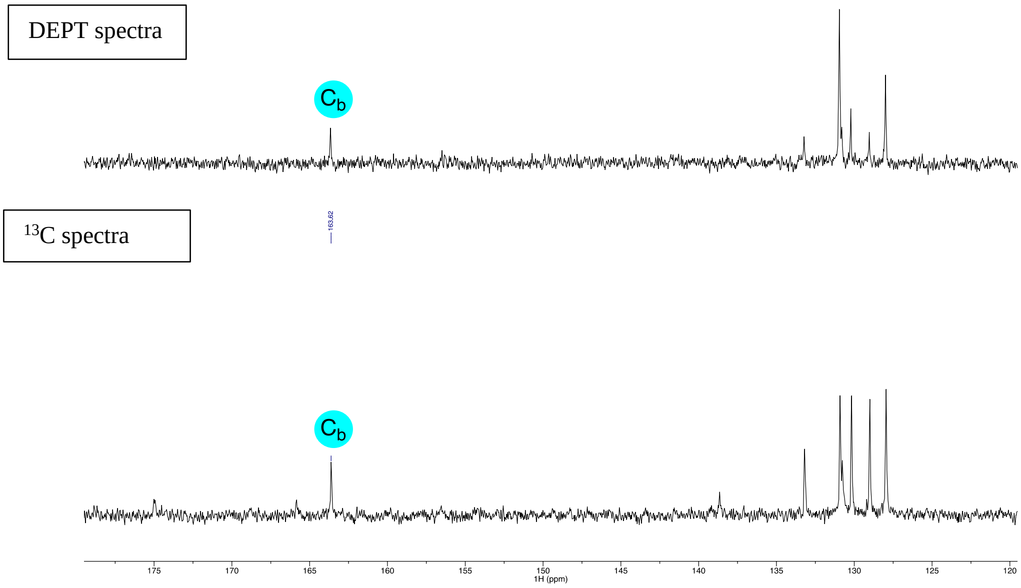

Figure S9. DEPT and ^{13}C NMR spectra after 28 min of the experiment.

The use of a higher concentration led to a faster formation of the cyclic imine **52**. After 28 min, piperidin-1-yl benzoate **42** was fully converted into 2,3,4,5-tetrahydropyridine **52**. It is important to note that at this higher concentration, formation of trimerization product **53** is also prominent since the beginning of the reaction.

- **Imine formation experiment at a concentration of 0.195 M, in the presence of P(O)Ph3:**

To evaluate whether the Lewis base additive has an effect on the imine formation, the following experiment was carried out.

A flame-dried NMR tube was charged with piperidin-1-yl benzoate **42** (20 mg, 0.97 mmol, 1 equiv) and triphenylphosphine oxide (1.4 mg, 0.05 mmol, 0.05 equiv). The NMR tube was subjected to vacuum and refilled with argon, repeating this cycle for three times. Then, THF-*d8* (0.5 mL, 0.2 M) CH_2Br_2 (17 mg, 0.1 mmol, 1 equiv, internal standard) were added. A ^{1}H NMR was acquired at that time (t = 0). Then, LiO^{t}Bu (24 mg, 0.3 mmol, 3 equiv) (weighed in a glove-box) was added under argon flow and ^{1}H NMR (8 scans) was acquired every 2 min. Note: the integrated signals were the imine **52** peaks at 7.70 ppm (H_b, in blue), and at 2.1 ppm (H_c, in green), and of the **42** peak at 2.6 ppm (H_a, in red).

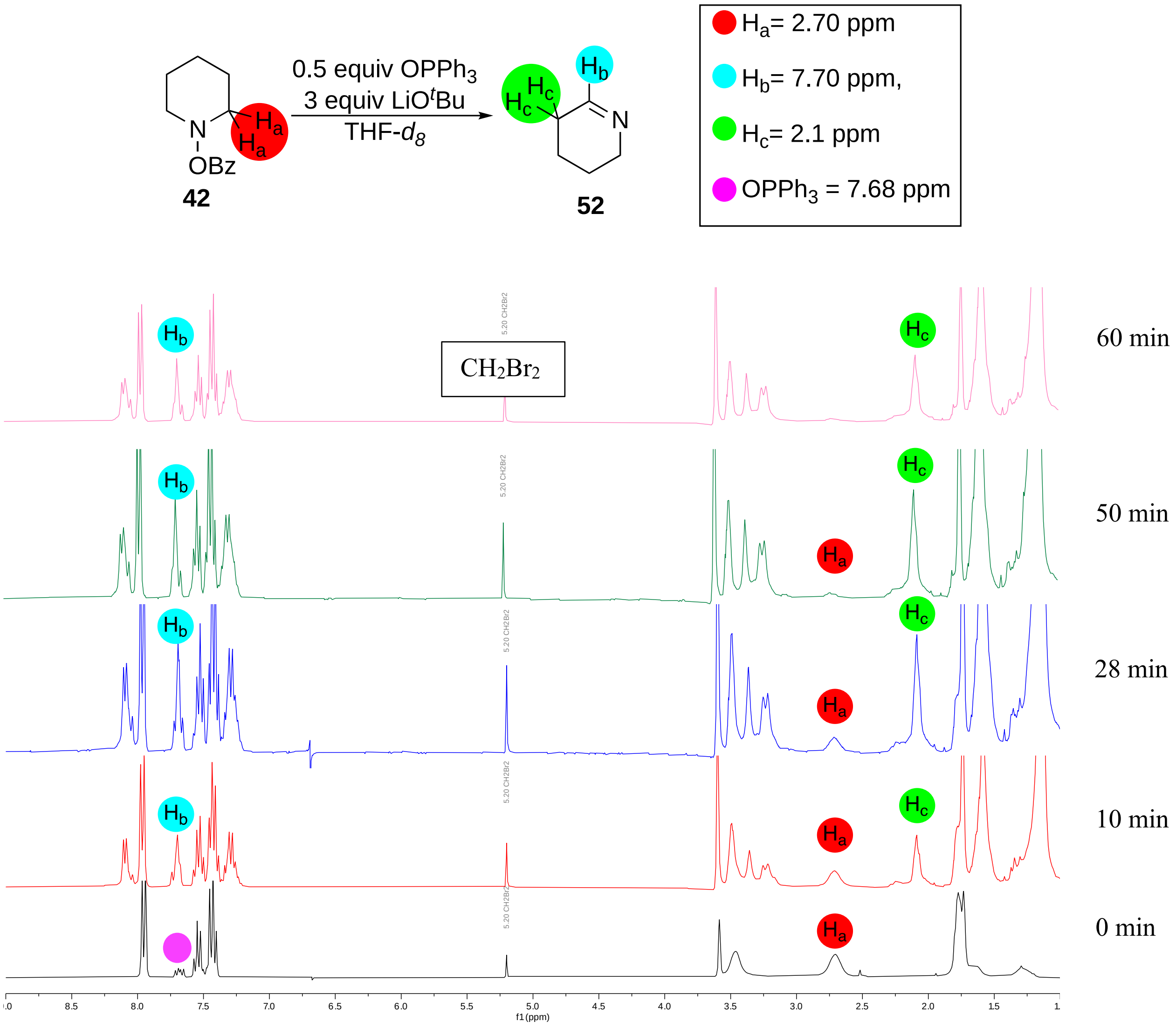

Figure S10. ^{1}H NMR at 0 min (black), 10 min (red), 28 min (blue), 50 min (green) and 60 min (purple).

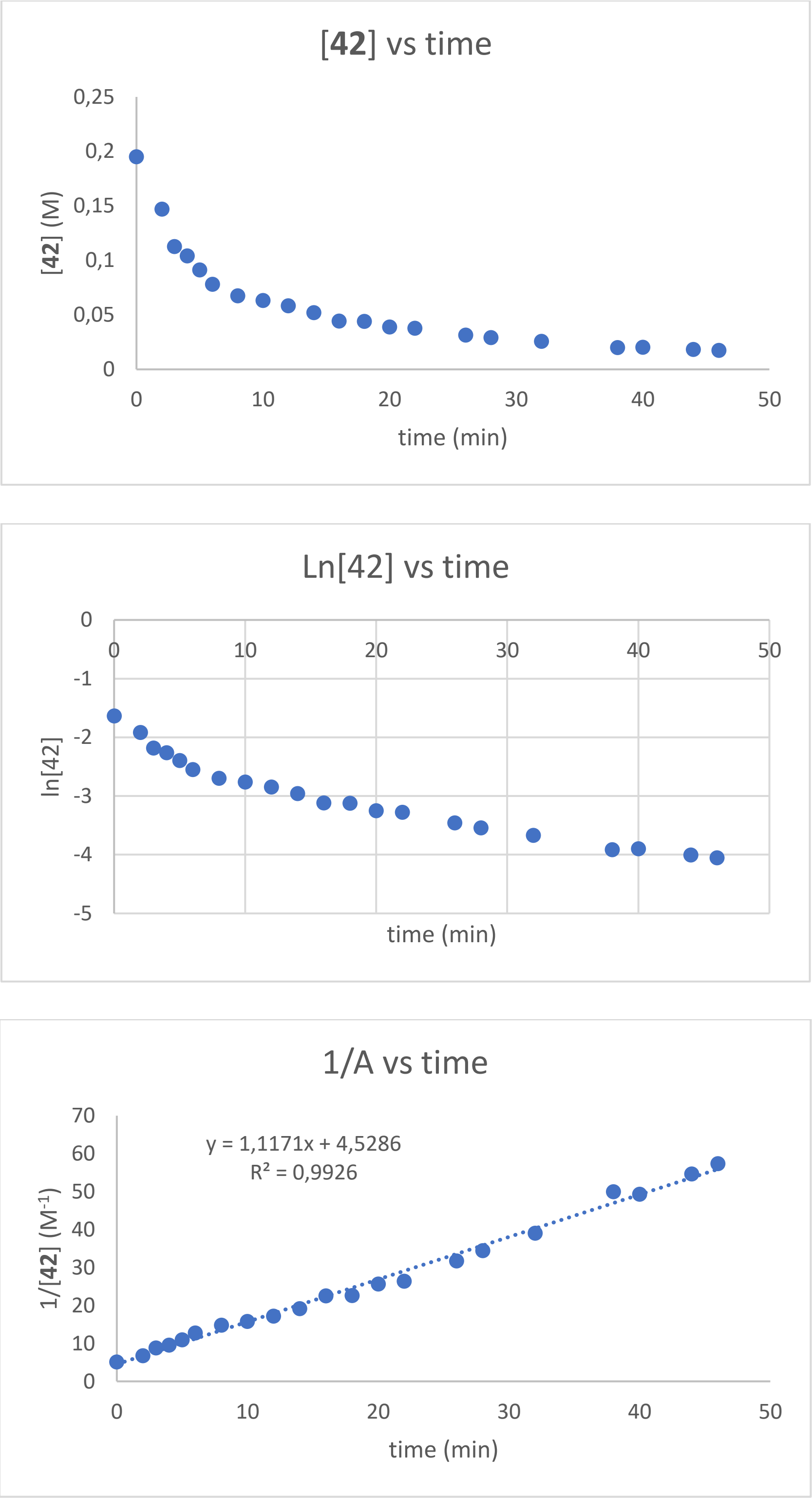

Figure S11. Imine formation plots at a concentration of 0.195 M in the presence of $P(O)Ph_3$. a) [**42**] vs time. b) ln[**42**] vs time. c) 1/[**42**] vs time.

Piperidin-1-yl benzoate **42** was fully converted into 2,3,4,5-tetrahydropyridine **52** after 60 min. No significant differences were observed in the presence of $P(O)Ph_3$ thus concluding that the Lewis base additive does not have any effect on the imine formation.

13. ^{31}P NMR studies of phosphine-Cu complexes

^{31}P NMR spectra was recorded in a Varian 500 MHz spectrometer at -20 ºC in a precooled spectrometer. Chemical shifts of ^{31}P NMR signals are reported in ppm relative to a phosphoric acid solution, 85% in D_2O as external standard (0 ppm).

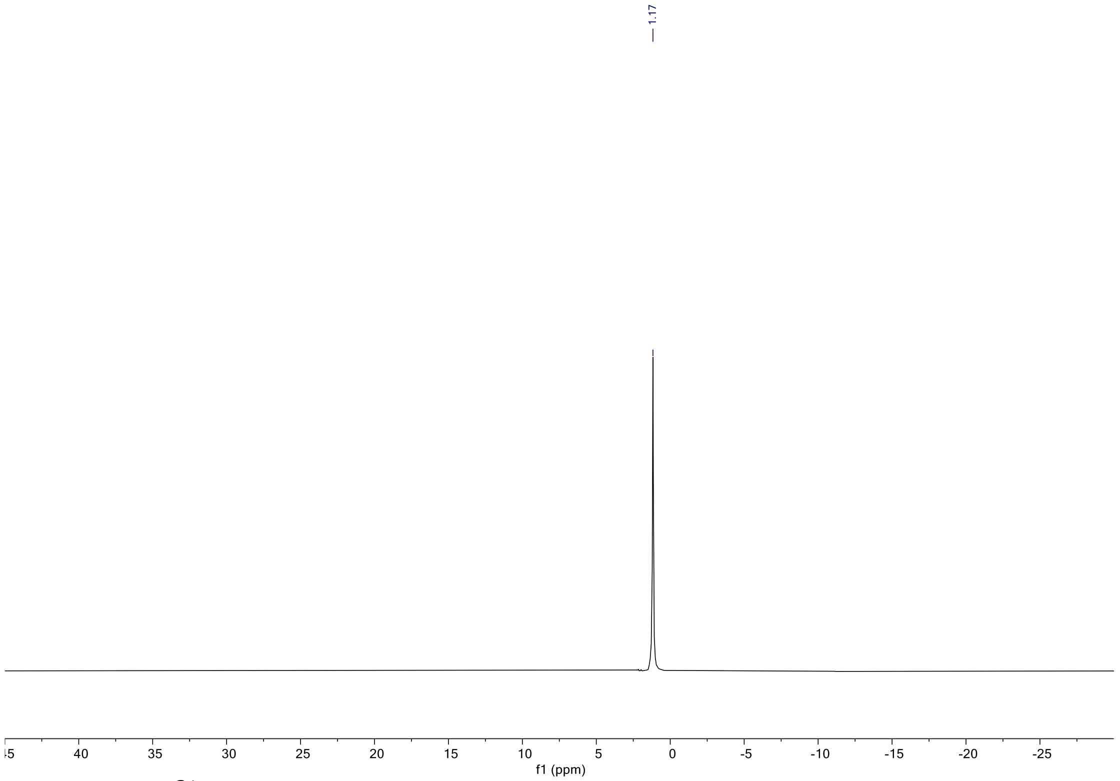

Figure S12. ^{31}P NMR spectrum of dcpe (1.2 ppm) in THF-d_8 at -20 ºC.

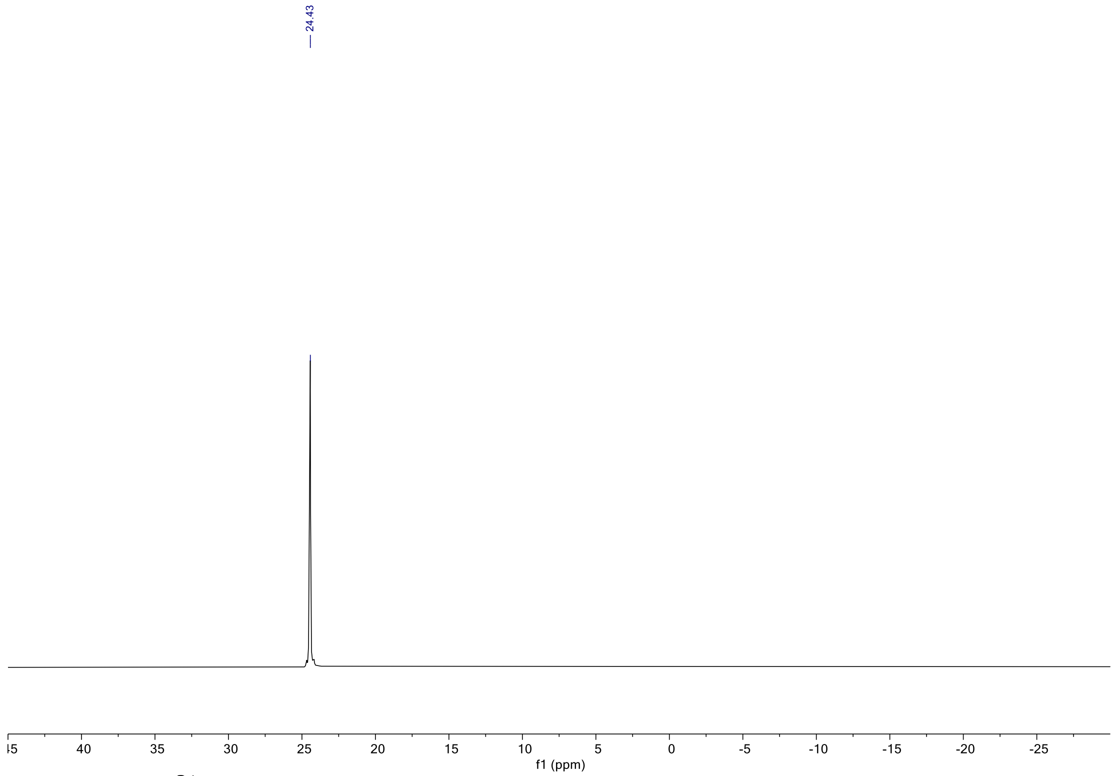

Figure S13. ^{31}P NMR spectrum of $P(O)Ph_3$ (24.4 ppm) in THF-d_8 at -20 ºC.

- **Preparation of reference copper complexes using dcpe:**

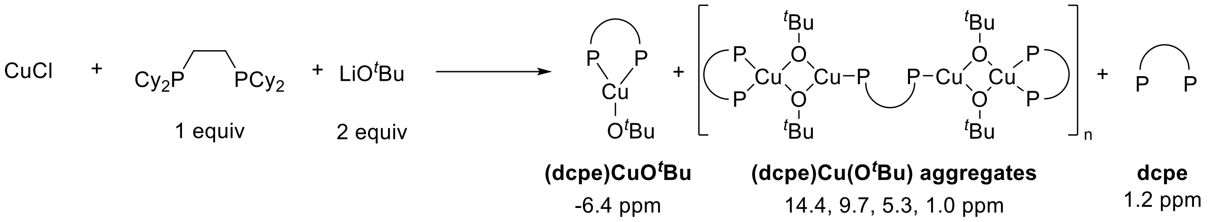

A) Inside a glovebox, an oven-dried screw cap vial equipped with a magnetic stirring bar was charged with CuCl (0.02 mmol, 1.0 equiv), dcpe (0.022 mmol, 1.1 equiv), LiO*t*Bu (0.04 mmol, 2.0 equiv) and THF (0.5 ml). The reaction mixture was stirred for 5 min and then, the mixture was transferred to an oven-dried NMR tube, equipped with a capillary of THF-d_8, while still in a glovebox. The NMR tube was removed from the glovebox and ^{31}P NMR spectra was recorded at -20 ºC (Fig. S14, Spectrum A). Resonances corresponding to (dcpe)CuO*t*Bu (-6.4 ppm) and free dcpe ligand (1.2 ppm) were detected, together with a set of signals (14.4, 9.7, 6.7 and 5.3 ppm) which could be related to the formation of Cu aggregates.[9]

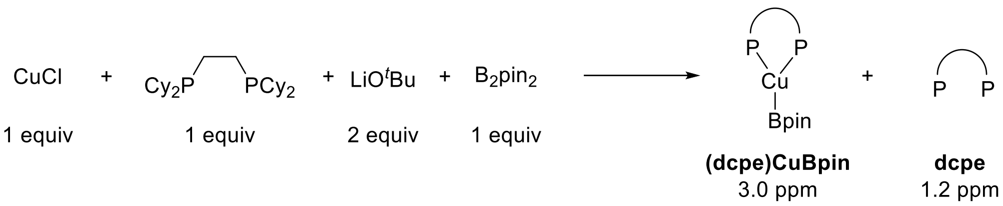

B) Inside a glovebox, an oven-dried screw cap vial equipped with a magnetic stirring bar was charged with CuCl (0.02 mmol, 1.0 equiv), dcpe (0.022 mmol, 1.1 equiv), B_2pin_2 (0.024 mmol, 1.2 equiv), LiO*t*Bu (0.04 mmol, 2.0 equiv) and THF (0.5 ml). The reaction mixture was stirred for 5 min and then, the mixture was transferred to an oven-dried NMR tube, equipped with a capillary of THF-d_8, while still in a glovebox. The NMR tube was removed from the glovebox and ^{31}P NMR spectra was recorded at -20 ºC (Figure S14, Spectrum B). Resonances corresponding to (dcpe)CuBpin (3.0 ppm) and free dcpe (1.2 ppm) were detected. Complete disappearance of the initial signals assigned to L-CuO*t*-Bu was observed.

There was a ~50% of free dcpe ligand, thus suggesting that the Cu(dcpe)(Bpin) and the (O*t*Bu)(Bpin)CuLi are in a ~1:1 ratio.

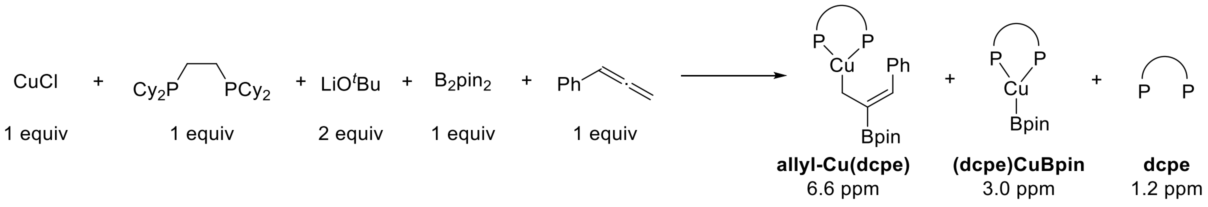

C) Inside a glovebox, an oven-dried screw cap vial equipped with a magnetic stirring bar was charged with CuCl (0.02 mmol, 1.0 equiv), dcpe (0.022 mmol, 1.1 equiv), B_2pin_2 (0.024

[9] Lee, J.; Radomkit, S.; Torker, S.; del Pozo, J.; Hoveyda, A. H. *Nat. Chem.* **2018**, *10*, 99–108.

mmol, 1.2 equiv), LiO^{t}Bu (0.04 mmol, 2.0 equiv) and THF (0.5 ml). The reaction mixture was stirred for 5 min and then phenylallene was added (0.04 mmol, 2.0 equiv, 2.5 M in THF). The mixture was stirred for 10 min and was transferred to an oven-dried NMR tube, equipped with a capillary of THF-d_8, while still in a glovebox. The NMR tube was removed from the glovebox and ^{31}P NMR spectra was recorded at -20 ºC (Figure S14, Spectrum C). Observation of the mixture revealed a new resonance at 6.6 ppm consistent with the formation of allyl-Cu(dcpe) complex (~50% conversion). Free dcpe (1.2 ppm) was still observed.

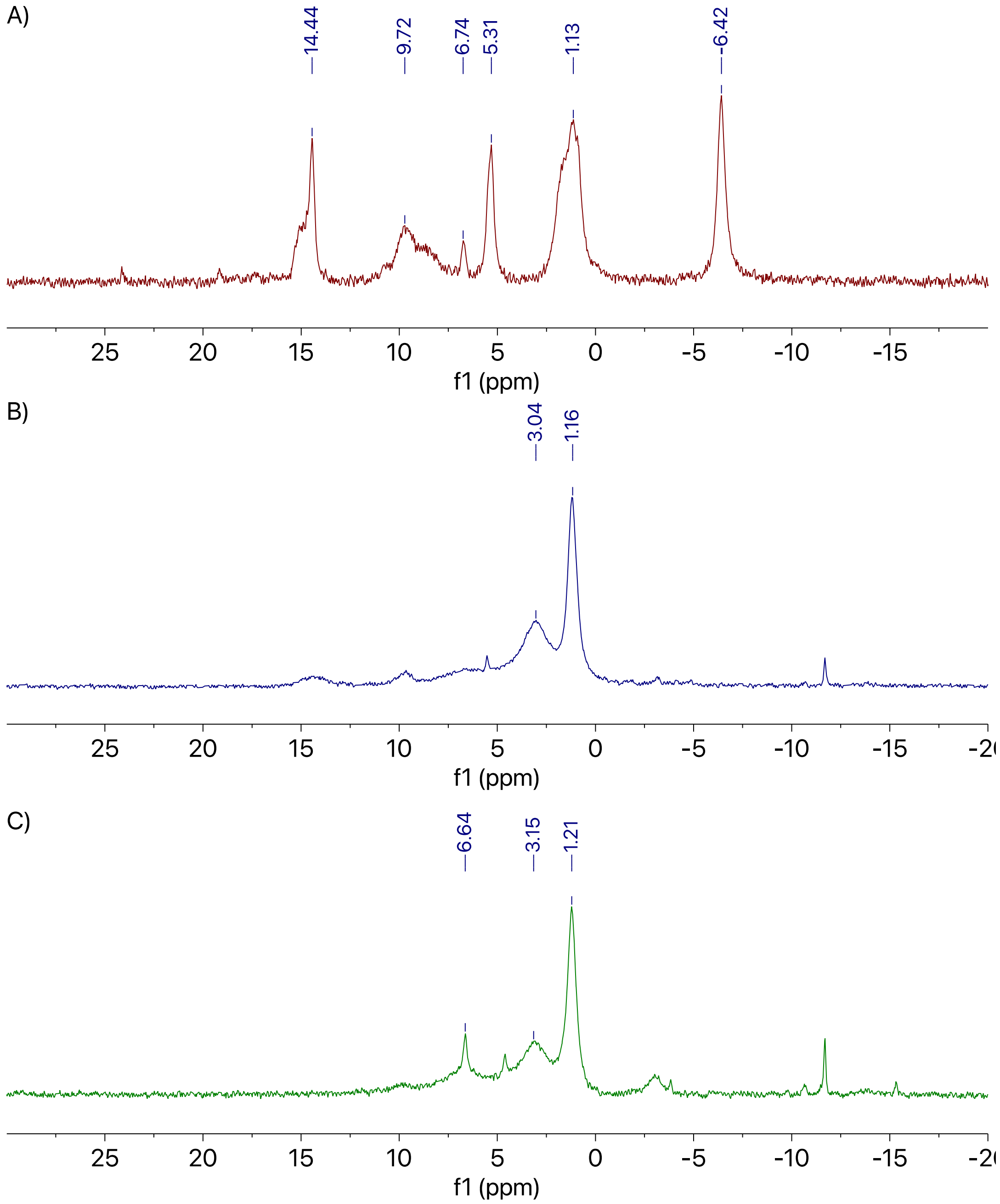

Figure S14. ^{31}P NMR spectra of A) CuCl + dcpe + LiO^{t}Bu, B) CuCl + dcpe + LiO^{t}Bu + B_2pin_2 and C) CuCl + dcpe + LiO^{t}Bu + B_2pin_2 + phenylallene.

- **Preparation of reference copper complexes using dcpe and P(O)Ph3:**

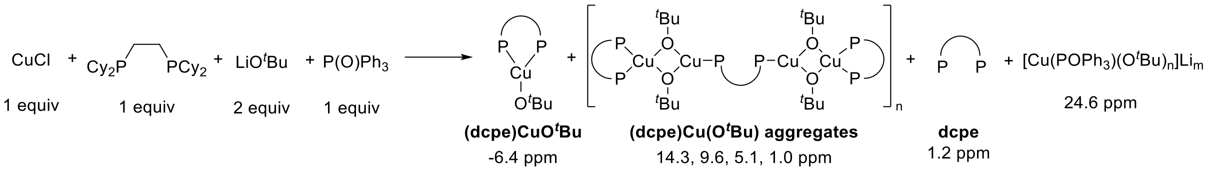

D) Inside a glovebox, an oven-dried screw cap vial equipped with a magnetic stirring bar was charged with CuCl (0.02 mmol, 1.0 equiv), dcpe (0.022 mmol, 1.1 equiv), $P(O)Ph_3$ (0.022 mmol, 1.1 equiv), LiO^{t}Bu (0.04 mmol, 2.0 equiv) and THF (0.5 ml). The reaction mixture was stirred for 5 min and then, the mixture was transferred to an oven-dried NMR tube, equipped with a capillary of THF-d_8, while still in a glovebox. The NMR tube was removed from the glovebox and ^{31}P NMR spectra was recorded at -20 ºC (Figure S15, Spectrum D). Resonances corresponding to (dcpe)CuO^{t}Bu (-6.4 ppm), free dcpe ligand (broad signal around 1 ppm), and Cu aggregates (14.3, 9.6, 5.1, 0.9 ppm) were detected. Broadening of the signal corresponding to $P(O)Ph_3$ (24.6 ppm) suggest that this compound might be coordinated.

Comparison of spectrum A with spectrum D suggests that formation of Cu aggregates is slightly diminished in the latter.

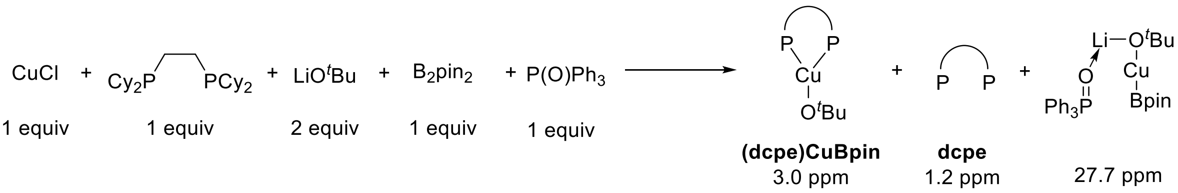

E) Inside a glovebox, an oven-dried screw cap vial equipped with a magnetic stirring bar was charged with CuCl (0.02 mmol, 1.0 equiv), dcpe (0.022 mmol, 1.1 equiv), $P(O)Ph_3$ (0.022 mmol, 1.1 equiv), B_2pin_2 (0.024 mmol, 1.2 equiv), LiO^{t}Bu (0.04 mmol, 2.0 equiv) and THF (0.5 ml). The reaction mixture was stirred for 5 min and then, the mixture was transferred to an oven-dried NMR tube, equipped with a capillary of THF-d_8, while still in a glovebox. The NMR tube was removed from the glovebox and ^{31}P NMR spectra was recorded at -20 ºC (Figure S15, Spectrum E). Resonances corresponding to (dcpe)CuBpin (3.0 ppm) and free dcpe (1.2 ppm) were observed, together with a resonance at 27.6 ppm which corresponds to a new coordinated $P(O)Ph_3$ species (likely ($POPh_3$)(O^{t}Bu)(Bpin)CuLi). Complete disappearance of the initial signals assigned to L-CuO^{t}Bu was observed.

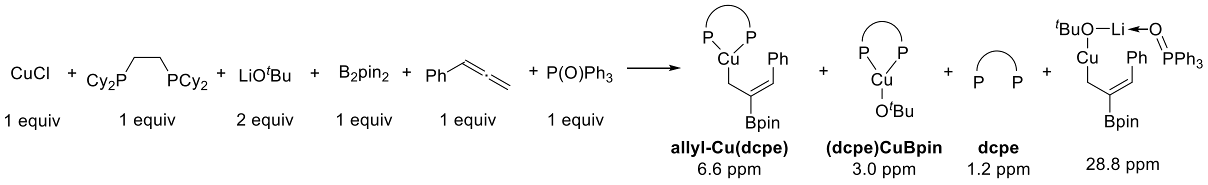

F) Inside a glovebox, an oven-dried screw cap vial equipped with a magnetic stirring bar was charged with CuCl (0.02 mmol, 1.0 equiv), dcpe (0.022 mmol, 1.1 equiv), $P(O)Ph_3$ (0.022 mmol, 1.1 equiv), B_2pin_2 (0.024 mmol, 1.2 equiv), LiO^{t}Bu (0.04 mmol, 2.0 equiv) and THF

(0.5 ml). The reaction mixture was stirred for 5 min and then phenylallene was added (0.04 mmol, 2.0 equiv, 2.5 M in THF). The mixture was stirred for 1 h and was transferred to an oven-dried NMR tube, equipped with a capillary of THF-d_8, while still in a glovebox. The NMR tube was removed from the glovebox and ^{31}P NMR spectra was recorded at -20 ºC (Figure S15, Spectrum F). Observation of the mixture revealed the formation of allyl-Cu(dcpe) complex (6.6 ppm) and the appearance of a new resonance at 28.8 ppm which might correspond to an allyl-Cu($POPh_3$) species (peak broadening might suggest the presence of other species). Free dcpe (1.1 ppm) was still observed.

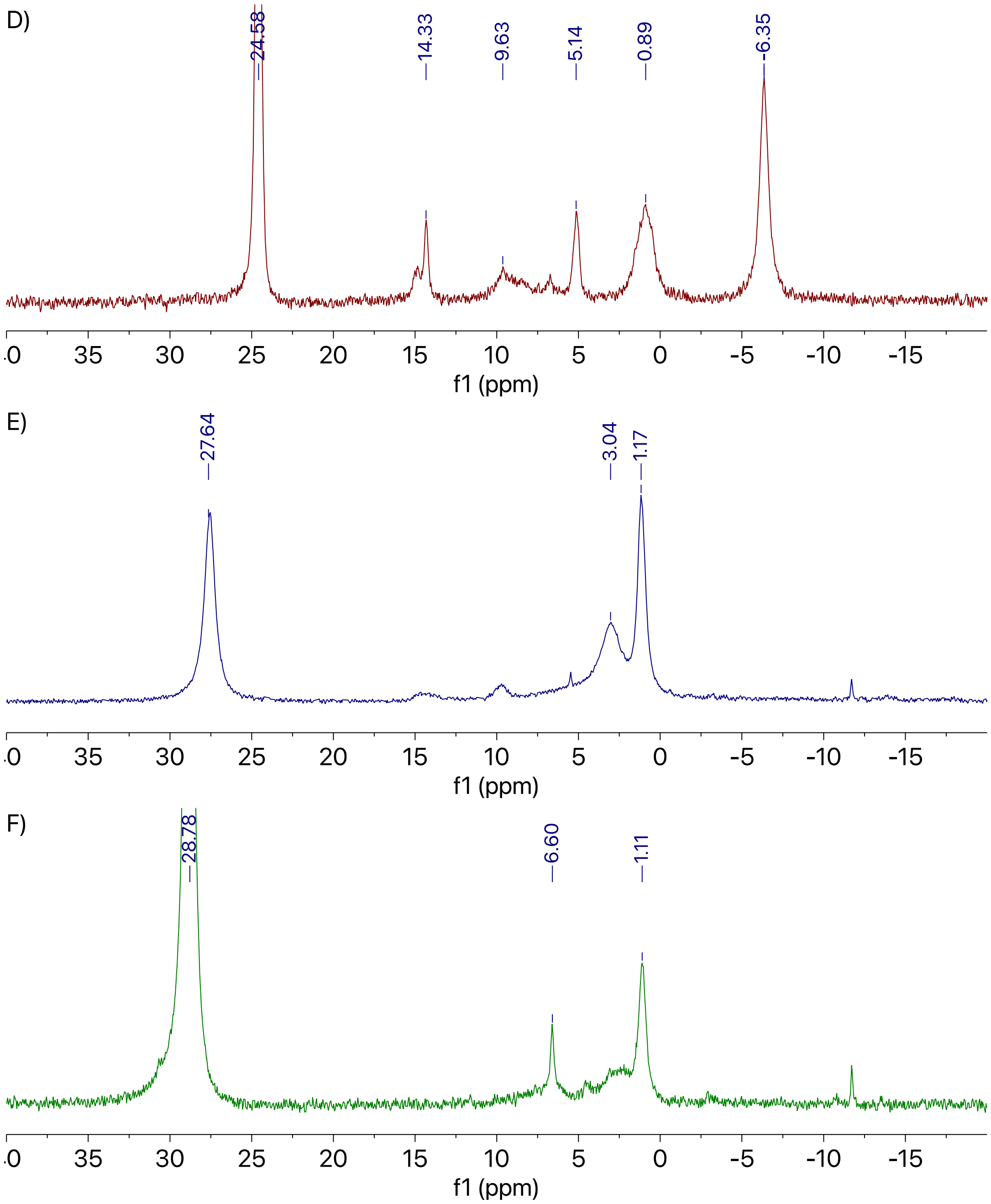

Figure S15. ^{31}P NMR spectra of D) CuCl + dcpe + P(O)Ph_3 + LiO^{t}Bu, E) CuCl + dcpe + P(O)Ph_3 +LiO^{t}Bu + B_2pin_2 and F) CuCl + dcpe + P(O)Ph_3 + LiO^{t}Bu, B_2pin_2 + phenylallene.

- **Preparation of reference copper complexes using $P(O)Ph_3$:**

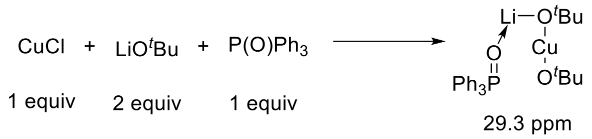

G) Inside a glovebox, an oven-dried screw cap vial equipped with a magnetic stirring bar was charged with CuCl (0.02 mmol, 1.0 equiv), $P(O)Ph_3$ (0.022 mmol, 1.1 equiv), LiO^{t}Bu (0.04 mmol, 2.0 equiv) and THF (0.5 ml). The reaction mixture was stirred for 5 min and then, the mixture was transferred to an oven-dried NMR tube, equipped with a capillary of THF-d_8, while still in a glovebox. The NMR tube was removed from the glovebox and ^{31}P NMR spectra was recorded at -20 ºC (Figure S16, Spectrum G). A single resonance at 29.3 ppm was observed. Chemical shift displacement from free $P(O)Ph_3$ and peak broadening suggests that $P(O)Ph_3$ may be coordinated most likely through metal ion chelation[10] forming $(POPh_3)(O^tBu)_2CuLi$ complex.

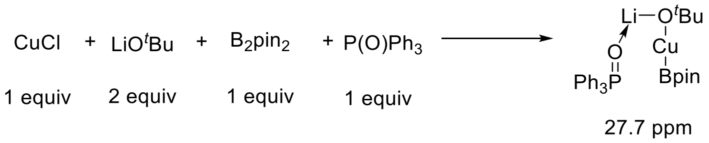

H) Inside a glovebox, an oven-dried screw cap vial equipped with a magnetic stirring bar was charged with CuCl (0.02 mmol, 1.0 equiv), $P(O)Ph_3$ (0.022 mmol, 1.1 equiv), B_2pin_2 (0.024 mmol, 1.2 equiv), LiO^{t}Bu (0.04 mmol, 2.0 equiv) and THF (0.5 ml). The reaction mixture was stirred for 5 min and then, the mixture was transferred to an oven-dried NMR tube, equipped with a capillary of THF-d_8, while still in a glovebox. The NMR tube was removed from the glovebox and ^{31}P NMR spectra was recorded at -20 ºC (Figure S16, Spectrum H). A single resonance at 27.7 ppm was observed. This peak perfectly overlaps with the peak observed in spectrum E and might correspond to $(POPh_3)(O^tBu)(Bpin)CuLi$.

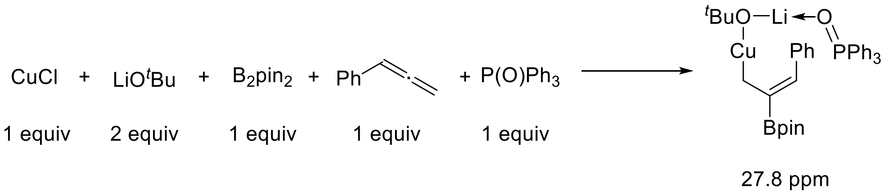

I) Inside a glovebox, an oven-dried screw cap vial equipped with a magnetic stirring bar was charged with CuCl (0.02 mmol, 1.0 equiv), $P(O)Ph_3$ (0.022 mmol, 1.1 equiv), B_2pin_2 (0.024 mmol, 1.2 equiv), LiO^{t}Bu (0.04 mmol, 2.0 equiv) and THF (0.5 ml). The reaction mixture was stirred for 5 min and then phenylallene was added (0.04 mmol, 2.0 equiv, 2.5 M in THF). The mixture was stirred for 1 h and was transferred to an oven-dried NMR tube, equipped with a capillary of THF-d_8, while still in a glovebox. The NMR tube was removed from the glovebox

[10] Konovalov, A. I.; Benet-Buchholz, J.; Martin, E.; Grushin, V. V. *Angew. Chem. Int. Ed.* **2013**, *52*, 11637–11641

and ^{31}P NMR spectra was recorded at -20 ºC (Figure S16, Spectrum I). A single resonance at 27.8 ppm was observed. This peak would correspond to an allyl-Cu(POPh$_3$)(O^{t}Bu)Li species.

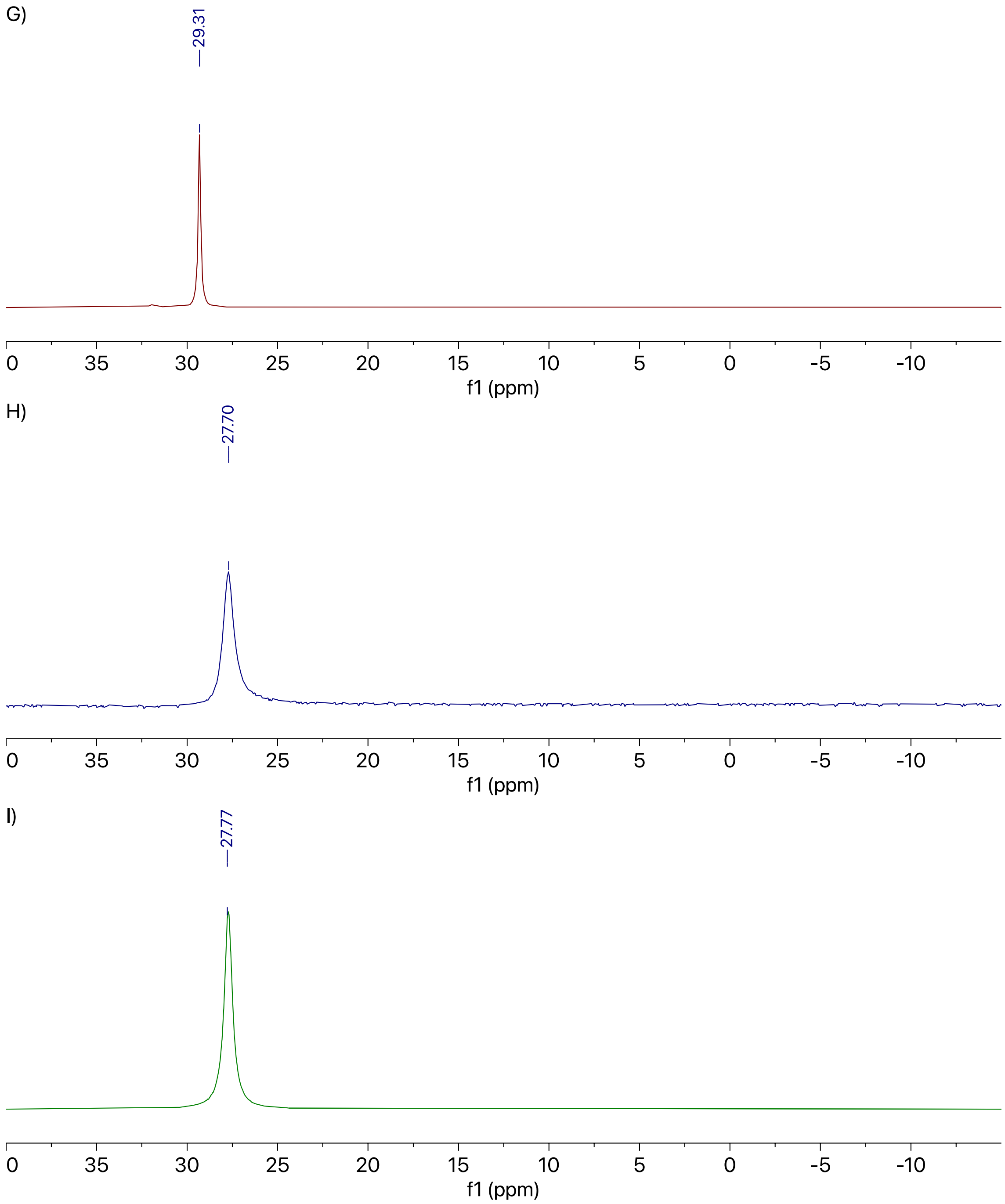

Figure S16. ^{31}P NMR spectra of G) CuCl + OPPh$_3$ + LiO^{t}Bu, H) CuCl + P(O)Ph$_3$ + LiO^{t}Bu + B$_2$pin$_2$ and I) CuCl + P(O)Ph$_3$ + LiO^{t}Bu + B$_2$pin$_2$ + phenylallene.

- **Preparation of copper complexes using dcpe (2.0 equiv):**

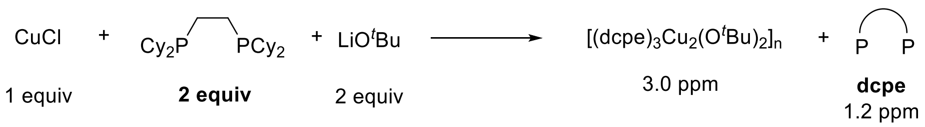

J) Inside a glovebox, an oven-dried screw cap vial equipped with a magnetic stirring bar was charged with CuCl (0.02 mmol, 1.0 equiv), dcpe (0.04 mmol, 2.0 equiv), LiO^{t}Bu (0.04 mmol, 2.0 equiv) and THF (0.5 ml). The reaction mixture was stirred for 5 min and then, the mixture was transferred to an oven-dried NMR tube, equipped with a capillary of THF-d_8, while still in a glovebox. The NMR tube was removed from the glovebox and ^{31}P NMR spectra was recorded at -20 ºC (Figure S17, Spectrum J). Observation of the mixture revealed two resonances at 3.0 ppm and 1.2 ppm (free dcpe ligand).

Interestingly, excess of dcpe ligand completely precluded the formation of the LCu-O^{t}Bu aggregates observed when 1 equiv of dcpe is used (see spectra A and D). However, the reaction did not lead to the formation of Cu(dcpe)-O^{t}Bu (resonance at -6.4 ppm in A and D) and a different Cu species (2.9 ppm) was formed under these conditions. The fact that free dcpe ligand was observed suggests that these species might have a $[(dcpe)_3(O^tBu)_2Cu_2]_n$ formula. These species might feature a different kinetic behavior than Cu(dcpe)-O^{t}Bu and that would explain the different results obtained depending on the amount of dcpe ligand used in the catalytic three-component coupling (Table S2 and Table 1, entries 11-13).

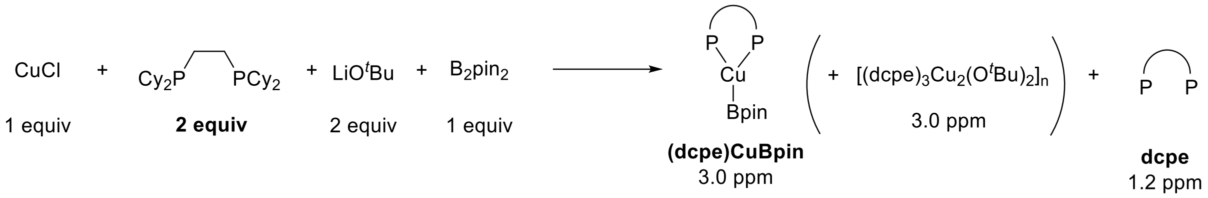

K) Inside a glovebox, an oven-dried screw cap vial equipped with a magnetic stirring bar was charged with CuCl (0.02 mmol, 1.0 equiv), dcpe (0.04 mmol, 2.0 equiv), B_2pin_2 (0.024 mmol, 1.2 equiv), LiO^{t}Bu (0.04 mmol, 2.0 equiv) and THF (0.5 ml). The reaction mixture was stirred for 5 min and then, the mixture was transferred to an oven-dried NMR tube, equipped with a capillary of THF-d_8, while still in a glovebox. The NMR tube was removed from the glovebox and ^{31}P NMR spectra was recorded at -20 ºC (Figure S17, Spectrum K). Observation of the mixture revealed two resonances at 3.0 ppm and 1.2 ppm (free dcpe ligand). Since the chemical shift (~3 ppm) of Cu(dcpe)Bpin and the Cu species observed in spectrum J, it is difficult to conclude if there is full conversion to the former under these conditions.

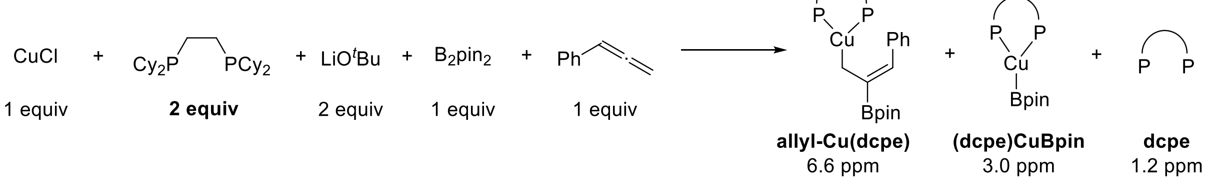

L) Inside a glovebox, an oven-dried screw cap vial equipped with a magnetic stirring bar was charged with CuCl (0.02 mmol, 1.0 equiv), dcpe (0.04 mmol, 2.0 equiv), B_2pin_2 (0.024 mmol, 1.2 equiv), LiO^{t}Bu (0.04 mmol, 2.0 equiv) and THF (0.5 ml). The reaction mixture was

stirred for 5 min and then phenylallene was added (0.04 mmol, 2.0 equiv, 2.5 M in THF). The mixture was stirred for 40 min and was transferred to an oven-dried NMR tube, equipped with a capillary of THF-d_8, while still in a glovebox. The NMR tube was removed from the glovebox and ^{31}P NMR spectra was recorded at -20 ºC (Figure S17, Spectrum L). Observation of the mixture revealed a new signal at 6.6 ppm consistent with the formation of allyl-Cu(dcpe) complex. Other species such as Cu(dcpe)Bpin and free dcpe were also observed.

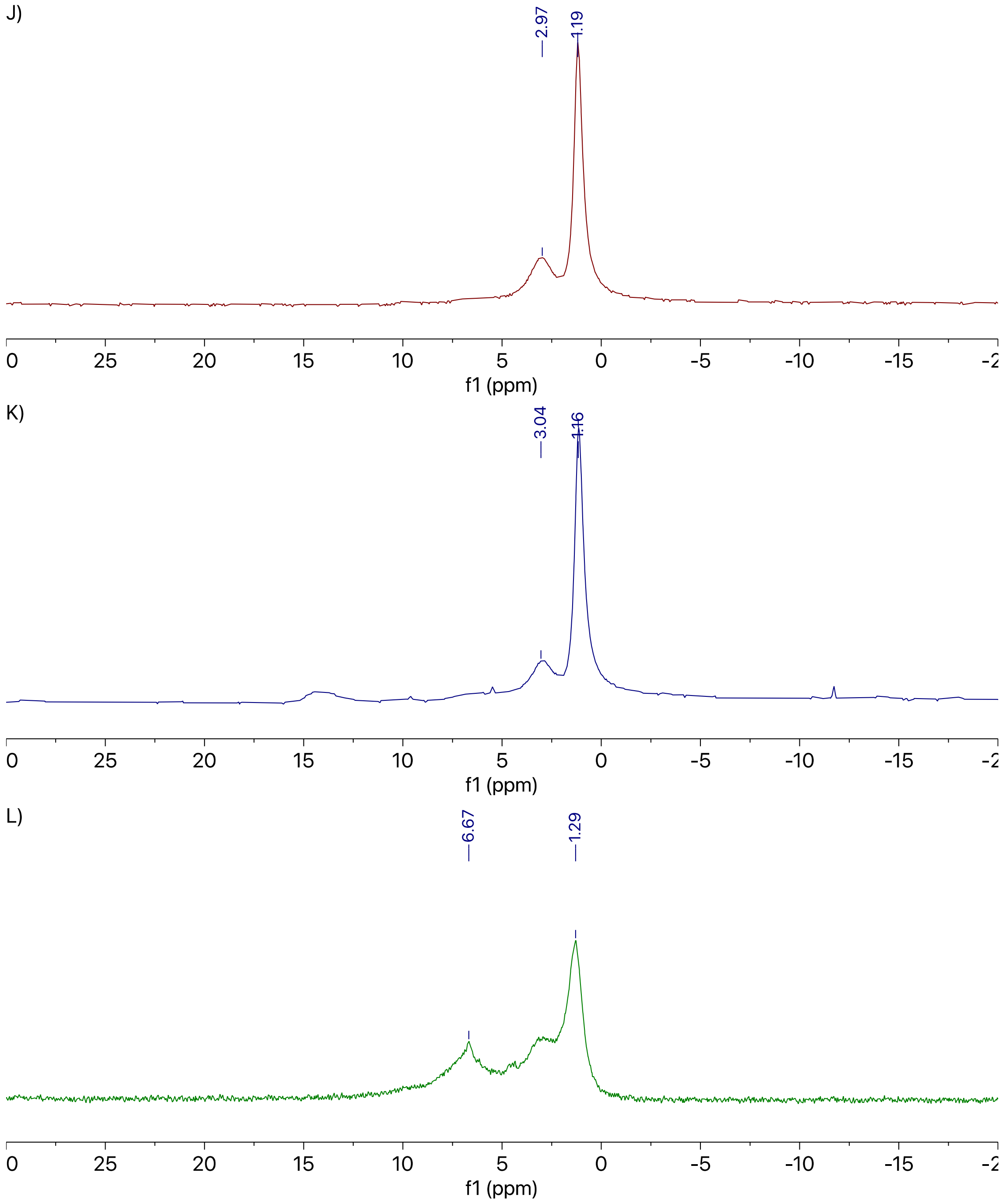

Figure S17. ^{31}P NMR spectra of J) CuCl + dcpe (2.0 equiv) + LiO^{t}Bu, K) CuCl + dcpe (2.0 equiv) + LiO^{t}Bu + B_2pin_2 and L) CuCl + dcpe (2.0 equiv) + LiO^{t}Bu + B_2pin_2 + phenylallene.

14. DFT calculations

14.1. Computational details

All electronic structure calculations were performed using the Gaussian 16 software package[11] at the CESGA facilities. The geometries of all intermediates and transition states involved were optimized at the B3LYP[12] level with Grimme D3 dispersion[13] and using the basis set SDD to describe the electrons of the copper atom[14] and the basis set 6-31G(d) for all other atoms (BS1).[15] Frequency calculations were performed at the same level to evaluate the zero-point vibrational energy and thermal corrections at 298 K and to confirm the nature of the stationary points, yielding one imaginary frequency for the transition states and none for the minima. Each transition state was further confirmed by following the steepest descent to both sides and identifying the minima present in the reaction energy profile. Single-point energies were calculated using B3LYP with the addition of Grimme D3 dispersion within self-consistent reaction field (SCRF) using the SMD model (THF);[16] the basis set SDD to describe the electrons of the copper atom, and the basis set 6-311++G(d,p) for all other atoms (BS2).[17] The resulting energies were used to correct the gas phase energies obtained from B3LYP-D3 and 6-31G(d)/SDD calculations.

---

[11] Frisch, M. J.; Trucks, G. W.; Schlegel, H. B.; Scuseria, G. E.; Robb, M. A.; Cheeseman, J. R.; Scalmani, G.; Barone, V.; Petersson, G. A.; Nakatsuji, H.; Li, X.; Caricato, M.; Marenich, A. V.; Bloino, J.; Janesko, B. G.; Gomperts, R.; Mennucci, B.; Hratchian, H. P.; Ortiz, J. V.; Izmaylov, A. F.; Sonnenberg, J. L.;Williams-Young, D.; Ding, F.; Lipparini, F.; Egidi, F.; Goings, J.; Peng, B.; Petrone, A.; Henderson, T.; Ranasinghe, D.; Zakrzewski, V. G.; Gao, J.; Rega, N.; Zheng, G.; Liang, W.; Hada, M.; Ehara, M.; Toyota, K.; Fukuda, R.; Hasegawa, J.; Ishida, M.; Nakajima, T.; Honda, Y.; Kitao, O.; Nakai, H.; Vreven, T.; Throssell, K.; Montgomery, J. A.; Jr.; Peralta, J. E.; Ogliaro, F.; Bearpark, M. J.; Heyd, J. J.; Brothers, E. N.; Kudin, K. N.; Staroverov, V. N.; Keith, T. A.; Kobayashi, R.; Normand, J.; Raghavachari, K.; Rendell, A. P.; Burant, J. C.; Iyengar, S. S.; Tomasi, J.; Cossi, M.; Millam, J. M.; Klene, M.; Adamo, C.; Cammi, R.; Ochterski, J. W.; Martin, R. L.; Morokuma, K.; Farkas, O.; Foresman, J. B.; Fox, D. J. *Gaussian 16, Revision B.01*, Gaussian, Inc., Wallingford CT, 2016.

[12] (a) Parr, R. G.; Wang, Y.*Density Functional Theory of Atoms andMolecules*; Oxford University Press: New York, 1989. (b) Becke, A. D. *J. Chem. Phys.* **1993**, *98*, 5648−5652. (c) Miehlich, B.; Savin, A.; Stoll, H.; Preuss, H. *Chem. Phys. Lett.* **1989**, *157*, 200−206. (d) Lee, C.;Yang, W.; Parr, R. G. *Phys. Rev. B: Condens. Matter Mater. Phys.* **1988**, 37, 785−789.

[13] Grimme, S.; Antony, J.; Ehrlich, S.; Krieg, H. *J. Chem. Phys.* **2010**, *132*, 154104–154119.

[14] (a) Dolg, M.; Wedig, U.; Stoll, H.; Preuss, H. *J. Chem. Phys.* **1987**, *86*, 866–872. (b) Martin, J. M. L.; Sundermann, A. . *J. Chem. Phys.* **2001**, *114*, 3408–3420.

[15] (a) Dill, J. D.; Pople, J. A. *J. Chem. Phys.* **1975**, *62*, 2921–2923. (b) Francl, M. M.; Pietro, W. J.; Hehre, W. J.; Binkley, J. S.; Gordon, M. S.; DeFrees, D. J.; Pople, J. A. *J. Chem. Phys.* **1982**, *77*, 3654–3665. (c) Hariharan, P. C.; Pople, J. A. *Theor. Chim. Acta* **1973**, *28*, 213–222. (d) Hehre, W. J.; Ditchfield, R.; Pople, J. A. *J. Chem. Phys.* **1972**, *56*, 2257–2261.

[16] Marenich, A. V.; Cramer, C. J.; Truhlar, D. G. *J. Phys. Chem. B* **2009**, *113*, 6378–6396.

[17] (a) Krishnan, R.; Binkley, J. S.; Seeger, R.; Pople, J. A. *J. Chem. Phys.* **1980**, *72*, 650–654. (b) McLean, A. D.; Chandler, G. S. *J. Chem. Phys.* **1980**, *72*, 5639–5648. (c) Clark, T.; Chandrasekhar, J.; Spitznagel, G. W.; Schelyer, P. v. R. *J. Comput. Chem.* **1983**, *4*, 294–301. (d) Francl, M. M.; Pietro, W. J.; Hehre, W. J.; Binkley, J. S.; Gordon, M. S.; DeFrees, D. J.; Pople, J. A. *J. Chem. Phys.* **1982**, *77*, 3654–3665. (e) Spitznagel, G. W.; Clark, T.; Schleyer, P. v. R.; Hehre, W. J. *J. Comput. Chem.* **1987**, *8*, 1109–1116.

14.2. Free energy profiles for the pathways associated to the Cu/dcpe system (Figure S18)

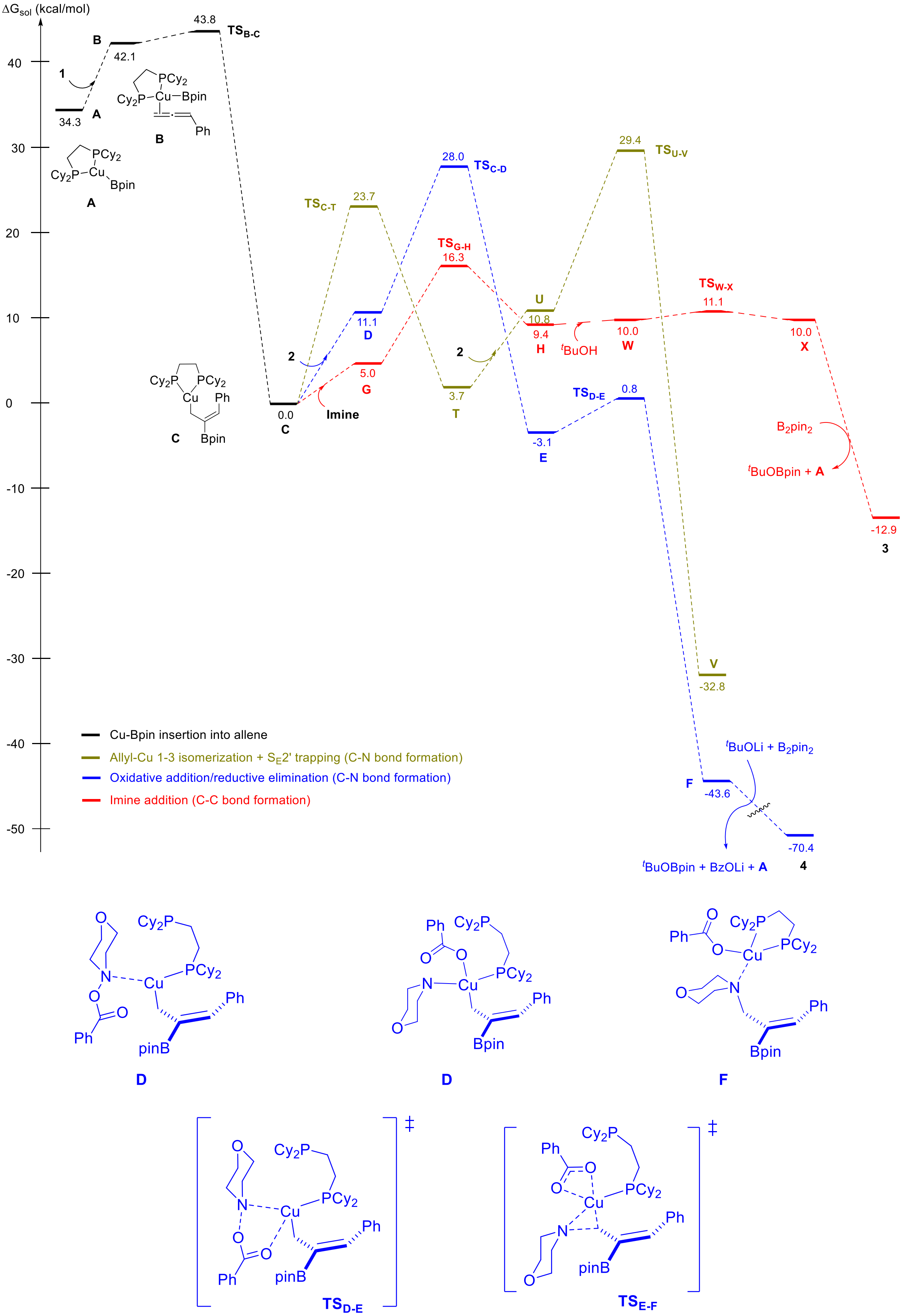

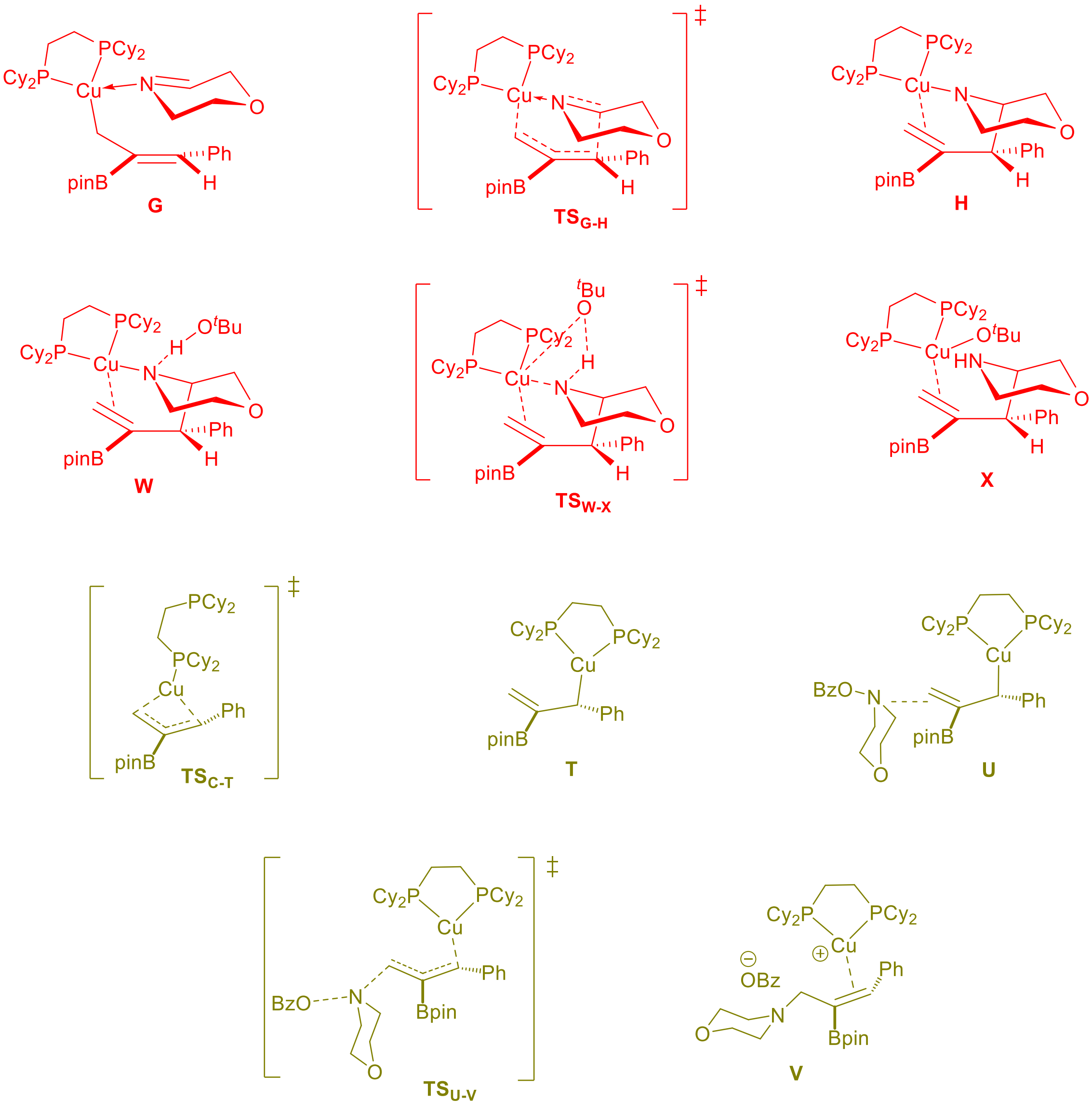

Figure S18. Free energy profile computed at the B3LYP-D3/6-311++G(d,p)-SDD$_{THF(SMD)}$//B3LYP-D3/6-31G(d)-SDD level of the possible pathways for Cu/dcpe system. Energies are relative to complex **C** combined with those of the relevant substrates.

14.3. Evaluation of explicit solvent molecules for calculations involving the phosphine-free Cu system (Figure S19)

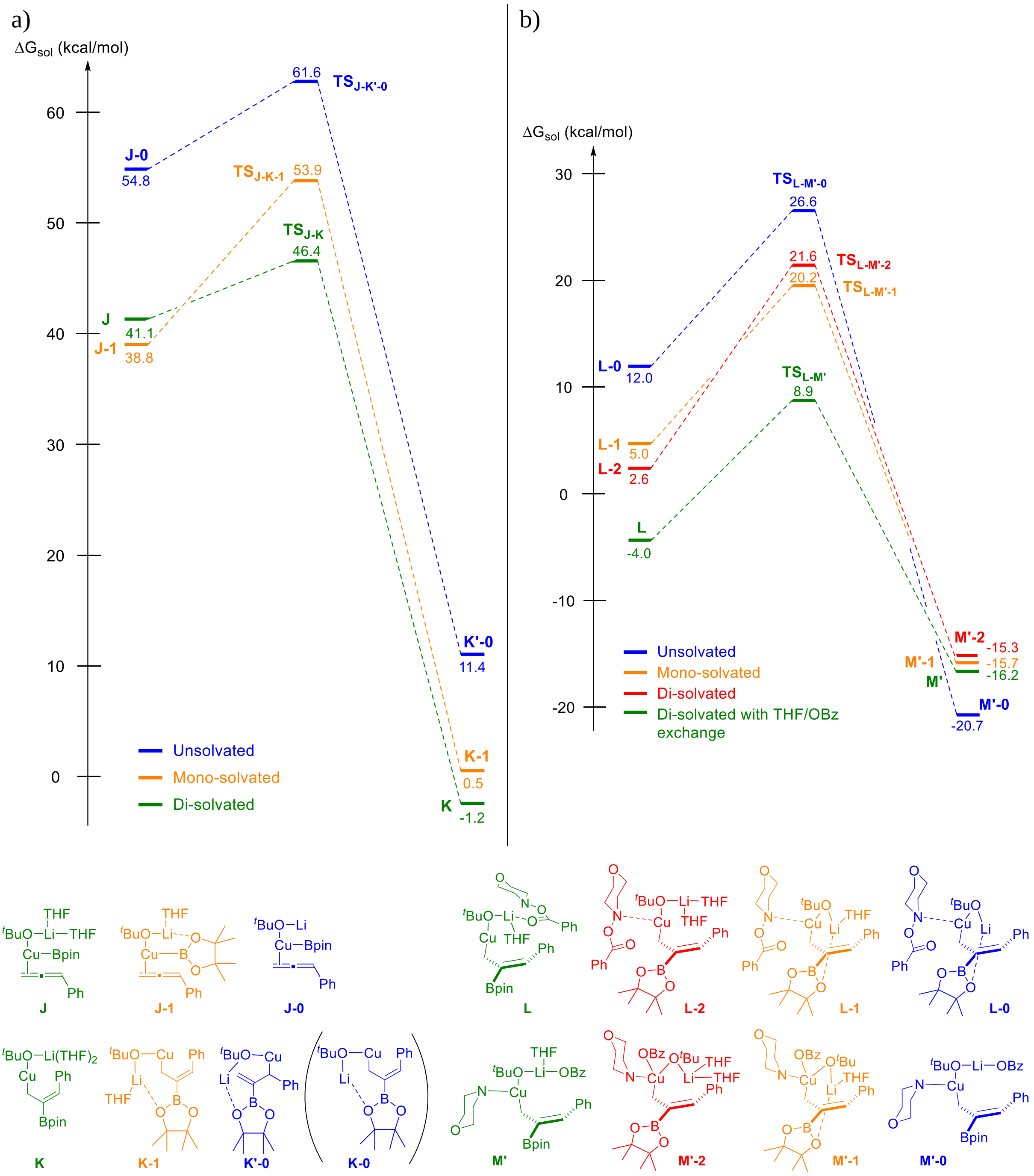

Figure S19. Free energy profile computed at the B3LYP-D3/6-311++G(d,p)-SDD$_{THF(SMD)}$//B3LYP-D3/6-31G(d)-SDD level of the a) Cu-Bpin insertion and b) oxidative addition of **2** into allyl-copper intermediate, for the phosphine-free Cu system featuring different solvation modes. Energies are relative to complex **C** combined with those of the relevant substrates.

Note: Two different pathways were found from di-solvated intermediate **K**. One involves coordination of morpholino benzoate **2** to the copper atom to afford complex **L-2** (Figure S19b, red pathway), and the other occurs through coordination of **2** to the Li atom with displacement of one THF molecule to generate intermediate **L** (Figure S19b, green pathway).

Note: Allene insertion into complex **J-0** leads preferentially to the branched allylcopper species **K'-0** which isomerizes to linear complex **K-0** through **TS$_{K'-K-0}$** ($\Delta G^{\ddagger}$ = 11.0 kcal/mol).

14.4. Free energy profiles for the pathways associated to the phosphine-free Cu system (Figure S20)

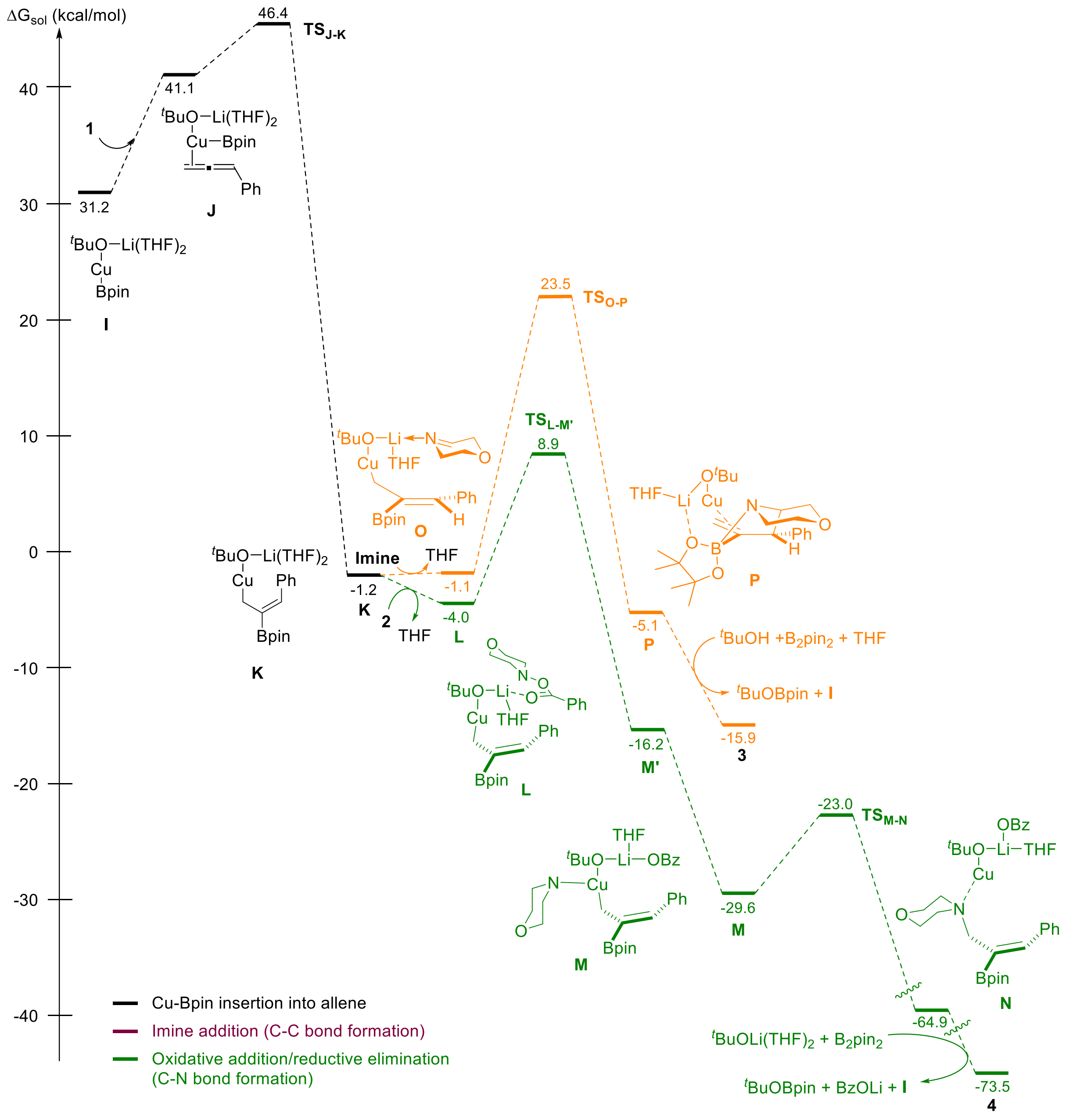

Figure S20. Free energy profile computed at the B3LYP-D3/6-311++G(d,p)-SDD$_{THF(SMD)}$//B3LYP-D3/6-31G(d)-SDD level of the possible pathways for the phosphine-free Cu system. Energies are relative to complex **C** combined with those of the relevant substrates.

Note: The step **M'-M** just involves a conformational change.

14.5. Free energy profile for the Cu/LiO^{t}Bu/P(O)Ph$_3$ system (Figure S21)

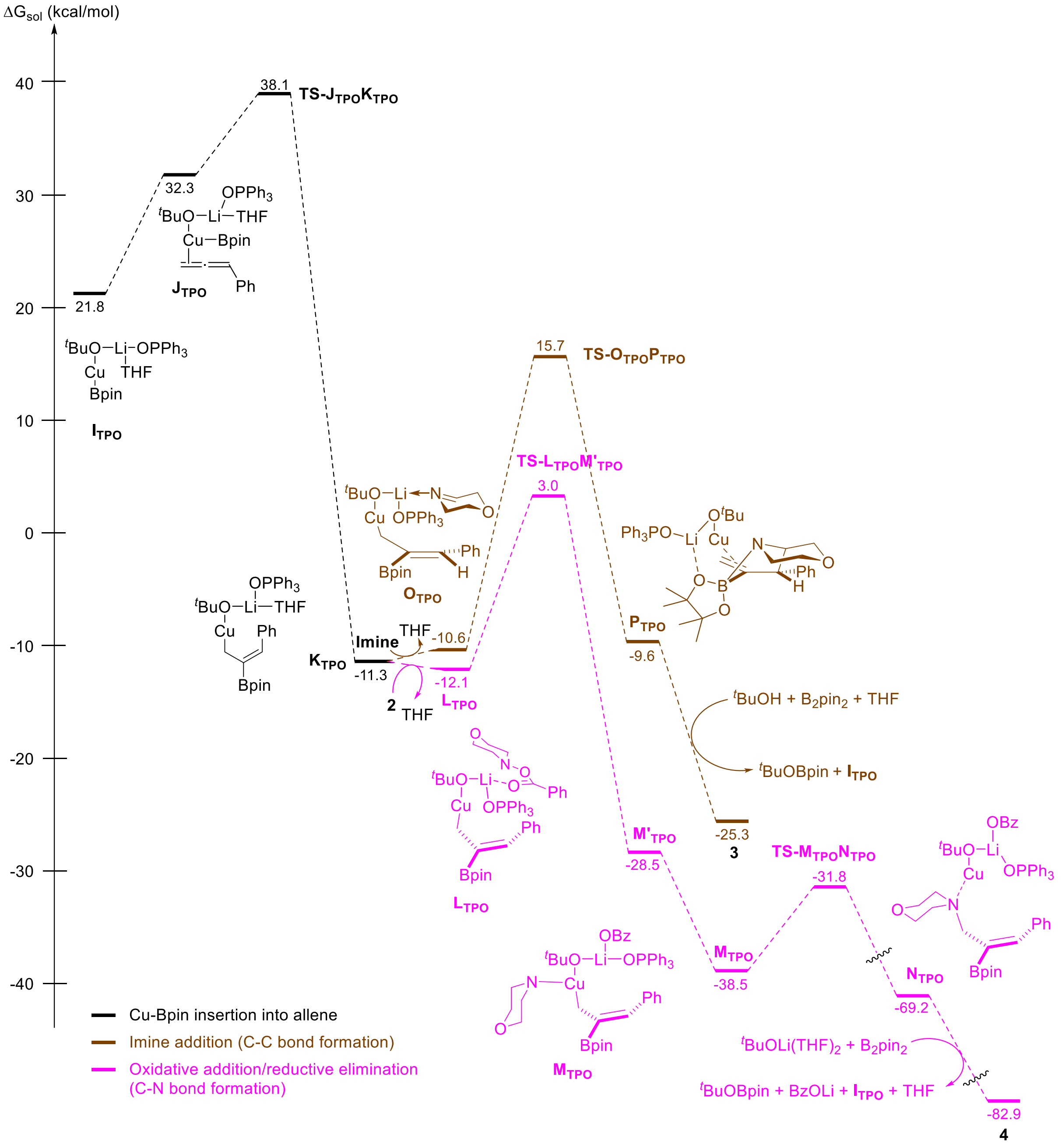

Figure S21. Free energy profile computed at the B3LYP-D3/6-311++G(d,p)-SDD$_{THF(SMD)}$//B3LYP-D3/6-31G(d)-SDD level of the possible pathways for the Cu/LiO^{t}Bu/P(O)Ph$_3$ system. Energies are relative to complex **C** combined with those of the relevant substrates.

Note: The step **M'$_{TPO}$-M$_{TPO}$** just involves a conformational change.

14.6. Free energy profiles for the addition of allyl copper complex C into alicyclic imine derived from morpholino benzoate 2 (Figure S22)

The observed *anti* diastereoselectivity of the reaction may arise from the addition of *Z*-allyl copper **B** through either the six-membered chair transition structure **TS$_{F\text{-}G}$**, in which copper interacts with the imine nitrogen atom, or through the half-chair-like structure **TS$_{F'\text{-}G'}$**, in which the imine nitrogen atom interacts with boron (Figure S21). The computational studies clearly show that the imine trapping takes place through six-membered chair structure **TS$_{F\text{-}G}$** (red pathway) since the half-chair-like transition structure involving coordination of the imine nitrogen atom to boron (purple pathway) was found to be much higher in energy.

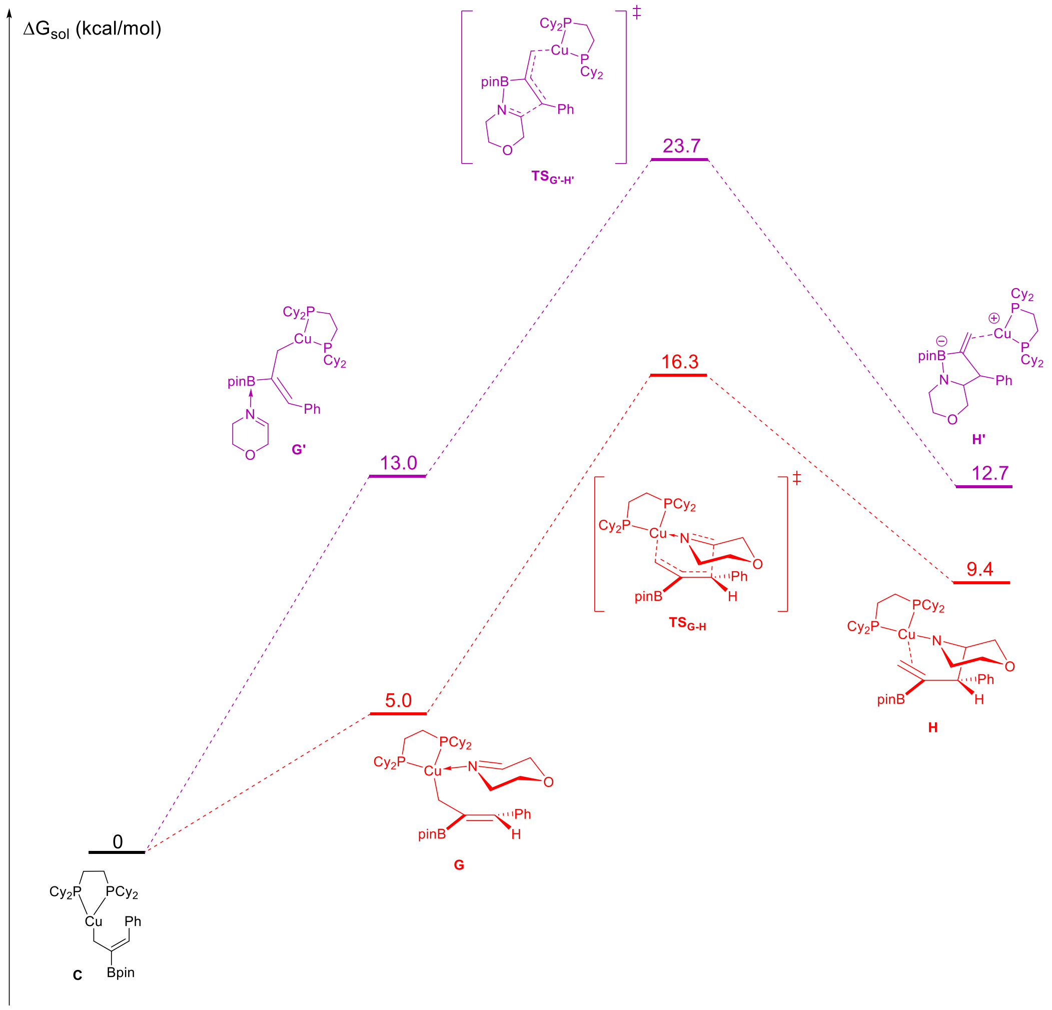

Figure S22. Free energy profile computed at the B3LYP-D3/6-311++G(d,p)-SDD$_{THF(SMD)}$//B3LYP-D3/6-31G(d)-SDD level for possible diastereomeric pathways for the imine trapping step. Energies are relative to complex **C** combined with those of the relevant substrates.

14.7. Free energy profile for the formation of the imine derived from morpholino benzoate 2 (Figure S23)

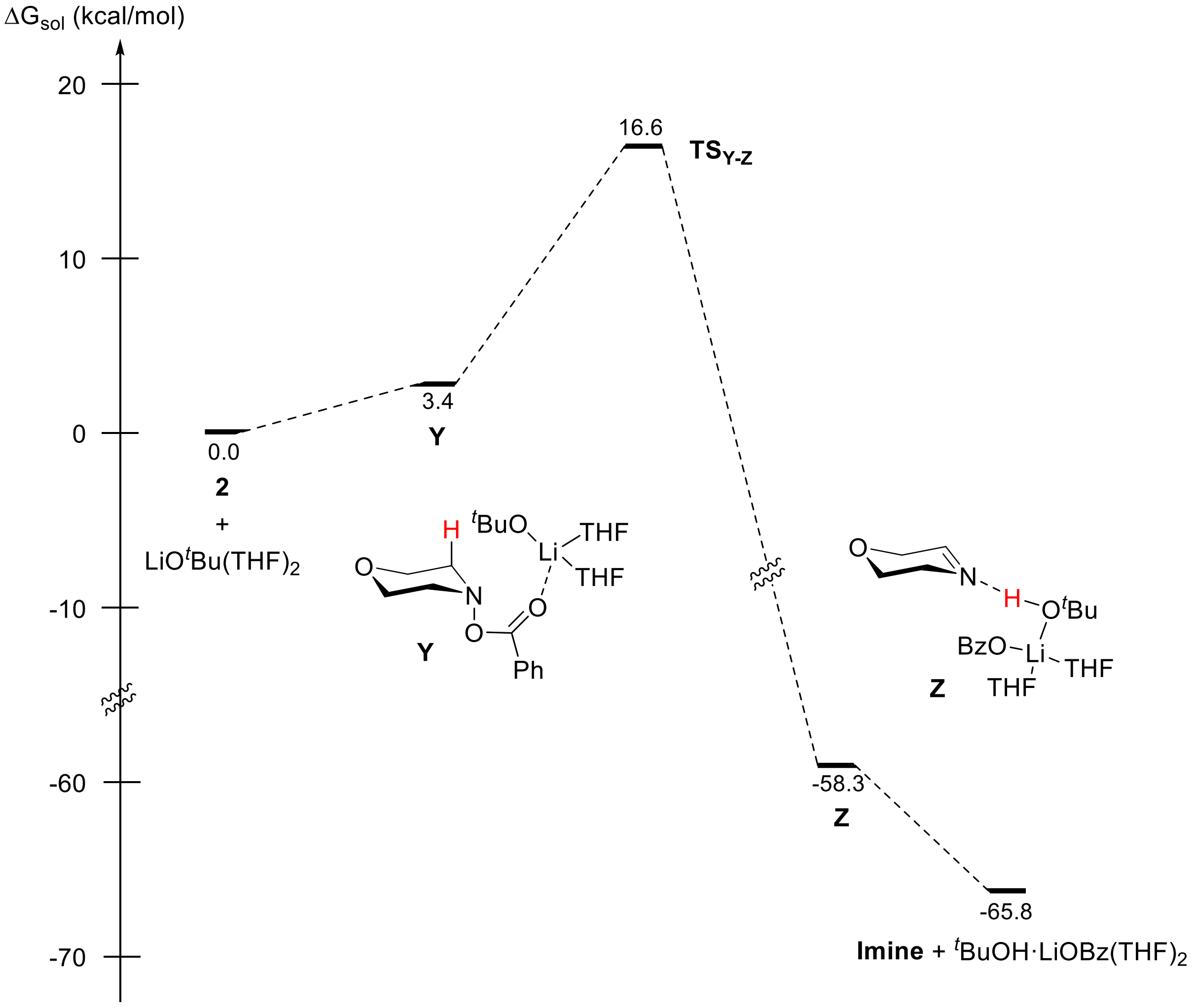

Figure S23. Free energy profile computed at the B3LYP-D3/6-311++G(d,p)-SDD$_{THF(SMD)}$//B3LYP-D3/6-31G(d)-SDD level for the formation of the imine form morpholino benzoate **2**.

14.8. Optimized transition states for the formation of compound 16: Explanation of the diastereocontrol over the three stereocenters

Reaction between allene (**28**), B_2pin_2 and 4-methylpiperidin-1-yl benzoate (**33**) led to the exclusive formation of **16** featuring three stereocenters (Scheme S3).

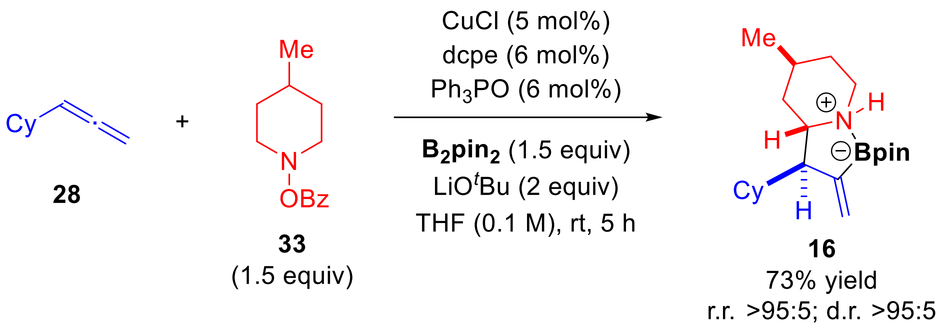

Scheme S3

As shown in Figure S23, the relative energy for transition state **TS1** (pathway to the 3*R**,3a*S**,5*S** diastereomer) is 3.9 kcal/mol lower than of **TS2** (pathway to the 3*R**,3a*S**,5*R** diastereomer), consistent with the observed diastereoselectivity. This difference in energy is likely due to the repulsive interaction between Me substituent in the piperidine ring with the allyl unit that is engendered in **TS2**.

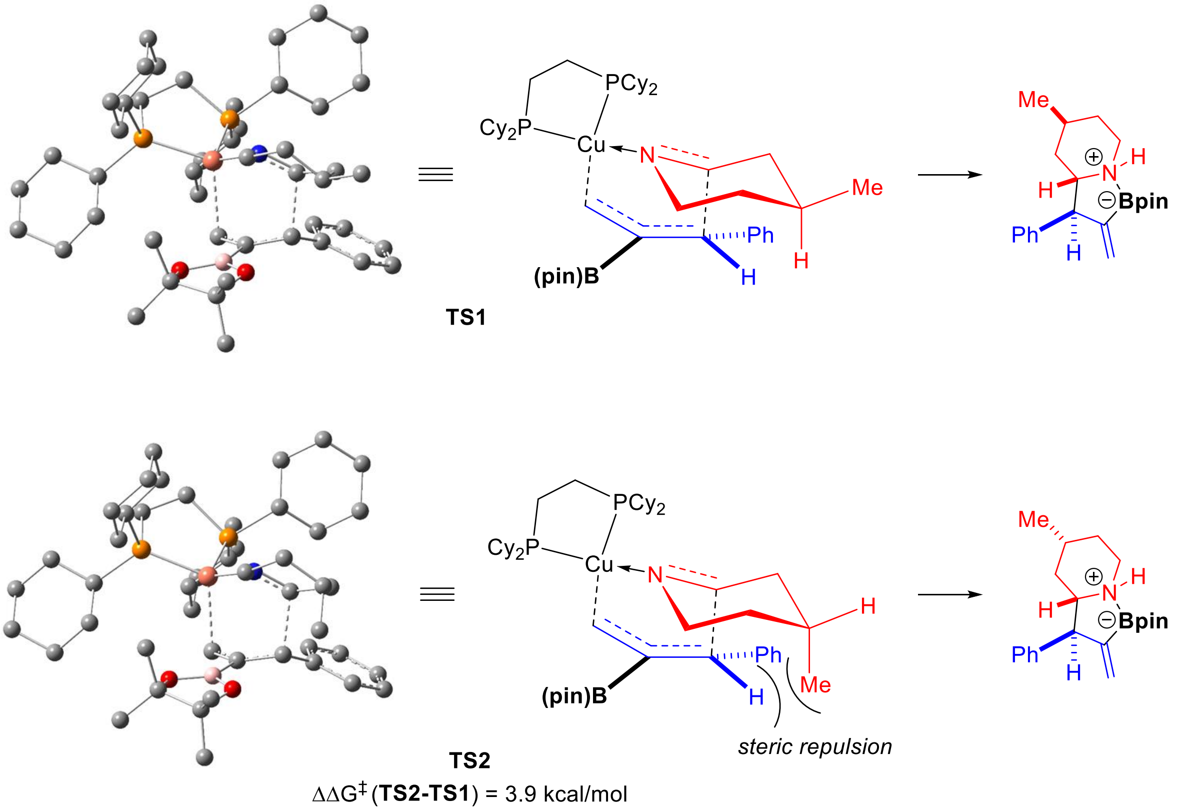

Figure S24. Optimized transition states for the formation of **16**. Energies are relative to **TS1**. Hydrogen atoms are omitted for clarity.

14.9. Cartesian coordinates in Å for all calculated structures

14.9.1. Cartesian coordinates for common species

1

Electronic Energy BS1 = -347.727276989 Hartree
Electronic Energy BS2 = -347.829047998 Hartree
Zero-point Energy Correction = 0.137981 Hartree
Thermal Correction to Enthalpy = 0.146616 Hartree
Thermal Correction to Free Energy = 0.105364 Hartree

Chemical symbol X, Y, Z

C	1.420602	-0.926449	0.000106
C	0.044595	-0.395661	0.000040
H	1.516042	-2.013423	0.000691
C	-0.224232	0.984615	0.000108
C	-1.038502	-1.288910	-0.000055
C	-1.534986	1.451201	0.000059
H	0.606284	1.685481	0.000227
C	-2.352244	-0.820103	-0.000132
H	-0.846124	-2.359506	-0.000093
C	-2.606558	0.551825	-0.000079
H	-1.724105	2.521612	0.000204
H	-3.176638	-1.528395	-0.000282
H	-3.629094	0.919351	-0.000076
C	2.523802	-0.211122	0.000330
C	3.612571	0.509298	-0.000298
H	4.091875	0.823838	0.926304
H	4.091472	0.822872	-0.927441

2

Electronic Energy BS1 = -707.392966226 Hartree
Electronic Energy BS2 = -707.607835325 Hartree
Zero-point Energy Correction = 0.230990 Hartree
Thermal Correction to Enthalpy = 0.244478 Hartree
Thermal Correction to Free Energy = 0.190618 Hartree

Chemical symbol X, Y, Z

N	-1.661375	-0.029335	0.295295
C	-2.329951	1.255614	0.503121
C	-2.220750	-0.713947	-0.876986
C	-3.816165	0.969180	0.735611
H	-2.211200	1.921006	-0.367805
H	-1.888842	1.738667	1.380606
C	-3.709793	-0.942796	-0.607464
H	-1.701979	-1.668334	-0.992283
H	-2.091095	-0.110077	-1.789396
H	-3.936922	0.377685	1.657596
H	-4.367048	1.907654	0.845013

O	-4.385480	0.285932	-0.371572
H	-3.827240	-1.618060	0.255066
H	-4.184346	-1.400509	-1.479853
O	0.301540	-1.795024	0.563987
C	0.610574	-0.665480	0.255224
O	-0.285396	0.330069	-0.010796
C	2.011933	-0.184550	0.077476
C	2.324489	1.142196	-0.252414
C	3.041094	-1.118523	0.260057
C	3.656603	1.525709	-0.398069
H	1.527000	1.862926	-0.393344
C	4.370459	-0.730447	0.114425
H	2.776040	-2.139030	0.516289
C	4.679886	0.592030	-0.215288
H	3.896967	2.553947	-0.653868
H	5.165333	-1.457150	0.257309
H	5.717376	0.894882	-0.329502

B_2pin_2

Electronic Energy BS1 = -822.591503553 Hartree
Electronic Energy BS2 = -822.839103640 Hartree
Zero-point Energy Correction = 0.366256 Hartree
Thermal Correction to Enthalpy = 0.386729 Hartree
Thermal Correction to Free Energy = 0.319103 Hartree

Chemical symbol X, Y, Z

B	0.851142	0.000335	-0.000332
O	1.613421	-0.674910	-0.924711
O	1.613322	0.675375	0.924238
C	2.997812	-0.628420	-0.471301
C	2.997859	0.628404	0.471319
C	3.905439	-0.523494	-1.693171
C	3.256514	-1.944447	0.272392
C	3.905016	0.523160	1.693503
C	3.257293	1.944341	-0.272270
H	4.951953	-0.407861	-1.388402
H	3.822481	-1.435668	-2.292363
H	3.628946	0.321649	-2.326967
H	3.034932	-2.779414	-0.399015
H	4.298787	-2.025612	0.598188
H	2.606763	-2.032951	1.148491
H	4.951590	0.407132	1.389091
H	3.822192	1.435377	2.292649
H	3.627987	-0.321871	2.327213
H	3.035781	2.779381	0.399068
H	4.299708	2.025135	-0.597708
H	2.607873	2.033071	-1.148590
B	-0.851141	0.000306	-0.000369
O	-1.613644	0.675319	-0.924692

O	-1.613100	-0.674954	0.924266
C	-2.998026	0.628370	-0.471266
C	-2.997644	-0.628449	0.471359
C	-3.905617	0.523137	-1.693131
C	-3.257153	1.944317	0.272417
C	-3.904843	-0.523507	1.693542
C	-3.256649	-1.944472	-0.272234
H	-4.952089	0.407151	-1.388355
H	-3.822971	1.435337	-2.292327
H	-3.628840	-0.321916	-2.326923
H	-3.035825	2.779354	-0.398988
H	-4.299457	2.025156	0.598196
H	-2.607444	2.033022	1.148526
H	-4.951458	-0.407835	1.389132
H	-3.821710	-1.435693	2.292691
H	-3.628101	0.321619	2.327252
H	-3.034880	-2.779439	0.399111
H	-4.299034	-2.025597	-0.597687
H	-2.607188	-2.033004	-1.148544

dcpe

Electronic Energy BS1 = -1702.51052313 Hartree
Electronic Energy BS2 = -1702.85585756 Hartree
Zero-point Energy Correction = 0.707399 Hartree
Thermal Correction to Enthalpy = 0.738165 Hartree
Thermal Correction to Free Energy = 0.644589 Hartree

Chemical symbol X, Y, Z

P	-2.172460	-0.200067	-1.086599
C	-0.780655	-0.495247	0.134753
C	-2.562844	1.637637	-0.813079
C	-3.599346	-1.150141	-0.287948
C	0.529316	0.160576	-0.333006
H	-0.653424	-1.581307	0.207209
H	-1.036191	-0.147703	1.141756
C	-3.988396	1.961974	-1.308977
C	-2.316525	2.271756	0.571584
H	-1.866829	2.128584	-1.512209
C	-3.375660	-2.662425	-0.522460
C	-3.932531	-0.882124	1.192502
H	-4.476086	-0.860105	-0.887419
H	0.770471	-0.157996	-1.355446
H	0.401178	1.249419	-0.374227
P	1.980171	-0.185304	0.825948
C	-4.246705	3.476922	-1.337021
H	-4.722019	1.491733	-0.638718
H	-4.152485	1.531925	-2.305826
C	-2.574317	3.788788	0.537723
H	-2.974811	1.814914	1.319812

H	-1.290640	2.086356	0.908953
C	-4.574505	-3.498396	-0.046683
H	-2.481114	-2.989248	0.028220
H	-3.177173	-2.853714	-1.584128
C	-5.127103	-1.727603	1.669453
H	-3.057255	-1.115229	1.814452
H	-4.156553	0.177678	1.348440
C	2.965530	1.407227	0.534559
C	2.893996	-1.482378	-0.204303
C	-3.989865	4.110375	0.037736
H	-5.275005	3.677038	-1.664939
H	-3.582324	3.941692	-2.080332
H	-2.413730	4.217814	1.535331
H	-1.839555	4.262897	-0.130276
C	-4.894296	-3.225063	1.429358
H	-4.373435	-4.565881	-0.204808
H	-5.453132	-3.248747	-0.659883
H	-5.320119	-1.534594	2.732807
H	-6.029080	-1.411674	1.124057
C	4.060668	1.631623	1.598452
C	3.527046	1.623349	-0.883311
H	2.202010	2.183723	0.707815
C	4.273036	-1.823028	0.394946
C	2.036372	-2.759986	-0.334791
H	3.046235	-1.078989	-1.216440
H	-4.142908	5.196219	-0.006147
H	-4.723456	3.717490	0.757731
H	-5.770770	-3.804626	1.746210
H	-4.050874	-3.563354	2.049776
C	4.704047	3.020694	1.450528
H	4.842813	0.867277	1.498592
H	3.637199	1.512454	2.603216
C	4.170949	3.013031	-1.026396
H	4.288156	0.859649	-1.094993
H	2.736599	1.495583	-1.633559
C	4.990757	-2.924835	-0.402137
H	4.142623	-2.153525	1.435917
H	4.906428	-0.929816	0.428099
C	2.761067	-3.858684	-1.129790
H	1.795188	-3.138848	0.669599
H	1.082385	-2.532376	-0.824169
C	5.258814	3.235310	0.034502
H	5.499189	3.148632	2.196332
H	3.947517	3.791221	1.661904
H	4.588792	3.132533	-2.034522
H	3.393431	3.783345	-0.914377
C	4.128477	-4.188506	-0.517090
H	5.954502	-3.158006	0.068746
H	5.216422	-2.549180	-1.411378
H	2.133699	-4.758085	-1.177909

H	2.902378	-3.518091	-2.166248
H	5.685806	4.241780	-0.060810
H	6.081668	2.526060	-0.140980
H	4.643068	-4.951368	-1.115154
H	3.981573	-4.618066	0.485071

Imine

Electronic Energy BS1 = -286.582718614 Hartree
Electronic Energy BS2 = -286.676670387 Hartree
Zero-point Energy Correction = 0.111001 Hartree
Thermal Correction to Enthalpy = 0.117062 Hartree
Thermal Correction to Free Energy = 0.082613 Hartree

Chemical symbol X, Y, Z

N	-0.520256	1.402259	-0.013752
C	-1.395165	0.231418	-0.156166
C	0.727462	1.174032	0.078666
C	-0.744762	-1.068711	0.307241
H	-1.681830	0.155589	-1.213263
H	-2.314337	0.425499	0.410764
C	1.402201	-0.179546	0.030226
H	1.395290	2.032315	0.212661
H	-0.626353	-1.062679	1.404395
H	-1.342678	-1.942257	0.031298
O	0.523559	-1.228985	-0.324736
H	1.882834	-0.371908	1.009281
H	2.201977	-0.163657	-0.720782

^{t}BuOH

Electronic Energy BS1 = -233.681480191 Hartree
Electronic Energy BS2 = -233.770192536 Hartree
Zero-point Energy Correction = 0.136578 Hartree
Thermal Correction to Enthalpy = 0.144110 Hartree
Thermal Correction to Free Energy = 0.107732 Hartree

Chemical symbol X, Y, Z

C	-0.005031	0.000004	0.014455
C	0.690919	1.264826	-0.509239
H	0.209800	2.157398	-0.096269
H	1.747933	1.276792	-0.211816
H	0.652304	1.319416	-1.603346
C	-1.488233	-0.000107	-0.359948
H	-1.615451	-0.000089	-1.447792
H	-1.980664	-0.887008	0.051857
H	-1.980838	0.886677	0.051901
C	0.691125	-1.264675	-0.509327
H	1.748116	-1.276518	-0.211813
H	0.210097	-2.157352	-0.096477

H	0.652604	-1.319156	-1.603439
O	0.010437	-0.000049	1.452424
H	0.939923	-0.000057	1.732152

^{t}BuOBpin

Electronic Energy BS1 = -644.443978209 Hartree
Electronic Energy BS2 = -644.637892116 Hartree
Zero-point Energy Correction = 0.311239 Hartree
Thermal Correction to Enthalpy = 0.328078 Hartree
Thermal Correction to Free Energy = 0.269843 Hartree

Chemical symbol X, Y, Z

C	-2.714877	-0.037508	0.005162
C	-3.919283	-0.916055	-0.341739
H	-4.856120	-0.385731	-0.139516
H	-3.893605	-1.192666	-1.400738
H	-3.900607	-1.835388	0.252480
C	-2.707513	0.309176	1.499222
H	-2.675329	-0.608715	2.096298
H	-1.837822	0.921789	1.750547
H	-3.614718	0.862763	1.766050
C	-2.696196	1.225787	-0.864579
H	-1.829805	1.847376	-0.624839
H	-2.649800	0.950642	-1.923963
H	-3.605697	1.814331	-0.700366
O	-1.563752	-0.861504	-0.306268
B	-0.275062	-0.475379	-0.172497
O	0.164881	0.732700	0.345942
O	0.757221	-1.302568	-0.561666
C	1.588785	0.809868	0.069981
C	1.980528	-0.710033	-0.058553
C	2.266654	1.560435	1.211493
C	1.745348	1.582072	-1.245622
C	3.107512	-1.003124	-1.044160
C	2.272487	-1.367399	1.296387
H	3.354707	1.560942	1.080921
H	1.923737	2.600004	1.225084
H	2.029385	1.114348	2.179650
H	1.259658	2.557454	-1.144105
H	2.798684	1.742433	-1.497412
H	1.266287	1.047001	-2.071606
H	4.029437	-0.493950	-0.740823
H	3.303357	-2.079688	-1.069235
H	2.846060	-0.685417	-2.055759
H	2.321677	-2.451536	1.158488
H	3.223577	-1.024682	1.716994
H	1.474773	-1.152761	2.014718

LiOBz

Electronic Energy BS1 = -427.836291681 Hartree
Electronic Energy BS2 = -427.978354604 Hartree
Zero-point Energy Correction = 0.106111 Hartree
Thermal Correction to Enthalpy = 0.115004 Hartree
Thermal Correction to Free Energy = 0.073011 Hartree

Chemical symbol	X,	Y,	Z
O	-2.216122	-1.113453	-0.000083
C	-1.586570	-0.000013	-0.000100
O	-2.216075	1.113530	-0.000131
C	-0.090572	0.000010	-0.000104
C	0.611587	-1.212118	-0.000083
C	0.611613	1.212135	-0.000055
C	2.005437	-1.211085	0.000045
H	0.048972	-2.139606	-0.000119
C	2.005465	1.211054	0.000054
H	0.048959	2.139590	-0.000064
C	2.703467	-0.000019	0.000104
H	2.548876	-2.152353	0.000093
H	2.548928	2.152309	0.000115
H	3.790637	-0.000027	0.000224
Li	-3.697122	-0.000107	0.000769

P(O)Ph$_3$

Electronic Energy BS1 = -1111.58625239 Hartree
Electronic Energy BS2 = -1111.83285827 Hartree
Zero-point Energy Correction = 0.279515 Hartree
Thermal Correction to Enthalpy = 0.297110 Hartree
Thermal Correction to Free Energy = 0.233241 Hartree

Chemical symbol	X,	Y,	Z
O	-0.000336	0.000512	2.442180
P	-0.000269	0.000368	0.939215
C	-1.555862	0.645928	0.229452
C	1.337006	1.024260	0.229575
C	0.218332	-1.669602	0.229635
C	-1.647526	1.224492	-1.044423
C	-2.706129	0.531370	1.022970
C	1.882237	0.814965	-1.045082
C	1.815333	2.076013	1.023805
C	0.891172	-2.609215	1.023549
C	-0.235481	-2.037393	-1.044974
C	-2.881510	1.662965	-1.526815
H	-0.756088	1.350392	-1.652804
C	-3.937065	0.974072	0.538843
H	-2.619383	0.109972	2.020273
C	2.879512	1.663564	-1.527716

H	1.543743	-0.019029	-1.653759
C	2.814896	2.919812	0.539517
H	1.408204	2.211020	2.021672
C	1.123293	-3.896432	0.538984
H	1.211615	-2.324112	2.021422
C	0.001801	-3.325109	-1.527803
H	-0.789050	-1.327656	-1.653666
C	-4.026671	1.535009	-0.737352
H	-2.947075	2.112744	-2.513958
H	-4.825324	0.885882	1.158604
C	3.343690	2.717718	-0.737546
H	3.300446	1.495963	-2.515531
H	3.184523	3.731917	1.159795
C	0.683777	-4.253607	-0.737993
H	1.642749	-4.622159	1.158958
H	-0.353821	-3.606045	-2.515563
H	-4.986098	1.880604	-1.113163
H	4.123169	3.375158	-1.113519
H	0.864289	-5.257136	-1.114179

3

Electronic Energy BS1 = -1046.30361151 Hartree
Electronic Energy BS2 = -1046.61066558 Hartree
Zero-point Energy Correction = 0.453561 Hartree
Thermal Correction to Enthalpy = 0.476797 Hartree
Thermal Correction to Free Energy = 0.403427 Hartree

Chemical symbol X, Y, Z

C	-0.294476	-0.084791	-1.321990
C	-1.534384	0.461207	-0.608245
H	-2.011752	1.228405	-1.231193
C	-2.576017	-0.583482	-0.252787
C	-3.930930	-0.372281	-0.528163
C	-2.191762	-1.774267	0.385923
C	-4.889895	-1.326583	-0.175568
H	-4.238159	0.544167	-1.027825
C	-3.147231	-2.724541	0.739042
H	-1.137823	-1.947267	0.592727
C	-4.500384	-2.504793	0.460076
H	-5.938248	-1.147330	-0.400572
H	-2.836374	-3.643495	1.229839
H	-5.242979	-3.249676	0.734065
C	-0.326045	-0.555044	-2.569901
H	-1.245556	-0.636862	-3.150960
H	0.586051	-0.889457	-3.059611
B	1.007286	0.086728	-0.404595
O	1.164814	-0.893064	0.644900
O	2.291220	0.363400	-0.967950
C	2.571026	-1.035949	0.897445

C	3.198224	-0.643374	-0.489109
C	2.837409	-2.471549	1.347115
C	2.975594	-0.064127	2.021031
C	4.603734	-0.049050	-0.417843
C	3.172849	-1.810593	-1.488856
H	3.912960	-2.662686	1.440785
H	2.372852	-2.646730	2.323697
H	2.411757	-3.187194	0.640606
H	2.305876	-0.213348	2.874713
H	4.005021	-0.229835	2.356613
H	2.901106	0.978559	1.692742
H	5.309477	-0.761784	0.024903
H	4.952199	0.193613	-1.427142
H	4.620204	0.871718	0.170769
H	3.411963	-1.422589	-2.484041
H	3.900934	-2.588766	-1.234298
H	2.175581	-2.258943	-1.529035
N	0.422014	1.526628	0.403259
C	0.562026	2.754549	-0.422287
C	-1.004568	1.159533	0.679207
C	-0.317500	3.878414	0.114566
H	0.260541	2.492993	-1.439995
H	1.622069	3.020085	-0.446032
C	-1.817337	2.375132	1.115792
H	-0.961792	0.426572	1.489409
H	0.044188	4.211018	1.104489
H	-0.298818	4.737782	-0.562103
O	-1.673240	3.458742	0.204692
H	-1.516050	2.694368	2.129113
H	-2.880572	2.118607	1.137237
H	0.920490	1.644943	1.285456

4

Electronic Energy BS1 = -1046.30504484 Hartree
Electronic Energy BS2 = -1046.60790394 Hartree
Zero-point Energy Correction = 0.453438 Hartree
Thermal Correction to Enthalpy = 0.477247 Hartree
Thermal Correction to Free Energy = 0.400528 Hartree

Chemical symbol X, Y, Z

B	-1.388430	-0.607073	-0.241997
O	-2.097294	-1.611039	0.381381
O	-2.195436	0.413863	-0.694614
C	-3.573572	-0.023146	-0.543192
C	-3.451028	-1.119594	0.578975
C	-3.503518	-0.537225	1.996866
C	-4.420164	-2.290154	0.449155
C	-4.429304	1.185755	-0.178145
C	-4.004852	-0.593736	-1.899440

H	-4.441048	1.893710	-1.012778
H	-4.035115	1.705141	0.697910
H	-5.461823	0.881357	0.027367
H	-3.396056	-1.462210	-2.169505
H	-3.862203	0.172793	-2.666987
H	-5.058410	-0.891585	-1.895234
H	-2.813179	0.306240	2.099102
H	-3.200481	-1.311021	2.708411
H	-4.511199	-0.197825	2.257648
H	-4.249464	-3.001609	1.263228
H	-4.283362	-2.820079	-0.495822
H	-5.457361	-1.941654	0.510949
C	0.152478	-0.590781	-0.405284
C	0.775297	0.560330	-1.173630
C	0.854533	-1.632282	0.105142
H	0.023571	0.961597	-1.876562
H	1.619714	0.194331	-1.772071
C	2.300787	-1.892322	0.095203
H	0.265862	-2.430450	0.559916
C	3.273193	-0.872675	0.112600
C	2.737477	-3.231834	0.093423
C	4.630306	-1.191913	0.092833
H	2.948738	0.161703	0.186031
C	4.093489	-3.546207	0.063382
H	1.998211	-4.029803	0.101204
C	5.047003	-2.524525	0.058993
H	5.367822	-0.393353	0.114768
H	4.407916	-4.586565	0.050456
H	6.106477	-2.766771	0.043895
N	1.300056	1.641165	-0.320219
C	1.901146	2.699938	-1.132379
C	0.296314	2.210799	0.583403
C	2.501939	3.772991	-0.227782
H	1.156368	3.169788	-1.804657
H	2.688678	2.261790	-1.758950
C	0.939254	3.296045	1.442420
H	-0.088011	1.416979	1.232639
H	-0.560159	2.632972	0.026645
H	3.330876	3.337083	0.355895
H	2.891072	4.606192	-0.821367
O	1.522162	4.319374	0.644372
H	1.705238	2.840158	2.092549
H	0.188967	3.783918	2.072464

BzOLi·THF$_2$·^{t}BuOH

Electronic Energy BS1 = -1126.55251772 Hartree
Electronic Energy BS2 = -1126.89102167 Hartree
Zero-point Energy Correction = 0.484991 Hartree
Thermal Correction to Enthalpy = 0.513948 Hartree

Thermal Correction to Free Energy = 0.422224 Hartree

Chemical symbol X, Y, Z

H	-0.932187	-1.463675	-0.537564
O	0.685341	-1.714133	-0.580865
C	1.239586	-0.577843	-0.566731
O	0.624569	0.527598	-0.417514
C	2.742872	-0.525403	-0.698900
C	3.476864	-1.707860	-0.856880
C	3.418522	0.700704	-0.647497
O	-1.872160	-1.130591	-0.365619
C	4.866505	-1.664695	-0.961302
H	2.937068	-2.648218	-0.894542
C	4.808690	0.744883	-0.750096
H	2.836535	1.608577	-0.527936
C	-2.666085	-2.217766	0.147163
C	5.535208	-0.438413	-0.907278
H	5.430088	-2.586144	-1.084356
H	5.326307	1.700071	-0.708703
C	-2.038113	-2.739672	1.447732
C	-2.715605	-3.323045	-0.915891
C	-4.058886	-1.643125	0.406866
H	6.618930	-0.405167	-0.987793
H	-1.011315	-3.072358	1.261956
H	-2.014744	-1.945148	2.201011
H	-2.610114	-3.583994	1.849687
H	-3.332638	-4.165683	-0.582991
H	-3.133514	-2.929744	-1.848832
H	-1.705854	-3.694668	-1.122683
H	-4.003501	-0.824940	1.134518
H	-4.490647	-1.255754	-0.522706
H	-4.730158	-2.411776	0.805037
Li	-1.130013	0.514941	0.302818
O	-2.015856	2.137326	-0.403262
C	-3.257742	1.859814	-1.080664
C	-1.185359	2.784616	-1.394145
C	-2.848212	1.371161	-2.480084
H	-3.797035	1.119401	-0.487502
H	-3.849330	2.785960	-1.126892
C	-1.447155	2.012289	-2.698069
H	-1.492351	3.837718	-1.470829
H	-0.154713	2.717585	-1.046581
H	-3.575711	1.670562	-3.240560
H	-2.771217	0.281410	-2.478940
H	-1.414987	2.671999	-3.570273
H	-0.685415	1.238642	-2.821632
O	-0.847575	0.666013	2.245885
C	-0.543714	2.010423	2.662951
C	0.253084	-0.169319	2.686567
C	0.958604	2.150369	2.413710
H	-1.170967	2.680233	2.069576

H	-0.794048	2.127117	3.728351
C	1.498909	0.744884	2.766810
H	-0.005604	-0.594334	3.664845
H	0.350242	-0.979633	1.962674
H	1.411960	2.947875	3.010250
H	1.133678	2.353092	1.354013
H	1.926619	0.722783	3.774273
H	2.272268	0.431681	2.062094

^{t}BuOLi(THF)$_2$

Electronic Energy BS1 = -705.635933863 Hartree
Electronic Energy BS2 = -705.852035059 Hartree
Zero-point Energy Correction = 0.365252 Hartree
Thermal Correction to Enthalpy = 0.386467 Hartree
Thermal Correction to Free Energy = 0.311226 Hartree

Chemical symbol X, Y, Z

O	0.123020	1.034346	0.000903
C	0.211810	2.407726	-0.009098
C	-1.162650	3.027312	-0.359667
C	1.247981	2.868775	-1.062065
C	0.651418	2.916418	1.383820
H	-1.491522	2.654676	-1.337820
H	-1.907605	2.723221	0.386374
H	-1.140962	4.124977	-0.395159
H	1.342835	3.961552	-1.118555
H	2.233553	2.451766	-0.819400
H	0.955342	2.494761	-2.051213
H	-0.072146	2.588144	2.140127
H	1.625719	2.483448	1.642603
H	0.733629	4.010719	1.432602
Li	-0.036449	-0.629331	-0.092584
O	1.501877	-1.721520	-0.550008
C	2.094561	-2.351170	0.616326
C	2.474073	-0.834494	-1.168962
C	3.514827	-1.776918	0.747973
H	1.469360	-2.103632	1.483764
H	2.078417	-3.436994	0.475619
C	3.421456	-0.458038	-0.037023
H	2.984229	-1.378140	-1.976043
H	1.919307	0.016548	-1.568151
H	4.243539	-2.449032	0.280066
H	3.807019	-1.637811	1.792846
H	4.391441	-0.100808	-0.395551
H	2.952312	0.326559	0.565842
O	-1.697029	-1.485373	0.422323
C	-2.480308	-2.064729	-0.651780
C	-2.484760	-0.479000	1.122070
C	-3.872349	-1.425083	-0.561125

H	-1.985077	-1.825518	-1.602520
H	-2.490691	-3.152496	-0.529298
C	-3.576418	-0.086142	0.133364
H	-2.896804	-0.931347	2.034100
H	-1.800768	0.337300	1.366290
H	-4.534673	-2.038713	0.060767
H	-4.339493	-1.311810	-1.543799
H	-4.452854	0.348555	0.623098
H	-3.175559	0.644498	-0.577588

THF

Electronic Energy BS1 = -232.456700173 Hartree
Electronic Energy BS2 = -232.532741598 Hartree
Zero-point Energy Correction = 0.117378 Hartree
Thermal Correction to Enthalpy = 0.123248 Hartree
Thermal Correction to Free Energy = 0.088866 Hartree

Chemical symbol X, Y, Z

C	-1.164577	-0.429506	0.134019
C	1.164381	-0.429708	-0.134511
C	-0.732049	0.996137	-0.231575
H	-1.529359	-0.475677	1.172309
H	-1.951863	-0.823512	-0.519677
C	0.732349	0.995858	0.231801
H	1.528093	-0.475640	-1.173223
H	-0.786533	1.145975	-1.316713
H	-1.345430	1.764005	0.249950
H	1.345906	1.763808	-0.249377
H	0.786912	1.145058	1.317002
O	-0.000075	-1.252081	0.000386
H	1.952254	-0.824047	0.518243

14.9.2. Cartesian coordinates for the Cu/dcpe system (Figures S18, S22 and S24)

A

Electronic Energy BS1 = -2311.19082506 Hartree
Electronic Energy BS2 = -2311.68344802 Hartree
Zero-point Energy Correction = 0.891573 Hartree
Thermal Correction to Enthalpy = 0.934767 Hartree
Thermal Correction to Free Energy = 0.814830 Hartree

Chemical symbol X, Y, Z

Cu	0.015256	-0.560033	-0.383326
P	2.201866	-0.347202	0.321915
C	2.114211	0.606503	1.936356
C	3.215714	-1.847161	0.787532
C	3.287977	0.669299	-0.830648

C	1.140830	1.806110	1.871155
H	3.103000	0.936639	2.273178
H	1.755922	-0.126595	2.668794
C	3.187558	-2.872433	-0.365886
C	4.659213	-1.601522	1.266047
H	2.644352	-2.280091	1.624551
C	2.407371	1.420673	-1.854653
C	4.281479	1.642143	-0.165561
H	3.867685	-0.083463	-1.386524
H	0.881376	2.139261	2.883965
H	1.637906	2.650134	1.377932
P	-0.400007	1.432338	0.849375
C	3.876073	-4.185297	0.039473
H	3.707878	-2.451452	-1.240064
H	2.150940	-3.052504	-0.673735
C	5.339218	-2.919548	1.676405
H	5.237347	-1.145933	0.449818
H	4.677518	-0.892766	2.103250
C	3.259835	2.148192	-2.904473
H	1.786659	2.156497	-1.323662
H	1.710860	0.721790	-2.334344
C	5.138089	2.366451	-1.218750
H	3.722165	2.393423	0.409882
H	4.926572	1.118629	0.547807
C	-0.820063	3.122310	0.153637
C	-1.697047	1.026201	2.138685
C	5.310996	-3.942224	0.530840
H	3.873956	-4.884636	-0.805940
H	3.293566	-4.660458	0.842595
H	6.372215	-2.724962	1.992426
H	4.815481	-3.337174	2.548977
C	4.263619	3.104182	-2.243441
H	2.610710	2.694046	-3.600642
H	3.806680	1.403550	-3.501706
H	5.823483	3.066781	-0.724288
H	5.764514	1.628191	-1.740984
C	-1.703635	3.003312	-1.107001
C	-1.395371	4.143451	1.152928
H	0.158737	3.501358	-0.182232
C	-3.072061	0.828690	1.463590
C	-1.267366	-0.260861	2.880545
H	-1.760848	1.849476	2.866185
H	5.771354	-4.885349	0.851203
H	5.918620	-3.562366	-0.304315
H	4.891188	3.587367	-3.002724
H	3.711994	3.907682	-1.732126
C	-1.914041	4.377453	-1.762249
H	-2.679554	2.578732	-0.839557
H	-1.248262	2.299444	-1.813961
C	-1.610082	5.512584	0.484654

H	-2.358558	3.778356	1.533786
H	-0.731514	4.245101	2.021434
C	-4.136254	0.319013	2.448340
H	-2.957715	0.095134	0.657567
H	-3.411480	1.767553	1.010996
C	-2.345936	-0.751509	3.858358
H	-1.078501	-1.048415	2.137095
H	-0.329649	-0.088426	3.423768
C	-2.495410	5.391511	-0.765041
H	-2.570918	4.279112	-2.635565
H	-0.948031	4.750813	-2.134785
H	-2.052946	6.214973	1.202511
H	-0.633445	5.929701	0.196715
C	-3.679414	-0.974985	3.133902
H	-5.079111	0.155037	1.910271
H	-4.336380	1.087662	3.210678
H	-2.008374	-1.678370	4.339748
H	-2.482031	-0.008639	4.659382
H	-2.615147	6.372279	-1.242371
H	-3.500956	5.062710	-0.462774
H	-4.444636	-1.329038	3.836657
H	-3.543619	-1.751702	2.370416
B	-1.531826	-1.646776	-1.014131
O	-2.460850	-2.220995	-0.122840
O	-2.003220	-1.808222	-2.321334
C	-3.459632	-2.949933	-0.877793
C	-3.378508	-2.257886	-2.279387
C	-4.806583	-2.820622	-0.168493
C	-3.018356	-4.420011	-0.904493
C	-3.637558	-3.175200	-3.472461
C	-4.260278	-1.003132	-2.381016
H	-5.608000	-3.276605	-0.762484
H	-4.767411	-3.332608	0.798955
H	-5.061523	-1.774392	0.017372
H	-2.894297	-4.768558	0.125880
H	-3.754575	-5.060776	-1.403284
H	-2.056067	-4.522981	-1.414868
H	-4.648571	-3.598818	-3.429349
H	-3.545589	-2.604688	-4.402740
H	-2.915503	-3.993950	-3.509269
H	-3.996411	-0.462090	-3.295252
H	-5.327236	-1.251128	-2.417609
H	-4.081952	-0.335474	-1.532086

B

Electronic Energy BS1 = -2658.93614109 Hartree
Electronic Energy BS2 = -2659.52451973 Hartree
Zero-point Energy Correction = 1.031988 Hartree
Thermal Correction to Enthalpy = 1.083794 Hartree

Thermal Correction to Free Energy = 0.944781 Hartree

Chemical symbol X, Y, Z			
Cu	0.040142	-0.472993	0.116480
C	1.236211	-2.215746	-0.312112
C	1.756199	-1.328123	0.606367
H	1.620456	-2.246783	-1.329391
C	2.810504	-0.875274	1.287081
H	2.716666	0.072040	1.811826
H	0.676845	-3.092025	0.011183
C	4.121793	-1.539087	1.378664
C	5.132871	-0.935509	2.149187
C	4.425746	-2.745179	0.718464
C	6.398625	-1.510110	2.257129
H	4.916052	-0.002597	2.665411
C	5.689411	-3.319596	0.827782
H	3.658208	-3.226636	0.120157
C	6.684592	-2.706662	1.596436
H	7.162435	-1.022719	2.858169
H	5.901583	-4.251806	0.309541
H	7.670263	-3.157144	1.678174
B	-0.007793	0.520660	1.855355
O	-0.854520	0.194314	2.917198
O	0.809382	1.592581	2.225344
C	-0.422331	0.912536	4.102779
C	0.336555	2.135577	3.485364
C	0.499278	-0.035227	4.881980
C	-1.651858	1.264513	4.935839
C	-0.589581	3.311003	3.142621
C	1.539095	2.628791	4.285177
H	0.837164	0.406931	5.825931
H	-0.049333	-0.955609	5.105007
H	1.374334	-0.303617	4.281952
H	-2.117956	0.346930	5.309867
H	-1.377040	1.884003	5.798032
H	-2.395952	1.800893	4.341898
H	-0.958679	3.816680	4.041651
H	-0.032788	4.035348	2.540474
H	-1.447021	2.970190	2.553357
H	2.010772	3.467027	3.761391
H	1.233331	2.975228	5.279645
H	2.287946	1.842350	4.403683
P	-2.016439	-1.097532	-0.690136
C	-1.943062	-0.762139	-2.527377
C	-2.387277	-2.921712	-0.570501
C	-3.577914	-0.226384	-0.091068
C	-1.370263	0.645136	-2.798005
H	-2.914570	-0.885149	-3.018231
H	-1.271077	-1.526116	-2.935567
C	-2.256241	-3.377216	0.899216
C	-3.705251	-3.430364	-1.182109

H	-1.552331	-3.370361	-1.131016
C	-3.241896	1.123989	0.576888
C	-4.692884	-0.034401	-1.140257
H	-3.962738	-0.890687	0.697939
H	-1.233001	0.806481	-3.873293
H	-2.086500	1.403383	-2.458895
P	0.224937	0.942676	-1.838336
C	-2.366870	-4.905363	1.017079
H	-3.054088	-2.912037	1.496940
H	-1.309853	-3.018880	1.320553
C	-3.808308	-4.961647	-1.068890
H	-4.551901	-2.978782	-0.646736
H	-3.790728	-3.122698	-2.231890
C	-4.489740	1.771278	1.195200
H	-2.813794	1.805008	-0.174240
H	-2.474967	0.974780	1.338331
C	-5.945305	0.598593	-0.509423
H	-4.328695	0.631134	-1.935932
H	-4.955557	-0.982330	-1.621051
C	0.118129	2.794394	-1.568518
C	1.592030	0.671077	-3.102076
C	-3.669318	-5.424777	0.389271
H	-2.302945	-5.205409	2.070436
H	-1.508863	-5.366694	0.505406
H	-4.761408	-5.305308	-1.491012
H	-3.011742	-5.420627	-1.673133
C	-5.615947	1.937949	0.165452
H	-4.225363	2.741440	1.636177
H	-4.844601	1.137955	2.022026
H	-6.719847	0.734555	-1.275329
H	-6.359747	-0.093463	0.238720
C	1.244207	3.329886	-0.661491
C	-0.025637	3.663993	-2.831801
H	-0.816570	2.875845	-0.989751
C	2.950459	0.603900	-2.364629
C	1.355693	-0.622165	-3.913017
H	1.612244	1.512925	-3.809824
H	-3.711992	-6.519723	0.446531
H	-4.523994	-5.045047	0.969029
H	-6.512742	2.358254	0.638106
H	-5.297506	2.657737	-0.603805
C	0.983468	4.798026	-0.288022
H	2.202920	3.272178	-1.195281
H	1.328289	2.714605	0.239101
C	-0.273081	5.137678	-2.462418
H	0.897098	3.596657	-3.425408
H	-0.839183	3.298831	-3.471188
C	4.112938	0.289924	-3.318222
H	2.899936	-0.166619	-1.585395
H	3.146669	1.544605	-1.840240

C	2.529891	-0.932745	-4.855580
H	1.219954	-1.463299	-3.219900
H	0.431078	-0.546337	-4.497436
C	0.827160	5.678637	-1.536805
H	1.797274	5.173012	0.345991
H	0.064054	4.855664	0.313279
H	-0.342597	5.746683	-3.373185
H	-1.245371	5.219127	-1.953706
C	3.859568	-1.009337	-4.093302
H	5.047157	0.220431	-2.747408
H	4.236889	1.118413	-4.032028
H	2.335662	-1.871684	-5.390210
H	2.596774	-0.143279	-5.619033
H	0.608256	6.716060	-1.253004
H	1.780937	5.696168	-2.085309
H	4.685759	-1.213656	-4.785985
H	3.824548	-1.849950	-3.384185

TS_{B-C}

Imaginary Freq = -80.0303 (cm^{-1})
Electronic Energy BS1 = -2658.93309589 Hartree
Electronic Energy BS2 = -2659.52150423 Hartree
Zero-point Energy Correction = 1.031135 Hartree
Thermal Correction to Enthalpy = 1.082316 Hartree
Thermal Correction to Free Energy = 0.944379 Hartree

Chemical symbol X, Y, Z

Cu	-0.081931	-0.211493	-0.375972
C	1.020212	-1.441636	-1.794124
C	1.515875	-1.328994	-0.497535
H	1.579771	-1.004424	-2.619168
C	2.665898	-1.353019	0.203130
H	2.673084	-0.891349	1.186960
H	0.298594	-2.211778	-2.059809
C	3.927373	-1.975170	-0.212876
C	5.033064	-1.904212	0.659282
C	4.104772	-2.644901	-1.441286
C	6.261837	-2.466855	0.320081
H	4.917429	-1.395071	1.614113
C	5.331519	-3.212362	-1.775664
H	3.266271	-2.720384	-2.125693
C	6.420193	-3.125526	-0.901629
H	7.097470	-2.392598	1.012041
H	5.441050	-3.725975	-2.728104
H	7.377251	-3.565249	-1.169663
B	-0.022181	-1.279345	1.330834
O	-0.681043	-2.493628	1.482838
O	0.596339	-0.905093	2.518202
C	-0.224072	-3.102752	2.722485

C	0.234420	-1.853743	3.557035
C	0.933907	-4.038548	2.351176
C	-1.376948	-3.897776	3.328590
C	-0.898992	-1.213853	4.369238
C	1.456271	-2.079706	4.443745
H	1.322434	-4.571983	3.225590
H	0.571591	-4.775131	1.627124
H	1.749867	-3.482608	1.880028
H	-1.636230	-4.730172	2.666251
H	-1.095403	-4.312281	4.303923
H	-2.268087	-3.278392	3.455472
H	-1.181858	-1.831078	5.228999
H	-0.562474	-0.238651	4.735843
H	-1.785075	-1.053393	3.748397
H	1.706468	-1.151055	4.967690
H	1.259009	-2.853932	5.194651
H	2.324938	-2.377620	3.852358
P	-2.184470	0.140316	-1.082416
C	-2.108093	1.746922	-2.034081
C	-2.856177	-1.109155	-2.288864
C	-3.510694	0.389569	0.225614
C	-1.279792	2.808864	-1.273146
H	-3.101910	2.137582	-2.279597
H	-1.613883	1.492520	-2.978780
C	-2.821216	-2.514447	-1.648798
C	-4.228977	-0.841147	-2.931497
H	-2.092740	-1.090957	-3.082370
C	-2.891713	0.838012	1.566411
C	-4.677531	1.328683	-0.142909
H	-3.916857	-0.622168	0.377281
H	-1.074983	3.668956	-1.921862
H	-1.861986	3.187269	-0.424602
P	0.318848	2.092304	-0.571191
C	-3.154507	-3.596224	-2.687949
H	-3.560086	-2.561644	-0.835191
H	-1.845690	-2.698707	-1.185809
C	-4.558493	-1.920380	-3.978243
H	-5.003267	-0.857123	-2.152254
H	-4.258155	0.154833	-3.391247
C	-3.941077	0.847912	2.686242
H	-2.477904	1.851173	1.454817
H	-2.046283	0.194558	1.821487
C	-5.740989	1.342485	0.969849
H	-4.289139	2.349194	-0.273370
H	-5.135763	1.044398	-1.095394
C	0.549565	3.107393	0.987138
C	1.627135	2.656343	-1.787914
C	-4.507983	-3.328103	-3.364355
H	-3.154122	-4.584708	-2.211952
H	-2.363209	-3.614061	-3.452113

H	-5.546515	-1.728829	-4.416176
H	-3.830691	-1.855881	-4.800589
C	-5.136479	1.742312	2.324230
H	-3.482473	1.180734	3.626030
H	-4.292492	-0.181146	2.855247
H	-6.556292	2.025517	0.698736
H	-6.184711	0.339185	1.051737
C	1.485537	2.402255	1.991413
C	0.948676	4.580299	0.786755
H	-0.457564	3.087943	1.433464
C	3.003074	2.111489	-1.343061
C	1.264888	2.156438	-3.204115
H	1.663816	3.755462	-1.809119
H	-4.708864	-4.085044	-4.132937
H	-5.308091	-3.420213	-2.614547
H	-5.901380	1.700622	3.109863
H	-4.797043	2.787675	2.269735
C	1.514473	3.159083	3.328461
H	2.504723	2.360963	1.583625
H	1.164166	1.367302	2.141699
C	0.982599	5.330251	2.129983
H	1.946912	4.625962	0.329233
H	0.259721	5.078777	0.091645
C	4.103757	2.389008	-2.377813
H	2.923364	1.029378	-1.177552
H	3.286466	2.550942	-0.379866
C	2.384994	2.428867	-4.220662
H	1.065960	1.077900	-3.157557
H	0.339046	2.634720	-3.547795
C	1.908694	4.631748	3.137656
H	2.205939	2.664159	4.022181
H	0.516568	3.107991	3.790446
H	1.300247	6.369193	1.972504
H	-0.036354	5.371155	2.543364
C	3.721396	1.835111	-3.756080
H	5.047682	1.948090	-2.034376
H	4.269021	3.474270	-2.457742
H	2.099843	2.021577	-5.199367
H	2.500355	3.515256	-4.352587
H	1.893082	5.162770	4.098069
H	2.943534	4.680982	2.766801
H	4.509858	2.047433	-4.489147
H	3.632523	0.740427	-3.691710

C

Electronic Energy BS1 = -2659.00418554 Hartree
Electronic Energy BS2 = -2659.59781010 Hartree
Zero-point Energy Correction = 1.035191 Hartree
Thermal Correction to Enthalpy = 1.086089 Hartree

Thermal Correction to Free Energy = 0.950920 Hartree

Chemical symbol X, Y, Z

Cu	0.244096	0.295294	-0.918151
C	-0.142639	-1.158554	-2.262766
C	-0.404720	-2.200269	-1.264185
H	0.687925	-1.325419	-2.949664
C	0.472295	-3.050088	-0.615519
H	0.025495	-3.707703	0.128555
H	-1.038987	-0.887271	-2.827244
C	1.910580	-3.220346	-0.733333
C	2.549842	-4.128415	0.148733
C	2.749179	-2.520589	-1.632962
C	3.928074	-4.307503	0.153359
H	1.936349	-4.686030	0.853660
C	4.131697	-2.708166	-1.633096
H	2.316381	-1.810214	-2.319927
C	4.737572	-3.592102	-0.738533
H	4.377266	-5.008011	0.853941
H	4.741943	-2.146246	-2.337188
H	5.815539	-3.730305	-0.739406
B	-1.897896	-2.417183	-0.869616
O	-2.969760	-1.942681	-1.607837
O	-2.318575	-3.167925	0.214130
C	-4.142457	-2.682435	-1.184631
C	-3.761752	-3.085692	0.284973
C	-4.262485	-3.889296	-2.125948
C	-5.376566	-1.795205	-1.315979
C	-4.095653	-1.996562	1.311292
C	-4.307063	-4.433524	0.749464
H	-5.161913	-4.478363	-1.917694
H	-4.312014	-3.527232	-3.157270
H	-3.387539	-4.540929	-2.038665
H	-5.528371	-1.531530	-2.367688
H	-6.269204	-2.324628	-0.963305
H	-5.273615	-0.869174	-0.749130
H	-5.174791	-1.905399	1.473866
H	-3.619581	-2.252990	2.261770
H	-3.704348	-1.028235	0.985872
H	-3.978891	-4.629884	1.775328
H	-5.403164	-4.435499	0.733577
H	-3.944615	-5.248328	0.119225
P	-1.198086	1.825669	0.103315
C	-0.079424	3.317139	0.303271
C	-2.671300	2.548385	-0.789750
C	-1.812302	1.384690	1.829496
C	1.310870	2.947038	0.862121
H	-0.535756	4.102424	0.915459
H	0.022196	3.719663	-0.712848
C	-3.655859	1.424001	-1.168985
C	-3.415748	3.706678	-0.098044

H	-2.228750	2.930788	-1.723758
C	-1.094238	0.108272	2.324799
C	-1.733329	2.505315	2.885319
H	-2.873699	1.131229	1.688461
H	2.011214	3.778753	0.717365
H	1.238103	2.785629	1.943415
P	2.008267	1.354966	0.124368
C	-4.792329	1.955430	-2.055180
H	-4.094184	1.012724	-0.247148
H	-3.133523	0.595579	-1.658264
C	-4.554335	4.240868	-0.985261
H	-3.842635	3.345056	0.848310
H	-2.726151	4.521110	0.155195
C	-1.616630	-0.344096	3.695852
H	-0.016567	0.307558	2.401715
H	-1.199646	-0.697426	1.588528
C	-2.270423	2.034076	4.247188
H	-0.686560	2.813211	3.013558
H	-2.281485	3.392835	2.550045
C	2.845850	0.600835	1.626651
C	3.354253	1.992371	-1.005259
C	-5.529769	3.122570	-1.382339
H	-5.491715	1.143647	-2.289719
H	-4.370489	2.293427	-3.013116
H	-5.086521	5.047870	-0.465294
H	-4.121295	4.683410	-1.894506
C	-1.527035	0.782263	4.734904
H	-1.058793	-1.227227	4.031385
H	-2.666416	-0.653357	3.595595
H	-2.184378	2.845110	4.981753
H	-3.342467	1.805413	4.153264
C	2.957628	-0.929082	1.504903
C	4.185259	1.241302	2.037299
H	2.125851	0.803643	2.434933
C	4.153240	0.825638	-1.615251
C	2.718902	2.843288	-2.126287
H	4.032112	2.630872	-0.419674
H	-6.311169	3.516948	-2.044118
H	-6.038649	2.752905	-0.479156
H	-1.931849	0.448594	5.698557
H	-0.469688	1.034898	4.906837
C	3.496772	-1.548047	2.801470
H	3.623197	-1.199009	0.677785
H	1.980095	-1.352566	1.252702
C	4.723469	0.604803	3.331107
H	4.923047	1.093470	1.237804
H	4.072138	2.326101	2.167244
C	5.212234	1.321617	-2.610707
H	3.452089	0.158340	-2.130230
H	4.625787	0.222318	-0.833298

C	3.773601	3.334348	-3.131525
H	1.967671	2.230625	-2.647778
H	2.186552	3.704933	-1.703655
C	4.841269	-0.921610	3.198711
H	3.598725	-2.630698	2.667508
H	2.769370	-1.389937	3.613156
H	5.695308	1.046769	3.586598
H	4.040485	0.843959	4.160166
C	4.575444	2.166213	-3.722937
H	5.746699	0.463821	-3.038694
H	5.961440	1.926028	-2.077486
H	3.285718	3.908576	-3.929434
H	4.462404	4.024344	-2.621471
H	5.207908	-1.355435	4.137753
H	5.587420	-1.160699	2.426423
H	5.344178	2.541100	-4.410342
H	3.901630	1.530414	-4.315881

D

Electronic Energy BS1 = -3366.41378363 Hartree
Electronic Energy BS2 = -3367.21500072 Hartree
Zero-point Energy Correction = 1.269634 Hartree
Thermal Correction to Enthalpy = 1.334493 Hartree
Thermal Correction to Free Energy = 1.168547 Hartree

Chemical symbol X, Y, Z

Cu	0.263083	-0.298877	-0.533239
B	2.925800	2.012188	-0.715466
O	3.486502	3.180631	-0.230788
O	3.714689	0.909369	-0.435397
C	5.010415	1.406340	-0.007469
C	4.647228	2.827072	0.557015
C	4.201132	2.794055	2.024707
C	5.717963	3.896030	0.356205
C	5.610094	0.446744	1.014125
C	5.891154	1.460101	-1.262425
H	5.789058	-0.523794	0.544056
H	4.942573	0.293793	1.862564
H	6.566450	0.833497	1.385365
H	5.477358	2.156433	-1.998802
H	5.920194	0.465274	-1.714266
H	6.915055	1.768490	-1.025074
H	3.454554	2.011532	2.186577
H	3.744095	3.755718	2.277428
H	5.043269	2.617598	2.702197
H	5.370180	4.848012	0.770288
H	5.933665	4.048838	-0.703395
H	6.645807	3.617210	0.869030
C	1.576807	1.952263	-1.487766

C	1.175411	0.644399	-2.035279
C	0.892596	3.142925	-1.536486
H	2.049619	0.021362	-2.261294
H	0.543850	0.712824	-2.928297
C	-0.426284	3.453352	-2.068435
H	1.391824	4.001367	-1.090677
C	-1.400575	2.489666	-2.424097
C	-0.794980	4.814767	-2.198940
C	-2.644309	2.877010	-2.924112
H	-1.191987	1.437564	-2.279617
C	-2.035339	5.196359	-2.696858
H	-0.074394	5.577392	-1.910321
C	-2.969914	4.226359	-3.077539
H	-3.365011	2.113106	-3.202708
H	-2.275740	6.252568	-2.794730
H	-3.934071	4.518216	-3.485404
P	-0.157343	-0.428644	1.656459
C	-1.782685	-0.884889	2.449496
C	1.049903	-1.616965	2.480729
C	0.264449	1.286765	2.283466
C	-3.014682	-0.083305	1.995078
H	-1.667430	-0.774540	3.534292
H	-1.924692	-1.955555	2.260938
C	2.489120	-1.059816	2.429154
C	0.710485	-2.137037	3.892727
H	1.028154	-2.473986	1.794972
C	-0.486497	2.399181	1.517842
C	0.186108	1.539697	3.799587
H	1.315463	1.369571	1.973506
H	-3.839992	-0.278179	2.690762
H	-2.802844	0.985049	2.076091
P	-3.580971	-0.382031	0.225499
C	3.501919	-2.121812	2.884152
H	2.568051	-0.188737	3.096244
H	2.727477	-0.719485	1.416208
C	1.737402	-3.190549	4.345163
H	0.705451	-1.308433	4.612924
H	-0.291385	-2.580232	3.917199
C	0.127275	3.769402	1.835312
H	-1.542858	2.413958	1.815668
H	-0.455127	2.214295	0.441630
C	0.758268	2.927649	4.147079
H	-0.863233	1.483348	4.124361
H	0.731732	0.771350	4.357583
C	-4.580921	1.207709	-0.026890
C	-4.929921	-1.698128	0.456300
C	3.171306	-2.646612	4.287828
H	4.515900	-1.703209	2.864458
H	3.484465	-2.947517	2.162305
H	1.494433	-3.533997	5.359115

H	1.659649	-4.068446	3.686610
C	0.083382	4.051468	3.344658
H	-0.406802	4.549185	1.278686
H	1.164620	3.783622	1.474477
H	0.657649	3.112332	5.224583
H	1.835305	2.926465	3.927153
C	-5.432956	1.148295	-1.311498
C	-5.460844	1.684214	1.148077
H	-3.794553	1.960979	-0.192616
C	-5.284852	-2.327195	-0.911422
C	-4.523900	-2.817797	1.438860
H	-5.827522	-1.208848	0.864682
H	3.885092	-3.425342	4.586017
H	3.275233	-1.827933	5.016211
H	0.560285	5.013429	3.572606
H	-0.966728	4.138271	3.662695
C	-6.069235	2.511024	-1.628464
H	-6.237572	0.412363	-1.176622
H	-4.827359	0.801466	-2.157447
C	-6.114777	3.043937	0.840576
H	-6.249562	0.942132	1.340267
H	-4.878957	1.770023	2.071679
C	-6.393517	-3.383818	-0.782845
H	-4.385573	-2.796563	-1.330001
H	-5.592335	-1.557419	-1.626351
C	-5.622539	-3.886741	1.568801
H	-3.602173	-3.299177	1.080054
H	-4.302601	-2.409411	2.431321
C	-6.932289	3.000824	-0.457505
H	-6.670351	2.439268	-2.544456
H	-5.272715	3.240377	-1.823563
H	-6.747541	3.352017	1.683326
H	-5.324895	3.803087	0.743685
C	-5.993007	-4.486833	0.205931
H	-6.612174	-3.815437	-1.767977
H	-7.320832	-2.903617	-0.435152
H	-5.294952	-4.674221	2.260429
H	-6.516045	-3.426700	2.016410
H	-7.351561	3.990872	-0.677604
H	-7.785472	2.318374	-0.324612
H	-6.801997	-5.219998	0.318503
H	-5.125455	-5.030547	-0.196972
N	0.442251	-2.616212	-1.864396
C	-0.296140	-2.320944	-3.098929
C	-0.012678	-3.892960	-1.294710
C	-1.775699	-2.208922	-2.735066
H	-0.141868	-3.114875	-3.845600
H	0.070599	-1.370253	-3.489056
C	-1.496393	-3.716987	-0.966701
H	0.560910	-4.090421	-0.388735

H	0.125799	-4.717861	-2.010612
H	-1.931634	-1.361376	-2.052905
H	-2.373486	-2.054647	-3.637368
O	-2.246725	-3.415026	-2.133694
H	-1.607736	-2.912951	-0.222622
H	-1.906670	-4.644092	-0.555982
O	2.492411	-2.740927	-0.138197
C	2.744904	-2.630561	-1.321572
O	1.820064	-2.739269	-2.310201
C	4.107970	-2.422340	-1.877089
C	4.319873	-1.953601	-3.180667
C	5.200724	-2.689252	-1.041237
C	5.620835	-1.757061	-3.642553
H	3.469296	-1.732786	-3.815292
C	6.498682	-2.502555	-1.510771
H	5.013095	-3.035886	-0.030189
C	6.709943	-2.036431	-2.812433
H	5.784793	-1.382306	-4.648773
H	7.344953	-2.714334	-0.863293
H	7.722460	-1.884616	-3.176590

$TS_{D\text{-}E}$

Imaginary Freq = -270.0482 (cm^{-1})
Electronic Energy BS1 = -3366.38408334 Hartree
Electronic Energy BS2 = -3367.18669407 Hartree
Zero-point Energy Correction = 1.267138 Hartree
Thermal Correction to Enthalpy = 1.331738 Hartree
Thermal Correction to Free Energy = 1.167204 Hartree

Chemical symbol X, Y, Z

Cu	0.327866	-0.986642	-0.435506
B	3.071763	1.871869	-0.830115
O	3.473294	3.174796	-0.609871
O	3.969511	0.958489	-0.314197
C	5.187414	1.676858	0.000592
C	4.654256	3.138748	0.233644
C	4.182415	3.379784	1.671982
C	5.600804	4.251414	-0.206663
C	5.845134	1.025025	1.212808
C	6.098216	1.549268	-1.227686
H	6.141635	0.002800	0.957799
H	5.162050	0.974059	2.062497
H	6.741682	1.579474	1.512720
H	5.640045	2.019487	-2.103503
H	6.238241	0.487349	-1.450206
H	7.077403	2.008553	-1.054914
H	3.516497	2.576904	2.001552
H	3.625266	4.320751	1.709685
H	5.024063	3.443354	2.369606

H	5.137725	5.225374	-0.018103
H	5.824280	4.185346	-1.273513
H	6.541333	4.204266	0.354246
C	1.787549	1.450925	-1.602119
C	1.594665	0.022633	-1.758040
C	1.005825	2.477041	-2.092536
H	2.517071	-0.534630	-1.597188
H	1.117327	-0.284781	-2.694829
C	-0.273222	2.436379	-2.780193
H	1.377337	3.485711	-1.919370
C	-1.094970	1.287405	-2.884295
C	-0.781093	3.633795	-3.342769
C	-2.319498	1.329511	-3.547631
H	-0.788240	0.368008	-2.406941
C	-2.005614	3.674594	-3.999851
H	-0.184867	4.540192	-3.261232
C	-2.784035	2.516396	-4.118758
H	-2.917266	0.423155	-3.613513
H	-2.357108	4.611288	-4.425915
H	-3.738200	2.543543	-4.637587
P	-0.092962	-0.242847	1.651887
C	-1.680704	-0.543073	2.596985
C	1.180809	-1.193906	2.667073
C	0.281605	1.575440	1.871081
C	-2.913213	0.265587	2.156764
H	-1.481995	-0.322272	3.651919
H	-1.877520	-1.620814	2.534522
C	2.600885	-0.650278	2.402696
C	0.938697	-1.384305	4.177612
H	1.142739	-2.184198	2.196447
C	-0.507873	2.453502	0.877520
C	0.171909	2.145242	3.297912
H	1.332500	1.627689	1.553886
H	-3.707851	0.134502	2.900622
H	-2.668254	1.328526	2.178748
P	-3.567087	-0.128115	0.434595
C	3.652245	-1.600029	2.994054
H	2.707578	0.339395	2.872609
H	2.771295	-0.524023	1.330617
C	2.008058	-2.316034	4.778144
H	0.971166	-0.416529	4.695423
H	-0.052493	-1.811538	4.367529
C	0.027011	3.891302	0.896754
H	-1.567427	2.481200	1.154480
H	-0.450277	2.031666	-0.126380
C	0.635718	3.613637	3.345030
H	-0.872652	2.084230	3.635726
H	0.765727	1.552930	4.002409
C	-4.321764	1.551210	0.001969
C	-5.081571	-1.204274	0.822258

C	3.430630	-1.811517	4.497953
H	4.659165	-1.205820	2.808912
H	3.582288	-2.559501	2.465280
H	1.842089	-2.422805	5.858045
H	1.890359	-3.317820	4.339344
C	-0.086287	4.488330	2.307865
H	-0.527809	4.501120	0.172767
H	1.074921	3.888832	0.569025
H	0.486484	4.015434	4.355742
H	1.715613	3.647229	3.150524
C	-5.149009	1.498194	-1.298406
C	-5.133094	2.276403	1.095028
H	-3.427173	2.156130	-0.213814
C	-5.554615	-1.915957	-0.466946
C	-4.814512	-2.264428	1.912972
H	-5.891485	-0.552185	1.183235
H	4.168285	-2.516560	4.902131
H	3.584741	-0.857120	5.024371
H	0.319549	5.507851	2.330904
H	-1.149992	4.567290	2.580194
C	-5.526459	2.910524	-1.771811
H	-6.071189	0.928131	-1.117543
H	-4.593526	0.975396	-2.083190
C	-5.525282	3.693648	0.637715
H	-6.045453	1.703084	1.314611
H	-4.571847	2.343286	2.033490
C	-6.805095	-2.773863	-0.216813
H	-4.746827	-2.564457	-0.830940
H	-5.755408	-1.190511	-1.262129
C	-6.054879	-3.138250	2.168349
H	-3.984407	-2.911863	1.596192
H	-4.505917	-1.796150	2.854165
C	-6.303161	3.669067	-0.685913
H	-6.120423	2.849849	-2.693264
H	-4.607557	3.456268	-2.026284
H	-6.115171	4.189780	1.419547
H	-4.609279	4.288859	0.507780
C	-6.547145	-3.814294	0.881382
H	-7.109546	-3.268994	-1.147732
H	-7.641057	-2.124731	0.084548
H	-5.827780	-3.890415	2.935240
H	-6.858089	-2.506227	2.575429
H	-6.525498	4.691916	-1.015789
H	-7.271831	3.172731	-0.523124
H	-7.453354	-4.400395	1.080643
H	-5.781776	-4.522046	0.529961
N	-0.111012	-2.666344	-1.359618
C	-0.788970	-2.643779	-2.643483
C	-0.455097	-3.873988	-0.626627
C	-2.301141	-2.579818	-2.344357

H	-0.566424	-3.549553	-3.220779
H	-0.471531	-1.765496	-3.209468
C	-1.972793	-3.793833	-0.359235
H	0.106270	-3.887717	0.307919
H	-0.229959	-4.774214	-1.213010
H	-2.537422	-1.648401	-1.813845
H	-2.865247	-2.621274	-3.281058
O	-2.700245	-3.709448	-1.574769
H	-2.177385	-2.920897	0.275172
H	-2.309468	-4.703146	0.148864
O	2.251125	-2.712386	0.116738
C	2.513097	-2.958616	-1.069687
O	1.627254	-3.204612	-1.993344
C	3.937915	-2.922921	-1.557223
C	4.282787	-3.294195	-2.862935
C	4.929762	-2.469849	-0.677370
C	5.610248	-3.215623	-3.283593
H	3.504923	-3.634590	-3.538163
C	6.255259	-2.391016	-1.100896
H	4.640387	-2.169123	0.322511
C	6.598276	-2.763547	-2.404398
H	5.874492	-3.503561	-4.297629
H	7.022113	-2.037342	-0.416049
H	7.632161	-2.700679	-2.734136

E

Electronic Energy BS1 = -3366.43948189 Hartree
Electronic Energy BS2 = -3367.23984833 Hartree
Zero-point Energy Correction = 1.270088 Hartree
Thermal Correction to Enthalpy = 1.334878 Hartree
Thermal Correction to Free Energy = 1.170724 Hartree

Chemical symbol X, Y, Z

Cu	-0.139859	0.274331	0.846744
B	3.440557	-1.519480	1.176204
O	4.412494	-2.352845	0.668767
O	3.933889	-0.307402	1.586026
C	5.384266	-0.408635	1.607964
C	5.659502	-1.606902	0.616636
C	5.851796	-1.167553	-0.838477
C	6.794847	-2.538586	1.034394
C	5.976042	0.931176	1.182834
C	5.766956	-0.719891	3.060253
H	5.683427	1.703127	1.901113
H	5.616869	1.236912	0.199620
H	7.070461	0.878313	1.158877
H	5.330892	-1.669301	3.386814
H	5.372415	0.071282	3.704576
H	6.852806	-0.768641	3.190901

H	5.020541	-0.540829	-1.170991
H	5.883285	-2.055443	-1.476631
H	6.785448	-0.612169	-0.972551
H	6.892580	-3.347765	0.303732
H	6.607172	-2.988181	2.011582
H	7.746019	-1.995831	1.073413
C	1.949120	-1.944191	1.340488
C	1.032827	-0.986912	1.971259
C	1.672736	-3.257540	1.070754
H	1.559770	-0.145595	2.424913
H	0.342671	-1.457480	2.666761
C	0.426650	-4.009347	1.156474
H	2.523444	-3.862842	0.760241
C	-0.853058	-3.416925	1.203671
C	0.500201	-5.420268	1.156030
C	-1.998686	-4.205885	1.290941
H	-0.952654	-2.342972	1.125762
C	-0.645110	-6.204975	1.241232
H	1.476843	-5.895323	1.098288
C	-1.903344	-5.598853	1.317483
H	-2.971178	-3.728312	1.339013
H	-0.559506	-7.288327	1.247738
H	-2.801335	-6.206875	1.387302
P	0.713178	0.136879	-1.386904
C	-0.679720	0.341286	-2.615913
C	1.689563	1.698312	-1.727137
C	1.694629	-1.345972	-1.982934
C	-1.942795	-0.530024	-2.491847
H	-0.254736	0.211275	-3.619100
H	-0.953546	1.395249	-2.520403
C	3.000592	1.755296	-0.924361
C	1.905192	2.175674	-3.179864
H	1.010310	2.427094	-1.283196
C	0.779157	-2.580522	-2.125960
C	2.534964	-1.163056	-3.261961
H	2.391451	-1.554232	-1.164263
H	-2.644106	-0.222473	-3.276573
H	-1.701894	-1.578293	-2.690716
P	-2.768148	-0.535145	-0.811952
C	3.552170	3.189484	-0.919987
H	3.738135	1.081668	-1.384094
H	2.838332	1.419897	0.102172
C	2.441550	3.620201	-3.159652
H	2.622416	1.535389	-3.704718
H	0.971709	2.144316	-3.752129
C	1.591716	-3.850461	-2.414409
H	0.096699	-2.418055	-2.970243
H	0.161197	-2.713074	-1.233168
C	3.325783	-2.444083	-3.588209
H	1.882040	-0.906968	-4.108670

H	3.238049	-0.334612	-3.140738
C	-3.924375	-2.013382	-1.038032
C	-3.968582	0.928344	-0.831115
C	3.740066	3.727566	-2.345674
H	4.501178	3.225708	-0.370204
H	2.845358	3.823260	-0.369211
H	2.603466	3.970023	-4.187622
H	1.679922	4.277162	-2.715081
C	2.421409	-3.679142	-3.693917
H	0.917195	-4.711760	-2.497323
H	2.262047	-4.052591	-1.569136
H	3.893515	-2.299374	-4.516337
H	4.060808	-2.612289	-2.789267
C	-4.907886	-2.123094	0.147073
C	-4.698717	-2.103996	-2.369047
H	-3.245373	-2.879045	-0.985433
C	-4.164437	1.388290	0.630984
C	-3.503705	2.110997	-1.702459
H	-4.934761	0.577387	-1.224957
H	4.084095	4.769357	-2.319134
H	4.527896	3.147652	-2.851304
H	3.024740	-4.575337	-3.886123
H	1.743822	-3.563502	-4.553249
C	-5.734700	-3.417030	0.078600
H	-5.594553	-1.265392	0.129626
H	-4.371855	-2.064902	1.102499
C	-5.540021	-3.391223	-2.443130
H	-5.362812	-1.233360	-2.467525
H	-4.012013	-2.073535	-3.222037
C	-5.115985	2.588566	0.737151
H	-3.183173	1.650797	1.045197
H	-4.550061	0.558007	1.235811
C	-4.456561	3.312952	-1.587986
H	-2.508914	2.411861	-1.362351
H	-3.425038	1.808520	-2.754385
C	-6.497803	-3.514046	-1.250029
H	-6.430672	-3.463588	0.925924
H	-5.061094	-4.282023	0.173097
H	-6.099046	-3.418653	-3.387473
H	-4.863191	-4.258588	-2.452476
C	-4.628224	3.757514	-0.128543
H	-5.203591	2.899308	1.786533
H	-6.124496	2.290514	0.410407
H	-4.074793	4.143159	-2.197961
H	-5.439394	3.043365	-2.003999
H	-7.057115	-4.456654	-1.303589
H	-7.239812	-2.703109	-1.297417
H	-5.327478	4.601485	-0.066965
H	-3.662259	4.112556	0.255749
N	-1.170464	0.487406	2.452619

C	-2.047384	-0.579685	2.886401
C	-0.491342	1.049575	3.608103
C	-3.070870	-0.018468	3.896386
H	-1.516803	-1.412493	3.389171
H	-2.580167	-0.984801	2.022438
C	-1.541236	1.595717	4.591159
H	0.173231	1.850090	3.280660
H	0.102631	0.293401	4.158820
H	-3.708446	0.723992	3.391934
H	-3.701691	-0.827633	4.280416
O	-2.425778	0.566892	5.012637
H	-2.104154	2.412330	4.109791
H	-1.053830	1.980857	5.492598
O	-0.810008	2.206318	0.111287
C	0.035138	2.990293	0.678563
O	0.992914	2.596628	1.381642
C	-0.110138	4.469229	0.409332
C	0.679574	5.379203	1.124839
C	-0.984621	4.947764	-0.575148
C	0.586132	6.747023	0.870101
H	1.362591	4.988316	1.871945
C	-1.072860	6.315670	-0.836780
H	-1.586132	4.240469	-1.133133
C	-0.290075	7.218420	-0.112265
H	1.198637	7.446531	1.433436
H	-1.753152	6.676962	-1.603981
H	-0.360511	8.284412	-0.313609

TS_{E-F}

Imaginary Freq = -191.0736 (cm^{-1})
Electronic Energy BS1 = -3366.42922944 Hartree
Electronic Energy BS2 = -3367.23164888 Hartree
Zero-point Energy Correction = 1.269273 Hartree
Thermal Correction to Enthalpy = 1.333898 Hartree
Thermal Correction to Free Energy = 1.168771 Hartree

Chemical symbol X, Y, Z

Cu	-0.220835	0.283224	-0.655865
B	-3.637514	-0.701801	-1.327403
O	-4.748974	-1.391891	-0.906040
O	-3.864837	0.637686	-1.504849
C	-5.305825	0.845825	-1.451037
C	-5.809569	-0.426343	-0.659888
C	-5.876679	-0.224640	0.856478
C	-7.122689	-1.018110	-1.166994
C	-5.585132	2.180932	-0.769799
C	-5.781571	0.884284	-2.908456
H	-5.151478	2.991681	-1.363179
H	-5.146563	2.225486	0.227101

H	-6.664750	2.351371	-0.689458
H	-5.564821	-0.059569	-3.418861
H	-5.246471	1.682085	-3.432057
H	-6.856108	1.081359	-2.977531
H	-4.926389	0.149608	1.242464
H	-6.080731	-1.187774	1.333453
H	-6.669993	0.475599	1.136484
H	-7.375851	-1.905376	-0.578202
H	-7.052095	-1.317980	-2.214561
H	-7.938342	-0.293945	-1.062187
C	-2.287321	-1.407605	-1.666606
C	-1.311639	-0.634624	-2.440934
C	-2.233921	-2.758522	-1.439081
H	-1.734324	0.275456	-2.853872
H	-0.742303	-1.217081	-3.157283
C	-1.147485	-3.699879	-1.662946
H	-3.149308	-3.208635	-1.058724
C	0.206953	-3.312120	-1.672261
C	-1.448457	-5.067502	-1.843300
C	1.214461	-4.242439	-1.918771
H	0.461776	-2.288509	-1.433178
C	-0.440975	-5.996252	-2.083582
H	-2.486846	-5.388691	-1.809832
C	0.896017	-5.585052	-2.134369
H	2.249739	-3.916985	-1.940680
H	-0.694560	-7.042350	-2.232619
H	1.683304	-6.308749	-2.326984
P	-0.775297	-0.107789	1.528141
C	0.683340	-0.408820	2.651146
C	-1.382921	1.521164	2.211406
C	-2.008400	-1.467935	1.923483
C	1.778884	-1.398820	2.218156
H	0.290475	-0.711310	3.630430
H	1.119547	0.585207	2.774321
C	-2.682857	1.991877	1.539843
C	-1.439762	1.748358	3.736118
H	-0.577659	2.155480	1.830666
C	-1.328850	-2.851437	1.837710
C	-2.785543	-1.346733	3.248422
H	-2.739156	-1.413637	1.106305
H	2.465159	-1.529347	3.063541
H	1.352343	-2.386565	2.016350
P	2.727242	-0.950891	0.663157
C	-2.902724	3.486666	1.816286
H	-3.531588	1.423000	1.946639
H	-2.641084	1.813576	0.462688
C	-1.655273	3.248359	4.015696
H	-2.259489	1.179326	4.189727
H	-0.516078	1.419446	4.224229
C	-2.349322	-3.990725	1.970074

H	-0.605761	-2.942767	2.659382
H	-0.765468	-2.949749	0.904976
C	-3.790812	-2.501748	3.411062
H	-2.083129	-1.350067	4.094173
H	-3.324398	-0.395899	3.288851
C	3.644418	-2.593556	0.435786
C	4.121649	0.193112	1.258127
C	-2.920688	3.779021	3.324108
H	-3.838497	3.826695	1.353395
H	-2.089854	4.042690	1.330673
H	-1.711041	3.421797	5.098429
H	-0.781587	3.805790	3.648711
C	-3.120370	-3.877090	3.292134
H	-1.837662	-4.958717	1.898325
H	-3.057292	-3.944020	1.131462
H	-4.309467	-2.407361	4.373720
H	-4.557724	-2.412105	2.628670
C	4.588219	-2.512650	-0.782780
C	4.390296	-3.179415	1.651763
H	2.834917	-3.295659	0.182753
C	4.552050	1.108757	0.091191
C	3.791374	1.058804	2.490997
H	4.976547	-0.446490	1.524348
H	-3.027303	4.856291	3.505246
H	-3.802390	3.295503	3.773019
H	-3.870447	-4.674148	3.371671
H	-2.421743	-4.015273	4.130869
C	5.229911	-3.871698	-1.107594
H	5.387135	-1.786565	-0.574253
H	4.046751	-2.130701	-1.658614
C	5.027090	-4.541291	1.321365
H	5.181394	-2.487033	1.971556
H	3.710086	-3.293844	2.503106
C	5.748639	1.993732	0.476125
H	3.693698	1.735129	-0.182897
H	4.812050	0.509267	-0.790514
C	4.999947	1.922332	2.892809
H	2.947434	1.713389	2.247621
H	3.499892	0.431008	3.341961
C	5.966240	-4.445100	0.111188
H	5.914745	-3.770570	-1.959371
H	4.445503	-4.579391	-1.416244
H	5.567897	-4.924416	2.196557
H	4.229088	-5.266457	1.101736
C	5.455804	2.821508	1.735286
H	6.009462	2.652973	-0.362077
H	6.626729	1.355649	0.660744
H	4.745670	2.531485	3.770278
H	5.832493	1.267629	3.193502
H	6.390348	-5.429325	-0.125270

H	6.812451	-3.788204	0.362089
H	6.340673	3.402839	2.025992
H	4.659812	3.545304	1.513213
N	0.631680	0.478835	-2.336869
C	1.586629	-0.425082	-2.940004
C	0.288034	1.550314	-3.257192
C	2.841454	0.387004	-3.324027
H	1.199703	-0.916300	-3.852363
H	1.885622	-1.184617	-2.218110
C	1.574595	2.315165	-3.617724
H	-0.413343	2.227905	-2.769699
H	-0.145439	1.159751	-4.196841
H	3.299463	0.779378	-2.406096
H	3.559221	-0.262483	-3.836982
O	2.522858	1.440210	-4.213445
H	1.989131	2.783820	-2.714045
H	1.353614	3.096062	-4.351837
O	1.389857	2.343719	0.655235
C	0.619941	2.964959	-0.121818
O	-0.495933	2.525915	-0.557310
C	1.041901	4.338528	-0.605081
C	0.207419	5.089862	-1.443151
C	2.289296	4.857947	-0.238186
C	0.617701	6.338127	-1.912394
H	-0.758256	4.675690	-1.714509
C	2.703478	6.103754	-0.708578
H	2.918098	4.262967	0.415066
C	1.868174	6.846562	-1.548282
H	-0.035266	6.915467	-2.562701
H	3.676404	6.496126	-0.422521
H	2.189971	7.817531	-1.916922

F

Electronic Energy BS1 = -3366.50997903 Hartree
Electronic Energy BS2 = -3367.30926523 Hartree
Zero-point Energy Correction = 1.273728 Hartree
Thermal Correction to Enthalpy = 1.338120 Hartree
Thermal Correction to Free Energy = 1.175720 Hartree

Chemical symbol X, Y, Z

Cu	-0.583307	-0.419550	-0.343416
B	2.219357	2.215411	-1.684847
O	3.043322	3.282953	-1.402241
O	2.913572	1.037784	-1.815862
C	4.334609	1.361144	-1.855897
C	4.375586	2.764558	-1.141686
C	4.515443	2.672801	0.380906
C	5.397386	3.745123	-1.710231
C	5.116569	0.255838	-1.154767

C	4.714361	1.421923	-3.340305
H	4.989683	-0.689940	-1.689739
H	4.774490	0.102496	-0.131501
H	6.184873	0.500033	-1.136962
H	4.150775	2.204079	-3.858993
H	4.470076	0.462255	-3.805918
H	5.783804	1.612120	-3.475748
H	3.769949	1.992102	0.797102
H	4.348288	3.664651	0.812157
H	5.510324	2.326585	0.678455
H	5.340224	4.692799	-1.165508
H	5.212173	3.951778	-2.766471
H	6.413207	3.348731	-1.601488
C	0.674715	2.410716	-1.815720
C	-0.202226	1.643686	-2.785308
C	0.185244	3.491106	-1.159997
H	0.211491	1.798352	-3.798256
H	-1.187851	2.108525	-2.797725
C	-1.203120	3.969304	-1.029521
H	0.911978	4.125525	-0.654426
C	-2.289362	3.097248	-0.820241
C	-1.447338	5.353499	-1.005902
C	-3.574361	3.598526	-0.617711
H	-2.097639	2.029030	-0.758455
C	-2.735135	5.853617	-0.818039
H	-0.613950	6.039178	-1.138444
C	-3.804693	4.976732	-0.623974
H	-4.398632	2.915283	-0.436767
H	-2.901874	6.927400	-0.811837
H	-4.807534	5.363065	-0.463924
P	0.225185	0.574605	1.707739
C	-1.245797	0.240790	2.826367
C	1.463705	-0.639286	2.422414
C	0.753589	2.345207	2.124970
C	-2.603242	0.306902	2.102585
H	-1.250038	0.915840	3.689114
H	-1.096262	-0.764306	3.222592
C	2.828607	-0.556478	1.713773
C	1.640412	-0.734405	3.952971
H	0.998290	-1.579263	2.100813
C	-0.479394	3.264627	2.261330
C	1.692181	2.571050	3.325716
H	1.296855	2.657476	1.222679
H	-3.407797	0.032301	2.794504
H	-2.798391	1.334693	1.771698
P	-2.680495	-0.740769	0.545700
C	3.727592	-1.744327	2.098613
H	3.338021	0.373330	2.003309
H	2.676747	-0.530692	0.630315
C	2.512384	-1.953849	4.303754

H	2.123407	0.165103	4.348698
H	0.670762	-0.819090	4.457378
C	-0.076913	4.744020	2.357284
H	-1.028248	2.997532	3.174990
H	-1.168619	3.119507	1.427580
C	2.102837	4.051148	3.432703
H	1.185644	2.266182	4.252091
H	2.590822	1.954308	3.237475
C	-4.296487	-0.119570	-0.224481
C	-3.111176	-2.489315	1.105408
C	3.884977	-1.863474	3.620501
H	4.708382	-1.643604	1.614936
H	3.284240	-2.668256	1.712270
H	2.629186	-2.028056	5.393126
H	2.001933	-2.868110	3.969598
C	0.883833	4.978579	3.530912
H	-0.974600	5.367040	2.456973
H	0.409656	5.049271	1.420737
H	2.763240	4.191755	4.298141
H	2.689475	4.320582	2.541817
C	-4.840984	-1.077801	-1.303909
C	-5.422325	0.229494	0.773435
H	-4.001110	0.805829	-0.736451
C	-2.712741	-3.483911	-0.010363
C	-2.428206	-2.889799	2.430115
H	-4.201222	-2.530736	1.255458
H	4.495223	-2.740430	3.871657
H	4.421911	-0.982653	4.006375
H	1.202987	6.028151	3.564403
H	0.354817	4.779120	4.474853
C	-6.054595	-0.478551	-2.031294
H	-5.146015	-2.021336	-0.832936
H	-4.060596	-1.328195	-2.025466
C	-6.662764	0.797794	0.058871
H	-5.707866	-0.675333	1.329037
H	-5.076890	0.957796	1.513808
C	-3.019497	-4.938247	0.378995
H	-1.636964	-3.377625	-0.185609
H	-3.223965	-3.242510	-0.947843
C	-2.745849	-4.345749	2.809989
H	-1.343985	-2.794487	2.305895
H	-2.750165	-2.225862	3.241767
C	-7.176204	-0.139826	-1.040972
H	-6.413667	-1.180611	-2.794271
H	-5.745826	0.434807	-2.561800
H	-7.451962	0.998819	0.794549
H	-6.403840	1.767381	-0.392762
C	-2.328564	-5.314481	1.695773
H	-2.695906	-5.606014	-0.429708
H	-4.108223	-5.070374	0.482475

H	-2.232214	-4.594942	3.747546
H	-3.825468	-4.448480	3.002781
H	-8.028830	0.313221	-1.562080
H	-7.543119	-1.069926	-0.582278
H	-2.572245	-6.347662	1.976026
H	-1.242146	-5.250473	1.557508
N	-0.421040	0.178414	-2.600496
C	-1.695796	-0.139770	-3.298084
C	0.683053	-0.578791	-3.263101
C	-1.906181	-1.642246	-3.439029
H	-1.691466	0.300061	-4.310519
H	-2.515416	0.311302	-2.729375
C	0.359585	-2.054649	-3.468873
H	1.575604	-0.473883	-2.652340
H	0.872594	-0.126376	-4.252457
H	-1.964376	-2.102028	-2.440794
H	-2.840789	-1.837172	-3.975703
O	-0.859842	-2.227662	-4.189333
H	0.303551	-2.560042	-2.502235
H	1.151655	-2.516676	-4.065280
O	0.493883	-3.475155	0.994787
C	1.113673	-2.967123	0.037120
O	0.862445	-1.844309	-0.525542
C	2.312659	-3.702767	-0.531508
C	3.151735	-3.087674	-1.470743
C	2.610091	-4.998530	-0.093505
C	4.269846	-3.758666	-1.965559
H	2.919180	-2.077427	-1.787606
C	3.724174	-5.674167	-0.592795
H	1.956751	-5.452128	0.644903
C	4.557589	-5.055446	-1.529182
H	4.918085	-3.272517	-2.691542
H	3.945910	-6.682138	-0.250719
H	5.427558	-5.580668	-1.915787

G

Electronic Energy BS1 = -2945.60634613 Hartree
Electronic Energy BS2 = -2946.28864871 Hartree
Zero-point Energy Correction = 1.148260 Hartree
Thermal Correction to Enthalpy = 1.205825 Hartree
Thermal Correction to Free Energy = 1.055635 Hartree

Chemical symbol X, Y, Z

C	0.680706	2.094160	-1.411236
C	-0.066084	3.244205	-1.207022
H	0.495601	4.110488	-0.861412
C	-1.467830	3.532430	-1.443266
C	-1.910214	4.874905	-1.300516
C	-2.460780	2.580310	-1.789551

C	-3.233304	5.245931	-1.511031
H	-1.177858	5.634463	-1.032697
C	-3.786688	2.958691	-2.005916
H	-2.192384	1.535620	-1.863980
C	-4.189484	4.289479	-1.874408
H	-3.522687	6.288449	-1.397194
H	-4.516301	2.196467	-2.274143
H	-5.222911	4.576665	-2.048911
C	0.262275	0.771986	-1.818817
H	-0.645302	0.704263	-2.420233
H	1.061910	0.185292	-2.276619
B	2.200569	2.262663	-1.124890
O	3.161954	1.341536	-1.499801
O	2.769349	3.363381	-0.505757
C	4.441581	1.784603	-0.988123
C	4.192547	3.321916	-0.766756
C	5.514952	1.437499	-2.016761
C	4.702805	1.026001	0.318848
C	4.926185	3.936883	0.421350
C	4.434781	4.156632	-2.031675
H	6.491666	1.829391	-1.710224
H	5.595601	0.349030	-2.108480
H	5.267304	1.839195	-3.001487
H	4.668865	-0.047721	0.120364
H	5.683335	1.273662	0.738988
H	3.936027	1.253263	1.064420
H	6.011689	3.851707	0.294929
H	4.671860	4.998585	0.499559
H	4.642672	3.454758	1.359825
H	4.042515	5.164886	-1.868543
H	5.500794	4.229530	-2.272261
H	3.909476	3.725561	-2.889660
Cu	-0.121463	-0.221226	0.006493
N	-0.099247	1.263033	1.525570
C	1.117283	1.609441	2.272577
C	-0.995709	2.176895	1.451237
C	0.873613	2.690300	3.319128
H	1.851341	1.969837	1.543418
H	1.511515	0.698790	2.733168
C	-0.959598	3.519547	2.128460
H	-1.887602	1.959396	0.869583
H	0.220963	2.303367	4.122550
H	1.814348	3.020420	3.769739
O	0.288931	3.833988	2.711902
H	-1.767071	3.543660	2.888052
H	-1.188076	4.293064	1.388117
P	-2.215354	-1.250993	0.205678
C	-1.823245	-2.930664	0.936238
C	-3.500428	-0.550735	1.388603
C	-3.102747	-1.596199	-1.407491

C	-0.485346	-3.510128	0.435461
H	-2.634914	-3.650522	0.776523
H	-1.762311	-2.758959	2.016420
C	-4.345196	0.546275	0.706461
C	-4.418008	-1.522250	2.159680
H	-2.861793	-0.050415	2.133856
C	-2.098596	-2.112281	-2.462576
C	-4.351299	-2.496819	-1.377215
H	-3.416393	-0.591612	-1.730241
H	-0.200206	-4.374679	1.047999
H	-0.602397	-3.879302	-0.588424
P	0.923228	-2.252510	0.392807
C	-5.183264	1.317380	1.736044
H	-5.022515	0.082513	-0.024357
H	-3.715584	1.238518	0.140499
C	-5.268135	-0.757272	3.191168
H	-5.082076	-2.045901	1.460160
H	-3.832725	-2.293759	2.672206
C	-2.745729	-2.176299	-3.853990
H	-1.771583	-3.124515	-2.184136
H	-1.205675	-1.478555	-2.471974
C	-5.001283	-2.584233	-2.770551
H	-4.066495	-3.506252	-1.046634
H	-5.084742	-2.124093	-0.655068
C	1.924152	-2.953025	-1.033081
C	1.850183	-2.689527	1.971115
C	-6.085400	0.367434	2.537231
H	-5.780291	2.085192	1.228763
H	-4.507409	1.848045	2.423698
H	-5.931190	-1.454537	3.719444
H	-4.599650	-0.323114	3.949794
C	-4.003803	-3.057757	-3.837980
H	-2.022389	-2.554353	-4.587605
H	-3.013965	-1.158161	-4.171477
H	-5.871623	-3.252250	-2.736160
H	-5.379444	-1.588774	-3.046852
C	3.066328	-2.025414	-1.489899
C	2.435931	-4.396078	-0.863842
H	1.173973	-2.948806	-1.840827
C	3.172644	-1.904034	2.070768
C	0.934834	-2.393247	3.180520
H	2.078097	-3.765755	1.964946
H	-6.647603	0.921426	3.299558
H	-6.828691	-0.076933	1.858367
H	-4.480684	-3.065677	-4.826199
H	-3.710961	-4.096225	-3.620816
C	3.682770	-2.529012	-2.803734
H	3.854903	-2.013059	-0.726378
H	2.717310	-0.995830	-1.592270
C	3.074391	-4.908771	-2.167982

H	3.187947	-4.420139	-0.061752
H	1.626044	-5.069598	-0.556769
C	3.870471	-2.084184	3.427725
H	2.969307	-0.838437	1.904440
H	3.852369	-2.214347	1.271285
C	1.656637	-2.572541	4.525248
H	0.557817	-1.363269	3.100038
H	0.055577	-3.047405	3.154113
C	4.188580	-3.972831	-2.662180
H	4.500170	-1.863468	-3.110184
H	2.925223	-2.480976	-3.600008
H	3.464377	-5.924383	-2.020910
H	2.294123	-4.979760	-2.940327
C	2.937282	-1.730927	4.592320
H	4.778253	-1.468008	3.458166
H	4.195596	-3.129896	3.533192
H	0.977436	-2.309716	5.346443
H	1.917001	-3.633027	4.658573
H	4.593431	-4.336601	-3.615171
H	5.018565	-3.991674	-1.939563
H	3.445944	-1.881256	5.552776
H	2.674304	-0.663506	4.537627

$TS_{G\text{-}H}$

Imaginary Freq = -242.9127 (cm^{-1})
Electronic Energy BS1 = -2945.59604634 Hartree
Electronic Energy BS2 = -2946.27375551 Hartree
Zero-point Energy Correction = 1.149075 Hartree
Thermal Correction to Enthalpy = 1.205420 Hartree
Thermal Correction to Free Energy = 1.058853 Hartree

Chemical symbol X, Y, Z

C	0.571743	2.050940	-1.251397
C	-0.290787	3.075266	-0.701632
H	0.276033	3.938691	-0.356553
C	-1.600528	3.471716	-1.247908
C	-1.970164	4.835646	-1.211713
C	-2.574125	2.570943	-1.736235
C	-3.220375	5.273883	-1.641705
H	-1.246810	5.560450	-0.843998
C	-3.824252	3.009212	-2.174716
H	-2.365770	1.509080	-1.741820
C	-4.161214	4.363067	-2.132259
H	-3.460185	6.333931	-1.598611
H	-4.544390	2.280010	-2.540982
H	-5.136350	4.702326	-2.471215
C	0.210393	0.856292	-1.845809
H	-0.794114	0.654369	-2.198432
H	0.981918	0.222944	-2.274176

B	2.106404	2.276650	-1.079623
O	3.066205	1.363314	-1.473363
O	2.666217	3.430230	-0.577092
C	4.357355	1.849476	-1.027814
C	4.088842	3.391890	-0.848130
C	5.397800	1.488164	-2.085165
C	4.680138	1.135181	0.290412
C	4.821587	4.045345	0.320400
C	4.311685	4.196810	-2.134756
H	6.377830	1.904431	-1.825500
H	5.494037	0.398855	-2.146493
H	5.109653	1.855894	-3.072154
H	4.671561	0.055667	0.120443
H	5.666110	1.420998	0.671422
H	3.930193	1.360199	1.053403
H	5.907353	3.969329	0.190700
H	4.554866	5.105473	0.372300
H	4.546377	3.584524	1.271620
H	3.916723	5.206915	-1.991178
H	5.374496	4.268926	-2.389011
H	3.778521	3.742895	-2.975971
Cu	-0.066197	-0.264276	0.243459
N	-0.102782	1.233234	1.533964
C	1.111956	1.663884	2.218547
C	-0.897744	2.250263	1.144178
C	0.854677	2.789154	3.218163
H	1.843303	2.035014	1.481993
H	1.561169	0.804994	2.730009
C	-1.013506	3.491804	2.013400
H	-1.854403	1.925921	0.738924
H	0.219045	2.421639	4.044585
H	1.791949	3.167053	3.641301
O	0.226739	3.890596	2.573970
H	-1.735801	3.273135	2.826424
H	-1.400625	4.340065	1.444170
P	-2.184132	-1.280301	0.186445
C	-1.760466	-2.947054	0.932627
C	-3.451921	-0.577316	1.386605
C	-3.091551	-1.657803	-1.409091
C	-0.426249	-3.532252	0.425738
H	-2.570792	-3.672953	0.798859
H	-1.683076	-2.755758	2.007739
C	-4.347295	0.484219	0.711193
C	-4.321100	-1.561367	2.197981
H	-2.797988	-0.047334	2.096183
C	-2.099705	-2.173267	-2.475987
C	-4.322646	-2.581181	-1.341240
H	-3.433275	-0.664651	-1.739199
H	-0.138152	-4.391518	1.044432
H	-0.548308	-3.909459	-0.594855

P	0.976767	-2.274159	0.368168
C	-5.181501	1.243836	1.753440
H	-5.028891	-0.007651	0.002507
H	-3.751965	1.192694	0.129736
C	-5.166600	-0.806164	3.240032
H	-4.987982	-2.115983	1.524778
H	-3.701829	-2.306690	2.708866
C	-2.771753	-2.273472	-3.853754
H	-1.747028	-3.173480	-2.186579
H	-1.215884	-1.527580	-2.518963
C	-4.998098	-2.702840	-2.719569
H	-4.012304	-3.579820	-1.001366
H	-5.046882	-2.210085	-0.609402
C	1.980650	-2.959640	-1.058962
C	1.909211	-2.661027	1.950825
C	-6.035308	0.280973	2.590493
H	-5.813931	1.986301	1.251277
H	-4.502670	1.803233	2.413845
H	-5.792379	-1.515871	3.796585
H	-4.491628	-0.338889	3.972308
C	-4.012725	-3.177113	-3.797749
H	-2.055750	-2.651242	-4.594687
H	-3.065569	-1.266254	-4.183411
H	-5.855075	-3.385717	-2.657123
H	-5.399767	-1.719278	-3.004859
C	3.098478	-2.010252	-1.532761
C	2.526462	-4.387794	-0.870111
H	1.226623	-2.987391	-1.862914
C	3.214321	-1.844682	2.031407
C	0.987550	-2.347899	3.152098
H	2.155762	-3.732968	1.967835
H	-6.594589	0.830182	3.358403
H	-6.781621	-0.196954	1.937952
H	-4.507221	-3.210687	-4.776602
H	-3.696570	-4.206066	-3.568706
C	3.726198	-2.519889	-2.838991
H	3.885655	-1.962350	-0.769665
H	2.725857	-0.990168	-1.650442
C	3.174758	-4.903732	-2.167603
H	3.279049	-4.382416	-0.068550
H	1.731541	-5.073939	-0.551947
C	3.917237	-1.986640	3.390416
H	2.982336	-0.787818	1.848956
H	3.900236	-2.154259	1.236353
C	1.712235	-2.487980	4.499950
H	0.597965	-1.325141	3.044899
H	0.118628	-3.016393	3.141618
C	4.266372	-3.948792	-2.675386
H	4.527490	-1.840191	-3.156486
H	2.967729	-2.503455	-3.635907

H	3.588582	-5.907553	-2.006616
H	2.395841	-5.004035	-2.938088
C	2.979915	-1.625998	4.549454
H	4.813670	-1.353608	3.406353
H	4.261419	-3.024275	3.515177
H	1.028321	-2.215927	5.313916
H	1.987435	-3.541671	4.657132
H	4.680146	-4.316317	-3.622942
H	5.096184	-3.936671	-1.952664
H	3.492277	-1.751929	5.511478
H	2.700347	-0.564729	4.474840

H

Electronic Energy BS1 = -2945.60785010 Hartree
Electronic Energy BS2 = -2946.28559545 Hartree
Zero-point Energy Correction = 1.150918 Hartree
Thermal Correction to Enthalpy = 1.207409 Hartree
Thermal Correction to Free Energy = 1.059551 Hartree

Chemical symbol X, Y, Z

C	0.488094	2.038357	-1.000041
C	-0.340207	2.979404	-0.138259
H	0.360971	3.648967	0.365342
C	-1.290569	3.862074	-0.928000
C	-1.057006	5.244051	-0.997548
C	-2.428858	3.360727	-1.580681
C	-1.919781	6.093123	-1.692698
H	-0.183825	5.653559	-0.495421
C	-3.294713	4.203640	-2.278060
H	-2.646320	2.298591	-1.544743
C	-3.045173	5.576478	-2.337783
H	-1.712236	7.159883	-1.728758
H	-4.169343	3.786497	-2.771937
H	-3.720596	6.234666	-2.878131
C	-0.022838	1.059909	-1.780580
H	-1.088356	0.909471	-1.923175
H	0.629111	0.421119	-2.371238
B	2.030379	2.227262	-0.954389
O	2.950082	1.307893	-1.412743
O	2.621892	3.374040	-0.477736
C	4.265296	1.774579	-1.003061
C	4.027747	3.320209	-0.833031
C	5.273930	1.382631	-2.078233
C	4.596170	1.076574	0.321108
C	4.834266	3.982742	0.279733
C	4.180831	4.100439	-2.144464
H	6.265518	1.787226	-1.845518
H	5.352630	0.291425	-2.127030
H	4.969534	1.742881	-3.063280

H	4.564755	-0.004832	0.171252
H	5.593772	1.349650	0.680381
H	3.861100	1.331567	1.090574
H	5.909550	3.881988	0.092533
H	4.592052	5.049215	0.322838
H	4.602002	3.546573	1.253540
H	3.795919	5.114192	-1.999387
H	5.228846	4.165015	-2.455496
H	3.603945	3.631278	-2.947674
Cu	-0.099143	-0.355482	0.405104
N	-0.348770	1.115007	1.633751
C	0.763703	1.620913	2.415720
C	-1.096695	2.196333	1.034947
C	0.300284	2.647937	3.451967
H	1.551565	2.105849	1.795995
H	1.250810	0.785476	2.936570
C	-1.534239	3.217456	2.098709
H	-2.002688	1.770815	0.588275
H	-0.348056	2.148619	4.194876
H	1.145043	3.113743	3.974301
O	-0.416843	3.713101	2.830756
H	-2.245544	2.725836	2.786082
H	-2.019274	4.093376	1.655351
P	-2.203826	-1.379490	0.071139
C	-1.810500	-2.984189	0.955338
C	-3.562161	-0.621805	1.123365
C	-2.987258	-1.876943	-1.560147
C	-0.470973	-3.609233	0.513500
H	-2.623251	-3.713793	0.867793
H	-1.745624	-2.703273	2.012123
C	-4.426064	0.362067	0.306906
C	-4.472825	-1.551531	1.951396
H	-2.958994	-0.028988	1.828301
C	-1.896790	-2.282516	-2.577192
C	-4.107793	-2.932835	-1.522187
H	-3.424523	-0.934618	-1.924228
H	-0.198475	-4.430078	1.188496
H	-0.573063	-4.048080	-0.484486
P	0.927937	-2.348484	0.426836
C	-5.319724	1.202288	1.231303
H	-5.063109	-0.200985	-0.390934
H	-3.798311	1.022780	-0.300090
C	-5.381176	-0.723463	2.879375
H	-5.096112	-2.162140	1.283655
H	-3.879949	-2.248501	2.553627
C	-2.494015	-2.502123	-3.975671
H	-1.421221	-3.217712	-2.249717
H	-1.106237	-1.523701	-2.606418
C	-4.702198	-3.165830	-2.922907
H	-3.701790	-3.881632	-1.142919

H	-4.899479	-2.629453	-0.828603
C	1.922651	-3.030061	-1.007030
C	1.876874	-2.705408	2.003587
C	-6.214289	0.302251	2.096371
H	-5.929920	1.893563	0.636500
H	-4.680229	1.820312	1.877698
H	-6.037300	-1.390932	3.452968
H	-4.750951	-0.194928	3.609281
C	-3.618047	-3.548565	-3.941214
H	-1.706926	-2.807944	-4.676791
H	-2.895327	-1.548155	-4.348319
H	-5.476019	-3.942856	-2.877218
H	-5.201257	-2.243803	-3.255974
C	2.968809	-2.033729	-1.541179
C	2.551249	-4.417820	-0.782257
H	1.151337	-3.129029	-1.788142
C	3.143400	-1.830578	2.074284
C	0.949418	-2.423006	3.208225
H	2.166668	-3.766356	2.021184
H	-6.816128	0.908403	2.785059
H	-6.922478	-0.231756	1.444698
H	-4.058343	-3.670612	-4.938746
H	-3.191574	-4.524307	-3.663285
C	3.605718	-2.555576	-2.838085
H	3.761960	-1.900500	-0.794430
H	2.526467	-1.045422	-1.691272
C	3.203196	-4.941393	-2.074471
H	3.317819	-4.343466	0.002254
H	1.800452	-5.133425	-0.423175
C	3.864966	-1.947442	3.425619
H	2.854456	-0.785329	1.903176
H	3.833967	-2.102178	1.268335
C	1.688449	-2.538565	4.550652
H	0.527818	-1.413006	3.102871
H	0.101960	-3.119197	3.203214
C	4.230521	-3.944105	-2.632720
H	4.360810	-1.843476	-3.195315
H	2.835114	-2.614372	-3.621515
H	3.675650	-5.914594	-1.889062
H	2.418140	-5.110296	-2.826573
C	2.922540	-1.628339	4.593006
H	4.733843	-1.276863	3.435951
H	4.253688	-2.970118	3.543907
H	1.000523	-2.292670	5.369399
H	2.004100	-3.581545	4.704490
H	4.651818	-4.320522	-3.573435
H	5.068082	-3.858238	-1.924127
H	3.446944	-1.737494	5.550660
H	2.600680	-0.579158	4.523175

W

Electronic Energy BS1 = -3179.33156318 Hartree
Electronic Energy BS2 = -3180.08034423 Hartree
Zero-point Energy Correction = 1.290853 Hartree
Thermal Correction to Enthalpy = 1.354780 Hartree
Thermal Correction to Free Energy = 1.192878 Hartree

Chemical symbol X, Y, Z

C	1.155342	2.041317	-0.877734
C	0.285215	3.030317	-0.111082
H	0.963450	3.606449	0.522449
C	-0.428427	4.029480	-1.006417
C	-0.019921	5.371299	-1.013421
C	-1.506295	3.670783	-1.831934
C	-0.657234	6.319489	-1.816089
H	0.809747	5.670833	-0.377479
C	-2.146776	4.612708	-2.637445
H	-1.853974	2.643453	-1.844572
C	-1.725003	5.944170	-2.633558
H	-0.319491	7.352853	-1.800590
H	-2.980402	4.305912	-3.264825
H	-2.224931	6.680269	-3.257639
C	0.716474	1.229692	-1.866134
H	-0.295732	1.262375	-2.254346
H	1.404871	0.566123	-2.382950
B	2.674131	2.036922	-0.536895
O	3.579251	1.105647	-0.996291
O	3.265972	3.021375	0.218328
C	4.838657	1.349029	-0.309536
C	4.703961	2.862985	0.099358
C	5.980992	1.033120	-1.270046
C	4.878814	0.403279	0.896167
C	5.342185	3.232949	1.434692
C	5.160968	3.828498	-1.000737
H	6.947656	1.278560	-0.815850
H	5.976083	-0.035952	-1.506811
H	5.882049	1.585963	-2.206572
H	4.801302	-0.626645	0.540435
H	5.812824	0.507761	1.457597
H	4.037253	0.589833	1.570100
H	6.421647	3.044297	1.414350
H	5.183255	4.297167	1.634466
H	4.899736	2.667400	2.257480
H	4.846207	4.841793	-0.734143
H	6.249574	3.819951	-1.118572
H	4.701296	3.572950	-1.960641
Cu	0.013385	-0.261648	0.086065
N	-0.233965	1.096119	1.513236
C	0.862104	1.373142	2.436464

C	-0.722460	2.326022	0.903688
C	0.499027	2.462792	3.452327
H	1.787198	1.693524	1.920622
H	1.106553	0.449514	2.979088
C	-1.087628	3.367462	1.973304
H	-1.632190	2.071068	0.347370
H	-0.280038	2.090233	4.138948
H	1.369990	2.761078	4.047772
O	0.030803	3.647035	2.812341
H	-1.929235	2.990207	2.575771
H	-1.378315	4.324095	1.527096
P	-2.132279	-0.926610	-0.773512
C	-2.075589	-2.685107	-0.140391
C	-3.697997	-0.238750	0.013247
C	-2.490119	-1.034358	-2.618402
C	-0.761474	-3.413638	-0.482158
H	-2.933883	-3.273107	-0.484766
H	-2.161225	-2.560739	0.944853
C	-4.242876	0.981960	-0.754324
C	-4.832668	-1.235124	0.333604
H	-3.308189	0.109099	0.976651
C	-1.245663	-1.481653	-3.416778
C	-3.715629	-1.855837	-3.063363
H	-2.676924	0.017365	-2.888325
H	-0.714772	-4.373183	0.045708
H	-0.728690	-3.649820	-1.549786
P	0.770263	-2.387995	-0.098172
C	-5.367901	1.670666	0.034145
H	-4.637851	0.669637	-1.731748
H	-3.442117	1.702214	-0.952134
C	-5.976802	-0.542708	1.095590
H	-5.233779	-1.674626	-0.588656
H	-4.449147	-2.062191	0.938942
C	-1.469553	-1.330263	-4.928959
H	-1.040104	-2.539106	-3.205766
H	-0.361531	-0.921946	-3.097021
C	-3.945354	-1.739389	-4.581527
H	-3.560233	-2.911451	-2.797226
H	-4.615009	-1.527566	-2.534738
C	1.891804	-2.929596	-1.501950
C	1.437203	-3.169736	1.470719
C	-6.505081	0.687428	0.344661
H	-5.746331	2.532873	-0.529609
H	-4.955712	2.063243	0.975064
H	-6.788342	-1.259555	1.277098
H	-5.609218	-0.229680	2.082446
C	-2.696986	-2.133856	-5.383652
H	-0.574766	-1.655119	-5.475522
H	-1.617286	-0.266756	-5.167919
H	-4.802059	-2.357973	-4.878436

H	-4.212071	-0.698845	-4.819351
C	3.214710	-2.145520	-1.567463
C	2.162785	-4.443126	-1.605923
H	1.300201	-2.633076	-2.382335
C	2.615815	-2.329113	2.003724
C	0.318484	-3.262414	2.530798
H	1.792096	-4.185393	1.241863
H	-7.291657	1.182153	0.928468
H	-6.968598	0.364389	-0.599976
H	-2.873305	-1.992500	-6.457377
H	-2.497044	-3.205813	-5.235259
C	3.970674	-2.459897	-2.867726
H	3.850049	-2.437349	-0.719999
H	3.040472	-1.071170	-1.472162
C	2.927987	-4.772172	-2.900983
H	2.764036	-4.763266	-0.743308
H	1.228832	-5.016986	-1.569725
C	3.131080	-2.833327	3.360012
H	2.281102	-1.287740	2.101431
H	3.436961	-2.322932	1.280963
C	0.852823	-3.774995	3.877340
H	-0.145535	-2.277124	2.662766
H	-0.480766	-3.929500	2.186311
C	4.232177	-3.966918	-3.009284
H	4.915668	-1.901891	-2.895020
H	3.375679	-2.108907	-3.724302
H	3.137998	-5.848416	-2.949248
H	2.285390	-4.537737	-3.762804
C	2.001736	-2.900513	4.395472
H	3.939980	-2.179438	3.711153
H	3.567574	-3.835723	3.234630
H	0.034402	-3.809489	4.607789
H	1.211638	-4.809054	3.760759
H	4.734544	-4.184280	-3.960412
H	4.917726	-4.287104	-2.210263
H	2.378451	-3.285618	5.351572
H	1.624868	-1.885338	4.586041
O	-1.927468	-0.733064	2.623819
C	-2.456085	-0.636145	3.941589
C	-3.392989	-1.840575	4.095774
H	-2.837210	-2.769222	3.922577
H	-3.833897	-1.878886	5.098577
H	-4.203182	-1.786145	3.360794
C	-1.325904	-0.716238	4.983267
H	-1.714745	-0.623514	6.004769
H	-0.800824	-1.672155	4.901095
H	-0.598137	0.085056	4.821816
C	-3.242236	0.674303	4.115553
H	-2.575404	1.537442	4.031223
H	-4.008130	0.762088	3.337719

H	-3.734527	0.719246	5.094578
H	-1.349344	0.061516	2.345721

TS_{W-X}

Imaginary Freq = -1185.6345 (cm^{-1})
Electronic Energy BS1 = -3179.32778667 Hartree
Electronic Energy BS2 = -3180.07566598 Hartree
Zero-point Energy Correction = 1.286928 Hartree
Thermal Correction to Enthalpy = 1.350386 Hartree
Thermal Correction to Free Energy = 1.189992 Hartree

Chemical symbol X, Y, Z

C	1.068992	1.931521	-0.939628
C	0.207722	2.975234	-0.222850
H	0.892984	3.553682	0.400159
C	-0.438038	3.964944	-1.180272
C	0.074480	5.266797	-1.275007
C	-1.536724	3.631731	-1.987153
C	-0.485842	6.203470	-2.145585
H	0.923722	5.543469	-0.654763
C	-2.101274	4.563020	-2.859317
H	-1.960214	2.634596	-1.934630
C	-1.577865	5.855176	-2.942696
H	-0.070132	7.206639	-2.198299
H	-2.954731	4.279224	-3.470362
H	-2.018830	6.582913	-3.618766
C	0.631957	1.121172	-1.934226
H	-0.369756	1.176347	-2.347661
H	1.322673	0.450119	-2.438479
B	2.591923	1.954992	-0.609419
O	3.520203	1.055801	-1.080856
O	3.159932	2.965624	0.129695
C	4.793390	1.369216	-0.445212
C	4.599448	2.878686	-0.043789
C	5.909433	1.100239	-1.449652
C	4.932020	0.443347	0.767388
C	5.268966	3.295723	1.261840
C	4.962382	3.855164	-1.168977
H	6.880822	1.394217	-1.036303
H	5.945151	0.030326	-1.679854
H	5.746975	1.640666	-2.384550
H	4.902608	-0.594216	0.428228
H	5.878834	0.609666	1.290925
H	4.107708	0.591289	1.471165
H	6.355130	3.163767	1.199989
H	5.062252	4.352349	1.457982
H	4.890508	2.716999	2.107055
H	4.607412	4.853911	-0.898032
H	6.044461	3.900272	-1.329800

H	4.479251	3.567633	-2.108099
Cu	0.015605	-0.232873	0.031085
N	-0.314512	1.159040	1.478701
C	0.751878	1.502883	2.426933
C	-0.823602	2.354073	0.790813
C	0.350964	2.657308	3.352592
H	1.684845	1.776089	1.910737
H	0.961809	0.613793	3.034035
C	-1.216481	3.446938	1.795191
H	-1.719914	2.043379	0.244068
H	-0.443061	2.326825	4.040749
H	1.204428	2.999373	3.948797
O	-0.106997	3.792201	2.621297
H	-2.055948	3.093118	2.412388
H	-1.518470	4.368212	1.287262
P	-2.109282	-1.020153	-0.732026
C	-2.024276	-2.713590	0.047797
C	-3.706090	-0.318719	-0.037573
C	-2.398589	-1.267346	-2.574785
C	-0.693630	-3.445176	-0.208418
H	-2.865033	-3.347479	-0.255698
H	-2.130379	-2.484948	1.115151
C	-4.220663	0.871077	-0.869648
C	-4.853894	-1.306295	0.265138
H	-3.344022	0.049932	0.931103
C	-1.124998	-1.770444	-3.288473
C	-3.606909	-2.121464	-3.004538
H	-2.574893	-0.238651	-2.928779
H	-0.634372	-4.345117	0.414221
H	-0.646104	-3.787769	-1.246265
P	0.819998	-2.364235	0.081628
C	-5.376182	1.587196	-0.153141
H	-4.574777	0.522701	-1.851198
H	-3.412701	1.584567	-1.062563
C	-6.025795	-0.589070	0.959814
H	-5.221607	-1.772110	-0.657863
H	-4.497223	-2.116175	0.908608
C	-1.287108	-1.735338	-4.815292
H	-0.929781	-2.808540	-2.989774
H	-0.254540	-1.186371	-2.974826
C	-3.773488	-2.126066	-4.535482
H	-3.468213	-3.152092	-2.646538
H	-4.525956	-1.749683	-2.543728
C	1.960692	-3.045973	-1.245185
C	1.477329	-2.958669	1.731971
C	-6.524484	0.613618	0.146679
H	-5.732097	2.428007	-0.762389
H	-5.001901	2.012683	0.789102
H	-6.844111	-1.300229	1.133059
H	-5.696666	-0.242625	1.948489

C	-2.494264	-2.577195	-5.254549
H	-0.370559	-2.097199	-5.299085
H	-1.426978	-0.693961	-5.141081
H	-4.617494	-2.768718	-4.817103
H	-4.030749	-1.108278	-4.864844
C	3.279465	-2.264988	-1.384220
C	2.243005	-4.559782	-1.177481
H	1.377364	-2.854238	-2.159250
C	2.619485	-2.028641	2.190360
C	0.341794	-2.981714	2.778285
H	1.869943	-3.979289	1.613773
H	-7.332694	1.127638	0.682285
H	-6.951259	0.258231	-0.803763
H	-2.628050	-2.520662	-6.342152
H	-2.298922	-3.633396	-5.015127
C	4.054710	-2.717761	-2.631407
H	3.904457	-2.455188	-0.500931
H	3.095952	-1.187952	-1.413603
C	3.024528	-5.025585	-2.419405
H	2.836656	-4.777457	-0.278504
H	1.312754	-5.133305	-1.087301
C	3.125909	-2.369046	3.599915
H	2.249420	-0.995175	2.172578
H	3.452613	-2.070290	1.482476
C	0.873857	-3.339057	4.174871
H	-0.165870	-2.007536	2.800648
H	-0.423793	-3.712908	2.492491
C	4.326004	-4.229354	-2.601268
H	4.997643	-2.160402	-2.708548
H	3.469736	-2.468232	-3.529436
H	3.240490	-6.099154	-2.345554
H	2.391251	-4.892277	-3.309242
C	1.979586	-2.373190	4.618945
H	3.903856	-1.651910	3.893374
H	3.601257	-3.361456	3.586008
H	0.046466	-3.335154	4.895047
H	1.275009	-4.364315	4.164213
H	4.841482	-4.547364	-3.516341
H	5.003075	-4.454260	-1.763496
H	2.352222	-2.644453	5.614907
H	1.560497	-1.359995	4.699748
O	-1.899264	-0.451125	2.553661
C	-2.525182	-0.381472	3.807932
C	-3.417892	-1.630187	3.945740
H	-2.807023	-2.532935	3.826011
H	-3.920599	-1.676221	4.920501
H	-4.183315	-1.634056	3.162540
C	-1.489890	-0.386774	4.954698
H	-1.967969	-0.310149	5.940080
H	-0.904713	-1.310261	4.931261

H	-0.795627	0.452753	4.849511
C	-3.399283	0.885954	3.927062
H	-2.776227	1.785929	3.905658
H	-4.097942	0.941502	3.084868
H	-3.979245	0.901721	4.858974
H	-1.204885	0.450964	2.132310

X

Electronic Energy BS1 = -3179.33202292 Hartree
Electronic Energy BS2 = -3180.08112023 Hartree
Zero-point Energy Correction = 1.291513 Hartree
Thermal Correction to Enthalpy = 1.355199 Hartree
Thermal Correction to Free Energy = 1.193608 Hartree

Chemical symbol X, Y, Z

C	1.172299	1.892921	-0.853152
C	0.373814	2.978375	-0.116493
H	1.068075	3.447804	0.583080
C	-0.079208	4.080692	-1.063712
C	0.693356	5.246532	-1.166703
C	-1.221147	3.971534	-1.869998
C	0.340548	6.270305	-2.046971
H	1.582133	5.343579	-0.547648
C	-1.580055	4.993644	-2.750232
H	-1.842637	3.084122	-1.812971
C	-0.800078	6.147988	-2.843270
H	0.954420	7.165442	-2.107307
H	-2.473510	4.887998	-3.360581
H	-1.080830	6.945307	-3.526225
C	0.682567	1.175741	-1.895714
H	-0.304243	1.346869	-2.316113
H	1.323364	0.483283	-2.435568
B	2.687075	1.805483	-0.498144
O	3.580904	0.895224	-1.008631
O	3.284456	2.743456	0.311873
C	4.863364	1.127450	-0.352241
C	4.721650	2.617850	0.132886
C	5.971761	0.875341	-1.369124
C	4.967576	0.134542	0.809126
C	5.403760	2.940024	1.458442
C	5.117254	3.642682	-0.937050
H	6.951709	1.110475	-0.938765
H	5.969575	-0.180606	-1.658741
H	5.831715	1.472745	-2.272446
H	4.907237	-0.882673	0.417688
H	5.917830	0.243640	1.341231
H	4.147427	0.272200	1.519546
H	6.485049	2.777145	1.387258
H	5.231362	3.990369	1.712838

H	5.007857	2.327786	2.271439
H	4.798067	4.636758	-0.609963
H	6.200087	3.658924	-1.097534
H	4.624282	3.425484	-1.889857
Cu	-0.003508	-0.230414	-0.047490
N	-0.345328	1.164137	1.448982
C	0.647530	1.419647	2.510789
C	-0.769180	2.410655	0.773426
C	0.206932	2.553440	3.441171
H	1.621120	1.659818	2.067734
H	0.750345	0.494738	3.085980
C	-1.194718	3.470208	1.798494
H	-1.631516	2.142761	0.157821
H	-0.657681	2.226108	4.038272
H	1.015319	2.834623	4.124496
O	-0.135990	3.732257	2.715491
H	-2.089398	3.120463	2.335738
H	-1.424880	4.421745	1.310044
P	-2.127282	-0.925453	-0.836711
C	-2.160489	-2.595640	-0.017267
C	-3.703670	-0.123992	-0.224431
C	-2.326266	-1.174420	-2.690334
C	-0.864701	-3.402884	-0.214288
H	-3.024380	-3.191059	-0.333351
H	-2.289605	-2.316827	1.038341
C	-4.080004	1.106356	-1.069030
C	-4.934342	-1.032151	-0.013870
H	-3.370779	0.188515	0.777568
C	-1.034099	-1.724150	-3.331862
C	-3.538543	-1.991589	-3.176818
H	-2.449554	-0.143216	-3.059122
H	-0.875853	-4.293085	0.424269
H	-0.797647	-3.765711	-1.244523
P	0.693763	-2.401364	0.114258
C	-5.219135	1.899280	-0.410396
H	-4.402678	0.791343	-2.072975
H	-3.208982	1.755927	-1.213371
C	-6.095649	-0.241992	0.616304
H	-5.274040	-1.459598	-0.965700
H	-4.675606	-1.870221	0.639443
C	-1.114173	-1.703510	-4.865299
H	-0.885804	-2.763530	-3.011690
H	-0.164464	-1.161394	-2.978409
C	-3.622304	-2.011162	-4.714431
H	-3.456726	-3.021265	-2.799277
H	-4.467130	-1.581120	-2.771599
C	1.845331	-3.165644	-1.156046
C	1.260803	-2.985431	1.800267
C	-6.451774	1.007805	-0.201096
H	-5.474916	2.773077	-1.023836

H	-4.873485	2.279778	0.561405
H	-6.973022	-0.894106	0.718170
H	-5.810149	0.060731	1.631467
C	-2.322397	-2.513228	-5.358454
H	-0.185072	-2.099411	-5.295679
H	-1.204323	-0.662582	-5.209751
H	-4.471061	-2.629512	-5.033521
H	-3.827440	-0.990057	-5.069215
C	3.208594	-2.458853	-1.250188
C	2.041361	-4.691090	-1.053765
H	1.311178	-2.958774	-2.096071
C	2.408239	-2.079008	2.293259
C	0.081268	-2.974345	2.797971
H	1.634748	-4.015889	1.709737
H	-7.252007	1.572069	0.294400
H	-6.842644	0.701746	-1.183722
H	-2.396140	-2.467729	-6.452284
H	-2.174229	-3.571581	-5.095856
C	4.012690	-2.974859	-2.453765
H	3.781928	-2.666013	-0.336296
H	3.082811	-1.374017	-1.306334
C	2.849291	-5.219262	-2.253060
H	2.582445	-4.925938	-0.126446
H	1.077814	-5.211168	-0.996612
C	2.843943	-2.406878	3.729486
H	2.070473	-1.036020	2.241733
H	3.269787	-2.156035	1.622797
C	0.553700	-3.332885	4.215248
H	-0.419197	-1.994180	2.793457
H	-0.682752	-3.696607	2.487367
C	4.199286	-4.498136	-2.387940
H	4.987427	-2.470774	-2.496708
H	3.482602	-2.710328	-3.381025
H	3.002477	-6.301513	-2.154290
H	2.263882	-5.066593	-3.172065
C	1.653813	-2.379596	4.697089
H	3.621202	-1.699168	4.047330
H	3.302135	-3.407158	3.750130
H	-0.299990	-3.314010	4.902613
H	0.941860	-4.363437	4.224299
H	4.736749	-4.859475	-3.273763
H	4.825149	-4.744262	-1.517001
H	1.979448	-2.644697	5.711020
H	1.244893	-1.360333	4.749934
O	-2.150184	-0.443648	2.474460
C	-2.863902	-0.404056	3.660838
C	-3.839055	-1.605491	3.721387
H	-3.270753	-2.537901	3.614042
H	-4.407982	-1.651366	4.660570
H	-4.552341	-1.550072	2.891529

C	-1.924411	-0.489337	4.892215
H	-2.465132	-0.415880	5.846139
H	-1.379386	-1.437796	4.883124
H	-1.185281	0.319453	4.857830
C	-3.682975	0.907378	3.777922
H	-3.007928	1.770396	3.821001
H	-4.320442	1.029062	2.894849
H	-4.321866	0.936569	4.671527
H	-1.178296	0.660602	1.925373

TS_{C-T}

Imaginary Freq = -15.1748 (cm^{-1})
Electronic Energy BS1 = -2658.96089566 Hartree
Electronic Energy BS2 = -2659.55826111 Hartree
Zero-point Energy Correction = 1.034297 Hartree
Thermal Correction to Enthalpy = 1.084998 Hartree
Thermal Correction to Free Energy = 0.949137 Hartree

Chemical symbol X, Y, Z

Cu	2.237784	0.239406	-0.922056
B	4.666867	-1.156827	-0.878928
O	4.172378	-2.431722	-1.066632
O	5.606642	-1.100523	0.126149
C	5.525829	-2.353689	0.854182
C	4.952487	-3.331022	-0.235988
C	6.038079	-3.936512	-1.133318
C	4.028927	-4.422681	0.297031
C	6.911845	-2.709392	1.381132
C	4.552726	-2.115385	2.017447
H	7.227542	-1.965655	2.119652
H	7.652570	-2.722569	0.578762
H	6.900067	-3.691942	1.866814
H	3.559565	-1.846722	1.642984
H	4.921830	-1.280208	2.620505
H	4.459839	-2.997819	2.659035
H	6.705207	-3.158288	-1.516741
H	5.558908	-4.424069	-1.987720
H	6.636937	-4.680225	-0.597152
H	3.670790	-5.039912	-0.533133
H	3.157805	-3.998996	0.801624
H	4.562482	-5.071316	1.001289
C	4.118999	0.093131	-1.651391
C	3.328750	-0.185110	-2.797451
C	4.259135	1.315611	-0.915777
H	3.314546	-1.206126	-3.163008
H	3.052927	0.570530	-3.523294
C	3.791540	2.662327	-1.190347
H	4.892993	1.238459	-0.038170
C	2.937882	3.044243	-2.261029

C	4.150925	3.694327	-0.279519
C	2.463091	4.351854	-2.382922
H	2.635627	2.313319	-2.997362
C	3.688568	4.995106	-0.416563
H	4.805661	3.443631	0.552682
C	2.828670	5.341380	-1.469602
H	1.808227	4.598613	-3.216341
H	3.993849	5.749664	0.305175
H	2.466971	6.360159	-1.578176
P	0.215538	0.396901	-0.138129
C	-0.942868	-0.171076	-1.494021
C	-2.479879	-0.039332	-1.568597
H	-0.650235	-1.217495	-1.644236
H	-2.740443	-0.307329	-2.599658
H	-2.792864	1.001868	-1.449662
H	-0.515535	0.360994	-2.354347
C	-0.115954	2.181780	0.318743
C	-1.541377	2.624455	0.700257
C	0.898490	2.614028	1.404000
H	0.168037	2.704890	-0.607558
C	-1.598482	4.141984	0.947260
H	-1.859398	2.108482	1.614276
H	-2.258306	2.355376	-0.079908
C	0.826078	4.126981	1.657854
H	0.678198	2.077614	2.339358
H	1.912788	2.335764	1.095334
C	-0.595793	4.571634	2.026621
H	-2.620002	4.429075	1.229639
H	-1.369177	4.667177	0.008688
H	1.533471	4.399378	2.451103
H	1.156551	4.650863	0.752155
H	-0.632461	5.659090	2.168301
H	-0.885085	4.117668	2.986850
C	0.143427	-0.697000	1.380903
C	-1.080273	-0.652217	2.307694
C	0.459816	-2.154044	0.969616
H	1.011566	-0.321435	1.943462
C	-0.869650	-1.541051	3.545128
H	-1.950370	-1.022329	1.758918
H	-1.295678	0.374613	2.621484
C	0.658702	-3.047016	2.204878
H	-0.375946	-2.547545	0.374869
H	1.352760	-2.184172	0.330706
C	-0.556254	-2.988894	3.142137
H	-1.764435	-1.504465	4.179878
H	-0.039229	-1.140058	4.145248
H	0.848902	-4.080074	1.886948
H	1.554339	-2.714557	2.749815
H	-0.381207	-3.604718	4.033247
H	-1.429291	-3.414481	2.626495

P	-3.495222	-1.184077	-0.468749
C	-4.212312	0.048339	0.771287
C	-5.174919	1.148289	0.287697
C	-4.795051	-0.653360	2.017479
H	-3.300573	0.558774	1.105173
C	-5.429878	2.176322	1.404692
H	-6.132464	0.695557	-0.000404
H	-4.782675	1.652717	-0.603623
C	-5.060416	0.362703	3.141086
H	-5.735660	-1.156022	1.754525
H	-4.113237	-1.436193	2.371154
C	-5.980581	1.498410	2.668517
H	-6.124357	2.950158	1.053070
H	-4.485317	2.686248	1.645617
H	-5.495072	-0.142664	4.013011
H	-4.099523	0.787004	3.469659
H	-6.118901	2.236452	3.468690
H	-6.975516	1.084168	2.446700
C	-4.882023	-1.693209	-1.650900
C	-5.978955	-2.471673	-0.893028
C	-5.528469	-0.655229	-2.593746
H	-4.358851	-2.422327	-2.292396
C	-6.958801	-3.149847	-1.864740
H	-6.539443	-1.773756	-0.255338
H	-5.528896	-3.217134	-0.225283
C	-6.495878	-1.339965	-3.576548
H	-6.085968	0.084315	-2.009567
H	-4.766927	-0.101108	-3.154352
C	-7.580839	-2.132334	-2.832318
H	-7.743344	-3.672610	-1.302688
H	-6.419287	-3.916002	-2.440665
H	-6.953819	-0.589359	-4.233585
H	-5.928494	-2.023012	-4.226014
H	-8.242799	-2.638634	-3.546142
H	-8.208994	-1.431523	-2.262079

T

Electronic Energy BS1 = -2658.99765392 Hartree
Electronic Energy BS2 = -2659.59254839 Hartree
Zero-point Energy Correction = 1.034286 Hartree
Thermal Correction to Enthalpy = 1.084619 Hartree
Thermal Correction to Free Energy = 0.951552 Hartree

Chemical symbol X, Y, Z

Cu	-0.413558	-0.132702	0.404072
B	2.589603	0.206888	2.047436
O	2.854419	-1.144113	2.172149
O	3.690860	0.907418	1.586739
C	4.841658	0.035019	1.724728

C	4.186418	-1.395062	1.664150
C	4.033234	-1.933399	0.236172
C	4.852390	-2.447939	2.547488
C	5.835935	0.339180	0.608448
C	5.469035	0.352129	3.089283
H	6.206191	1.363984	0.715611
H	5.376806	0.247679	-0.377145
H	6.693722	-0.341376	0.660376
H	4.765333	0.142395	3.900843
H	5.714698	1.417890	3.124099
H	6.384580	-0.223979	3.259967
H	3.539501	-1.200849	-0.407289
H	3.413355	-2.833715	0.262512
H	5.001749	-2.192092	-0.204990
H	4.318853	-3.399080	2.450198
H	4.830988	-2.157746	3.599949
H	5.894266	-2.606552	2.245723
C	1.227793	0.852647	2.440317
C	1.267508	2.157501	2.822713
C	0.077588	-0.068718	2.374210
H	2.201909	2.711207	2.791199
H	0.403781	2.692389	3.208177
C	-1.302675	0.237130	2.788192
H	0.367683	-1.086637	2.647800
C	-1.942976	1.485875	2.564232
C	-2.116291	-0.785991	3.342297
C	-3.295646	1.678831	2.841130
H	-1.367644	2.298136	2.138251
C	-3.462745	-0.585649	3.630095
H	-1.662247	-1.755162	3.540455
C	-4.075107	0.647764	3.374368
H	-3.743022	2.651800	2.644580
H	-4.041382	-1.397964	4.065711
H	-5.126360	0.803614	3.600332
P	-0.521841	1.651775	-1.089410
C	-1.594480	0.982314	-2.477770
C	-1.335208	3.285267	-0.664693
C	1.149791	2.035443	-1.834614
C	-1.425353	-0.534730	-2.730973
H	-1.452331	1.539392	-3.410554
H	-2.619569	1.183457	-2.146602
C	-0.365369	4.173329	0.143088
C	-2.009001	4.106281	-1.782653
H	-2.130460	2.960016	0.023380
C	1.974228	0.735369	-1.959820
C	1.182595	2.833223	-3.150471
H	1.633215	2.636134	-1.050665
H	-2.261772	-0.908864	-3.334672
H	-0.513727	-0.710522	-3.313022
P	-1.246862	-1.537240	-1.144927

C	-1.108226	5.372078	0.751917
H	0.421321	4.548265	-0.528067
H	0.139337	3.591323	0.924103
C	-2.753492	5.312597	-1.180547
H	-1.253409	4.467950	-2.492010
H	-2.711038	3.490550	-2.356649
C	3.415899	1.033729	-2.394185
H	1.507293	0.068055	-2.699175
H	1.964983	0.204012	-1.000603
C	2.629037	3.120044	-3.591613
H	0.674198	2.260181	-3.939508
H	0.636379	3.776086	-3.042727
C	-0.171663	-2.965595	-1.692098
C	-2.971665	-2.182788	-0.833946
C	-1.820115	6.192434	-0.334640
H	-0.405790	6.003334	1.310184
H	-1.847095	5.003969	1.478956
H	-3.215402	5.904326	-1.981370
H	-3.574211	4.943296	-0.547882
C	3.451234	1.828688	-3.707476
H	3.977992	0.096517	-2.498710
H	3.906867	1.611707	-1.599450
H	2.628194	3.661701	-4.546159
H	3.101784	3.783040	-2.852337
C	0.532669	-3.624628	-0.486908
C	-0.844173	-4.012121	-2.599007
H	0.614113	-2.463106	-2.279186
C	-2.995083	-3.148477	0.368792
C	-3.920350	-0.990535	-0.571543
H	-3.315380	-2.717067	-1.732629
H	-2.383777	7.019251	0.115618
H	-1.063806	6.647682	-0.991703
H	4.486772	2.060290	-3.986564
H	3.037242	1.208354	-4.516900
C	1.540664	-4.683286	-0.959078
H	-0.210743	-4.103069	0.163825
H	1.021253	-2.854652	0.121843
C	0.167625	-5.078248	-3.054601
H	-1.657018	-4.503620	-2.047039
H	-1.303124	-3.527915	-3.471042
C	-4.424341	-3.620263	0.684151
H	-2.580393	-2.629879	1.243912
H	-2.356578	-4.018082	0.176830
C	-5.348161	-1.462675	-0.253425
H	-3.532128	-0.403466	0.273648
H	-3.947322	-0.325152	-1.442591
C	0.869090	-5.735125	-1.855590
H	2.013762	-5.162824	-0.092707
H	2.345549	-4.188547	-1.523344
H	-0.337126	-5.836647	-3.666568

H	0.922634	-4.603916	-3.699202
C	-5.365971	-2.434292	0.933304
H	-4.406475	-4.284466	1.557557
H	-4.803248	-4.217809	-0.159057
H	-5.982168	-0.592123	-0.043663
H	-5.770886	-1.958367	-1.140595
H	1.606502	-6.469209	-2.203800
H	0.126112	-6.290353	-1.263869
H	-6.387282	-2.790349	1.119513
H	-5.040347	-1.897460	1.833028

U

Electronic Energy BS1 = -3366.18414038 Hartree
Electronic Energy BS2 = -3367.20857246 Hartree
Zero-point Energy Correction = 1.261793 Hartree
Thermal Correction to Enthalpy = 1.329025 Hartree
Thermal Correction to Free Energy = 1.148645 Hartree

Chemical symbol X, Y, Z

Cu	-0.419016	-0.208145	-0.078833
B	-3.047324	1.337340	2.188686
O	-4.068066	2.232496	2.461942
O	-3.460519	0.027438	2.385226
C	-4.741793	0.061100	3.062084
C	-5.288716	1.485810	2.668062
C	-6.053273	1.496887	1.335485
C	-6.113211	2.189268	3.746451
C	-5.588562	-1.116035	2.580734
C	-4.454037	-0.083439	4.564444
H	-5.110600	-2.057489	2.871724
H	-5.698691	-1.116321	1.493801
H	-6.587274	-1.090597	3.032614
H	-3.849018	0.750846	4.932982
H	-3.889722	-1.007041	4.728813
H	-5.376230	-0.132555	5.153871
H	-5.480794	0.997602	0.547116
H	-6.212283	2.535521	1.028790
H	-7.030440	1.008585	1.420257
H	-6.414681	3.179579	3.389356
H	-5.539708	2.325682	4.666144
H	-7.022110	1.622157	3.980148
C	-1.607070	1.753390	1.755531
C	-0.592319	0.691511	1.725443
C	-1.468539	3.098907	1.483769
H	-0.863891	-0.135157	2.392312
H	0.429073	1.012811	1.939038
C	-0.313869	3.887877	1.079513
H	-2.369463	3.699877	1.599679
C	0.949813	3.366956	0.704810

C	-0.454808	5.298448	1.034543
C	1.994687	4.213786	0.324444
H	1.110924	2.297895	0.694233
C	0.588713	6.136456	0.660258
H	-1.415331	5.730484	1.308686
C	1.831386	5.600254	0.301945
H	2.950457	3.777671	0.037227
H	0.435720	7.213523	0.646536
H	2.651808	6.251157	0.010319
P	-0.296745	-2.541325	-0.618592
C	0.274802	-2.493738	-2.403833
C	1.037046	-3.572728	0.214009
C	-1.850215	-3.628011	-0.601686
C	-0.493935	-1.441869	-3.230852
H	0.217501	-3.471615	-2.895165
H	1.336577	-2.222069	-2.352650
C	0.784217	-3.651068	1.735608
C	1.318561	-4.975468	-0.361025
H	1.932836	-2.951145	0.068383
C	-3.121550	-2.760811	-0.453821
C	-2.011875	-4.621413	-1.771565
H	-1.750511	-4.213952	0.324944
H	-0.056935	-1.363108	-4.234380
H	-1.530539	-1.773325	-3.369527
P	-0.559665	0.257849	-2.400146
C	1.951335	-4.338347	2.465397
H	-0.137323	-4.220251	1.929930
H	0.619901	-2.644499	2.136873
C	2.492903	-5.654156	0.368161
H	0.424505	-5.604882	-0.249262
H	1.533267	-4.919960	-1.435445
C	-4.381554	-3.628649	-0.296483
H	-3.233579	-2.127647	-1.347460
H	-3.019852	-2.077712	0.396934
C	-3.270870	-5.491440	-1.604166
H	-2.094451	-4.063096	-2.715185
H	-1.129106	-5.263355	-1.866062
C	-2.273598	0.834626	-2.942250
C	0.685690	1.219068	-3.444227
C	2.249205	-5.727724	1.882479
H	1.727957	-4.414638	3.537452
H	2.850559	-3.710506	2.375486
H	2.655569	-6.659484	-0.041932
H	3.414102	-5.084475	0.175582
C	-4.534008	-4.633284	-1.447376
H	-5.269460	-2.987162	-0.230517
H	-4.322844	-4.175679	0.656500
H	-3.371152	-6.167861	-2.463130
H	-3.152535	-6.129943	-0.716042
C	-2.774752	2.065298	-2.155577

C	-2.472079	1.029611	-4.459069
H	-2.904474	-0.014515	-2.631462
C	0.676985	2.729281	-3.129788
C	2.100380	0.630875	-3.244821
H	0.403356	1.078780	-4.499104
H	3.115334	-6.175489	2.386309
H	1.394868	-6.392813	2.078810
H	-5.410850	-5.272879	-1.284054
H	-4.716609	-4.086016	-2.384540
C	-4.244101	2.370551	-2.492681
H	-2.165465	2.943246	-2.404644
H	-2.650615	1.908232	-1.078640
C	-3.941495	1.346205	-4.793604
H	-1.841874	1.859908	-4.806525
H	-2.149444	0.138172	-5.012646
C	1.750142	3.491126	-3.926978
H	0.845049	2.882560	-2.056530
H	-0.305430	3.158336	-3.353549
C	3.164988	1.401581	-4.044343
H	2.354351	0.655517	-2.176828
H	2.119114	-0.424479	-3.545869
C	-4.454330	2.559006	-4.002705
H	-4.569946	3.264691	-1.946874
H	-4.876917	1.541890	-2.139590
H	-4.048567	1.518833	-5.872607
H	-4.561373	0.468962	-4.554572
C	3.148324	2.898718	-3.709421
H	1.733585	4.548582	-3.635561
H	1.505135	3.455130	-4.999612
H	4.154899	0.972870	-3.841566
H	2.978203	1.268890	-5.120882
H	-5.515266	2.733564	-4.223677
H	-3.914186	3.459028	-4.332456
H	3.890238	3.433666	-4.316407
H	3.439843	3.038934	-2.658498
O	2.745519	-0.518051	0.281276
C	3.835107	-0.110442	0.635684
O	4.081087	0.523331	1.810164
C	5.095008	-0.275918	-0.152774
N	2.864160	0.850690	2.570131
C	5.038375	-1.043235	-1.325005
C	6.311138	0.306084	0.235563
C	3.207104	2.075531	3.312873
C	2.613570	-0.266555	3.500077
C	6.181170	-1.225637	-2.100775
H	4.091120	-1.486572	-1.612119
C	7.452198	0.122974	-0.544256
H	6.357502	0.899923	1.141099
C	4.256690	1.859562	4.405948
H	2.262681	2.403067	3.761260

H	3.512067	2.839562	2.593146
C	3.687028	-0.412606	4.579769
H	2.494639	-1.178866	2.910047
H	1.644790	-0.037728	3.958331
C	7.390042	-0.642033	-1.711566
H	6.130911	-1.822917	-3.006969
H	8.390740	0.578484	-0.241060
H	5.238869	1.635441	3.958831
H	4.357609	2.758814	5.021551
O	3.868943	0.809567	5.285140
H	4.639996	-0.737581	4.131125
H	3.385468	-1.158129	5.322375
H	8.281529	-0.783220	-2.316735

$TS_{U\text{-}V}$

Imaginary Freq = -338.7901 (cm^{-1})
Electronic Energy BS1 = -3366.36761180 Hartree
Electronic Energy BS2 = -3367.17869019 Hartree
Zero-point Energy Correction = 1.265015 Hartree
Thermal Correction to Enthalpy = 1.330451 Hartree
Thermal Correction to Free Energy = 1.161296 Hartree

Chemical symbol X, Y, Z

Cu	-1.329553	-0.499777	-0.211666
B	-0.534691	2.837672	-0.190276
O	-1.831336	3.119512	-0.579715
O	0.005193	3.844426	0.586156
C	-0.897952	4.978156	0.522829
C	-2.265357	4.290304	0.160164
C	-3.021731	3.780102	1.393837
C	-3.194305	5.112370	-0.727757
C	-0.870234	5.696460	1.868740
C	-0.375021	5.898091	-0.587426
H	0.120891	6.131204	2.032313
H	-1.075055	5.009090	2.692642
H	-1.607788	6.506808	1.891853
H	-0.385544	5.387866	-1.555617
H	0.660396	6.170993	-0.361975
H	-0.968090	6.815138	-0.666402
H	-2.363426	3.195077	2.040967
H	-3.837166	3.130125	1.065527
H	-3.445803	4.603648	1.977550
H	-4.108662	4.544669	-0.928799
H	-2.725345	5.347508	-1.685528
H	-3.474838	6.049112	-0.232744
C	0.223835	1.530460	-0.537919
C	1.377417	1.304806	0.200258
C	-0.356283	0.737304	-1.596962
H	1.624873	1.983566	1.009611

H	1.920712	0.376275	0.198589
C	0.084511	-0.497899	-2.245383
H	-1.158362	1.240159	-2.137348
C	1.237078	-1.258264	-1.903288
C	-0.716942	-1.014671	-3.306636
C	1.527144	-2.461743	-2.544971
H	1.943876	-0.891081	-1.176702
C	-0.415656	-2.211890	-3.942078
H	-1.588390	-0.442289	-3.618722
C	0.703532	-2.963586	-3.555950
H	2.431372	-2.992814	-2.256542
H	-1.050915	-2.559178	-4.754067
H	0.938481	-3.900828	-4.052726
P	-0.493409	-1.586489	1.650364
C	-2.011570	-2.487913	2.302602
C	0.839470	-2.906996	1.542471
C	0.004654	-0.382198	2.988614
C	-3.364791	-1.850617	1.900058
H	-1.964356	-2.596857	3.391908
H	-1.952791	-3.499474	1.892768
C	2.249438	-2.275211	1.563122
C	0.769318	-4.088382	2.533282
H	0.682200	-3.308575	0.529736
C	-1.002267	0.787218	3.040836
C	0.265983	-0.936399	4.400253
H	0.945149	0.028832	2.596610
H	-4.178816	-2.563990	2.081323
H	-3.560163	-0.979443	2.536116
P	-3.435795	-1.190011	0.140150
C	3.327573	-3.317704	1.237464
H	2.448224	-1.858879	2.560743
H	2.331864	-1.452403	0.852869
C	1.853078	-5.132810	2.207163
H	0.915570	-3.725270	3.558906
H	-0.215142	-4.569591	2.508674
C	-0.506821	1.901110	3.974882
H	-1.975112	0.420584	3.401742
H	-1.159405	1.181411	2.031038
C	0.759210	0.179206	5.339790
H	-0.660174	-1.368111	4.807213
H	1.004723	-1.743416	4.369294
C	-4.876132	0.001900	0.234109
C	-3.961102	-2.658148	-0.896376
C	3.257929	-4.511958	2.197953
H	4.310725	-2.837144	1.269339
H	3.184556	-3.662975	0.203556
H	1.799693	-5.958269	2.928863
H	1.642522	-5.564549	1.217383
C	-0.220868	1.360689	5.383680
H	-1.248186	2.709442	4.022472

H	0.406046	2.338484	3.547292
H	0.917109	-0.224247	6.348035
H	1.737642	0.533692	4.984634
C	-4.823509	1.039501	-0.910172
C	-6.265514	-0.653174	0.361269
H	-4.677001	0.552277	1.167813
C	-4.317562	-2.228604	-2.335631
C	-2.817828	-3.700059	-0.931242
H	-4.845950	-3.116388	-0.429674
H	4.004750	-5.269375	1.927952
H	3.507202	-4.172860	3.214909
H	0.173538	2.157781	6.026186
H	-1.165879	1.027264	5.838929
C	-5.951982	2.074423	-0.770591
H	-4.927001	0.534303	-1.878666
H	-3.844580	1.532816	-0.917427
C	-7.371916	0.406602	0.495784
H	-6.467728	-1.262481	-0.529561
H	-6.289067	-1.335799	1.220644
C	-4.667061	-3.437019	-3.219890
H	-3.457012	-1.699751	-2.766494
H	-5.157488	-1.526534	-2.332982
C	-3.168626	-4.905208	-1.818261
H	-1.908115	-3.216026	-1.314939
H	-2.590714	-4.054452	0.079156
C	-7.330361	1.404701	-0.670761
H	-5.921621	2.770791	-1.618225
H	-5.785039	2.676491	0.134742
H	-8.352602	-0.082832	0.549700
H	-7.237910	0.950826	1.442524
C	-3.529976	-4.466557	-3.242431
H	-4.894049	-3.092484	-4.236692
H	-5.582398	-3.912644	-2.836472
H	-2.322462	-5.603265	-1.834928
H	-4.018404	-5.446021	-1.375007
H	-8.115907	2.161340	-0.552059
H	-7.543718	0.872105	-1.609538
H	-3.811881	-5.334879	-3.850997
H	-2.644116	-4.017936	-3.710667
O	4.174595	-0.615612	-0.675146
C	5.119881	0.180047	-0.741572
O	5.074818	1.435099	-1.079718
C	6.522074	-0.302309	-0.423939
N	3.204390	1.946084	-1.009618
C	6.748956	-1.678966	-0.298190
C	7.587822	0.589431	-0.248484
C	2.837970	1.720621	-2.411774
C	3.387386	3.378337	-0.720491
C	8.023147	-2.158576	0.002849
H	5.912045	-2.353244	-0.446117

C	8.861285	0.110078	0.060621
H	7.402806	1.652935	-0.355378
C	3.627820	2.563094	-3.416483
H	1.769539	1.957003	-2.512626
H	2.956539	0.651653	-2.609464
C	4.161343	4.149921	-1.794376
H	3.885544	3.455426	0.252452
H	2.383991	3.809622	-0.624337
C	9.082065	-1.264300	0.186147
H	8.192306	-3.228725	0.094815
H	9.682977	0.807961	0.201759
H	4.668499	2.211353	-3.454481
H	3.185228	2.472449	-4.413791
O	3.586174	3.945422	-3.076774
H	5.214284	3.834328	-1.789130
H	4.113783	5.226400	-1.595465
H	10.075478	-1.637040	0.423579

V

Electronic Energy BS1 = -3366.48387183 Hartree
Electronic Energy BS2 = -3367.28620677 Hartree
Zero-point Energy Correction = 1.271317 Hartree
Thermal Correction to Enthalpy = 1.336412 Hartree
Thermal Correction to Free Energy = 1.169808 Hartree

Chemical symbol X, Y, Z

Cu	-1.051433	-0.255868	-0.098431
B	-0.646943	2.786052	-0.542149
O	-1.934256	3.237446	-0.753866
O	0.043661	3.564955	0.356531
C	-0.712695	4.789341	0.533054
C	-2.172624	4.311484	0.203592
C	-2.871771	3.683018	1.414577
C	-3.070160	5.354554	-0.451923
C	-0.496835	5.291488	1.956907
C	-0.164325	5.799054	-0.482859
H	0.551460	5.577236	2.087041
H	-0.725939	4.518972	2.694129
H	-1.119779	6.170514	2.157784
H	-0.302350	5.438133	-1.506452
H	0.909537	5.919219	-0.315044
H	-0.648181	6.775956	-0.380660
H	-2.243546	2.908374	1.867298
H	-3.805552	3.222296	1.085891
H	-3.108471	4.432326	2.176428
H	-4.053728	4.919507	-0.657902
H	-2.649871	5.702837	-1.397612
H	-3.208141	6.216156	0.211023
C	-0.035971	1.490644	-1.161163

C	1.482557	1.472782	-1.047101
C	-0.843818	0.712137	-1.976349
H	1.790967	1.587769	-0.008327
H	1.958362	0.554556	-1.380477
C	-0.587625	-0.580577	-2.646894
H	-1.829648	1.121580	-2.199013
C	0.502657	-1.430038	-2.347057
C	-1.562461	-1.048034	-3.556461
C	0.594081	-2.694064	-2.931748
H	1.299031	-1.125637	-1.678664
C	-1.459954	-2.306044	-4.142234
H	-2.410120	-0.408823	-3.793982
C	-0.382302	-3.141283	-3.823927
H	1.448876	-3.317130	-2.684476
H	-2.222151	-2.639839	-4.841468
H	-0.304324	-4.126654	-4.275525
P	-0.124592	-1.301873	1.696404
C	-1.597594	-1.561599	2.845298
C	0.609959	-3.005465	1.413505
C	1.119973	-0.254759	2.563891
C	-2.893752	-0.838730	2.408043
H	-1.321345	-1.232867	3.853704
H	-1.784756	-2.634757	2.916219
C	2.051383	-2.875314	0.889392
C	0.537769	-4.027074	2.566897
H	0.004956	-3.402676	0.587908
C	0.572198	1.173323	2.760557
C	1.738922	-0.778444	3.871184
H	1.925471	-0.194776	1.812366
H	-3.757837	-1.261945	2.935483
H	-2.827964	0.217068	2.701346
P	-3.212740	-0.745588	0.558988
C	2.583845	-4.235424	0.422174
H	2.706310	-2.486003	1.677774
H	2.123004	-2.154155	0.074083
C	1.085337	-5.390385	2.102909
H	1.125169	-3.668863	3.421492
H	-0.490225	-4.161553	2.924892
C	1.707482	2.114181	3.193530
H	-0.221897	1.172082	3.526767
H	0.124490	1.546387	1.833466
C	2.882423	0.160356	4.300085
H	0.976171	-0.830795	4.664572
H	2.133180	-1.790429	3.737308
C	-4.681249	0.421838	0.529296
C	-3.842128	-2.425285	0.016415
C	2.512821	-5.277904	1.546056
H	3.611368	-4.111436	0.061892
H	1.987526	-4.582703	-0.436311
H	1.049554	-6.104387	2.935887

H	0.422206	-5.787967	1.318947
C	2.390274	1.605717	4.472146
H	1.318646	3.130345	3.336007
H	2.444924	2.157341	2.381524
H	3.336798	-0.202473	5.231191
H	3.652868	0.130385	3.518388
C	-4.797856	1.171813	-0.816294
C	-6.022511	-0.201355	0.968189
H	-4.408736	1.170616	1.286503
C	-4.397342	-2.350085	-1.423971
C	-2.698278	-3.460235	0.076451
H	-4.645987	-2.739112	0.697911
H	2.857882	-6.258280	1.193294
H	3.191880	-4.976863	2.357342
H	3.229171	2.260729	4.739581
H	1.675068	1.650441	5.308738
C	-5.948626	2.192034	-0.791335
H	-4.973491	0.457442	-1.630710
H	-3.853133	1.681556	-1.032262
C	-7.141255	0.853833	0.992481
H	-6.310343	-0.999558	0.272425
H	-5.922507	-0.667155	1.956805
C	-4.821694	-3.730865	-1.950746
H	-3.617309	-1.941390	-2.079658
H	-5.249338	-1.665522	-1.476150
C	-3.126951	-4.837323	-0.451220
H	-1.861405	-3.089455	-0.532124
H	-2.327120	-3.565436	1.099803
C	-7.277117	1.548266	-0.370194
H	-6.042683	2.664893	-1.776819
H	-5.704385	2.997023	-0.082445
H	-8.089220	0.383343	1.282073
H	-6.913662	1.606167	1.762180
C	-3.667513	-4.739174	-1.882554
H	-5.184787	-3.630531	-2.981393
H	-5.667402	-4.103084	-1.353440
H	-2.272336	-5.524168	-0.405932
H	-3.903725	-5.253866	0.207069
H	-8.073193	2.302221	-0.335986
H	-7.576986	0.806206	-1.125188
H	-3.996974	-5.723519	-2.237357
H	-2.859017	-4.408790	-2.549845
O	3.443518	-1.044747	-1.164242
C	4.106754	-0.512089	-0.224213
O	3.636352	0.053820	0.804413
C	5.623099	-0.534431	-0.364423
N	1.905809	2.646669	-1.831043
C	6.223945	-1.143824	-1.472620
C	6.434567	0.058539	0.610485
C	2.354083	2.307276	-3.188010

C	2.871629	3.535848	-1.174015
C	7.612600	-1.158920	-1.606414
H	5.577857	-1.597190	-2.217398
C	7.823518	0.046660	0.479685
H	5.950254	0.527070	1.461307
C	3.792530	1.769857	-3.192024
H	2.310187	3.218456	-3.800404
H	1.660560	1.575568	-3.620593
C	4.295125	2.973100	-1.225945
H	2.545258	3.709452	-0.144517
H	2.851186	4.498710	-1.704733
C	8.416274	-0.562718	-0.630204
H	8.070542	-1.632073	-2.472274
H	8.445883	0.512824	1.240481
H	3.842092	0.801057	-2.674312
H	4.154621	1.637719	-4.217957
O	4.671381	2.712290	-2.577336
H	4.360082	2.062688	-0.614066
H	5.019223	3.700855	-0.841974
H	9.498885	-0.571410	-0.734639

G’

Electronic Energy BS1 = -2945.59636996 Hartree
Electronic Energy BS2 = -2946.28067721 Hartree
Zero-point Energy Correction = 1.148884 Hartree
Thermal Correction to Enthalpy = 1.205365 Hartree
Thermal Correction to Free Energy = 1.060357 Hartree

Chemical symbol X, Y, Z

C	-1.359943	-1.352310	-1.542415
C	-0.739748	-2.558168	-1.789353
H	-1.298716	-3.446239	-1.486812
C	0.579377	-2.871355	-2.312061
C	0.992234	-4.230371	-2.334470
C	1.554853	-1.920965	-2.710253
C	2.282268	-4.610299	-2.684112
H	0.272881	-4.993544	-2.042736
C	2.851372	-2.305119	-3.058522
H	1.299549	-0.873520	-2.725427
C	3.235952	-3.646772	-3.042176
H	2.551297	-5.664646	-2.677863
H	3.566882	-1.539087	-3.353048
H	4.245281	-3.940977	-3.317876
C	-0.872760	-0.017812	-1.887703
H	-0.323866	0.060844	-2.833645
H	-1.673481	0.726225	-1.870569
B	-2.787620	-1.430096	-0.759699
O	-3.804355	-2.289423	-1.331475
O	-3.446734	-0.153745	-0.495137

C	-4.703932	-1.442651	-2.045677
C	-4.702676	-0.107438	-1.184390
C	-6.060095	-2.147570	-2.128585
C	-4.156767	-1.238748	-3.471443
C	-4.747228	1.182967	-2.013608
C	-5.837148	-0.048529	-0.147877
H	-6.812956	-1.508787	-2.605324
H	-5.957498	-3.059226	-2.726433
H	-6.421959	-2.437596	-1.138799
H	-4.019491	-2.222706	-3.930438
H	-4.843113	-0.653314	-4.094083
H	-3.182416	-0.744266	-3.457422
H	-5.662491	1.241378	-2.614894
H	-4.725238	2.048706	-1.341350
H	-3.884414	1.254443	-2.678316
H	-5.661494	0.794797	0.526999
H	-6.813915	0.090687	-0.624510
H	-5.888735	-0.957273	0.457320
Cu	0.399802	0.542587	-0.430851
P	2.278161	-0.139491	0.787496
P	0.431073	2.516691	0.761249
C	2.340450	1.013337	2.272300
C	2.578722	-1.806439	1.597827
C	3.825993	0.238865	-0.217759
C	1.886948	2.456473	1.952463
C	0.667166	4.168773	-0.084674
C	-1.078607	2.647268	1.857291
H	3.332490	1.026906	2.736171
H	1.658923	0.560442	3.001892
C	2.269389	-2.940701	0.601092
C	3.953124	-2.021576	2.261286
H	1.815987	-1.836531	2.394702
C	3.481605	1.149034	-1.418894
C	5.020803	0.829785	0.557689
H	4.116502	-0.740805	-0.625553
H	1.671875	3.001985	2.880094
H	2.705347	2.990069	1.453862
C	-0.044074	4.197107	-1.454977
C	0.324167	5.412821	0.755827
H	1.749913	4.188790	-0.289322
C	-2.348646	2.760381	0.984546
C	-1.150758	1.390919	2.753426
H	-0.992815	3.534086	2.502381
C	2.386916	-4.326216	1.251541
H	2.961023	-2.889813	-0.249506
H	1.273971	-2.809975	0.170460
C	4.042464	-3.406814	2.925275
H	4.734859	-1.953256	1.492909
H	4.158998	-1.236835	2.999884
C	4.696124	1.353690	-2.336672

H	3.137783	2.126516	-1.048309
H	2.648559	0.721316	-1.985510
C	6.239914	1.021645	-0.361236
H	4.734352	1.808976	0.968185
H	5.292809	0.201153	1.410523
C	0.279781	5.493671	-2.213424
H	-1.129672	4.124634	-1.312303
H	0.245477	3.315880	-2.039944
C	0.642606	6.705634	-0.015375
H	-0.746128	5.400371	1.003270
H	0.869025	5.395196	1.708920
C	-3.631286	2.705392	1.828195
H	-2.369784	1.931469	0.265669
H	-2.328145	3.692787	0.408932
C	-2.449079	1.328884	3.571398
H	-1.092260	0.504507	2.105564
H	-0.288773	1.355142	3.430365
C	3.755765	-4.526885	1.914755
H	2.204443	-5.092481	0.489660
H	1.602135	-4.445646	2.015687
H	5.034019	-3.538104	3.377245
H	3.312084	-3.461614	3.746726
C	5.899425	1.911936	-1.564697
H	4.427884	2.018836	-3.167077
H	4.969552	0.386653	-2.783409
H	7.072600	1.450594	0.210831
H	6.576725	0.037965	-0.720004
C	-0.068926	6.734257	-1.376697
H	-0.259169	5.511926	-3.168720
H	1.353038	5.511481	-2.456445
H	0.358477	7.579138	0.585233
H	1.729422	6.771354	-0.173517
C	-3.681396	1.419117	2.662674
H	-4.501916	2.763340	1.163651
H	-3.681110	3.579923	2.494920
H	-2.464612	0.403155	4.163100
H	-2.462885	2.158701	4.293410
H	3.807370	-5.506680	2.405973
H	4.534854	-4.519792	1.138297
H	6.768943	2.009423	-2.226573
H	5.660260	2.924671	-1.206138
H	0.192415	7.649667	-1.922096
H	-1.156553	6.763744	-1.213352
H	-4.601184	1.379697	3.260418
H	-3.696578	0.565272	1.972994
N	-2.500088	-2.094969	0.755961
C	-3.640681	-2.669146	1.482733
C	-1.351850	-2.073256	1.315787
C	-3.398871	-2.636111	2.983684
H	-3.783921	-3.690899	1.119198

H	-4.521690	-2.092902	1.204879
C	-1.073789	-2.586955	2.696032
H	-0.531873	-1.632182	0.753628
H	-3.409284	-1.594979	3.344189
H	-4.170080	-3.196397	3.517832
O	-2.154860	-3.259897	3.298477
H	-0.738157	-1.725479	3.305810
H	-0.233049	-3.289052	2.648299

$TS_{G'-H'}$

Imaginary Freq = -200.5302 (cm^{-1})
Electronic Energy BS1 = -2945.58475535 Hartree
Electronic Energy BS2 = -2946.26269354 Hartree
Zero-point Energy Correction = 1.148930 Hartree
Thermal Correction to Enthalpy = 1.204656 Hartree
Thermal Correction to Free Energy = 1.059460 Hartree

Chemical symbol X, Y, Z

C	-0.613527	-1.629679	-1.631407
C	0.437633	-2.583746	-1.601934
H	0.115181	-3.618473	-1.502528
C	1.839064	-2.480286	-1.977349
C	2.662235	-3.621883	-1.786355
C	2.486607	-1.309870	-2.444925
C	4.032371	-3.592091	-2.022100
H	2.198977	-4.540141	-1.429896
C	3.859429	-1.289083	-2.694253
H	1.910977	-0.411641	-2.607144
C	4.648919	-2.420417	-2.479352
H	4.624465	-4.489265	-1.855830
H	4.315648	-0.374433	-3.068703
H	5.717534	-2.397365	-2.675167
C	-0.595160	-0.332261	-2.186705
H	0.062953	-0.098525	-3.026419
H	-1.565734	0.161571	-2.240707
B	-1.987754	-2.124709	-0.845464
O	-2.804558	-3.043485	-1.621750
O	-2.865525	-1.026403	-0.451375
C	-4.054777	-2.429177	-1.913865
C	-4.217742	-1.396917	-0.736745
C	-5.133199	-3.514698	-1.965722
C	-3.950705	-1.749491	-3.290972
C	-5.012936	-0.142917	-1.110131
C	-4.838900	-2.028771	0.521408
H	-6.130989	-3.078761	-2.098646
H	-4.938040	-4.183969	-2.810493
H	-5.132467	-4.118759	-1.054935
H	-3.633701	-2.499432	-4.022754
H	-4.906957	-1.324873	-3.618308

H	-3.196586	-0.958784	-3.277472
H	-6.012412	-0.407123	-1.475976
H	-5.136938	0.502102	-0.233242
H	-4.497190	0.432424	-1.882065
H	-4.767588	-1.318841	1.350000
H	-5.895500	-2.281386	0.377427
H	-4.301589	-2.935169	0.810743
Cu	0.159706	0.645497	-0.510785
P	2.192876	1.150726	0.539285
P	-0.890314	2.310278	0.713324
C	1.779127	2.527648	1.752189
C	3.158442	-0.019210	1.642693
C	3.381871	1.911048	-0.701576
C	0.534789	3.349087	1.359082
C	-1.941461	3.630200	-0.093798
C	-1.746096	1.700563	2.269520
H	2.636186	3.192464	1.899594
H	1.609418	2.033889	2.714505
C	3.642319	-1.257564	0.867575
C	4.311759	0.572237	2.478141
H	2.375567	-0.352511	2.345264
C	2.591057	2.506486	-1.890566
C	4.390316	2.955148	-0.182316
H	3.936941	1.041131	-1.079390
H	0.217165	3.974803	2.202450
H	0.793144	4.030074	0.537899
C	-2.985544	3.016825	-1.050643
C	-2.569591	4.673705	0.851507
H	-1.205383	4.155770	-0.724557
C	-3.200816	1.265961	1.994122
C	-0.950753	0.493487	2.825061
H	-1.740012	2.511920	3.013610
C	4.237496	-2.311221	1.813485
H	4.405896	-0.963497	0.136667
H	2.825534	-1.685240	0.283541
C	4.889282	-0.483825	3.437197
H	5.111708	0.900743	1.802756
H	3.985561	1.454593	3.041351
C	3.531324	2.968940	-3.014385
H	1.998780	3.364771	-1.537337
H	1.874340	1.774150	-2.278224
C	5.345246	3.402419	-1.303377
H	3.843872	3.836414	0.183623
H	4.964517	2.568601	0.664084
C	-3.732864	4.110945	-1.827734
H	-3.709571	2.422470	-0.484983
H	-2.491615	2.320188	-1.737362
C	-3.327562	5.752962	0.058349
H	-3.268756	4.177362	1.536743
H	-1.797143	5.141107	1.475304

C	-3.857921	0.651755	3.240986
H	-3.190856	0.527431	1.184633
H	-3.799324	2.117912	1.656454
C	-1.610972	-0.122115	4.067241
H	-0.899540	-0.269570	2.037265
H	0.079416	0.777133	3.063102
C	5.367604	-1.726778	2.671903
H	4.595378	-3.163844	1.224176
H	3.446373	-2.693431	2.475331
H	5.712571	-0.047844	4.017431
H	4.113649	-0.776607	4.160650
C	4.573778	3.972495	-2.501847
H	2.946535	3.405459	-3.833654
H	4.045651	2.089861	-3.429444
H	6.052763	4.145656	-0.914326
H	5.942538	2.538958	-1.631031
C	-4.375926	5.131225	-0.876519
H	-4.494412	3.653262	-2.471352
H	-3.027909	4.631494	-2.493442
H	-3.800385	6.461036	0.750868
H	-2.607661	6.330524	-0.540618
C	-3.053983	-0.543971	3.767474
H	-4.883957	0.347556	2.998291
H	-3.935807	1.414231	4.031136
H	-1.019125	-0.982564	4.405959
H	-1.603047	0.611060	4.887765
H	5.749697	-2.481321	3.370773
H	6.207817	-1.445185	2.019928
H	5.266492	4.249460	-3.306188
H	4.062856	4.896884	-2.192962
H	-4.889244	5.916181	-1.445848
H	-5.143855	4.625798	-0.272027
H	-3.527405	-0.962083	4.664793
H	-3.042473	-1.335830	3.006030
N	-1.369478	-2.994192	0.403631
C	-1.779604	-4.399701	0.518419
C	-0.089632	-2.719938	0.642963
C	-0.964893	-5.125462	1.576379
H	-1.654575	-4.904763	-0.446890
H	-2.846972	-4.432981	0.755735
C	0.846043	-3.681555	1.323657
H	0.193116	-1.675632	0.739112
H	-1.159564	-4.705095	2.578502
H	-1.206149	-6.192056	1.593591
O	0.423118	-5.026688	1.264384
H	0.967783	-3.357927	2.378125
H	1.831460	-3.633557	0.851510

H’

Electronic Energy BS1 = -2945.61204003 Hartree
Electronic Energy BS2 = -2946.28700323 Hartree
Zero-point Energy Correction = 1.152426 Hartree
Thermal Correction to Enthalpy = 1.207817 Hartree
Thermal Correction to Free Energy = 1.066219 Hartree

Chemical symbol X, Y, Z

C	-0.515985	-1.494829	-1.597239
C	0.611851	-2.462833	-1.305640
H	0.234684	-3.445838	-1.623322
C	2.014617	-2.330603	-1.855275
C	2.825057	-3.482499	-1.841670
C	2.591175	-1.145988	-2.340082
C	4.152936	-3.448340	-2.264186
H	2.400700	-4.413260	-1.471206
C	3.916358	-1.110839	-2.785064
H	2.013285	-0.232655	-2.353849
C	4.708443	-2.257304	-2.741131
H	4.752697	-4.354145	-2.227574
H	4.328260	-0.177862	-3.164093
H	5.740246	-2.228280	-3.081011
C	-0.558066	-0.429953	-2.448091
H	0.199173	-0.215378	-3.202076
H	-1.507403	0.090785	-2.574718
B	-1.769881	-2.087928	-0.676980
O	-2.442453	-3.146658	-1.448202
O	-2.829475	-1.096728	-0.394295
C	-3.748620	-2.735384	-1.810265
C	-4.100179	-1.674273	-0.700566
C	-4.658145	-3.969077	-1.823994
C	-3.714393	-2.127001	-3.225102
C	-5.061920	-0.578639	-1.169827
C	-4.677755	-2.324392	0.570297
H	-5.706041	-3.692955	-1.995807
H	-4.346699	-4.643585	-2.629181
H	-4.587626	-4.520022	-0.883175
H	-3.244817	-2.849717	-3.900462
H	-4.717980	-1.898075	-3.602775
H	-3.116683	-1.213527	-3.245847
H	-5.998697	-1.013867	-1.538463
H	-5.310046	0.089773	-0.337704
H	-4.619671	0.023503	-1.966553
H	-4.758337	-1.568141	1.356726
H	-5.675430	-2.745378	0.400924
H	-4.021548	-3.118565	0.933095
Cu	0.028796	0.590254	-0.582068
P	2.035481	1.256101	0.496213
P	-1.173983	2.158888	0.580707
C	1.450482	2.641771	1.624121
C	3.061817	0.177632	1.654119
C	3.215049	2.048771	-0.721463

C	0.160662	3.342490	1.156353
C	-2.376259	3.358131	-0.199273
C	-1.857986	1.454471	2.184739
H	2.248218	3.376061	1.780023
H	1.266737	2.179475	2.598096
C	4.048700	-0.710983	0.866654
C	3.784507	0.882117	2.824150
H	2.301522	-0.487269	2.092286
C	2.416445	2.819212	-1.797262
C	4.352675	2.922708	-0.162435
H	3.669242	1.180059	-1.215612
H	-0.213151	3.989172	1.959512
H	0.373236	3.990740	0.297561
C	-3.448238	2.630556	-1.034879
C	-3.000626	4.411192	0.738481
H	-1.725520	3.894394	-0.910566
C	-3.336102	1.026670	2.076331
C	-1.005813	0.221725	2.581418
H	-1.759490	2.232737	2.958140
C	4.754205	-1.720463	1.785490
H	4.809632	-0.077513	0.389768
H	3.533784	-1.237140	0.060619
C	4.481861	-0.143110	3.736073
H	4.535556	1.580429	2.434191
H	3.084463	1.476182	3.420739
C	3.334941	3.313617	-2.925191
H	1.920382	3.685047	-1.335049
H	1.619841	2.184016	-2.207239
C	5.267861	3.424680	-1.294164
H	3.930854	3.787607	0.369043
H	4.946360	2.361144	0.565491
C	-4.315657	3.630878	-1.812948
H	-4.089664	2.036151	-0.378572
H	-2.969377	1.916901	-1.714358
C	-3.880058	5.397667	-0.050358
H	-3.614646	3.910705	1.497578
H	-2.219379	4.960534	1.277962
C	-3.829469	0.366218	3.375137
H	-3.416274	0.314711	1.249162
H	-3.974185	1.886210	1.846251
C	-1.489928	-0.416938	3.890500
H	-1.085828	-0.535499	1.790032
H	0.052879	0.486224	2.675784
C	5.465527	-1.020645	2.950239
H	5.462200	-2.313928	1.194231
H	4.011923	-2.424128	2.185707
H	4.996569	0.380752	4.551562
H	3.719281	-0.783279	4.203680
C	4.477058	4.178313	-2.372721
H	2.750459	3.873941	-3.665501

H	3.756606	2.443115	-3.449280
H	6.056089	4.064814	-0.878468
H	5.772223	2.561129	-1.752265
C	-4.951334	4.665544	-0.872316
H	-5.091416	3.091275	-2.370286
H	-3.694925	4.151580	-2.557869
H	-4.345794	6.113410	0.638968
H	-3.242004	5.983244	-0.729159
C	-2.959537	-0.834898	3.769625
H	-4.874434	0.055430	3.246455
H	-3.820295	1.106520	4.190205
H	-0.862820	-1.288658	4.116509
H	-1.369056	0.293056	4.723459
H	5.932788	-1.758255	3.614333
H	6.276897	-0.390179	2.556202
H	5.144476	4.493780	-3.184073
H	4.054928	5.095979	-1.936350
H	-5.549929	5.386562	-1.442906
H	-5.642763	4.152221	-0.187721
H	-3.316594	-1.272748	4.710559
H	-3.036793	-1.609824	2.996658
N	-0.968689	-2.701904	0.533569
C	-1.283318	-4.113078	0.795493
C	0.456533	-2.469624	0.276177
C	-0.291821	-4.772062	1.738472
H	-1.307143	-4.715255	-0.125966
H	-2.288551	-4.175241	1.229084
C	1.402634	-3.336297	1.109874
H	0.707748	-1.441245	0.587676
H	-0.305607	-4.288689	2.732037
H	-0.517353	-5.836303	1.862031
O	1.024328	-4.695546	1.189466
H	1.437229	-2.908162	2.129951
H	2.415169	-3.308453	0.701498

TS1

Imaginary Freq = -347.6325 (cm^{-1})
Electronic Energy BS1 = -2949.02900575 Hartree
Electronic Energy BS2 = -2949.70056270 Hartree
Zero-point Energy Correction = 1.201388 Hartree
Thermal Correction to Enthalpy = 1.259507 Hartree
Thermal Correction to Free Energy = 1.108771 Hartree

Chemical symbol X, Y, Z

C	0.740359	1.724536	-1.540890
C	-0.083223	2.839482	-1.092661
H	0.525548	3.725726	-0.917263
C	-1.368691	3.210333	-1.724988
C	-1.654644	4.571360	-1.963323

C	-2.392218	2.287451	-2.032866
C	-2.875585	4.986144	-2.491512
H	-0.892025	5.312226	-1.732135
C	-3.613441	2.698716	-2.568026
H	-2.244871	1.238061	-1.813593
C	-3.866659	4.050799	-2.804504
H	-3.053443	6.045260	-2.663756
H	-4.375887	1.954022	-2.789161
H	-4.818381	4.371437	-3.219772
C	0.317534	0.507175	-2.024059
H	-0.697391	0.324065	-2.356475
H	1.051135	-0.219530	-2.358302
B	2.279739	1.891923	-1.359349
O	3.204961	0.922668	-1.692000
O	2.879544	3.045121	-0.898706
C	4.509124	1.368514	-1.242430
C	4.307228	2.928780	-1.117618
C	5.546162	0.927856	-2.273122
C	4.784138	0.681214	0.100942
C	5.027570	3.581881	0.059186
C	4.612127	3.684639	-2.416725
H	6.541488	1.303855	-2.010304
H	5.589895	-0.166147	-2.301067
H	5.290386	1.279149	-3.274901
H	4.729618	-0.401856	-0.037441
H	5.777478	0.934822	0.485711
H	4.036467	0.960275	0.847614
H	6.112578	3.453378	-0.027078
H	4.809113	4.654381	0.071523
H	4.696833	3.161431	1.011542
H	4.266229	4.717478	-2.313073
H	5.684531	3.695246	-2.638249
H	4.082264	3.235472	-3.262461
Cu	-0.105727	-0.343271	0.210043
N	0.011686	1.282736	1.310969
C	1.285665	1.594112	1.947085
C	-0.664816	2.338512	0.782188
C	1.268797	2.848471	2.830301
H	2.061684	1.740292	1.175512
H	1.594632	0.724228	2.537230
C	-0.649865	3.685689	1.498890
H	-1.662633	2.049791	0.456022
H	0.620079	2.676623	3.702721
H	2.280680	3.048898	3.211896
H	-1.347764	3.620862	2.351089
H	-1.040677	4.467117	0.836470
P	-2.305220	-1.169922	0.283137
C	-1.989390	-2.814939	1.128255
C	-3.389537	-0.236216	1.506215
C	-3.383226	-1.574275	-1.196567

C	-0.726349	-3.539174	0.616531
H	-2.860328	-3.477502	1.066972
H	-1.856707	-2.568410	2.185762
C	-4.243963	0.841250	0.802541
C	-4.267266	-1.046335	2.483259
H	-2.625018	0.290330	2.097526
C	-2.540087	-2.255753	-2.296861
C	-4.679225	-2.370306	-0.951889
H	-3.667372	-0.581089	-1.575914
H	-0.499869	-4.395341	1.264667
H	-0.907598	-3.942879	-0.385544
P	0.776535	-2.411974	0.471043
C	-4.897724	1.778550	1.828841
H	-5.034834	0.355871	0.212710
H	-3.644868	1.425913	0.099996
C	-4.933381	-0.113794	3.511803
H	-5.045031	-1.588514	1.928938
H	-3.676287	-1.801474	3.012846
C	-3.340113	-2.394798	-3.600986
H	-2.245600	-3.259288	-1.956718
H	-1.613415	-1.696626	-2.467602
C	-5.484748	-2.525203	-2.254856
H	-4.429051	-3.367655	-0.562615
H	-5.297586	-1.881368	-0.192489
C	1.708324	-3.265993	-0.913165
C	1.696852	-2.762295	2.069966
C	-5.753531	0.992383	2.831976
H	-5.504565	2.530876	1.309805
H	-4.108246	2.324966	2.365166
H	-5.567278	-0.699486	4.190272
H	-4.149305	0.346979	4.130614
C	-4.646296	-3.169267	-3.368767
H	-2.730250	-2.892506	-4.365653
H	-3.572423	-1.391144	-3.986122
H	-6.390456	-3.116122	-2.067195
H	-5.820547	-1.530733	-2.584239
C	2.914967	-2.459331	-1.433638
C	2.113374	-4.728954	-0.649669
H	0.953658	-3.261899	-1.717047
C	3.058703	-2.038045	2.082424
C	0.814500	-2.304005	3.254455
H	1.866119	-3.845720	2.157406
H	-6.181281	1.666135	3.585211
H	-6.601442	0.535407	2.299507
H	-5.227638	-3.227237	-4.297508
H	-4.403704	-4.204157	-3.083579
C	3.478116	-3.092491	-2.714911
H	3.708289	-2.458292	-0.675672
H	2.650527	-1.412598	-1.599857
C	2.698924	-5.373030	-1.919499

H	2.868012	-4.754984	0.149627
H	1.257645	-5.316959	-0.295158
C	3.764215	-2.121876	3.444856
H	2.901828	-0.985120	1.817204
H	3.713344	-2.458585	1.312799
C	1.546090	-2.389401	4.603083
H	0.488276	-1.268928	3.076671
H	-0.093620	-2.916209	3.301451
C	3.874942	-4.558227	-2.480706
H	4.340625	-2.512534	-3.068057
H	2.718848	-3.039814	-3.509414
H	3.013883	-6.402782	-1.706677
H	1.907930	-5.436876	-2.681506
C	2.866077	-1.609179	4.576966
H	4.701441	-1.551996	3.405733
H	4.039126	-3.167670	3.648931
H	0.890677	-2.013715	5.399065
H	1.755965	-3.443884	4.837350
H	4.241041	-5.012749	-3.409995
H	4.708844	-4.592260	-1.763393
H	3.380051	-1.691995	5.542860
H	2.654038	-0.541575	4.419047
C	0.740880	4.049768	2.034347
H	1.411851	4.196306	1.175205
C	0.716252	5.336693	2.862247
H	1.719412	5.596337	3.223674
H	0.339308	6.181913	2.272744
H	0.064174	5.224787	3.738852

TS2

Imaginary Freq = -348.3665 (cm^{-1})
Electronic Energy BS1 = -2949.02614365 Hartree
Electronic Energy BS2 = -2949.69683828 Hartree
Zero-point Energy Correction = 1.202139 Hartree
Thermal Correction to Enthalpy = 1.259972 Hartree
Thermal Correction to Free Energy = 1.111188 Hartree

Chemical symbol X, Y, Z

C	0.596086	1.856452	-1.447792
C	-0.262988	2.912989	-0.925635
H	0.315969	3.800836	-0.680520
C	-1.554664	3.298082	-1.535898
C	-1.882109	4.667279	-1.641476
C	-2.546530	2.380459	-1.946161
C	-3.112107	5.095906	-2.135458
H	-1.144234	5.403987	-1.330550
C	-3.777478	2.806671	-2.447097
H	-2.367977	1.317968	-1.842749
C	-4.072610	4.166950	-2.547503

H	-3.321273	6.161167	-2.202782
H	-4.514059	2.065188	-2.750547
H	-5.032102	4.498182	-2.935661
C	0.219680	0.652286	-1.997591
H	-0.792120	0.437282	-2.319050
H	0.979419	-0.022452	-2.380830
B	2.131556	2.062489	-1.274208
O	3.082971	1.117698	-1.602773
O	2.702911	3.234532	-0.826361
C	4.374052	1.604055	-1.155443
C	4.131444	3.158214	-1.059820
C	5.428232	1.171388	-2.171457
C	4.655424	0.952810	0.203834
C	4.846201	3.856877	0.093610
C	4.397690	3.894298	-2.378522
H	6.410999	1.580746	-1.911193
H	5.503155	0.078783	-2.177028
H	5.168314	1.495385	-3.181385
H	4.634514	-0.133463	0.087190
H	5.636853	1.243229	0.593075
H	3.890932	1.226012	0.936137
H	5.933191	3.755922	-0.003756
H	4.598093	4.922871	0.085551
H	4.538373	3.449238	1.058922
H	4.018500	4.917108	-2.294326
H	5.467119	3.935236	-2.610617
H	3.875607	3.408249	-3.208587
Cu	-0.049805	-0.349378	0.204007
N	-0.069036	1.226845	1.374689
C	1.109316	1.652967	2.117387
C	-0.847359	2.232578	0.891939
C	0.796138	2.699247	3.195935
H	1.868491	2.063688	1.427387
H	1.561128	0.769507	2.581421
C	-1.086957	3.482555	1.740355
H	-1.780079	1.850551	0.480772
H	0.121672	2.240670	3.933009
H	1.715655	2.984912	3.726813
H	-1.906795	3.223890	2.430217
H	-1.464702	4.298484	1.113602
P	-2.216747	-1.292597	0.222656
C	-1.824016	-2.939080	1.031268
C	-3.381310	-0.459546	1.445477
C	-3.237435	-1.718711	-1.291037
C	-0.521687	-3.585289	0.514597
H	-2.658978	-3.644078	0.947632
H	-1.712346	-2.709985	2.095076
C	-4.296335	0.574762	0.753184
C	-4.218574	-1.347441	2.390099
H	-2.662875	0.102010	2.061851

C	-2.332996	-2.313082	-2.393367
C	-4.484836	-2.601476	-1.096240
H	-3.576198	-0.733854	-1.646866
H	-0.253857	-4.440795	1.147692
H	-0.674746	-3.978600	-0.496102
P	0.920732	-2.378710	0.403859
C	-5.022366	1.444435	1.790495
H	-5.046226	0.051556	0.142597
H	-3.729439	1.214460	0.072494
C	-4.957881	-0.484706	3.429681
H	-4.952155	-1.925066	1.811601
H	-3.587954	-2.075423	2.911824
C	-3.098825	-2.462756	-3.716812
H	-1.983185	-3.306310	-2.076548
H	-1.439835	-1.693193	-2.529126
C	-5.255596	-2.766682	-2.418767
H	-4.178560	-3.592728	-0.732274
H	-5.146144	-2.176601	-0.334388
C	1.909538	-3.158657	-0.984656
C	1.841242	-2.715074	2.005990
C	-5.838626	0.582566	2.763701
H	-5.668908	2.168564	1.279421
H	-4.276239	2.027885	2.349503
H	-5.561637	-1.126345	4.084639
H	-4.213829	0.010400	4.071052
C	-4.357993	-3.323456	-3.533709
H	-2.444918	-2.898737	-4.482808
H	-3.386564	-1.464653	-4.078261
H	-6.125537	-3.418385	-2.266459
H	-5.647231	-1.785642	-2.725900
C	3.048397	-2.258200	-1.502165
C	2.423611	-4.587097	-0.723017
H	1.156556	-3.210718	-1.788324
C	3.162840	-1.922343	2.047918
C	0.927313	-2.327814	3.191665
H	2.064856	-3.790086	2.072074
H	-6.317874	1.210018	3.525880
H	-6.648951	0.085271	2.209300
H	-4.917267	-3.389442	-4.475379
H	-4.056265	-4.349375	-3.273646
C	3.669673	-2.846389	-2.778126
H	3.833892	-2.186886	-0.739192
H	2.696758	-1.238079	-1.672990
C	3.064424	-5.181077	-1.990552
H	3.173976	-4.558476	0.080111
H	1.612430	-5.238452	-0.374185
C	3.863067	-2.006874	3.412865
H	2.950647	-0.872104	1.812290
H	3.842712	-2.285658	1.270518
C	1.651207	-2.409387	4.544807

H	0.552092	-1.306293	3.033660
H	0.050137	-2.984716	3.216583
C	4.178341	-4.276460	-2.540280
H	4.486633	-2.200886	-3.125847
H	2.914606	-2.854318	-3.578402
H	3.455719	-6.184366	-1.778073
H	2.286131	-5.302709	-2.758578
C	2.932628	-1.566873	4.549609
H	4.771521	-1.391148	3.395858
H	4.187224	-3.042949	3.592737
H	0.971974	-2.083983	5.342956
H	1.909264	-3.457870	4.756882
H	4.587696	-4.699867	-3.466213
H	5.005511	-4.245742	-1.815061
H	3.442595	-1.649554	5.517636
H	2.670799	-0.506979	4.416626
C	0.118178	3.942245	2.587349
C	1.113536	4.823456	1.815824
H	1.887547	5.206669	2.494147
H	1.617275	4.286222	1.009339
H	0.602651	5.686660	1.370881
H	-0.277059	4.555216	3.410570

14.9.3. Cartesian coordinates for the phosphine-free Cu system (Figures S19 and S20)

J-0

Electronic Energy BS1 = -1197.07617094 Hartree
Electronic Energy BS2 = -1197.39271205 Hartree
Zero-point Energy Correction = 0.451266 Hartree
Thermal Correction to Enthalpy = 0.480468 Hartree
Thermal Correction to Free Energy = 0.391393 Hartree

Chemical symbol X, Y, Z

Cu	0.556711	-1.291901	0.054771
C	-1.132870	-2.558216	0.324022
C	-1.312568	-1.155272	0.128187
H	-1.327565	-2.934596	1.336174
C	-2.209823	-0.190922	-0.088811
H	-1.835465	0.802525	-0.321749
H	-1.539336	-3.198618	-0.469361
C	-3.675014	-0.329732	-0.071034
C	-4.458195	0.793428	-0.396703
C	-4.339417	-1.526117	0.260111
C	-5.850337	0.726425	-0.398600
H	-3.961599	1.726556	-0.653525
C	-5.730583	-1.592045	0.259922
H	-3.756318	-2.402661	0.521864
C	-6.494508	-0.468304	-0.070211

H	-6.432433	1.607619	-0.656040
H	-6.223472	-2.525544	0.520191
H	-7.579787	-0.524050	-0.069717
B	0.877977	0.660833	0.021565
O	0.518724	1.531260	-0.992538
O	1.485740	1.331593	1.069658
C	1.118372	2.827937	-0.707306
C	1.320293	2.761520	0.846384
C	0.169325	3.919043	-1.193028
C	2.439487	2.882518	-1.484577
C	2.557279	3.482495	1.372807
C	0.076053	3.186630	1.636391
H	0.546928	4.911890	-0.921434
H	0.081466	3.872695	-2.283272
H	-0.830191	3.797279	-0.769221
H	2.232965	2.712161	-2.545617
H	2.933878	3.853961	-1.376539
H	3.124028	2.099771	-1.143078
H	2.508726	4.554029	1.145633
H	2.616891	3.365749	2.459692
H	3.472630	3.072927	0.940059
H	0.217589	2.919858	2.688122
H	-0.098115	4.266049	1.569956
H	-0.813912	2.662473	1.274032
O	2.046719	-2.532520	0.015221
C	3.411095	-2.175313	-0.154484
Li	0.812588	-3.704004	0.219781
C	4.223870	-3.478786	-0.218455
C	3.570719	-1.390589	-1.466057
C	3.865468	-1.323608	1.040628
H	5.294200	-3.282147	-0.351289
H	4.095336	-4.049356	0.710084
H	3.883210	-4.096812	-1.058827
H	3.229430	-1.998138	-2.312205
H	2.955746	-0.484891	-1.433460
H	4.614462	-1.101872	-1.639185
H	3.268497	-0.408880	1.100380
H	3.723987	-1.884131	1.972106
H	4.924623	-1.050087	0.956833

$\mathbf{TS_{J\text{-}K'\text{-}0}}$

Imaginary Freq = -138.8054 (cm^{-1})
Electronic Energy BS1 = -1197.06901403 Hartree
Electronic Energy BS2 = -1197.38261047 Hartree
Zero-point Energy Correction = 0.450839 Hartree
Thermal Correction to Enthalpy = 0.479336 Hartree
Thermal Correction to Free Energy = 0.392135 Hartree

Chemical symbol X, Y, Z			
Cu	1.000329	-1.172520	-0.128913
C	-0.575859	-2.181912	0.979519
C	-0.843800	-1.003900	0.168544
H	-0.448287	-1.990236	2.053319
C	-1.909608	-0.523613	-0.507707
H	-1.734812	0.221927	-1.277888
H	-1.266097	-3.007279	0.780391
C	-3.320402	-0.880401	-0.302010
C	-4.259147	-0.476120	-1.272526
C	-3.802522	-1.566095	0.831304
C	-5.615160	-0.764865	-1.133501
H	-3.910387	0.066217	-2.148728
C	-5.159559	-1.845874	0.973512
H	-3.106702	-1.862477	1.608168
C	-6.074196	-1.454575	-0.008987
H	-6.315033	-0.447711	-1.902628
H	-5.507324	-2.370679	1.860090
H	-7.131355	-1.679619	0.103894
B	0.509277	0.741959	-0.043319
O	0.204973	1.585747	-1.089970
O	0.722592	1.417787	1.139976
C	0.396902	2.952250	-0.618538
C	0.263669	2.789030	0.937893
C	-0.660760	3.839295	-1.267366
C	1.802034	3.375335	-1.063077
C	1.141357	3.722256	1.764407
C	-1.188829	2.836436	1.427907
H	-0.604987	4.860471	-0.872560
H	-0.494150	3.880999	-2.348524
H	-1.666960	3.451083	-1.094821
H	1.876544	3.268104	-2.149516
H	2.012659	4.417415	-0.800136
H	2.563687	2.735796	-0.606155
H	0.881624	4.769580	1.570901
H	0.988413	3.523574	2.829945
H	2.200777	3.576089	1.542673
H	-1.216794	2.525802	2.476782
H	-1.607967	3.845378	1.352302
H	-1.817404	2.146198	0.857074
O	2.578400	-2.205136	0.059202
C	3.882211	-1.697029	-0.192495
Li	1.448019	-3.279377	0.864414
C	4.875867	-2.852567	-0.002944
C	3.945772	-1.167986	-1.633256
C	4.174330	-0.564389	0.804867
H	5.908130	-2.531900	-0.185258
H	4.815399	-3.238423	1.022570
H	4.640277	-3.668888	-0.695765
H	3.702119	-1.970326	-2.338550

H	3.209304	-0.365606	-1.763457
H	4.939800	-0.774231	-1.876756
H	3.417959	0.222551	0.702293
H	4.123164	-0.944541	1.831843
H	5.166880	-0.126684	0.643389

K'-0

Electronic Energy BS1 = -1197.15482717 Hartree
Electronic Energy BS2 = -1197.46539105 Hartree
Zero-point Energy Correction = 0.453085 Hartree
Thermal Correction to Enthalpy = 0.481728 Hartree
Thermal Correction to Free Energy = 0.394829 Hartree

Chemical symbol X, Y, Z

Cu	-0.653079	0.748733	-0.518883
C	-0.946212	-1.224347	2.159148
C	-0.663641	-1.298169	0.818702
H	-0.188000	-1.482432	2.896873
C	-1.546185	-1.009818	-0.343264
H	-1.257608	-1.622257	-1.203395
H	-1.944833	-1.029365	2.538257
C	-3.026142	-0.934653	-0.178809
C	-3.870437	-1.741101	-0.962904
C	-3.640619	-0.050227	0.730843
C	-5.259229	-1.673635	-0.843451
H	-3.424772	-2.431705	-1.676012
C	-5.026948	0.012554	0.860717
H	-3.013835	0.614907	1.320517
C	-5.847757	-0.800743	0.074127
H	-5.883400	-2.310157	-1.466539
H	-5.469007	0.707412	1.571154
H	-6.929004	-0.749843	0.171221
B	0.833273	-1.524326	0.437437
O	1.350707	-2.160138	-0.649328
O	1.829224	-0.894114	1.202418
C	2.720916	-1.690463	-0.828186
C	3.129400	-1.264311	0.630331
C	3.549388	-2.826569	-1.416805
C	2.648966	-0.507537	-1.801283
C	4.054240	-0.055190	0.712923
C	3.657680	-2.421500	1.480520
H	4.603369	-2.535906	-1.490592
H	3.187233	-3.057962	-2.422914
H	3.474945	-3.734070	-0.813798
H	2.165459	-0.842409	-2.723614
H	3.647412	-0.131216	-2.047576
H	2.051657	0.311209	-1.387288
H	4.999464	-0.261360	0.199181
H	4.282926	0.169031	1.760680

H	3.596421	0.828762	0.262421
H	3.724376	-2.099412	2.524318
H	4.653077	-2.734254	1.150078
H	2.984923	-3.282943	1.431056
O	0.588477	1.956505	0.303461
C	0.766914	3.311770	-0.067440
Li	0.823712	0.740200	1.589725
C	1.849894	3.915931	0.841584
C	-0.560654	4.065796	0.112693
C	1.217358	3.374715	-1.536427
H	2.049183	4.965986	0.597501
H	2.786224	3.354581	0.734411
H	1.533632	3.865889	1.891529
H	-0.893207	3.993406	1.154532
H	-1.332183	3.608607	-0.519480
H	-0.472558	5.125849	-0.155131
H	0.459595	2.901237	-2.173373
H	2.157360	2.826463	-1.666043
H	1.364089	4.406681	-1.877250

$TS_{K'\text{-}K\text{-}0}$

Imaginary Freq = -133.0368 (cm^{-1})
Electronic Energy BS1 = -1197.12878495 Hartree
Electronic Energy BS2 = -1197.44717047 Hartree
Zero-point Energy Correction = 0.452260 Hartree
Thermal Correction to Enthalpy = 0.480433 Hartree
Thermal Correction to Free Energy = 0.394182 Hartree

Chemical symbol X, Y, Z

Cu	0.732336	0.834466	-0.534003
C	0.681281	-0.605370	-2.190900
C	0.538804	-1.171608	-0.873831
H	-0.171535	-0.717074	-2.859777
C	1.487310	-1.364804	0.160766
H	1.070763	-1.755383	1.085097
H	1.631729	-0.590722	-2.711344
C	2.938709	-1.252657	0.162413
C	3.644118	-1.713600	1.303325
C	3.717394	-0.688848	-0.879110
C	5.026777	-1.624656	1.396870
H	3.077999	-2.149386	2.124124
C	5.105952	-0.596921	-0.777673
H	3.234360	-0.298019	-1.764487
C	5.775329	-1.064424	0.353713
H	5.528383	-1.994412	2.288321
H	5.667905	-0.153979	-1.596937
H	6.857263	-0.995105	0.425202
B	-0.946561	-1.360643	-0.431963
O	-1.420894	-2.188040	0.541705

O	-1.986821	-0.590209	-1.001449
C	-2.781771	-1.785295	0.864983
C	-3.255171	-1.099923	-0.467544
C	-3.575242	-3.025307	1.261450
C	-2.675440	-0.808631	2.041224
C	-4.212171	0.072862	-0.283108
C	-3.794225	-2.088529	-1.504217
H	-4.627436	-2.771359	1.432684
H	-3.167714	-3.437651	2.189230
H	-3.519318	-3.800941	0.494808
H	-2.155619	-1.307196	2.864389
H	-3.662049	-0.491588	2.394330
H	-2.095643	0.077221	1.766513
H	-5.129989	-0.257637	0.214826
H	-4.495505	0.486052	-1.258409
H	-3.763149	0.869706	0.315715
H	-3.906500	-1.577092	-2.465516
H	-4.770414	-2.484090	-1.207012
H	-3.103496	-2.925568	-1.642317
O	-0.467840	2.341057	-0.521293
C	-0.471013	3.367772	0.452112
Li	-1.356373	1.204842	-1.557408
C	-1.419343	4.475253	-0.034992
C	0.955382	3.920047	0.603831
C	-0.961049	2.796550	1.792702
H	-1.469110	5.311043	0.673052
H	-2.433217	4.073643	-0.160672
H	-1.079135	4.858459	-1.003461
H	1.315961	4.287637	-0.362948
H	1.627493	3.116413	0.933968
H	1.007072	4.735765	1.335093
H	-0.323396	1.952581	2.083803
H	-1.989939	2.429441	1.691503
H	-0.940965	3.544484	2.594562

K-0

Electronic Energy BS1 = -1197.15925696 Hartree
Electronic Energy BS2 = -1197.47458768 Hartree
Zero-point Energy Correction = 0.453674 Hartree
Thermal Correction to Enthalpy = 0.482335 Hartree
Thermal Correction to Free Energy = 0.393711 Hartree

Chemical symbol X, Y, Z

Cu	0.691131	1.483500	-1.011933
C	0.777482	-0.252340	-1.863025
C	0.341597	-1.213349	-0.809532
H	0.039126	-0.211116	-2.677709
C	1.132424	-2.001912	-0.014086
H	0.617008	-2.694393	0.651379

H	1.751183	-0.501806	-2.296121
C	2.589872	-2.060311	0.100445
C	3.175125	-3.201556	0.691402
C	3.454377	-1.015407	-0.292258
C	4.552797	-3.312233	0.853463
H	2.527810	-4.012163	1.020203
C	4.833615	-1.121956	-0.115310
H	3.038429	-0.104872	-0.707008
C	5.393585	-2.271416	0.446576
H	4.973337	-4.209198	1.301769
H	5.475299	-0.298266	-0.419499
H	6.469939	-2.353692	0.572956
B	-1.157070	-1.313466	-0.434874
O	-1.806698	-2.453922	-0.038892
O	-2.049047	-0.204889	-0.376875
C	-3.090333	-2.100137	0.532673
C	-3.403455	-0.742852	-0.183063
C	-4.083026	-3.219844	0.237511
C	-2.879905	-1.949660	2.044513
C	-4.217560	0.252616	0.637562
C	-4.007030	-0.914050	-1.579448
H	-5.089283	-2.944616	0.573393
H	-3.780479	-4.126151	0.770459
H	-4.117330	-3.452611	-0.828799
H	-2.456027	-2.879768	2.433236
H	-3.820779	-1.750551	2.567811
H	-2.173963	-1.142332	2.266744
H	-5.199115	-0.167748	0.880773
H	-4.391618	1.171176	0.063104
H	-3.722044	0.511683	1.578180
H	-3.991442	0.048369	-2.100717
H	-5.043222	-1.261855	-1.524393
H	-3.427481	-1.629751	-2.169534
O	-0.237929	2.781204	0.061118
C	0.362367	3.911242	0.666817
Li	-1.504171	1.582776	0.023685
C	-0.727022	4.674288	1.437711
C	0.962983	4.807873	-0.428321
C	1.464132	3.439871	1.630923
H	-0.328042	5.569520	1.929222
H	-1.163844	4.028184	2.210209
H	-1.524782	4.984214	0.751969
H	0.180190	5.117329	-1.129553
H	1.716714	4.243825	-0.991838
H	1.437697	5.703716	-0.009687
H	2.220278	2.870468	1.076769
H	1.037321	2.776793	2.392232
H	1.957433	4.280790	2.133704

L-0

Electronic Energy BS1 = -1904.57740187 Hartree
Electronic Energy BS2 = -1905.09758112 Hartree
Zero-point Energy Correction = 0.686492 Hartree
Thermal Correction to Enthalpy = 0.729070 Hartree
Thermal Correction to Free Energy = 0.610741 Hartree

Chemical symbol X, Y, Z

Cu	-0.206699	-0.015103	0.008579
B	2.792041	0.228853	-1.183106
O	3.568724	0.518815	-0.053970
O	3.211479	-0.907982	-1.812603
C	4.490219	-1.306063	-1.235839
C	4.473390	-0.608847	0.179512
C	3.842513	-1.469927	1.276295
C	5.815965	-0.054445	0.644320
C	4.548489	-2.830767	-1.191477
C	5.572296	-0.756044	-2.171870
H	4.507113	-3.224880	-2.211732
H	3.717582	-3.256696	-0.625766
H	5.487783	-3.163539	-0.735493
H	5.526632	0.336107	-2.228043
H	5.403018	-1.151878	-3.177459
H	6.574948	-1.049623	-1.845115
H	2.887044	-1.888850	0.957958
H	3.660779	-0.853698	2.162381
H	4.504760	-2.295212	1.556486
H	5.698659	0.418620	1.624778
H	6.206440	0.693134	-0.049219
H	6.550399	-0.861490	0.742590
C	1.539373	1.111018	-1.477880
C	0.358117	0.384855	-1.901256
C	1.693455	2.453362	-1.116529
H	0.587246	-0.548258	-2.422785
H	-0.423394	0.946508	-2.416457
C	0.705101	3.515298	-0.992072
H	2.714969	2.776563	-0.902601
C	-0.696353	3.315328	-1.019922
C	1.156142	4.835908	-0.741883
C	-1.579382	4.372761	-0.800471
H	-1.099150	2.322768	-1.167126
C	0.271653	5.887214	-0.532961
H	2.228259	5.024582	-0.713396
C	-1.110694	5.664920	-0.561010
H	-2.649667	4.177872	-0.815706
H	0.659123	6.886315	-0.346569
H	-1.804841	6.485199	-0.398985
N	-1.232801	-2.284099	0.051474
C	-1.065196	-3.018293	1.311384
C	-0.645100	-3.043370	-1.057346

C	0.441731	-3.106501	1.560063
H	-1.513021	-4.021696	1.246625
H	-1.547156	-2.451031	2.108153
C	0.851037	-3.185286	-0.763051
H	-0.789668	-2.470061	-1.976088
H	-1.122661	-4.030194	-1.153500
H	0.835596	-2.085847	1.676599
H	0.641059	-3.675837	2.472054
O	1.094456	-3.801622	0.497655
H	1.317258	-2.194237	-0.805322
H	1.320027	-3.820349	-1.519385
O	-2.524803	0.018734	0.211408
C	-3.173271	-0.959498	-0.117261
O	-2.657940	-2.217292	-0.221616
C	-4.618330	-0.923974	-0.451087
C	-5.342841	-2.078322	-0.786557
C	-5.260244	0.323683	-0.422247
C	-6.699123	-1.979462	-1.090674
H	-4.844253	-3.040705	-0.806819
C	-6.615020	0.415371	-0.729313
H	-4.682274	1.203491	-0.159400
C	-7.335634	-0.735340	-1.063285
H	-7.260139	-2.872778	-1.349915
H	-7.109645	1.382149	-0.708865
H	-8.393194	-0.662576	-1.302215
O	0.644690	0.472792	1.761662
C	-0.064778	0.996266	2.869201
C	0.956587	1.356314	3.964582
C	-1.042716	-0.061750	3.408085
C	-0.853279	2.251392	2.450488
H	1.544386	0.470551	4.233745
H	1.648530	2.128967	3.600063
H	0.473715	1.743731	4.870271
H	-1.596228	0.298372	4.284169
H	-1.762943	-0.313700	2.623082
H	-0.494910	-0.968184	3.692741
H	-0.178090	3.016471	2.048806
H	-1.562376	1.993655	1.657750
H	-1.407040	2.687827	3.291926
Li	1.943828	1.309497	0.864754

$TS_{L\text{-}M'\text{-}0}$

Imaginary Freq = -248.5694 (cm^{-1})
Electronic Energy BS1 = -1904.55123435 Hartree
Electronic Energy BS2 = -1905.07105450 Hartree
Zero-point Energy Correction = 0.684267 Hartree
Thermal Correction to Enthalpy = 0.726904 Hartree
Thermal Correction to Free Energy = 0.607594 Hartree

Chemical symbol X, Y, Z			
Cu	0.073886	-0.766857	0.631413
B	1.871615	1.327858	-1.493412
O	2.860824	2.280639	-1.549488
O	2.360839	0.044217	-1.630867
C	3.779874	0.142317	-1.922029
C	4.127210	1.583918	-1.393470
C	4.443851	1.611718	0.107648
C	5.199557	2.322825	-2.186340
C	4.495792	-0.996275	-1.200792
C	3.931531	0.000440	-3.440518
H	4.194314	-1.955682	-1.634514
H	4.244382	-1.010996	-0.137806
H	5.582482	-0.902719	-1.306497
H	3.415993	0.813412	-3.960925
H	3.478256	-0.944822	-3.755385
H	4.983640	-0.000176	-3.743692
H	3.649725	1.117777	0.675828
H	4.494857	2.654149	0.437183
H	5.400756	1.129122	0.332505
H	5.355697	3.316711	-1.755395
H	4.910048	2.450260	-3.231731
H	6.150725	1.779606	-2.147415
C	0.367204	1.611760	-1.178095
C	-0.456589	0.446038	-1.147223
C	0.055603	2.909874	-0.761230
H	-0.118773	-0.364705	-1.792235
H	-1.537576	0.555838	-1.167429
C	-1.146143	3.406502	-0.104253
H	0.840354	3.653111	-0.886933
C	-2.175192	2.584302	0.440088
C	-1.259369	4.804954	0.135843
C	-3.231467	3.139266	1.171999
H	-2.169784	1.509271	0.306175
C	-2.320442	5.343431	0.846495
H	-0.488150	5.460840	-0.261702
C	-3.320953	4.512971	1.376465
H	-3.994635	2.475308	1.570553
H	-2.374054	6.419226	0.995351
H	-4.150777	4.937722	1.934073
N	-0.228736	-2.567042	0.122152
C	-0.609765	-3.615491	1.066437
C	0.580632	-3.112178	-0.961499
C	0.689867	-4.200466	1.654426
H	-1.165197	-4.411103	0.555693
H	-1.228537	-3.168828	1.845659
C	1.855884	-3.692065	-0.313553
H	0.844907	-2.310870	-1.653672
H	0.042648	-3.909138	-1.488112
H	1.207751	-3.433229	2.246497

H	0.444634	-5.045382	2.304608
O	1.531073	-4.699694	0.627804
H	2.425561	-2.876578	0.155639
H	2.475308	-4.161145	-1.083892
O	-2.389039	-1.090655	0.986199
C	-2.652135	-1.594031	-0.114532
O	-1.870952	-2.420766	-0.765450
C	-3.921242	-1.244582	-0.839210
C	-4.213432	-1.754031	-2.111509
C	-4.818784	-0.365987	-0.216974
C	-5.392304	-1.381507	-2.756252
H	-3.511881	-2.431634	-2.585935
C	-5.995133	0.005113	-0.864488
H	-4.572683	0.015174	0.768437
C	-6.283338	-0.501939	-2.135028
H	-5.615462	-1.774535	-3.744428
H	-6.685511	0.691505	-0.381817
H	-7.200156	-0.210718	-2.640892
O	0.932591	0.479415	1.833203
C	1.439217	0.094512	3.097413
C	2.677182	-0.797593	2.901239
C	0.356391	-0.664192	3.884377
C	1.837538	1.374008	3.853716
H	2.415795	-1.647774	2.261943
H	3.475708	-0.238616	2.402575
H	3.063906	-1.180930	3.853335
H	0.702428	-0.956473	4.883438
H	-0.537220	-0.038695	3.987612
H	0.060870	-1.568796	3.339312
H	2.566975	1.942625	3.265048
H	0.956537	2.007596	4.019285
H	2.280803	1.149347	4.831273
Li	0.045587	1.815625	1.114436

M'-0

Electronic Energy BS1 = -1904.62544165 Hartree
Electronic Energy BS2 = -1905.14844291 Hartree
Zero-point Energy Correction = 0.686165 Hartree
Thermal Correction to Enthalpy = 0.729161 Hartree
Thermal Correction to Free Energy = 0.609601 Hartree

Chemical symbol X, Y, Z

Cu	0.124908	-0.856030	0.705532
B	2.668009	0.911530	-1.053170
O	3.479295	2.014937	-1.133041
O	3.355589	-0.248522	-0.784563
C	4.775711	0.045989	-0.903742
C	4.816022	1.613261	-0.714801
C	4.974215	2.049117	0.746156

C	5.831921	2.340307	-1.591631
C	5.522170	-0.753033	0.160438
C	5.193849	-0.410077	-2.306181
H	5.417058	-1.823198	-0.044652
H	5.124347	-0.560175	1.159055
H	6.589733	-0.506537	0.152723
H	4.647484	0.141949	-3.077301
H	4.955618	-1.472136	-2.418282
H	6.267340	-0.274056	-2.471879
H	4.251610	1.546737	1.393425
H	4.793617	3.126044	0.815075
H	5.982775	1.840332	1.117463
H	5.773236	3.417035	-1.404468
H	5.640486	2.172112	-2.653419
H	6.850010	2.007729	-1.359918
C	1.117879	0.939676	-1.198101
C	0.439203	-0.356686	-1.230520
C	0.500858	2.157043	-1.157327
H	1.038740	-1.158525	-1.652099
H	-0.615148	-0.431466	-1.499579
C	-0.940095	2.443076	-1.124010
H	1.144837	3.026985	-1.032503
C	-1.872684	1.831512	-1.980724
C	-1.418445	3.367894	-0.167603
C	-3.235171	2.103706	-1.862688
H	-1.530939	1.134548	-2.736505
C	-2.787101	3.615396	-0.031924
H	-0.705743	3.876675	0.476856
C	-3.701626	2.976574	-0.878384
H	-3.938679	1.598553	-2.515623
H	-3.136539	4.317164	0.721324
H	-4.767128	3.154466	-0.769463
N	0.412238	-2.600844	0.334066
C	-0.471398	-3.330992	-0.561006
C	1.785168	-3.056166	0.169442
C	-0.373561	-4.829779	-0.228596
H	-0.174971	-3.195523	-1.616491
H	-1.493751	-2.967879	-0.454061
C	1.838369	-4.560728	0.497360
H	2.448102	-2.500373	0.839161
H	2.148023	-2.916402	-0.862924
H	-0.750336	-5.005291	0.792375
H	-0.970664	-5.415355	-0.934019
O	0.966342	-5.292463	-0.345844
H	1.570771	-4.712478	1.556707
H	2.846896	-4.951768	0.326692
O	-2.435685	-0.385833	0.645733
C	-3.174259	-0.719146	-0.360579
O	-2.752529	-1.199874	-1.425114
C	-4.661500	-0.461005	-0.214785

C	-5.497674	-0.632891	-1.325702
C	-5.215479	-0.021324	0.993953
C	-6.859570	-0.345816	-1.238273
H	-5.051977	-0.988539	-2.249181
C	-6.579299	0.261337	1.086523
H	-4.567457	0.086423	1.858205
C	-7.403213	0.106214	-0.031852
H	-7.498975	-0.475633	-2.107972
H	-7.000907	0.598875	2.030212
H	-8.464807	0.330004	0.038134
O	-0.048610	0.678717	1.822299
C	0.969595	1.068692	2.728566
C	2.116830	0.040802	2.721250
C	0.348027	1.133603	4.133951
C	1.507362	2.451877	2.321471
H	1.741038	-0.946931	3.016316
H	2.550185	-0.045144	1.718279
H	2.915575	0.323397	3.418121
H	1.082621	1.427605	4.893865
H	-0.469922	1.865159	4.150502
H	-0.065191	0.154998	4.401647
H	1.917154	2.418717	1.308603
H	0.694317	3.187629	2.334888
H	2.292010	2.800226	3.004329
Li	-1.706784	1.106964	1.287830

J-1

Electronic Energy BS1 = -1429.59052835 Hartree
Electronic Energy BS2 = -1429.97140831 Hartree
Zero-point Energy Correction = 0.570240 Hartree
Thermal Correction to Enthalpy = 0.606098 Hartree
Thermal Correction to Free Energy = 0.500650 Hartree

Chemical symbol X, Y, Z

Cu	-0.322722	-0.929631	0.519315
C	-1.872589	-2.343582	0.340436
C	-2.228303	-1.024825	0.177045
H	-2.024588	-2.851211	1.293226
C	-3.089976	-0.046124	-0.089298
H	-2.714913	0.973705	-0.111178
H	-1.716194	-2.989240	-0.523823
C	-4.526339	-0.222965	-0.366441
C	-5.309961	0.913874	-0.634296
C	-5.157182	-1.481181	-0.377591
C	-6.673505	0.801614	-0.904222
H	-4.837424	1.893674	-0.628895
C	-6.518580	-1.592951	-0.646726
H	-4.567032	-2.369392	-0.172454
C	-7.285500	-0.453320	-0.911893

H	-7.258301	1.695186	-1.108306
H	-6.986387	-2.574516	-0.650019
H	-8.348032	-0.544345	-1.121590
B	0.090316	1.068366	0.541166
O	-0.619093	2.174241	0.107694
O	1.364256	1.518257	1.000109
C	0.260573	3.326898	0.009192
C	1.395133	2.978547	1.030285
C	-0.540153	4.583348	0.338390
C	0.756158	3.376952	-1.443106
C	2.793177	3.448587	0.639296
C	1.066909	3.392267	2.468439
H	0.107817	5.467843	0.349697
H	-1.311981	4.735065	-0.422802
H	-1.036585	4.498361	1.307361
H	-0.110894	3.406156	-2.109543
H	1.373769	4.261680	-1.632948
H	1.337966	2.481939	-1.685108
H	2.820840	4.539499	0.535583
H	3.510942	3.166998	1.418311
H	3.117691	2.993981	-0.299182
H	1.791648	2.931695	3.147423
H	1.114976	4.479197	2.595202
H	0.069005	3.046391	2.752833
O	1.510977	-1.639488	0.794733
C	1.888032	-2.984818	0.989925
Li	2.371419	-0.070209	0.709390
C	3.414005	-3.035853	1.203220
C	1.176559	-3.545035	2.233008
C	1.518157	-3.813901	-0.252829
H	3.774492	-4.058731	1.367464
H	3.930993	-2.630864	0.324029
H	3.692646	-2.433127	2.077824
H	1.442085	-2.946735	3.112124
H	0.093364	-3.475549	2.092577
H	1.440822	-4.592485	2.426212
H	0.441448	-3.740309	-0.430764
H	2.034041	-3.417485	-1.135926
H	1.785979	-4.871861	-0.140379
O	3.552515	0.203461	-0.808869
C	2.861937	-0.354689	-1.963047
C	4.980762	0.022591	-0.944223
C	3.893537	-1.247298	-2.647573
H	2.543734	0.473848	-2.608661
H	1.984933	-0.889276	-1.588490
C	5.203424	-0.492034	-2.370075
H	5.312246	-0.709884	-0.196945
H	5.469271	0.981018	-0.742166
H	3.911063	-2.234406	-2.171730
H	3.688414	-1.382476	-3.713370

H	6.094352	-1.121581	-2.449598
H	5.313991	0.346980	-3.066959

TS_{J-K-1}

Imaginary Freq = -237.4504 (cm^{-1})
Electronic Energy BS1 = -1429.56540867 Hartree
Electronic Energy BS2 = -1429.94913547 Hartree
Zero-point Energy Correction = 0.570060 Hartree
Thermal Correction to Enthalpy = 0.604911 Hartree
Thermal Correction to Free Energy = 0.502524 Hartree

**Chemical symbol**	**X,**	**Y,**	**Z**
Cu	-0.026346	-1.391442	-0.359011
C	-1.699393	-2.048311	0.810496
C	-1.853800	-0.924873	-0.024142
H	-1.248532	-1.966625	1.799696
C	-2.864809	-0.370127	-0.751853
H	-2.600843	0.270218	-1.588197
H	-2.345228	-2.909374	0.653962
C	-4.294380	-0.451025	-0.466351
C	-5.189539	0.228542	-1.323207
C	-4.848051	-1.134910	0.639412
C	-6.563194	0.218855	-1.097000
H	-4.788717	0.767346	-2.179355
C	-6.220795	-1.130641	0.870574
H	-4.186659	-1.660003	1.319450
C	-7.091167	-0.461320	0.003895
H	-7.224563	0.746433	-1.780431
H	-6.617592	-1.661378	1.733131
H	-8.163145	-0.472308	0.182582
B	-0.339082	0.597598	-0.195507
O	-0.744996	1.678591	-0.942846
O	0.356531	1.048652	0.962228
C	-0.250291	2.905140	-0.345756
C	0.043457	2.474342	1.133452
C	-1.329141	3.975107	-0.493981
C	1.011187	3.308542	-1.120318
C	1.233358	3.163717	1.789845
C	-1.191436	2.530472	2.038048
H	-1.042301	4.894331	0.030253
H	-1.464389	4.213346	-1.553625
H	-2.287790	3.628244	-0.102942
H	0.762990	3.370692	-2.183995
H	1.388345	4.283944	-0.793214
H	1.805321	2.567588	-0.994561
H	1.061307	4.243842	1.858809
H	1.371262	2.778397	2.806116
H	2.154298	2.993133	1.227408
H	-0.962010	2.032631	2.985197
H	-1.486576	3.563412	2.249943

H	-2.035163	2.007754	1.577290
O	1.836233	-1.729546	-0.090484
C	2.331282	-3.022190	0.232315
Li	1.979025	-0.050688	0.609870
C	3.867369	-2.956649	0.178172
C	1.865892	-3.418302	1.643243
C	1.815361	-4.040385	-0.797017
H	4.325472	-3.917105	0.442380
H	4.197565	-2.683363	-0.830620
H	4.238842	-2.197309	0.879199
H	2.247094	-2.701794	2.383314
H	0.771672	-3.398566	1.680149
H	2.213261	-4.419785	1.925447
H	0.719694	-4.076557	-0.760061
H	2.114090	-3.733042	-1.805686
H	2.202827	-5.048540	-0.605815
O	3.515401	0.929531	0.009558
C	3.864309	0.493950	-1.341077
C	4.709748	1.293250	0.741547
C	5.377453	0.294001	-1.314669
H	3.567872	1.287529	-2.037584
H	3.288743	-0.412904	-1.542246
C	5.826044	1.360208	-0.302141
H	4.911262	0.518102	1.493272
H	4.522125	2.242432	1.252417
H	5.619531	-0.708874	-0.946295
H	5.830510	0.418313	-2.302304
H	6.811113	1.163593	0.130534
H	5.848536	2.349965	-0.772930

K-1

Electronic Energy BS1 = -1429.65838530 Hartree
Electronic Energy BS2 = -1430.03760701 Hartree
Zero-point Energy Correction = 0.573653 Hartree
Thermal Correction to Enthalpy = 0.608362 Hartree
Thermal Correction to Free Energy = 0.505799 Hartree

Chemical symbol X, Y, Z

Cu	-0.099978	-1.289895	1.482007
C	-0.936574	0.245030	2.315701
C	-0.948809	1.303142	1.272739
H	-0.241875	0.512081	3.126259
C	-1.998779	1.699893	0.482852
H	-1.817596	2.550626	-0.174686
H	-1.922959	0.055007	2.750534
C	-3.331070	1.115805	0.326691
C	-4.340692	1.881121	-0.297819
C	-3.650894	-0.212302	0.685902
C	-5.612319	1.363246	-0.524114

H	-4.112482	2.900713	-0.602211
C	-4.921900	-0.733854	0.444600
H	-2.889452	-0.844817	1.127361
C	-5.913338	0.049291	-0.150590
H	-6.370952	1.982792	-0.996530
H	-5.137808	-1.761996	0.726252
H	-6.905446	-0.358262	-0.326426
B	0.385431	1.957009	0.821012
O	0.560735	3.269765	0.468556
O	1.563958	1.215979	0.583847
C	1.807600	3.391101	-0.266408
C	2.631107	2.175495	0.281378
C	2.414370	4.759091	0.026736
C	1.461241	3.258660	-1.755125
C	3.592374	1.539706	-0.719178
C	3.341773	2.464096	1.606044
H	3.402963	4.851277	-0.437377
H	1.769141	5.541380	-0.384468
H	2.511125	4.932166	1.100665
H	0.701644	4.005523	-2.004216
H	2.338544	3.432705	-2.387465
H	1.057862	2.266813	-1.981762
H	4.349194	2.265336	-1.036026
H	4.118457	0.697560	-0.253009
H	3.069472	1.181668	-1.610329
H	3.700775	1.522146	2.031927
H	4.197156	3.131985	1.462677
H	2.655581	2.922149	2.324512
O	1.123907	-2.197114	0.329680
C	1.656001	-3.476838	0.607416
Li	1.485697	-0.553769	-0.311160
C	2.528599	-3.902792	-0.585992
C	2.508894	-3.414078	1.886809
C	0.504008	-4.480486	0.791412
H	2.982170	-4.888924	-0.430534
H	1.922797	-3.943480	-1.498700
H	3.337614	-3.176707	-0.740239
H	3.321995	-2.689125	1.759211
H	1.887291	-3.076820	2.724906
H	2.945840	-4.387806	2.141653
H	-0.132043	-4.162365	1.626790
H	-0.116295	-4.508267	-0.111615
H	0.870057	-5.493931	0.997267
O	0.792039	-0.294677	-2.111612
C	-0.659815	-0.395493	-2.065985
C	1.285326	-1.253439	-3.084544
C	-0.935260	-1.867710	-2.335171
H	-1.085637	0.260308	-2.837682
H	-0.983236	-0.045111	-1.084388
C	0.110943	-2.199052	-3.416356

H	2.126449	-1.779469	-2.622668
H	1.646530	-0.706731	-3.963059
H	-0.734544	-2.432557	-1.420387
H	-1.963535	-2.050227	-2.659884
H	0.416631	-3.249044	-3.396113
H	-0.282123	-1.982800	-4.415766

L-1

Electronic Energy BS1 = -2137.07526153 Hartree
Electronic Energy BS2 = -2137.66700975 Hartree
Zero-point Energy Correction = 0.807094 Hartree
Thermal Correction to Enthalpy = 0.855774 Hartree
Thermal Correction to Free Energy = 0.725130 Hartree

Chemical symbol X, Y, Z

Cu	-0.848361	0.906414	-0.318178
C	-1.110530	-1.197641	-1.688363
C	-0.555690	0.107824	-2.096808
C	-0.491108	-2.386257	-1.375281
H	-1.233877	0.643401	-2.773256
H	0.453958	0.095128	-2.512041
C	0.912326	-2.772417	-1.376103
H	-1.154598	-3.195531	-1.067615
C	2.001241	-1.873174	-1.453093
C	1.226891	-4.146276	-1.231195
C	3.317841	-2.332853	-1.412915
H	1.817511	-0.807910	-1.506843
C	2.540381	-4.600305	-1.178383
H	0.410215	-4.862803	-1.163909
C	3.601071	-3.693071	-1.274866
H	4.131211	-1.619165	-1.479787
H	2.738617	-5.663814	-1.065698
H	4.630771	-4.038337	-1.237907
N	1.482087	2.997969	-0.532666
C	1.776237	4.237387	0.196266
C	1.074717	3.291959	-1.908168
C	0.503370	5.090346	0.200261
H	2.600698	4.788367	-0.283278
H	2.059343	3.973950	1.219004
C	-0.189753	4.147552	-1.829501
H	0.858085	2.344965	-2.405183
H	1.864151	3.833580	-2.453547
H	-0.277461	4.580096	0.780902
H	0.707972	6.059029	0.665135
O	0.049764	5.356327	-1.119142
H	-0.990910	3.561360	-1.349907
H	-0.515225	4.430024	-2.834352
O	2.294119	1.287061	1.339933
C	2.991860	1.414421	0.356195

O	2.733007	2.270611	-0.669330
C	4.265831	0.674396	0.126443
C	5.082092	0.896364	-0.992261
C	4.634456	-0.283507	1.080250
C	6.253595	0.156766	-1.153018
H	4.795086	1.637973	-1.728838
C	5.802172	-1.023015	0.913741
H	3.987126	-0.439115	1.935828
C	6.613311	-0.804273	-0.203470
H	6.883535	0.326697	-2.021540
H	6.077156	-1.773418	1.649305
H	7.523665	-1.382733	-0.335683
B	-2.636815	-1.155175	-1.383461
O	-3.145448	-1.653163	-0.168562
O	-3.617295	-0.579361	-2.140358
C	-4.607168	-1.551845	-0.234156
C	-4.797213	-0.416137	-1.302980
C	-5.111907	-2.919752	-0.700507
C	-5.137832	-1.221197	1.156152
C	-6.038502	-0.561711	-2.175974
C	-4.708238	0.990599	-0.701711
H	-4.726702	-3.159332	-1.696295
H	-4.759975	-3.687559	-0.004284
H	-6.205652	-2.952746	-0.730226
H	-4.955711	-2.060541	1.837217
H	-4.656279	-0.330094	1.565443
H	-6.218765	-1.046337	1.121025
H	-6.079673	0.264790	-2.891874
H	-6.027638	-1.496999	-2.740170
H	-6.946351	-0.528680	-1.562800
H	-3.811104	1.093253	-0.083790
H	-4.643036	1.716159	-1.518399
H	-5.591112	1.227139	-0.097750
C	-0.400728	-3.489701	2.083648
C	0.759789	-1.421906	2.018354
C	0.971313	-3.731439	2.705419
H	-0.436073	-3.852628	1.050356
H	-1.233083	-3.917891	2.650494
C	1.788058	-2.557994	2.135486
H	0.814909	-0.700314	2.835235
H	0.905023	-3.671470	3.798168
H	1.385395	-4.706707	2.433035
H	2.626108	-2.280431	2.781614
H	2.187337	-2.814791	1.151882
O	-1.776966	0.812899	1.363501
C	-1.856014	1.838734	2.336223
Li	-1.911612	-0.991302	1.286669
C	-2.618999	3.039613	1.747227
C	-0.442268	2.266256	2.758267
C	-2.619427	1.289935	3.554688

H	-2.135657	3.358684	0.817020
H	-3.649730	2.758650	1.505399
H	-2.646310	3.890709	2.439083
H	-0.461588	3.120334	3.448065
H	0.075452	1.436047	3.248982
H	0.146376	2.521943	1.874265
H	-3.631873	0.982553	3.264411
H	-2.095788	0.416349	3.963204
H	-2.707141	2.038459	4.351735
H	0.835148	-0.878200	1.074134
O	-0.557655	-2.056897	2.096200

$TS_{L\text{-}M'\text{-}1}$

Imaginary Freq = -359.7219 (cm^{-1})
Electronic Energy BS1 = -2137.04969097 Hartree
Electronic Energy BS2 = -2137.64029838 Hartree
Zero-point Energy Correction = 0.804225 Hartree
Thermal Correction to Enthalpy = 0.852696 Hartree
Thermal Correction to Free Energy = 0.722685 Hartree

Chemical symbol X, Y, Z

Cu	-0.460070	1.272217	0.066380
C	-0.655331	-1.146307	-1.630151
C	-0.167325	0.203293	-1.777730
C	0.003903	-2.345965	-1.407212
H	-0.840235	0.823560	-2.379391
H	0.874762	0.358331	-2.057876
C	1.422133	-2.650554	-1.338959
H	-0.633389	-3.227335	-1.317433
C	2.441220	-1.684736	-1.167455
C	1.833332	-4.005194	-1.415051
C	3.785138	-2.052017	-1.110315
H	2.173449	-0.648207	-1.027508
C	3.173002	-4.371343	-1.342900
H	1.073877	-4.774283	-1.545541
C	4.164691	-3.393753	-1.198353
H	4.538495	-1.281668	-0.982960
H	3.447688	-5.421880	-1.407371
H	5.213454	-3.674446	-1.149980
N	0.213735	3.056873	-0.336115
C	0.508977	4.078994	0.664833
C	-0.244190	3.684082	-1.568737
C	-0.815605	4.813686	0.956529
H	1.247194	4.798148	0.287842
H	0.889803	3.590265	1.561809
C	-1.566371	4.400380	-1.224446
H	-0.402064	2.916181	-2.326115
H	0.483371	4.420149	-1.932089
H	-1.531108	4.116246	1.413589

H	-0.628028	5.638357	1.650659
O	-1.353665	5.382979	-0.225520
H	-2.309411	3.655712	-0.897248
H	-1.945519	4.920300	-2.109239
O	1.871274	1.325618	1.002897
C	2.470169	1.821254	0.041265
O	1.952427	2.672822	-0.810950
C	3.906056	1.478622	-0.235116
C	4.510831	1.755280	-1.468012
C	4.642292	0.853835	0.780315
C	5.842555	1.401669	-1.683272
H	3.929983	2.233136	-2.249509
C	5.979076	0.521305	0.569211
H	4.147459	0.631436	1.719593
C	6.579679	0.791190	-0.664225
H	6.306096	1.602180	-2.645190
H	6.549043	0.040947	1.359772
H	7.618962	0.521867	-0.832996
B	-2.204920	-1.229113	-1.564166
O	-2.824237	-1.961230	-0.546524
O	-3.106162	-0.544779	-2.330438
C	-4.270476	-1.859637	-0.750016
C	-4.379213	-0.555758	-1.625089
C	-4.688117	-3.130293	-1.495294
C	-4.952064	-1.777690	0.609786
C	-5.508023	-0.567743	-2.649782
C	-4.405820	0.729348	-0.789924
H	-4.191426	-3.193609	-2.468308
H	-4.393652	-4.003331	-0.904476
H	-5.771173	-3.165331	-1.650680
H	-4.803865	-2.714094	1.158662
H	-4.544691	-0.961004	1.207542
H	-6.029870	-1.621738	0.488517
H	-5.495313	0.367512	-3.217869
H	-5.402845	-1.393471	-3.356998
H	-6.480145	-0.651404	-2.150549
H	-3.569152	0.745923	-0.085214
H	-4.300749	1.584435	-1.465985
H	-5.346499	0.838895	-0.239661
C	0.080700	-3.957556	1.827365
C	1.076550	-1.828006	2.209729
C	1.400022	-4.174803	2.566732
H	0.162433	-4.257428	0.777010
H	-0.775035	-4.463182	2.286153
C	2.173152	-2.894683	2.211088
H	0.944360	-1.371427	3.197282
H	1.225702	-4.239686	3.647641
H	1.910281	-5.085996	2.241191
H	2.967081	-2.660881	2.927065
H	2.619786	-2.984507	1.218532

O	-1.593412	0.264412	1.314960
C	-1.928675	0.752438	2.603696
Li	-1.405413	-1.497263	0.866059
C	-3.116941	1.725766	2.497095
C	-0.727967	1.473359	3.240857
C	-2.326694	-0.441269	3.496255
H	-2.890652	2.495394	1.750726
H	-4.021900	1.202970	2.169196
H	-3.333672	2.216843	3.454079
H	-0.932206	1.766900	4.278264
H	0.154956	0.829722	3.214086
H	-0.486132	2.374979	2.671091
H	-3.165589	-0.988223	3.048026
H	-1.487060	-1.135945	3.605323
H	-2.636866	-0.113072	4.495823
H	1.231656	-1.035855	1.474498
O	-0.161086	-2.534896	1.889370

M'-1

Electronic Energy BS1 = -2137.10822482 Hartree
Electronic Energy BS2 = -2137.69827721 Hartree
Zero-point Energy Correction = 0.806606 Hartree
Thermal Correction to Enthalpy = 0.855473 Hartree
Thermal Correction to Free Energy = 0.723406 Hartree

Chemical symbol X, Y, Z

Cu	-0.506296	1.334694	-0.118228
C	-0.565873	-0.823488	-1.682058
C	-0.597415	0.599500	-2.000947
C	0.487006	-1.701578	-1.780907
H	-1.554326	0.928570	-2.397965
H	0.247712	1.060929	-2.503269
C	1.862802	-1.574973	-2.246469
H	0.244010	-2.727464	-1.504428
C	2.557134	-0.359502	-2.419724
C	2.565325	-2.776021	-2.501080
C	3.890663	-0.356793	-2.822417
H	2.084556	0.585437	-2.197898
C	3.894190	-2.769265	-2.911222
H	2.047326	-3.724811	-2.376959
C	4.566135	-1.553858	-3.071305
H	4.407651	0.592358	-2.925836
H	4.407299	-3.708258	-3.101069
H	5.607772	-1.541153	-3.380172
N	-1.629120	2.690341	-0.629567
C	-1.146270	3.596763	-1.661738
C	-3.054153	2.474691	-0.775949
C	-1.885463	4.937296	-1.500771
H	-1.358874	3.221456	-2.679603

H	-0.066781	3.729716	-1.551131
C	-3.769821	3.834370	-0.650967
H	-3.396890	1.796163	0.004340
H	-3.314198	2.041506	-1.759045
H	-1.634510	5.380388	-0.523179
H	-1.591831	5.632719	-2.293359
O	-3.290108	4.754760	-1.617385
H	-3.620085	4.230068	0.366865
H	-4.843641	3.717832	-0.831323
O	1.311078	0.841599	0.505427
C	2.185548	1.672320	0.030212
O	1.971874	2.487408	-0.885116
C	3.564266	1.585788	0.634516
C	4.667784	1.994453	-0.124866
C	3.766740	1.118248	1.938328
C	5.957913	1.894974	0.394122
H	4.489225	2.391833	-1.118662
C	5.055431	1.036766	2.467605
H	2.906383	0.843223	2.536943
C	6.155147	1.412425	1.691425
H	6.809915	2.200646	-0.207924
H	5.201924	0.684143	3.485614
H	7.160215	1.340637	2.099417
B	-1.888031	-1.444814	-1.138284
O	-1.845416	-2.380655	-0.102112
O	-3.158470	-1.166516	-1.539029
C	-3.192052	-2.954148	0.015152
C	-4.082598	-1.814026	-0.606069
C	-3.174553	-4.240328	-0.815706
C	-3.461619	-3.263835	1.482611
C	-5.288636	-2.308477	-1.397279
C	-4.495254	-0.752068	0.415670
H	-2.962388	-4.026744	-1.867988
H	-2.388383	-4.900259	-0.435658
H	-4.130760	-4.768786	-0.753046
H	-2.778568	-4.047095	1.828654
H	-3.322483	-2.383268	2.113195
H	-4.487248	-3.625303	1.614081
H	-5.832362	-1.451127	-1.805005
H	-4.993299	-2.948535	-2.231636
H	-5.970728	-2.867556	-0.746825
H	-3.618705	-0.356086	0.939319
H	-4.981936	0.072095	-0.114026
H	-5.206846	-1.157760	1.142589
C	0.988822	-3.774166	1.330015
C	1.796387	-1.786735	2.319182
C	2.505619	-3.679481	1.054355
H	0.389732	-3.885197	0.422203
H	0.738389	-4.591047	2.016907
C	2.825341	-2.189116	1.273659

H	2.088159	-2.081123	3.336991
H	3.060061	-4.302289	1.764879
H	2.762034	-4.010486	0.044742
H	3.852086	-2.011095	1.603299
H	2.653372	-1.606212	0.365368
O	-1.399540	0.295556	1.540013
C	-1.569878	0.931123	2.791535
Li	-0.777750	-1.378290	1.310961
C	-2.607908	2.063127	2.673393
C	-0.237446	1.531022	3.285659
C	-2.068417	-0.108761	3.816719
H	-2.265446	2.794052	1.934691
H	-3.572787	1.661305	2.342336
H	-2.762151	2.575661	3.631322
H	-0.363461	2.076529	4.229541
H	0.503985	0.740558	3.443400
H	0.167605	2.215761	2.533579
H	-3.034070	-0.520433	3.497864
H	-1.350305	-0.936646	3.902569
H	-2.199133	0.324999	4.816090
H	1.541272	-0.729702	2.281804
O	0.600827	-2.518053	1.939879

L-2

Electronic Energy BS1 = -2369.56047161 Hartree
Electronic Energy BS2 = -2370.22268809 Hartree
Zero-point Energy Correction = 0.926878 Hartree
Thermal Correction to Enthalpy = 0.982275 Hartree
Thermal Correction to Free Energy = 0.833255 Hartree

Chemical symbol X, Y, Z

Cu	-0.092739	0.501275	0.400573
C	-0.456882	-1.123209	-1.599560
C	-0.023110	0.281325	-1.536898
C	0.304452	-2.269229	-1.622343
H	-0.761070	0.953917	-1.981739
H	0.954143	0.501008	-1.962838
C	1.748292	-2.454599	-1.610890
H	-0.248799	-3.206454	-1.658507
C	2.713452	-1.423108	-1.489916
C	2.250132	-3.777946	-1.718704
C	4.079743	-1.704410	-1.533499
H	2.400891	-0.398076	-1.357232
C	3.612609	-4.056235	-1.731803
H	1.538190	-4.596715	-1.807181
C	4.545346	-3.015295	-1.652118
H	4.784426	-0.879363	-1.470309
H	3.950508	-5.086319	-1.818894
H	5.611386	-3.224487	-1.684238

N	-0.800898	3.813411	-0.185430
C	-0.831833	5.233893	0.177251
C	-1.975642	3.448463	-0.979861
C	-2.107178	5.473958	0.989426
H	-0.831644	5.873173	-0.720228
H	0.052918	5.448266	0.781236
C	-3.217698	3.758568	-0.141691
H	-1.928725	2.379624	-1.197325
H	-2.012466	4.015262	-1.924605
H	-2.061531	4.892256	1.923963
H	-2.199535	6.534783	1.239026
O	-3.264815	5.125677	0.242339
H	-3.231863	3.111573	0.749076
H	-4.118797	3.562516	-0.730490
O	1.651263	3.451879	0.773952
C	1.484592	3.305456	-0.419530
O	0.328609	3.603464	-1.075119
C	2.535311	2.816605	-1.358374
C	2.296250	2.597529	-2.722432
C	3.799823	2.541156	-0.820580
C	3.311691	2.091678	-3.533130
H	1.314526	2.801026	-3.133267
C	4.812574	2.037932	-1.634267
H	3.963073	2.718959	0.236823
C	4.568267	1.808461	-2.991749
H	3.118700	1.907935	-4.586060
H	5.789583	1.821050	-1.210680
H	5.354058	1.406261	-3.625355
B	-2.001791	-1.317524	-1.568006
O	-2.654885	-2.491529	-1.226825
O	-2.889170	-0.301077	-1.859609
C	-4.060680	-2.314084	-1.551797
C	-4.216874	-0.750290	-1.495583
C	-4.263081	-2.891201	-2.958133
C	-4.904491	-3.080597	-0.538667
C	-5.221220	-0.165111	-2.484059
C	-4.488622	-0.220047	-0.082460
H	-3.664721	-2.344105	-3.693135
H	-3.933772	-3.934840	-2.962717
H	-5.313933	-2.855223	-3.263647
H	-4.707462	-4.153899	-0.629696
H	-4.676762	-2.777108	0.485308
H	-5.971967	-2.913503	-0.721621
H	-5.247340	0.924385	-2.378531
H	-4.948767	-0.396317	-3.516049
H	-6.228274	-0.551295	-2.289048
H	-3.764790	-0.619344	0.633135
H	-4.379675	0.868750	-0.085907
H	-5.499659	-0.466480	0.258547
C	3.550062	-2.255634	1.958094

C	3.114152	0.018205	2.539821
C	4.852298	-1.456481	1.840746
H	3.214434	-2.618725	0.982132
H	3.618774	-3.096119	2.656580
C	4.341442	-0.021372	1.636324
H	3.382726	0.223092	3.584937
H	5.436061	-1.527373	2.766260
H	5.467560	-1.815499	1.011849
H	5.078886	0.743001	1.900971
H	4.039581	0.130778	0.597285
O	-0.135117	0.209558	2.272029
C	-0.877556	0.981857	3.197030
Li	0.687533	-1.375990	2.260305
C	-2.365257	0.951854	2.805922
C	-0.360928	2.428458	3.196027
C	-0.692996	0.356128	4.591078
H	-2.475980	1.330558	1.784320
H	-2.739182	-0.078525	2.821576
H	-2.985588	1.558802	3.477722
H	-0.924023	3.059647	3.897016
H	0.696661	2.448738	3.480907
H	-0.433167	2.848721	2.189423
H	-1.052675	-0.681591	4.595771
H	0.369915	0.352708	4.861628
H	-1.241789	0.909584	5.362850
H	2.333446	0.713652	2.223302
O	2.558390	-1.324007	2.463545
O	-0.248835	-3.011570	2.102686
C	0.190037	-4.215085	1.418270
C	-1.697150	-2.922926	2.068223
C	-1.080481	-4.853955	0.848032
H	0.677631	-4.860558	2.159205
H	0.914508	-3.934707	0.650081
C	-2.163215	-4.352602	1.815509
H	-1.990266	-2.257070	1.250413
H	-2.024512	-2.499189	3.020950
H	-1.287274	-4.463384	-0.152345
H	-1.008734	-5.944542	0.798045
H	-3.167364	-4.392503	1.387357
H	-2.155744	-4.929786	2.748222

$\mathbf{TS}_{\mathbf{L\text{-}M'\text{-}2}}$

Imaginary Freq = -381.0358 (cm^{-1})
Electronic Energy BS1 = -2369.53328200 Hartree
Electronic Energy BS2 = -2370.19304452 Hartree
Zero-point Energy Correction = 0.924389 Hartree
Thermal Correction to Enthalpy = 0.979287 Hartree
Thermal Correction to Free Energy = 0.833888 Hartree

Chemical symbol X, Y, Z			
Cu	-0.096075	1.499077	0.477277
C	-0.465992	-0.664724	-1.461277
C	0.319277	0.536148	-1.388768
C	-0.064639	-1.995703	-1.419308
H	-0.102917	1.360618	-1.966435
H	1.391142	0.464784	-1.551956
C	1.260934	-2.591004	-1.420315
H	-0.865615	-2.724255	-1.523344
C	2.473739	-1.905109	-1.158629
C	1.370129	-3.979013	-1.701472
C	3.702164	-2.562219	-1.213685
H	2.458511	-0.863111	-0.873139
C	2.596050	-4.634912	-1.733226
H	0.460799	-4.538336	-1.914977
C	3.780984	-3.928546	-1.494856
H	4.608549	-1.990345	-1.030753
H	2.630302	-5.699341	-1.954660
H	4.742976	-4.432579	-1.531375
N	0.232524	3.379159	0.070426
C	0.574558	4.415307	1.047918
C	-0.584402	3.951225	-0.999223
C	-0.744626	4.953935	1.635364
H	1.115154	5.238188	0.563442
H	1.198220	3.967457	1.820495
C	-1.875894	4.478734	-0.349126
H	-0.822459	3.175204	-1.726797
H	-0.058640	4.779234	-1.489782
H	-1.260368	4.157857	2.190792
H	-0.524198	5.778578	2.319826
O	-1.585973	5.472382	0.617787
H	-2.421268	3.636532	0.103048
H	-2.506641	4.944398	-1.112471
O	2.481775	2.080869	1.006548
C	2.626689	2.440699	-0.164054
O	1.812529	3.252369	-0.807924
C	3.761522	1.921506	-0.997739
C	3.804995	2.101495	-2.386346
C	4.767363	1.184018	-0.357613
C	4.843613	1.539827	-3.128221
H	3.014055	2.661912	-2.872454
C	5.808124	0.630968	-1.101184
H	4.708283	1.048247	0.716940
C	5.845207	0.804958	-2.488302
H	4.868246	1.668497	-4.206752
H	6.587344	0.060948	-0.601926
H	6.651308	0.365075	-3.069454
B	-1.997455	-0.416629	-1.610804
O	-2.974326	-1.399385	-1.566160
O	-2.540661	0.835047	-1.835328

C	-4.206546	-0.804092	-2.053775
C	-3.981609	0.717155	-1.733530
C	-4.273647	-1.099687	-3.557550
C	-5.390151	-1.447223	-1.338033
C	-4.622941	1.688004	-2.720321
C	-4.357927	1.086694	-0.294400
H	-3.434824	-0.634151	-4.084052
H	-4.208945	-2.181636	-3.708213
H	-5.209048	-0.740653	-3.999361
H	-5.452232	-2.506753	-1.607178
H	-5.294699	-1.375788	-0.252692
H	-6.327921	-0.965407	-1.636932
H	-4.398272	2.717225	-2.421156
H	-4.241531	1.540527	-3.733050
H	-5.712251	1.567271	-2.731853
H	-3.864333	0.423398	0.420186
H	-4.012485	2.102904	-0.085671
H	-5.440589	1.050887	-0.134016
C	2.008133	-3.275532	1.985680
C	2.269036	-0.952956	2.405371
C	3.406838	-3.039059	2.575482
H	2.059949	-3.667845	0.967183
H	1.393367	-3.943172	2.600214
C	3.662383	-1.555403	2.261529
H	2.038226	-0.735415	3.457357
H	3.403561	-3.202861	3.659686
H	4.150805	-3.705791	2.130129
H	4.387188	-1.090716	2.937445
H	4.015500	-1.437875	1.234357
O	-0.823943	0.242544	1.771520
C	-1.187863	0.655576	3.076478
Li	-0.367664	-1.456204	1.239359
C	-2.574363	1.320496	3.023810
C	-0.157174	1.648946	3.645780
C	-1.237154	-0.577159	4.001475
H	-2.554242	2.142527	2.298377
H	-3.325918	0.597774	2.686978
H	-2.886431	1.717377	3.998464
H	-0.405058	1.948651	4.671926
H	0.841663	1.205434	3.637141
H	-0.116535	2.548152	3.025958
H	-1.966131	-1.305653	3.635349
H	-0.258402	-1.068683	4.029732
H	-1.515811	-0.300354	5.025757
H	2.102425	-0.053168	1.813096
O	1.366417	-1.980552	1.933256
O	-1.604778	-2.963517	1.495421
C	-1.326742	-4.263049	0.930884
C	-3.036385	-2.721012	1.484738
C	-2.545568	-4.586196	0.070915

H	-1.206370	-4.990775	1.746332
H	-0.390318	-4.192218	0.374985
C	-3.680252	-3.993089	0.919598
H	-3.215723	-1.856266	0.843270
H	-3.360133	-2.493069	2.504482
H	-2.483360	-4.055443	-0.884307
H	-2.656561	-5.657957	-0.119954
H	-4.580262	-3.772219	0.340128
H	-3.953810	-4.681476	1.728180

M'-2

Electronic Energy BS1 = -2369.58970595 Hartree
Electronic Energy BS2 = -2370.25414208 Hartree
Zero-point Energy Correction = 0.927031 Hartree
Thermal Correction to Enthalpy = 0.981990 Hartree
Thermal Correction to Free Energy = 0.836066 Hartree

Chemical symbol X, Y, Z

Cu	0.516424	1.493771	0.109246
C	-1.772662	0.287404	-1.187992
C	-0.870315	1.432639	-1.348793
C	-1.589219	-0.968148	-1.703078
H	-1.397574	2.380037	-1.271011
H	-0.231722	1.398528	-2.234338
C	-0.463599	-1.575750	-2.411912
H	-2.448070	-1.631850	-1.614121
C	0.865417	-1.106408	-2.363253
C	-0.721910	-2.743971	-3.168206
C	1.881571	-1.761915	-3.057706
H	1.154186	-0.255625	-1.760545
C	0.292153	-3.395723	-3.863687
H	-1.739476	-3.127251	-3.214464
C	1.601951	-2.903949	-3.812055
H	2.886538	-1.360627	-2.979076
H	0.064219	-4.286561	-4.443296
H	2.396810	-3.412775	-4.351130
N	1.050622	3.125554	-0.569440
C	1.815430	3.187822	-1.802247
C	0.131967	4.247717	-0.509109
C	2.622101	4.498406	-1.801953
H	1.155163	3.200443	-2.691730
H	2.483238	2.326655	-1.867598
C	0.971581	5.542501	-0.505602
H	-0.469410	4.194733	0.405828
H	-0.550616	4.298979	-1.377986
H	3.350769	4.474674	-0.975965
H	3.158307	4.618128	-2.748465
O	1.766214	5.628695	-1.674317
H	1.603574	5.560328	0.397759

H	0.316919	6.420738	-0.502051
O	2.476707	0.808751	0.605454
C	3.446861	0.511745	-0.174916
O	3.397494	0.427300	-1.422228
C	4.760363	0.183009	0.514644
C	5.864122	-0.210836	-0.252322
C	4.877722	0.223537	1.909842
C	7.063136	-0.568138	0.363978
H	5.752703	-0.235146	-1.331466
C	6.076051	-0.134415	2.529377
H	4.018059	0.534462	2.494311
C	7.170866	-0.533968	1.757820
H	7.914114	-0.874524	-0.239725
H	6.157462	-0.101658	3.613287
H	8.104342	-0.814500	2.239762
B	-3.165939	0.573760	-0.554890
O	-4.137073	-0.391048	-0.346714
O	-3.618448	1.840106	-0.282329
C	-5.411898	0.304042	-0.252199
C	-4.977938	1.743209	0.224434
C	-6.018216	0.287677	-1.661106
C	-6.314692	-0.453148	0.716749
C	-5.790669	2.890011	-0.371746
C	-4.912435	1.881469	1.747599
H	-5.374074	0.814622	-2.371828
H	-6.110599	-0.750415	-1.995134
H	-7.010552	0.749318	-1.679665
H	-6.538906	-1.445395	0.311730
H	-5.841259	-0.582082	1.692319
H	-7.262128	0.078653	0.857234
H	-5.408871	3.843345	0.006588
H	-5.720607	2.908595	-1.461333
H	-6.845570	2.806598	-0.086674
H	-4.343193	1.065093	2.196417
H	-4.406161	2.817952	1.998715
H	-5.914762	1.898217	2.188231
C	1.265462	-3.930399	-0.144326
C	2.441350	-2.403162	1.225094
C	2.774536	-4.196460	-0.300359
H	0.788842	-3.701872	-1.099413
H	0.737440	-4.766664	0.329465
C	3.414145	-2.872757	0.151065
H	2.598874	-2.931497	2.177046
H	3.091253	-5.018594	0.351791
H	3.029988	-4.461336	-1.330286
H	4.433920	-2.984836	0.528211
H	3.429151	-2.147541	-0.665983
O	-0.248982	0.227434	1.369522
C	-0.250807	0.632770	2.736524
Li	-0.329661	-1.531427	0.875975

C	-1.676910	1.073477	3.101566
C	0.717808	1.802159	2.999312
C	0.175387	-0.568303	3.602565
H	-1.996742	1.863398	2.414283
H	-2.371253	0.233360	2.997458
H	-1.745554	1.450279	4.130135
H	0.746931	2.045148	4.068746
H	1.723866	1.550322	2.657412
H	0.393420	2.706818	2.467154
H	-0.470294	-1.435152	3.407850
H	1.208140	-0.850173	3.373657
H	0.109698	-0.339168	4.673306
H	2.436873	-1.326721	1.389283
O	1.137945	-2.759249	0.698067
O	-1.854515	-2.718816	1.176660
C	-2.185857	-3.824676	0.310251
C	-3.001726	-2.377917	1.999045
C	-3.709411	-3.810324	0.225386
H	-1.815226	-4.757408	0.757668
H	-1.679667	-3.668241	-0.644862
C	-4.084632	-3.408229	1.659966
H	-3.312582	-1.368068	1.726278
H	-2.693340	-2.398363	3.048707
H	-4.039273	-3.034594	-0.473366
H	-4.124636	-4.774766	-0.081913
H	-5.089517	-2.985614	1.739400
H	-4.022372	-4.274558	2.329055

I

Electronic Energy BS1 = -1314.34124039 Hartree
Electronic Energy BS2 = -1314.68783364 Hartree
Zero-point Energy Correction = 0.550723 Hartree
Thermal Correction to Enthalpy = 0.583812 Hartree
Thermal Correction to Free Energy = 0.484826 Hartree

Chemical symbol X, Y, Z

Cu	-0.674375	-0.020618	-0.975752
B	1.268886	-0.165960	-0.946266
O	1.980206	-1.239249	-0.384192
O	2.167430	0.846356	-1.292340
C	3.344372	-0.819487	-0.131131
C	3.524105	0.354410	-1.152431
C	4.275900	-2.011256	-0.336007
C	3.397945	-0.355805	1.332753
C	4.412153	1.501026	-0.673953
C	3.974988	-0.119336	-2.540760
H	5.325261	-1.710969	-0.230028
H	4.064226	-2.779793	0.415324
H	4.137329	-2.459219	-1.322240

H	3.057797	-1.173407	1.976896
H	4.410800	-0.072178	1.638590
H	2.732288	0.499232	1.488935
H	5.430781	1.149677	-0.470216
H	4.465501	2.273016	-1.448461
H	4.013669	1.964179	0.232186
H	3.873322	0.710966	-3.246275
H	5.019325	-0.451251	-2.539794
H	3.343301	-0.940503	-2.892670
O	-2.521842	0.194881	-0.451859
C	-3.594833	0.243310	-1.368549
Li	-2.050266	0.248124	1.277806
C	-4.907594	0.355720	-0.573720
C	-3.432812	1.469541	-2.285769
C	-3.603832	-1.041964	-2.217141
H	-5.784018	0.404064	-1.231366
H	-5.024584	-0.512285	0.087497
H	-4.893489	1.260842	0.046305
H	-3.433364	2.388848	-1.688370
H	-2.472687	1.408726	-2.812467
H	-4.237408	1.538968	-3.028550
H	-2.654587	-1.130699	-2.759766
H	-3.702103	-1.920533	-1.569129
H	-4.425673	-1.051247	-2.944001
O	-1.608174	-1.455845	2.073821
C	-2.310922	-2.536968	1.406691
C	-0.186197	-1.779459	2.150547
C	-1.242859	-3.256839	0.591464
H	-3.093752	-2.083128	0.794452
H	-2.760704	-3.187907	2.168616
C	-0.013494	-3.159988	1.509562
H	0.117004	-1.738213	3.201877
H	0.358651	-1.029705	1.571552
H	-1.522067	-4.286446	0.347358
H	-1.058925	-2.705491	-0.337304
H	-0.030643	-3.952694	2.266807
H	0.925293	-3.208776	0.953365
O	-1.201208	1.851882	1.919817
C	-1.182574	2.914435	0.924539
C	0.031508	1.881039	2.686181
C	0.296101	3.202369	0.697643
H	-1.717220	3.783494	1.331556
H	-1.704628	2.529387	0.046269
C	0.869960	3.034315	2.114487
H	0.529927	0.915962	2.555311
H	-0.218605	2.009417	3.744439
H	0.721577	2.458405	0.016492
H	0.468887	4.200255	0.283056
H	1.939138	2.801134	2.116402

H	0.720847	3.946653	2.703648

J

Electronic Energy BS1 = -1662.07595134 Hartree
Electronic Energy BS2 = -1662.52292678 Hartree
Zero-point Energy Correction = 0.690281 Hartree
Thermal Correction to Enthalpy = 0.732393 Hartree
Thermal Correction to Free Energy = 0.611978 Hartree

Chemical symbol X, Y, Z

Cu	-0.268398	1.032092	0.560241
C	-1.976227	2.170413	1.041272
C	-2.207708	0.887422	0.598779
H	-1.936853	2.405889	2.105131
C	-3.014439	-0.082749	0.170668
H	-2.561082	-0.986491	-0.228668
H	-2.096358	3.019312	0.368218
C	-4.486550	-0.045011	0.170222
C	-5.191213	-1.158030	-0.322704
C	-5.227414	1.055393	0.640381
C	-6.585244	-1.174319	-0.348156
H	-4.632796	-2.016847	-0.689617
C	-6.619381	1.038308	0.615776
H	-4.698432	1.922041	1.025394
C	-7.307056	-0.075238	0.121520
H	-7.107763	-2.045874	-0.734549
H	-7.173235	1.898315	0.984233
H	-8.393643	-0.084587	0.103662
B	0.136268	-0.943750	0.273134
O	-0.216049	-1.751629	-0.813911
O	0.608802	-1.752935	1.310589
C	0.211890	-3.116875	-0.557015
C	0.306383	-3.140827	1.005408
C	-0.813536	-4.070806	-1.162374
C	1.573918	-3.289505	-1.244166
C	1.413764	-4.022942	1.575979
C	-1.029592	-3.463303	1.688754
H	-0.566754	-5.112534	-0.925127
H	-0.821431	-3.961251	-2.251950
H	-1.820614	-3.858540	-0.796121
H	1.463968	-3.074294	-2.312273
H	1.959776	-4.309310	-1.137661
H	2.300275	-2.584931	-0.829339
H	1.255259	-5.073546	1.305292
H	1.417266	-3.948720	2.668317
H	2.398237	-3.716191	1.214881
H	-0.934085	-3.262414	2.760164
H	-1.314012	-4.512725	1.553719

H	-1.830069	-2.826076	1.301191
O	1.390925	2.070527	0.460734
C	1.751581	3.163582	1.269240
Li	2.017995	0.750101	-0.556713
C	3.248598	3.450714	1.042727
C	1.511551	2.814752	2.749466
C	0.923141	4.400654	0.878002
H	3.601265	4.300302	1.640535
H	3.432600	3.675891	-0.015315
H	3.843566	2.569032	1.311191
H	2.084288	1.918700	3.015133
H	0.449479	2.592024	2.905697
H	1.800221	3.631934	3.422786
H	-0.140849	4.184112	1.009372
H	1.091239	4.645893	-0.177419
H	1.180674	5.278590	1.483617
O	1.739167	1.074668	-2.470131
C	1.422146	2.458961	-2.725744
C	0.736188	0.307959	-3.173895
C	-0.110591	2.538585	-2.666791
H	1.918599	3.050559	-1.955508
H	1.810974	2.731037	-3.717912
C	-0.575498	1.092682	-2.998583
H	1.032201	0.235184	-4.230437
H	0.705176	-0.683982	-2.723830
H	-0.503974	3.282788	-3.366010
H	-0.420069	2.815852	-1.655783
H	-1.191921	1.045919	-3.901583
H	-1.150538	0.675486	-2.167729
O	3.483218	-0.404693	-0.165074
C	3.757591	-0.736902	1.222285
C	4.607652	-0.753457	-1.004031
C	4.919573	-1.724436	1.156919
H	4.037438	0.181783	1.753668
H	2.834371	-1.134918	1.645857
C	5.719104	-1.192735	-0.044200
H	4.296694	-1.568732	-1.668840
H	4.871704	0.115549	-1.614496
H	4.548942	-2.736167	0.952371
H	5.498569	-1.752901	2.084511
H	6.383846	-1.937549	-0.491479
H	6.325757	-0.331062	0.258110

$\mathbf{TS_{J\text{-}K}}$

Imaginary Freq = -179.5983 (cm^{-1})
Electronic Energy BS1 = -1662.06094005 Hartree
Electronic Energy BS2 = -1662.51213393 Hartree
Zero-point Energy Correction = 0.689293 Hartree
Thermal Correction to Enthalpy = 0.730945 Hartree

Thermal Correction to Free Energy = 0.609656 Hartree

Chemical symbol X, Y, Z

Cu	0.184333	1.404661	0.429231
C	-1.564346	2.047904	1.610447
C	-1.695922	1.089593	0.605760
H	-1.122164	1.810316	2.577458
C	-2.645409	0.691835	-0.289931
H	-2.313670	0.191392	-1.194801
H	-2.136751	2.970681	1.550943
C	-4.091743	0.725149	-0.099215
C	-4.917297	0.164865	-1.100668
C	-4.728761	1.239747	1.052316
C	-6.302627	0.128351	-0.966773
H	-4.451896	-0.244709	-1.995398
C	-6.113173	1.189569	1.188434
H	-4.121594	1.668128	1.842384
C	-6.913785	0.640856	0.180947
H	-6.908160	-0.305027	-1.759607
H	-6.574391	1.588898	2.088996
H	-7.994940	0.615326	0.288201
B	-0.326253	-0.556567	0.406198
O	-0.533545	-1.399584	-0.682261
O	-0.295762	-1.280893	1.593418
C	-0.615499	-2.770760	-0.207012
C	-0.879481	-2.588378	1.332695
C	-1.735206	-3.472535	-0.971846
C	0.737357	-3.424561	-0.516014
C	-0.191919	-3.610497	2.233325
C	-2.368968	-2.483088	1.685631
H	-1.891468	-4.489992	-0.594627
H	-1.470720	-3.539423	-2.032719
H	-2.673553	-2.919548	-0.892288
H	0.905911	-3.398031	-1.597353
H	0.770967	-4.470000	-0.191701
H	1.546432	-2.869543	-0.032845
H	-0.553690	-4.623583	2.022505
H	-0.411810	-3.380381	3.280557
H	0.893542	-3.593588	2.106190
H	-2.460050	-2.189030	2.735631
H	-2.886424	-3.438223	1.545476
H	-2.863628	-1.717086	1.082203
O	1.958382	2.015874	0.228632
C	2.402369	3.268167	0.723068
Li	2.423437	0.563913	-0.685209
C	3.887075	3.409864	0.349780
C	2.234249	3.299701	2.251004
C	1.581007	4.395196	0.075864
H	4.307495	4.359925	0.700748
H	4.007351	3.361675	-0.739628
H	4.465787	2.591778	0.797246

H	2.805991	2.482056	2.706030
H	1.176916	3.157516	2.502715
H	2.574906	4.248916	2.682599
H	0.520299	4.256565	0.317087
H	1.691468	4.357096	-1.014288
H	1.894610	5.386127	0.426473
O	2.143792	0.418109	-2.572353
C	1.128831	1.331366	-3.086318
C	1.988532	-0.885051	-3.184290
C	0.425623	0.576515	-4.216327
H	0.442251	1.565869	-2.265483
H	1.630726	2.248929	-3.405106
C	0.581232	-0.888168	-3.775729
H	2.761048	-1.004044	-3.956277
H	2.138709	-1.636673	-2.405279
H	0.932446	0.749038	-5.173398
H	-0.619109	0.883118	-4.321149
H	0.474626	-1.601167	-4.599117
H	-0.139377	-1.129386	-2.989446
O	3.253829	-0.909146	0.204152
C	3.109567	-0.902387	1.656805
C	4.312890	-1.806208	-0.194887
C	3.888945	-2.127204	2.128538
H	3.535777	0.032557	2.038110
H	2.042289	-0.929759	1.880868
C	5.028406	-2.201894	1.099497
H	3.861625	-2.676376	-0.689170
H	4.957243	-1.288152	-0.912649
H	3.261099	-3.024516	2.069732
H	4.241647	-2.024678	3.158675
H	5.494446	-3.189272	1.032282
H	5.807821	-1.470361	1.342826

K

Electronic Energy BS1 = -1662.14818219 Hartree
Electronic Energy BS2 = -1662.59852280 Hartree
Zero-point Energy Correction = 0.694323 Hartree
Thermal Correction to Enthalpy = 0.735110 Hartree
Thermal Correction to Free Energy = 0.620164 Hartree

Chemical symbol X, Y, Z

Cu	0.865180	-1.112687	1.383719
C	0.683385	0.585688	2.294114
C	0.387838	1.579237	1.235707
H	-0.161252	0.490464	2.987751
C	1.273383	2.334190	0.507234
H	0.834546	3.052846	-0.184012
H	1.591752	0.806515	2.867954
C	2.732643	2.331465	0.478162

C	3.390973	3.382973	-0.204425
C	3.547102	1.305692	1.011769
C	4.774909	3.417249	-0.340793
H	2.789381	4.183286	-0.631667
C	4.933926	1.335997	0.861895
H	3.089395	0.466005	1.517966
C	5.560127	2.389623	0.193160
H	5.244504	4.245363	-0.866561
H	5.529557	0.525534	1.276092
H	6.641624	2.411741	0.088247
B	-1.109476	1.812353	0.883834
O	-1.549276	2.642148	-0.135499
O	-2.169961	1.222803	1.551732
C	-2.980552	2.468361	-0.269098
C	-3.377558	1.917140	1.147568
C	-3.603946	3.810348	-0.641358
C	-3.203284	1.445395	-1.391230
C	-4.545621	0.935713	1.155090
C	-3.614849	3.026359	2.180827
H	-4.697189	3.736023	-0.669853
H	-3.255685	4.115338	-1.633494
H	-3.322860	4.591438	0.068132
H	-2.726092	1.815223	-2.303996
H	-4.269096	1.296512	-1.596328
H	-2.750922	0.483062	-1.132792
H	-5.464867	1.428273	0.818189
H	-4.708349	0.564832	2.172187
H	-4.355246	0.077912	0.507806
H	-3.673074	2.574994	3.175790
H	-4.546210	3.568591	1.986721
H	-2.786056	3.740981	2.182099
O	0.861108	-2.648954	0.259314
C	1.838171	-3.666827	0.376094
Li	-0.061751	-1.898722	-1.075923
C	1.509925	-4.755828	-0.660722
C	1.787054	-4.264105	1.793182
C	3.235910	-3.083985	0.100701
H	2.221203	-5.589551	-0.620246
H	1.539392	-4.334230	-1.674382
H	0.503008	-5.151679	-0.479779
H	0.782288	-4.651542	1.997178
H	2.001903	-3.481446	2.531003
H	2.512056	-5.077285	1.923256
H	3.447279	-2.278704	0.814193
H	3.272100	-2.654996	-0.907292
H	4.022782	-3.844072	0.184648
O	0.618956	-0.795270	-2.447310
C	1.853957	-0.087145	-2.094503
C	-0.217866	0.059109	-3.261755
C	1.801788	1.236048	-2.858390

H	1.854057	0.065804	-1.011600
H	2.697973	-0.725061	-2.368679
C	0.290188	1.474656	-3.004303
H	-0.100643	-0.231429	-4.314985
H	-1.255666	-0.103998	-2.958874
H	2.269448	1.132411	-3.845110
H	2.315529	2.029706	-2.312157
H	0.037117	2.162564	-3.817210
H	-0.130171	1.862977	-2.071446
O	-1.968900	-1.838935	-0.827944
C	-2.321635	-2.127754	0.570884
C	-2.950728	-2.421313	-1.708860
C	-3.650766	-2.885831	0.510107
H	-1.506690	-2.731100	0.979412
H	-2.363384	-1.175533	1.103523
C	-3.615028	-3.524024	-0.888361
H	-3.672711	-1.646410	-2.002645
H	-2.435475	-2.778575	-2.606482
H	-4.499843	-2.197110	0.589609
H	-3.737505	-3.621069	1.315108
H	-4.603383	-3.796487	-1.270279
H	-2.984200	-4.420714	-0.884751

L

Electronic Energy BS1 = -2137.08553120 Hartree
Electronic Energy BS2 = -2137.67791120 Hartree
Zero-point Energy Correction = 0.806847 Hartree
Thermal Correction to Enthalpy = 0.855784 Hartree
Thermal Correction to Free Energy = 0.721689 Hartree

Chemical symbol X, Y, Z

Cu	-1.005198	1.350164	-0.848743
B	-3.478080	-0.975423	-1.248977
O	-4.069224	-2.112656	-0.729725
O	-4.265579	0.144936	-1.068251
C	-5.553458	-0.293548	-0.568602
C	-5.202181	-1.688557	0.067702
C	-4.704708	-1.580203	1.516101
C	-6.299526	-2.743692	-0.036494
C	-6.079348	0.750862	0.412356
C	-6.485190	-0.403697	-1.781976
H	-6.282023	1.685630	-0.120200
H	-5.352045	0.964323	1.198492
H	-7.012737	0.411229	0.876254
H	-6.114575	-1.150729	-2.490824
H	-6.514096	0.563376	-2.293073
H	-7.504660	-0.673968	-1.486762
H	-3.920520	-0.822425	1.604898
H	-4.279860	-2.543429	1.815275

H	-5.515697	-1.326309	2.206758
H	-5.957407	-3.677672	0.421111
H	-6.554307	-2.952974	-1.077606
H	-7.203826	-2.415731	0.489031
C	-2.045843	-0.919002	-1.860402
C	-1.623197	0.383407	-2.415429
C	-1.297076	-2.055219	-1.667102
H	-2.479623	0.951425	-2.799560
H	-0.858666	0.320722	-3.198020
C	0.136312	-2.282168	-1.782706
H	-1.830369	-2.907729	-1.247871
C	1.097527	-1.280318	-2.062565
C	0.637685	-3.573622	-1.482081
C	2.464677	-1.561742	-2.037213
H	0.765306	-0.275096	-2.278396
C	2.000535	-3.849860	-1.457004
H	-0.073242	-4.365785	-1.254858
C	2.931393	-2.841810	-1.733356
H	3.174529	-0.767770	-2.253032
H	2.341481	-4.854855	-1.218482
H	3.997932	-3.047013	-1.705568
N	2.655843	2.075378	-0.527714
C	3.116421	3.277581	0.193290
C	2.467700	2.373429	-1.958216
C	2.065986	4.374292	-0.007354
H	4.089838	3.607563	-0.197914
H	3.215973	3.024626	1.252924
C	1.476166	3.534399	-2.095749
H	2.073258	1.479244	-2.445456
H	3.428825	2.648140	-2.417257
H	1.109695	4.034882	0.409143
H	2.390263	5.290261	0.494682
O	1.930173	4.680313	-1.388860
H	0.486237	3.221968	-1.736260
H	1.397162	3.815377	-3.149545
O	2.888540	0.323495	1.401631
C	3.726248	0.258703	0.509610
O	3.778127	1.129449	-0.516338
C	4.778081	-0.780495	0.430782
C	5.774287	-0.758720	-0.556776
C	4.721059	-1.838200	1.349113
C	6.702724	-1.795174	-0.624092
H	5.811229	0.059402	-1.267121
C	5.649951	-2.872227	1.275509
H	3.934419	-1.842719	2.095720
C	6.639866	-2.852788	0.288464
H	7.472774	-1.780968	-1.389792
H	5.599244	-3.696692	1.980600
H	7.361819	-3.662604	0.228816
O	-0.333528	2.134940	0.751197

C	-1.246749	2.713313	1.669635
C	-1.914413	3.939883	1.021325
C	-0.455662	3.142966	2.919022
C	-2.329603	1.693460	2.075545
H	-1.155570	4.670557	0.719706
H	-2.456297	3.626642	0.120723
H	-2.621166	4.430837	1.702380
H	-1.102121	3.610484	3.671647
H	0.025408	2.269647	3.378384
H	0.326879	3.860874	2.646950
H	-2.881133	1.363939	1.186793
H	-1.857401	0.818394	2.532425
H	-3.044940	2.120774	2.790378
Li	1.022645	0.918681	1.000467
O	0.303072	-0.597343	2.044230
C	-0.689872	-1.532540	1.558067
C	0.703364	-0.943082	3.384757
C	-1.257285	-2.184514	2.814138
H	-0.198439	-2.263442	0.908391
H	-1.410120	-0.971071	0.961626
C	-0.008596	-2.263104	3.707994
H	0.381204	-0.136525	4.057397
H	1.794943	-1.010188	3.416379
H	-2.018888	-1.541107	3.270285
H	-1.709532	-3.159169	2.609461
H	-0.236956	-2.363610	4.773326
H	0.613809	-3.115225	3.409845

$TS_{L\text{-}M'}$

Imaginary Freq = -274.6503 (cm^{-1})
Electronic Energy BS1 = -2137.06275292 Hartree
Electronic Energy BS2 = -2137.65551605 Hartree
Zero-point Energy Correction = 0.804700 Hartree
Thermal Correction to Enthalpy = 0.853439 Hartree
Thermal Correction to Free Energy = 0.719941 Hartree

Chemical symbol X, Y, Z

Cu	-0.512850	1.281478	-0.301145
B	-3.137202	-0.665277	-1.287005
O	-3.779073	-1.815132	-0.866782
O	-3.941952	0.450158	-1.174454
C	-5.287678	-0.006889	-0.882197
C	-5.024957	-1.419158	-0.240360
C	-4.758040	-1.353033	1.268985
C	-6.083776	-2.475500	-0.543479
C	-5.964618	1.006202	0.036644
C	-6.026581	-0.084444	-2.224289
H	-6.084765	1.957494	-0.491480
H	-5.373217	1.194688	0.934632

H	-6.958091	0.652082	0.335345
H	-5.548810	-0.808231	-2.891999
H	-5.987096	0.897399	-2.705539
H	-7.076344	-0.367093	-2.092679
H	-3.998338	-0.600071	1.497895
H	-4.384210	-2.325458	1.603717
H	-5.666186	-1.115433	1.832627
H	-5.807585	-3.421413	-0.066308
H	-6.174082	-2.654150	-1.617012
H	-7.060851	-2.167858	-0.153593
C	-1.661778	-0.619698	-1.784224
C	-1.095943	0.709711	-2.080855
C	-1.024722	-1.836237	-1.845366
H	-1.871660	1.416525	-2.397840
H	-0.278425	0.697384	-2.808362
C	0.374980	-2.160404	-2.101824
H	-1.642856	-2.710255	-1.641365
C	1.443578	-1.249056	-1.937499
C	0.711310	-3.489407	-2.449989
C	2.766036	-1.643141	-2.137161
H	1.243603	-0.238962	-1.607156
C	2.031450	-3.879816	-2.654632
H	-0.089189	-4.217550	-2.565302
C	3.071041	-2.955959	-2.503518
H	3.559919	-0.917082	-1.994671
H	2.252196	-4.908554	-2.929795
H	4.103866	-3.257115	-2.657361
N	1.655645	2.144264	-0.665857
C	1.790511	3.292580	0.244230
C	1.794360	2.606065	-2.053295
C	0.749147	4.372079	-0.118685
H	2.788294	3.733529	0.141680
H	1.621906	2.938698	1.261291
C	0.748929	3.702904	-2.351550
H	1.657812	1.753246	-2.720602
H	2.789725	3.036932	-2.207589
H	-0.263005	3.983055	0.060510
H	0.908722	5.249748	0.515594
O	0.901155	4.794473	-1.460798
H	-0.261309	3.278265	-2.284870
H	0.914014	4.089815	-3.361666
O	2.756708	0.480478	1.418254
C	3.535348	0.638416	0.462432
O	3.336216	1.412349	-0.558031
C	4.845026	-0.097625	0.428041
C	5.790305	0.136629	-0.580490
C	5.107371	-1.051340	1.420030
C	6.984675	-0.581714	-0.597149
H	5.577891	0.876864	-1.344223
C	6.301676	-1.769417	1.399339

H	4.363874	-1.220312	2.191528
C	7.241215	-1.536240	0.391235
H	7.714779	-0.398459	-1.380615
H	6.499615	-2.512636	2.166891
H	8.171664	-2.097646	0.375371
O	-0.340367	1.449602	1.605849
C	-1.371862	1.919823	2.454022
C	-2.585515	2.389784	1.633503
C	-0.814980	3.095407	3.277546
C	-1.807553	0.782412	3.397842
H	-2.298936	3.211213	0.966377
H	-2.963570	1.578552	1.002894
H	-3.399245	2.742469	2.280556
H	-1.555026	3.493063	3.983233
H	0.066002	2.771927	3.845571
H	-0.506343	3.906458	2.608332
H	-2.160256	-0.071592	2.810518
H	-0.959841	0.445886	4.007641
H	-2.611319	1.095511	4.075516
Li	1.003213	0.258690	1.907214
O	0.499428	-1.546186	2.345195
C	-0.447596	-2.093467	1.384610
C	0.496246	-2.330618	3.554488
C	-1.328032	-3.040879	2.192169
H	0.114176	-2.615312	0.604100
H	-0.983920	-1.259312	0.928625
C	-0.335921	-3.577757	3.236311
H	0.036714	-1.737413	4.356663
H	1.532704	-2.553043	3.827812
H	-2.136817	-2.487361	2.682474
H	-1.774764	-3.819384	1.567785
H	-0.818125	-3.993532	4.126023
H	0.297397	-4.355173	2.793349

M'

Electronic Energy BS1 = -2137.10964591 Hartree
Electronic Energy BS2 = -2137.69812922 Hartree
Zero-point Energy Correction = 0.806682 Hartree
Thermal Correction to Enthalpy = 0.855454 Hartree
Thermal Correction to Free Energy = 0.722514 Hartree

Chemical symbol X, Y, Z

Cu	-0.310942	1.044498	-0.247620
B	-2.923883	-1.072794	-0.813187
O	-3.709889	-2.080002	-0.315875
O	-3.624977	0.077200	-1.089335
C	-5.043303	-0.244234	-1.001163
C	-5.046377	-1.540852	-0.098186
C	-5.171061	-1.245070	1.399458

C	-6.061183	-2.605469	-0.507530
C	-5.777311	0.956156	-0.411259
C	-5.512075	-0.502011	-2.437996
H	-5.693209	1.807911	-1.093589
H	-5.356869	1.253681	0.550946
H	-6.840793	0.729026	-0.276770
H	-4.972620	-1.344258	-2.882651
H	-5.307172	0.387171	-3.041809
H	-6.585587	-0.711838	-2.480498
H	-4.449465	-0.491021	1.717906
H	-4.967318	-2.162743	1.959485
H	-6.177951	-0.898031	1.653280
H	-5.976079	-3.467786	0.161061
H	-5.892076	-2.952750	-1.528933
H	-7.082066	-2.214536	-0.432653
C	-1.397661	-1.218404	-1.114207
C	-0.775261	-0.101839	-1.803312
C	-0.817066	-2.434055	-0.813759
H	-1.466650	0.500967	-2.383357
H	0.184889	-0.238185	-2.294970
C	0.507554	-2.974645	-1.085684
H	-1.496754	-3.161619	-0.369763
C	1.664425	-2.195562	-1.305763
C	0.638124	-4.382579	-1.127132
C	2.883814	-2.802977	-1.596584
H	1.640925	-1.124943	-1.155659
C	1.855916	-4.984983	-1.427834
H	-0.236457	-5.000649	-0.937454
C	2.984864	-4.195277	-1.672448
H	3.759885	-2.177310	-1.745780
H	1.928285	-6.068513	-1.466259
H	3.938801	-4.663441	-1.899790
N	0.070728	2.468955	-1.499139
C	0.714372	3.484179	-0.647377
C	-1.095239	3.095871	-2.125816
C	-0.230760	4.204275	0.323347
H	1.141345	4.232931	-1.336610
H	1.548161	3.040947	-0.098560
C	-2.046678	3.772593	-1.134108
H	-1.642624	2.362692	-2.725628
H	-0.714597	3.865552	-2.818002
H	-0.538044	3.500692	1.110419
H	0.276848	5.050748	0.797025
O	-1.369342	4.744052	-0.345253
H	-2.502879	2.998923	-0.497797
H	-2.847176	4.306927	-1.657682
O	3.137056	1.603194	1.609269
C	3.036182	1.080835	0.463067
O	1.977782	0.471425	0.082871
C	4.197760	1.126155	-0.486932

C	4.023472	0.765905	-1.830363
C	5.465685	1.505346	-0.029796
C	5.109876	0.776436	-2.705172
H	3.030378	0.497086	-2.174124
C	6.553490	1.506677	-0.902876
H	5.577072	1.793602	1.010435
C	6.377531	1.140755	-2.240669
H	4.968957	0.504666	-3.748155
H	7.537863	1.794699	-0.542965
H	7.225177	1.144899	-2.921315
O	-0.361792	0.977342	1.652679
C	-1.445403	0.972399	2.569472
C	-2.664915	1.695411	1.977804
C	-0.989404	1.685559	3.854002
C	-1.805178	-0.491951	2.879470
H	-2.443096	2.752013	1.800100
H	-2.939033	1.242268	1.020004
H	-3.528119	1.639630	2.652963
H	-1.786656	1.711120	4.606956
H	-0.126231	1.168318	4.291365
H	-0.694180	2.715754	3.625363
H	-2.035532	-1.029478	1.953315
H	-0.958870	-0.991381	3.359880
H	-2.670926	-0.563504	3.549182
Li	1.442850	0.658823	2.027072
O	1.973532	-0.973700	2.978194
C	1.379940	-2.161737	2.384071
C	3.401309	-1.172981	3.111348
C	2.542811	-3.104182	2.034777
H	0.823095	-1.839807	1.500255
H	0.679526	-2.601390	3.101754
C	3.751577	-2.155664	1.998271
H	3.615277	-1.594656	4.103982
H	3.872482	-0.193683	3.014657
H	2.676302	-3.855494	2.822297
H	2.377402	-3.625340	1.090106
H	4.706146	-2.664228	2.164555
H	3.795390	-1.631831	1.037998

M

Electronic Energy BS1 = -2137.12697578 Hartree
Electronic Energy BS2 = -2137.71826259 Hartree
Zero-point Energy Correction = 0.806519 Hartree
Thermal Correction to Enthalpy = 0.855695 Hartree
Thermal Correction to Free Energy = 0.721313 Hartree

Chemical symbol X, Y, Z

Cu	0.115101	-0.802660	-0.095189
B	-3.363773	-0.472218	0.721486

O	-4.547954	0.208870	0.584132
O	-3.391391	-1.724373	0.150181
C	-4.769124	-2.003789	-0.225969
C	-5.396345	-0.558253	-0.316968
C	-5.260420	0.079728	-1.704333
C	-6.838313	-0.451049	0.171388
C	-4.761509	-2.787492	-1.534989
C	-5.361739	-2.853800	0.903706
H	-4.293313	-3.764031	-1.375049
H	-4.197340	-2.265689	-2.310852
H	-5.783767	-2.953041	-1.893141
H	-5.353217	-2.306809	1.851684
H	-4.751659	-3.753788	1.026865
H	-6.390195	-3.157514	0.683461
H	-4.233002	0.020861	-2.071345
H	-5.530378	1.137703	-1.634181
H	-5.922044	-0.401732	-2.431796
H	-7.178095	0.585774	0.085163
H	-6.932229	-0.750574	1.217275
H	-7.499540	-1.079271	-0.436046
C	-2.086622	0.096161	1.402837
C	-0.991448	-0.850079	1.589053
C	-2.090949	1.425072	1.739904
H	-1.317902	-1.884637	1.661603
H	-0.225333	-0.612464	2.328512
C	-1.049555	2.305286	2.251631
H	-3.046193	1.929157	1.593101
C	0.331855	2.014636	2.229905
C	-1.447073	3.564563	2.758287
C	1.263351	2.930687	2.711829
H	0.705414	1.102860	1.790575
C	-0.516280	4.473577	3.251514
H	-2.504737	3.818207	2.768134
C	0.846688	4.157711	3.234730
H	2.318247	2.681231	2.649799
H	-0.850759	5.430136	3.644362
H	1.576770	4.868793	3.612328
N	0.677224	-2.493989	0.186997
C	1.522788	-2.789533	1.330135
C	-0.208766	-3.610495	-0.090585
C	2.367602	-4.031590	0.996457
H	0.919964	-3.014576	2.230206
H	2.163361	-1.930290	1.529569
C	0.664885	-4.844537	-0.399551
H	-0.850283	-3.377570	-0.946751
H	-0.854255	-3.856200	0.772030
H	3.038310	-3.800397	0.155604
H	2.972960	-4.327938	1.857846
O	1.526363	-5.137848	0.685585
H	1.245902	-4.653276	-1.316799

H	0.035857	-5.727693	-0.552950
O	3.291921	0.765571	-1.386833
C	3.341250	0.108956	-0.307793
O	2.528101	0.306552	0.660602
C	4.371739	-0.978253	-0.154394
C	4.753531	-1.422940	1.118093
C	4.937735	-1.571589	-1.289337
C	5.682648	-2.454422	1.255150
H	4.311694	-0.949299	1.989107
C	5.851468	-2.617675	-1.153702
H	4.642562	-1.204314	-2.266887
C	6.225969	-3.060504	0.118363
H	5.980299	-2.789441	2.245648
H	6.274874	-3.086180	-2.038555
H	6.940572	-3.872888	0.223644
O	0.003844	0.831488	-0.986955
C	-0.790346	0.998634	-2.152483
C	-1.375687	-0.350640	-2.610703
C	0.116046	1.560569	-3.262524
C	-1.926449	1.988894	-1.843206
H	-0.563976	-1.054702	-2.833921
H	-2.004169	-0.787968	-1.826301
H	-1.986801	-0.239173	-3.515451
H	-0.438340	1.722860	-4.195301
H	0.542692	2.519045	-2.947483
H	0.944854	0.870982	-3.455207
H	-2.568057	1.602506	-1.047369
H	-1.505630	2.939576	-1.498479
H	-2.546174	2.184754	-2.727357
Li	1.680019	1.597890	-0.621283
O	1.899631	3.510782	-0.537417
C	0.879296	4.499232	-0.339307
C	2.911873	4.130903	-1.347144
C	0.758185	5.222989	-1.693303
H	1.195962	5.183322	0.459615
H	-0.021232	3.978405	-0.010098
C	2.133644	4.971461	-2.377273
H	3.518990	3.327521	-1.768012
H	3.540663	4.765616	-0.705413
H	-0.052761	4.792401	-2.288080
H	0.543096	6.287541	-1.560196
H	2.005650	4.411497	-3.308245
H	2.659293	5.900338	-2.617814

$\mathbf{TS_{M\text{-}N}}$

Imaginary Freq = -216.3256 (cm^{-1})
Electronic Energy BS1 = -2137.11798599 Hartree
Electronic Energy BS2 = -2137.70866678 Hartree
Zero-point Energy Correction = 0.806420 Hartree

Thermal Correction to Enthalpy = 0.855079 Hartree
Thermal Correction to Free Energy = 0.722180 Hartree

Chemical symbol	X,	Y,	Z
Cu	-0.021517	-0.712509	-0.214201
B	-3.322410	-0.341194	0.726265
O	-4.436892	0.451617	0.616662
O	-3.468947	-1.565730	0.115246
C	-4.869375	-1.705807	-0.258384
C	-5.366002	-0.207192	-0.290737
C	-5.203703	0.462989	-1.659473
C	-6.782909	0.010562	0.233886
C	-4.938984	-2.437253	-1.595454
C	-5.526573	-2.542757	0.844939
H	-4.560155	-3.457537	-1.476338
H	-4.334575	-1.938865	-2.356260
H	-5.973789	-2.497204	-1.950556
H	-5.460723	-2.036681	1.813177
H	-4.999142	-3.498111	0.926039
H	-6.580016	-2.744127	0.625869
H	-4.193886	0.329863	-2.053473
H	-5.379615	1.537178	-1.549290
H	-5.920171	0.064920	-2.385411
H	-7.029836	1.075788	0.188406
H	-6.882938	-0.314290	1.271626
H	-7.509633	-0.535301	-0.378231
C	-2.004398	0.083258	1.435086
C	-1.044796	-0.984921	1.741563
C	-1.873898	1.409773	1.757896
H	-1.526258	-1.929180	1.964366
H	-0.232428	-0.758839	2.427497
C	-0.768941	2.180540	2.312372
H	-2.764984	2.008092	1.570259
C	0.581206	1.774269	2.283413
C	-1.062618	3.450772	2.859200
C	1.586059	2.593835	2.791446
H	0.880084	0.853042	1.806757
C	-0.060542	4.259499	3.386251
H	-2.094952	3.793171	2.873218
C	1.271579	3.831694	3.357694
H	2.616565	2.261110	2.714184
H	-0.314716	5.226796	3.811691
H	2.057476	4.466770	3.758106
N	0.125642	-2.380769	0.475231
C	1.188191	-2.775886	1.383988
C	-0.789904	-3.474846	0.207222
C	1.950569	-3.946069	0.736176
H	0.779958	-3.113708	2.354063
H	1.861313	-1.930521	1.534831
C	0.023287	-4.621842	-0.419075
H	-1.583074	-3.141490	-0.464815

H	-1.255371	-3.853703	1.135667
H	2.448543	-3.590071	-0.177832
H	2.711936	-4.331228	1.419737
O	1.070395	-5.026104	0.445467
H	0.425699	-4.291461	-1.391123
H	-0.614428	-5.498480	-0.575295
O	3.385132	0.501435	-1.467059
C	3.381799	-0.153377	-0.386006
O	2.574933	0.096141	0.573712
C	4.339831	-1.304406	-0.218479
C	4.630272	-1.807203	1.056663
C	4.928394	-1.897264	-1.342376
C	5.490648	-2.895063	1.206590
H	4.174666	-1.331536	1.919248
C	5.774057	-2.997461	-1.194853
H	4.704693	-1.484791	-2.320882
C	6.056739	-3.498204	0.079358
H	5.718467	-3.274951	2.199516
H	6.215871	-3.463504	-2.071968
H	6.718371	-4.353092	0.194022
O	0.054154	0.914053	-1.111310
C	-0.751346	1.190654	-2.242964
C	-1.415268	-0.096883	-2.768864
C	0.148848	1.780012	-3.343329
C	-1.832967	2.208709	-1.838109
H	-0.644255	-0.829115	-3.037342
H	-2.053869	-0.544403	-1.997005
H	-2.033356	0.096068	-3.655353
H	-0.420558	2.035363	-4.245760
H	0.635838	2.690494	-2.977742
H	0.932436	1.062042	-3.609879
H	-2.451535	1.802805	-1.032570
H	-1.360057	3.124082	-1.465617
H	-2.484736	2.473939	-2.680512
Li	1.810798	1.434586	-0.726787
O	2.191340	3.310754	-0.485626
C	1.225672	4.335484	-0.212147
C	3.243074	3.932544	-1.237999
C	1.176095	5.196957	-1.490384
H	1.561010	4.917660	0.656365
H	0.289624	3.837921	0.045935
C	2.518943	4.880181	-2.210663
H	3.814173	3.131268	-1.710785
H	3.896604	4.488177	-0.548733
H	0.324438	4.914664	-2.116266
H	1.072075	6.259314	-1.249473
H	2.335286	4.374885	-3.163459
H	3.108928	5.778009	-2.417761

N

Electronic Energy BS1 = -2137.18393631 Hartree
Electronic Energy BS2 = -2137.77662250 Hartree
Zero-point Energy Correction = 0.809628 Hartree
Thermal Correction to Enthalpy = 0.858420 Hartree
Thermal Correction to Free Energy = 0.723430 Hartree

Chemical symbol X, Y, Z

Cu	-0.139180	-0.553372	-0.541065
B	-3.506102	-0.303986	0.847504
O	-4.229358	0.712467	0.268510
O	-4.140587	-1.524195	0.765432
C	-5.491003	-1.278730	0.276755
C	-5.327207	0.099486	-0.464427
C	-4.858825	-0.050259	-1.916893
C	-6.538843	1.022766	-0.389171
C	-5.904881	-2.447939	-0.611024
C	-6.394381	-1.197697	1.512507
H	-5.972697	-3.360752	-0.010769
H	-5.181023	-2.621177	-1.410514
H	-6.885999	-2.261416	-1.061831
H	-6.102705	-0.362349	2.156731
H	-6.292446	-2.122627	2.088084
H	-7.445909	-1.074667	1.234043
H	-3.979736	-0.699346	-1.983229
H	-4.573256	0.931822	-2.302786
H	-5.648664	-0.462143	-2.553265
H	-6.321362	1.956398	-0.917166
H	-6.791735	1.270067	0.644071
H	-7.409607	0.555985	-0.863298
C	-2.121919	-0.102028	1.519156
C	-1.296857	-1.312217	1.908909
C	-1.773309	1.177979	1.815660
H	-1.936372	-2.069707	2.382664
H	-0.527656	-1.031399	2.629714
C	-0.568996	1.733842	2.450641
H	-2.510069	1.937282	1.551885
C	0.721542	1.204167	2.271861
C	-0.700868	2.909722	3.217662
C	1.835180	1.812426	2.848203
H	0.892483	0.362356	1.615857
C	0.407160	3.503354	3.817303
H	-1.686647	3.351023	3.347059
C	1.682320	2.954674	3.635618
H	2.816700	1.396531	2.643555
H	0.280176	4.401767	4.415708
H	2.549299	3.429227	4.087606
N	-0.583287	-1.966435	0.746258
C	0.646784	-2.660978	1.241383
C	-1.449075	-2.987820	0.080933

C	1.342835	-3.396396	0.101182
H	0.359622	-3.371806	2.031016
H	1.325026	-1.907183	1.641949
C	-0.679052	-3.713211	-1.019312
H	-2.329795	-2.492157	-0.324877
H	-1.782531	-3.716891	0.834629
H	1.701864	-2.661517	-0.633784
H	2.207739	-3.943596	0.483553
O	0.479799	-4.349065	-0.510794
H	-0.409728	-3.000016	-1.817857
H	-1.309843	-4.495989	-1.451955
O	3.814304	0.316918	-1.454657
C	3.626335	-0.440030	-0.457889
O	2.581938	-0.377685	0.273403
C	4.669070	-1.475866	-0.119926
C	4.575740	-2.217899	1.064835
C	5.735661	-1.712485	-0.995829
C	5.530157	-3.189076	1.367455
H	3.752866	-2.013862	1.741942
C	6.687566	-2.687890	-0.697877
H	5.796004	-1.123889	-1.905273
C	6.586354	-3.428595	0.483307
H	5.453471	-3.758115	2.290798
H	7.509359	-2.871592	-1.385474
H	7.328928	-4.187987	0.715487
O	0.338094	0.832260	-1.660857
C	-0.480719	1.247411	-2.736773
C	-0.971696	0.022587	-3.531176
C	0.367968	2.146610	-3.651886
C	-1.688239	2.031964	-2.191305
H	-0.112099	-0.577322	-3.849448
H	-1.606728	-0.606854	-2.892844
H	-1.553277	0.310317	-4.416196
H	-0.209580	2.520808	-4.505897
H	0.740132	3.007885	-3.086157
H	1.231253	1.587812	-4.031205
H	-2.228824	1.424004	-1.458431
H	-1.352984	2.944014	-1.684749
H	-2.383315	2.321443	-2.990384
Li	2.075736	1.099403	-1.039654
O	2.342831	2.863753	-0.275433
C	1.220648	3.734570	-0.042065
C	3.453487	3.686987	-0.647548
C	1.433087	4.961977	-0.963723
H	1.205195	4.016432	1.017208
H	0.327818	3.150075	-0.265684
C	2.836056	4.730942	-1.582434
H	4.198099	3.033752	-1.107432
H	3.882966	4.154607	0.252373
H	0.660222	5.029530	-1.735243

H	1.401098	5.890153	-0.383982
H	2.747300	4.308847	-2.588847
H	3.430833	5.647074	-1.648365

O

Electronic Energy BS1 = -1716.27302184 Hartree
Electronic Energy BS2 = -1716.74111047 Hartree
Zero-point Energy Correction = 0.687790 Hartree
Thermal Correction to Enthalpy = 0.728891 Hartree
Thermal Correction to Free Energy = 0.612736 Hartree

Chemical symbol X, Y, Z

Cu	0.478326	1.364704	-1.406326
B	-1.006799	-1.844815	-0.976412
O	-1.189141	-2.821920	-0.010180
O	-2.217413	-1.377303	-1.459255
C	-3.253674	-2.280364	-0.998071
C	-2.602758	-2.906484	0.289951
C	-2.845854	-2.069230	1.553032
C	-2.958759	-4.368147	0.548865
C	-4.533500	-1.486400	-0.750678
C	-3.475890	-3.301471	-2.121426
H	-4.879011	-1.042957	-1.690307
H	-4.373221	-0.680400	-0.032471
H	-5.325543	-2.140709	-0.369108
H	-2.563985	-3.876015	-2.310894
H	-3.733022	-2.765079	-3.039792
H	-4.287002	-3.997476	-1.883102
H	-2.579792	-1.022148	1.381846
H	-2.216925	-2.457123	2.360009
H	-3.891088	-2.124457	1.876663
H	-2.445607	-4.716691	1.450971
H	-2.650641	-5.007868	-0.280745
H	-4.037869	-4.484210	0.702463
C	0.384671	-1.342279	-1.457700
C	0.407348	-0.254791	-2.467178
C	1.450503	-1.969216	-0.861543
H	-0.520444	-0.241426	-3.051723
H	1.259626	-0.307262	-3.155442
C	2.882670	-1.711222	-0.944057
H	1.208073	-2.782760	-0.179292
C	3.466280	-0.553732	-1.511602
C	3.760568	-2.636476	-0.329314
C	4.843574	-0.334639	-1.445667
H	2.837243	0.193074	-1.976474
C	5.133490	-2.424131	-0.279750
H	3.338875	-3.521230	0.140363
C	5.688394	-1.264570	-0.835803
H	5.257628	0.572367	-1.880588

H	5.774225	-3.156342	0.205529
H	6.760823	-1.092793	-0.794911
O	0.392977	2.661801	-0.012837
C	0.784956	4.010035	-0.155569
C	2.263034	4.074020	-0.581263
C	0.599897	4.703832	1.205490
C	-0.092176	4.698646	-1.217074
H	2.891329	3.577932	0.167757
H	2.393307	3.544081	-1.532544
H	2.614407	5.106370	-0.701948
H	0.892223	5.760365	1.173051
H	-0.452646	4.648877	1.512920
H	1.207463	4.201137	1.968052
H	-0.001739	4.162956	-2.169711
H	-1.145137	4.671176	-0.911585
H	0.194577	5.746101	-1.373204
Li	-0.414361	1.479201	1.065724
N	0.846522	0.187587	1.941047
C	0.534733	-1.085205	2.607664
C	2.078717	0.468538	1.742228
C	1.709391	-1.618669	3.420770
H	0.242597	-1.795249	1.826020
H	-0.339586	-0.928543	3.250965
C	3.249306	-0.373683	2.169196
H	2.292692	1.403240	1.219986
H	1.874396	-0.984962	4.310224
H	1.522173	-2.642016	3.758628
O	2.883373	-1.661747	2.620827
H	3.798153	0.183462	2.954091
H	3.930684	-0.505434	1.324301
O	-2.330555	1.362919	0.967186
C	-2.806925	1.771821	-0.361178
C	-3.260582	1.801483	1.979334
C	-4.192036	2.376277	-0.127272
H	-2.790908	0.888320	-1.000714
H	-2.090173	2.502749	-0.743665
C	-4.087769	2.899780	1.314469
H	-2.688295	2.140141	2.849398
H	-3.885941	0.949488	2.279903
H	-4.423050	3.160815	-0.853534
H	-4.971443	1.608449	-0.197344
H	-3.539533	3.849058	1.337055
H	-5.057505	3.047608	1.798877

$TS_{O\text{-}P}$

Imaginary Freq = -366.3556 (cm^{-1})
Electronic Energy BS1 = -1716.23774885 Hartree
Electronic Energy BS2 = -1716.70327215 Hartree
Zero-point Energy Correction = 0.687025 Hartree

Thermal Correction to Enthalpy = 0.727177 Hartree
Thermal Correction to Free Energy = 0.614098 Hartree

Chemical symbol	X,	Y,	Z
Cu	0.421355	1.290572	-1.393608
B	-0.023687	-1.631792	-0.982021
O	-0.223599	-2.711392	-0.162768
O	-1.073709	-1.425025	-1.854940
C	-1.899780	-2.620931	-1.810125
C	-1.589600	-3.172182	-0.372392
C	-2.460819	-2.526688	0.711483
C	-1.611263	-4.691224	-0.240798
C	-3.350699	-2.226754	-2.060576
C	-1.400142	-3.542864	-2.929746
H	-3.458494	-1.844703	-3.080680
H	-3.676055	-1.445248	-1.372683
H	-4.012203	-3.093062	-1.947216
H	-0.360134	-3.838421	-2.759982
H	-1.449160	-3.003601	-3.880572
H	-2.012102	-4.447258	-3.009406
H	-2.411752	-1.436016	0.653610
H	-2.089942	-2.825406	1.694622
H	-3.504308	-2.847760	0.621909
H	-1.362488	-4.973841	0.786840
H	-0.883409	-5.162068	-0.904873
H	-2.607553	-5.084392	-0.473630
C	1.318368	-0.810164	-1.060512
C	1.527337	-0.127303	-2.278717
C	2.224923	-0.877222	0.069877
H	0.887533	-0.389365	-3.123143
H	2.517936	0.241441	-2.542520
C	3.653994	-0.512144	-0.120041
H	2.098485	-1.809521	0.624103
C	4.049433	0.765452	-0.562926
C	4.668977	-1.426891	0.216145
C	5.398447	1.103323	-0.683330
H	3.292002	1.508848	-0.796824
C	6.016614	-1.091668	0.096135
H	4.387519	-2.410857	0.584206
C	6.390557	0.176159	-0.359209
H	5.673434	2.097685	-1.027329
H	6.777941	-1.821670	0.360332
H	7.440839	0.438707	-0.454032
O	-0.647518	2.428593	-0.364520
C	-0.806107	3.805899	-0.647896
C	0.575237	4.470281	-0.786690
C	-1.572694	4.448816	0.522014
C	-1.602799	3.966747	-1.954248
H	1.158169	4.305159	0.126186
H	1.123748	4.017889	-1.623717
H	0.499175	5.549650	-0.968062

H	-1.744997	5.518742	0.355838
H	-2.544571	3.959632	0.654092
H	-1.002869	4.334900	1.452650
H	-1.063176	3.476316	-2.774470
H	-2.580494	3.481168	-1.859232
H	-1.757262	5.020227	-2.218372
Li	-1.030029	1.337666	1.067670
N	0.210534	0.072318	1.757336
C	-0.023591	-1.027204	2.687474
C	1.524375	0.285289	1.468671
C	0.937689	-1.023413	3.872519
H	0.085477	-1.998075	2.177768
H	-1.058795	-0.972818	3.054666
C	2.533267	0.116724	2.610659
H	1.699386	1.227276	0.933900
H	0.768861	-0.130862	4.502355
H	0.819489	-1.918329	4.494668
O	2.280862	-1.034054	3.399183
H	2.453193	1.025555	3.237963
H	3.561787	0.037583	2.259277
O	-2.931662	1.093662	1.286714
C	-3.753742	1.183456	0.096508
C	-3.711211	0.627174	2.408670
C	-5.054563	0.461328	0.451488
H	-3.194958	0.740456	-0.732000
H	-3.925677	2.242202	-0.129036
C	-5.170167	0.733309	1.960257
H	-3.468174	1.248053	3.275958
H	-3.428498	-0.410502	2.625360
H	-5.907249	0.831826	-0.124673
H	-4.959940	-0.615193	0.269858
H	-5.551895	1.745568	2.137470
H	-5.820568	0.025119	2.481688

P

Electronic Energy BS1 = -1716.29128446 Hartree
Electronic Energy BS2 = -1716.75352430 Hartree
Zero-point Energy Correction = 0.689963 Hartree
Thermal Correction to Enthalpy = 0.729364 Hartree
Thermal Correction to Free Energy = 0.618727 Hartree

Chemical symbol X, Y, Z

Cu	0.902800	0.614852	-1.679039
B	-0.312055	-1.423584	-0.266368
O	-0.844134	-2.772424	-0.309925
O	-1.290791	-0.620532	-1.085398
C	-2.140955	-1.513530	-1.814019
C	-2.151822	-2.779925	-0.882180
C	-3.210247	-2.658912	0.229664

C	-2.351471	-4.109302	-1.614239
C	-3.496765	-0.837407	-2.019871
C	-1.512256	-1.802302	-3.184975
H	-3.373857	0.042390	-2.661792
H	-3.926418	-0.508175	-1.071408
H	-4.206391	-1.517732	-2.504529
H	-0.594144	-2.382602	-3.076511
H	-1.269319	-0.851147	-3.671067
H	-2.195945	-2.362087	-3.832355
H	-3.155787	-1.682126	0.719144
H	-3.024454	-3.431448	0.981925
H	-4.225841	-2.792413	-0.160655
H	-2.365580	-4.928251	-0.887261
H	-1.535683	-4.300959	-2.314530
H	-3.301030	-4.122369	-2.162960
C	1.267857	-1.280444	-0.784780
C	1.799065	-1.229050	-2.055345
C	2.100320	-1.155866	0.485296
H	1.209744	-1.514087	-2.925397
H	2.876595	-1.173525	-2.214242
C	3.546331	-0.731551	0.382140
H	2.065449	-2.151252	0.955127
C	3.914265	0.540764	-0.085580
C	4.567695	-1.604693	0.781107
C	5.253432	0.924818	-0.154195
H	3.145426	1.238418	-0.405739
C	5.910403	-1.227183	0.712679
H	4.304495	-2.591762	1.154467
C	6.259095	0.040697	0.244457
H	5.510471	1.916433	-0.518342
H	6.682727	-1.923977	1.028105
H	7.302956	0.338171	0.191295
O	-0.116659	2.014390	-0.948855
C	0.273332	3.368986	-0.826287
C	1.115140	3.780366	-2.045952
C	1.090173	3.567218	0.464618
C	-1.007214	4.220619	-0.763155
H	2.027713	3.169994	-2.091293
H	0.543996	3.605805	-2.964930
H	1.411281	4.836027	-2.009840
H	1.366729	4.617861	0.616927
H	0.507032	3.235533	1.332393
H	2.010717	2.974217	0.430298
H	-1.609336	4.050559	-1.662894
H	-1.602661	3.928156	0.109192
H	-0.784159	5.291851	-0.686869
Li	-1.233516	0.889909	0.124845
N	-0.199625	-0.751957	1.176652
C	-0.567889	-1.702812	2.245913
C	1.184618	-0.233157	1.341649

C	-0.147628	-1.230894	3.627625
H	-0.117509	-2.692929	2.081923
H	-1.649763	-1.855355	2.229737
C	1.611011	0.006959	2.790850
H	1.227342	0.758720	0.876202
H	-0.662493	-0.289387	3.895527
H	-0.392026	-1.986864	4.381276
O	1.259485	-1.041471	3.675951
H	1.149143	0.948359	3.140629
H	2.697657	0.122929	2.842330
O	-2.760751	1.597790	1.064704
C	-3.916619	2.111886	0.366386
C	-3.148334	1.085006	2.364641
C	-5.110872	1.445912	1.045025
H	-3.802612	1.866063	-0.692182
H	-3.945866	3.203872	0.475218
C	-4.643226	1.395427	2.508881
H	-2.525605	1.564191	3.125963
H	-2.947429	0.009455	2.376484
H	-6.041268	2.003630	0.904371
H	-5.252359	0.431714	0.653230
H	-4.790419	2.370915	2.986673
H	-5.166358	0.642574	3.105311

14.9.4. Cartesian coordinates for the Cu/LiO^{t}Bu/P(O)Ph$_3$ system (Figure S21)

I_{TPO}

Electronic Energy BS1 = -2193.47579079 Hartree
Electronic Energy BS2 = -2194.00413746 Hartree
Zero-point Energy Correction = 0.712017 Hartree
Thermal Correction to Enthalpy = 0.757252 Hartree
Thermal Correction to Free Energy = 0.630373 Hartree

Chemical symbol X, Y, Z

Cu	1.702740	-1.630954	-0.331318
B	2.757157	-0.027478	-0.674334
O	2.125214	1.229354	-0.767510
O	4.136697	0.157288	-0.765619
C	3.122134	2.277288	-0.701220
C	4.414819	1.522797	-1.164338
C	2.676163	3.430958	-1.596831
C	3.182492	2.742956	0.760415
C	5.706951	1.977862	-0.489724
C	4.591308	1.515124	-2.689488
H	3.439076	4.217841	-1.635104
H	1.753829	3.864606	-1.196442
H	2.473293	3.090220	-2.614808
H	2.180510	3.051434	1.073516

H	3.867702	3.587965	0.892184
H	3.505635	1.926024	1.411725
H	5.909220	3.034603	-0.702891
H	6.547211	1.385903	-0.867003
H	5.660843	1.840949	0.593074
H	5.382298	0.805002	-2.949315
H	4.867451	2.503117	-3.075130
H	3.670524	1.188393	-3.182876
O	0.425811	-2.914776	0.312948
C	0.054083	-4.066189	-0.416071
Li	-0.458300	-1.789918	1.425508
C	-1.057275	-4.793041	0.363566
C	1.278525	-4.987533	-0.562423
C	-0.467954	-3.660983	-1.806297
H	-1.381097	-5.711684	-0.141395
H	-1.931437	-4.137442	0.471345
H	-0.698816	-5.059091	1.365732
H	1.657949	-5.262649	0.428504
H	2.076290	-4.451520	-1.091229
H	1.043387	-5.905307	-1.116797
H	0.317415	-3.129112	-2.355839
H	-1.317678	-2.978360	-1.703520
H	-0.783077	-4.530366	-2.398286
O	-1.983153	-0.742823	1.258982
P	-2.371328	0.343373	0.263697
C	-4.166325	0.629371	0.338356
C	-1.558033	1.915156	0.691075
C	-1.959720	-0.009952	-1.463017
C	-4.792774	1.594481	-0.464750
C	-4.925461	-0.132975	1.234881
C	-1.390203	2.958285	-0.230953
C	-1.117706	2.076582	2.012126
C	-0.643707	0.207631	-1.900946
C	-2.895750	-0.607466	-2.323971
C	-6.169126	1.791908	-0.371024
H	-4.208478	2.187081	-1.163674
C	-6.304541	0.068261	1.325247
H	-4.427663	-0.876023	1.850281
C	-0.793276	4.153586	0.169100
H	-1.698140	2.828846	-1.264227
C	-0.516915	3.272240	2.406996
H	-1.234463	1.257559	2.715043
C	-0.272597	-0.165771	-3.192385
H	0.099503	0.661156	-1.253950
C	-2.516383	-0.973470	-3.614435
H	-3.913448	-0.784246	-1.988561
C	-6.925385	1.028361	0.524292
H	-6.652611	2.538657	-0.994687
H	-6.892239	-0.525360	2.019923
C	-0.356863	4.311605	1.487514

H	-0.658850	4.956863	-0.549838
H	-0.168469	3.389559	3.429345
C	-1.205022	-0.752856	-4.048524
H	0.754171	-0.008595	-3.508491
H	-3.240924	-1.435485	-4.279163
H	-7.998524	1.183667	0.595553
H	0.114855	5.241031	1.794513
H	-0.911689	-1.047139	-5.052721
O	0.746499	-1.290944	2.892379
C	1.501483	-0.053908	2.784922
C	1.635659	-2.383015	3.256982
C	2.973763	-0.446273	2.922703
H	1.171720	0.615429	3.589944
H	1.281077	0.405903	1.817376
C	2.904309	-1.716121	3.784184
H	1.821320	-2.977020	2.355487
H	1.116848	-2.996617	4.000506
H	3.382814	-0.677480	1.933099
H	3.574175	0.349735	3.374123
H	3.783883	-2.357227	3.671902
H	2.795602	-1.460809	4.845490

J_{TPO}

Electronic Energy BS1 = -2541.21108859 Hartree
Electronic Energy BS2 = -2541.83861434 Hartree
Zero-point Energy Correction = 0.851678 Hartree
Thermal Correction to Enthalpy = 0.905920 Hartree
Thermal Correction to Free Energy = 0.757838 Hartree

Chemical symbol X, Y, Z

Cu	-1.559840	0.594352	-1.528930
C	-3.028067	-0.551402	-2.515318
C	-3.235564	-0.291530	-1.174813
H	-3.484418	0.069116	-3.286227
C	-3.877170	-0.550315	-0.033590
H	-3.479546	-0.127439	0.885745
H	-2.655522	-1.523036	-2.839424
C	-5.074098	-1.394264	0.117887
C	-5.622523	-1.569241	1.401800
C	-5.693685	-2.051472	-0.962305
C	-6.746082	-2.369782	1.603341
H	-5.156657	-1.067575	2.247655
C	-6.816669	-2.849656	-0.760529
H	-5.284343	-1.925256	-1.960011
C	-7.349855	-3.015211	0.522178
H	-7.150939	-2.488762	2.605376
H	-7.280779	-3.346660	-1.609040
H	-8.226169	-3.639490	0.675475
B	-1.405188	1.532079	0.272125

O	-1.002726	0.913734	1.462306
O	-1.820636	2.830688	0.538775
C	-0.965281	1.899973	2.532828
C	-1.899812	3.032229	1.973423
C	-1.449147	1.229872	3.816715
C	0.496517	2.336651	2.686096
C	-1.440694	4.456288	2.281934
C	-3.373997	2.860216	2.363613
H	-1.505265	1.953457	4.638914
H	-0.744645	0.442039	4.102110
H	-2.433533	0.774100	3.685349
H	1.107155	1.458887	2.908550
H	0.617411	3.058477	3.502079
H	0.863707	2.777902	1.756176
H	-1.415279	4.637370	3.363175
H	-2.138212	5.170757	1.832284
H	-0.447384	4.648938	1.870124
H	-3.975748	3.572790	1.791157
H	-3.537815	3.042074	3.431727
H	-3.730450	1.855450	2.118411
O	0.076482	0.954465	-2.507696
C	0.480328	0.427108	-3.751706
Li	0.960062	1.289255	-0.962880
C	1.881411	0.991187	-4.061074
C	-0.508403	0.866714	-4.844159
C	0.549084	-1.108679	-3.681401
H	2.271487	0.628227	-5.020450
H	2.583229	0.698235	-3.269255
H	1.843330	2.086848	-4.094304
H	-0.555088	1.961414	-4.882366
H	-1.507787	0.493940	-4.598657
H	-0.224788	0.491497	-5.835803
H	-0.428647	-1.507517	-3.395052
H	1.268461	-1.418338	-2.916239
H	0.844270	-1.553673	-4.640591
O	2.319982	0.332130	-0.135420
P	2.469498	-1.050985	0.478154
C	4.115433	-1.715894	0.082536
C	2.384120	-0.993128	2.295906
C	1.248185	-2.257114	-0.113308
C	4.729296	-2.707652	0.861213
C	4.757467	-1.231315	-1.065682
C	1.414335	-1.671354	3.044974
C	3.317054	-0.171886	2.952871
C	-0.120030	-1.981428	0.063262
C	1.642298	-3.414069	-0.804088
C	5.975106	-3.213297	0.489545
H	4.238319	-3.077274	1.757514
C	6.004207	-1.739508	-1.433008
H	4.277645	-0.455973	-1.655724

C	1.372385	-1.521540	4.433504
H	0.687460	-2.306340	2.550614
C	3.273784	-0.027696	4.337511
H	4.067977	0.361192	2.376117
C	-1.075702	-2.863309	-0.442710
H	-0.443114	-1.081810	0.579908
C	0.679124	-4.285279	-1.313427
H	2.694730	-3.630721	-0.955401
C	6.611631	-2.730096	-0.657468
H	6.450751	-3.979558	1.095360
H	6.501112	-1.361687	-2.322215
C	2.297572	-0.700290	5.079371
H	0.613163	-2.045462	5.007305
H	3.994849	0.612538	4.837851
C	-0.678063	-4.011298	-1.131611
H	-2.127678	-2.632796	-0.311675
H	0.990363	-5.174277	-1.854985
H	7.582813	-3.124313	-0.944303
H	2.259633	-0.583261	6.158941
H	-1.427606	-4.689626	-1.530637
O	1.217319	3.174268	-0.628827
C	2.549727	3.722807	-0.559229
C	0.364569	4.006757	-1.464601
C	2.462802	5.105564	-1.213439
H	3.232243	3.056967	-1.103175
H	2.856122	3.749485	0.491501
C	1.331440	4.902909	-2.234550
H	-0.307308	4.570985	-0.810273
H	-0.221009	3.329808	-2.089490
H	2.172976	5.860792	-0.472787
H	3.410966	5.415266	-1.663656
H	0.868058	5.839137	-2.560380
H	1.708583	4.377868	-3.120657

TS-J_{TPO}-K_{TPO}

Imaginary Freq = -170.2983 (cm^{-1})
Electronic Energy BS1 = -2541.20053671 Hartree
Electronic Energy BS2 = -2541.83066268 Hartree
Zero-point Energy Correction = 0.851234 Hartree
Thermal Correction to Enthalpy = 0.904707 Hartree
Thermal Correction to Free Energy = 0.759280 Hartree

Chemical symbol X, Y, Z

Cu	-1.291894	0.885021	-1.533708
C	-3.263890	0.507554	-2.395873
C	-2.916453	-0.051760	-1.163638
H	-3.500425	1.565931	-2.505544
C	-3.228515	-1.183696	-0.465662
H	-2.518295	-1.542804	0.273730

H	-3.589759	-0.141945	-3.205986
C	-4.482401	-1.928574	-0.514522
C	-4.629977	-3.054961	0.327681
C	-5.586355	-1.581250	-1.325629
C	-5.806592	-3.798753	0.353468
H	-3.798185	-3.341633	0.969197
C	-6.765587	-2.320094	-1.288176
H	-5.510388	-0.715991	-1.975085
C	-6.887504	-3.437891	-0.455799
H	-5.882140	-4.662249	1.010851
H	-7.599572	-2.022792	-1.920318
H	-7.807054	-4.016941	-0.439703
B	-1.771293	0.971502	0.418881
O	-1.171558	0.143466	1.363519
O	-2.536794	1.953603	1.027333
C	-1.461559	0.671931	2.688962
C	-2.719546	1.573929	2.423000
C	-1.687979	-0.506644	3.630721
C	-0.229211	1.476115	3.115653
C	-2.789593	2.848671	3.259347
C	-4.045689	0.808650	2.505132
H	-1.977151	-0.158699	4.629646
H	-0.762442	-1.084098	3.722375
H	-2.468022	-1.171492	3.252563
H	0.644515	0.821573	3.089208
H	-0.337063	1.878943	4.128986
H	-0.055420	2.296373	2.415884
H	-2.848961	2.611624	4.328061
H	-3.684080	3.416995	2.985093
H	-1.919395	3.488200	3.091119
H	-4.849266	1.455779	2.140463
H	-4.275972	0.512675	3.534414
H	-4.027507	-0.082806	1.872284
O	0.296989	1.701821	-2.132850
C	0.621120	1.832835	-3.506398
Li	1.167029	1.744937	-0.549897
C	2.030253	2.445126	-3.588940
C	-0.401133	2.761406	-4.182232
C	0.608655	0.451370	-4.180281
H	2.351714	2.592938	-4.627177
H	2.755447	1.785249	-3.095544
H	2.047432	3.417249	-3.080433
H	-0.390187	3.745883	-3.699060
H	-1.404768	2.335341	-4.067314
H	-0.194018	2.893781	-5.251574
H	-0.391574	0.011026	-4.095532
H	1.313012	-0.216863	-3.674688
H	0.876242	0.511171	-5.243021
O	2.466093	0.680112	0.240843
P	2.709316	-0.823338	0.259732

C	4.477261	-1.162947	-0.000184
C	2.292903	-1.538710	1.880560
C	1.798871	-1.755181	-1.001185
C	5.058549	-2.384364	0.370477
C	5.256458	-0.176568	-0.619617
C	1.625350	-2.761100	2.033029
C	2.690831	-0.818616	3.018735
C	0.395164	-1.796457	-0.926087
C	2.458621	-2.377914	-2.072324
C	6.409662	-2.617596	0.116468
H	4.458739	-3.146054	0.861850
C	6.608933	-0.414276	-0.870867
H	4.797911	0.769546	-0.891582
C	1.351914	-3.252986	3.311553
H	1.305026	-3.320578	1.160238
C	2.418259	-1.313986	4.292307
H	3.196093	0.135777	2.900184
C	-0.336099	-2.452944	-1.915907
H	-0.124382	-1.310249	-0.105329
C	1.719716	-3.036544	-3.056158
H	3.541308	-2.348131	-2.141489
C	7.184189	-1.633371	-0.505249
H	6.859432	-3.563158	0.406205
H	7.212350	0.351703	-1.349931
C	1.746526	-2.531544	4.439628
H	0.823971	-4.195696	3.423655
H	2.721173	-0.748541	5.169058
C	0.325922	-3.073824	-2.977763
H	-1.420061	-2.452893	-1.854830
H	2.233807	-3.513304	-3.886097
H	8.237224	-1.816771	-0.700902
H	1.528555	-2.914529	5.432788
H	-0.247436	-3.579692	-3.749780
O	0.877609	3.407761	0.358511
C	1.704817	4.105828	1.305435
C	-0.240185	4.245227	-0.061033
C	0.828538	5.242319	1.831489
H	2.597415	4.492343	0.793026
H	2.020263	3.390018	2.069880
C	0.018536	5.612728	0.577679
H	-1.156842	3.777486	0.307393
H	-0.259740	4.248769	-1.152951
H	0.162621	4.869504	2.619172
H	1.412390	6.073630	2.238039
H	-0.910672	6.143098	0.805569
H	0.619306	6.246494	-0.085859

K_{TPO}

Electronic Energy BS1 = -2541.28060818 Hartree

Electronic Energy BS2 = -2541.91259879 Hartree
Zero-point Energy Correction = 0.854999 Hartree
Thermal Correction to Enthalpy = 0.908295 Hartree
Thermal Correction to Free Energy = 0.762468 Hartree

Chemical symbol X, Y, Z

Cu	-1.893268	-0.866345	-1.733657
C	-3.630417	-0.198616	-1.177711
C	-3.481606	0.210284	0.233662
H	-3.824950	0.674077	-1.815758
C	-3.780570	-0.502717	1.367096
H	-3.625151	0.009923	2.316853
H	-4.412260	-0.947725	-1.351510
C	-4.219190	-1.888559	1.522217
C	-4.723174	-2.307609	2.775329
C	-4.064635	-2.881905	0.527249
C	-5.072824	-3.631993	3.019107
H	-4.833048	-1.568468	3.566628
C	-4.403334	-4.211820	0.778375
H	-3.635643	-2.615339	-0.430599
C	-4.916351	-4.598031	2.018919
H	-5.464042	-3.915474	3.993671
H	-4.260994	-4.952808	-0.005401
H	-5.187241	-5.633938	2.206299
B	-2.857027	1.613774	0.482114
O	-2.195714	1.963536	1.646846
O	-2.876275	2.656035	-0.429828
C	-1.578474	3.256854	1.434223
C	-2.452299	3.850883	0.273503
C	-1.614541	4.040066	2.743514
C	-0.127821	2.992387	1.015792
C	-1.700001	4.756931	-0.695703
C	-3.724935	4.547386	0.772769
H	-1.238731	5.059367	2.595829
H	-0.976870	3.545274	3.483466
H	-2.626798	4.093908	3.150251
H	0.360850	2.398780	1.792033
H	0.438356	3.922542	0.899921
H	-0.084770	2.436325	0.074043
H	-1.316039	5.644022	-0.178836
H	-2.374796	5.088863	-1.491719
H	-0.863265	4.230256	-1.155029
H	-4.380226	4.741927	-0.081689
H	-3.499037	5.499191	1.265334
H	-4.267620	3.908274	1.476152
O	-0.118679	-1.280320	-2.283952
C	0.195466	-2.510207	-2.908619
Li	0.928886	0.127206	-1.901445
C	1.727383	-2.678623	-2.905981
C	-0.329161	-2.484342	-4.355168
C	-0.442745	-3.678964	-2.136906

H	2.036796	-3.601486	-3.411866
H	2.098965	-2.708956	-1.875505
H	2.204254	-1.833386	-3.420985
H	0.130477	-1.653584	-4.904741
H	-1.413407	-2.321048	-4.341763
H	-0.118711	-3.418477	-4.891794
H	-1.535537	-3.583425	-2.148135
H	-0.115971	-3.655182	-1.093543
H	-0.174961	-4.649924	-2.573717
O	2.517037	0.356494	-0.964856
P	2.836210	-0.209092	0.415526
C	4.459930	-1.021788	0.409252
C	2.892122	1.128449	1.645820
C	1.626352	-1.419483	1.004292
C	5.220119	-1.175358	1.577441
C	4.925454	-1.546723	-0.804887
C	2.512700	0.938403	2.982080
C	3.308171	2.396295	1.212099
C	0.300017	-0.995102	1.197263
C	1.955884	-2.772793	1.181575
C	6.437413	-1.854436	1.530273
H	4.866511	-0.759432	2.517080
C	6.143996	-2.225614	-0.845894
H	4.332007	-1.417594	-1.704747
C	2.548907	2.010708	3.875892
H	2.162891	-0.034081	3.315533
C	3.346298	3.462581	2.109155
H	3.575655	2.542042	0.169730
C	-0.681987	-1.915208	1.552087
H	0.016934	0.045712	1.064157
C	0.966365	-3.688087	1.545016
H	2.976178	-3.111548	1.031111
C	6.898114	-2.380547	0.319825
H	7.027543	-1.970180	2.435044
H	6.503913	-2.632335	-1.786750
C	2.964622	3.270994	3.440132
H	2.242503	1.863446	4.907597
H	3.662357	4.444947	1.769251
C	-0.351725	-3.261395	1.724149
H	-1.703333	-1.576831	1.670605
H	1.225369	-4.735264	1.675223
H	7.847018	-2.908902	0.285732
H	2.984762	4.105337	4.135857
H	-1.132235	-3.971918	1.981649
O	0.321293	1.880422	-2.390934
C	1.272295	2.950309	-2.601268
C	-0.828355	2.046444	-3.265875
C	0.659742	3.861639	-3.676240
H	2.219114	2.502861	-2.924277
H	1.440978	3.458152	-1.645937

C	-0.313668	2.919829	-4.406166
H	-1.635335	2.517061	-2.695293
H	-1.148627	1.046275	-3.565499
H	0.108397	4.687065	-3.213095
H	1.420786	4.292227	-4.333888
H	-1.118210	3.450262	-4.923974
H	0.222756	2.306605	-5.140392

L_{TPO}

Electronic Energy BS1 = -3016.22773767 Hartree
Electronic Energy BS2 = -3016.99556329 Hartree
Zero-point Energy Correction = 0.968917 Hartree
Thermal Correction to Enthalpy = 1.029531 Hartree
Thermal Correction to Free Energy = 0.870710 Hartree

Chemical symbol X, Y, Z

P	0.685715	-2.015307	0.703573
C	-0.614282	-3.182991	0.216928
C	2.195587	-2.596579	-0.126409
C	0.962766	-2.190746	2.494786
C	-0.476395	-4.566991	0.412048
C	-1.735988	-2.673197	-0.447600
C	3.075612	-3.532890	0.435441
C	2.415621	-2.119031	-1.427125
C	2.111221	-1.609408	3.059749
C	0.001132	-2.774725	3.331594
C	-1.462015	-5.434770	-0.052972
H	0.401474	-4.964735	0.915509
C	-2.708590	-3.551404	-0.933771
H	-1.833901	-1.604341	-0.605578
C	4.165472	-3.994499	-0.306031
H	2.917575	-3.894084	1.447871
C	3.504158	-2.585777	-2.162029
H	1.742380	-1.384236	-1.854775
C	2.296692	-1.626302	4.441152
H	2.839630	-1.119603	2.421417
C	0.189474	-2.784351	4.714696
H	-0.894539	-3.215032	2.904707
C	-2.574753	-4.925792	-0.732966
H	-1.357962	-6.505415	0.101100
H	-3.556363	-3.139464	-1.469917
C	4.375614	-3.525169	-1.605736
H	4.847454	-4.719576	0.130422
H	3.667134	-2.202329	-3.164079
C	1.336157	-2.213497	5.269644
H	3.184631	-1.171948	4.871825
H	-0.562357	-3.233438	5.357530
H	-3.333664	-5.606243	-1.110789
H	5.224286	-3.885977	-2.180934

H	1.478925	-2.219404	6.346775
Cu	-1.551946	2.206059	0.183751
B	-3.966182	0.454798	-1.501378
O	-4.400450	-0.861649	-1.537297
O	-4.907675	1.288179	-0.935755
C	-6.135325	0.537796	-0.780764
C	-5.614912	-0.945936	-0.744248
C	-5.206580	-1.401825	0.662711
C	-6.544934	-1.973882	-1.383142
C	-6.837576	1.009057	0.489751
C	-6.998851	0.842302	-2.012071
H	-7.151651	2.050787	0.369771
H	-6.172899	0.956192	1.354734
H	-7.728729	0.402692	0.689818
H	-6.501825	0.509588	-2.928752
H	-7.145210	1.924420	-2.080786
H	-7.980110	0.359894	-1.948594
H	-4.527326	-0.680970	1.125415
H	-4.682201	-2.358723	0.596074
H	-6.080201	-1.527308	1.310973
H	-6.102510	-2.972841	-1.305874
H	-6.713128	-1.759260	-2.440709
H	-7.512548	-1.991926	-0.868638
C	-2.571863	0.951635	-1.990324
C	-2.236760	2.344271	-1.629070
C	-1.800811	0.031600	-2.656952
H	-3.135861	2.970829	-1.584118
H	-1.498581	2.815593	-2.290554
C	-0.424724	0.123689	-3.132376
H	-2.267801	-0.929832	-2.868525
C	0.525971	1.051443	-2.643978
C	0.030853	-0.803788	-4.098596
C	1.836959	1.066090	-3.119995
H	0.235118	1.729733	-1.852046
C	1.334422	-0.775953	-4.588499
H	-0.666217	-1.550623	-4.472856
C	2.249659	0.167484	-4.108608
H	2.547070	1.779062	-2.709176
H	1.639576	-1.496073	-5.344458
H	3.268322	0.194574	-4.487115
N	1.883620	3.257497	0.100012
C	2.152442	3.970503	1.366450
C	1.557475	4.224235	-0.965760
C	0.909483	4.793238	1.720077
H	3.026325	4.628216	1.249836
H	2.350094	3.227545	2.143356
C	0.361304	5.074870	-0.524778
H	1.316459	3.670726	-1.875575
H	2.422486	4.876875	-1.155605
H	0.064982	4.109226	1.870710

H	1.098075	5.357673	2.638006
O	0.634327	5.743964	0.697067
H	-0.534011	4.442801	-0.435555
H	0.174038	5.843869	-1.279721
O	2.744770	0.899961	0.953758
C	3.457579	1.486746	0.147630
O	3.171463	2.703549	-0.348261
C	4.736178	0.953254	-0.380602
C	5.562045	1.711810	-1.226648
C	5.109444	-0.349007	-0.021301
C	6.752904	1.168001	-1.701065
H	5.269373	2.718093	-1.503711
C	6.303081	-0.885343	-0.496924
H	4.450430	-0.939222	0.603998
C	7.125904	-0.129052	-1.334782
H	7.391092	1.755145	-2.355055
H	6.578351	-1.899275	-0.225382
H	8.056045	-0.550333	-1.706735
O	-0.623794	1.955097	1.825540
C	-1.325505	1.616781	3.007556
C	-2.203186	2.806651	3.437228
C	-0.288732	1.306900	4.101522
C	-2.215217	0.381949	2.766138
H	-1.580354	3.689075	3.623580
H	-2.904049	3.053287	2.630173
H	-2.777232	2.587523	4.346808
H	-0.763634	1.073000	5.062461
H	0.327243	0.449444	3.810141
H	0.371108	2.171717	4.242745
H	-2.961433	0.616603	1.997665
H	-1.608848	-0.450766	2.398676
H	-2.738751	0.065697	3.677963
Li	0.747092	1.106174	0.934180
O	0.326354	-0.604398	0.288397

TS-L_{TPO}-M'_{TPO}

Imaginary Freq = -248.0145 (cm^{-1})
Electronic Energy BS1 = -3016.20631112 Hartree
Electronic Energy BS2 = -3016.97111321 Hartree
Zero-point Energy Correction = 0.967025 Hartree
Thermal Correction to Enthalpy = 1.027303 Hartree
Thermal Correction to Free Energy = 0.870315 Hartree

Chemical symbol X, Y, Z

P	1.155989	-2.097843	0.503612
C	0.079966	-3.358586	-0.233285
C	2.787166	-2.283728	-0.270808
C	1.375376	-2.475011	2.273026
C	0.497258	-4.686424	-0.415734

C	-1.186325	-2.952779	-0.675022
C	3.802662	-3.080817	0.276967
C	2.995855	-1.614111	-1.485639
C	2.067148	-1.525882	3.047706
C	0.821292	-3.603577	2.890327
C	-0.358533	-5.606066	-1.020683
H	1.491796	-4.995290	-0.103063
C	-2.029502	-3.872802	-1.301669
H	-1.493252	-1.919214	-0.562929
C	5.018095	-3.215116	-0.396958
H	3.650258	-3.584126	1.227958
C	4.213040	-1.749709	-2.150110
H	2.217313	-0.981631	-1.896251
C	2.196311	-1.709563	4.423274
H	2.480492	-0.634743	2.581431
C	0.959379	-3.784336	4.268610
H	0.271588	-4.331808	2.302241
C	-1.620160	-5.197336	-1.466629
H	-0.036516	-6.634449	-1.160134
H	-2.989851	-3.529834	-1.671237
C	5.221266	-2.551517	-1.610051
H	5.806265	-3.831281	0.027647
H	4.368917	-1.214872	-3.081456
C	1.643175	-2.839005	5.034809
H	2.723595	-0.968768	5.017867
H	0.523101	-4.658822	4.743405
H	-2.277757	-5.912355	-1.954311
H	6.171300	-2.651139	-2.128396
H	1.741074	-2.977995	6.108047
Cu	-1.544896	2.043156	0.275557
B	-3.692325	0.108422	-1.431859
O	-3.985650	-1.239623	-1.548609
O	-4.721118	0.810295	-0.839230
C	-5.876442	-0.063301	-0.776027
C	-5.216747	-1.491135	-0.816745
C	-4.819036	-2.006961	0.570822
C	-6.019258	-2.552038	-1.565313
C	-6.659623	0.251301	0.495445
C	-6.726886	0.242517	-2.015763
H	-7.068201	1.264879	0.432484
H	-6.023057	0.201127	1.381049
H	-7.494687	-0.447970	0.618868
H	-6.172177	0.024761	-2.933915
H	-6.977917	1.307425	-2.017189
H	-7.656892	-0.335702	-2.021285
H	-4.259102	-1.250558	1.124765
H	-4.175936	-2.884873	0.458673
H	-5.698306	-2.291047	1.158506
H	-5.490861	-3.510714	-1.529951
H	-6.160812	-2.281906	-2.613984

H	-7.002179	-2.691442	-1.101064
C	-2.359799	0.773360	-1.890384
C	-2.190976	2.201739	-1.554997
C	-1.469783	-0.037733	-2.552943
H	-3.153686	2.723880	-1.505383
H	-1.502598	2.741340	-2.212564
C	-0.126102	0.258475	-3.041532
H	-1.806197	-1.048861	-2.776505
C	0.712134	1.247762	-2.480375
C	0.404794	-0.509018	-4.102408
C	1.985921	1.492274	-2.989573
H	0.382688	1.788898	-1.604874
C	1.674808	-0.259226	-4.618792
H	-0.203873	-1.302301	-4.531130
C	2.470538	0.754768	-4.074406
H	2.604926	2.247768	-2.516343
H	2.046477	-0.859024	-5.446255
H	3.461394	0.953757	-4.475008
N	0.494500	3.458385	0.309691
C	0.542874	4.080218	1.640066
C	0.312785	4.501972	-0.703396
C	-0.746200	4.896916	1.880499
H	1.395292	4.766430	1.698042
H	0.626276	3.291808	2.386920
C	-0.965012	5.321674	-0.402669
H	0.241865	4.032600	-1.685969
H	1.164512	5.191061	-0.687453
H	-1.608508	4.216934	1.907021
H	-0.666355	5.410960	2.843497
O	-0.902765	5.896037	0.889301
H	-1.846821	4.675870	-0.506912
H	-1.032962	6.148207	-1.116866
O	2.306380	1.476837	1.414927
C	2.849898	2.173928	0.536094
O	2.318031	3.203841	-0.041693
C	4.237448	1.842471	0.060626
C	4.909377	2.662657	-0.856871
C	4.855085	0.676459	0.529846
C	6.183842	2.315573	-1.301748
H	4.424980	3.565707	-1.212272
C	6.129703	0.332287	0.084619
H	4.321808	0.038557	1.224190
C	6.795560	1.149675	-0.831842
H	6.700463	2.953914	-2.013372
H	6.593072	-0.583021	0.439711
H	7.788370	0.878364	-1.181554
O	-1.016280	1.493188	2.023257
C	-1.917563	0.876397	2.924086
C	-3.337504	1.440215	2.730427
C	-1.424850	1.178116	4.349893

C	-1.930393	-0.645528	2.685842
H	-3.322688	2.531260	2.837934
H	-3.710700	1.209553	1.724886
H	-4.041869	1.028193	3.465086
H	-2.059757	0.709880	5.112730
H	-0.401472	0.803799	4.475159
H	-1.414870	2.260909	4.520486
H	-2.133353	-0.845651	1.629004
H	-0.955028	-1.081701	2.925577
H	-2.688830	-1.152919	3.295958
Li	0.537093	0.829203	1.310314
O	0.577384	-0.714673	0.288299

M'$_{TPO}$

Electronic Energy BS1 = -3016.25676508 Hartree
Electronic Energy BS2 = -3017.02006416 Hartree
Zero-point Energy Correction = 0.967421 Hartree
Thermal Correction to Enthalpy = 1.028442 Hartree
Thermal Correction to Free Energy = 0.869129 Hartree

Chemical symbol X, Y, Z

P	2.370894	1.342935	0.304532
C	3.740773	1.282982	-0.892397
C	1.540673	2.933699	0.036485
C	3.086526	1.375092	1.975580
C	4.650624	2.341574	-1.032651
C	3.853036	0.148550	-1.708408
C	1.860603	4.095760	0.752933
C	0.569176	2.979833	-0.973567
C	2.212611	1.581981	3.059261
C	4.440915	1.100801	2.212740
C	5.677604	2.257258	-1.972267
H	4.550664	3.232273	-0.417307
C	4.881068	0.073539	-2.651648
H	3.141263	-0.663406	-1.600498
C	1.205201	5.292626	0.460001
H	2.598078	4.062069	1.549783
C	-0.073139	4.180609	-1.268150
H	0.289186	2.073521	-1.497869
C	2.702488	1.516229	4.363206
H	1.158333	1.778380	2.879936
C	4.921812	1.038556	3.522013
H	5.118669	0.935443	1.380863
C	5.793878	1.122244	-2.781584
H	6.382198	3.077468	-2.078943
H	4.963356	-0.805940	-3.284881
C	0.240337	5.335906	-0.549485
H	1.439443	6.188446	1.028670
H	-0.838476	4.199098	-2.037028

C	4.054284	1.245559	4.596163
H	2.025536	1.674040	5.198267
H	5.972279	0.826152	3.701260
H	6.592552	1.061216	-3.516115
H	-0.282289	6.264130	-0.761353
H	4.430283	1.194359	5.614594
Cu	-2.435734	-1.533072	0.101925
B	0.805918	-2.970767	-1.122577
O	2.114342	-2.645634	-1.412364
O	0.700831	-4.133557	-0.399111
C	2.019848	-4.745442	-0.361702
C	2.968605	-3.515781	-0.621526
C	3.340730	-2.746765	0.652006
C	4.222393	-3.827411	-1.434778
C	2.207540	-5.435317	0.985184
C	2.049418	-5.778846	-1.495187
H	1.489283	-6.255749	1.081717
H	2.046053	-4.747940	1.816635
H	3.217748	-5.853005	1.064755
H	1.915091	-5.296303	-2.468451
H	1.226101	-6.484831	-1.350197
H	2.990394	-6.338560	-1.507726
H	2.447744	-2.439847	1.199402
H	3.883392	-1.838270	0.378048
H	3.980374	-3.346426	1.307854
H	4.802252	-2.909758	-1.578855
H	3.974959	-4.230885	-2.419195
H	4.855201	-4.549896	-0.907054
C	-0.406698	-2.171723	-1.697267
C	-1.736415	-2.680210	-1.432469
C	-0.086642	-1.057633	-2.442471
H	-1.771342	-3.661756	-0.962002
H	-2.512718	-2.509223	-2.172050
C	-0.861737	0.004843	-3.061661
H	0.982681	-0.910368	-2.572953
C	-2.256912	0.191161	-2.936388
C	-0.128449	0.959295	-3.808258
C	-2.873967	1.286573	-3.536573
H	-2.871247	-0.481575	-2.345761
C	-0.750615	2.051334	-4.402816
H	0.949079	0.840696	-3.898848
C	-2.132727	2.219871	-4.267034
H	-3.946823	1.415261	-3.418709
H	-0.161860	2.772279	-4.963292
H	-2.625864	3.073506	-4.725063
N	-4.077911	-1.366544	-0.807782
C	-4.840404	-0.210392	-0.323705
C	-4.916488	-2.563225	-0.728144
C	-5.476141	-0.441139	1.049073
H	-5.649630	-0.003939	-1.048362

H	-4.185830	0.661918	-0.287660
C	-5.539636	-2.765133	0.657388
H	-4.332181	-3.450678	-1.004412
H	-5.739956	-2.471297	-1.459336
H	-4.677777	-0.521589	1.808740
H	-6.138487	0.384064	1.333231
O	-6.279849	-1.619302	1.061597
H	-4.734716	-2.971940	1.387076
H	-6.243463	-3.605503	0.669015
O	-0.810256	1.341811	1.995079
C	-1.624163	1.562371	1.052109
O	-1.876818	0.704998	0.138538
C	-2.283755	2.909397	0.948999
C	-3.046071	3.236122	-0.180541
C	-2.084005	3.868544	1.948274
C	-3.609421	4.505434	-0.304170
H	-3.154051	2.497740	-0.967281
C	-2.647451	5.138271	1.825058
H	-1.475819	3.603800	2.806539
C	-3.411353	5.459096	0.698843
H	-4.195962	4.754673	-1.185069
H	-2.489158	5.880059	2.603796
H	-3.849386	6.449613	0.602757
O	-0.939587	-1.853133	1.205994
C	-1.139063	-2.546886	2.432394
C	-1.590229	-3.988196	2.141859
C	-2.184772	-1.813838	3.293591
C	0.209928	-2.554470	3.169478
H	-2.553962	-3.977741	1.616267
H	-0.858050	-4.480575	1.496572
H	-1.714081	-4.567586	3.064996
H	-2.297058	-2.279000	4.280856
H	-1.891949	-0.765975	3.420117
H	-3.165208	-1.835148	2.802868
H	0.961970	-3.057666	2.557675
H	0.547820	-1.527866	3.357050
H	0.137620	-3.074480	4.132417
Li	-0.160177	-0.193945	0.955801
O	1.448587	0.156669	0.080759

M_{TPO}

Electronic Energy BS1 = -3016.27015676 Hartree
Electronic Energy BS2 = -3017.03503271 Hartree
Zero-point Energy Correction = 0.967701 Hartree
Thermal Correction to Enthalpy = 1.028911 Hartree
Thermal Correction to Free Energy = 0.868136 Hartree

Chemical symbol X, Y, Z

P	-2.564753	-0.967535	0.136053

C	-3.756839	-0.614940	-1.192571
C	-1.990596	-2.671648	-0.112167
C	-3.468907	-0.936301	1.714343
C	-4.697862	-1.559704	-1.626124
C	-3.705654	0.652182	-1.792047
C	-2.593447	-3.769933	0.519197
C	-0.916928	-2.874043	-0.991511
C	-2.753240	-1.252982	2.884105
C	-4.809241	-0.535544	1.801335
C	-5.590969	-1.233860	-2.647190
H	-4.722569	-2.549801	-1.177996
C	-4.599223	0.969338	-2.817323
H	-2.962916	1.373102	-1.465606
C	-2.120056	-5.059147	0.272294
H	-3.411728	-3.617863	1.217144
C	-0.452223	-4.165221	-1.235768
H	-0.424807	-2.025663	-1.451352
C	-3.385312	-1.165834	4.123869
H	-1.710621	-1.555446	2.820024
C	-5.433587	-0.452877	3.047796
H	-5.364358	-0.287523	0.901751
C	-5.542349	0.030637	-3.242556
H	-6.318272	-1.967432	-2.984237
H	-4.552123	1.949854	-3.283888
C	-1.049655	-5.256818	-0.603276
H	-2.577277	-5.906922	0.775321
H	0.394965	-4.307324	-1.897898
C	-4.722919	-0.766632	4.207656
H	-2.830720	-1.408391	5.026221
H	-6.472757	-0.141525	3.111129
H	-6.236023	0.279341	-4.041466
H	-0.667445	-6.258694	-0.776102
H	-5.209935	-0.699462	5.176990
Cu	2.576269	1.211751	0.406355
B	-0.189069	3.137492	-1.018795
O	-1.547979	3.134748	-1.263592
O	0.217424	4.238819	-0.308493
C	-0.927418	5.127707	-0.185772
C	-2.141257	4.149781	-0.413101
C	-2.600540	3.434694	0.862857
C	-3.335037	4.757287	-1.144450
C	-0.889009	5.796124	1.184312
C	-0.783369	6.176787	-1.295244
H	-0.005413	6.438283	1.257415
H	-0.836800	5.059909	1.987651
H	-1.778803	6.418733	1.333010
H	-0.810625	5.706465	-2.283266
H	0.183396	6.677242	-1.184226
H	-1.574661	6.931955	-1.243423
H	-1.759067	2.933896	1.345058

H	-3.332147	2.665081	0.599665
H	-3.066014	4.128438	1.570784
H	-4.110166	3.994620	-1.275090
H	-3.056088	5.130067	-2.132620
H	-3.764581	5.582405	-0.565126
C	0.756989	2.041031	-1.597649
C	2.185279	2.146744	-1.346156
C	0.138032	1.071015	-2.346867
H	2.522266	3.141378	-1.050301
H	2.850425	1.675587	-2.068542
C	0.622433	-0.150890	-2.970521
H	-0.924026	1.229524	-2.511634
C	1.812213	-0.812206	-2.604565
C	-0.199442	-0.758836	-3.947528
C	2.174046	-2.013642	-3.209726
H	2.405434	-0.427823	-1.792833
C	0.166879	-1.954678	-4.555217
H	-1.139893	-0.282404	-4.214062
C	1.361177	-2.586876	-4.191120
H	3.088668	-2.511764	-2.899545
H	-0.481678	-2.402955	-5.302876
H	1.645847	-3.526291	-4.657584
N	4.320881	0.904728	-0.001030
C	4.731610	-0.327401	-0.649998
C	5.171271	2.002927	-0.424902
C	6.200316	-0.617814	-0.299544
H	4.659408	-0.252566	-1.752575
H	4.089245	-1.140046	-0.304658
C	6.635920	1.673553	-0.085379
H	4.872455	2.923436	0.089061
H	5.105766	2.177524	-1.516151
H	6.287609	-0.792840	0.785457
H	6.555886	-1.505541	-0.832652
O	7.038330	0.458396	-0.697204
H	6.747025	1.607441	1.009991
H	7.304317	2.453536	-0.464265
O	0.377431	-1.479107	2.142625
C	1.256276	-1.846578	1.307845
O	1.780910	-1.059467	0.454234
C	1.658703	-3.297769	1.273536
C	2.537487	-3.765916	0.288694
C	1.102334	-4.203236	2.183981
C	2.858344	-5.120569	0.214899
H	2.933309	-3.058411	-0.430778
C	1.422041	-5.558875	2.112376
H	0.411192	-3.827350	2.930455
C	2.299730	-6.020782	1.127361
H	3.538117	-5.477324	-0.555181
H	0.983519	-6.257562	2.820437
H	2.547190	-7.078015	1.070417

O	1.072154	1.695190	1.442118
C	1.407518	2.187557	2.737734
C	2.122321	3.542776	2.592611
C	2.305018	1.183149	3.490704
C	0.094964	2.363629	3.520299
H	3.088644	3.404731	2.088122
H	1.517436	4.211869	1.975241
H	2.313447	4.010658	3.566420
H	2.519518	1.526271	4.510671
H	1.811627	0.206839	3.536803
H	3.265621	1.053913	2.974247
H	-0.550560	3.095201	3.027358
H	-0.444522	1.410360	3.573254
H	0.285485	2.708318	4.543856
Li	0.110896	0.146679	1.098399
O	-1.443291	0.053974	0.084772

TS-M$_{TPO}$-N$_{TPO}$

Imaginary Freq = -177.8442 (cm^{-1})
Electronic Energy BS1 = -3016.25892062 Hartree
Electronic Energy BS2 = -3017.02475913 Hartree
Zero-point Energy Correction = 0.967798 Hartree
Thermal Correction to Enthalpy = 1.028491 Hartree
Thermal Correction to Free Energy = 0.868552 Hartree

Chemical symbol X, Y, Z

P	2.680449	-0.713287	0.147147
C	3.321886	-1.831355	-1.139207
C	3.395766	0.931330	-0.135262
C	3.335454	-1.275325	1.747767
C	4.602750	-1.707074	-1.694712
C	2.462731	-2.850956	-1.579116
C	4.689852	1.256209	0.302319
C	2.620918	1.890906	-0.799307
C	2.964829	-0.540014	2.888672
C	4.128560	-2.422774	1.879681
C	5.024501	-2.602174	-2.679093
H	5.261567	-0.905023	-1.373610
C	2.889422	-3.739291	-2.567610
H	1.461818	-2.930120	-1.166108
C	5.206534	2.528898	0.060308
H	5.280694	0.526860	0.850486
C	3.139923	3.165057	-1.029139
H	1.605812	1.655656	-1.095313
C	3.388225	-0.962765	4.147591
H	2.342170	0.346047	2.787601
C	4.551748	-2.835428	3.145306
H	4.413568	-2.993342	1.000639
C	4.169070	-3.618076	-3.115598

H	6.016246	-2.501454	-3.111629
H	2.216453	-4.520631	-2.910686
C	4.430243	3.483226	-0.604201
H	6.205416	2.781276	0.406037
H	2.518080	3.912364	-1.510386
C	4.180769	-2.107770	4.277441
H	3.095722	-0.398518	5.028794
H	5.166902	-3.725657	3.245298
H	4.498874	-4.309354	-3.886739
H	4.825018	4.481534	-0.772616
H	4.507605	-2.432820	5.261753
Cu	-2.876249	0.235177	0.522120
B	-1.889600	-2.414895	-1.173751
O	-0.823803	-3.285458	-1.152486
O	-3.054429	-2.978230	-0.714475
C	-2.788824	-4.394957	-0.499612
C	-1.226102	-4.413828	-0.324888
C	-0.775104	-4.079784	1.100550
C	-0.528576	-5.666665	-0.843569
C	-3.578745	-4.863978	0.715870
C	-3.261596	-5.123646	-1.763306
H	-4.651423	-4.816963	0.501469
H	-3.379490	-4.233279	1.582614
H	-3.323733	-5.900886	0.963379
H	-2.691508	-4.798345	-2.639353
H	-4.316001	-4.886787	-1.935504
H	-3.161224	-6.209402	-1.662671
H	-1.231986	-3.143264	1.431517
H	0.309949	-3.934285	1.104655
H	-1.021824	-4.883857	1.802196
H	0.552664	-5.568664	-0.699758
H	-0.717170	-5.823589	-1.908448
H	-0.865524	-6.551891	-0.292448
C	-1.798439	-0.962988	-1.734952
C	-3.020161	-0.177974	-1.749388
C	-0.587711	-0.610404	-2.281268
H	-3.927625	-0.772035	-1.706860
H	-3.072832	0.660910	-2.434789
C	-0.092273	0.615893	-2.881854
H	0.147724	-1.408525	-2.295266
C	-0.641477	1.893439	-2.654641
C	1.056723	0.520976	-3.700031
C	-0.081158	3.022038	-3.248871
H	-1.444372	2.004503	-1.945043
C	1.611215	1.648580	-4.295086
H	1.515035	-0.453207	-3.852294
C	1.039481	2.906232	-4.075728
H	-0.506824	4.000777	-3.045893
H	2.496967	1.552566	-4.916853
H	1.477956	3.791535	-4.528335

N	-4.053490	1.299670	-0.365323
C	-3.701317	2.644707	-0.787423
C	-5.419746	0.972102	-0.724741
C	-4.681472	3.633134	-0.134227
H	-3.777573	2.759470	-1.885182
H	-2.682118	2.864195	-0.462357
C	-6.360794	2.001381	-0.072985
H	-5.667783	-0.032257	-0.366970
H	-5.573748	1.009233	-1.819379
H	-4.543047	3.608216	0.958868
H	-4.496380	4.650291	-0.494143
O	-6.025025	3.319201	-0.473307
H	-6.298624	1.901901	1.023536
H	-7.394569	1.829025	-0.389977
O	0.630438	1.612020	2.099896
C	0.100081	2.354767	1.220423
O	-0.766550	1.935556	0.389262
C	0.564103	3.785125	1.105023
C	0.050581	4.617274	0.102791
C	1.569695	4.268716	1.949880
C	0.540158	5.913279	-0.059484
H	-0.716474	4.224068	-0.554521
C	2.061443	5.563762	1.789763
H	1.964883	3.606509	2.712586
C	1.550402	6.387979	0.782736
H	0.138760	6.552800	-0.842131
H	2.847654	5.930265	2.445009
H	1.936431	7.396406	0.655349
O	-1.764044	-0.755496	1.651780
C	-2.075494	-0.765389	3.044716
C	-3.279061	-1.690440	3.286582
C	-2.400994	0.653942	3.552587
C	-0.838594	-1.294292	3.795170
H	-4.125919	-1.358012	2.672657
H	-3.034534	-2.716654	2.995925
H	-3.588559	-1.692977	4.339302
H	-2.581169	0.658108	4.635221
H	-1.567727	1.326555	3.330009
H	-3.302118	1.043151	3.059526
H	-0.562171	-2.288074	3.428485
H	0.012046	-0.621719	3.639595
H	-1.026342	-1.363664	4.873839
Li	-0.165621	0.083579	1.111911
O	1.166111	-0.725519	0.098236

N_{TPO}

Electronic Energy BS1 = -3016.32480400 Hartree
Electronic Energy BS2 = -3017.08794316 Hartree
Zero-point Energy Correction = 0.971410 Hartree

Thermal Correction to Enthalpy = 1.032026 Hartree
Thermal Correction to Free Energy = 0.872075 Hartree

**Chemical symbol X, Y, Z**			
P	2.068748	-1.749737	0.184478
C	2.109331	-3.121837	-1.011988
C	3.550029	-0.735414	-0.091043
C	2.246429	-2.483654	1.840516
C	3.279496	-3.836367	-1.305317
C	0.902950	-3.462814	-1.640640
C	4.801469	-1.056599	0.455646
C	3.409881	0.404316	-0.893631
C	2.534129	-1.635089	2.924479
C	2.001038	-3.845855	2.067193
C	3.240657	-4.890967	-2.217395
H	4.219732	-3.562792	-0.833877
C	0.872042	-4.517449	-2.555681
H	0.001851	-2.899765	-1.420784
C	5.905282	-0.245456	0.188044
H	4.909332	-1.920112	1.106382
C	4.514631	1.213762	-1.154105
H	2.436277	0.667723	-1.288544
C	2.579406	-2.155359	4.217410
H	2.687026	-0.572846	2.757779
C	2.045956	-4.357343	3.365055
H	1.775480	-4.505020	1.234200
C	2.036807	-5.232770	-2.842078
H	4.148924	-5.441921	-2.446048
H	-0.064074	-4.771870	-3.045553
C	5.761858	0.888090	-0.617186
H	6.872713	-0.491377	0.617702
H	4.387288	2.105169	-1.759545
C	2.336010	-3.513546	4.439305
H	2.794407	-1.495355	5.053161
H	1.854126	-5.413148	3.536135
H	2.011239	-6.052584	-3.555258
H	6.620477	1.524757	-0.813892
H	2.368535	-3.913201	5.449457
Cu	-2.389266	1.054809	0.651840
B	-2.669157	-0.896446	-1.642224
O	-2.152202	-2.171073	-1.646362
O	-3.975237	-0.856719	-1.205127
C	-4.413746	-2.227719	-0.978235
C	-3.048263	-3.008790	-0.861921
C	-2.480076	-3.050759	0.561170
C	-3.053969	-4.405903	-1.477392
C	-5.271635	-2.245678	0.281769
C	-5.240604	-2.623416	-2.206269
H	-6.193543	-1.679674	0.109858
H	-4.742318	-1.789343	1.119478
H	-5.546639	-3.271933	0.550091

H	-4.631935	-2.589685	-3.115328
H	-6.065576	-1.913671	-2.323213
H	-5.660460	-3.629158	-2.101702
H	-2.384072	-2.049628	0.993805
H	-1.474487	-3.482259	0.525574
H	-3.097779	-3.676143	1.215564
H	-2.060652	-4.853481	-1.369545
H	-3.301947	-4.381252	-2.541287
H	-3.774477	-5.051523	-0.962718
C	-1.867490	0.357122	-2.089381
C	-2.542930	1.711422	-2.137571
C	-0.602308	0.137939	-2.522970
H	-3.410474	1.656390	-2.806988
H	-1.868950	2.462376	-2.552223
C	0.418634	1.076986	-3.020980
H	-0.273927	-0.898310	-2.535400
C	0.723497	2.288277	-2.374584
C	1.188017	0.699636	-4.137821
C	1.745524	3.109549	-2.855099
H	0.237364	2.525639	-1.435338
C	2.196633	1.528045	-4.625211
H	0.987490	-0.253028	-4.622625
C	2.475099	2.741096	-3.987831
H	1.990285	4.020853	-2.317076
H	2.774997	1.221930	-5.493012
H	3.270775	3.382265	-4.358575
N	-3.045502	2.225501	-0.798403
C	-2.540594	3.612537	-0.559116
C	-4.539004	2.263819	-0.782375
C	-3.118659	4.178092	0.734094
H	-2.831524	4.253835	-1.405049
H	-1.454104	3.564631	-0.477456
C	-5.046522	2.866665	0.523930
H	-4.900327	1.241947	-0.907642
H	-4.891223	2.882434	-1.622520
H	-2.738231	3.599552	1.591176
H	-2.804709	5.218784	0.855436
O	-4.541298	4.177988	0.724193
H	-4.767353	2.213298	1.368434
H	-6.137218	2.948636	0.495945
O	1.546366	1.255263	2.024309
C	1.194291	2.228899	1.293805
O	0.068124	2.285938	0.705755
C	2.174870	3.350775	1.060153
C	1.761915	4.541856	0.449962
C	3.521290	3.188504	1.406031
C	2.681458	5.557491	0.186886
H	0.715688	4.654651	0.184307
C	4.445449	4.196821	1.131745
H	3.827729	2.256824	1.868489

C	4.028145	5.383282	0.522344
H	2.351794	6.482579	-0.279907
H	5.492238	4.055583	1.388969
H	4.747725	6.170480	0.311046
O	-1.679424	-0.118696	1.893765
C	-2.011876	-0.082956	3.272827
C	-3.504877	-0.405221	3.454766
C	-1.697054	1.303535	3.863644
C	-1.158642	-1.155868	3.972553
H	-4.108636	0.312285	2.883747
H	-3.722850	-1.409969	3.076924
H	-3.810419	-0.357199	4.507500
H	-1.890396	1.341369	4.943383
H	-0.647756	1.556851	3.679607
H	-2.318448	2.064792	3.374601
H	-1.337490	-2.134218	3.512744
H	-0.094145	-0.923317	3.864800
H	-1.389113	-1.223170	5.043173
Li	0.052058	0.237712	1.246077
O	0.785361	-0.962903	-0.001812

O_{TPO}

Electronic Energy BS1 = -2595.41207069 Hartree
Electronic Energy BS2 = -2596.05908150 Hartree
Zero-point Energy Correction = 0.849141 Hartree
Thermal Correction to Enthalpy = 0.902325 Hartree
Thermal Correction to Free Energy = 0.759787 Hartree

Chemical symbol X, Y, Z

Cu	-1.582334	-1.178738	-1.447468
B	-1.727408	2.304893	-0.905210
O	-1.874662	3.285649	0.063097
O	-0.510143	2.404942	-1.552451
C	0.078468	3.679052	-1.191594
C	-0.613852	3.985052	0.187161
C	0.128058	3.364870	1.378364
C	-0.899216	5.460684	0.453906
C	1.596511	3.539423	-1.128992
C	-0.313148	4.666987	-2.298658
H	1.987095	3.271734	-2.114947
H	1.902357	2.759119	-0.432227
H	2.054533	4.486065	-0.818905
H	-1.400428	4.778797	-2.355903
H	0.037108	4.273296	-3.257773
H	0.134565	5.653891	-2.140688
H	0.340314	2.305653	1.201325
H	-0.503767	3.443182	2.268408
H	1.069533	3.889073	1.576368
H	-1.390956	5.571048	1.425928

H	-1.560000	5.882642	-0.306325
H	0.031897	6.039008	0.472643
C	-2.797697	1.221617	-1.223503
C	-2.495778	0.284143	-2.331707
C	-3.895607	1.238770	-0.400081
H	-1.804049	0.739187	-3.051749
H	-3.382025	-0.071087	-2.870984
C	-5.022349	0.320865	-0.290780
H	-3.934897	2.046139	0.329832
C	-5.093824	-0.952120	-0.905396
C	-6.090401	0.678084	0.567388
C	-6.164526	-1.813439	-0.658230
H	-4.291416	-1.284188	-1.549739
C	-7.162637	-0.175450	0.800262
H	-6.049263	1.636970	1.077455
C	-7.207784	-1.435014	0.189486
H	-6.180596	-2.790025	-1.137314
H	-7.961112	0.134062	1.470383
H	-8.042467	-2.106763	0.372512
O	-0.602205	-2.362940	-0.324511
C	-0.324866	-3.690779	-0.721507
C	-1.648183	-4.443842	-0.952466
C	0.470325	-4.373830	0.404534
C	0.503848	-3.682763	-2.019222
H	-2.245678	-4.439964	-0.033117
H	-2.229483	-3.937927	-1.733039
H	-1.482353	-5.485299	-1.256529
H	0.700617	-5.419572	0.166121
H	1.414377	-3.848133	0.575862
H	-0.110690	-4.353272	1.335180
H	-0.066571	-3.192084	-2.817287
H	1.425303	-3.112389	-1.865472
H	0.768812	-4.696390	-2.346865
Li	-0.099922	-1.111484	0.890719
N	-1.742078	-0.554450	1.930250
C	-1.917705	0.698908	2.675935
C	-2.726597	-1.369082	1.898889
C	-3.015471	0.598567	3.729032
H	-2.153376	1.479300	1.943767
H	-0.957400	0.958713	3.136312
C	-4.044250	-1.180193	2.600334
H	-2.589861	-2.282413	1.316163
H	-2.692970	-0.065616	4.550772
H	-3.248175	1.580114	4.152456
O	-4.216928	0.113095	3.144354
H	-4.121228	-1.956364	3.387510
H	-4.862130	-1.348255	1.894597
O	1.487430	-0.150763	1.085996
P	2.861211	-0.274053	0.439056
C	3.524057	-1.965036	0.512550

C	2.854293	0.240623	-1.300832
C	4.066849	0.765047	1.317375
C	3.886494	-2.679394	-0.636627
C	3.623144	-2.576790	1.773778
C	4.001620	0.734804	-1.943344
C	1.647244	0.146659	-2.004210
C	3.587565	1.872528	2.030766
C	5.444855	0.504803	1.276038
C	4.335136	-3.997320	-0.525942
H	3.807442	-2.213733	-1.613791
C	4.072359	-3.891121	1.878876
H	3.340332	-2.026422	2.667282
C	3.933900	1.119497	-3.281501
H	4.936581	0.836896	-1.400171
C	1.582592	0.539292	-3.340578
H	0.747634	-0.210761	-1.516493
C	4.481908	2.721884	2.682710
H	2.519336	2.051731	2.080637
C	6.335579	1.359669	1.926245
H	5.819385	-0.369525	0.750282
C	4.425813	-4.602824	0.727835
H	4.603003	-4.551287	-1.420926
H	4.141039	-4.363174	2.854752
C	2.724907	1.021255	-3.980576
H	4.820512	1.505871	-3.776696
H	0.630416	0.480585	-3.858125
C	5.854839	2.469379	2.626547
H	4.106645	3.577478	3.237317
H	7.402084	1.155231	1.893828
H	4.768335	-5.630534	0.810549
H	2.675652	1.332280	-5.020757
H	6.550081	3.131769	3.135009

TS-O$_{TPO}$-P$_{TPO}$

Imaginary Freq = -380.5053 (cm^{-1})
Electronic Energy BS1 = -2595.37640427 Hartree
Electronic Energy BS2 = -2596.02009008 Hartree
Zero-point Energy Correction = 0.849121 Hartree
Thermal Correction to Enthalpy = 0.900980 Hartree
Thermal Correction to Free Energy = 0.762692 Hartree

Chemical symbol X, Y, Z

Cu	-1.717786	0.933099	1.423282
B	-1.795550	-1.932848	0.628040
O	-1.902864	-3.056699	-0.146140
O	-0.644746	-1.907750	1.386635
C	-0.072872	-3.245716	1.335443
C	-0.657452	-3.803418	-0.014666
C	0.218276	-3.462740	-1.224662

C	-0.996511	-5.291053	-0.005416
C	1.445605	-3.140959	1.384260
C	-0.594205	-3.980926	2.576510
H	1.758046	-2.701291	2.335378
H	1.823910	-2.505488	0.584821
H	1.901434	-4.133844	1.293907
H	-1.686346	-4.054522	2.561435
H	-0.303401	-3.415219	3.467162
H	-0.176712	-4.990249	2.652807
H	0.458068	-2.396525	-1.248712
H	-0.322739	-3.711290	-2.141193
H	1.149019	-4.040734	-1.203904
H	-1.410059	-5.577883	-0.977349
H	-1.738218	-5.529712	0.759972
H	-0.096992	-5.890692	0.175497
C	-2.964170	-0.890006	0.826290
C	-3.072937	-0.364064	2.131869
C	-3.835376	-0.624425	-0.302182
H	-2.507036	-0.851698	2.927710
H	-3.985662	0.139786	2.449779
C	-5.145875	0.040341	-0.083967
H	-3.908185	-1.497144	-0.955201
C	-5.249923	1.314063	0.509814
C	-6.332326	-0.558385	-0.547392
C	-6.485282	1.947766	0.651839
H	-4.350562	1.822422	0.846406
C	-7.567188	0.072487	-0.406129
H	-6.274247	-1.530297	-1.032098
C	-7.652670	1.329820	0.199350
H	-6.533219	2.931320	1.113755
H	-8.466374	-0.418006	-0.771337
H	-8.614941	1.822519	0.310849
O	-0.421367	1.951140	0.531864
C	-0.404473	3.370961	0.588132
C	-1.837727	3.927070	0.662386
C	0.279651	3.892572	-0.686668
C	0.389284	3.808395	1.831329
H	-2.424421	3.564726	-0.188540
H	-2.331733	3.590020	1.584157
H	-1.849672	5.024206	0.654121
H	0.332149	4.987966	-0.698232
H	1.298561	3.502958	-0.757523
H	-0.279948	3.566543	-1.572206
H	-0.078441	3.389247	2.731093
H	1.416273	3.432873	1.774278
H	0.424583	4.900334	1.932573
Li	-0.061939	0.719259	-0.892635
N	-1.576466	-0.035828	-1.801033
C	-1.556713	-1.052764	-2.847102
C	-2.816255	0.463700	-1.539757

C	-2.413340	-0.685922	-4.054828
H	-1.930998	-2.016840	-2.463812
H	-0.518532	-1.216376	-3.167113
C	-3.769056	0.663101	-2.723011
H	-2.793778	1.364797	-0.916334
H	-1.999023	0.202662	-4.565032
H	-2.471836	-1.509031	-4.776765
O	-3.749625	-0.421917	-3.634604
H	-3.445241	1.589161	-3.236902
H	-4.805691	0.794138	-2.410833
O	1.669261	-0.026229	-1.029614
P	3.009592	0.246982	-0.366964
C	3.570727	1.957222	-0.621929
C	2.987784	-0.053574	1.425586
C	4.290412	-0.821429	-1.088639
C	3.924192	2.802490	0.437967
C	3.586090	2.446959	-1.938741
C	4.008854	-0.746579	2.092308
C	1.865224	0.400157	2.136760
C	3.879600	-2.001595	-1.724269
C	5.655482	-0.508657	-1.008066
C	4.281717	4.128535	0.183497
H	3.904789	2.432772	1.458316
C	3.944848	3.769845	-2.187451
H	3.300356	1.796982	-2.761375
C	3.904024	-0.987630	3.462975
H	4.872340	-1.113591	1.546204
C	1.767527	0.150550	3.505220
H	1.070791	0.939736	1.625089
C	4.830981	-2.868401	-2.263392
H	2.820150	-2.222620	-1.803833
C	6.602741	-1.380652	-1.545472
H	5.975389	0.417073	-0.536591
C	4.289896	4.612132	-1.125505
H	4.543793	4.783598	1.009423
H	3.947345	4.147192	-3.206025
C	2.782410	-0.543316	4.169175
H	4.692736	-1.530502	3.976605
H	0.889399	0.493520	4.045626
C	6.190605	-2.560994	-2.170722
H	4.510481	-3.780234	-2.759597
H	7.659539	-1.136245	-1.483989
H	4.561036	5.646071	-1.320269
H	2.699990	-0.741343	5.234681
H	6.929826	-3.236712	-2.592273

$\mathbf{P_{TPO}}$

Electronic Energy BS1 = -2595.42462371 Hartree
Electronic Energy BS2 = -2596.06463119 Hartree

Zero-point Energy Correction = 0.852284 Hartree
Thermal Correction to Enthalpy = 0.903420 Hartree
Thermal Correction to Free Energy = 0.766979 Hartree

Chemical symbol X, Y, Z

Cu	-2.013019	0.625074	1.553983
B	-1.814745	-1.641765	-0.033465
O	-1.976878	-3.073789	-0.223050
O	-0.527265	-1.503872	0.725156
C	-0.168990	-2.790515	1.257720
C	-0.813453	-3.772405	0.212192
C	0.122920	-4.024267	-0.984950
C	-1.247794	-5.121642	0.791939
C	1.350432	-2.909680	1.376237
C	-0.783381	-2.939171	2.657756
H	1.721330	-2.287422	2.193514
H	1.849956	-2.593839	0.459261
H	1.632684	-3.948057	1.587281
H	-1.872439	-2.991955	2.606734
H	-0.498968	-2.068753	3.258976
H	-0.420148	-3.842030	3.161067
H	0.548194	-3.088234	-1.354701
H	-0.453574	-4.480553	-1.795392
H	0.946751	-4.697217	-0.720595
H	-1.656544	-5.747596	-0.008576
H	-2.027702	-4.994925	1.546103
H	-0.400623	-5.653061	1.242528
C	-3.148225	-0.958022	0.682513
C	-3.478958	-0.769536	2.007311
C	-3.999376	-0.511582	-0.495391
H	-2.932672	-1.279743	2.799636
H	-4.442721	-0.348795	2.296048
C	-5.239029	0.319131	-0.268237
H	-4.291803	-1.442339	-1.006160
C	-5.201650	1.544562	0.415857
C	-6.469649	-0.101740	-0.793149
C	-6.350846	2.320342	0.570840
H	-4.264953	1.891875	0.839662
C	-7.623597	0.669047	-0.640111
H	-6.519403	-1.044038	-1.334116
C	-7.568779	1.885289	0.043240
H	-6.293225	3.265371	1.105372
H	-8.564576	0.319084	-1.056891
H	-8.465152	2.487851	0.163890
O	-0.587081	1.581080	0.812515
C	-0.545805	2.976315	0.538769
C	-1.849020	3.657567	0.984634
C	-0.353705	3.172087	-0.976572
C	0.641074	3.595091	1.298702
H	-2.696675	3.258510	0.417707
H	-2.025168	3.466152	2.051370

H	-1.816571	4.743099	0.829187
H	-0.258163	4.232831	-1.239924
H	0.550780	2.652095	-1.306082
H	-1.206794	2.756388	-1.524864
H	0.534388	3.407183	2.373571
H	1.585185	3.153080	0.966415
H	0.701319	4.678578	1.138323
Li	-0.032147	0.083266	-0.383158
N	-1.730815	-0.755534	-1.355549
C	-1.528761	-1.562924	-2.576339
C	-2.908014	0.151484	-1.393598
C	-1.914814	-0.837929	-3.853106
H	-2.104775	-2.496787	-2.530631
H	-0.471529	-1.842570	-2.641266
C	-3.358042	0.602191	-2.788714
H	-2.635069	1.084200	-0.880570
H	-1.253161	0.028408	-4.036098
H	-1.842510	-1.515783	-4.710045
O	-3.266696	-0.403536	-3.779561
H	-2.741268	1.466248	-3.096178
H	-4.401320	0.929989	-2.752653
O	1.730910	0.129802	-1.015592
P	3.089883	0.339244	-0.371962
C	3.684732	2.041566	-0.610783
C	3.119150	0.028449	1.421706
C	4.319297	-0.764864	-1.131763
C	4.467154	2.704273	0.345364
C	3.323100	2.700502	-1.795577
C	4.196717	-0.616785	2.047020
C	2.011154	0.438132	2.182161
C	3.854522	-1.958761	-1.702436
C	5.690584	-0.471495	-1.144135
C	4.886910	4.015486	0.114376
H	4.729457	2.206282	1.274397
C	3.740537	4.011677	-2.018869
H	2.701085	2.189355	-2.524313
C	4.168959	-0.847082	3.423502
H	5.046948	-0.957541	1.464234
C	1.993605	0.202550	3.556892
H	1.148607	0.907508	1.712657
C	4.759179	-2.858795	-2.266026
H	2.789236	-2.166901	-1.713247
C	6.591344	-1.376272	-1.707322
H	6.051581	0.464670	-0.726306
C	4.522673	4.668945	-1.064979
H	5.486990	4.529441	0.860046
H	3.450069	4.522842	-2.932333
C	3.067878	-0.437850	4.179681
H	5.002141	-1.354698	3.901681
H	1.128102	0.512224	4.136667

C	6.126250	-2.570434	-2.264905
H	4.396062	-3.782113	-2.708524
H	7.653158	-1.146383	-1.717967
H	4.842696	5.692772	-1.238327
H	3.045086	-0.625616	5.249918
H	6.829265	-3.272234	-2.705346

14.9.5. Cartesian coordinates for the imine formation (Figure S23)

Y

Electronic Energy BS1 = -1413.06574135 Hartree
Electronic Energy BS2 = -1413.48186270 Hartree
Zero-point Energy Correction = 0.598445 Hartree
Thermal Correction to Enthalpy = 0.633327 Hartree
Thermal Correction to Free Energy = 0.529238 Hartree

Chemical symbol X, Y, Z

N	-0.098162	1.248988	-0.199225
C	0.176923	2.259404	0.841378
C	0.434633	1.711455	-1.503358
C	-0.204644	3.680243	0.428853
H	1.253897	2.166180	0.995924
H	-0.344010	1.959264	1.754794
C	0.034070	3.144890	-1.839562
H	0.101138	1.008533	-2.271221
H	1.518672	1.613566	-1.368746
H	-1.300523	3.777684	0.346695
H	0.149553	4.394606	1.178527
O	0.413309	4.039853	-0.799974
H	-1.052688	3.208907	-2.018484
H	0.552246	3.478315	-2.743901
O	-1.567386	1.213118	-0.287828
C	-2.046048	0.021930	-0.719662
O	-1.349062	-0.870850	-1.161526
C	-3.527422	-0.054797	-0.583158
C	-4.286822	0.979583	-0.016942
C	-4.159895	-1.225740	-1.024880
O	2.660498	0.415263	0.227827
C	-5.667788	0.837890	0.105607
H	-3.794656	1.883528	0.323740
C	-5.539783	-1.362703	-0.898411
H	-3.552676	-2.012950	-1.459602
C	3.909112	0.764436	0.699844
C	-6.294956	-0.331140	-0.333190
H	-6.255308	1.639189	0.544675
H	-6.027154	-2.271395	-1.240063
C	3.832271	2.064787	1.540628
C	4.878800	1.012930	-0.481350

C	4.493509	-0.354138	1.596859
H	-7.371819	-0.438252	-0.234761
H	3.434687	2.882457	0.925963
H	3.156111	1.914675	2.392905
H	4.809431	2.377523	1.932838
H	5.880124	1.323842	-0.153279
H	4.983413	0.099561	-1.078591
H	4.469128	1.795228	-1.131961
H	3.801794	-0.558367	2.424018
H	4.607603	-1.277785	1.017100
H	5.474284	-0.095716	2.019452
Li	1.207834	-0.568488	0.224619
O	1.337527	-2.267820	-0.906379
C	2.544232	-2.960364	-0.510934
C	1.422309	-2.036313	-2.321821
C	3.604269	-2.667840	-1.600006
H	2.819463	-2.576868	0.473093
H	2.324830	-4.033390	-0.430334
C	2.889293	-1.678976	-2.544216
H	1.135951	-2.951475	-2.864746
H	0.705752	-1.249707	-2.560980
H	3.878032	-3.588118	-2.127904
H	4.514988	-2.238827	-1.175033
H	3.204238	-1.775412	-3.588186
H	3.059828	-0.655654	-2.198464
O	0.377453	-1.518225	1.764436
C	-0.640362	-2.522217	1.556751
C	0.027185	-0.792038	2.947785
C	-1.925073	-1.989128	2.239968
H	-0.730181	-2.655408	0.479484
H	-0.303802	-3.463184	2.011457
C	-1.490764	-0.633761	2.845786
H	0.310394	-1.371777	3.840191
H	0.596777	0.140063	2.938183
H	-2.260514	-2.677397	3.022491
H	-2.746637	-1.870837	1.528657
H	-1.964155	-0.422803	3.809432
H	-1.731570	0.183691	2.159095

TS_{Y-Z}

Imaginary Freq = -1034.3316 (cm^{-1})
Electronic Energy BS1 = -1413.04237916 Hartree
Electronic Energy BS2 = -1413.45607722 Hartree
Zero-point Energy Correction = 0.592387 Hartree
Thermal Correction to Enthalpy = 0.627046 Hartree
Thermal Correction to Free Energy = 0.524487 Hartree

Chemical symbol X, Y, Z

N	0.077375	1.532081	0.010855

C	0.158218	2.710770	0.878866
C	0.764901	1.668592	-1.195415
C	0.089296	4.035960	0.126184
H	1.131464	2.624980	1.374339
H	-0.623107	2.629550	1.638077
C	0.829722	3.038783	-1.849841
H	0.472444	0.879152	-1.895595
H	1.843590	1.134325	-0.760795
H	-0.917201	4.171601	-0.302064
H	0.299652	4.873906	0.798642
O	1.076288	4.069036	-0.895250
H	-0.107572	3.259105	-2.393674
H	1.655191	3.075982	-2.567850
O	-1.667582	1.499407	-0.278326
C	-1.993446	0.248093	-0.478542
O	-1.197109	-0.698700	-0.543879
C	-3.471537	0.013449	-0.609549
C	-4.394002	1.063780	-0.512303
C	-3.927736	-1.295320	-0.816653
O	2.560175	0.114603	-0.151903
C	-5.759444	0.804924	-0.621009
H	-4.031228	2.073174	-0.351363
C	-5.293355	-1.551443	-0.922853
H	-3.196785	-2.093867	-0.890432
C	3.830017	0.505212	0.301368
C	-6.210870	-0.501703	-0.825407
H	-6.472407	1.621501	-0.545383
H	-5.643167	-2.567829	-1.082462
C	3.739186	1.835837	1.080686
C	4.766713	0.687298	-0.909973
C	4.393294	-0.580939	1.236535
H	-7.275992	-0.701471	-0.908744
H	3.304433	2.622084	0.451739
H	3.100220	1.704064	1.963216
H	4.723175	2.179123	1.424074
H	5.783632	0.973017	-0.611914
H	4.821029	-0.246515	-1.481059
H	4.371681	1.466093	-1.573492
H	3.688533	-0.766226	2.055697
H	4.536682	-1.519392	0.690687
H	5.359716	-0.287747	1.666242
Li	0.837590	-0.650565	0.250702
O	0.991700	-2.532609	-0.610478
C	2.218807	-3.220639	-0.290698
C	0.883196	-2.497091	-2.042795
C	3.136054	-3.102249	-1.532684
H	2.626580	-2.739787	0.599412
H	1.987017	-4.268610	-0.056834
C	2.313699	-2.237874	-2.511733
H	0.502977	-3.464117	-2.409868

H	0.160302	-1.717822	-2.289756
H	3.340939	-4.092087	-1.955162
H	4.094327	-2.636505	-1.289453
H	2.476000	-2.502772	-3.561348
H	2.554672	-1.183289	-2.354943
O	0.404451	-1.264640	2.085181
C	-0.656964	-2.241031	2.165631
C	0.127856	-0.269410	3.079058
C	-1.913505	-1.481332	2.654077
H	-0.756463	-2.672461	1.170051
H	-0.357930	-3.023296	2.876665
C	-1.388048	-0.066305	3.006122
H	0.438946	-0.640734	4.067845
H	0.719408	0.614418	2.829959
H	-2.350266	-1.975430	3.527679
H	-2.679682	-1.436209	1.876620
H	-1.802941	0.323141	3.940827
H	-1.627985	0.637132	2.205117

Z

Electronic Energy BS1 = -1413.15576871 Hartree
Electronic Energy BS2 = -1413.57813107 Hartree
Zero-point Energy Correction = 0.597767 Hartree
Thermal Correction to Enthalpy = 0.633663 Hartree
Thermal Correction to Free Energy = 0.527183 Hartree

Chemical symbol X, Y, Z

N	0.032762	2.583169	0.130520
C	-0.754267	3.094125	1.261372
C	-0.538977	2.485776	-1.002266
C	-2.256813	3.068862	1.002862
H	-0.406751	4.114106	1.471554
H	-0.520924	2.465028	2.124781
C	-1.960824	2.884897	-1.304686
H	0.028771	2.044690	-1.823690
H	1.443771	1.552183	0.365742
H	-2.617330	2.033525	1.046943
H	-2.796846	3.664106	1.744729
O	-2.547868	3.648637	-0.272079
H	-2.534424	1.961772	-1.508635
H	-1.986692	3.496644	-2.214986
O	-1.107576	-0.058974	1.417804
C	-1.411095	-0.399248	0.251455
O	-0.582225	-0.591501	-0.704954
C	-2.878818	-0.607864	-0.078092
C	-3.843618	-0.499548	0.931942
C	-3.288460	-0.905051	-1.384472
O	2.163457	0.855484	0.402695
C	-5.195589	-0.679992	0.641696

H	-3.506038	-0.271577	1.938078
C	-4.641143	-1.080926	-1.678249
H	-2.529610	-0.995767	-2.154831
C	3.062972	1.159302	1.489039
C	-5.597505	-0.968640	-0.665442
H	-5.937087	-0.594706	1.432268
H	-4.951013	-1.307684	-2.695568
C	2.280335	1.215907	2.807643
C	3.738390	2.505905	1.196977
C	4.089635	0.025583	1.523431
H	-6.651522	-1.106275	-0.893726
H	1.537531	2.019165	2.782901
H	1.752002	0.274014	2.977447
H	2.953852	1.401204	3.652417
H	4.452117	2.770351	1.985686
H	4.274068	2.461128	0.242413
H	2.988544	3.302247	1.130079
H	3.585433	-0.939990	1.636996
H	4.671637	0.007458	0.595882
H	4.785055	0.160695	2.358839
Li	1.201255	-0.753447	-0.111364
O	2.089427	-1.279547	-1.866733
C	3.522028	-1.264545	-1.968752
C	1.607123	-0.463563	-2.949029
C	3.903720	0.143351	-2.481982
H	3.919639	-1.505307	-0.981133
H	3.836227	-2.044986	-2.676881
C	2.554558	0.742616	-2.959351
H	1.672339	-1.034169	-3.888484
H	0.562805	-0.237794	-2.730181
H	4.637028	0.078286	-3.291785
H	4.334440	0.752701	-1.683970
H	2.619355	1.213927	-3.944754
H	2.211711	1.488110	-2.237243
O	1.483552	-2.413447	0.956292
C	1.200590	-3.611712	0.206629
C	0.711035	-2.503106	2.180496
C	-0.301917	-3.823099	0.393226
H	1.508529	-3.426759	-0.823739
H	1.786271	-4.445866	0.623529
C	-0.537881	-3.353252	1.850031
H	1.336166	-2.973436	2.951275
H	0.453544	-1.484506	2.468829
H	-0.604220	-4.861172	0.223738
H	-0.843530	-3.178979	-0.304313
H	-0.622945	-4.201695	2.536798
H	-1.446015	-2.752406	1.932382

15. Microkinetic simulations

Microkinetic simulations were performed using COPASI software.[18]

15.1 Thermal rate constants k (298 K) for steps involving the most significant transitions states for microkinetic simulations

The thermal rate coefficient, $k_i(T)$, for each process i presenting a barrier is calculated according to transition state theory:

$$k_i(T) = \sigma \frac{k_B T}{h} \left(\frac{RT}{P_0}\right)^{\Delta n} e^{-\frac{\Delta G_i^{\ddagger}}{RT}}$$

Where σ is the reaction path degeneracy, T is the temperature (298 K), h is Planck's constant, $\Delta G^{\ddagger}_i$ is the free energy of activation, p_0 is 1 bar and Δn = 1 (0) for bimolecular (unimolecular) reactions.

Table S7. Thermal rate constants k (298 K) used for microkinetic simulations of reaction with Cu(dcpe)(Bpin) (A) as catalyst.

Step	ΔG‡	*k* (298 K)
**A + 1 → C**	9.5 kcal mol^{-1}	1.6E+07 M^{-1} s^{-1}
**C + 2 → 4 + A**	28.0 kcal mol^{-1}	3.8E-07 M^{-1} s^{-1}
**C + Imina → 3 + A**	16.3 kcal mol^{-1}	1.5E+02 M^{-1} s^{-1}

Table S8. Thermal rate constants k (298 K) used for microkinetic simulations of reaction with Cu (O^{t}Bu)(Bpin)Li(THF)$_2$ (I) as catalyst.

Step	ΔG‡	*k* (298 K)
**I + 1 → K**	15.2 kcal mol^{-1}	1.0E+03 M^{-1} s^{-1}
**K + 2 → 4 + I**[a]	10.1 kcal mol^{-1}	5.7E+06 M^{-1} s^{-1}
**K + Imina → 3 + I**[b]	24.7 kcal mol^{-1}	1.0E-04 M^{-1} s^{-1}

[a] We assumed pre-equilibrium conditions between **K** and **L** before transformation into products and recovery of catalyst.[19]

[b] We assumed pre-equilibrium conditions between **K** and **O** before transformation into products and recovery of catalyst.[19]

Table S9. Thermal rate constants k (298 K) used for microkinetic simulations of reaction with Cu(^{t}BuO)(Bpin)Li(THF)(OPPh$_3$) (I$_{TPO}$)as catalyst.

Step	ΔG‡	*k* (298 K)
**I$_{TPO}$ + 1 → K$_{TPO}$**	16.3 kcal mol^{-1}	1.5E+02 M^{-1} s^{-1}
**K$_{TPO}$ + 2 → 4 + I$_{TPO}$**[a]	14.3 kcal mol^{-1}	4.6E+03 M^{-1} s^{-1}
**K$_{TPO}$ + Imina → 3 + I$_{TPO}$**[b]	27.0 kcal mol^{-1}	2.1E-06 M^{-1} s^{-1}

[a] We assumed pre-equilibrium conditions between **K$_{TPO}$** and **L$_{TPO}$** before transformation into products and recovery of catalyst.[19]

[b] We assumed pre-equilibrium conditions between **K$_{TPO}$** and **O$_{TPO}$** before transformation into products and recovery of catalyst.[19]

---

[18] a) COPASI 4.36, (2022) Build 260. http://copasi.org/; b) Hoops, S.; Sahle, S.; Gauges, R.; Lee, C.; Pahle, J.; Simus, N.; Singhal, M.; Xu, L.; Mendes, P.; Kummer, U. *Bioinformatics* **2006**, *22*, 3067-3074.

[19] Atkins, P.; De Paula, J. *Atkins' Physical Chemistry*; Oxford, 2006, p 815.

Table S10. Thermal rate constants k (298 K) used for microkinetic simulations of formation of the imine derived from morpholino benzoate 2.

Step	ΔG‡	*k* (298 K)
**^{t}BuOLi(THF)$_2$ + 2 → Imine + Li·^{t}BuOH·OBz·(THF)$_2$**	16.6 kcal mol^{-1}	9.3E+01 M^{-1} s^{-1}

15.2 Microkinetic simulation for the reaction in the absence of dcpe ligand (conditions: Table 1, entry 14)

Initial concentrations:

I	I$_{TPO}$	A	1	2	^{t}BuOLi	Volume
0.005 M	0.0 M	0.0 M	0.1 M	0.15 M	0.2 M	3 mL

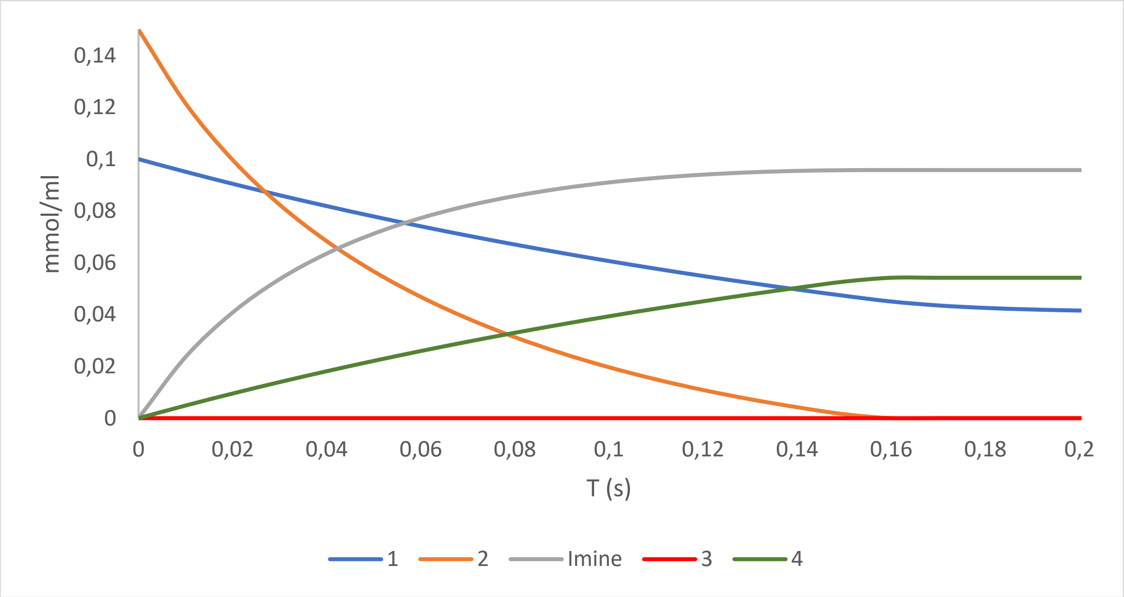

<u>*Observation:*</u> According to the experimental result, only C-N coupling product **4** is obtained under these conditions.

15.3 Microkinetic simulation for the reaction in the absence of dcpe ligand and in the presence of 6 mol% of P(O)Ph$_3$ (conditions: Table 1, entry 15)

Initial concentrations:

I	I$_{TPO}$	A	1	2	^{t}BuOLi	Volume
0.0 M	0.005 M	0.0 M	0.1 M	0.15 M	0.2 M	3 mL

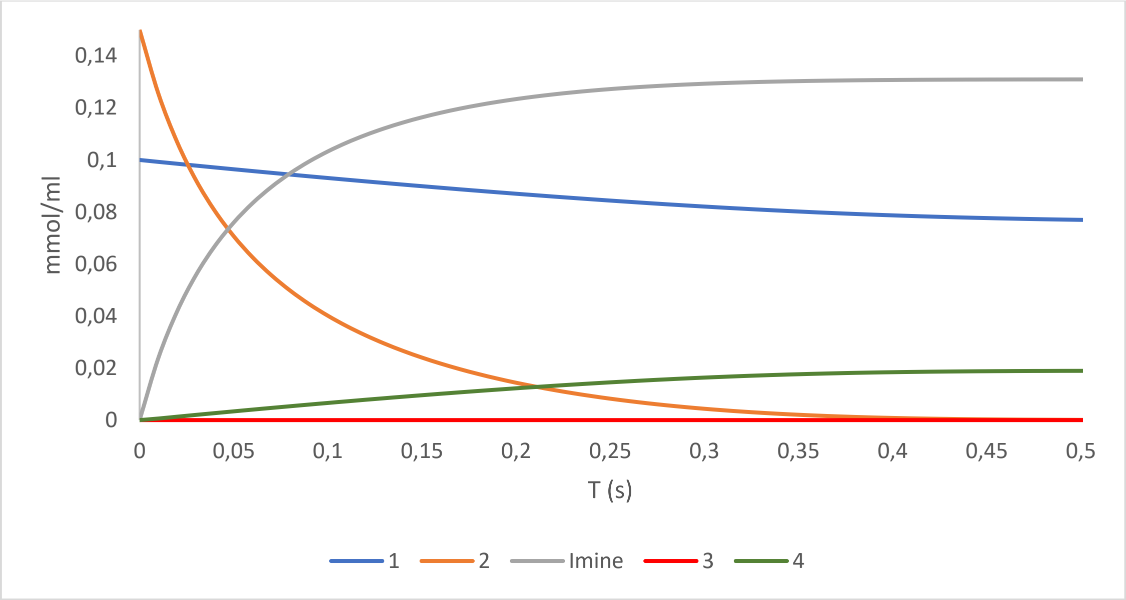

Observation: According to the experimental result, only C-N coupling product **4** is obtained under these conditions albeit in a lower amount than in the absence of $P(O)Ph_3$.

15.4 Microkinetic simulation for the reaction using dcpe ligand (conditions: Table 1, entry 10)

Initial concentrations:

**I**	$\mathbf{I_{TPO}}$	**A**	**1**	**2**	^{t}**BuOLi**	**Volume**
0.0025 M	0.0 M	0.0025 M	0.1 M	0.15 M	0.2 M	3 mL

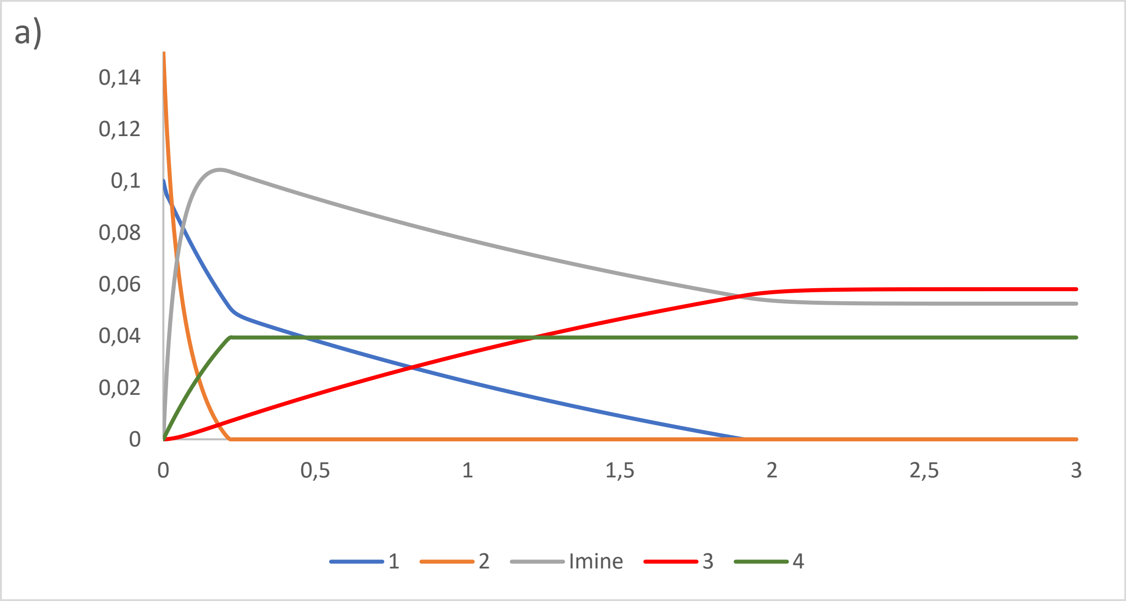

Observations: According to NMR experiments, partial ligand dissociation leads to the formation of a ~1:1 mixture of **A** and **I**. Under these conditions, C-C coupling product **3** and C-N coupling product **4** are formed in a 1.5:1 ratio. This is in accordance with the experimental result.

15.5 Microkinetic simulation for the reaction using dcpe ligand and $P(O)Ph_3$ as catalytic additive (conditions: Table 1, entry 12)

Initial concentrations:

**I**	**I_{TPO}**	**A**	**1**	**2**	**^{t}BuOLi**	**Volume**
0.0 M	0.0025 M	0.0025 M	0.1 M	0.15 M	0.2 M	3 mL

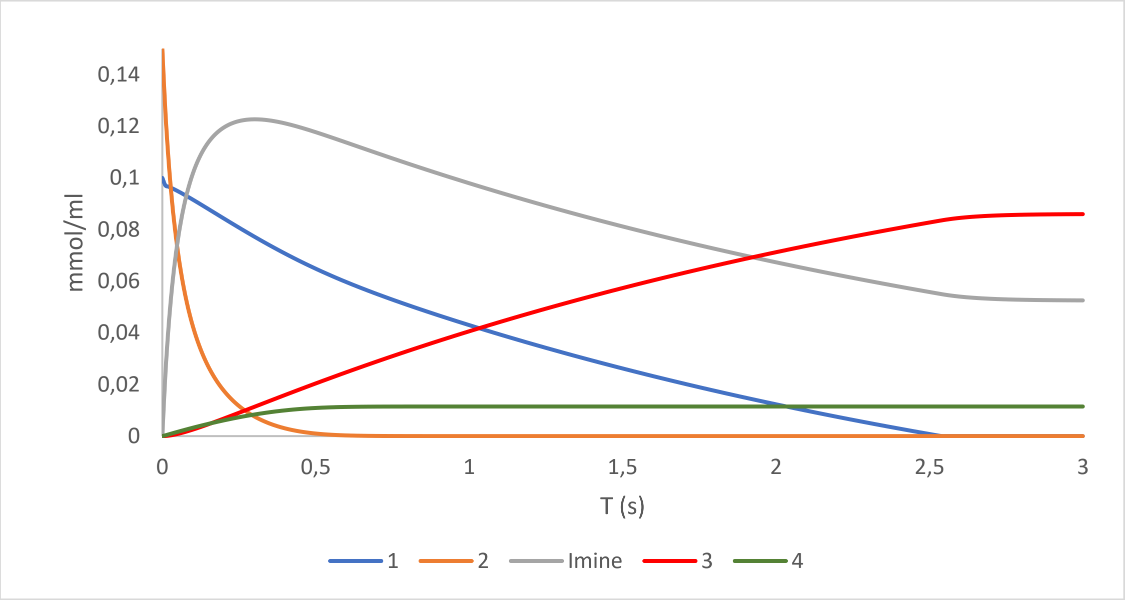

<u>*Observations:*</u> According to NMR experiments, **I_{TPO}** would form by coordination of $P(O)Ph_3$ to species **I**. Assuming a 1:1 mixture of **A** and **I_{TPO}**, the simulation shows that under these conditions C-C coupling product **3** is almost exclusively formed (**3**:**4**, 7.5:1 ratio). This result demonstrates the inhibiting activity of the catalytic Lewis base.
